# A reverse vaccinology approach to the identification and characterization of *Ctenocephalides felis* candidate protective antigens for the control of cat flea infestations

**DOI:** 10.1186/s13071-018-2618-x

**Published:** 2018-01-18

**Authors:** Marinela Contreras, Margarita Villar, Sara Artigas-Jerónimo, Lidiia Kornieieva, Sergіі Mуtrofanov, José de la Fuente

**Affiliations:** 1 SaBio. Instituto de Investigación en Recursos Cinegéticos IREC-CSIC-UCLM-JCCM, Ronda de Toledo s/n, 13005 Ciudad Real, Spain; 2 Acro Veterinary Laboratories, 15a Privokzalna Street, Pilipovichi village, Kyiv region Ukraine; 3 Department of Veterinary Pathobiology, Center for Veterinary Health Sciences, Oklahoma State University, Stillwater, OK 74078 USA

**Keywords:** Flea, Immunology, Proteomics, Transcriptomics, Vaccine, Vaccinology

## Abstract

**Background:**

Despite the abundance of the domestic cat flea, *Ctenocephalides felis* (Bouché, 1835) and disease risks associated with them, flea control is difficult and requires the development of new control interventions such as vaccines. In this study, a reverse vaccinology approach was designed to achieve a rational selection of cat flea candidate protective antigens.

**Methods:**

Based on transcriptomics and proteomics data from unfed adult fleas it was possible to select more specific candidate protective antigens based on highly represented and functionally relevant proteins present in the predicted exoproteome. The protective capacity of the recombinant antigens was evaluated for the control of *C. felis* infestations in vaccinated cats.

**Results:**

Vaccination with recombinant antigens induced an antibody response in immunized cats. Furthermore, a correlation was obtained between the effect of vaccination (antibody levels) and vaccine efficacy on flea phenotype (egg hatchability). The results suggested that the main effect of vaccination with these antigens was on reducing cat flea egg hatchability and fertility, with an overall vaccine efficacy of 32–46%. Although vaccination with these antigens did not have an effect on flea infestations, vaccines affecting reproductive capacity could reduce cat flea populations, particularly under conditions of direct insect transmission between cats.

**Conclusions:**

These results support the development of vaccines with protective antigens affecting flea reproduction and development after feeding on immunized animals for the control of cat flea infestations.

**Electronic supplementary material:**

The online version of this article (10.1186/s13071-018-2618-x) contains supplementary material, which is available to authorized users.

## Background

The domestic cat flea, *Ctenocephalides felis* (Bouché, 1835) (Siphonoptera: Pulicidae), is one of the most abundant and widespread flea species worldwide [1]. Cat fleas together with other flea species such as *Ctenocephalides canis* and *Pulex irritans* cause direct damage to the skin, discomfort, nuisance, allergic reactions, anemia, and may transmit pathogens such as *Yersinia pestis* (plague), *Rickettsia typhi* (murine typhus), *Rickettsia felis* (murine typhus-like illness) and *Bartonella* spp. (cat-scratch disease) that are of public health importance [1–6]. Fleas of pets are also competent intermediate hosts of the tapeworm *Dipylidium caninum* (pulicosis) and the filarial nematode *Acanthocheilonema reconditum* (subcutaneous infection in animals and ocular disease in humans) [1, 7]. These fleas have a low degree of species-specificity, being able to infest humans, companion animals and wildlife [1].

Despite flea abundance and disease risks associated with them, flea control is difficult and requires integrated approaches combining safe and effective insecticides such as insect development inhibitors (e.g. Lufenuron), juvenile hormone analogues (e.g. Pyriproxyfen and S-methoprene), and adulticides (e.g. Fipronil, Dinotefuran, Spinetoram, Spinosad, Selamectin) available to be used either on the animal or in the environment, or both [1, 8–13]. Additionally, resistance or reduced susceptibility has been reported for some of these compounds [1, 14–16], suggesting the need for alternative control methods.

Vaccination is an environmentally friendly alternative for the control of vector infestations and pathogen infections that allows control of several vector-borne diseases by targeting their common vector [17–20]. Vaccines have several advantages over pesticides including (i) no contamination of the environment and animal products; (ii) avoiding selection of pesticide resistant arthropod vectors; (iii) targeting a broad but selective range of vector species; and (iv) reducing vector competence for pathogen transmission [20]. The experience with the only commercial vaccines available for the control of ectoparasite infestations and containing *Rhipicephalus microplus* BM86 or BM95 antigens demonstrated that these vaccines control cattle tick populations while reducing acaricide applications [17, 19]. Recently, vaccination with Subolesin/Akirin suggested the possibility of developing universal vaccines for the control of multiple ectoparasite vector species including ticks, mosquitoes, sand flies, sea lice and poultry red mite [19, 21, 22].

Few experiments have been conducted to develop vaccines against *Ctenocephalides* spp. fleas with conflicting but promising results [23–25]. The hypothesis behind vaccine action is that vectors feeding on immunized hosts ingest antibodies specific for the target antigen that could reduce its levels and biological activity and/or interact with conserved epitopes in other proteins resulting in reduced feeding, developmental and reproductive performance [18–20, 26, 27]. However, cell-mediated immunity may also play a role in vector control [25].

One of the limiting steps in the development of vaccines for the control of ectoparasite vectors is the identification of protective antigens [19, 22, 28]. Recent developments in vector genomics and the application of systems biology to the study of vector-host-pathogen interactions have advanced our understanding of the genetic factors and molecular pathways involved at the host-vector-pathogen interface, which can lead to discovery of candidate protective antigens [19, 20, 22, 29–36]. However, very limited information is available for *Ctenocephalides* spp. fleas [32, 37–40]. For these flea species, genome sequence is not available (https://www.vectorbase.org/search/site/Ctenocephalides), and as of May 2017 only 5869 ESTs, 7230 nucleotide sequences, and 432 (GenBank) or 271 (Uniprot) protein sequences were available in the GenBank or Uniprot databases. Therefore, the identification of cat flea candidate protective antigens may be more feasible using de novo generated mRNA and protein datasets.

Reverse vaccinology focuses on a certain group of genes/proteins identified based on the use of genome-scale or omics technologies and bioinformatics followed by the screening of protective antigens for the development of next-generation vaccines [31, 34, 41]. This translational research, in which basic biological information on vectors or pathogens translates into the identification and subsequent evaluation of new candidate protective antigens, have been successful in research for anti-bacterial and anti-tick vaccines [34, 42].

Herein, we used a reverse vaccinology approach using transcriptomics and proteomics data for the identification of candidate protective antigens in the cat flea putative exoproteome (secreted and membrane proteins). Candidate protective antigens were characterized in cats vaccinated and experimentally infested with cat fleas. The results showed that the main effect of vaccination with these antigens was on reducing cat flea eggs hatchability and fertility, with an overall vaccine efficacy of 32–46% on the control of cat flea infestations by considering the cumulative effect on the different developmental stages.

## Methods

### Cat fleas

**Fig. 1:**
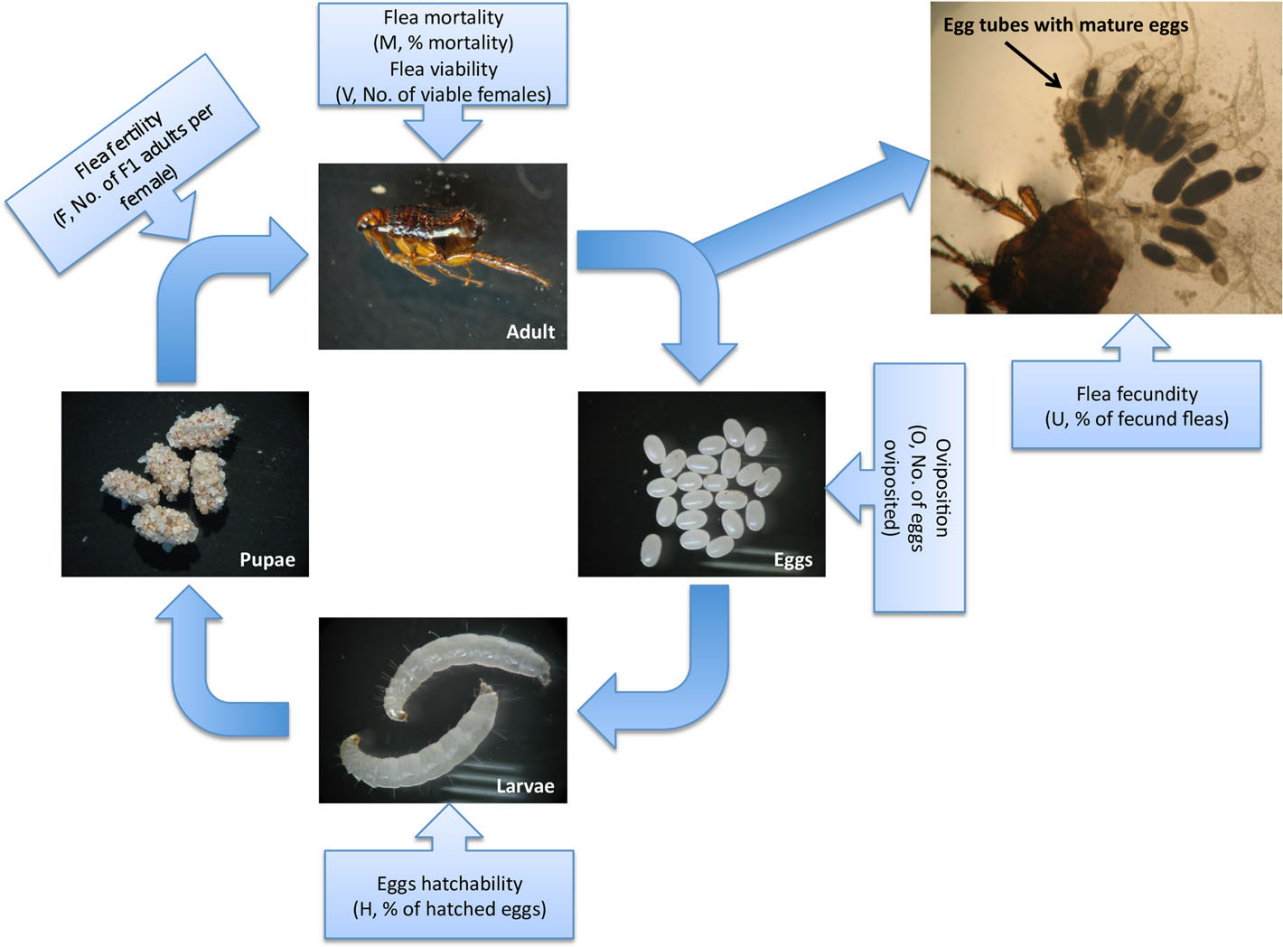
Cat flea (*Ctenocephalides felis*) life cycle and vaccine efficacy. Representative images of different cat flea developmental stages were taken from the laboratory colony maintained at the LLC ACRO Vet Lab (Kyiv region, Ukraine) and used in this study. Vaccine efficacy (E) was determined by considering flea mortality (M, % mortality), flea fertility (F, No. of F1 adults per female), oviposition (O, No. of eggs oviposited), eggs hatchability (H, % of hatched eggs), flea viability (V, No. of viable females), and flea fecundity (U, % of fecund fleas)

Domestic cat fleas (*C. felis*) used in this study were laboratory-reared at the LLC ACRO Vet Lab (Kyiv region, Ukraine) using a European cat flea strain maintained at the Laboratory of Ecology and Toxicology of Taras Shevchenko National University of Kyiv as a closed colony since March 2003. Since 2008 (51st generation), the flea colony was maintained at LLC ACRO Vet Lab and standardized by main morphophysiological indicators [43] (Fig. 1). Fleas were cultivated in a climate chamber with a 24 h dark cycle at 21 ± 1 °C and a relative humidity of 81 ± 2%. To support that the flea colony behave as natural fleas, each flea generation was evaluated for main morphophysiological indicators such as mean weight of females and males, cycle period, male/female percentage ratio, and number of egg tubes in females. Adult mixed breed shorthair cats were used to feed adults. Egg collection began on the 2nd-3rd day after feeding and continued daily during 3 weeks. Larvae were fed using a dried bovine blood based feeding medium, which also contains baker’s yeast, trefoil powder and potato farina mixed with coarse-grained sand, and added to eggs at a proportion of 2 mg medium per egg.

### Cat flea RNA and protein preparation

Mixed female and male (sex ratio approximately 50:50) unfed fleas of similar age were used. Approximately 150 μg fleas were pulverized in liquid nitrogen and used for RNA and protein extraction. RNA was purified using the RNeasy MinElute Cleanup Kit (Qiagen, Valencia, CA, USA) and characterized using the Agilent 2100 Bioanalyzer (Santa Clara, CA, USA) as previously described [44]. RNA concentration was determined using the Nanodrop ND-1000 (NanoDrop Technologies Wilmington, DE, USA). Protein extraction was conducted as described by Villar et al. [44]. Briefly, cat flea samples were homogenized in STM solution (0.25 M sucrose, 1 mM MgCl_2_, 10 mM Tris-HCl, pH 7.4) supplemented with complete mini protease inhibitor cocktail (Roche, Basel, Switzerland) (10 ml/g tissue), sonicated-vortexed for 3 cycles, and centrifuged at 260× *g* for 5 min at room temperature to remove cellular debris. The supernatant was then centrifuged at 13,000× *g* for 30 min at 4 °C and the pellet fraction enriched in crude plasma membranes was collected, resuspended in 150 μl STM solution supplemented with 0.7% *n*-Dodecyl β-D-maltoside (DDM) and 0.5% amidosulfobetaine-14 (ASB14) detergents (Sigma-Aldrich, St. Louis, MI, USA), incubated on a shaker for 1 h at 4 °C (vortex of 5 s after 15 min periods) and centrifuged at 13,000× *g* for 30 min at 4 °C. The pellet was stored at -80 °C for further processing and protein concentration of the soluble plasma membrane was determined using the BCA Protein Assay (Thermo Scientific, San Jose, CA, USA) using BSA as a standard. Soluble plasma membrane proteins were precipitated in aliquots of 150 μg following the methanol/chloroform procedure [45], dried and also stored at -80 °C until used.

### Transcriptomics data acquisition and analysis

Purified RNA was used for library preparation using the TruSeq RNA sample preparation kit v.1 and the standard low throughput procedure (Illumina, San Diego, CA, USA) [44]. Briefly, messenger RNA from 3 μg total RNA was captured using poly-dT magnetic beads and polyA+ RNA was chemically fragmented and used for library preparation for Illumina sequencing. Fragmentation time was reduced to 1 min in order to recover fragments of an increased size that may facilitate assembly of pair-end reads. With this procedure we obtained 60% the RNA library corresponding to a size above 200–600 bp (excluding adapters), as compared to the standard procedure, where the higher percentage of RNA (typically 70%) is in the 100–200 bp range. RNA was then used for cDNA synthesis and remaining RNA was removed, following an end repair procedure and preparation of double-stranded cDNA for adaptor ligation as previously described [44]. An enrichment procedure based on PCR was then performed to ensure that all molecules in the library conserved the adapters at both ends. The number of PCR cycles was adjusted to 10 and the final amplified library was checked again on a BioAnalyzer 2100 (Agilent, Santa Clara, CA, USA). Finally, the library was titrated by quantitative PCR using a reference standard for quantification, showing a final concentration of 201.65 nM.

The library was denatured and seeded on a HiSeq flowcell (Illumina; Parque Científico de Madrid, Madrid, Spain), where clusters were formed and sequenced using a 2 × 100 pair-end sequencing protocol. Quality filtering was automatically performed according to Illumina specifications and a total number of 71.9 million pair-end, index-selected, pass-filter sequences were obtained. The percentage of pass-filter reads with a quality above of Q30 was higher than 90% and used for de novo transcript assembly. Sequence reads were trimmed at the error probability higher than 0.05 and reads with at least one N (unknown nucleotide) were filtered out. The remaining pairs of reads were assembled only when the two members of the pair remained after filtering and trimming. As previously described [44], Oases [46] was used for read assembly using a K = 81 value very close to the total length of the read (~100 bp) to avoid misassembly. Transcripts were clustered by similarity to a set of reference proteins to build Unigenes. In order to have a uniform similarity distance between Unigenes, these reference proteins were UniRef90 [47] representative proteins. Reference proteins were used for transcript clustering to obtain a protein-centered analysis of gene expression that is more useful for functional analysis of a de novo transcriptome.

Functional annotations were done based on Uniprot reference proteins using Blast e values < 10E-10. A set of 158,072 reference proteins downloaded from Uniprot on September 2013 was selected, and included all proteins that were representative of Uniref90 clusters belonging to the taxa Siphonaptera, Strepsiptera, Neuropterida, Mecoptera, Hymenoptera and Coleoptera*.* All these taxa are included in the taxonomic node Endopterygota. Considering the availability of annotations, *Drosophila melanogaster* proteins were also included. A set of 13,654 reference proteins from *Bartonella*, *Yersinia*, *Dipylidium* and *Rickettsia* were included to exclude these sequences from further analysis because *C. felis* could carry these microorganisms [1, 3, 4, 7]. The single nucleotide polymorphisms (SNPs) calling was performed by mapping filtered reads to the transcripts with Bowtie tool [48] and then calling the SNPs with samtools, bcftools and vcftools [49]. The variants were filtered by selecting a minimum number of 2 alternate bases, a maximum of 3 bp around a gap and a window size of 10 bp for filtering adjacent gaps. For the selection of putative Unigenes encoding secreted and membrane proteins in the exoproteome (secreted and membrane proteins), a semi-automated strategy was used by searching first with a script the terms “membrane”, “signal peptide”, “secreted” and “secretion” in all the Uniprot functional annotation fields (name, keywords, features, functional comments, gene ontology (GO) annotation) and then manually filtering the results. GO annotation for biological process (BP), molecular function (MF), and cellular component (CC) was conducted using Blast2GO software (version 3.0; www.blast2go.com).

### Proteomics data acquisition and analysis

Precipitated plasma membrane soluble and pellet fractions were loaded onto a 12% SDS-PAGE gel to concentrate the whole proteome in an unseparated protein band in the stacking/resolving gel interface, which was excised, cut into 2 × 2 mm cubes and digested overnight at 37 °C with 60 ng/μl sequencing grade trypsin (Promega, Madison, WI, USA) at 5:1 protein:trypsin (*w*/w) ratio in 50 mM ammonium bicarbonate, pH 8.8 containing 10% (*v*/v) acetonitrile [44, 50]. The resulting tryptic peptides from the gel band were extracted, desalted, onto OMIX Pipette tips C_18_ (Agilent Technologies), dried-down and stored at −20 °C until mass spectrometry analysis [44]. The desalted protein digest was resuspended in 0.1% formic acid and analyzed by reverse phase (RP)-liquid chromatography (LC)-mass spectrometry (MS)/MS (RP-LC-MS/MS) using an Easy-nLC II system coupled to an ion trap LCQ Fleet mass spectrometer (Thermo Scientific). The peptides were concentrated (on-line) by reverse phase chromatography using a 0.1 mm × 20 mm C18 RP precolumn (Thermo Scientific), and then separated using a 0.075 mm × 100 mm C18 RP column (Thermo Scientific) operating at 0.3 μl/min. Peptides were eluted using a 180 min gradient from 5 to 35% solvent B (Solvent A: 0.1% formic acid in water, solvent B: 0.1% formic acid in acetonitrile). ESI ionization was done using a Fused-silica PicoTip Emitter ID 10 μm (New Objective, Woburn, MA, USA) interface. Peptides were detected in survey scans from 400 to 1600 amu (1 μscan), followed by three data dependent MS/MS scans (Top 3), using an isolation width of 2 mass-to-charge ratio units, normalized collision energy of 35%, and dynamic exclusion applied during 30 s periods.

The MS/MS raw files were searched against the Neoptera (845,983 entries in January 2014) and Carnivora (136,353 entries in January 2014) Uniprot databases and against a database created from transcriptomics data of predicted exoproteome proteins for proteomics informed by transcriptomics (PIT) [51]. The database for PIT was developed using a program to extract all the sequence fragments corresponding to BLAST hits with actual reference proteins. We used all the transcripts found in the flea sample and all the sequences corresponding to all the BLASTX hits of these transcripts with the reference predicted exoproteome proteins (see Dataset 1 in Additional file 1). The sequences were split when the program detected stop codons (*), undefined amino acids (x) or gaps (-). The search of MS/MS raw files against these databases was conducted using the SEQUEST algorithm (Proteome Discoverer 1.3; Thermo Scientific) with the constraints previously described [44]. For peptide validation, the Proteome Discoverer 1.3 software was used by filtering based on the q-value generated by Percolator that is defined as the minimal false discovery rate (FDR) at with the identification is considered correct [44, 52, 53]. A FDR < 0.01 was considered as a condition for successful peptide assignments. Therefore, only peptides with q-values ≤ 0.01 and delta Cn > 0.15 were used for protein identification. As for transcriptomics data, GO annotation for BP, MF and CC was conducted using Blast2GO software (version 3.0; www.blast2go.com).

### Criteria for selection of candidate protective antigens

The selection of genes encoding candidate protective antigens was based on membrane proteins highly represented based on the number of peptide-spectrum matches (PSMs) selected after proteomics analysis and PIT against the database of transcripts encoding putative secreted and membrane proteins that may be functionally important for insect feeding, metabolism, development and/or reproduction. Putative secreted and membrane proteins were selected to increase and/or favor antibody-antigen interactions in fleas feeding on vaccinated hosts. Following these criteria, a total of 6 candidate protective antigens (F1-F6) were selected for further characterization (Table 1).

**Table 1:** Proteins selected as candidate protective antigens

Sequence ID	Uniprot accession No.	Description	Transcript identified	Protein levels (PSMs)
F1	C6H0K8	*C. felis* arginine kinase 1 (argk1)	Yes	43^b^
F2^a^	Q27766	*C. felis* sodium/potassium-transporting ATPase subunit alpha (Na^+^/K^+^ ATPase)	Yes	3^b^; 62^c^
F3	Q8IS50	*C. felis* serpin 4	No	4^b^
F4	Q8MZR6	*C. felis* juvenile hormone epoxide hydrolase 1 (JHEH I)	Yes	2^c^
F5	Q7KVA1 (de novo sequence locus 4025)	*Drosophila melanogaster* xylosyltransferase (oxt)	Yes	2^c^
F6	Q9VAF0 (de novo sequence locus 4149)	*Drosophila melanogaster* zinc transporter ZIP13 homolog (Zip99C)	Yes	2^c^

^a^The F2 antigen could not be produced in *E. coli* and was not included in the vaccination trial

^b^Identified after searching MS/MS raw files against the Neoptera Uniprot database

^c^Identified by PIT using a database created from transcriptomics data of predicted transmembrane and secreted proteins

### Cloning of cat flea genes encoding candidate protective antigens and production of recombinant proteins

Coding sequences for selected candidate protective antigens were amplified by RT-PCR using sequence-specific primers (F1-F4) or synthesized (GenScript, Hong Kong) with optimized codon usage for *Escherichia coli* (F5, F6) (see Datasets 2 and 3 in Additional file 2). The DNA coding sequences were cloned into the expression vector pET101 and expressed in *Escherichia coli* strain BL21 as previously described [54]. Recombinant proteins produced using this expression system were fused to Histidine tags for purification by affinity to Ni^2+^ [27, 54, 55]. Transformed *E. coli* strains were induced with isopropyl β-D-1-thiogalactopyranoside (IPTG) for 4.5 h to produce recombinant proteins, which were purified to > 95% of total cell proteins by Ni affinity chromatography (Genscript Corporation, Piscataway, NJ, USA) as previously described [27, 54, 55] using 1 ml HisTrap FF columns mounted on an AKTA-FPLC system (GE Healthcare, Piscataway, NJ, USA) in the presence of 7 M urea lysis buffer.

### Vaccine formulations

The purified denatured recombinant proteins were refolded by dialysis against 1000 volumes of PBS (137 mM NaCl, 2.7 mM KCl, 10 mM Na_2_HPO_4_, 1.8 mM KH_2_PO_4_), pH 7.4 for 12 h at 4 °C. Recombinant proteins were then concentrated using an Amicon Ultra-15 ultrafiltration device (cut off 10 kDa) (Millipore-Merck, Darmstadt, Germany), and adjusted to 0.5 mg/ml. For vaccine formulation, recombinant proteins or PBS saline control were adjuvated in Montanide ISA 50 V2 (Seppic, Paris, France) to a final protein concentration of 250 μg/ml as previously described [54, 55].

### Cat vaccination and flea infestation

Domestic cats (*Felis catus*) were used in this study. Cats were healthy males and females of mixed breeds, older than 6 months, not pregnant or lactating and that have not been treated with an acaricide/insecticide for at least three months before the start of the study. Only cats that kept a minimum of 50% fleas at pre-test infestation were used in the study. All animals were chip identified. Cats were randomly assigned to 6 study groups with 3 cats each. Immunizations, cat flea infestations, collections and evaluations were done blinded and the key to the experimental groups was not disclosed until the end of the experiment. Cats were kept in individual cages placed in the same room and maintained in a 12 h light/12 h dark cycle. Each cage had a sleeping area, one manger, one drinking dish, and toilet mat with absorbent. Combined extract and input ventilation was provided. Animals were acclimatized for 9 days prior to first vaccination. Then, cats were injected subcutaneously (dorsum between shoulders) at days 0 (T1) and 14 (T2) with 0.5 ml (50 μg) doses of recombinant protein vaccines or adjuvant/saline control using a syringe with removable needle. Two weeks after the last immunization (day 28; T3), cats in vaccinated and control groups and wearing Elizabethan collars were infested with 100 (70% females: 30% males) adult unfed fleas.

### Vaccine efficacy

Vaccine efficacy (E) was determined by considering flea mortality (M), flea fertility (F), oviposition (O), eggs hatchability (H), flea viability (V), and flea fecundity (U) (Fig. 1). On day 32, cat cages were thoroughly cleaned. Then on days 33–37, flea eggs were daily collected, counted to calculate O, and placed in glass jars with feeding medium and sand at 21 ± 1 °C and 81 ± 2% relative humidity (RH) with a 24 h dark cycle for approximately one month until F1 generation adults appeared. Flea F was calculated by dividing the number of adult F1 fleas on each jar by the amount of viable female fleas collected from cats on days 36–38. Flea eggs H was calculated by determining the percent of hatched larvae obtained from 25 eggs collected from each cat and incubated for 4–7 days in Petri dishes at 21 ± 1 °C and 81 ± 2% RH with a 24 h dark cycle. Groups of 6 cats each (one cat from each study group per day) were sedated and combed on days 36–38 to collect adult fleas, which were placed in individual glass jars for each cat. A fine-toothed flea comb was used to recover fleas present in the animal’s fur. Combing was conducted by several strokes of the comb in various areas of the animal, each time moving in the same direction following the pattern of the hair coat. Movement, from one part of the animal’s fur to the next was done via strokes overlapping each other, so that no area of fur was left uncombed. Flea M and V were calculated by counting the number of adult and female fleas collected from each cat to determine the percent mortality and number of viable females, respectively. To calculate flea U, 10 viable female fleas from each cat were dissected and the condition of the first two oocytes in egg tubes was determined under binocular optical microscope MOTIC SFC-11/12 series. Fleas were considered fecund when the presence of mature eggs and/or chorion was observed at the base of the egg tube.

Adapting the method used to calculate vaccine E for tick vaccines [19, 20], cat flea vaccine E was calculated as E = 100 (l - ∏_*k*=*1 to n*_ a_*k*_), where a_*k*_ represent the reduction in the studied developmental processes (*k*) in cat fleas fed on vaccinated cats when compared to the controls fed on adjuvant/saline injected cats. The M, H and U were compared between groups by a Fisher’s exact test (*P* = 0.05; http://www.socscistatistics.com/tests/fisher/Default2.aspx). Additionally, data for all variables were compared between fleas fed on vaccinated and control cats by Student’s t-test with unequal variance (*P* = 0.05).

### Analysis of cat IgG antibody response by ELISA

Blood samples were collected from each cat before immunization (T1), before flea infestation (T3), and after adult flea counts at days 36–38 (T4) into sterile tubes and maintained at 4 °C until serum was separated by centrifugation and stored at -20 °C. An indirect ELISA test was performed to detect IgG antibodies against recombinant proteins in serum samples from vaccinated and control cats collected at T1, T3 and T4 as described previously [27, 56]. High absorption capacity polystyrene microtiter plates were coated with 50 μl (0.02 μg/ml solution of purified recombinant proteins) per well in carbonate-bicarbonate buffer (Sigma-Aldrich). After an overnight incubation at 4 °C, coated plates were blocked with 200 μl/well of blocking solution (5% skim milk in PBS). Serum samples or PBS as negative control were diluted (1:100, 1:500, 1:1000 and 1:3000, *v*/v) in blocking solution and 50 μl/well were added into duplicate wells of the antigen-coated plates. After an overnight incubation at 4 °C, the plates were washed three times with a washing solution (PBS containing 0.05% Tween 20). A goat anti-cat IgG-peroxidase conjugate (Sigma-Aldrich) was added (diluted 1:500 in blocking solution) and incubated at room temperature (RT) for 1 h. After three washes with washing solution, 200 μl/well of substrate solution (Fast OPD, Sigma-Aldrich) was added. Finally, the reaction was stopped with 50 μl/well of 3 N H_2_SO_4_ and the optical density (OD) was measured in a spectrophotometer at 450 nm. Antibody levels in vaccinated and control rabbits were expressed as the OD_450nm_ (OD_cat sera_ – OD_PBS control_) and compared between vaccinated and control groups by ANOVA test (*P* = 0.05). A correlation analysis was conducted in Microsoft Excel (version 12.0) between the antibody levels (OD_450nm_ at 1:500 dilution of primary antibodies) at time of flea infestations (T3) and H or F in individual vaccinated and control cats to correlate the effect of vaccination on eggs hatchability or flea fertility with antibody levels. The Pearson’s correlation coefficient was calculated using the Pearson’s Correlation Coefficient Calculator (http://www.socscistatistics.com/tests/pearson/Default2.aspx) (*r* = -0.5).

### Analysis of cat IgG antibody response by western blot

Western blot analysis was conducted as previously described [54]. Briefly, 10 μg of purified recombinant proteins were separated by electrophoresis in an SDS-12% polyacrylamide gel (Life Science, Hercules, CA, USA), stained with Coomassie Brilliant Blue (Sigma-Aldrich) or transferred to a nitrocellulose membrane. The membrane was blocked with 5% BSA (Sigma-Aldrich) for 2 h at RT and washed four times with TBS (50 mM Tris-Cl, pH 7.5, 150 mM NaCl, 0.5% Tween 20). Pooled sera collected at T3 from vaccinated cats were used as primary antibodies at a 1:500 dilution in TBS, and an anti-cat IgG-horseradish peroxidase (HRP) conjugate (Sigma-Aldrich) diluted 1:1000 in TBS with 3% BSA was used as secondary antibodies. The membrane was finally washed five times with TBS and developed with TMB (3,3′, 5,5′- tetramethylbenzidine) stabilized substrate for HRP (Promega, Madrid, Spain) according to the manufacturer recommendations.

### Determination of cat flea mRNA levels by real-time RT-PCR

The expression of selected genes was characterized using total RNA extracted from midguts dissected from unfed and fed cat fleas. Total RNA was extracted using TriReagent (Sigma-Aldrich) following manufacturer’s recommendations. Real-time RT-PCR was performed on RNA samples using gene-specific forward (Fw) and reverse (Rv) oligonucleotide primers (F1-Fw: 5′-CAT GCA AAT GGG AGG AGA TT-3′, F1-Rv: 5′-GCG GTT AGC AGC CAA CTT AG-3′; F3-Fw: 5′-CAA AGC CGG TGA TCT AGA CC-3′, F3-Rv: 5′-GCA ATT CTA CAG CCT TGG CA-3′; F4-Fw: 5′-TGG CCA GGA TCT TTT GTG GA-3′, F4-Rv: 5′-ATC ATA GAA CCC CAG TCG CC-3′; F5-Fw: 5′-TGG GGA GGT GCA TCT TTA CT-3′, F5-Rv: 5′-TGT ACT TCT CGC CCA TGT GA-3′; F6-Fw: 5′-TTT CAC ACA CGG TCT TGC TG-3′, F6-Rv: 5′-CTT CCA TTG CAC TCG TAG CC-3′) and the iScript One-Step RT-PCR Kit with SYBR Green and the CFX96 Touch Real-Time PCR Detection System (Bio-Rad, Hercules, CA, USA). A dissociation curve was run at the end of the reaction to ensure that only one amplicon was formed and that the amplicons denatured consistently in the same temperature range for every sample. The mRNA levels were normalized against cat flea 18S rRNA (GenBank: AF136859; Cf18SFw: 5′-TGC TCA CCG TTT GAC TTG G-3′, Cf18SRv: 5′-GTT TCT CAG GCT CCC TCT CC-3′) [57] using the genNorm method (ddCT method as implemented by Bio-Rad iQ5 Standard Edition, version 2.0). Normalized Ct values were compared between unfed and fed fleas by Student’s t-test with unequal variance (*P* = 0.05; *n* = 3 biological replicates).

## Results

### Selection of candidate protective antigens

**Fig. 2:**
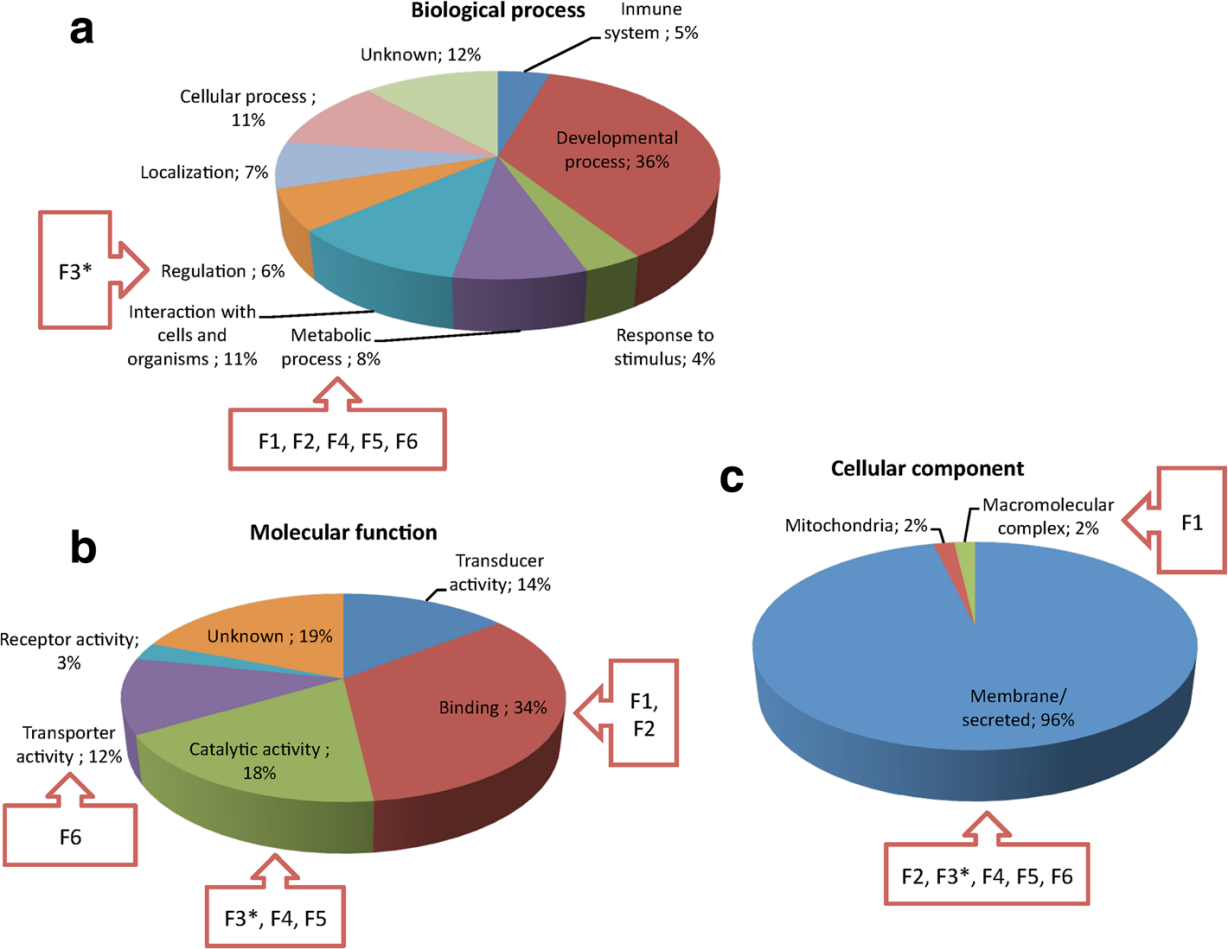
Transcripts in unfed cat flea encoding putative transmembrane and secreted proteins. Transcripts in cat flea encoding proteins with putative transmembrane and signal peptide in the exoproteome (*n* = 177) were functionally annotated and grouped according to the BP (**a**), MF (**b**) and CC (**c**) of the encoded proteins. The percent of proteins on each category is shown. GO annotation for BP, MF and CC was conducted using Blast2GO software (version 3.0; www.blast2go.com). Proteins selected as candidate protective antigens (F1-F6; Table 1) are shown. *The transcript for F3 was not identified in the cat flea transcriptome (Table 1), but was included in the figure to compare with other candidate protective antigens

After RNAseq, 71,871,801 illumina 100 bp paired-end reads (7,259,051,901 bp) from the flea samples were pre-processed. Finally, a total of 68,351,915 pre-processed 100 bp paired-end reads were de novo assembled obtaining 59,558 transcripts. These transcripts were annotated and clustered by similarity to Uniprot proteins, building a set of 11,627 Unigenes (see Dataset 4 in Additional file 3). The analysis of predicted transcripts encoding proteins in the exoproteome resulted in 1620 Unigenes, of which 67 and 110 encoded for predicted proteins with signal peptide and transmembrane regions, respectively (see Dataset 4 in Additional file 3). Transcripts in cat flea encoding proteins with putative transmembrane and signal peptide in the exoproteome (*n* = 177) were functionally annotated, and the most represented GO annotations corresponded to developmental process (BP), binding (MF) and membrane/secreted proteins (CC) (Fig. 2a-c).

In the antigen selection process, the analysis of polymorphisms of the candidate genes is crucial since the vaccine has to be designed to be efficacious for the majority of the target individuals. The analysis of SNPs in the set of transcripts provided a specific view of the genetic diversity and allowed to take into account the polymorphisms of the candidates from the first stages of the vaccine design process. However, it is important to note that, in some cases, different transcripts of the same locus could be actual SNPs. If two transcripts have the same sequence but differ in one position (or in several positions) they could be considered by the de novo assembler (Oases in this case) as different transcripts and hence, some interesting variants could be hidden as different transcripts of the same locus.

**Fig. 3:**
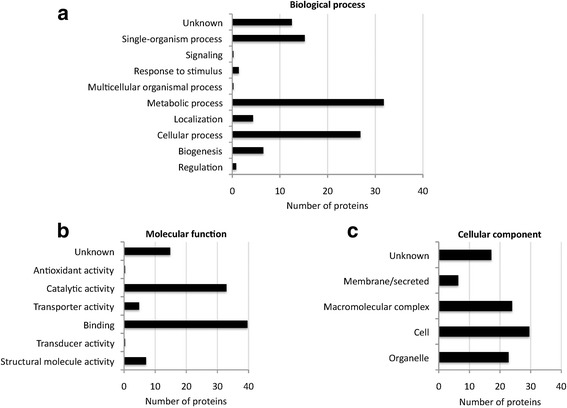
Insect proteins identified in unfed cat flea plasma membrane. Proteomics of plasma membrane soluble and pellet fractions resulted in 218 flea proteins when MS/MS raw files were searched against the Neoptera and Carnivora Uniprot databases. The proteins were functionally annotated and grouped according to the BP (**a**), MF (**b**) and CC (**c**). The same protein could have several GO annotations, a fact that was included in the results shown in the graphs. GO annotation for BP, MF and CC was conducted using Blast2GO software (version 3.0; www.blast2go.com)

**Fig. 4:**
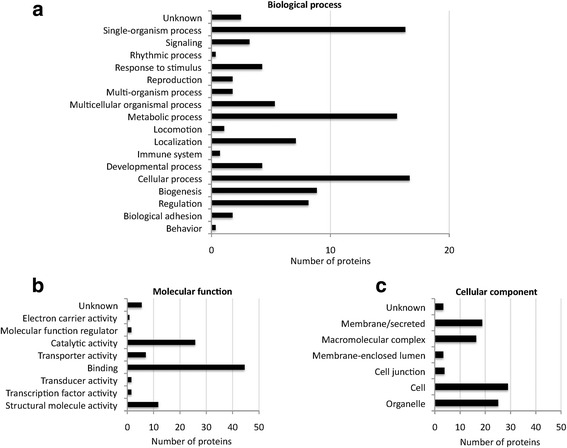
Host proteins identified in unfed cat flea plasma membrane. Proteomics of plasma membrane soluble and pellet fractions resulted in 82 host proteins when MS/MS raw files were searched against the Neoptera and Carnivora Uniprot databases. The proteins were functionally annotated and grouped according to the BP (**a**), MF (**b**), and CC (**c**). The same protein could have several GO annotations, a fact that was included in the results shown in the graphs. GO annotation for BP, MF and CC was conducted using Blast2GO software (version 3.0; www.blast2go.com)

Proteomics of plasma membrane soluble and pellet fractions resulted in 218 flea and 82 host proteins when MS/MS raw files were searched against the Neoptera and Carnivora Uniprot databases, respectively (see Dataset 5 in Additional file 4). Flea and host derived proteins were functionally annotated. The most represented GO annotations for cat flea proteins corresponded to metabolic process (BP), binding (MF) and cell proteins (CC) (Fig. 3a-c). For host proteins, the most represented GO annotations corresponded to single-organism and cellular processes (BP), binding (MF) and cell proteins (CC) (Fig. 4a-c). The PIT using a database created from transcriptomics data of predicted secreted and membrane proteins resulted in 11 identifications corresponding to 7 different proteins in the plasma membrane (see Dataset 5 in Additional file 4).

An algorithm was developed to select genes encoding candidate protective antigens. The selection was based on membrane proteins highly represented based on the number of peptide-spectrum matches (PSMs) selected after proteomics analysis and PIT against the database of transcripts encoding putative secreted and membrane proteins that may be functionally important for insect feeding, metabolism, development and/or reproduction (see Dataset 4 in Additional file 3 and Figs. 2a-c and 3a-c). Secreted and membrane proteins are part of the exoproteome, and were selected to increase and/or favor antibody-antigen interactions in fleas feeding on vaccinated hosts. Following these criteria, a total of 6 candidate protective antigens (F1-F6) were selected for further characterization (see Dataset 2 in Additional file 2 and Table 1). Four of them (F1-F4; Table 1) were reported before in *C. felis* while for F5 and F6 (Table 1) de novo sequence assembly was conducted to obtain coding sequences for the cat flea (see Dataset 3 in Additional file 2).

### Characterization of cat flea candidate protective antigens

The selected candidate protective antigens were classified in different BP (Fig. 2a), MF (Fig. 2b) and CC (Fig. 2c) GO. However, most of the selected antigens were involved in metabolic processes with catalytic and binding functions, and located in the cell membrane or secreted (Fig. 2a-c). These candidate protective antigens included arginine kinase (argk1) (ATP:l-arginine omega-N-phosphotransferase, EC2.7.3.3) (F1; Table 1), sodium/potassium-transporting ATPase subunit alpha (Na^+^/K^+^ ATPase), also known as Na^+^/K^+^ pump (EC 3.6.3.9) (F2; Table 1), serpin 4 (F3; Table 1), juvenile hormone epoxide hydrolase 1 (JHEH I; EC 3.3.2.9) (F4; Table 1), xylosyltransferase (oxt; EC 2.4.2.26) (F5; Table 1), and zinc transporter ZIP13 homolog (Zip99C) (F6; Table 1).

**Fig. 5:**
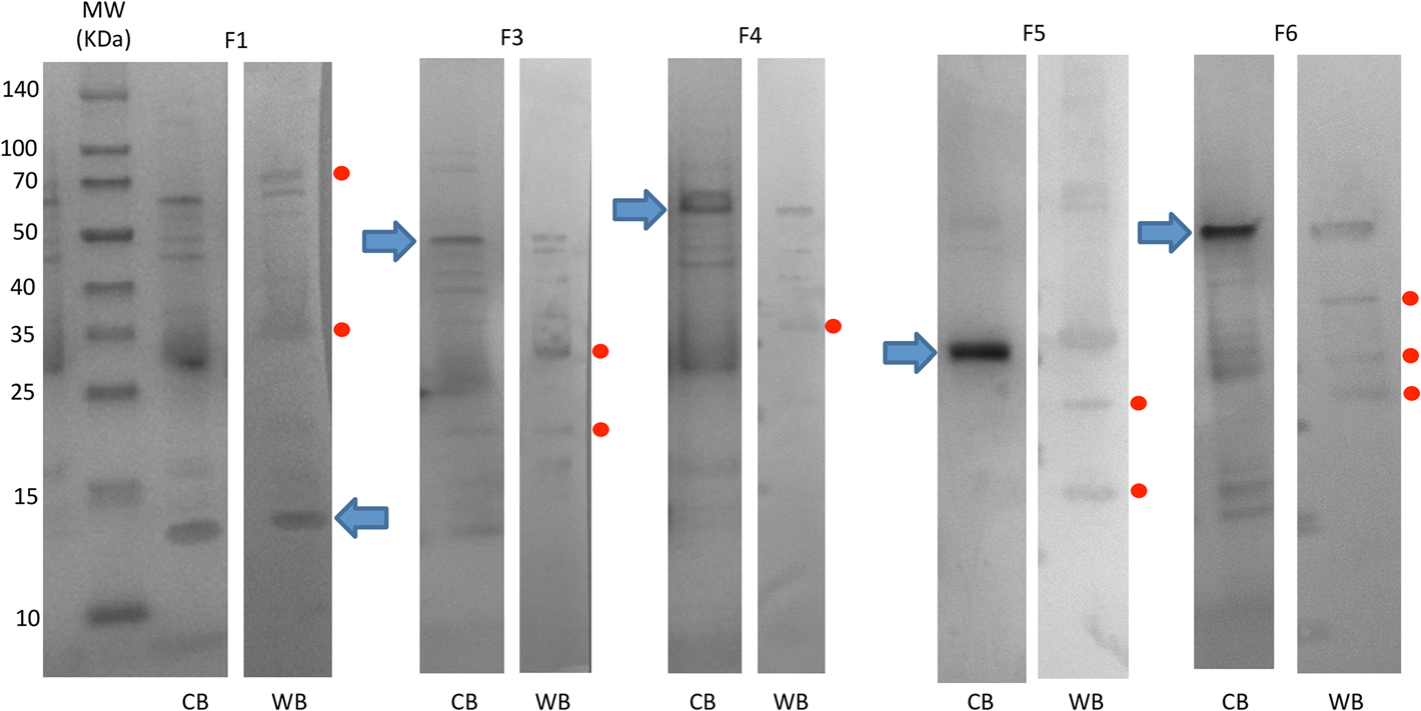
Production and characterization of recombinant cat flea proteins. Samples of recombinant proteins produced in *E.coli* were taken after purification by Ni affinity chromatography. Ten μg proteins were loaded per well in an SDS-12% polyacrylamide gel. The gel was stained with Coomassie Brilliant Blue (CB) or used for Western blot analysis (WB) using sera from vaccinated cats collected at T3. The position of the recombinant proteins is indicated with arrows. Some of the other protein bands in some of the samples correspond to *E. coli* contamination proteins, and aggregation or degradation products of the recombinant antigens (red dots). *Abbreviations*: MW, molecular weight markers (spectra multicolor broad range protein ladder; Thermo Scientific)

Once the candidate protective antigens were selected (Table 1), the objective of the study was to evaluate the protective capacity of the recombinant antigens for the control of *C. felis* infestations in vaccinated cats. Recombinant proteins were produced in *E. coli*, except for F2, which was not produced or produced at very low levels and removed from the study. Recombinant proteins showed the expected molecular weight when produced in *E. coli* and after purification by Ni^2+^ affinity chromatography (Fig. 5). Sera from vaccinated cats recognized recombinant proteins, showing that the IgG antibody response in vaccinated cats was directed against these antigens (Fig. 5).

**Fig. 6:**
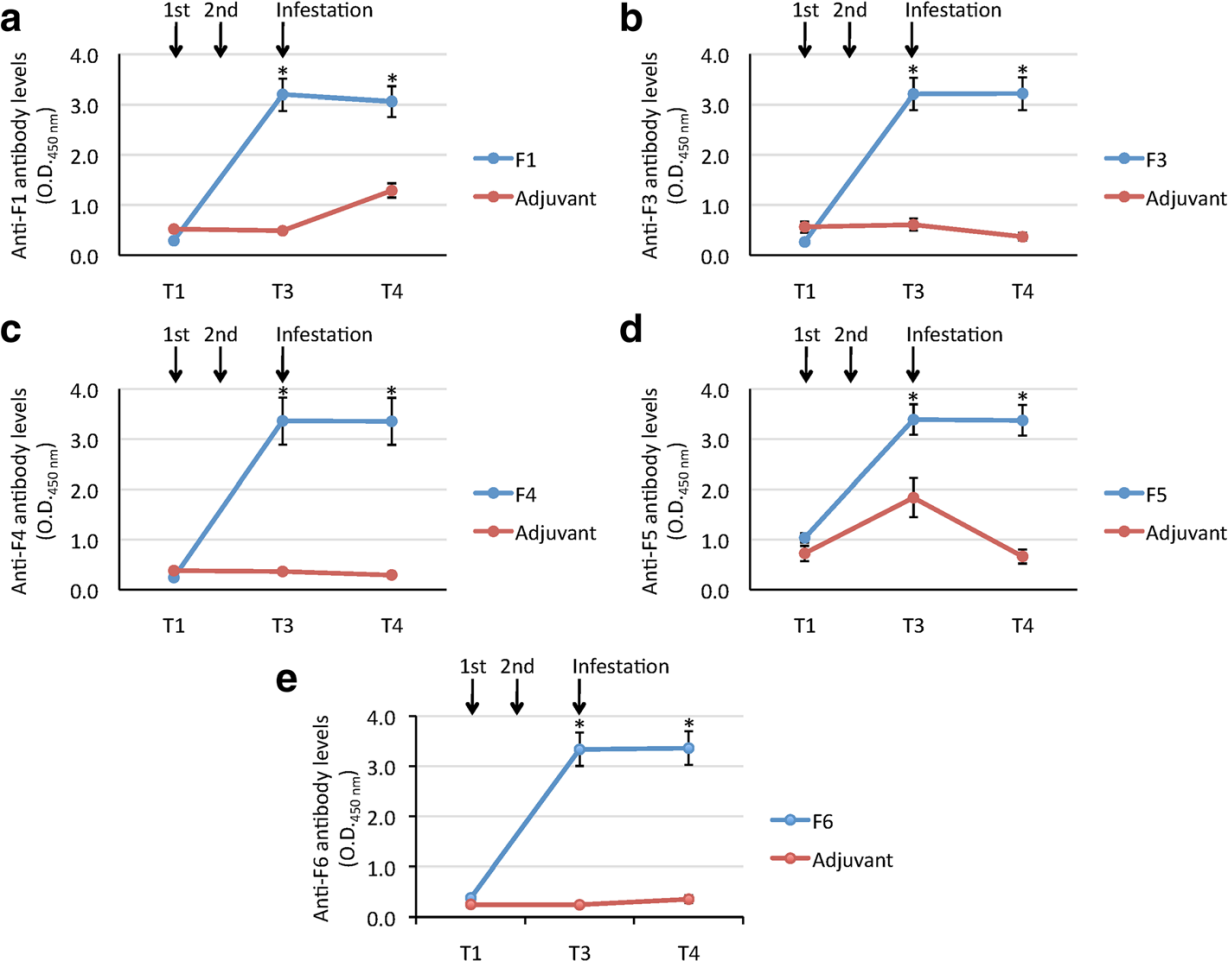
Antibody response in cats vaccinated with cat flea recombinant proteins. Antibody levels were determined by ELISA in vaccinated and control cats against the recombinant F1 (**a**), F3 (**b**), F4 (**c**), F5 (**d**) or F6 (**e**) proteins used for vaccination. Serum samples were collected before first immunization (T1) and cat flea infestations (T3), and after adult flea counts at days 36–38 (T4). Antibody levels in vaccinated cats were expressed as the average ± SD. OD_450nm_ (OD_cat sera_ – OD_PBS control_) at 1:500 dilution of primary antibodies, and compared between vaccinated and control groups by ANOVA test (*P < 0.05; *n* = 3)

**Fig. 7:**
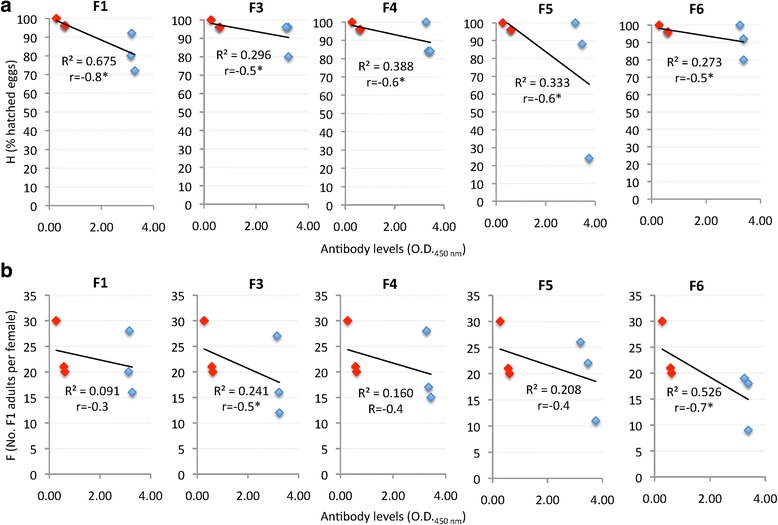
Effect of cat vaccination on flea biology. Antibody levels negatively correlated with vaccine efficacy on flea egg hatchability (**a**) and flea fertility (**b**) in cats vaccinated with recombinant antigens. The correlation analysis was conducted using Microsoft Excel (version 12.0) to compare the vaccine effects on flea biology after feeding on vaccinated (blue marks) and control (red marks) cats with antibody levels (1:500 dilution of primary antibodies) at time of flea infestation (T3). The linear correlation coefficients (*R*^2^) and Pearson correlation coefficient are shown (*r < -0.5; *n* = 6)

The IgG antibody response against cat flea recombinant proteins in vaccinated cats increased after immunization, and remained higher than in controls until T4 in all groups (Fig. 6a-e). Vaccination did not affect adult flea infestations (M) and fecundity (U) (Table 2). Vaccination with F4 and F6 antigens resulted in the reduction of cat flea oviposition (DO = 6% and 23%, respectively; Table 2), and only the F4 antigen had an effect on reducing the number of viable females (DV = 4%; Table 2). However, vaccination with all antigens reduced egg hatchability (H) and flea fertility (F) when compare to controls (Table 2). These results suggested that the main effect of vaccination with these putative exoproteome antigens was on cat flea eggs hatchability and fertility, which may results in the reduction of insect populations over time. The significance of the results was affected by animal-to-animal variations in both control and vaccinated groups (Table 2), suggesting that future trials should include more than 3 cats per group. Nevertheless, egg hatchability was significantly reduced by 16% (*P* = 0.0004), 8% (*P* = 0.03) and 27% (*P* = 0.00001) in F1, F4 and F5 vaccinated cats, respectively when compared to controls (Table 2). Furthermore, a negative correlation was obtained between the effect of vaccination on cat flea eggs hatchability (H) or flea fertility (F) and antibody levels in vaccinated and control cats at infestation time (T3) (Fig. 7a, b). The correlation coefficients varied between different antigens and between H and F (Fig. 7a, b), and were statistically significant for H for all antigens (Fig. 7a) and for F3 and F6 for F (Fig. 7b). Although preliminary, these results provided an additional support for the effect of vaccination with cat flea recombinant antigens by showing a correlation between the effect of vaccination (antibody levels) and vaccine efficacy on flea phenotype. The vaccines showed an overall efficacy of 32–46% on the control of cat flea infestations by considering the cumulative effect on the different developmental stages (Table 2).

**Table 2:** Effect of cat vaccination with recombinant proteins on *C. felis* infestations. Results are shown for each infested cat (*n* = 3 per group) with average (Ave) ± SD. The M, H and U were compared between groups by a Fisher’s exact test (^a^*P* < 0.05). Additionally, data were analyzed statistically to compare results between cat fleas fed on vaccinated and control cats by Student’s t-test with unequal variance (^b^*P* ≤ 0.05). Vaccine efficacy (E) was determined by considering flea mortality (M, % mortality), flea fertility (F, No. of F1 adults per female), oviposition (O, No. of eggs oviposited), egg hatchability (H, % of hatched eggs), flea viability (V, No. of viable females), and flea fecundity (U, % of fecund fleas)

Group	Cat no.	Parameter												
		M	DM^a^	V	DV^b^	O	DO^c^	H	DH^d^	F	DF^e^	U	DU^f^	E^g^
F1	66326	33	-55	50	-15	1950	-11	80	+16	28	+19	100	0	32
	67437	6		74		1930		92		16		100		
	63522	21		63		1877		72		20		100		
	Ave ± SD	20 ± 14		62 ± 12		1919 ± 38		81 ± 10^a,b^		21 ± 6		100 ± 0		
F3	69095	16	-244	66	-28	2586	-8	96	+6	27	+31	100	0	35
	70317	2		70		1283		80		12		100		
	83184	10		71		1735		96		16		100		
	Ave ± SD	9 ± 7		69 ± 3		1868 ± 662		91 ± 9		18 ± 8		100 ± 0		
F4	66125	24	0	55	+4	1400	+6	84	+8	17	+23	100	0	32
	57297	13		65		1510		84		15		100		
	68830	57		36		1944		100		28		100		
	Ave ± SD	31 ± 23		52 ± 15		1618 ± 288		89 ± 9^a^		20 ± 7		100 ± 0		
F5	90839	16	-94	64	-22	2576	-14	100	+27	26	+23	100	0	44
	69835	31		58		2102		88		22		100		
	66988	0		75		1256		24		11		100		
	Ave ± SD	16 ± 16		66 ± 9		1978 ± 669		71 ± 41^a^		20 ± 8		100 ± 0		
F6	53329	14	-48	63	-15	1429	+23	80	+6	18	+42	100	0	46
	69949	23		64		791		92		9		100		
	67895	26		58		1778		100		19		100		
	Ave ± SD	21 ± 6		62 ± 3		1333 ± 501		91 ± 10		15 ± 6		100 ± 0		
Control	67996	34	–	56	–	2379	–	96	–	30	–	100	–	–
	65041	25		59		1528		100		20		100		
	71307	35		47		1286		96		21		100		
	Ave ± SD	31 ± 6		54 ± 6		1731 ± 574		97 ± 2		26 ± 6		100 ± 0		

^a^DM (% increase in flea mortality) = 100 – [(Mc, % flea mortality in control cats × 100) / Mv, % flea mortality in vaccinated cats]

^b^DV (% reduction in viable females) = 100 – [(Vv, No. viable females in vaccinated cats × 100) / Vc, No. viable females in control cats]

^c^DO (% reduction in oviposition) = 100 – [(Ov, No. eggs oviposited by flea in vaccinated cats × 100) / Oc, No. eggs oviposited by flea in control cats]

^d^DH (% reduction in egg hatchability) = 100 – [(Hv, % hatched eggs in vaccinated cats × 100) / Hc, % hatched eggs in control cats]

^e^DF (% reduction in flea fertility) = 100 - [(Fv, % hatched eggs in vaccinated cats × 100) / Fc, % hatched eggs in control cats]

^f^DU (% reduction in flea fecundity) = 100 – [(Uv, % fecund females in vaccinated cats × 100) / Uc, % fecund females in control cats]

^g^E (% vaccine efficacy) = 100 × [1- (DM × DV × DH × DF × DU)]

**Fig. 8:**
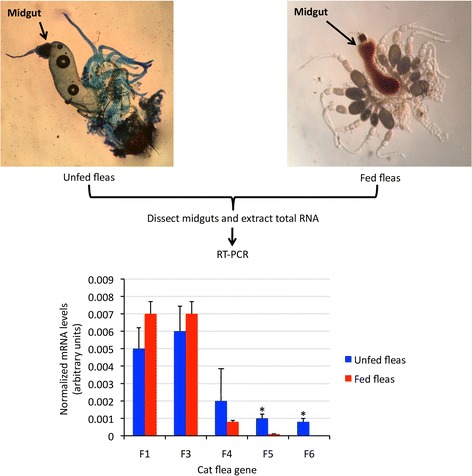
Expression of cat flea candidate protective antigens. Representative images of midguts from unfed (stained with methylene blue) and fed cat fleas from the laboratory colony maintained at the LLC ACRO Vet Lab (Kyiv region, Ukraine) and used in this study. Total RNA was extracted from midguts dissected from unfed and fed cat flea and used for RT-PCR suing gene-specific oligonucleotide primers. The mRNA levels were normalized against cat flea *18S* rRNA, presented as average + SD, and normalized Ct values were compared between unfed and fed fleas by Student's t-test with unequal variance (*P < 0.05; *n* = 3 biological replicates)

To gain additional information on the function of these proteins during cat flea life cycle, the expression profile was characterized by RT-PCR in midguts collected from unfed and fed fleas (Fig. 8). The results showed that expression of F5 and F6 coding genes decreased in fed fleas when compare to unfed fleas, therefore suggesting that these proteins do not play a major role during cat flea feeding.

## Discussion

The reverse vaccinology approach used in this study was designed to achieve a rational selection of candidate protective antigens [31, 34, 41]. Based on transcriptomics and proteomics data it was possible to select more specific candidate protective antigens based on highly represented and functionally relevant proteins present in the putative cat flea exoproteome.

The transcriptomics and proteomics data obtained here was focused on the identification of candidate protective antigens. Nevertheless, it provided new databases with cat flea transcripts and proteins, with particular emphasis on the insect exoproteome. In cat fleas, the most abundant MF at both transcriptomics and proteomics levels was binding, which probably reflected the presence of interacting proteins in the predicted exoproteome. An interesting finding was the identification of vertebrate host proteins in unfed cat fleas. Vertebrate host proteins have been identified in the midguts, salivary glands and saliva of fed ticks [58–65]. Additionally, vertebrate host proteins and particularly alpha and beta-globin chains of hemoglobin have been identified in unfed tick nymphs and adults, suggesting that host proteins persisting in the tick after molting may serve as a reserve for nutrients until next infestation and feeding cycle are completed [66, 67]. Taken together, these results suggested that the persistence of host proteins in unfed blood-feeding ectoparasites might be a general mechanism to provide nutrients in off-host parasites.

Six candidate protective antigens were selected, with potentially relevant functions in cat flea biology. The arginine kinase (F1) is an enzyme crucial for the energy metabolism of insects and other invertebrates that has been described before in *C. felis* [68]. The sodium/potassium-transporting ATPase subunit alpha (F2) is a transmembrane ATPase that helps maintain resting potential, avail transport and regulate cellular volume and energy, and has been also described before in *C. felis* [69]. The serpin 4 (F3) is a member of the serpins family of serine protease inhibitors that are involved in the regulation of the proteolytic cascades involved in blood clotting, inflammatory response and tissue remodeling [70]. The genes encoding serpin proteins have been isolated and characterized before in *C. felis* [70]. The juvenile hormone epoxide hydrolase 1 (F4) catalyzes hydrolysis of the juvenile hormone, involved in the regulation of insect development and reproduction, and is produced in all *C. felis* stages from first instar larvae to adults [71]. The xylosyltransferase (F5) catalyzes the first step in the biosynthesis of glycosaminoglycan by transferring D-xylose from UDP-D-xylose to specific serine residues of the core protein and is essential for animal development [72]. The zinc transporter ZIP13 homolog (F6) is a metal ion transporter that plays a major role in maintaining the correct concentrations of the various metal ions in the different cellular compartments and is essential for many metabolic processes including development and immune response [73]. The effect of vaccination with these antigens on egg hatchability and flea fertility correlated with their role in metabolic and developmental processes in cat fleas and other insects [68, 70–73]. Furthermore, the result that only the F4 and F6 antigens had an effect on reducing oviposition correlated with the role of these proteins in insect development and reproduction [71].

These results supported the selection of cat flea candidate protective antigens using the reverse vaccinology approach. Vaccines against ectoparasites are not designed to prevent infestations but to reduce arthropod populations by affecting their feeding, reproduction and development after feeding on immunized animals and ingesting antigen-specific antibodies that interact with and affect target protein function [19]. Therefore, vaccine E was determined by considering the effect on different cat flea developmental stages (Fig. 1). Based on previous results with vaccines containing tick [19] and cat flea [24] antigens, and the finding that ingested cat intact IgG passage through midgut into the hemocoel of cat fleas [74], the results suggested an effect of the vaccines described here based on the interactions between ingested anti-cat flea antigen antibodies and target proteins that affect protein function resulting in reduced cat flea fitness. Furthermore, the correlation between vaccination and cat flea phenotype, which has been proposed as a correlate of protection in tick vaccine trials [19, 27, 55, 56], showed a negative correlation between cat flea eggs hatchability (H) or flea fertility (F) and antibody levels in vaccinated and control cats, providing additional support for the effect of vaccination on flea reproductive capacity. As previously reported in ticks [19], vaccines affecting reproductive capacity could reduce cat flea populations, particularly under conditions of direct insect transmission between cats [75]. However, vaccination with these antigens did not have an effect on flea infestations, which is necessary for an effective control of these insects in cats and dogs.

Previous results of vaccine trials against cat flea infestations showed that immunization of cats with membrane antigens extracted from the gut of unfed fleas did not affect flea infestations and fecundity [23]. However, Heath et al. [24] reported reduction in cat flea infestations and egg production in dogs vaccinated with soluble antigens extracted from midguts of fed fleas, suggesting that flea protective antigens may be present in the midgut of fed but not unfed fleas. Using a vaccine combination of DNA and coded protein of flea salivary antigen FSA1, Jin et al. [25] showed that this co-immunization approach was effective in reducing flea allergy dermatitis in cats.

## Conclusions

In summary, although still on the initial phases of research, these results support the development of vaccines for the control of cat flea infestations. The advance provided by new omic technologies will improve the identification of candidate protective antigens by using different pipelines such as the reverse vaccinology used here. Single or combined protective antigens affecting insect infestation, reproduction and development after feeding on immunized animals would result in the control of cat flea infestations, therefore reducing the disease risks associated with them.

## Additional files

Additional file 1: Dataset 1. Sequences of predicted transmembrane and secreted proteins encoded in the transcriptome and used for PIT. (PDF 303 kb)

Additional file 2: Dataset 2. Coding sequences for selected candidate protective antigens. **Dataset 3.** De novo sequence assembly process for sequences F5 and F6. (PDF 148 kb)

Additional file 3: Dataset 4. Transcriptomics data analysis including predicted transcripts encoding proteins in the exoproteome. (XLS 17203 kb)

Additional file 4: Dataset 5. Proteomics data for cat flea and host proteins identified in the plasma membrane. (XLSX 26 kb)
