# Supplementary material for: A reverse vaccinology approach to the identification and characterization of Ctenocephalides felis candidate protective antigens for the control of cat flea infestations
Source: Parasit Vectors. 2018 Jan 18;11:43. doi: 10.1186/s13071-018-2618-x (PMC5774092; doi:10.1186/s13071-018-2618-x)
Supplement: Supplementary file 1 — Sequences of predicted transmembrane and secreted proteins encoded in the transcriptome and used for PIT. (PDF 303 kb) [file 13071_2018_2618_MOESM1_ESM.pdf]

Additional file 1. Dataset 1. Sequences of predicted transmembrane and secreted proteins encoded in the transcriptome and used for PIT.

>Locus_12657_Transcript_1/1_Confidence_1.000_Length_2513|UNIGENE REPRESENTATIVE TRANSCRIPT|sp|A1Z8N1|TRE11_DROME Facilitated trehalose transporter Tret1-1 OS=Drosophila melanogaster GN=Tret1-1 PE=1 SV=1|e=5.47E-157|from=339|to=1955|frame=-1|Fragment_1
LGSDDDEEIAVARLIFQGQREVSIDSKRKLRI
>Locus_12657_Transcript_1/1_Confidence_1.000_Length_2513|UNIGENE REPRESENTATIVE TRANSCRIPT|sp|A1Z8N1|TRE11_DROME Facilitated trehalose transporter Tret1-1 OS=Drosophila melanogaster GN=Tret1-1 PE=1 SV=1|e=5.47E-157|from=339|to=1955|frame=-1|Fragment_2
EM
>Locus_12657_Transcript_1/1_Confidence_1.000_Length_2513|UNIGENE REPRESENTATIVE TRANSCRIPT|sp|A1Z8N1|TRE11_DROME Facilitated trehalose transporter Tret1-1 OS=Drosophila melanogaster GN=Tret1-1 PE=1 SV=1|e=5.47E-157|from=339|to=1955|frame=-1|Fragment_3
ELQRMKEENLRSSVPYLASL
>Locus_12657_Transcript_1/1_Confidence_1.000_Length_2513|UNIGENE REPRESENTATIVE TRANSCRIPT|sp|A1Z8N1|TRE11_DROME Facilitated trehalose transporter Tret1-1 OS=Drosophila melanogaster GN=Tret1-1 PE=1 SV=1|e=5.47E-157|from=339|to=1955|frame=-1|Fragment_4
VYQPTTTPIRIATQ
>Locus_12657_Transcript_1/1_Confidence_1.000_Length_2513|UNIGENE REPRESENTATIVE TRANSCRIPT|sp|A1Z8N1|TRE11_DROME Facilitated trehalose transporter Tret1-1 OS=Drosophila melanogaster GN=Tret1-1 PE=1 SV=1|e=5.47E-157|from=339|to=1955|frame=-1|Fragment_5
GYTSPALVTMTSENNT
>Locus_12657_Transcript_1/1_Confidence_1.000_Length_2513|UNIGENE REPRESENTATIVE TRANSCRIPT|sp|A1Z8N1|TRE11_DROME Facilitated trehalose transporter Tret1-1 OS=Drosophila melanogaster GN=Tret1-1 PE=1 SV=1|e=5.47E-157|from=339|to=1955|frame=-1|Fragment_6
FGVTSQQVSWV
>Locus_12657_Transcript_1/1_Confidence_1.000_Length_2513|UNIGENE REPRESENTATIVE TRANSCRIPT|sp|A1Z8N1|TRE11_DROME Facilitated trehalose transporter Tret1-1 OS=Drosophila melanogaster GN=Tret1-1 PE=1 SV=1|e=5.47E-157|from=339|to=1955|frame=-1|Fragment_7
IEKFGRRMTILATAPPFALAWLLIACATDIWYVLSGRALSGLCVGIATLSLPVYLGETLQ
SEVRGTLGLLPTALGNIGILVVFSTGMYLDWRALAYIGAA
>Locus_12657_Transcript_1/1_Confidence_1.000_Length_2513|UNIGENE REPRESENTATIVE TRANSCRIPT|sp|A1Z8N1|TRE11_DROME Facilitated trehalose transporter Tret1-1 OS=Drosophila melanogaster GN=Tret1-1 PE=1 SV=1|e=5.47E-157|from=339|to=1955|frame=-1|Fragment_8
PHWQVAHGNEQGARKSLQWLRGGNELEVAAELKNIV
>Locus_12657_Transcript_1/1_Confidence_1.000_Length_2513|UNIGENE REPRESENTATIVE TRANSCRIPT|sp|A1Z8N1|TRE11_DROME Facilitated trehalose transporter Tret1-1 OS=Drosophila melanogaster GN=Tret1-1 PE=1 SV=1|e=5.47E-157|from=339|to=1955|frame=-1|Fragment_9
STIESENISKNITILDLFKRNNLKPLLISLGLMLFQQFSGINAVIFYTVMIFKDAGSSVD
ENLCTIIVGFVNFGATFLATVLIDRLGRKVLLYISGIAMTITLITLGGFFYVKHIGTDVS
TIGWLPLACFVIYVVGFSLGFGPVPWLMMGEILPSKVRGTAASVATSFNWLCTFLVTKTF
TDMLVVMGTYGAFWLFGTVCVIALFFVFFFVPETQGRSLEDIELSMRGHSRKVGSLANMK
PL

```
>Locus_6993_Transcript_4/7_Confidence_0.316_Length_767|UNIGENE
REPRESENTATIVE TRANSCRIPT|sp|A8J4S9|TREA_APIME Trehalase OS=Apis
mellifera PE=1 SV=1|e=6.78314E-41|from=319|to=765|frame=1|Fragment_1
CKSEIYCRGELLDTVQMAKIFNDSKTFVDMKMKYDENKTMEKFYEMMNEIEPNQPNQTTI
KQFVNNYFEPVGKEF
>Locus_6993_Transcript_4/7_Confidence_0.316_Length_767|UNIGENE
REPRESENTATIVE TRANSCRIPT|sp|A8J4S9|TREA_APIME Trehalase OS=Apis
mellifera PE=1 SV=1|e=6.78314E-41|from=319|to=765|frame=1|Fragment_2
EFLNKIYDDSLRQFAEELHILWKQLGRKMDPKIKDEEEKYSIIYVENPVVVPGGRFREFY
YW
>Locus_6993_Transcript_2/7_Confidence_0.263_Length_718|.|sp|A8J4S9|
TREA_APIME Trehalase OS=Apis mellifera PE=1 SV=1|e=2.24081E-19|
from=438|to=710|frame=3|Fragment_1
ANLQTVRVRYSEIYCRGELLDTVQMAKIFNDSKTFVDMKMKYDENKTMEKFYEMMNEIEP
NQPNQTTIKQFVNNYFEPVGKEFIPWIPDDW
>Locus_6993_Transcript_6/7_Confidence_0.263_Length_575|.|sp|A8J4S9|
TREA_APIME Trehalase OS=Apis mellifera PE=1 SV=1|e=1.35417E-20|
from=319|to=567|frame=1|Fragment_1
CKSEIYCRGELLDTVQMAKIFNDSKTFVDMKMKYDENKTMEKFYEMMNEIEPNQPNQTTI
KQFVNNYFEPVGKEFIPWIPDDW

>Locus_16221_Transcript_1/1_Confidence_1.000_Length_698|UNIGENE
REPRESENTATIVE TRANSCRIPT|sp|D2A0H5|SNMP1_TRICA Sensory neuron
membrane protein 1 OS=Tribolium castaneum GN=SNMP01 PE=3 SV=1|
e=2.07455E-46|from=5|to=622|frame=-2|Fragment_1
YSFFGYRNNTALDSFTISRGLRNVGDLGKVLKFRGSQTLDMWMR
>Locus_16221_Transcript_1/1_Confidence_1.000_Length_698|UNIGENE
REPRESENTATIVE TRANSCRIPT|sp|D2A0H5|SNMP1_TRICA Sensory neuron
membrane protein 1 OS=Tribolium castaneum GN=SNMP01 PE=3 SV=1|
e=2.07455E-46|from=5|to=622|frame=-2|Fragment_2
PVNETKSCTKLRGTDSTIFPPFLEPSEGLWSFSSDICRSIAAIYEGEGYYKGIPIRRYNA
TFGDPKTDPDTKCYCPKDPRPCPLAGTFDLSPCTGAPIIASLPHFFAADPKLVKDIKGLN
PDKDKHEIFVDFESITGKPLSARKRMQFNMPL

>Locus_8710_Transcript_1/2_Confidence_0.750_Length_8144|UNIGENE
REPRESENTATIVE TRANSCRIPT|sp|O97394|SDK_DROME Protein sidekick
OS=Drosophila melanogaster GN=sdk PE=1 SV=2|e=0|from=95|to=6547|
frame=2|Fragment_1
ESLQAPRFTTQPSASSNIISEGRTKILQCQALGYPQPRYRWLRDGQPLGDFSSEPFYKIL
NTRREDAGSYQCVASNDVGSIMSERIDFTVAYMGVFEDQIERTVFVESGQAAILDLPLIE
SHPTPSVTWQSDMGSLPYDHKFAVTSNHQLVILSASEADQRSYRAHAINTQSGKEENSAF
IRVNVTGDYAYEVAPEIIIKPTDLKIVKGQQLAELQCIANARPLHELETLWLKDGILIES
SGISYNFNDPWNRTLGLLSANLTYSGQYTCNVRLRSGGFPTVTSKADVVVLEKPRFINNM
RAETLGDYGSQVVLPCDVIGEPMPIIKWYKNVQE
>Locus_8710_Transcript_1/2_Confidence_0.750_Length_8144|UNIGENE
REPRESENTATIVE TRANSCRIPT|sp|O97394|SDK_DROME Protein sidekick
OS=Drosophila melanogaster GN=sdk PE=1 SV=2|e=0|from=95|to=6547|
frame=2|Fragment_2
IQDKGYEVQEDGSLLIKRLATHDSAMFQCVARNEAGEQSGYTWLKVKTSKPILEVPPENV
TVLDGKDATISCRAAGAPAPNVTWYYNDDGPLEINSHLQLLESGDLLISDVRETDAGKYK
CVRANEAGTVSGEAYLTVMVRTQIIQPPVDTNVLLGLTATLQCKVSNDPSVPYHIDWYQN
HELIV
>Locus_8710_Transcript_1/2_Confidence_0.750_Length_8144|UNIGENE
REPRESENTATIVE TRANSCRIPT|sp|O97394|SDK_DROME Protein sidekick
```

OS=Drosophila melanogaster GN=sdk PE=1 SV=2|e=0|from=95|to=6547|
frame=2|Fragment_3
FGNSPRINILIDGTLEIQAVRATDVGEYSCYVTSPGGNETRTARLSVIELPFAPTNIKAE
KLDTLTQRAINVSWTPGFDGNSPTKNFIVEKREVPEL
>Locus_8710_Transcript_1/2_Confidence_0.750_Length_8144|UNIGENE
REPRESENTATIVE TRANSCRIPT|sp|O97394|SDK_DROME Protein sidekick
OS=Drosophila melanogaster GN=sdk PE=1 SV=2|e=0|from=95|to=6547|
frame=2|Fragment_4
GPLPDPLLNWVTELSNVSANQRWILLTNLKAAAAYQFRVSTVNSVGEGSPSEPSNVVMLP
QEAPSGPPVGFVGSARSSSEIITQWQPPLEEHRNGQILGYVIRYRLFGYNNSPWTSRNIT
NEAQRNFLIQELITWKDYIVQIAAYNNKGVGVFTEGAKIKTKEGVPESPPTNVKAKALNS
TSVRIWWKPPDPQKINGINQGYKLQAWISEIEENSNVIQVEARMLTVPPSLFDPLAEQST
IMNGLEKFTEYNITVLCFTDPGDGVRSKPTPVRTSEDVPDEVSLLQFNDVSDRAVNVAWA
PPRQVNGILTGYRVRYQIKDTPSTLKIVNLSSNVTSLMVTQLQATTHYWFEVTAHTAKGN
GVPRTATIQSGVEPVLPDPPYQLALSNIEAFSVVLQFTPGFDGNSSITLWKVEAQTARNS
TWFTIFEVSDPDATTLTVTGLVPFTVYRLRLIANNVVGHSLPSEPSKEFQTIQARPSHPP
KNVTVRAMSATELRVRWIPLQQIEWFGNPRGYNITYASIEPNP
>Locus_8710_Transcript_1/2_Confidence_0.750_Length_8144|UNIGENE
REPRESENTATIVE TRANSCRIPT|sp|O97394|SDK_DROME Protein sidekick
OS=Drosophila melanogaster GN=sdk PE=1 SV=2|e=0|from=95|to=6547|
frame=2|Fragment_5
VPEVRSVLIEDHTANSHVLDGLEEWTLYSISMHACNDVGSSFSSPAAIERTREAVPSFGP
LNVEANATSSTTIVVKWKEVPLEHQNGQIEGYKVYYGAGVR
>Locus_8710_Transcript_1/2_Confidence_0.750_Length_8144|UNIGENE
REPRESENTATIVE TRANSCRIPT|sp|O97394|SDK_DROME Protein sidekick
OS=Drosophila melanogaster GN=sdk PE=1 SV=2|e=0|from=95|to=6547|
frame=2|Fragment_6
DPVRYKQILDNSTFTTTLTELKKYVQYHVQVLAFTRLGDGALSSPPIRIQTFEDAPGAPS
NVSFPDVSYTTARIIWDVPQDPNGEILAYRVTYHMNLNPELNYSREFPPSDRTFRATELM
AEQYYMFSVTAQTRLGWGKIASALVYTTNNREIPQPPSLPQISRSQVQAQQITFSWTPGR
DGYAPLRYYTVQLRENEGPWHSLPERVNPAVTSYTATNLRPYSTYQFRIRATNDIGPSAY
STESIEVRTLPAAPSKEISNIRAVPITPTKVRVEWDVLPYQYWSGDNDTGGYRVLFQPVT
DFPAALQATPKEDVSGINSDMLILSDLTPDRNYEVIVVAFNSQGLGPPSPPITVYVGEAV
PTGEPQSIQVQAVSSTEVLLKWKPPKQSMQNGELLGYKIFYLVTDSPQEPENDKPIEEEI
EVVPATYSAHSLVFLDKYTEYKIQILAFNPAGDGPRSLPVTVRTLQGLPGPPMNLQFTDI
TMNSLNVSWDPPKKRNGDILGYIVTYETTEQNERFSKQVKQKVSETSLIVQSLEEEVTYT
FTVRAQTKIDFGPAILGNITTGPQEGSPLSPKELTITKTISSVQMHWSNSASGRGPILGY
YIEAKRKNEGRWETVWRTSNGPLQEFVISYQSLLPSTSYKFRIITYNQYGISYPLYSEDS
ILTPSKLYLEYGYLQHKPFYRQNWFMVALAATSIVIIIMVIAVLCVKSKSYKYKQEAQKT
LEESMAMDIDDRQELALELYRSRHNASTMSGSGAGTLSRRLANGTLGRKSNNNNIHGGGV
MNPALGKSPPRPSPASVAYHSDEESLKGYDENPDDSSVT
>Locus_8710_Transcript_1/2_Confidence_0.750_Length_8144|UNIGENE
REPRESENTATIVE TRANSCRIPT|sp|O97394|SDK_DROME Protein sidekick
OS=Drosophila melanogaster GN=sdk PE=1 SV=2|e=0|from=95|to=6547|
frame=2|Fragment_7
VRSDPHSFVNHYANVNDSLRQSWKRQKPVRNYSSYTDSEPEGSAVVSLNGGQIIMNNMAR
SRAPLPGFSSFV

>Locus_6595_Transcript_1/3_Confidence_0.714_Length_1093|UNIGENE
REPRESENTATIVE TRANSCRIPT|sp|P0CY46|EGFR_APIME Epidermal growth
factor receptor OS=Apis mellifera GN=Egfr PE=2 SV=1|e=1.12262E-35|
from=1|to=702|frame=1|Fragment_1
TTQDEKDLIKNLATTLDGPEPVVEADEYLQPKSR
>Locus_6595_Transcript_1/3_Confidence_0.714_Length_1093|UNIGENE

REPRESENTATIVE TRANSCRIPT|sp|P0CY46|EGFR_APIME Epidermal growth factor receptor OS=Apis mellifera GN=Egfr PE=2 SV=1|e=1.12262E-35|from=1|to=702|frame=1|Fragment_2
TVTVLPP
>Locus_6595_Transcript_1/3_Confidence_0.714_Length_1093|UNIGENE REPRESENTATIVE TRANSCRIPT|sp|P0CY46|EGFR_APIME Epidermal growth factor receptor OS=Apis mellifera GN=Egfr PE=2 SV=1|e=1.12262E-35|from=1|to=702|frame=1|Fragment_3
SAGPQVAHN
>Locus_6595_Transcript_1/3_Confidence_0.714_Length_1093|UNIGENE REPRESENTATIVE TRANSCRIPT|sp|P0CY46|EGFR_APIME Epidermal growth factor receptor OS=Apis mellifera GN=Egfr PE=2 SV=1|e=1.12262E-35|from=1|to=702|frame=1|Fragment_4
NHENG
>Locus_6595_Transcript_1/3_Confidence_0.714_Length_1093|UNIGENE REPRESENTATIVE TRANSCRIPT|sp|P0CY46|EGFR_APIME Epidermal growth factor receptor OS=Apis mellifera GN=Egfr PE=2 SV=1|e=1.12262E-35|from=1|to=702|frame=1|Fragment_5
PGS
>Locus_6595_Transcript_1/3_Confidence_0.714_Length_1093|UNIGENE REPRESENTATIVE TRANSCRIPT|sp|P0CY46|EGFR_APIME Epidermal growth factor receptor OS=Apis mellifera GN=Egfr PE=2 SV=1|e=1.12262E-35|from=1|to=702|frame=1|Fragment_6
PESFMGSL
>Locus_6595_Transcript_1/3_Confidence_0.714_Length_1093|UNIGENE REPRESENTATIVE TRANSCRIPT|sp|P0CY46|EGFR_APIME Epidermal growth factor receptor OS=Apis mellifera GN=Egfr PE=2 SV=1|e=1.12262E-35|from=1|to=702|frame=1|Fragment_7
GQKRYCSDPLKG
>Locus_6595_Transcript_1/3_Confidence_0.714_Length_1093|UNIGENE REPRESENTATIVE TRANSCRIPT|sp|P0CY46|EGFR_APIME Epidermal growth factor receptor OS=Apis mellifera GN=Egfr PE=2 SV=1|e=1.12262E-35|from=1|to=702|frame=1|Fragment_8
ETTD
>Locus_6595_Transcript_1/3_Confidence_0.714_Length_1093|UNIGENE REPRESENTATIVE TRANSCRIPT|sp|P0CY46|EGFR_APIME Epidermal growth factor receptor OS=Apis mellifera GN=Egfr PE=2 SV=1|e=1.12262E-35|from=1|to=702|frame=1|Fragment_9
EVDTKRGEAHIGRLKLDLPLDEDDYLMPS
>Locus_6595_Transcript_1/3_Confidence_0.714_Length_1093|UNIGENE REPRESENTATIVE TRANSCRIPT|sp|P0CY46|EGFR_APIME Epidermal growth factor receptor OS=Apis mellifera GN=Egfr PE=2 SV=1|e=1.12262E-35|from=1|to=702|frame=1|Fragment_10
TQTSAYMDLIGDSKNPEQ
>Locus_6595_Transcript_1/3_Confidence_0.714_Length_1093|UNIGENE REPRESENTATIVE TRANSCRIPT|sp|P0CY46|EGFR_APIME Epidermal growth factor receptor OS=Apis mellifera GN=Egfr PE=2 SV=1|e=1.12262E-35|from=1|to=702|frame=1|Fragment_11
RAYPDFL
>Locus_6595_Transcript_1/3_Confidence_0.714_Length_1093|UNIGENE REPRESENTATIVE TRANSCRIPT|sp|P0CY46|EGFR_APIME Epidermal growth factor receptor OS=Apis mellifera GN=Egfr PE=2 SV=1|e=1.12262E-35|from=1|to=702|frame=1|Fragment_12
PAIDNPEYLLSSEPYSGPPPTQTLGIPLTG

>Locus_6595_Transcript_1/3_Confidence_0.714_Length_1093|UNIGENE REPRESENTATIVE TRANSCRIPT|sp|P0CY46|EGFR_APIME Epidermal growth factor receptor OS=Apis mellifera GN=Egfr PE=2 SV=1|e=1.12262E-35|from=1|to=702|frame=1|Fragment_13
IDKVDLKQSVDSLQRQQRTNKEDEISDHEYYND
>Locus_6595_Transcript_1/3_Confidence_0.714_Length_1093|UNIGENE REPRESENTATIVE TRANSCRIPT|sp|P0CY46|EGFR_APIME Epidermal growth factor receptor OS=Apis mellifera GN=Egfr PE=2 SV=1|e=1.12262E-35|from=1|to=702|frame=1|Fragment_14
VQRELQ
>Locus_6595_Transcript_1/3_Confidence_0.714_Length_1093|UNIGENE REPRESENTATIVE TRANSCRIPT|sp|P0CY46|EGFR_APIME Epidermal growth factor receptor OS=Apis mellifera GN=Egfr PE=2 SV=1|e=1.12262E-35|from=1|to=702|frame=1|Fragment_15
PLRRSETTV
>Locus_6595_Transcript_2/3_Confidence_0.714_Length_1092|.|sp|P0CY46|EGFR_APIME Epidermal growth factor receptor OS=Apis mellifera GN=Egfr PE=2 SV=1|e=1.12262E-35|from=1|to=702|frame=1|Fragment_1
TTQDEKDLIKNLATTLDGPEPVVEADEYLQPKSR
>Locus_6595_Transcript_2/3_Confidence_0.714_Length_1092|.|sp|P0CY46|EGFR_APIME Epidermal growth factor receptor OS=Apis mellifera GN=Egfr PE=2 SV=1|e=1.12262E-35|from=1|to=702|frame=1|Fragment_2
TVTVLPP
>Locus_6595_Transcript_2/3_Confidence_0.714_Length_1092|.|sp|P0CY46|EGFR_APIME Epidermal growth factor receptor OS=Apis mellifera GN=Egfr PE=2 SV=1|e=1.12262E-35|from=1|to=702|frame=1|Fragment_3
SAGPQVAHN
>Locus_6595_Transcript_2/3_Confidence_0.714_Length_1092|.|sp|P0CY46|EGFR_APIME Epidermal growth factor receptor OS=Apis mellifera GN=Egfr PE=2 SV=1|e=1.12262E-35|from=1|to=702|frame=1|Fragment_4
NHENG
>Locus_6595_Transcript_2/3_Confidence_0.714_Length_1092|.|sp|P0CY46|EGFR_APIME Epidermal growth factor receptor OS=Apis mellifera GN=Egfr PE=2 SV=1|e=1.12262E-35|from=1|to=702|frame=1|Fragment_5
PGS
>Locus_6595_Transcript_2/3_Confidence_0.714_Length_1092|.|sp|P0CY46|EGFR_APIME Epidermal growth factor receptor OS=Apis mellifera GN=Egfr PE=2 SV=1|e=1.12262E-35|from=1|to=702|frame=1|Fragment_6
PESFMGSL
>Locus_6595_Transcript_2/3_Confidence_0.714_Length_1092|.|sp|P0CY46|EGFR_APIME Epidermal growth factor receptor OS=Apis mellifera GN=Egfr PE=2 SV=1|e=1.12262E-35|from=1|to=702|frame=1|Fragment_7
GQKRYCSDPLKG
>Locus_6595_Transcript_2/3_Confidence_0.714_Length_1092|.|sp|P0CY46|EGFR_APIME Epidermal growth factor receptor OS=Apis mellifera GN=Egfr PE=2 SV=1|e=1.12262E-35|from=1|to=702|frame=1|Fragment_8
ETTD
>Locus_6595_Transcript_2/3_Confidence_0.714_Length_1092|.|sp|P0CY46|EGFR_APIME Epidermal growth factor receptor OS=Apis mellifera GN=Egfr PE=2 SV=1|e=1.12262E-35|from=1|to=702|frame=1|Fragment_9
EVDTKRGEAHIGRLKLDLPLDEDDYLMPS
>Locus_6595_Transcript_2/3_Confidence_0.714_Length_1092|.|sp|P0CY46|EGFR_APIME Epidermal growth factor receptor OS=Apis mellifera GN=Egfr PE=2 SV=1|e=1.12262E-35|from=1|to=702|frame=1|Fragment_10

TQTSAYMDLIGDSKNPEQ
>Locus_6595_Transcript_2/3_Confidence_0.714_Length_1092|.|sp|P0CY46|
EGFR_APIME Epidermal growth factor receptor OS=Apis mellifera
GN=Egfr PE=2 SV=1|e=1.12262E-35|from=1|to=702|frame=1|Fragment_11
RAYPDFL
>Locus_6595_Transcript_2/3_Confidence_0.714_Length_1092|.|sp|P0CY46|
EGFR_APIME Epidermal growth factor receptor OS=Apis mellifera
GN=Egfr PE=2 SV=1|e=1.12262E-35|from=1|to=702|frame=1|Fragment_12
PAIDNPEYLLSSEPYSGPPPTQTLGIPLTG
>Locus_6595_Transcript_2/3_Confidence_0.714_Length_1092|.|sp|P0CY46|
EGFR_APIME Epidermal growth factor receptor OS=Apis mellifera
GN=Egfr PE=2 SV=1|e=1.12262E-35|from=1|to=702|frame=1|Fragment_13
IDKVDLKQSVDSLQRQQRTNKEDEISDHEYYND
>Locus_6595_Transcript_2/3_Confidence_0.714_Length_1092|.|sp|P0CY46|
EGFR_APIME Epidermal growth factor receptor OS=Apis mellifera
GN=Egfr PE=2 SV=1|e=1.12262E-35|from=1|to=702|frame=1|Fragment_14
VQRELQ
>Locus_6595_Transcript_2/3_Confidence_0.714_Length_1092|.|sp|P0CY46|
EGFR_APIME Epidermal growth factor receptor OS=Apis mellifera
GN=Egfr PE=2 SV=1|e=1.12262E-35|from=1|to=702|frame=1|Fragment_15
PLRRSETTV
>Locus_6595_Transcript_3/3_Confidence_0.714_Length_1092|.|sp|P0CY46|
EGFR_APIME Epidermal growth factor receptor OS=Apis mellifera
GN=Egfr PE=2 SV=1|e=1.12262E-35|from=1|to=702|frame=1|Fragment_1
TTQDEKDLIKNLATTLDGPEPVVEADEYLQPKSR
>Locus_6595_Transcript_3/3_Confidence_0.714_Length_1092|.|sp|P0CY46|
EGFR_APIME Epidermal growth factor receptor OS=Apis mellifera
GN=Egfr PE=2 SV=1|e=1.12262E-35|from=1|to=702|frame=1|Fragment_2
TVTVLPP
>Locus_6595_Transcript_3/3_Confidence_0.714_Length_1092|.|sp|P0CY46|
EGFR_APIME Epidermal growth factor receptor OS=Apis mellifera
GN=Egfr PE=2 SV=1|e=1.12262E-35|from=1|to=702|frame=1|Fragment_3
SAGPQVAHN
>Locus_6595_Transcript_3/3_Confidence_0.714_Length_1092|.|sp|P0CY46|
EGFR_APIME Epidermal growth factor receptor OS=Apis mellifera
GN=Egfr PE=2 SV=1|e=1.12262E-35|from=1|to=702|frame=1|Fragment_4
NHENG
>Locus_6595_Transcript_3/3_Confidence_0.714_Length_1092|.|sp|P0CY46|
EGFR_APIME Epidermal growth factor receptor OS=Apis mellifera
GN=Egfr PE=2 SV=1|e=1.12262E-35|from=1|to=702|frame=1|Fragment_5
PGS
>Locus_6595_Transcript_3/3_Confidence_0.714_Length_1092|.|sp|P0CY46|
EGFR_APIME Epidermal growth factor receptor OS=Apis mellifera
GN=Egfr PE=2 SV=1|e=1.12262E-35|from=1|to=702|frame=1|Fragment_6
PESFMGSL
>Locus_6595_Transcript_3/3_Confidence_0.714_Length_1092|.|sp|P0CY46|
EGFR_APIME Epidermal growth factor receptor OS=Apis mellifera
GN=Egfr PE=2 SV=1|e=1.12262E-35|from=1|to=702|frame=1|Fragment_7
GQKRYCSDPLKG
>Locus_6595_Transcript_3/3_Confidence_0.714_Length_1092|.|sp|P0CY46|
EGFR_APIME Epidermal growth factor receptor OS=Apis mellifera
GN=Egfr PE=2 SV=1|e=1.12262E-35|from=1|to=702|frame=1|Fragment_8
ETTD
>Locus_6595_Transcript_3/3_Confidence_0.714_Length_1092|.|sp|P0CY46|

EGFR_APIME Epidermal growth factor receptor OS=Apis mellifera
GN=Egfr PE=2 SV=1|e=1.12262E-35|from=1|to=702|frame=1|Fragment_9
EVDTKRGEAHIGRLKLDLPLDEDDYLMPS
>Locus_6595_Transcript_3/3_Confidence_0.714_Length_1092|.|sp|P0CY46|
EGFR_APIME Epidermal growth factor receptor OS=Apis mellifera
GN=Egfr PE=2 SV=1|e=1.12262E-35|from=1|to=702|frame=1|Fragment_10
TQTSAYMDLIGDSKNPEQ
>Locus_6595_Transcript_3/3_Confidence_0.714_Length_1092|.|sp|P0CY46|
EGFR_APIME Epidermal growth factor receptor OS=Apis mellifera
GN=Egfr PE=2 SV=1|e=1.12262E-35|from=1|to=702|frame=1|Fragment_11
RAYPDFL
>Locus_6595_Transcript_3/3_Confidence_0.714_Length_1092|.|sp|P0CY46|
EGFR_APIME Epidermal growth factor receptor OS=Apis mellifera
GN=Egfr PE=2 SV=1|e=1.12262E-35|from=1|to=702|frame=1|Fragment_12
PAIDNPEYLLSSEPYSGPPPTQTLGIPLTG
>Locus_6595_Transcript_3/3_Confidence_0.714_Length_1092|.|sp|P0CY46|
EGFR_APIME Epidermal growth factor receptor OS=Apis mellifera
GN=Egfr PE=2 SV=1|e=1.12262E-35|from=1|to=702|frame=1|Fragment_13
IDKVDLKQSVDSLQRQQRTNKEDEISDHEYYND
>Locus_6595_Transcript_3/3_Confidence_0.714_Length_1092|.|sp|P0CY46|
EGFR_APIME Epidermal growth factor receptor OS=Apis mellifera
GN=Egfr PE=2 SV=1|e=1.12262E-35|from=1|to=702|frame=1|Fragment_14
VQRELQ
>Locus_6595_Transcript_3/3_Confidence_0.714_Length_1092|.|sp|P0CY46|
EGFR_APIME Epidermal growth factor receptor OS=Apis mellifera
GN=Egfr PE=2 SV=1|e=1.12262E-35|from=1|to=702|frame=1|Fragment_15
PLRRSETTV
>Locus_12106_Transcript_1/1_Confidence_1.000_Length_799|.|sp|P0CY46|
EGFR_APIME Epidermal growth factor receptor OS=Apis mellifera
GN=Egfr PE=2 SV=1|e=4.142E-129|from=3|to=797|frame=3|Fragment_1
EEAYIMATVEHPNLLQLLAVCMTSQMMLITQLMPLGCLLDYVRTHREKIGSKALLNWSTQ
IARGMAYLEERRLVHRDLAARNVLVQNPNCVKITDFGLAKLLDVNEDEYKAAGGKMPIKW
LALECIQHRIFTHKSDVWAFGVTIWELLTYGARPYENISAKDVPELIENGLKLPQPAICT
LDIYCILVSCWMLDAEARPSFKQLTETFAEKAADPGRYLYIPGDKFMRLPTFTTQDEKDL
IKNLATTLDGPEPVVEADEYLQPKS
>Locus_18909_Transcript_1/1_Confidence_1.000_Length_635|.|sp|P0CY46|
EGFR_APIME Epidermal growth factor receptor OS=Apis mellifera
GN=Egfr PE=2 SV=1|e=6.3169E-105|from=1|to=633|frame=1|Fragment_1
TVEHPNLLQLLAVCMTSQMMLITQLMPLGCLLDYVRTHREKIGSKALLNWSTQIARGMAY
LEERRLVHRDLAARNVLVQNPNCVKITDFGLAKLLDVNEDEYKAAGGKMPIKWLALECIQ
HRIFTHKSDVWAFGVTIWELLTYGARPYENISAKDVPELIENGLKLPQPAICTLDIYCIL
VSCWMLDAEARPSFKQLTETFAEKAADPGRY
>Locus_20651_Transcript_1/1_Confidence_1.000_Length_418|.|sp|P0CY46|
EGFR_APIME Epidermal growth factor receptor OS=Apis mellifera
GN=Egfr PE=2 SV=1|e=3.07229E-60|from=3|to=416|frame=3|Fragment_1
DHLLKDSGACVRSCPEKKKAVDGECVPCDGPCPKTCPASENIHAGNIDSFKDCTIIEGSL
EILDHTFKGYQQVYANFSFGPRYHEMHPDRLEVFSTLKEVTGFINIQGSHEAFTNLSYFR
NLETIGGRQLKENFFASL
>Locus_18908_Transcript_1/1_Confidence_1.000_Length_221|.|sp|P0CY46|
EGFR_APIME Epidermal growth factor receptor OS=Apis mellifera
GN=Egfr PE=2 SV=1|e=6.02979E-25|from=3|to=221|frame=-1|Fragment_1
LDAEARPSFKQLTETFAEKAADPGRYLYIPGDKFMRLPTFTTQDEKDLIKNLATTLDGPE
PVVEADEYLQPKS

>Locus_26399_Transcript_1/1_Confidence_1.000_Length_565|UNIGENE
REPRESENTATIVE TRANSCRIPT|sp|P10040|CRB_DROME Protein crumbs
OS=Drosophila melanogaster GN=crb PE=1 SV=3|e=1.17387E-45|from=1|
to=564|frame=1|Fragment_1
MCECPAGFNGDYCENNIDECLEQPCIHG
>Locus_26399_Transcript_1/1_Confidence_1.000_Length_565|UNIGENE
REPRESENTATIVE TRANSCRIPT|sp|P10040|CRB_DROME Protein crumbs
OS=Drosophila melanogaster GN=crb PE=1 SV=3|e=1.17387E-45|from=1|
to=564|frame=1|Fragment_2
VCIDRVAAFECDCSNTGYSGPLCERDIDECALTKVSCGKDGFCHNTPGSYRCVCTAPYCG
SGCRLLDPCTTGENPCRNGGTCTQTCTDRPSYECTCAEGFAGVNCTEEVSL
>Locus_26399_Transcript_1/1_Confidence_1.000_Length_565|UNIGENE
REPRESENTATIVE TRANSCRIPT|sp|P10040|CRB_DROME Protein crumbs
OS=Drosophila melanogaster GN=crb PE=1 SV=3|e=1.17387E-45|from=1|
to=564|frame=1|Fragment_3
GGPSGAD
>Locus_26399_Transcript_1/1_Confidence_1.000_Length_565|UNIGENE
REPRESENTATIVE TRANSCRIPT|sp|P10040|CRB_DROME Protein crumbs
OS=Drosophila melanogaster GN=crb PE=1 SV=3|e=1.17387E-45|from=1|
to=564|frame=1|Fragment_4
IMARSKRATRGTYSPSAQ
>Locus_26399_Transcript_1/1_Confidence_1.000_Length_565|.|sp|P10040|
CRB_DROME Protein crumbs OS=Drosophila melanogaster GN=crb PE=1
SV=3|e=1.49186E-24|from=4|to=399|frame=1|Fragment_1
CECPAGFNGDYCENNIDECLEQPCIH
>Locus_26399_Transcript_1/1_Confidence_1.000_Length_565|.|sp|P10040|
CRB_DROME Protein crumbs OS=Drosophila melanogaster GN=crb PE=1
SV=3|e=1.49186E-24|from=4|to=399|frame=1|Fragment_2
GVCIDRVAAFECDCSNTGYSGPLCERDIDECALTKVSCGKDGFCHNTPGSYRCVCTAPYC
GSGCRLLDPCTTGENPCRNGGTCTQTCTDRP
>Locus_26399_Transcript_1/1_Confidence_1.000_Length_565|.|sp|P10040|
CRB_DROME Protein crumbs OS=Drosophila melanogaster GN=crb PE=1
SV=3|e=1.49186E-24|from=4|to=399|frame=1|Fragment_3
SYECTCAEGFAGVNC
>Locus_26399_Transcript_1/1_Confidence_1.000_Length_565|.|sp|P10040|
CRB_DROME Protein crumbs OS=Drosophila melanogaster GN=crb PE=1
SV=3|e=1.49186E-24|from=4|to=399|frame=1|Fragment_1
CECPAGFNGDYCENNIDECLEQPCI
>Locus_26399_Transcript_1/1_Confidence_1.000_Length_565|.|sp|P10040|
CRB_DROME Protein crumbs OS=Drosophila melanogaster GN=crb PE=1
SV=3|e=1.49186E-24|from=4|to=399|frame=1|Fragment_2
HGVCIDRVAAFECDCSNTGYSGPLCERDIDECALTKVSCGKDGFCHNTPGSYRCVCTAPY
CGSGCRLLDPCTTGENPCRNGGTCTQ
>Locus_26399_Transcript_1/1_Confidence_1.000_Length_565|.|sp|P10040|
CRB_DROME Protein crumbs OS=Drosophila melanogaster GN=crb PE=1
SV=3|e=1.49186E-24|from=4|to=399|frame=1|Fragment_3
TCTDRPSYECTCAEGFAGVNC
>Locus_26399_Transcript_1/1_Confidence_1.000_Length_565|.|sp|P10040|
CRB_DROME Protein crumbs OS=Drosophila melanogaster GN=crb PE=1
SV=3|e=1.18207E-21|from=4|to=414|frame=1|Fragment_1
CECPAGFNGDYCENNIDECLEQPCIHG
>Locus_26399_Transcript_1/1_Confidence_1.000_Length_565|.|sp|P10040|
CRB_DROME Protein crumbs OS=Drosophila melanogaster GN=crb PE=1
SV=3|e=1.18207E-21|from=4|to=414|frame=1|Fragment_2

VCIDRVAAFECDCSNTGYSGPLCERDIDECALTKVSCGKDGFCHNTPGSYRCVCTAP
>Locus_26399_Transcript_1/1_Confidence_1.000_Length_565|.|sp|P10040|
CRB_DROME Protein crumbs OS=Drosophila melanogaster GN=crb PE=1
SV=3|e=1.18207E-21|from=4|to=414|frame=1|Fragment_3
YCGSGCRL
>Locus_26399_Transcript_1/1_Confidence_1.000_Length_565|.|sp|P10040|
CRB_DROME Protein crumbs OS=Drosophila melanogaster GN=crb PE=1
SV=3|e=1.18207E-21|from=4|to=414|frame=1|Fragment_4
LDPCTTGENPCRNGGTCTQTCTDRPSYECTCAEGFAGVNCTEEVS
>Locus_26399_Transcript_1/1_Confidence_1.000_Length_565|.|sp|P10040|
CRB_DROME Protein crumbs OS=Drosophila melanogaster GN=crb PE=1
SV=3|e=1.30693E-20|from=4|to=411|frame=1|Fragment_1
CECPAGFNGDYCENNIDECLEQP
>Locus_26399_Transcript_1/1_Confidence_1.000_Length_565|.|sp|P10040|
CRB_DROME Protein crumbs OS=Drosophila melanogaster GN=crb PE=1
SV=3|e=1.30693E-20|from=4|to=411|frame=1|Fragment_2
CIHGVCIDRVAAFECDCSNTGYSGPLCERDIDECALTKVSCGKDGFCHNTPGSYRCVCTA
PYCGSGCRL
>Locus_26399_Transcript_1/1_Confidence_1.000_Length_565|.|sp|P10040|
CRB_DROME Protein crumbs OS=Drosophila melanogaster GN=crb PE=1
SV=3|e=1.30693E-20|from=4|to=411|frame=1|Fragment_3
LDPCTTGENPCRNGGTCTQTCTDRPSYECTCAEGFAGVNCTEEV
>Locus_26399_Transcript_1/1_Confidence_1.000_Length_565|.|sp|P10040|
CRB_DROME Protein crumbs OS=Drosophila melanogaster GN=crb PE=1
SV=3|e=4.96634E-20|from=4|to=399|frame=1|Fragment_1
CECPAGFNGDYCENNIDECLEQPCIHG
>Locus_26399_Transcript_1/1_Confidence_1.000_Length_565|.|sp|P10040|
CRB_DROME Protein crumbs OS=Drosophila melanogaster GN=crb PE=1
SV=3|e=4.96634E-20|from=4|to=399|frame=1|Fragment_2
VCIDRVAAFECDCSNTGYSGPLCERDIDECALTKVSCGKDGFCHNTPGSYRCVCTAPYCG
SGCRL
>Locus_26399_Transcript_1/1_Confidence_1.000_Length_565|.|sp|P10040|
CRB_DROME Protein crumbs OS=Drosophila melanogaster GN=crb PE=1
SV=3|e=4.96634E-20|from=4|to=399|frame=1|Fragment_3
LDPCTTGENPCRNGGTCTQ
>Locus_26399_Transcript_1/1_Confidence_1.000_Length_565|.|sp|P10040|
CRB_DROME Protein crumbs OS=Drosophila melanogaster GN=crb PE=1
SV=3|e=4.96634E-20|from=4|to=399|frame=1|Fragment_4
TCTDRP
>Locus_26399_Transcript_1/1_Confidence_1.000_Length_565|.|sp|P10040|
CRB_DROME Protein crumbs OS=Drosophila melanogaster GN=crb PE=1
SV=3|e=4.96634E-20|from=4|to=399|frame=1|Fragment_5
SYECTCAEGFAGVNC
>Locus_26399_Transcript_1/1_Confidence_1.000_Length_565|.|sp|P10040|
CRB_DROME Protein crumbs OS=Drosophila melanogaster GN=crb PE=1
SV=3|e=4.96634E-20|from=4|to=417|frame=1|Fragment_1
CECPAGFNGDYCENNIDECLEQPCI
>Locus_26399_Transcript_1/1_Confidence_1.000_Length_565|.|sp|P10040|
CRB_DROME Protein crumbs OS=Drosophila melanogaster GN=crb PE=1
SV=3|e=4.96634E-20|from=4|to=417|frame=1|Fragment_2
HGVCIDRVAAFECDCSNTGYSGPLCERDIDECALTKVSCGKDGFCHNTPGSYRCVCTAPY
CGSGCR
>Locus_26399_Transcript_1/1_Confidence_1.000_Length_565|.|sp|P10040|
CRB_DROME Protein crumbs OS=Drosophila melanogaster GN=crb PE=1

SV=3|e=4.96634E-20|from=4|to=417|frame=1|Fragment_3
LLDPCTTGENPCRNGGTCTQTCTDRPS
>Locus_26399_Transcript_1/1_Confidence_1.000_Length_565|.|sp|P10040|
CRB_DROME Protein crumbs OS=Drosophila melanogaster GN=crb PE=1
SV=3|e=4.96634E-20|from=4|to=417|frame=1|Fragment_4
YECTCAEGFAGVNCTEEVSL
>Locus_26399_Transcript_1/1_Confidence_1.000_Length_565|.|sp|P10040|
CRB_DROME Protein crumbs OS=Drosophila melanogaster GN=crb PE=1
SV=3|e=1.59762E-18|from=4|to=399|frame=1|Fragment_1
CECPA
>Locus_26399_Transcript_1/1_Confidence_1.000_Length_565|.|sp|P10040|
CRB_DROME Protein crumbs OS=Drosophila melanogaster GN=crb PE=1
SV=3|e=1.59762E-18|from=4|to=399|frame=1|Fragment_2
GFNGDYCENNIDECLEQPCIHGV
>Locus_26399_Transcript_1/1_Confidence_1.000_Length_565|.|sp|P10040|
CRB_DROME Protein crumbs OS=Drosophila melanogaster GN=crb PE=1
SV=3|e=1.59762E-18|from=4|to=399|frame=1|Fragment_3
CIDRVAAFECDCSNTGYSGPLCERDIDECALTKVSCGKDGFC
>Locus_26399_Transcript_1/1_Confidence_1.000_Length_565|.|sp|P10040|
CRB_DROME Protein crumbs OS=Drosophila melanogaster GN=crb PE=1
SV=3|e=1.59762E-18|from=4|to=399|frame=1|Fragment_4
HNTPGSYRCVCTAPYCGSGCRL
>Locus_26399_Transcript_1/1_Confidence_1.000_Length_565|.|sp|P10040|
CRB_DROME Protein crumbs OS=Drosophila melanogaster GN=crb PE=1
SV=3|e=1.59762E-18|from=4|to=399|frame=1|Fragment_5
LDPCTTGENPCRNGGTCTQTCTDRPSYECTCAEGFAGVNC
>Locus_26399_Transcript_1/1_Confidence_1.000_Length_565|.|sp|P10040|
CRB_DROME Protein crumbs OS=Drosophila melanogaster GN=crb PE=1
SV=3|e=7.92889E-18|from=4|to=411|frame=1|Fragment_1
CECPAGFNGDYCENNIDECLEQPCIH
>Locus_26399_Transcript_1/1_Confidence_1.000_Length_565|.|sp|P10040|
CRB_DROME Protein crumbs OS=Drosophila melanogaster GN=crb PE=1
SV=3|e=7.92889E-18|from=4|to=411|frame=1|Fragment_2
GVCIDRVAAFECDCSN
>Locus_26399_Transcript_1/1_Confidence_1.000_Length_565|.|sp|P10040|
CRB_DROME Protein crumbs OS=Drosophila melanogaster GN=crb PE=1
SV=3|e=7.92889E-18|from=4|to=411|frame=1|Fragment_3
TGYSGPLCERDIDECALTKVSCGKDGFCHNTPGSYRCVCTAPYCGSGCRL
>Locus_26399_Transcript_1/1_Confidence_1.000_Length_565|.|sp|P10040|
CRB_DROME Protein crumbs OS=Drosophila melanogaster GN=crb PE=1
SV=3|e=7.92889E-18|from=4|to=411|frame=1|Fragment_4
LDPCTTGENPCRNGGTCTQTCTDRPSYECTC
>Locus_26399_Transcript_1/1_Confidence_1.000_Length_565|.|sp|P10040|
CRB_DROME Protein crumbs OS=Drosophila melanogaster GN=crb PE=1
SV=3|e=7.92889E-18|from=4|to=411|frame=1|Fragment_5
AEGFAGVNCTEEV
>Locus_26399_Transcript_1/1_Confidence_1.000_Length_565|.|sp|P10040|
CRB_DROME Protein crumbs OS=Drosophila melanogaster GN=crb PE=1
SV=3|e=2.47049E-11|from=58|to=399|frame=1|Fragment_1
CLEQPCI
>Locus_26399_Transcript_1/1_Confidence_1.000_Length_565|.|sp|P10040|
CRB_DROME Protein crumbs OS=Drosophila melanogaster GN=crb PE=1
SV=3|e=2.47049E-11|from=58|to=399|frame=1|Fragment_2
HGVCIDRVAAFECDCSNTGYSGPLCERDIDECALTKVSCGKDGFC

>Locus_26399_Transcript_1/1_Confidence_1.000_Length_565|.|sp|P10040|
CRB_DROME Protein crumbs OS=Drosophila melanogaster GN=crb PE=1
SV=3|e=2.47049E-11|from=58|to=399|frame=1|Fragment_3
HNTPGSYRCVCTAPYCGSGCRL
>Locus_26399_Transcript_1/1_Confidence_1.000_Length_565|.|sp|P10040|
CRB_DROME Protein crumbs OS=Drosophila melanogaster GN=crb PE=1
SV=3|e=2.47049E-11|from=58|to=399|frame=1|Fragment_4
LDP
>Locus_26399_Transcript_1/1_Confidence_1.000_Length_565|.|sp|P10040|
CRB_DROME Protein crumbs OS=Drosophila melanogaster GN=crb PE=1
SV=3|e=2.47049E-11|from=58|to=399|frame=1|Fragment_5
CTTGENPCRNGGTCTQTCTDRPSYECTCAEGFAGVNC

>Locus_7699_Transcript_5/12_Confidence_0.500_Length_2557|UNIGENE
REPRESENTATIVE TRANSCRIPT|sp|P11584|ITBX_DROME Integrin beta-PS
OS=Drosophila melanogaster GN=mys PE=1 SV=3|e=0|from=186|to=2498|
frame=3|Fragment_1
LLALFLFILNESNAQVAEKLTAQNPCIAKTTCSECIQTKSCAWCYQQDFGDRARCFQPSL
TSSSSICDERFTWNPDNEQRMILQEALS
>Locus_7699_Transcript_5/12_Confidence_0.500_Length_2557|UNIGENE
REPRESENTATIVE TRANSCRIPT|sp|P11584|ITBX_DROME Integrin beta-PS
OS=Drosophila melanogaster GN=mys PE=1 SV=3|e=0|from=186|to=2498|
frame=3|Fragment_2
KAHTHLGTGA
>Locus_7699_Transcript_5/12_Confidence_0.500_Length_2557|UNIGENE
REPRESENTATIVE TRANSCRIPT|sp|P11584|ITBX_DROME Integrin beta-PS
OS=Drosophila melanogaster GN=mys PE=1 SV=3|e=0|from=186|to=2498|
frame=3|Fragment_3
TQGQKEIVQISPQKVGLKLRINEVHKLNVYYAQAEDYPVDLYYLMDLSKSMEDDKERLSA
LGDLLSEAMRNITSNFRLGFGSFVDKVVMPYVSTVPKKLKAPCDNCEAPYGYRNHMPLSM
DTQLFSGEVRNASVSGNLDAPEGGFDAIMQAVVCRERIGWREKARRLLVFSTDAGFHYAG
DGKLGGIITPNDGECHMDANGMYSYSSLQDYPSISQVNQKVKQNAINVIFAVTESQVNVY
KKLSSHIEGSSTGELSNDSSNVVDLVKDQYSKISSSVEMKDNATSAVKIKYHSHCLSPGA
GLGQETNKCDGLKVGDVVKFTTEIVVTECPANISEWRQTIQIYPVGINESLIIDLEMLCD
CPCEHPGGEAYEPFSAKCGGAGTYMCGICDCDPAHFGRHCECSATDVTSHLDLAMGCRRD
NTTTVDCSGKGTCVCGVCECEQRANIEEQISGKYCECDNFSCDRHNGILCSGPEHGVCVC
GQCDCLPGWTGPACDCRDTNATCIAPGSTGEEMCSGHGVCECGVCKCDVAEDGRYSGRYC
EKCPTCPGRCQEFKECVMCQMYKTGPLTE
>Locus_7699_Transcript_5/12_Confidence_0.500_Length_2557|UNIGENE
REPRESENTATIVE TRANSCRIPT|sp|P11584|ITBX_DROME Integrin beta-PS
OS=Drosophila melanogaster GN=mys PE=1 SV=3|e=0|from=186|to=2498|
frame=3|Fragment_4
EECG
>Locus_7699_Transcript_5/12_Confidence_0.500_Length_2557|UNIGENE
REPRESENTATIVE TRANSCRIPT|sp|P11584|ITBX_DROME Integrin beta-PS
OS=Drosophila melanogaster GN=mys PE=1 SV=3|e=0|from=186|to=2498|
frame=3|Fragment_5
NCT
>Locus_7699_Transcript_5/12_Confidence_0.500_Length_2557|UNIGENE
REPRESENTATIVE TRANSCRIPT|sp|P11584|ITBX_DROME Integrin beta-PS
OS=Drosophila melanogaster GN=mys PE=1 SV=3|e=0|from=186|to=2498|
frame=3|Fragment_6
FVPIGQDVVEADEDKDEHLCSYYDEDDCRFTYVYSNEEDKVVVRAQNERECPPKVFM
>Locus_7699_Transcript_3/12_Confidence_0.219_Length_689|.|sp|P11584|

ITBX_DROME Integrin beta-PS OS=Drosophila melanogaster GN=mys PE=1 SV=3|e=6.66263E-82|from=1|to=630|frame=1|Fragment_1
TVDCSGKGTCVCGVCECEQRANIEEQISGKYCECDNFSCDRHNGILCSGPEHGVCVCGQC
DCLPGWTGPACDCRDTNATCIAPGSTGEEMCSGHGVCECGVCKCDVAEDGRYSGRYCEKC
PTCPGRCQEFKECVMCQMYKTGPLTE
>Locus_7699_Transcript_3/12_Confidence_0.219_Length_689|.|sp|P11584|
ITBX_DROME Integrin beta-PS OS=Drosophila melanogaster GN=mys PE=1
SV=3|e=6.66263E-82|from=1|to=630|frame=1|Fragment_2
EECG
>Locus_7699_Transcript_3/12_Confidence_0.219_Length_689|.|sp|P11584|
ITBX_DROME Integrin beta-PS OS=Drosophila melanogaster GN=mys PE=1
SV=3|e=6.66263E-82|from=1|to=630|frame=1|Fragment_3
NCT
>Locus_7699_Transcript_3/12_Confidence_0.219_Length_689|.|sp|P11584|
ITBX_DROME Integrin beta-PS OS=Drosophila melanogaster GN=mys PE=1
SV=3|e=6.66263E-82|from=1|to=630|frame=1|Fragment_4
FVPIGQDVVEADEDKDEHLCSYYDEDDCRFTYVYSNEEDKVVVRAQNERECPPKVFM
>Locus_7699_Transcript_4/12_Confidence_0.156_Length_458|.|sp|P11584|
ITBX_DROME Integrin beta-PS OS=Drosophila melanogaster GN=mys PE=1
SV=3|e=9.54484E-46|from=4|to=399|frame=1|Fragment_1
TCIAPGSTGEEMCSGHGVCECGVCKCDVAEDGRYSGRYCEKCPTCPGRCQEFKECVMCQM
YKTGPLTE
>Locus_7699_Transcript_4/12_Confidence_0.156_Length_458|.|sp|P11584|
ITBX_DROME Integrin beta-PS OS=Drosophila melanogaster GN=mys PE=1
SV=3|e=9.54484E-46|from=4|to=399|frame=1|Fragment_2
EECG
>Locus_7699_Transcript_4/12_Confidence_0.156_Length_458|.|sp|P11584|
ITBX_DROME Integrin beta-PS OS=Drosophila melanogaster GN=mys PE=1
SV=3|e=9.54484E-46|from=4|to=399|frame=1|Fragment_3
NCT
>Locus_7699_Transcript_4/12_Confidence_0.156_Length_458|.|sp|P11584|
ITBX_DROME Integrin beta-PS OS=Drosophila melanogaster GN=mys PE=1
SV=3|e=9.54484E-46|from=4|to=399|frame=1|Fragment_4
FVPIGQDVVEADEDKDEHLCSYYDEDDCRFTYVYSNEEDKVVVRAQNERECPPKVFM
>Locus_11779_Transcript_1/2_Confidence_0.800_Length_437|.|sp|P11584|
ITBX_DROME Integrin beta-PS OS=Drosophila melanogaster GN=mys PE=1
SV=3|e=2.62048E-55|from=3|to=437|frame=-1|Fragment_1
DCDPAHFGRHCECSATDVTSHLDLAMGCRRDNTTTVDCSGKGTCVCGVCECEQRANIEEQ
ISGKYCECDNFSCDRHNGILCSGPEHGVCVCGQCDCLPGWTGPACDCRDTNATCIAPGST
GEEMCSGHGVCECGVCKCDVAEDGR
>Locus_15585_Transcript_1/1_Confidence_1.000_Length_437|.|sp|P11584|
ITBX_DROME Integrin beta-PS OS=Drosophila melanogaster GN=mys PE=1
SV=3|e=2.62048E-55|from=1|to=435|frame=1|Fragment_1
DCDPAHFGRHCECSATDVTSHLDLAMGCRRDNTTTVDCSGKGTCVCGVCECEQRANIEEQ
ISGKYCECDNFSCDRHNGILCSGPEHGVCVCGQCDCLPGWTGPACDCRDTNATCIAPGST
GEEMCSGHGVCECGVCKCDVAEDGR
>Locus_11779_Transcript_2/2_Confidence_0.600_Length_218|.|sp|P11584|
ITBX_DROME Integrin beta-PS OS=Drosophila melanogaster GN=mys PE=1
SV=3|e=9.65325E-23|from=3|to=218|frame=-1|Fragment_1
DCDPAHFGRHCECSATDVTSHLDLAMGCRRDNTTTVDCSGKGTCVCGVCECEQRANIEEQ
ISGKYCECDNFS

>Locus_1711_Transcript_6/11_Confidence_0.593_Length_4778|UNIGENE
REPRESENTATIVE TRANSCRIPT|sp|P13607|ATNA_DROME Sodium/potassium-

transporting ATPase subunit alpha OS=Drosophila melanogaster
GN=Atpalpha PE=1 SV=3|e=0|from=1761|to=4349|frame=-1|Fragment_1
DDKHGRSDSYRVATV
>Locus_1711_Transcript_6/11_Confidence_0.593_Length_4778|UNIGENE
REPRESENTATIVE TRANSCRIPT|sp|P13607|ATNA_DROME Sodium/potassium-
transporting ATPase subunit alpha OS=Drosophila melanogaster
GN=Atpalpha PE=1 SV=3|e=0|from=1761|to=4349|frame=-1|Fragment_2
PTIDDNLTADGQYKSRRKTPTKKQRKEGELDDLKQELDIDFHKVSPEELYQRFNTHPENG
LSHAKAKENLERDGPNALTPPKQTPEWVKFCKNLFGGFALLLWIGAILCFVAYSIQASTV
EEPADDNLYLGIVLAAVVIVTGIFSYYQESKSSKIMESFKNMVPQFATVLREGEKLTLRA
EDLVLGDVVEVKFGDRIPADIRIIESRGFKVDNSSLTGESEPQSRGPEFTNENPLETKNL
AFFSTNAVEGTAKGVVISCGDNTVMGRIAGLASGLDTGETPIAKEIHHFIHLITGVAVFL
GVTFFVIAIILNYHWLDAVIFLIGIIVANVPEG
>Locus_1711_Transcript_6/11_Confidence_0.593_Length_4778|UNIGENE
REPRESENTATIVE TRANSCRIPT|sp|P13607|ATNA_DROME Sodium/potassium-
transporting ATPase subunit alpha OS=Drosophila melanogaster
GN=Atpalpha PE=1 SV=3|e=0|from=1761|to=4349|frame=-1|Fragment_3
AKRMASKNCLVKNLEAVETLGSTSTICSDKTGTLTQNRMTVAHMWFDNQIIEADTTEDQS
GVVYDRTSPGFKALARIATLCNRAEFKGGQEGVPILKKEVSGDASEAALLKCMELALGDV
MSIRKRNKKVCEIPFNSTNKYQVSIHETEDASDPRHVMVMKGAPERILEKCSTIFIGGKE
KLLDEEMKEAFNNAY
>Locus_1711_Transcript_6/11_Confidence_0.593_Length_4778|UNIGENE
REPRESENTATIVE TRANSCRIPT|sp|P13607|ATNA_DROME Sodium/potassium-
transporting ATPase subunit alpha OS=Drosophila melanogaster
GN=Atpalpha PE=1 SV=3|e=0|from=1761|to=4349|frame=-1|Fragment_4
GFCDLMLPTDKFPLGFKFDSDDPNFPIENLRFVGLMSMI
>Locus_1711_Transcript_6/11_Confidence_0.593_Length_4778|UNIGENE
REPRESENTATIVE TRANSCRIPT|sp|P13607|ATNA_DROME Sodium/potassium-
transporting ATPase subunit alpha OS=Drosophila melanogaster
GN=Atpalpha PE=1 SV=3|e=0|from=1761|to=4349|frame=-1|Fragment_5
AKCRSAGIKVIMVTGDHPITAKAIAKSVGIISEGNETVEDIAQRLNIPVSEVNPREAKAA
VVHGTELRELNSDQLDEILRYHTEIVFARTSPQQKLIIVEGCQRMGAIVAVTGDGVNDSP
ALKKADIGVAMGIAGSDVSKQAADMILLDDNFASIVTGVEEGRLIFDNLKKSIAYTLTSN
IPEISPFLAFILCDIPLPLGTVTILCIDLGTDMVPAISLAYEESESDIMKRRPRNPYSDK
LVNERCL
>Locus_1711_Transcript_6/11_Confidence_0.593_Length_4778|.|sp|
P13607|ATNA_DROME Sodium/potassium-transporting ATPase subunit alpha
OS=Drosophila melanogaster GN=Atpalpha PE=1 SV=3|e=1.4074E-111|
from=1147|to=1767|frame=-3|Fragment_1
VPAISLAYEHAEADIMKRPPRDPVNDKLVNSRLISMAYGQIGMIQAAAGFFVYFVIMAEN
GFLPMKLFGIRKQWDSKAVNDLTDSYGQEWTYRDRKTLEYTCHTAFFVSIVVVQWADLIV
CKTRRNSLLHQGMRNWALNFGLVFETALAAFLSYTPGMDKGLRMFPLKFVWWLPALPFMI
SIFIYDETRRFYLRRNPGGWLEQETYY
>Locus_1711_Transcript_3/11_Confidence_0.519_Length_4684|.|sp|
P13607|ATNA_DROME Sodium/potassium-transporting ATPase subunit alpha
OS=Drosophila melanogaster GN=Atpalpha PE=1 SV=3|e=0|from=1148|
to=4255|frame=-1|Fragment_1
DDKHGRSDSYRVATV
>Locus_1711_Transcript_3/11_Confidence_0.519_Length_4684|.|sp|
P13607|ATNA_DROME Sodium/potassium-transporting ATPase subunit alpha
OS=Drosophila melanogaster GN=Atpalpha PE=1 SV=3|e=0|from=1148|
to=4255|frame=-1|Fragment_2
PTIDDNLTADGQYKSRRKTPTKKQRKEGELDDLKQELDIDFHKVSPEELYQRFNTHPENG
LSHAKAKENLERDGPNALTPPKQTPEWVKFCKNLFGGFALLLWIGAILCFVAYSIQASTV

EEPADDNLYLGIVLAAVVIVTGIFSYYQESKSSKIMESFKNMVPQFATVLREGEKLTLRA
EDLVLGDVVEVKFGDRIPADIRIIESRGFKVDNSSLTGESEPQSRGPEFTNENPLETKNL
AFFSTNAVEGTAKGVVISCGDNTVMGRIAGLASGLDTGETPIAKEIHHFIHLITGVAVFL
GVTFFVIAIILNYHWLDAVIFLIGIIVANVPEG
>Locus_1711_Transcript_3/11_Confidence_0.519_Length_4684|.|sp|
P13607|ATNA_DROME Sodium/potassium-transporting ATPase subunit alpha
OS=Drosophila melanogaster GN=Atpalpha PE=1 SV=3|e=0|from=1148|
to=4255|frame=-1|Fragment_3
AKRMASKNCLVKNLEAVETLGSTSTICSDKTGTLTQNRMTVAHMWFDNQIIEADTTEDQS
GVVYDRTSPGFKALARIATLCNRAEFKGGQEGVPILKKEVSGDASEAALLKCMELALGDV
MSIRKRNKKVCEIPFNSTNKYQVSIHETEDASDPRHVMVMKGAPERILEKCSTIFIGGKE
KLLDEEMKEAFNNAY
>Locus_1711_Transcript_3/11_Confidence_0.519_Length_4684|.|sp|
P13607|ATNA_DROME Sodium/potassium-transporting ATPase subunit alpha
OS=Drosophila melanogaster GN=Atpalpha PE=1 SV=3|e=0|from=1148|
to=4255|frame=-1|Fragment_4
GFCDLMLPTDKFPLGFKFDSDDPNFPIENLRFVGLMSMI
>Locus_1711_Transcript_3/11_Confidence_0.519_Length_4684|.|sp|
P13607|ATNA_DROME Sodium/potassium-transporting ATPase subunit alpha
OS=Drosophila melanogaster GN=Atpalpha PE=1 SV=3|e=0|from=1148|
to=4255|frame=-1|Fragment_5
AKCRSAGIKVIMVTGDHPITAKAIAKSVGIISEGNETVEDIAQRLNIPVSEVNPREAKAA
VVHGTELRELNSDQLDEILRYHTEIVFARTSPQQKLIIVEGCQRMGAIVAVTGDGVNDSP
ALKKADIGVAMGIAGSDVSKQAADMILLDDNFASIVTGVEEGRLIFDNLKKSIAYTLTSN
IPEISPFLAFILCDIPLPLGTVTILCIDLGTDMVPAISLAYEHAEADIMKRPPRDPVNDK
LVNSRLISMAYGQIGMIQAAAGFFVYFVIMAENGFLPMKLFGIRKQWDSKAVNDLTDSYG
QEWTYRDRKTLEYTCHTAFFVSIVVVQWADLIVCKTRRNSLLHQGMRNWALNFGLVFETA
LAAFLSYTPGMDKGLRMFPLKFVWWLPALPFMISIFIYDETRRFYLRRNPGGWLEQETYY
>Locus_1711_Transcript_4/11_Confidence_0.481_Length_4683|.|sp|
P13607|ATNA_DROME Sodium/potassium-transporting ATPase subunit alpha
OS=Drosophila melanogaster GN=Atpalpha PE=1 SV=3|e=0|from=1147|
to=4254|frame=-1|Fragment_1
DDKHGRSDSYRVATV
>Locus_1711_Transcript_4/11_Confidence_0.481_Length_4683|.|sp|
P13607|ATNA_DROME Sodium/potassium-transporting ATPase subunit alpha
OS=Drosophila melanogaster GN=Atpalpha PE=1 SV=3|e=0|from=1147|
to=4254|frame=-1|Fragment_2
PTIDDNLTADGQYKSRRKTPTKKQRKEGELDDLKQELDIDFHKVSPEELYQRFNTHPENG
LSHAKAKENLERDGPNALTPPKQTPEWVKFCKNLFGGFALLLWIGAILCFVAYSIQASTV
EEPADDNLYLGIVLAAVVIVTGIFSYYQESKSSKIMESFKNMVPQFATVLREGEKLTLRA
EDLVLGDVVEVKFGDRIPADIRIIESRGFKVDNSSLTGESEPQSRGPEFTNENPLETKNL
AFFSTNAVEGTAKGVVISCGDNTVMGRIAGLASGLDTGETPIAKEIHHFIHLITGVAVFL
GVTFFVIAIILNYHWLDAVIFLIGIIVANVPEG
>Locus_1711_Transcript_4/11_Confidence_0.481_Length_4683|.|sp|
P13607|ATNA_DROME Sodium/potassium-transporting ATPase subunit alpha
OS=Drosophila melanogaster GN=Atpalpha PE=1 SV=3|e=0|from=1147|
to=4254|frame=-1|Fragment_3
AKRMASKNCLVKNLEAVETLGSTSTICSDKTGTLTQNRMTVAHMWFDNQIIEADTTEDQS
GVVYDRTSPGFKALARIATLCNRAEFKGGQEGVPILKKEVSGDASEAALLKCMELALGDV
MSIRKRNKKVCEIPFNSTNKYQVSIHETEDASDPRHVMVMKGAPERILEKCSTIFIGGKE
KLLDEEMKEAFNNAY
>Locus_1711_Transcript_4/11_Confidence_0.481_Length_4683|.|sp|
P13607|ATNA_DROME Sodium/potassium-transporting ATPase subunit alpha
OS=Drosophila melanogaster GN=Atpalpha PE=1 SV=3|e=0|from=1147|

to=4254|frame=-1|Fragment_4
GFCDLMLPTDKFPLGFKFDSDDPNFPIENLRFVGLMSMI
>Locus_1711_Transcript_4/11_Confidence_0.481_Length_4683|.|sp|
P13607|ATNA_DROME Sodium/potassium-transporting ATPase subunit alpha
OS=Drosophila melanogaster GN=Atpalpha PE=1 SV=3|e=0|from=1147|
to=4254|frame=-1|Fragment_5
AKCRSAGIKVIMVTGDHPITAKAIAKSVGIISEGNETVEDIAQRLNIPVSEVNPREAKAA
VVHGTELRELNSDQLDEILRYHTEIVFARTSPQQKLIIVEGCQRMGAIVAVTGDGVNDSP
ALKKADIGVAMGIAGSDVSKQAADMILLDDNFASIVTGVEEGRLIFDNLKKSIAYTLTSN
IPEISPFLAFILCDIPLPLGTVTILCIDLGTDMVPAISLAYEESESDIMKRRPRNPYSDK
LVNERLISMAYGQIGMIQAAAGFFVYFVIMAENGFLPMKLFGIRKQWDSKAVNDLTDSYG
QEWTYRDRKTLEYTCHTAFFVSIVVVQWADLIVCKTRRNSLLHQGMRNWALNFGLVFETA
LAAFLSYTPGMDKGLRMFPLKFVWWLPALPFMISIFIYDETRRFYLRRNPGGWLEQETYY
>Locus_1711_Transcript_5/11_Confidence_0.481_Length_4683|.|sp|
P13607|ATNA_DROME Sodium/potassium-transporting ATPase subunit alpha
OS=Drosophila melanogaster GN=Atpalpha PE=1 SV=3|e=0|from=1147|
to=4254|frame=-1|Fragment_1
DDKHGRSDSYRVATV
>Locus_1711_Transcript_5/11_Confidence_0.481_Length_4683|.|sp|
P13607|ATNA_DROME Sodium/potassium-transporting ATPase subunit alpha
OS=Drosophila melanogaster GN=Atpalpha PE=1 SV=3|e=0|from=1147|
to=4254|frame=-1|Fragment_2
PTIDDNLTADGQYKSRRKTPTKKQRKEGELDDLKQELDIDFHKVSPEELYQRFNTHPENG
LSHAKAKENLERDGPNALTPPKQTPEWVKFCKNLFGGFALLLWIGAILCFVAYSIQASTV
EEPADDNLYLGIVLAAVVIVTGIFSYYQESKSSKIMESFKNMVPQFATVLREGEKLTLRA
EDLVLGDVVEVKFGDRIPADIRIIESRGFKVDNSSLTGESEPQSRGPEFTNENPLETKNL
AFFSTNAVEGTAKGVVISCGDNTVMGRIAGLASGLDTGETPIAKEIHHFIHLITGVAVFL
GVTFFVIAIILNYHWLDAVIFLIGIIVANVPEG
>Locus_1711_Transcript_5/11_Confidence_0.481_Length_4683|.|sp|
P13607|ATNA_DROME Sodium/potassium-transporting ATPase subunit alpha
OS=Drosophila melanogaster GN=Atpalpha PE=1 SV=3|e=0|from=1147|
to=4254|frame=-1|Fragment_3
AKRMASKNCLVKNLEAVETLGSTSTICSDKTGTLTQNRMTVAHMWFDNQIIEADTTEDQS
GVVYDRTSPGFKALARIATLCNRAEFKGGQEGVPILKKEVSGDASEAALLKCMELALGDV
MSIRKRNKKVCEIPFNSTNKYQVSIHETEDASDPRHVMVMKGAPERILEKCSTIFIGGKE
KLLDEEMKEAFNNAY
>Locus_1711_Transcript_5/11_Confidence_0.481_Length_4683|.|sp|
P13607|ATNA_DROME Sodium/potassium-transporting ATPase subunit alpha
OS=Drosophila melanogaster GN=Atpalpha PE=1 SV=3|e=0|from=1147|
to=4254|frame=-1|Fragment_4
GFCDLMLPTDKFPLGFKFDSDDPNFPIENLRFVGLMSMI
>Locus_1711_Transcript_5/11_Confidence_0.481_Length_4683|.|sp|
P13607|ATNA_DROME Sodium/potassium-transporting ATPase subunit alpha
OS=Drosophila melanogaster GN=Atpalpha PE=1 SV=3|e=0|from=1147|
to=4254|frame=-1|Fragment_5
AKCRSAGIKVIMVTGDHPITAKAIAKSVGIISEGNETVEDIAQRLNIPVSEVNPREAKAA
VVHGTELRELNSDQLDEILRYHTEIVFARTSPQQKLIIVEGCQRMGAIVAVTGDGVNDSP
ALKKADIGVAMGIAGSDVSKQAADMILLDDNFASIVTGVEEGRLIFDNLKKSIAYTLTSN
IPEISPFLAFILCDIPLPLGTVTILCIDLGTDMVPAISLAYEAPESDIMKRQPRDPYRDN
LVNRRLISMAYGQIGMIQAAAGFFVYFVIMAENGFLPMKLFGIRKQWDSKAVNDLTDSYG
QEWTYRDRKTLEYTCHTAFFVSIVVVQWADLIVCKTRRNSLLHQGMRNWALNFGLVFETA
LAAFLSYTPGMDKGLRMFPLKFVWWLPALPFMISIFIYDETRRFYLRRNPGGWLEQETYY
>Locus_1711_Transcript_10/11_Confidence_0.481_Length_4683|.|sp|
P13607|ATNA_DROME Sodium/potassium-transporting ATPase subunit alpha

OS=Drosophila melanogaster GN=Atpalpha PE=1 SV=3|e=0|from=1147|
to=4254|frame=-1|Fragment_1
DDKHGRSDSYRVATV
>Locus_1711_Transcript_10/11_Confidence_0.481_Length_4683|.|sp|
P13607|ATNA_DROME Sodium/potassium-transporting ATPase subunit alpha
OS=Drosophila melanogaster GN=Atpalpha PE=1 SV=3|e=0|from=1147|
to=4254|frame=-1|Fragment_2
PTIDDNLTADGQYKSRRKTPTKKQRKEGELDDLKQELDIDFHKVSPEELYQRFNTHPENG
LSHAKAKENLERDGPNALTPPKQTPEWVKFCKNLFGGFALLLWIGAILCFVAYSIQASTV
EEPADDNLYLGIVLAAVVIVTGIFSYYQESKSSKIMESFKNMVPQFATVLREGEKLTLRA
EDLVLGDVVEVKFGDRIPADIRIIESRGFKVDNSSLTGESEPQSRGPEFTNENPLETKNL
AFFSTNAVEGTAKGVVISCGDNTVMGRIAGLASGLDTGETPIAKEIHHFIHLITGVAVFL
GVTFFVIAIILNYHWLDAVIFLIGIIVANVPEG
>Locus_1711_Transcript_10/11_Confidence_0.481_Length_4683|.|sp|
P13607|ATNA_DROME Sodium/potassium-transporting ATPase subunit alpha
OS=Drosophila melanogaster GN=Atpalpha PE=1 SV=3|e=0|from=1147|
to=4254|frame=-1|Fragment_3
AKRMASKNCLVKNLEAVETLGSTSTICSDKTGTLTQNRMTVAHMWFDNQIIEADTTEDQS
GVVYDRTSPGFKALARIATLCNRAEFKGGQEGVPILKKEVSGDASEAALLKCMELALGDV
MSIRKRNKKVCEIPFNSTNKYQVSIHETEDASDPRHVMVMKGAPERILEKCSTIFIGGKE
KLLDEEMKEAFNNAY
>Locus_1711_Transcript_10/11_Confidence_0.481_Length_4683|.|sp|
P13607|ATNA_DROME Sodium/potassium-transporting ATPase subunit alpha
OS=Drosophila melanogaster GN=Atpalpha PE=1 SV=3|e=0|from=1147|
to=4254|frame=-1|Fragment_4
GFCDLMLPTDKFPLGFKFDSDDPNFPIENLRFVGLMSMI
>Locus_1711_Transcript_10/11_Confidence_0.481_Length_4683|.|sp|
P13607|ATNA_DROME Sodium/potassium-transporting ATPase subunit alpha
OS=Drosophila melanogaster GN=Atpalpha PE=1 SV=3|e=0|from=1147|
to=4254|frame=-1|Fragment_5
AKCRSAGIKVIMVTGDHPITAKAIAKSVGIISEGNETVEDIAQRLNIPVSEVNPREAKAA
VVHGTELRELNSDQLDEILRYHTEIVFARTSPQQKLIIVEGCQRMGAIVAVTGDGVNDSP
ALKKADIGVAMGIAGSDVSKQAADMILLDDNFASIVTGVEEGRLIFDNLKKSIAYTLTSN
IPEISPFLAFILCDIPLPLGTVTILCIDLGTDMVPAISLAYEHAEADIMKRPPRDPVNDK
LVNSRLISMAYGQIGMIQAAAGFFVYFVIMAENGFLPMKLFGIRKQWDSKAVNDLTDSYG
QEWTYRDRKTLEYTCHTAFFVSIVVVQWADLIVCKTRRNSLLHQGMRNWALNFGLVFETA
LAAFLSYTPGMDKGLRMFPLKFVWWLPALPFMISIFIYDETRRFYLRRNPGGWLEQETYY
>Locus_1711_Transcript_11/11_Confidence_0.519_Length_4683|.|sp|
P13607|ATNA_DROME Sodium/potassium-transporting ATPase subunit alpha
OS=Drosophila melanogaster GN=Atpalpha PE=1 SV=3|e=0|from=1147|
to=4254|frame=-1|Fragment_1
DDKHGRSDSYRVATV
>Locus_1711_Transcript_11/11_Confidence_0.519_Length_4683|.|sp|
P13607|ATNA_DROME Sodium/potassium-transporting ATPase subunit alpha
OS=Drosophila melanogaster GN=Atpalpha PE=1 SV=3|e=0|from=1147|
to=4254|frame=-1|Fragment_2
PTIDDNLTADGQYKSRRKTPTKKQRKEGELDDLKQELDIDFHKVSPEELYQRFNTHPENG
LSHAKAKENLERDGPNALTPPKQTPEWVKFCKNLFGGFALLLWIGAILCFVAYSIQASTV
EEPADDNLYLGIVLAAVVIVTGIFSYYQESKSSKIMESFKNMVPQFATVLREGEKLTLRA
EDLVLGDVVEVKFGDRIPADIRIIESRGFKVDNSSLTGESEPQSRGPEFTNENPLETKNL
AFFSTNAVEGTAKGVVISCGDNTVMGRIAGLASGLDTGETPIAKEIHHFIHLITGVAVFL
GVTFFVIAIILNYHWLDAVIFLIGIIVANVPEG
>Locus_1711_Transcript_11/11_Confidence_0.519_Length_4683|.|sp|
P13607|ATNA_DROME Sodium/potassium-transporting ATPase subunit alpha

OS=Drosophila melanogaster GN=Atpalpha PE=1 SV=3|e=0|from=1147|
to=4254|frame=-1|Fragment_3
AKRMASKNCLVKNLEAVETLGSTSTICSDKTGTLTQNRMTVAHMWFDNQIIEADTTEDQS
GVVYDRTSPGFKALARIATLCNRAEFKGGQEGVPILKKEVSGDASEAALLKCMELALGDV
MSIRKRNKKVCEIPFNSTNKYQVSIHETEDASDPRHVMVMKGAPERILEKCSTIFIGGKE
KLLDEEMKEAFNNAY
>Locus_1711_Transcript_11/11_Confidence_0.519_Length_4683|.|sp|
P13607|ATNA_DROME Sodium/potassium-transporting ATPase subunit alpha
OS=Drosophila melanogaster GN=Atpalpha PE=1 SV=3|e=0|from=1147|
to=4254|frame=-1|Fragment_4
GFCDLMLPTDKFPLGFKFDSDDPNFPIENLRFVGLMSMI
>Locus_1711_Transcript_11/11_Confidence_0.519_Length_4683|.|sp|
P13607|ATNA_DROME Sodium/potassium-transporting ATPase subunit alpha
OS=Drosophila melanogaster GN=Atpalpha PE=1 SV=3|e=0|from=1147|
to=4254|frame=-1|Fragment_5
AKCRSAGIKVIMVTGDHPITAKAIAKSVGIISEGNETVEDIAQRLNIPVSEVNPREAKAA
VVHGTELRELNSDQLDEILRYHTEIVFARTSPQQKLIIVEGCQRMGAIVAVTGDGVNDSP
ALKKADIGVAMGIAGSDVSKQAADMILLDDNFASIVTGVEEGRLIFDNLKKSIAYTLTSN
IPEISPFLAFILCDIPLPLGTVTILCIDLGTDMVPAISLAYEHAEADIMKRPPRDPVNDK
LVNSRLISMAYGQIGMIQAAAGFFVYFVIMAENGFLPMKLFGIRKQWDSKAVNDLTDSYG
QEWTYRDRKTLEYTCHTAFFVSIVVVQWADLIVCKTRRNSLLHQGMRNWALNFGLVFETA
LAAFLSYTPGMDKGLRMFPLKFVWWLPALPFMISIFIYDETRRFYLRRNPGGWLEQETYY
>Locus_1711_Transcript_7/11_Confidence_0.481_Length_4661|.|sp|
P13607|ATNA_DROME Sodium/potassium-transporting ATPase subunit alpha
OS=Drosophila melanogaster GN=Atpalpha PE=1 SV=3|e=0|from=1125|
to=4232|frame=-1|Fragment_1
DDKHGRSDSYRVATV
>Locus_1711_Transcript_7/11_Confidence_0.481_Length_4661|.|sp|
P13607|ATNA_DROME Sodium/potassium-transporting ATPase subunit alpha
OS=Drosophila melanogaster GN=Atpalpha PE=1 SV=3|e=0|from=1125|
to=4232|frame=-1|Fragment_2
PTIDDNLTADGQYKSRRKTPTKKQRKEGELDDLKQELDIDFHKVSPEELYQRFNTHPENG
LSHAKAKENLERDGPNALTPPKQTPEWVKFCKNLFGGFALLLWIGAILCFVAYSIQASTV
EEPADDNLYLGIVLAAVVIVTGIFSYYQESKSSKIMESFKNMVPQFATVLREGEKLTLRA
EDLVLGDVVEVKFGDRIPADIRIIESRGFKVDNSSLTGESEPQSRGPEFTNENPLETKNL
AFFSTNAVEGTAKGVVISCGDNTVMGRIAGLASGLDTGETPIAKEIHHFIHLITGVAVFL
GVTFFVIAIILNYHWLDAVIFLIGIIVANVPEG
>Locus_1711_Transcript_7/11_Confidence_0.481_Length_4661|.|sp|
P13607|ATNA_DROME Sodium/potassium-transporting ATPase subunit alpha
OS=Drosophila melanogaster GN=Atpalpha PE=1 SV=3|e=0|from=1125|
to=4232|frame=-1|Fragment_3
AKRMASKNCLVKNLEAVETLGSTSTICSDKTGTLTQNRMTVAHMWFDNQIIEADTTEDQS
GVVYDRTSPGFKALARIATLCNRAEFKGGQEGVPILKKEVSGDASEAALLKCMELALGDV
MSIRKRNKKVCEIPFNSTNKYQVSIHETEDASDPRHVMVMKGAPERILEKCSTIFIGGKE
KLLDEEMKEAFNNAY
>Locus_1711_Transcript_7/11_Confidence_0.481_Length_4661|.|sp|
P13607|ATNA_DROME Sodium/potassium-transporting ATPase subunit alpha
OS=Drosophila melanogaster GN=Atpalpha PE=1 SV=3|e=0|from=1125|
to=4232|frame=-1|Fragment_4
GFCDLMLPTDKFPLGFKFDSDDPNFPIENLRFVGLMSMI
>Locus_1711_Transcript_7/11_Confidence_0.481_Length_4661|.|sp|
P13607|ATNA_DROME Sodium/potassium-transporting ATPase subunit alpha
OS=Drosophila melanogaster GN=Atpalpha PE=1 SV=3|e=0|from=1125|
to=4232|frame=-1|Fragment_5

AKCRSAGIKVIMVTGDHPITAKAIAKSVGIISEGNETVEDIAQRLNIPVSEVNPREAKAA
VVHGTELRELNSDQLDEILRYHTEIVFARTSPQQKLIIVEGCQRMGAIVAVTGDGVNDSP
ALKKADIGVAMGIAGSDVSKQAADMILLDDNFASIVTGVEEGRLIFDNLKKSIAYTLTSN
IPEISPFLAFILCDIPLPLGTVTILCIDLGTDMVPAISLAYEHAEADIMKRPPRDPVNDK
LVNSRLISMAYGQIGMIQAAAGFFVYFVIMAENGFLPMKLFGIRKQWDSKAVNDLTDSYG
QEWTYRDRKTLEYTCHTAFFVSIVVVQWADLIVCKTRRNSLLHQGMRNWALNFGLVFETA
LAAFLSYTPGMDKGLRMFPLKFVWWLPALPFMISIFIYDETRRFYLRRNPGGWLEQETYY
>Locus_1711_Transcript_9/11_Confidence_0.519_Length_4623|.|sp|
P13607|ATNA_DROME Sodium/potassium-transporting ATPase subunit alpha
OS=Drosophila melanogaster GN=Atpalpha PE=1 SV=3|e=0|from=1147|
to=4254|frame=-1|Fragment_1
DDKHGRSDSYRVATV
>Locus_1711_Transcript_9/11_Confidence_0.519_Length_4623|.|sp|
P13607|ATNA_DROME Sodium/potassium-transporting ATPase subunit alpha
OS=Drosophila melanogaster GN=Atpalpha PE=1 SV=3|e=0|from=1147|
to=4254|frame=-1|Fragment_2
PTIDDNLTADGQYKSRRKTPTKKQRKEGELDDLKQELDIDFHKVSPEELYQRFNTHPENG
LSHAKAKENLERDGPNALTPPKQTPEWVKFCKNLFGGFALLLWIGAILCFVAYSIQASTV
EEPADDNLYLGIVLAAVVIVTGIFSYYQESKSSKIMESFKNMVPQFATVLREGEKLTLRA
EDLVLGDVVEVKFGDRIPADIRIIESRGFKVDNSSLTGESEPQSRGPEFTNENPLETKNL
AFFSTNAVEGTAKGVVISCGDNTVMGRIAGLASGLDTGETPIAKEIHHFIHLITGVAVFL
GVTFFVIAIILNYHWLDAVIFLIGIIVANVPEG
>Locus_1711_Transcript_9/11_Confidence_0.519_Length_4623|.|sp|
P13607|ATNA_DROME Sodium/potassium-transporting ATPase subunit alpha
OS=Drosophila melanogaster GN=Atpalpha PE=1 SV=3|e=0|from=1147|
to=4254|frame=-1|Fragment_3
AKRMASKNCLVKNLEAVETLGSTSTICSDKTGTLTQNRMTVAHMWFDNQIIEADTTEDQS
GVVYDRTSPGFKALARIATLCNRAEFKGGQEGVPILKKEVSGDASEAALLKCMELALGDV
MSIRKRNKKVCEIPFNSTNKYQVSIHETEDASDPRHVMVMKGAPERILEKCSTIFIGGKE
KLLDEEMKEAFNNAY
>Locus_1711_Transcript_9/11_Confidence_0.519_Length_4623|.|sp|
P13607|ATNA_DROME Sodium/potassium-transporting ATPase subunit alpha
OS=Drosophila melanogaster GN=Atpalpha PE=1 SV=3|e=0|from=1147|
to=4254|frame=-1|Fragment_4
GFCDLMLPTDKFPLGFKFDSDDPNFPIENLRFVGLMSMI
>Locus_1711_Transcript_9/11_Confidence_0.519_Length_4623|.|sp|
P13607|ATNA_DROME Sodium/potassium-transporting ATPase subunit alpha
OS=Drosophila melanogaster GN=Atpalpha PE=1 SV=3|e=0|from=1147|
to=4254|frame=-1|Fragment_5
AKCRSAGIKVIMVTGDHPITAKAIAKSVGIISEGNETVEDIAQRLNIPVSEVNPREAKAA
VVHGTELRELNSDQLDEILRYHTEIVFARTSPQQKLIIVEGCQRMGAIVAVTGDGVNDSP
ALKKADIGVAMGIAGSDVSKQAADMILLDDNFASIVTGVEEGRLIFDNLKKSIAYTLTSN
IPEISPFLAFILCDIPLPLGTVTILCIDLGTDMVPAISLAYEHAEADIMKRPPRDPVNDK
LVNSRLISMAYGQIGMIQAAAGFFVYFVIMAENGFLPMKLFGIRKQWDSKAVNDLTDSYG
QEWTYRDRKTLEYTCHTAFFVSIVVVQWADLIVCKTRRNSLLHQGMRNWALNFGLVFETA
LAAFLSYTPGMDKGLRMFPLKFVWWLPALPFMISIFIYDETRRFYLRRNPGGWLEQETYY
>Locus_1711_Transcript_8/11_Confidence_0.519_Length_4605|.|sp|
P13607|ATNA_DROME Sodium/potassium-transporting ATPase subunit alpha
OS=Drosophila melanogaster GN=Atpalpha PE=1 SV=3|e=0|from=1147|
to=4170|frame=-1|Fragment_1
KSRRKTPTKKQRKEGELDDLKQELDIDFHKVSPEELYQRFNTHPENGLSHAKAKENLERD
GPNALTPPKQTPEWVKFCKNLFGGFALLLWIGAILCFVAYSIQASTVEEPADDNLYLGIV
LAAVVIVTGIFSYYQESKSSKIMESFKNMVPQFATVLREGEKLTLRAEDLVLGDVVEVKF
GDRIPADIRIIESRGFKVDNSSLTGESEPQSRGPEFTNENPLETKNLAFFSTNAVEGTAK

GVVISCGDNTVMGRIAGLASGLDTGETPIAKEIHHFIHLITGVAVFLGVTFFVIAIILNY
HWLDAVIFLIGIIVANVPEG
>Locus_1711_Transcript_8/11_Confidence_0.519_Length_4605|.|sp|
P13607|ATNA_DROME Sodium/potassium-transporting ATPase subunit alpha
OS=Drosophila melanogaster GN=Atpalpha PE=1 SV=3|e=0|from=1147|
to=4170|frame=-1|Fragment_2
AKRMASKNCLVKNLEAVETLGSTSTICSDKTGTLTQNRMTVAHMWFDNQIIEADTTEDQS
GVVYDRTSPGFKALARIATLCNRAEFKGGQEGVPILKKEVSGDASEAALLKCMELALGDV
MSIRKRNKKVCEIPFNSTNKYQVSIHETEDASDPRHVMVMKGAPERILEKCSTIFIGGKE
KLLDEEMKEAFNNAY
>Locus_1711_Transcript_8/11_Confidence_0.519_Length_4605|.|sp|
P13607|ATNA_DROME Sodium/potassium-transporting ATPase subunit alpha
OS=Drosophila melanogaster GN=Atpalpha PE=1 SV=3|e=0|from=1147|
to=4170|frame=-1|Fragment_3
GFCDLMLPTDKFPLGFKFDSDDPNFPIENLRFVGLMSMI
>Locus_1711_Transcript_8/11_Confidence_0.519_Length_4605|.|sp|
P13607|ATNA_DROME Sodium/potassium-transporting ATPase subunit alpha
OS=Drosophila melanogaster GN=Atpalpha PE=1 SV=3|e=0|from=1147|
to=4170|frame=-1|Fragment_4
AKCRSAGIKVIMVTGDHPITAKAIAKSVGIISEGNETVEDIAQRLNIPVSEVNPREAKAA
VVHGTELRELNSDQLDEILRYHTEIVFARTSPQQKLIIVEGCQRMGAIVAVTGDGVNDSP
ALKKADIGVAMGIAGSDVSKQAADMILLDDNFASIVTGVEEGRLIFDNLKKSIAYTLTSN
IPEISPFLAFILCDIPLPLGTVTILCIDLGTDMVPAISLAYEHAEADIMKRPPRDPVNDK
LVNSRLISMAYGQIGMIQAAAGFFVYFVIMAENGFLPMKLFGIRKQWDSKAVNDLTDSYG
QEWTYRDRKTLEYTCHTAFFVSIVVVQWADLIVCKTRRNSLLHQGMRNWALNFGLVFETA
LAAFLSYTPGMDKGLRMFPLKFVWWLPALPFMISIFIYDETRRFYLRRNPGGWLEQETYY
>Locus_1711_Transcript_2/11_Confidence_0.222_Length_3271|.|sp|
P13607|ATNA_DROME Sodium/potassium-transporting ATPase subunit alpha
OS=Drosophila melanogaster GN=Atpalpha PE=1 SV=3|e=0|from=260|
to=2842|frame=-1|Fragment_1
DDKHGRSDSYRVATV
>Locus_1711_Transcript_2/11_Confidence_0.222_Length_3271|.|sp|
P13607|ATNA_DROME Sodium/potassium-transporting ATPase subunit alpha
OS=Drosophila melanogaster GN=Atpalpha PE=1 SV=3|e=0|from=260|
to=2842|frame=-1|Fragment_2
PTIDDNLTADGQYKSRRKTPTKKQRKEGELDDLKQELDIDFHKVSPEELYQRFNTHPENG
LSHAKAKENLERDGPNALTPPKQTPEWVKFCKNLFGGFALLLWIGAILCFVAYSIQASTV
EEPADDNLYLGIVLAAVVIVTGIFSYYQESKSSKIMESFKNMVPQFATVLREGEKLTLRA
EDLVLGDVVEVKFGDRIPADIRIIESRGFKVDNSSLTGESEPQSRGPEFTNENPLETKNL
AFFSTNAVEGTAKGVVISCGDNTVMGRIAGLASGLDTGETPIAKEIHHFIHLITGVAVFL
GVTFFVIAIILNYHWLDAVIFLIGIIVANVPEG
>Locus_1711_Transcript_2/11_Confidence_0.222_Length_3271|.|sp|
P13607|ATNA_DROME Sodium/potassium-transporting ATPase subunit alpha
OS=Drosophila melanogaster GN=Atpalpha PE=1 SV=3|e=0|from=260|
to=2842|frame=-1|Fragment_3
AKRMASKNCLVKNLEAVETLGSTSTICSDKTGTLTQNRMTVAHMWFDNQIIEADTTEDQS
GVVYDRTSPGFKALARIATLCNRAEFKGGQEGVPILKKEVSGDASEAALLKCMELALGDV
MSIRKRNKKVCEIPFNSTNKYQVSIHETEDASDPRHVMVMKGAPERILEKCSTIFIGGKE
KLLDEEMKEAFNNAY
>Locus_1711_Transcript_2/11_Confidence_0.222_Length_3271|.|sp|
P13607|ATNA_DROME Sodium/potassium-transporting ATPase subunit alpha
OS=Drosophila melanogaster GN=Atpalpha PE=1 SV=3|e=0|from=260|
to=2842|frame=-1|Fragment_4
GFCDLMLPTDKFPLGFKFDSDDPNFPIENLRFVGLMSMI

>Locus_1711_Transcript_2/11_Confidence_0.222_Length_3271|.|sp|
P13607|ATNA_DROME Sodium/potassium-transporting ATPase subunit alpha
OS=Drosophila melanogaster GN=Atpalpha PE=1 SV=3|e=0|from=260|
to=2842|frame=-1|Fragment_5
AKCRSAGIKVIMVTGDHPITAKAIAKSVGIISEGNETVEDIAQRLNIPVSEVNPREAKAA
VVHGTELRELNSDQLDEILRYHTEIVFARTSPQQKLIIVEGCQRMGAIVAVTGDGVNDSP
ALKKADIGVAMGIAGSDVSKQAADMILLDDNFASIVTGVEEGRLIFDNLKKSIAYTLTSN
IPEISPFLAFILCDIPLPLGTVTILCIDLGTDMVPAISLAYEESESDIMKRRPRNPYSDK
LVNER
>Locus_4333_Transcript_2/5_Confidence_0.615_Length_3375|UNIGENE
REPRESENTATIVE TRANSCRIPT|sp|P14599|A4_DROME Beta-amyloid-like
protein OS=Drosophila melanogaster GN=Appl PE=1 SV=2|e=7.9105E-130|
from=765|to=2234|frame=-2|Fragment_1
TSTQAPPSAQTP
>Locus_4333_Transcript_2/5_Confidence_0.615_Length_3375|UNIGENE
REPRESENTATIVE TRANSCRIPT|sp|P14599|A4_DROME Beta-amyloid-like
protein OS=Drosophila melanogaster GN=Appl PE=1 SV=2|e=7.9105E-130|
from=765|to=2234|frame=-2|Fragment_2
DPYFTHFDPRVEHQNYKEAQQRLEETHREKVTKVMKDWSDLEERYQDMRLADPRAAQGFK
RRMTARFQASVQALEEQGEAEKRQLAAMHQQRVLAHINQRRKEAMACYTQALTDQPPNTH
RVQKCLQKLLRALHKDR
>Locus_4333_Transcript_2/5_Confidence_0.615_Length_3375|UNIGENE
REPRESENTATIVE TRANSCRIPT|sp|P14599|A4_DROME Beta-amyloid-like
protein OS=Drosophila melanogaster GN=Appl PE=1 SV=2|e=7.9105E-130|
from=765|to=2234|frame=-2|Fragment_3
RPRTLERLVDIDRAVNQSMQMLRRYPELADKIGQLMDDYIQALRSKDETPGSMLAMTEDA
EIAILDKYRTEVERKLAEKERQRLFEKQRKEQRSRARAELRQEKMRSEAARHELTAKVAV
TEEQT
>Locus_4333_Transcript_2/5_Confidence_0.615_Length_3375|UNIGENE
REPRESENTATIVE TRANSCRIPT|sp|P14599|A4_DROME Beta-amyloid-like
protein OS=Drosophila melanogaster GN=Appl PE=1 SV=2|e=7.9105E-130|
from=765|to=2234|frame=-2|Fragment_4
STQRPLVAAQHNSGVVDDAAV
>Locus_4333_Transcript_2/5_Confidence_0.615_Length_3375|UNIGENE
REPRESENTATIVE TRANSCRIPT|sp|P14599|A4_DROME Beta-amyloid-like
protein OS=Drosophila melanogaster GN=Appl PE=1 SV=2|e=7.9105E-130|
from=765|to=2234|frame=-2|Fragment_5
QRAVEQVA
>Locus_4333_Transcript_2/5_Confidence_0.615_Length_3375|UNIGENE
REPRESENTATIVE TRANSCRIPT|sp|P14599|A4_DROME Beta-amyloid-like
protein OS=Drosophila melanogaster GN=Appl PE=1 SV=2|e=7.9105E-130|
from=765|to=2234|frame=-2|Fragment_6
LQRQTEARASHAMTHDLGHGEPGYSLRTHDLFTPSGRLGEGRSVYFT
>Locus_4333_Transcript_2/5_Confidence_0.615_Length_3375|UNIGENE
REPRESENTATIVE TRANSCRIPT|sp|P14599|A4_DROME Beta-amyloid-like
protein OS=Drosophila melanogaster GN=Appl PE=1 SV=2|e=7.9105E-130|
from=765|to=2234|frame=-2|Fragment_7
ARSPGAQGFVEVDQGCGTVPVPVTPEERHVANMQVNGYENPTYKYFEVKE
>Locus_4333_Transcript_3/5_Confidence_0.538_Length_3375|.|sp|P14599|
A4_DROME Beta-amyloid-like protein OS=Drosophila melanogaster
GN=Appl PE=1 SV=2|e=7.9105E-130|from=765|to=2234|frame=-2|Fragment_1
TSTQAPPSAQTP
>Locus_4333_Transcript_3/5_Confidence_0.538_Length_3375|.|sp|P14599|
A4_DROME Beta-amyloid-like protein OS=Drosophila melanogaster

GN=Appl PE=1 SV=2|e=7.9105E-130|from=765|to=2234|frame=-2|Fragment_2
DPYFTHFDPRVEHQNYKEAQQRLEETHREKVTKVMKDWSDLEERYQDMRLADPRAAQGFK
RRMTARFQASVQALEEQGEAEKRQLAAMHQQRVLAHINQRRKEAMACYTQALTDQPPNTH
RVQKCLQKLLRALHKDR
>Locus_4333_Transcript_3/5_Confidence_0.538_Length_3375|.|sp|P14599|
A4_DROME Beta-amyloid-like protein OS=Drosophila melanogaster
GN=Appl PE=1 SV=2|e=7.9105E-130|from=765|to=2234|frame=-2|Fragment_3
RPRTLERLVDIDRAVNQSMQMLRRYPELADKIGQLMDDYIQALRSKDETPGSMLAMTEDA
EIAILDKYRTEVERKLAEKERQRLFEKQRKEQRSRARAELRQEKMRSEAARHELTAKVAV
TEEQT
>Locus_4333_Transcript_3/5_Confidence_0.538_Length_3375|.|sp|P14599|
A4_DROME Beta-amyloid-like protein OS=Drosophila melanogaster
GN=Appl PE=1 SV=2|e=7.9105E-130|from=765|to=2234|frame=-2|Fragment_4
STQRPLVAAQHNSGVVDDAAV
>Locus_4333_Transcript_3/5_Confidence_0.538_Length_3375|.|sp|P14599|
A4_DROME Beta-amyloid-like protein OS=Drosophila melanogaster
GN=Appl PE=1 SV=2|e=7.9105E-130|from=765|to=2234|frame=-2|Fragment_5
QRAVEQVA
>Locus_4333_Transcript_3/5_Confidence_0.538_Length_3375|.|sp|P14599|
A4_DROME Beta-amyloid-like protein OS=Drosophila melanogaster
GN=Appl PE=1 SV=2|e=7.9105E-130|from=765|to=2234|frame=-2|Fragment_6
LQRQTEARASHAMTHDLGHGEPGYSLRTHDLFTPSGRLGEGRSVYFT
>Locus_4333_Transcript_3/5_Confidence_0.538_Length_3375|.|sp|P14599|
A4_DROME Beta-amyloid-like protein OS=Drosophila melanogaster
GN=Appl PE=1 SV=2|e=7.9105E-130|from=765|to=2234|frame=-2|Fragment_7
ARSPGAQGFVEVDQGCGTVPVPVTPEERHVANMQVNGYENPTYKYFEVKE
>Locus_4333_Transcript_5/5_Confidence_0.538_Length_3375|.|sp|P14599|
A4_DROME Beta-amyloid-like protein OS=Drosophila melanogaster
GN=Appl PE=1 SV=2|e=7.9105E-130|from=765|to=2234|frame=-2|Fragment_1
TSTQAPPSAQTP
>Locus_4333_Transcript_5/5_Confidence_0.538_Length_3375|.|sp|P14599|
A4_DROME Beta-amyloid-like protein OS=Drosophila melanogaster
GN=Appl PE=1 SV=2|e=7.9105E-130|from=765|to=2234|frame=-2|Fragment_2
DPYFTHFDPRVEHQNYKEAQQRLEETHREKVTKVMKDWSDLEERYQDMRLADPRAAQGFK
RRMTARFQASVQALEEQGEAEKRQLAAMHQQRVLAHINQRRKEAMACYTQALTDQPPNTH
RVQKCLQKLLRALHKDR
>Locus_4333_Transcript_5/5_Confidence_0.538_Length_3375|.|sp|P14599|
A4_DROME Beta-amyloid-like protein OS=Drosophila melanogaster
GN=Appl PE=1 SV=2|e=7.9105E-130|from=765|to=2234|frame=-2|Fragment_3
RPRTLERLVDIDRAVNQSMQMLRRYPELADKIGQLMDDYIQALRSKDETPGSMLAMTEDA
EIAILDKYRTEVERKLAEKERQRLFEKQRKEQRSRARAELRQEKMRSEAARHELTAKVAV
TEEQT
>Locus_4333_Transcript_5/5_Confidence_0.538_Length_3375|.|sp|P14599|
A4_DROME Beta-amyloid-like protein OS=Drosophila melanogaster
GN=Appl PE=1 SV=2|e=7.9105E-130|from=765|to=2234|frame=-2|Fragment_4
STQRPLVAAQHNSGVVDDAAV
>Locus_4333_Transcript_5/5_Confidence_0.538_Length_3375|.|sp|P14599|
A4_DROME Beta-amyloid-like protein OS=Drosophila melanogaster
GN=Appl PE=1 SV=2|e=7.9105E-130|from=765|to=2234|frame=-2|Fragment_5
QRAVEQVA
>Locus_4333_Transcript_5/5_Confidence_0.538_Length_3375|.|sp|P14599|
A4_DROME Beta-amyloid-like protein OS=Drosophila melanogaster
GN=Appl PE=1 SV=2|e=7.9105E-130|from=765|to=2234|frame=-2|Fragment_6
LQRQTEARASHAMTHDLGHGEPGYSLRTHDLFTPSGRLGEGRSVYFT

>Locus_4333_Transcript_5/5_Confidence_0.538_Length_3375|.|sp|P14599|
A4_DROME Beta-amyloid-like protein OS=Drosophila melanogaster
GN=Appl PE=1 SV=2|e=7.9105E-130|from=765|to=2234|frame=-2|Fragment_7
ARSPGAQGFVEVDQGCGTVPVPVTPEERHVANMQVNGYENPTYKYFEVKE
>Locus_4333_Transcript_4/5_Confidence_0.538_Length_3375|.|sp|P14599|
A4_DROME Beta-amyloid-like protein OS=Drosophila melanogaster
GN=Appl PE=1 SV=2|e=2.5447E-128|from=765|to=2204|frame=-2|Fragment_1
TPDPYFTHFDPRVEHQNYKEAQQRLEETHREKVTKVMKDWSDLEERYQDMRLADPRAAQG
FKRRMTARFQASVQALEEQGEAEKRQLAAMHQQRVLAHINQRRKEAMACYTQALTDQPPN
THRVQKCLQKLLRALHKDR
>Locus_4333_Transcript_4/5_Confidence_0.538_Length_3375|.|sp|P14599|
A4_DROME Beta-amyloid-like protein OS=Drosophila melanogaster
GN=Appl PE=1 SV=2|e=2.5447E-128|from=765|to=2204|frame=-2|Fragment_2
RPRTLERLVDIDRAVNQSMQMLRRYPELADKIGQLMDDYIQALRSKDETPGSMLAMTEDA
EIAILDKYRTEVERKLAEKERQRLFEKQRKEQRSRARAELRQEKMRSEAARHELTAKVAV
TEEQT
>Locus_4333_Transcript_4/5_Confidence_0.538_Length_3375|.|sp|P14599|
A4_DROME Beta-amyloid-like protein OS=Drosophila melanogaster
GN=Appl PE=1 SV=2|e=2.5447E-128|from=765|to=2204|frame=-2|Fragment_3
STQRPLVAAQHNSGVVDDAAV
>Locus_4333_Transcript_4/5_Confidence_0.538_Length_3375|.|sp|P14599|
A4_DROME Beta-amyloid-like protein OS=Drosophila melanogaster
GN=Appl PE=1 SV=2|e=2.5447E-128|from=765|to=2204|frame=-2|Fragment_4
QRAVEQVA
>Locus_4333_Transcript_4/5_Confidence_0.538_Length_3375|.|sp|P14599|
A4_DROME Beta-amyloid-like protein OS=Drosophila melanogaster
GN=Appl PE=1 SV=2|e=2.5447E-128|from=765|to=2204|frame=-2|Fragment_5
LQRQTEARASHAMTHDLGHGEPGYSLRTHDLFTPSGRLGEGRSVYFT
>Locus_4333_Transcript_4/5_Confidence_0.538_Length_3375|.|sp|P14599|
A4_DROME Beta-amyloid-like protein OS=Drosophila melanogaster
GN=Appl PE=1 SV=2|e=2.5447E-128|from=765|to=2204|frame=-2|Fragment_6
ARSPGAQGFVEVDQGCGTVPVPVTPEERHVANMQVNGYENPTYKYFEVKE
>Locus_4333_Transcript_2/5_Confidence_0.615_Length_3375|.|sp|P14599|
A4_DROME Beta-amyloid-like protein OS=Drosophila melanogaster
GN=Appl PE=1 SV=2|e=5.19928E-81|from=2574|to=3131|frame=-2|
Fragment_1
RWEPQVAVLCDPGQGYHPQHLADDGRWTADLTAKPPRGSACLRDKMDILEHCKKVYPNRD
ITNIVESSHYLKVSGWCRLGATNQAKCKAA
>Locus_4333_Transcript_2/5_Confidence_0.615_Length_3375|.|sp|P14599|
A4_DROME Beta-amyloid-like protein OS=Drosophila melanogaster
GN=Appl PE=1 SV=2|e=5.19928E-81|from=2574|to=3131|frame=-2|
Fragment_2
RWVKPFRCLEGPFQSDALLVPEGCLFDHIHNQSHCWQFSRWNQTGARACSERGLQLRSFA
MLLPCGISLFSGVEFVCCPKHYK
>Locus_4333_Transcript_2/5_Confidence_0.615_Length_3375|.|sp|P14599|
A4_DROME Beta-amyloid-like protein OS=Drosophila melanogaster
GN=Appl PE=1 SV=2|e=5.19928E-81|from=2574|to=3131|frame=-2|
Fragment_3
DNSRVKKIDLPAM
>Locus_4333_Transcript_3/5_Confidence_0.538_Length_3375|.|sp|P14599|
A4_DROME Beta-amyloid-like protein OS=Drosophila melanogaster
GN=Appl PE=1 SV=2|e=5.19928E-81|from=2574|to=3131|frame=-2|
Fragment_1
RWEPQVAVLCDPGQGYHPQHLADDGRWTADLTAKPPRGSACLRDKMDILEHCKKVYPNRD

ITNIVESSHYLKVSGWCRLGATNQAKCKAA
>Locus_4333_Transcript_3/5_Confidence_0.538_Length_3375|.|sp|P14599|
A4_DROME Beta-amyloid-like protein OS=Drosophila melanogaster
GN=Appl PE=1 SV=2|e=5.19928E-81|from=2574|to=3131|frame=-2|
Fragment_2
RWVKPFRCLEGPFQSDALLVPEGCLFDHIHNQSHCWQFSRWNQTGARACSERGLQLRSFA
MLLPCGISLFSGVEFVCCPKHYK
>Locus_4333_Transcript_3/5_Confidence_0.538_Length_3375|.|sp|P14599|
A4_DROME Beta-amyloid-like protein OS=Drosophila melanogaster
GN=Appl PE=1 SV=2|e=5.19928E-81|from=2574|to=3131|frame=-2|
Fragment_3
DNSRVKKIDLPAM
>Locus_4333_Transcript_4/5_Confidence_0.538_Length_3375|.|sp|P14599|
A4_DROME Beta-amyloid-like protein OS=Drosophila melanogaster
GN=Appl PE=1 SV=2|e=5.19928E-81|from=2574|to=3131|frame=-2|
Fragment_1
RWEPQVAVLCDPGQGYHPQHLADDGRWTADLTAKPPRGSACLRDKMDILEHCKKVYPNRD
ITNIVESSHYLKVSGWCRLGATNQAKCKAA
>Locus_4333_Transcript_4/5_Confidence_0.538_Length_3375|.|sp|P14599|
A4_DROME Beta-amyloid-like protein OS=Drosophila melanogaster
GN=Appl PE=1 SV=2|e=5.19928E-81|from=2574|to=3131|frame=-2|
Fragment_2
RWVKPFRCLEGPFQSDALLVPEGCLFDHIHNQSHCWQFSRWNQTGARACSERGLQLRSFA
MLLPCGISLFSGVEFVCCPKHYK
>Locus_4333_Transcript_4/5_Confidence_0.538_Length_3375|.|sp|P14599|
A4_DROME Beta-amyloid-like protein OS=Drosophila melanogaster
GN=Appl PE=1 SV=2|e=5.19928E-81|from=2574|to=3131|frame=-2|
Fragment_3
DNSRVKKIDLPAM
>Locus_4333_Transcript_5/5_Confidence_0.538_Length_3375|.|sp|P14599|
A4_DROME Beta-amyloid-like protein OS=Drosophila melanogaster
GN=Appl PE=1 SV=2|e=5.19928E-81|from=2574|to=3131|frame=-2|
Fragment_1
RWEPQVAVLCDPGQGYHPQHLADDGRWTADLTAKPPRGSACLRDKMDILEHCKKVYPNRD
ITNIVESSHYLKVSGWCRLGATNQAKCKAA
>Locus_4333_Transcript_5/5_Confidence_0.538_Length_3375|.|sp|P14599|
A4_DROME Beta-amyloid-like protein OS=Drosophila melanogaster
GN=Appl PE=1 SV=2|e=5.19928E-81|from=2574|to=3131|frame=-2|
Fragment_2
RWVKPFRCLEGPFQSDALLVPEGCLFDHIHNQSHCWQFSRWNQTGARACSERGLQLRSFA
MLLPCGISLFSGVEFVCCPKHYK
>Locus_4333_Transcript_5/5_Confidence_0.538_Length_3375|.|sp|P14599|
A4_DROME Beta-amyloid-like protein OS=Drosophila melanogaster
GN=Appl PE=1 SV=2|e=5.19928E-81|from=2574|to=3131|frame=-2|
Fragment_3
DNSRVKKIDLPAM
>Locus_4333_Transcript_1/5_Confidence_0.538_Length_2663|.|sp|P14599|
A4_DROME Beta-amyloid-like protein OS=Drosophila melanogaster
GN=Appl PE=1 SV=2|e=6.0976E-130|from=765|to=2234|frame=-1|Fragment_1
TSTQAPPSAQTP
>Locus_4333_Transcript_1/5_Confidence_0.538_Length_2663|.|sp|P14599|
A4_DROME Beta-amyloid-like protein OS=Drosophila melanogaster
GN=Appl PE=1 SV=2|e=6.0976E-130|from=765|to=2234|frame=-1|Fragment_2
DPYFTHFDPRVEHQNYKEAQQRLEETHREKVTKVMKDWSDLEERYQDMRLADPRAAQGFK

RRMTARFQASVQALEEQGEAEKRQLAAMHQQRVLAHINQRRKEAMACYTQALTDQPPNTH
RVQKCLQKLLRALHKDR
>Locus_4333_Transcript_1/5_Confidence_0.538_Length_2663|.|sp|P14599|
A4_DROME Beta-amyloid-like protein OS=Drosophila melanogaster
GN=Appl PE=1 SV=2|e=6.0976E-130|from=765|to=2234|frame=-1|Fragment_3
RPRTLERLVDIDRAVNQSMQMLRRYPELADKIGQLMDDYIQALRSKDETPGSMLAMTEDA
EIAILDKYRTEVERKLAEKERQRLFEKQRKEQRSRARAELRQEKMRSEAARHELTAKVAV
TEEQT
>Locus_4333_Transcript_1/5_Confidence_0.538_Length_2663|.|sp|P14599|
A4_DROME Beta-amyloid-like protein OS=Drosophila melanogaster
GN=Appl PE=1 SV=2|e=6.0976E-130|from=765|to=2234|frame=-1|Fragment_4
STQRPLVAAQHNSGVVDDAAV
>Locus_4333_Transcript_1/5_Confidence_0.538_Length_2663|.|sp|P14599|
A4_DROME Beta-amyloid-like protein OS=Drosophila melanogaster
GN=Appl PE=1 SV=2|e=6.0976E-130|from=765|to=2234|frame=-1|Fragment_5
QRAVEQVA
>Locus_4333_Transcript_1/5_Confidence_0.538_Length_2663|.|sp|P14599|
A4_DROME Beta-amyloid-like protein OS=Drosophila melanogaster
GN=Appl PE=1 SV=2|e=6.0976E-130|from=765|to=2234|frame=-1|Fragment_6
LQRQTEARASHAMTHDLGHGEPGYSLRTHDLFTPSGRLGEGRSVYFT
>Locus_4333_Transcript_1/5_Confidence_0.538_Length_2663|.|sp|P14599|
A4_DROME Beta-amyloid-like protein OS=Drosophila melanogaster
GN=Appl PE=1 SV=2|e=6.0976E-130|from=765|to=2234|frame=-1|Fragment_7
ARSPGAQGFVEVDQGCGTVPVPVTPEERHVANMQVNGYENPTYKYFEVKE

>Locus_6862_Transcript_1/3_Confidence_0.714_Length_3370|UNIGENE
REPRESENTATIVE TRANSCRIPT|sp|P14773|HMDH_DROME 3-hydroxy-3-
methylglutaryl-coenzyme A reductase OS=Drosophila melanogaster
GN=Hmgcr PE=2 SV=2|e=0|from=496|to=3111|frame=-2|Fragment_1
MVGRLFRAHGEFCASHPWEVIV
>Locus_6862_Transcript_1/3_Confidence_0.714_Length_3370|UNIGENE
REPRESENTATIVE TRANSCRIPT|sp|P14773|HMDH_DROME 3-hydroxy-3-
methylglutaryl-coenzyme A reductase OS=Drosophila melanogaster
GN=Hmgcr PE=2 SV=2|e=0|from=496|to=3111|frame=-2|Fragment_2
LDRG
>Locus_6862_Transcript_1/3_Confidence_0.714_Length_3370|UNIGENE
REPRESENTATIVE TRANSCRIPT|sp|P14773|HMDH_DROME 3-hydroxy-3-
methylglutaryl-coenzyme A reductase OS=Drosophila melanogaster
GN=Hmgcr PE=2 SV=2|e=0|from=496|to=3111|frame=-2|Fragment_3
VPAGPGTVSPLARHCG
>Locus_6862_Transcript_1/3_Confidence_0.714_Length_3370|UNIGENE
REPRESENTATIVE TRANSCRIPT|sp|P14773|HMDH_DROME 3-hydroxy-3-
methylglutaryl-coenzyme A reductase OS=Drosophila melanogaster
GN=Hmgcr PE=2 SV=2|e=0|from=496|to=3111|frame=-2|Fragment_4
AWRGGSCPVP
>Locus_6862_Transcript_1/3_Confidence_0.714_Length_3370|UNIGENE
REPRESENTATIVE TRANSCRIPT|sp|P14773|HMDH_DROME 3-hydroxy-3-
methylglutaryl-coenzyme A reductase OS=Drosophila melanogaster
GN=Hmgcr PE=2 SV=2|e=0|from=496|to=3111|frame=-2|Fragment_5
GGPQQTAT
>Locus_6862_Transcript_1/3_Confidence_0.714_Length_3370|UNIGENE
REPRESENTATIVE TRANSCRIPT|sp|P14773|HMDH_DROME 3-hydroxy-3-
methylglutaryl-coenzyme A reductase OS=Drosophila melanogaster
GN=Hmgcr PE=2 SV=2|e=0|from=496|to=3111|frame=-2|Fragment_6

DAEYNAADVILMTIIRCSAVLYSYYQFCNLHKLGSKYILAIAGLFT
>Locus_6862_Transcript_1/3_Confidence_0.714_Length_3370|UNIGENE
REPRESENTATIVE TRANSCRIPT|sp|P14773|HMDH_DROME 3-hydroxy-3-
methylglutaryl-coenzyme A reductase OS=Drosophila melanogaster
GN=Hmgcr PE=2 SV=2|e=0|from=496|to=3111|frame=-2|Fragment_7
VINFLRSDIS
>Locus_6862_Transcript_1/3_Confidence_0.714_Length_3370|UNIGENE
REPRESENTATIVE TRANSCRIPT|sp|P14773|HMDH_DROME 3-hydroxy-3-
methylglutaryl-coenzyme A reductase OS=Drosophila melanogaster
GN=Hmgcr PE=2 SV=2|e=0|from=496|to=3111|frame=-2|Fragment_8
AEVTQNIARGMSLLGPTITLDTLVEALVIGVGTLSGVPRLEVLCCFACLSVLVNYVVFMT
FYPACLSLILELSRTGEGSNLGRGEA
>Locus_6862_Transcript_1/3_Confidence_0.714_Length_3370|UNIGENE
REPRESENTATIVE TRANSCRIPT|sp|P14773|HMDH_DROME 3-hydroxy-3-
methylglutaryl-coenzyme A reductase OS=Drosophila melanogaster
GN=Hmgcr PE=2 SV=2|e=0|from=496|to=3111|frame=-2|Fragment_9
LLAKALHEEDQKPNPVVQRVKVIMSVGLMIVHAHSRWSLQDGGDHDGPAAPLVGSEQHRT
MTQNRTEETSLPGHLMRWMAVGAEQ
>Locus_6862_Transcript_1/3_Confidence_0.714_Length_3370|UNIGENE
REPRESENTATIVE TRANSCRIPT|sp|P14773|HMDH_DROME 3-hydroxy-3-
methylglutaryl-coenzyme A reductase OS=Drosophila melanogaster
GN=Hmgcr PE=2 SV=2|e=0|from=496|to=3111|frame=-2|Fragment_10
VKFIFFEDNQQQLAGQLRMSESENTDNSNNATTMRDRERERP
>Locus_6862_Transcript_1/3_Confidence_0.714_Length_3370|UNIGENE
REPRESENTATIVE TRANSCRIPT|sp|P14773|HMDH_DROME 3-hydroxy-3-
methylglutaryl-coenzyme A reductase OS=Drosophila melanogaster
GN=Hmgcr PE=2 SV=2|e=0|from=496|to=3111|frame=-2|Fragment_11
IFGIGDEATEDKATQTVTMIEESQSNEVEVEPEPRSLDVCLEIYRSER
>Locus_6862_Transcript_1/3_Confidence_0.714_Length_3370|UNIGENE
REPRESENTATIVE TRANSCRIPT|sp|P14773|HMDH_DROME 3-hydroxy-3-
methylglutaryl-coenzyme A reductase OS=Drosophila melanogaster
GN=Hmgcr PE=2 SV=2|e=0|from=496|to=3111|frame=-2|Fragment_12
GAAALTDAEVALLVRA
>Locus_6862_Transcript_1/3_Confidence_0.714_Length_3370|UNIGENE
REPRESENTATIVE TRANSCRIPT|sp|P14773|HMDH_DROME 3-hydroxy-3-
methylglutaryl-coenzyme A reductase OS=Drosophila melanogaster
GN=Hmgcr PE=2 SV=2|e=0|from=496|to=3111|frame=-2|Fragment_13
RHIPLYRIEAAVGDDERGVKLRRRILTSEGLFSDTALRDLPYLDYDYSRVMNACCENVLG
YVQVPVGFAGPLLLDGRKYYVPMATTEGALVASTNRGCKALLARGVNSVVEDVGMTRAPC
IRFPTVRRADCAKNWLERRANFEAVKASFDSTSRFARLQEIYVGMDGPQLYVRFRATTGD
AMGMNMVSKGAEMALRHVQKVFPDMEIVSLSGNYCSDKKPAAINWIKGRGKRVVCDAVIP
GTVLSSVFRTNAATLVRCNKLKNMVGTSLAGSIGGNNAHAANMVTAIFIATGQDPAQNVT
SSNCSTGMEVCGDNNEDLYMTCTMPSLEVGTIGGGTVLPGQGACLEMLGVKGAHPAQPAE
NAKSLARIICATVMAGELSLLAALVNSDLVRSHMRHNRSSVAVNPGLNNSESSTVS
>Locus_6862_Transcript_2/3_Confidence_0.714_Length_3370|.|sp|P14773|
HMDH_DROME 3-hydroxy-3-methylglutaryl-coenzyme A reductase
OS=Drosophila melanogaster GN=Hmgcr PE=2 SV=2|e=0|from=496|to=3111|
frame=-2|Fragment_1
MVGRLFRAHGEFCASHPWEVIV
>Locus_6862_Transcript_2/3_Confidence_0.714_Length_3370|.|sp|P14773|
HMDH_DROME 3-hydroxy-3-methylglutaryl-coenzyme A reductase
OS=Drosophila melanogaster GN=Hmgcr PE=2 SV=2|e=0|from=496|to=3111|
frame=-2|Fragment_2
LDRG

>Locus_6862_Transcript_2/3_Confidence_0.714_Length_3370|.|sp|P14773|
HMDH_DROME 3-hydroxy-3-methylglutaryl-coenzyme A reductase
OS=Drosophila melanogaster GN=Hmgcr PE=2 SV=2|e=0|from=496|to=3111|
frame=-2|Fragment_3
VPAGPGTVSPLARHCG
>Locus_6862_Transcript_2/3_Confidence_0.714_Length_3370|.|sp|P14773|
HMDH_DROME 3-hydroxy-3-methylglutaryl-coenzyme A reductase
OS=Drosophila melanogaster GN=Hmgcr PE=2 SV=2|e=0|from=496|to=3111|
frame=-2|Fragment_4
AWRGGSCPVP
>Locus_6862_Transcript_2/3_Confidence_0.714_Length_3370|.|sp|P14773|
HMDH_DROME 3-hydroxy-3-methylglutaryl-coenzyme A reductase
OS=Drosophila melanogaster GN=Hmgcr PE=2 SV=2|e=0|from=496|to=3111|
frame=-2|Fragment_5
GGPQQTAT
>Locus_6862_Transcript_2/3_Confidence_0.714_Length_3370|.|sp|P14773|
HMDH_DROME 3-hydroxy-3-methylglutaryl-coenzyme A reductase
OS=Drosophila melanogaster GN=Hmgcr PE=2 SV=2|e=0|from=496|to=3111|
frame=-2|Fragment_6
DAEYNAADVILMTIIRCSAVLYSYYQFCNLHKLGSKYILAIAGLFT
>Locus_6862_Transcript_2/3_Confidence_0.714_Length_3370|.|sp|P14773|
HMDH_DROME 3-hydroxy-3-methylglutaryl-coenzyme A reductase
OS=Drosophila melanogaster GN=Hmgcr PE=2 SV=2|e=0|from=496|to=3111|
frame=-2|Fragment_7
VINFLRSDIS
>Locus_6862_Transcript_2/3_Confidence_0.714_Length_3370|.|sp|P14773|
HMDH_DROME 3-hydroxy-3-methylglutaryl-coenzyme A reductase
OS=Drosophila melanogaster GN=Hmgcr PE=2 SV=2|e=0|from=496|to=3111|
frame=-2|Fragment_8
AEVTQNIARGMSLLGPTITLDTLVEALVIGVGTLSGVPRLEVLCCFACLSVLVNYVVFMT
FYPACLSLILELSRTGEGSNLGRGEA
>Locus_6862_Transcript_2/3_Confidence_0.714_Length_3370|.|sp|P14773|
HMDH_DROME 3-hydroxy-3-methylglutaryl-coenzyme A reductase
OS=Drosophila melanogaster GN=Hmgcr PE=2 SV=2|e=0|from=496|to=3111|
frame=-2|Fragment_9
LLAKALHEEDQKPNPVVQRVKVIMSVGLMIVHAHSRWSLQDGGDHDGPAAPLVGSEQHRT
MTQNRTEETSLPGHLMRWMAVGAEQ
>Locus_6862_Transcript_2/3_Confidence_0.714_Length_3370|.|sp|P14773|
HMDH_DROME 3-hydroxy-3-methylglutaryl-coenzyme A reductase
OS=Drosophila melanogaster GN=Hmgcr PE=2 SV=2|e=0|from=496|to=3111|
frame=-2|Fragment_10
VKFIFFEDNQQQLAGQLRMSESENTDNSNNATTMRDRERERP
>Locus_6862_Transcript_2/3_Confidence_0.714_Length_3370|.|sp|P14773|
HMDH_DROME 3-hydroxy-3-methylglutaryl-coenzyme A reductase
OS=Drosophila melanogaster GN=Hmgcr PE=2 SV=2|e=0|from=496|to=3111|
frame=-2|Fragment_11
IFGIGDEATEDKATQTVTMIEESQSNEVEVEPEPRSLDVCLEIYRSER
>Locus_6862_Transcript_2/3_Confidence_0.714_Length_3370|.|sp|P14773|
HMDH_DROME 3-hydroxy-3-methylglutaryl-coenzyme A reductase
OS=Drosophila melanogaster GN=Hmgcr PE=2 SV=2|e=0|from=496|to=3111|
frame=-2|Fragment_12
GAAALTDAEVALLVRA
>Locus_6862_Transcript_2/3_Confidence_0.714_Length_3370|.|sp|P14773|
HMDH_DROME 3-hydroxy-3-methylglutaryl-coenzyme A reductase

OS=Drosophila melanogaster GN=Hmgcr PE=2 SV=2|e=0|from=496|to=3111|
frame=-2|Fragment_13
RHIPLYRIEAAVGDDERGVKLRRRILTSEGLFSDTALRDLPYLDYDYSRVMNACCENVLG
YVQVPVGFAGPLLLDGRKYYVPMATTEGALVASTNRGCKALLARGVNSVVEDVGMTRAPC
IRFPTVRRADCAKNWLERRANFEAVKASFDSTSRFARLQEIYVGMDGPQLYVRFRATTGD
AMGMNMVSKGAEMALRHVQKVFPDMEIVSLSGNYCSDKKPAAINWIKGRGKRVVCDAVIP
GTVLSSVFRTNAATLVRCNKLKNMVGTSLAGSIGGNNAHAANMVTAIFIATGQDPAQNVT
SSNCSTGMEVCGDNNEDLYMTCTMPSLEVGTIGGGTVLPGQGACLEMLGVKGAHPAQPAE
NAKSLARIICATVMAGELSLLAALVNSDLVRSHMRHNRSSVAVNPGLNNSESSTVS
>Locus_6862_Transcript_3/3_Confidence_0.714_Length_3370|.|sp|P14773|
HMDH_DROME 3-hydroxy-3-methylglutaryl-coenzyme A reductase
OS=Drosophila melanogaster GN=Hmgcr PE=2 SV=2|e=0|from=496|to=3111|
frame=-2|Fragment_1
MVGRLFRAHGEFCASHPWEVIV
>Locus_6862_Transcript_3/3_Confidence_0.714_Length_3370|.|sp|P14773|
HMDH_DROME 3-hydroxy-3-methylglutaryl-coenzyme A reductase
OS=Drosophila melanogaster GN=Hmgcr PE=2 SV=2|e=0|from=496|to=3111|
frame=-2|Fragment_2
LDRG
>Locus_6862_Transcript_3/3_Confidence_0.714_Length_3370|.|sp|P14773|
HMDH_DROME 3-hydroxy-3-methylglutaryl-coenzyme A reductase
OS=Drosophila melanogaster GN=Hmgcr PE=2 SV=2|e=0|from=496|to=3111|
frame=-2|Fragment_3
VPAGPGTVSPLARHCG
>Locus_6862_Transcript_3/3_Confidence_0.714_Length_3370|.|sp|P14773|
HMDH_DROME 3-hydroxy-3-methylglutaryl-coenzyme A reductase
OS=Drosophila melanogaster GN=Hmgcr PE=2 SV=2|e=0|from=496|to=3111|
frame=-2|Fragment_4
AWRGGSCPVP
>Locus_6862_Transcript_3/3_Confidence_0.714_Length_3370|.|sp|P14773|
HMDH_DROME 3-hydroxy-3-methylglutaryl-coenzyme A reductase
OS=Drosophila melanogaster GN=Hmgcr PE=2 SV=2|e=0|from=496|to=3111|
frame=-2|Fragment_5
GGPQQTAT
>Locus_6862_Transcript_3/3_Confidence_0.714_Length_3370|.|sp|P14773|
HMDH_DROME 3-hydroxy-3-methylglutaryl-coenzyme A reductase
OS=Drosophila melanogaster GN=Hmgcr PE=2 SV=2|e=0|from=496|to=3111|
frame=-2|Fragment_6
DAEYNAADVILMTIIRCSAVLYSYYQFCNLHKLGSKYILAIAGLFT
>Locus_6862_Transcript_3/3_Confidence_0.714_Length_3370|.|sp|P14773|
HMDH_DROME 3-hydroxy-3-methylglutaryl-coenzyme A reductase
OS=Drosophila melanogaster GN=Hmgcr PE=2 SV=2|e=0|from=496|to=3111|
frame=-2|Fragment_7
VINFLRSDIS
>Locus_6862_Transcript_3/3_Confidence_0.714_Length_3370|.|sp|P14773|
HMDH_DROME 3-hydroxy-3-methylglutaryl-coenzyme A reductase
OS=Drosophila melanogaster GN=Hmgcr PE=2 SV=2|e=0|from=496|to=3111|
frame=-2|Fragment_8
AEVTQNIARGMSLLGPTITLDTLVEALVIGVGTLSGVPRLEVLCCFACLSVLVNYVVFMT
FYPACLSLILELSRTGEGSNLGRGEA
>Locus_6862_Transcript_3/3_Confidence_0.714_Length_3370|.|sp|P14773|
HMDH_DROME 3-hydroxy-3-methylglutaryl-coenzyme A reductase
OS=Drosophila melanogaster GN=Hmgcr PE=2 SV=2|e=0|from=496|to=3111|
frame=-2|Fragment_9

LLAKALHEEDQKPNPVVQRVKVIMSVGLMIVHAHSRWSLQDGGDHDGPAAPLVGSEQHRT
MTQNRTEETSLPGHLMRWMAVGAEQ
>Locus_6862_Transcript_3/3_Confidence_0.714_Length_3370|.|sp|P14773|
HMDH_DROME 3-hydroxy-3-methylglutaryl-coenzyme A reductase
OS=Drosophila melanogaster GN=Hmgcr PE=2 SV=2|e=0|from=496|to=3111|
frame=-2|Fragment_10
VKFIFFEDNQQQLAGQLRMSESENTDNSNNATTMRDRERERP
>Locus_6862_Transcript_3/3_Confidence_0.714_Length_3370|.|sp|P14773|
HMDH_DROME 3-hydroxy-3-methylglutaryl-coenzyme A reductase
OS=Drosophila melanogaster GN=Hmgcr PE=2 SV=2|e=0|from=496|to=3111|
frame=-2|Fragment_11
IFGIGDEATEDKATQTVTMIEESQSNEVEVEPEPRSLDVCLEIYRSER
>Locus_6862_Transcript_3/3_Confidence_0.714_Length_3370|.|sp|P14773|
HMDH_DROME 3-hydroxy-3-methylglutaryl-coenzyme A reductase
OS=Drosophila melanogaster GN=Hmgcr PE=2 SV=2|e=0|from=496|to=3111|
frame=-2|Fragment_12
GAAALTDAEVALLVRA
>Locus_6862_Transcript_3/3_Confidence_0.714_Length_3370|.|sp|P14773|
HMDH_DROME 3-hydroxy-3-methylglutaryl-coenzyme A reductase
OS=Drosophila melanogaster GN=Hmgcr PE=2 SV=2|e=0|from=496|to=3111|
frame=-2|Fragment_13
RHIPLYRIEAAVGDDERGVKLRRRILTSEGLFSDTALRDLPYLDYDYSRVMNACCENVLG
YVQVPVGFAGPLLLDGRKYYVPMATTEGALVASTNRGCKALLARGVNSVVEDVGMTRAPC
IRFPTVRRADCAKNWLERRANFEAVKASFDSTSRFARLQEIYVGMDGPQLYVRFRATTGD
AMGMNMVSKGAEMALRHVQKVFPDMEIVSLSGNYCSDKKPAAINWIKGRGKRVVCDAVIP
GTVLSSVFRTNAATLVRCNKLKNMVGTSLAGSIGGNNAHAANMVTAIFIATGQDPAQNVT
SSNCSTGMEVCGDNNEDLYMTCTMPSLEVGTIGGGTVLPGQGACLEMLGVKGAHPAQPAE
NAKSLARIICATVMAGELSLLAALVNSDLVRSHMRHNRSSVAVNPGLNNSESSTVS

>Locus_17319_Transcript_1/1_Confidence_1.000_Length_590|UNIGENE
REPRESENTATIVE TRANSCRIPT|sp|P16395|ACM1_DROME Muscarinic
acetylcholine receptor DM1 OS=Drosophila melanogaster GN=mAcR-60C
PE=2 SV=2|e=3.61859E-48|from=3|to=545|frame=3|Fragment_1
ERTVPNTECYIQFIETNHYITFGTAIAAFYVPVTVMCILYFKIWRETKKRQKDLPNLQAG
>Locus_17319_Transcript_1/1_Confidence_1.000_Length_590|UNIGENE
REPRESENTATIVE TRANSCRIPT|sp|P16395|ACM1_DROME Muscarinic
acetylcholine receptor DM1 OS=Drosophila melanogaster GN=mAcR-60C
PE=2 SV=2|e=3.61859E-48|from=3|to=545|frame=3|Fragment_2
EATHVNHTG
>Locus_17319_Transcript_1/1_Confidence_1.000_Length_590|UNIGENE
REPRESENTATIVE TRANSCRIPT|sp|P16395|ACM1_DROME Muscarinic
acetylcholine receptor DM1 OS=Drosophila melanogaster GN=mAcR-60C
PE=2 SV=2|e=3.61859E-48|from=3|to=545|frame=3|Fragment_3
EWARPRSESAGDPDD
>Locus_17319_Transcript_1/1_Confidence_1.000_Length_590|UNIGENE
REPRESENTATIVE TRANSCRIPT|sp|P16395|ACM1_DROME Muscarinic
acetylcholine receptor DM1 OS=Drosophila melanogaster GN=mAcR-60C
PE=2 SV=2|e=3.61859E-48|from=3|to=545|frame=3|Fragment_4
GCGNTTDSG
>Locus_17319_Transcript_1/1_Confidence_1.000_Length_590|UNIGENE
REPRESENTATIVE TRANSCRIPT|sp|P16395|ACM1_DROME Muscarinic
acetylcholine receptor DM1 OS=Drosophila melanogaster GN=mAcR-60C
PE=2 SV=2|e=3.61859E-48|from=3|to=545|frame=3|Fragment_5
GTYVKRTGL

>Locus_17319_Transcript_1/1_Confidence_1.000_Length_590|UNIGENE REPRESENTATIVE TRANSCRIPT|sp|P16395|ACM1_DROME Muscarinic acetylcholine receptor DM1 OS=Drosophila melanogaster GN=mAcR-60C PE=2 SV=2|e=3.61859E-48|from=3|to=545|frame=3|Fragment_6
ALFLWYASCGGVREWCVAWWHSGR
>Locus_17319_Transcript_1/1_Confidence_1.000_Length_590|UNIGENE REPRESENTATIVE TRANSCRIPT|sp|P16395|ACM1_DROME Muscarinic acetylcholine receptor DM1 OS=Drosophila melanogaster GN=mAcR-60C PE=2 SV=2|e=3.61859E-48|from=3|to=545|frame=3|Fragment_7
GYATPVSIETPLQSSVSRCTSLNVIRDPY

>Locus_18519_Transcript_1/2_Confidence_0.667_Length_996|UNIGENE REPRESENTATIVE TRANSCRIPT|sp|P16621|LAR_DROME Tyrosine-protein phosphatase Lar OS=Drosophila melanogaster GN=Lar PE=1 SV=2|e=2.8018E-123|from=1|to=996|frame=1|Fragment_1
RGAGPYCADVSARTERDLGRAPMSVQAVATSDSSAEVWWEGVPSRGKVVGYRVFY
>Locus_18519_Transcript_1/2_Confidence_0.667_Length_996|UNIGENE REPRESENTATIVE TRANSCRIPT|sp|P16621|LAR_DROME Tyrosine-protein phosphatase Lar OS=Drosophila melanogaster GN=Lar PE=1 SV=2|e=2.8018E-123|from=1|to=996|frame=1|Fragment_2
LDEWQHKDVARTESADLVNLEKYATYRVAVAARTSDGGLWRLSEKVNVKVKPEDVPLDLR
AHDVSTHSMTLSWSPPIRLNPVNYKVSFDAVKEFVDSQGFTRSQTVPRREVVLPAHAKNH
MIDELSPFTTYCVNVSAVPNDYQYRPPARITVTTQMAAPQPMVKPDFYGVVNGEEIQVIL
PQASEEYGPISHYYLIVVPEDESLAHKHPDQFLTDEMIAGAKMVNNNGAPGTSASMAKAS
LLDAPYIAAKFPQRNIPYTFHLG
>Locus_18519_Transcript_2/2_Confidence_0.667_Length_672|.|sp|P16621|LAR_DROME Tyrosine-protein phosphatase Lar OS=Drosophila melanogaster GN=Lar PE=1 SV=2|e=9.83485E-91|from=4|to=672|frame=1|Fragment_1
RLSEKVNVKVKPEDVPLDLRAHDVSTHSMTLSWSPPIRLNPVNYKVSFDAVKEFVDSQGF
TRSQTVPRREVVLPAHAKNHMIDELSPFTTYCVNVSAVPNDYQYRPPARITVTTQMAAPQ
PMVKPDFYGVVNGEEIQVILPQASEEYGPISHYYLIVVPEDESLAHKHPDQFLTDEMIAG
AKMVNNNGAPGTSASMAKASLLDAPYIAAKFPQRNIPYTFHLG
>Locus_16047_Transcript_3/5_Confidence_0.200_Length_437|.|sp|P16621|LAR_DROME Tyrosine-protein phosphatase Lar OS=Drosophila melanogaster GN=Lar PE=1 SV=2|e=2.38114E-40|from=2|to=436|frame=2|Fragment_1
GDIYDGFTNRRLERDRRYRIFVRAVVDTPQKHLYTSSPFSEYLSLSMREV
>Locus_16047_Transcript_3/5_Confidence_0.200_Length_437|.|sp|P16621|LAR_DROME Tyrosine-protein phosphatase Lar OS=Drosophila melanogaster GN=Lar PE=1 SV=2|e=2.38114E-40|from=2|to=436|frame=2|Fragment_2
VSVNRNTEEAGLVWIVGP
>Locus_16047_Transcript_3/5_Confidence_0.200_Length_437|.|sp|P16621|LAR_DROME Tyrosine-protein phosphatase Lar OS=Drosophila melanogaster GN=Lar PE=1 SV=2|e=2.38114E-40|from=2|to=436|frame=2|Fragment_3
SI
>Locus_16047_Transcript_3/5_Confidence_0.200_Length_437|.|sp|P16621|LAR_DROME Tyrosine-protein phosphatase Lar OS=Drosophila melanogaster GN=Lar PE=1 SV=2|e=2.38114E-40|from=2|to=436|frame=2|Fragment_4
RRRNRPCKAPDQAAVTRPLMAADLGAGPTPQDPVEMRRLNF
>Locus_26560_Transcript_1/1_Confidence_1.000_Length_276|.|sp|P16621|

LAR_DROME Tyrosine-protein phosphatase Lar OS=Drosophila melanogaster GN=Lar PE=1 SV=2|e=3.05006E-29|from=3|to=275|frame=-2|Fragment_1
EHINGGQITARRIGDLERGVEYEFRVAGRNAIGVGQEAVKYYSTPEGAPDGPPTEVKVRF
QTPDSIVVRWLPPTREHRNGRIVRYDAQLRR
>Locus_28164_Transcript_1/1_Confidence_1.000_Length_246|.|sp|P16621|LAR_DROME Tyrosine-protein phosphatase Lar OS=Drosophila melanogaster GN=Lar PE=1 SV=2|e=6.13889E-17|from=2|to=244|frame=2|Fragment_1
DESLAHKHPDQFLTDEMIAGAKMVNNNGAPGTSASMAKASLLDAPYIAAKFPQRNIPYTF
HLGSGDIYDGFTNRRLERDRR

>Locus_30763_Transcript_1/1_Confidence_1.000_Length_522|UNIGENE REPRESENTATIVE TRANSCRIPT|sp|P17971|KCNAL_DROME Potassium voltage-gated channel protein Shal OS=Drosophila melanogaster GN=Shal PE=1 SV=2|e=8.88888E-95|from=2|to=520|frame=-3|Fragment_1
PCGERYKIVFFCLDTACVMIFTAEYLLRLFAAPDRCKFVRSVMSIIDVVAILPYYIGLGI
TDNDDVSGAFVTLRVFRVFRIFKFSRHSQGLRILGYTLKSCASELGFLVFSLAMAIIIFA
TVMFYAEKNVDGTNFTSIPAAFWYTIVTMTTLGYGDMVPQTIAGKIVGGVCSL
>Locus_29656_Transcript_1/1_Confidence_1.000_Length_268|.|sp|P17971|KCNAL_DROME Potassium voltage-gated channel protein Shal OS=Drosophila melanogaster GN=Shal PE=1 SV=2|e=1.46442E-47|from=2|to=268|frame=2|Fragment_1
KLSENGDQNLQQLTNIRQKMWRAFENPHTSTAALVFYYVTGFFIAVSVMANVVETVPCGH
RPGRAGTLPCGERYKIVFFCLDTACVMIF
>Locus_12205_Transcript_1/3_Confidence_0.714_Length_2995|UNIGENE REPRESENTATIVE TRANSCRIPT|sp|P18168|SERR_DROME Protein serrate OS=Drosophila melanogaster GN=Ser PE=1 SV=3|e=0|from=509|to=2989|frame=-1|Fragment_1
ANGYQCLCDAGWEGPNCETNIDDCVGNPCEHGGSCIDLVDGFRCECPSEWTGEHCETDVD
EC
>Locus_12205_Transcript_1/3_Confidence_0.714_Length_2995|UNIGENE REPRESENTATIVE TRANSCRIPT|sp|P18168|SERR_DROME Protein serrate OS=Drosophila melanogaster GN=Ser PE=1 SV=3|e=0|from=509|to=2989|frame=-1|Fragment_2
LSS
>Locus_12205_Transcript_1/3_Confidence_0.714_Length_2995|UNIGENE REPRESENTATIVE TRANSCRIPT|sp|P18168|SERR_DROME Protein serrate OS=Drosophila melanogaster GN=Ser PE=1 SV=3|e=0|from=509|to=2989|frame=-1|Fragment_3
PCANAVSCSNTHGGFHCTCAPGWGGALCARNIDDCVGQCLNGATCIDLVDDYHCACASGY
TGRDCSQDVDECQTNPCRHGGECVDLVGGFRCICPVGRSGTLCEDDIDRCAPNPCQNGAE
CLNTPGGYYCHCPGGLTGRHCEGPRHPVLA
>Locus_12205_Transcript_1/3_Confidence_0.714_Length_2995|UNIGENE REPRESENTATIVE TRANSCRIPT|sp|P18168|SERR_DROME Protein serrate OS=Drosophila melanogaster GN=Ser PE=1 SV=3|e=0|from=509|to=2989|frame=-1|Fragment_4
NDGCMPN
>Locus_12205_Transcript_1/3_Confidence_0.714_Length_2995|UNIGENE REPRESENTATIVE TRANSCRIPT|sp|P18168|SERR_DROME Protein serrate OS=Drosophila melanogaster GN=Ser PE=1 SV=3|e=0|from=509|to=2989|frame=-1|Fragment_5
PCQNNGTCLLGGPNGAAFTCLCSAGTRGRLC
>Locus_12205_Transcript_1/3_Confidence_0.714_Length_2995|UNIGENE

REPRESENTATIVE TRANSCRIPT|sp|P18168|SERR_DROME Protein serrate
OS=Drosophila melanogaster GN=Ser PE=1 SV=3|e=0|from=509|to=2989|
frame=-1|Fragment_6
SEMSNSGVI
>Locus_12205_Transcript_1/3_Confidence_0.714_Length_2995|UNIGENE
REPRESENTATIVE TRANSCRIPT|sp|P18168|SERR_DROME Protein serrate
OS=Drosophila melanogaster GN=Ser PE=1 SV=3|e=0|from=509|to=2989|
frame=-1|Fragment_7
GGSEDDCEGVT
>Locus_12205_Transcript_1/3_Confidence_0.714_Length_2995|UNIGENE
REPRESENTATIVE TRANSCRIPT|sp|P18168|SERR_DROME Protein serrate
OS=Drosophila melanogaster GN=Ser PE=1 SV=3|e=0|from=509|to=2989|
frame=-1|Fragment_8
GRCLNGGIC
>Locus_12205_Transcript_1/3_Confidence_0.714_Length_2995|UNIGENE
REPRESENTATIVE TRANSCRIPT|sp|P18168|SERR_DROME Protein serrate
OS=Drosophila melanogaster GN=Ser PE=1 SV=3|e=0|from=509|to=2989|
frame=-1|Fragment_9
DLQRGRCNCLAGWTGPQCEEPLDQCHGEPCHNGGTCVGGPGWFRCECSRGFTGPDCRINV
NECASSPCQGGAPCKDRIGGYTCLCPPGRAGERCEIETSG
>Locus_12205_Transcript_1/3_Confidence_0.714_Length_2995|UNIGENE
REPRESENTATIVE TRANSCRIPT|sp|P18168|SERR_DROME Protein serrate
OS=Drosophila melanogaster GN=Ser PE=1 SV=3|e=0|from=509|to=2989|
frame=-1|Fragment_10
SGCWAPPNS
>Locus_12205_Transcript_1/3_Confidence_0.714_Length_2995|UNIGENE
REPRESENTATIVE TRANSCRIPT|sp|P18168|SERR_DROME Protein serrate
OS=Drosophila melanogaster GN=Ser PE=1 SV=3|e=0|from=509|to=2989|
frame=-1|Fragment_11
TWLN
>Locus_12205_Transcript_1/3_Confidence_0.714_Length_2995|UNIGENE
REPRESENTATIVE TRANSCRIPT|sp|P18168|SERR_DROME Protein serrate
OS=Drosophila melanogaster GN=Ser PE=1 SV=3|e=0|from=509|to=2989|
frame=-1|Fragment_12
ECNTCTCVQGIQRCSNLWCGLQDCLGVN
>Locus_12205_Transcript_1/3_Confidence_0.714_Length_2995|UNIGENE
REPRESENTATIVE TRANSCRIPT|sp|P18168|SERR_DROME Protein serrate
OS=Drosophila melanogaster GN=Ser PE=1 SV=3|e=0|from=509|to=2989|
frame=-1|Fragment_13
STKCAPHEVCVPAPQESCLSSPCTARGDCRALEPSRRVAPPALPASPDCWPNQAALGEQC
ARVSALLETGKAPRGATSETLCDALRTLLGARLVREPTADLRH
>Locus_12205_Transcript_1/3_Confidence_0.714_Length_2995|UNIGENE
REPRESENTATIVE TRANSCRIPT|sp|P18168|SERR_DROME Protein serrate
OS=Drosophila melanogaster GN=Ser PE=1 SV=3|e=0|from=509|to=2989|
frame=-1|Fragment_14
IVVVLCEMKTGSNDTVEVTVSSPPGGNAGAQIVALSVRLLGELISRGAAS
>Locus_12205_Transcript_1/3_Confidence_0.714_Length_2995|UNIGENE
REPRESENTATIVE TRANSCRIPT|sp|P18168|SERR_DROME Protein serrate
OS=Drosophila melanogaster GN=Ser PE=1 SV=3|e=0|from=509|to=2989|
frame=-1|Fragment_15
SDGALPGAIREVKVETALLSSETQGGEYFIALICVMSIIVLAAVVLGIFVMRQRMRGRSI
SGMGLTPSPSDAFRAEEEKSNNLQNEENFRRYANPLKEE
>Locus_12205_Transcript_1/3_Confidence_0.714_Length_2995|UNIGENE
REPRESENTATIVE TRANSCRIPT|sp|P18168|SERR_DROME Protein serrate

OS=Drosophila melanogaster GN=Ser PE=1 SV=3|e=0|from=509|to=2989|
frame=-1|Fragment_16
SIELSPKVSVEMHHHKSSQDLNPNVSLVRPIPGEALAGPSTTAQDFMHEYSQRLHKNKSL
NPDLVHVDADLLHVDCNLKTAH
>Locus_12205_Transcript_1/3_Confidence_0.714_Length_2995|UNIGENE
REPRESENTATIVE TRANSCRIPT|sp|P18168|SERR_DROME Protein serrate
OS=Drosophila melanogaster GN=Ser PE=1 SV=3|e=0|from=509|to=2989|
frame=-1|Fragment_17
RNSQILLYKAQNPDMRKNTTGTFDNLNKDFGKRSINM
>Locus_12205_Transcript_1/3_Confidence_0.714_Length_2995|UNIGENE
REPRESENTATIVE TRANSCRIPT|sp|P18168|SERR_DROME Protein serrate
OS=Drosophila melanogaster GN=Ser PE=1 SV=3|e=0|from=509|to=2989|
frame=-1|Fragment_18
STASSDSDVLTVLV
>Locus_12205_Transcript_3/3_Confidence_0.714_Length_2995|.|sp|
P18168|SERR_DROME Protein serrate OS=Drosophila melanogaster GN=Ser
PE=1 SV=3|e=0|from=509|to=2989|frame=-1|Fragment_1
ANGYQCLCDAGWEGPNCETNIDDCVGNPCEHGGSCIDLVDGFRCECPSEWTGEHCETDVD
EC
>Locus_12205_Transcript_3/3_Confidence_0.714_Length_2995|.|sp|
P18168|SERR_DROME Protein serrate OS=Drosophila melanogaster GN=Ser
PE=1 SV=3|e=0|from=509|to=2989|frame=-1|Fragment_2
LSS
>Locus_12205_Transcript_3/3_Confidence_0.714_Length_2995|.|sp|
P18168|SERR_DROME Protein serrate OS=Drosophila melanogaster GN=Ser
PE=1 SV=3|e=0|from=509|to=2989|frame=-1|Fragment_3
PCANAVSCSNTHGGFHCTCAPGWGGALCARNIDDCVGQCLNGATCIDLVDDYHCACASGY
TGRDCSQDVDECQTNPCRHGGECVDLVGGFRCICPVGRSGTLCEDDIDRCAPNPCQNGAE
CLNTPGGYYCHCPGGLTGRHCEGPRHPVLA
>Locus_12205_Transcript_3/3_Confidence_0.714_Length_2995|.|sp|
P18168|SERR_DROME Protein serrate OS=Drosophila melanogaster GN=Ser
PE=1 SV=3|e=0|from=509|to=2989|frame=-1|Fragment_4
NDGCMPN
>Locus_12205_Transcript_3/3_Confidence_0.714_Length_2995|.|sp|
P18168|SERR_DROME Protein serrate OS=Drosophila melanogaster GN=Ser
PE=1 SV=3|e=0|from=509|to=2989|frame=-1|Fragment_5
PCQNNGTCLLGGPNGAAFTCLCSAGTRGRLC
>Locus_12205_Transcript_3/3_Confidence_0.714_Length_2995|.|sp|
P18168|SERR_DROME Protein serrate OS=Drosophila melanogaster GN=Ser
PE=1 SV=3|e=0|from=509|to=2989|frame=-1|Fragment_6
SEMSNSGVI
>Locus_12205_Transcript_3/3_Confidence_0.714_Length_2995|.|sp|
P18168|SERR_DROME Protein serrate OS=Drosophila melanogaster GN=Ser
PE=1 SV=3|e=0|from=509|to=2989|frame=-1|Fragment_7
GGSEDDCEGVT
>Locus_12205_Transcript_3/3_Confidence_0.714_Length_2995|.|sp|
P18168|SERR_DROME Protein serrate OS=Drosophila melanogaster GN=Ser
PE=1 SV=3|e=0|from=509|to=2989|frame=-1|Fragment_8
GRCLNGGIC
>Locus_12205_Transcript_3/3_Confidence_0.714_Length_2995|.|sp|
P18168|SERR_DROME Protein serrate OS=Drosophila melanogaster GN=Ser
PE=1 SV=3|e=0|from=509|to=2989|frame=-1|Fragment_9
DLQRGRCNCLAGWTGPQCEEPLDQCHGEPCHNGGTCVGGPGWFRCECSRGFTGPDCRINV
NECASSPCQGGAPCKDRIGGYTCLCPPGRAGERCEIETSG

>Locus_12205_Transcript_3/3_Confidence_0.714_Length_2995|.|sp|
P18168|SERR_DROME Protein serrate OS=Drosophila melanogaster GN=Ser
PE=1 SV=3|e=0|from=509|to=2989|frame=-1|Fragment_10
SGCWAPPNS
>Locus_12205_Transcript_3/3_Confidence_0.714_Length_2995|.|sp|
P18168|SERR_DROME Protein serrate OS=Drosophila melanogaster GN=Ser
PE=1 SV=3|e=0|from=509|to=2989|frame=-1|Fragment_11
TWLN
>Locus_12205_Transcript_3/3_Confidence_0.714_Length_2995|.|sp|
P18168|SERR_DROME Protein serrate OS=Drosophila melanogaster GN=Ser
PE=1 SV=3|e=0|from=509|to=2989|frame=-1|Fragment_12
ECNTCTCVQGIQRCSNLWCGLQDCLGVN
>Locus_12205_Transcript_3/3_Confidence_0.714_Length_2995|.|sp|
P18168|SERR_DROME Protein serrate OS=Drosophila melanogaster GN=Ser
PE=1 SV=3|e=0|from=509|to=2989|frame=-1|Fragment_13
STKCAPHEVCVPAPQESCLSSPCTARGDCRALEPSRRVAPPALPASPDCWPNQAALGEQC
ARVSALLETGKAPRGATSETLCDALRTLLGARLVREPTADLRH
>Locus_12205_Transcript_3/3_Confidence_0.714_Length_2995|.|sp|
P18168|SERR_DROME Protein serrate OS=Drosophila melanogaster GN=Ser
PE=1 SV=3|e=0|from=509|to=2989|frame=-1|Fragment_14
IVVVLCEMKTGSNDTVEVTVSSPPGGNAGAQIVALSVRLLGELISRGAAS
>Locus_12205_Transcript_3/3_Confidence_0.714_Length_2995|.|sp|
P18168|SERR_DROME Protein serrate OS=Drosophila melanogaster GN=Ser
PE=1 SV=3|e=0|from=509|to=2989|frame=-1|Fragment_15
SDGALPGAIREVKVETALLSSETQGGEYFIALICVMSIIVLAAVVLGIFVMRQRMRGRSI
SGMGLTPSPSDAFRAEEEKSNNLQNEENFRRYANPLKEE
>Locus_12205_Transcript_3/3_Confidence_0.714_Length_2995|.|sp|
P18168|SERR_DROME Protein serrate OS=Drosophila melanogaster GN=Ser
PE=1 SV=3|e=0|from=509|to=2989|frame=-1|Fragment_16
SIELSPKVSVEMHHHKSSQDLNPNVSLVRPIPGEALAGPSTTAQDFMHEYSQRLHKNKSL
NPDLVHVDADLLHVDCNLKTAH
>Locus_12205_Transcript_3/3_Confidence_0.714_Length_2995|.|sp|
P18168|SERR_DROME Protein serrate OS=Drosophila melanogaster GN=Ser
PE=1 SV=3|e=0|from=509|to=2989|frame=-1|Fragment_17
RNSQILLYKAQNPDMRKNTTGTFDNLNKDFGKRSINM
>Locus_12205_Transcript_3/3_Confidence_0.714_Length_2995|.|sp|
P18168|SERR_DROME Protein serrate OS=Drosophila melanogaster GN=Ser
PE=1 SV=3|e=0|from=509|to=2989|frame=-1|Fragment_18
STASSDSDVLTVLV
>Locus_12205_Transcript_1/3_Confidence_0.714_Length_2995|.|sp|
P18168|SERR_DROME Protein serrate OS=Drosophila melanogaster GN=Ser
PE=1 SV=3|e=2.69588E-57|from=1859|to=2986|frame=-1|Fragment_1
NGYQCLCDAGWEGPNCETNIDDC
>Locus_12205_Transcript_1/3_Confidence_0.714_Length_2995|.|sp|
P18168|SERR_DROME Protein serrate OS=Drosophila melanogaster GN=Ser
PE=1 SV=3|e=2.69588E-57|from=1859|to=2986|frame=-1|Fragment_2
VGNPCEHGGSCIDLV
>Locus_12205_Transcript_1/3_Confidence_0.714_Length_2995|.|sp|
P18168|SERR_DROME Protein serrate OS=Drosophila melanogaster GN=Ser
PE=1 SV=3|e=2.69588E-57|from=1859|to=2986|frame=-1|Fragment_3
DGFRCECPSEWTGEHCETDVDECLSSPCANAVSCS
>Locus_12205_Transcript_1/3_Confidence_0.714_Length_2995|.|sp|
P18168|SERR_DROME Protein serrate OS=Drosophila melanogaster GN=Ser
PE=1 SV=3|e=2.69588E-57|from=1859|to=2986|frame=-1|Fragment_4

NTHGGFHCTCAPGWGGALCARNIDDC
>Locus_12205_Transcript_1/3_Confidence_0.714_Length_2995|.|sp|
P18168|SERR_DROME Protein serrate OS=Drosophila melanogaster GN=Ser
PE=1 SV=3|e=2.69588E-57|from=1859|to=2986|frame=-1|Fragment_5
VGQCLNGATCIDLVD
>Locus_12205_Transcript_1/3_Confidence_0.714_Length_2995|.|sp|
P18168|SERR_DROME Protein serrate OS=Drosophila melanogaster GN=Ser
PE=1 SV=3|e=2.69588E-57|from=1859|to=2986|frame=-1|Fragment_6
DYHCACASGYTGRDCSQDVDECQTNPCRHGGECVDLVGGFRCICPVGRSGTLCEDDIDRC
>Locus_12205_Transcript_1/3_Confidence_0.714_Length_2995|.|sp|
P18168|SERR_DROME Protein serrate OS=Drosophila melanogaster GN=Ser
PE=1 SV=3|e=2.69588E-57|from=1859|to=2986|frame=-1|Fragment_7
APNPCQNGAECLNTPGGYYCHCPGGLTGRHCEGPRHPVLANDGCMPNPCQNNGTCLLGGP
NGAAFTCLCSAGTRGRLCSEMSNSGVIGGSEDDCEGVTGRCLNGGIC
>Locus_12205_Transcript_1/3_Confidence_0.714_Length_2995|.|sp|
P18168|SERR_DROME Protein serrate OS=Drosophila melanogaster GN=Ser
PE=1 SV=3|e=2.69588E-57|from=1859|to=2986|frame=-1|Fragment_8
DLQRG
>Locus_12205_Transcript_1/3_Confidence_0.714_Length_2995|.|sp|
P18168|SERR_DROME Protein serrate OS=Drosophila melanogaster GN=Ser
PE=1 SV=3|e=2.69588E-57|from=1859|to=2986|frame=-1|Fragment_9
RCNCLAGWTGPQCEEPLDQCHGEPCHNGGTCVGGPGWFRCECSRGFTGPDCRINVNECAS
SPCQGGAPCKDRIGGYTCLCPPGRAGERCE
>Locus_12205_Transcript_3/3_Confidence_0.714_Length_2995|.|sp|
P18168|SERR_DROME Protein serrate OS=Drosophila melanogaster GN=Ser
PE=1 SV=3|e=2.69588E-57|from=1859|to=2986|frame=-1|Fragment_1
NGYQCLCDAGWEGPNCETNIDDC
>Locus_12205_Transcript_3/3_Confidence_0.714_Length_2995|.|sp|
P18168|SERR_DROME Protein serrate OS=Drosophila melanogaster GN=Ser
PE=1 SV=3|e=2.69588E-57|from=1859|to=2986|frame=-1|Fragment_2
VGNPCEHGGSCIDLV
>Locus_12205_Transcript_3/3_Confidence_0.714_Length_2995|.|sp|
P18168|SERR_DROME Protein serrate OS=Drosophila melanogaster GN=Ser
PE=1 SV=3|e=2.69588E-57|from=1859|to=2986|frame=-1|Fragment_3
DGFRCECPSEWTGEHCETDVDECLSSPCANAVSCS
>Locus_12205_Transcript_3/3_Confidence_0.714_Length_2995|.|sp|
P18168|SERR_DROME Protein serrate OS=Drosophila melanogaster GN=Ser
PE=1 SV=3|e=2.69588E-57|from=1859|to=2986|frame=-1|Fragment_4
NTHGGFHCTCAPGWGGALCARNIDDC
>Locus_12205_Transcript_3/3_Confidence_0.714_Length_2995|.|sp|
P18168|SERR_DROME Protein serrate OS=Drosophila melanogaster GN=Ser
PE=1 SV=3|e=2.69588E-57|from=1859|to=2986|frame=-1|Fragment_5
VGQCLNGATCIDLVD
>Locus_12205_Transcript_3/3_Confidence_0.714_Length_2995|.|sp|
P18168|SERR_DROME Protein serrate OS=Drosophila melanogaster GN=Ser
PE=1 SV=3|e=2.69588E-57|from=1859|to=2986|frame=-1|Fragment_6
DYHCACASGYTGRDCSQDVDECQTNPCRHGGECVDLVGGFRCICPVGRSGTLCEDDIDRC
>Locus_12205_Transcript_3/3_Confidence_0.714_Length_2995|.|sp|
P18168|SERR_DROME Protein serrate OS=Drosophila melanogaster GN=Ser
PE=1 SV=3|e=2.69588E-57|from=1859|to=2986|frame=-1|Fragment_7
APNPCQNGAECLNTPGGYYCHCPGGLTGRHCEGPRHPVLANDGCMPNPCQNNGTCLLGGP
NGAAFTCLCSAGTRGRLCSEMSNSGVIGGSEDDCEGVTGRCLNGGIC
>Locus_12205_Transcript_3/3_Confidence_0.714_Length_2995|.|sp|
P18168|SERR_DROME Protein serrate OS=Drosophila melanogaster GN=Ser

PE=1 SV=3|e=2.69588E-57|from=1859|to=2986|frame=-1|Fragment_8
DLQRG
>Locus_12205_Transcript_3/3_Confidence_0.714_Length_2995|.|sp|
P18168|SERR_DROME Protein serrate OS=Drosophila melanogaster GN=Ser
PE=1 SV=3|e=2.69588E-57|from=1859|to=2986|frame=-1|Fragment_9
RCNCLAGWTGPQCEEPLDQCHGEPCHNGGTCVGGPGWFRCECSRGFTGPDCRINVNECAS
SPCQGGAPCKDRIGGYTCLCPPGRAGERCE
>Locus_12205_Transcript_1/3_Confidence_0.714_Length_2995|.|sp|
P18168|SERR_DROME Protein serrate OS=Drosophila melanogaster GN=Ser
PE=1 SV=3|e=2.2875E-48|from=1859|to=2977|frame=-1|Fragment_1
QCLCDAGWEGPNCETNID
>Locus_12205_Transcript_1/3_Confidence_0.714_Length_2995|.|sp|
P18168|SERR_DROME Protein serrate OS=Drosophila melanogaster GN=Ser
PE=1 SV=3|e=2.2875E-48|from=1859|to=2977|frame=-1|Fragment_2
DCVGNPCEHGGSCIDLVDGFRCECPSEWTGEHCETDVDECLSSPCANAVSCSNTHGGFHC
TCAPGWGGALCARNIDDCVGQ
>Locus_12205_Transcript_1/3_Confidence_0.714_Length_2995|.|sp|
P18168|SERR_DROME Protein serrate OS=Drosophila melanogaster GN=Ser
PE=1 SV=3|e=2.2875E-48|from=1859|to=2977|frame=-1|Fragment_3
CLNGATCIDLVDD
>Locus_12205_Transcript_1/3_Confidence_0.714_Length_2995|.|sp|
P18168|SERR_DROME Protein serrate OS=Drosophila melanogaster GN=Ser
PE=1 SV=3|e=2.2875E-48|from=1859|to=2977|frame=-1|Fragment_4
YHCACASGYTGRDCSQDVDECQTNPCRHGGECV
>Locus_12205_Transcript_1/3_Confidence_0.714_Length_2995|.|sp|
P18168|SERR_DROME Protein serrate OS=Drosophila melanogaster GN=Ser
PE=1 SV=3|e=2.2875E-48|from=1859|to=2977|frame=-1|Fragment_5
DLVGGFRCICPVGRSGTLCEDDIDRCAP
>Locus_12205_Transcript_1/3_Confidence_0.714_Length_2995|.|sp|
P18168|SERR_DROME Protein serrate OS=Drosophila melanogaster GN=Ser
PE=1 SV=3|e=2.2875E-48|from=1859|to=2977|frame=-1|Fragment_6
NPCQNGAECLNTPG
>Locus_12205_Transcript_1/3_Confidence_0.714_Length_2995|.|sp|
P18168|SERR_DROME Protein serrate OS=Drosophila melanogaster GN=Ser
PE=1 SV=3|e=2.2875E-48|from=1859|to=2977|frame=-1|Fragment_7
GYYCHCPGGLTGRHCEGPRHPVLANDGCMPNPCQNNGTC
>Locus_12205_Transcript_1/3_Confidence_0.714_Length_2995|.|sp|
P18168|SERR_DROME Protein serrate OS=Drosophila melanogaster GN=Ser
PE=1 SV=3|e=2.2875E-48|from=1859|to=2977|frame=-1|Fragment_8
LLGGPNGAAFTCLCSAGTRGRLC
>Locus_12205_Transcript_1/3_Confidence_0.714_Length_2995|.|sp|
P18168|SERR_DROME Protein serrate OS=Drosophila melanogaster GN=Ser
PE=1 SV=3|e=2.2875E-48|from=1859|to=2977|frame=-1|Fragment_9
SEMSNSGVIGGSEDDCEGVT
>Locus_12205_Transcript_1/3_Confidence_0.714_Length_2995|.|sp|
P18168|SERR_DROME Protein serrate OS=Drosophila melanogaster GN=Ser
PE=1 SV=3|e=2.2875E-48|from=1859|to=2977|frame=-1|Fragment_10
GRCLNGGICDLQRG
>Locus_12205_Transcript_1/3_Confidence_0.714_Length_2995|.|sp|
P18168|SERR_DROME Protein serrate OS=Drosophila melanogaster GN=Ser
PE=1 SV=3|e=2.2875E-48|from=1859|to=2977|frame=-1|Fragment_11
RCNCLAGWTGPQCEEPLDQCHGEPCHNGGTCVGGPGWFRCECSRGFTGPDCRINVNECAS
SPCQGGAPCKDRIGGYTCLCPPGRAGERCE
>Locus_12205_Transcript_3/3_Confidence_0.714_Length_2995|.|sp|

P18168|SERR_DROME Protein serrate OS=Drosophila melanogaster GN=Ser PE=1 SV=3|e=2.2875E-48|from=1859|to=2977|frame=-1|Fragment_1
QCLCDAGWEGPNCETNID
>Locus_12205_Transcript_3/3_Confidence_0.714_Length_2995|.|sp|P18168|SERR_DROME Protein serrate OS=Drosophila melanogaster GN=Ser PE=1 SV=3|e=2.2875E-48|from=1859|to=2977|frame=-1|Fragment_2
DCVGNPCEHGGSCIDLVDGFRCECPSEWTGEHCETDVDECLSSPCANAVSCSNTHGGFHC
TCAPGWGGALCARNIDDCVGQ
>Locus_12205_Transcript_3/3_Confidence_0.714_Length_2995|.|sp|P18168|SERR_DROME Protein serrate OS=Drosophila melanogaster GN=Ser PE=1 SV=3|e=2.2875E-48|from=1859|to=2977|frame=-1|Fragment_3
CLNGATCIDLVDD
>Locus_12205_Transcript_3/3_Confidence_0.714_Length_2995|.|sp|P18168|SERR_DROME Protein serrate OS=Drosophila melanogaster GN=Ser PE=1 SV=3|e=2.2875E-48|from=1859|to=2977|frame=-1|Fragment_4
YHCACASGYTGRDCSQDVDECQTNPCRHGGECV
>Locus_12205_Transcript_3/3_Confidence_0.714_Length_2995|.|sp|P18168|SERR_DROME Protein serrate OS=Drosophila melanogaster GN=Ser PE=1 SV=3|e=2.2875E-48|from=1859|to=2977|frame=-1|Fragment_5
DLVGGFRCICPVGRSGTLCEDDIDRCAP
>Locus_12205_Transcript_3/3_Confidence_0.714_Length_2995|.|sp|P18168|SERR_DROME Protein serrate OS=Drosophila melanogaster GN=Ser PE=1 SV=3|e=2.2875E-48|from=1859|to=2977|frame=-1|Fragment_6
NPCQNGAECLNTPG
>Locus_12205_Transcript_3/3_Confidence_0.714_Length_2995|.|sp|P18168|SERR_DROME Protein serrate OS=Drosophila melanogaster GN=Ser PE=1 SV=3|e=2.2875E-48|from=1859|to=2977|frame=-1|Fragment_7
GYYCHCPGGLTGRHCEGPRHPVLANDGCMPNPCQNNGTC
>Locus_12205_Transcript_3/3_Confidence_0.714_Length_2995|.|sp|P18168|SERR_DROME Protein serrate OS=Drosophila melanogaster GN=Ser PE=1 SV=3|e=2.2875E-48|from=1859|to=2977|frame=-1|Fragment_8
LLGGPNGAAFTCLCSAGTRGRLC
>Locus_12205_Transcript_3/3_Confidence_0.714_Length_2995|.|sp|P18168|SERR_DROME Protein serrate OS=Drosophila melanogaster GN=Ser PE=1 SV=3|e=2.2875E-48|from=1859|to=2977|frame=-1|Fragment_9
SEMSNSGVIGGSEDDCEGVT
>Locus_12205_Transcript_3/3_Confidence_0.714_Length_2995|.|sp|P18168|SERR_DROME Protein serrate OS=Drosophila melanogaster GN=Ser PE=1 SV=3|e=2.2875E-48|from=1859|to=2977|frame=-1|Fragment_10
GRCLNGGICDLQRG
>Locus_12205_Transcript_3/3_Confidence_0.714_Length_2995|.|sp|P18168|SERR_DROME Protein serrate OS=Drosophila melanogaster GN=Ser PE=1 SV=3|e=2.2875E-48|from=1859|to=2977|frame=-1|Fragment_11
RCNCLAGWTGPQCEEPLDQCHGEPCHNGGTCVGGPGWFRCECSRGFTGPDCRINVNECAS
SPCQGGAPCKDRIGGYTCLCPPGRAGERCE
>Locus_12205_Transcript_1/3_Confidence_0.714_Length_2995|.|sp|P18168|SERR_DROME Protein serrate OS=Drosophila melanogaster GN=Ser PE=1 SV=3|e=9.30868E-42|from=1766|to=2995|frame=-1|Fragment_1
NAANGYQCLCDAGWEGPNCETNIDDCVGNPCEHG
>Locus_12205_Transcript_1/3_Confidence_0.714_Length_2995|.|sp|P18168|SERR_DROME Protein serrate OS=Drosophila melanogaster GN=Ser PE=1 SV=3|e=9.30868E-42|from=1766|to=2995|frame=-1|Fragment_2
GSCIDLVDGFRCECPSEWTGEHCETDVDECLSSPCANAVSCSNTHGGFHCTCAPGWGGAL
CARNIDDC

>Locus_12205_Transcript_1/3_Confidence_0.714_Length_2995|.|sp|
P18168|SERR_DROME Protein serrate OS=Drosophila melanogaster GN=Ser
PE=1 SV=3|e=9.30868E-42|from=1766|to=2995|frame=-1|Fragment_3
VGQCLNGATCIDLVDDY
>Locus_12205_Transcript_1/3_Confidence_0.714_Length_2995|.|sp|
P18168|SERR_DROME Protein serrate OS=Drosophila melanogaster GN=Ser
PE=1 SV=3|e=9.30868E-42|from=1766|to=2995|frame=-1|Fragment_4
HCACASGYTGRDCSQDVDECQTNPCRHGGECVDLVGGFRCICPVGRSGTLCEDDIDRCAP
NPCQNGAECLNTPGGYYCHCPGGLTGRHCEGPRHPVLANDGCMPNPCQNNGTCLLGGPNG
AAFTCLCSAGTRGRLCSEMSNSGVIGGSEDDCEGVTGRCLNGGICDLQRGRCNCLAGWTG
>Locus_12205_Transcript_1/3_Confidence_0.714_Length_2995|.|sp|
P18168|SERR_DROME Protein serrate OS=Drosophila melanogaster GN=Ser
PE=1 SV=3|e=9.30868E-42|from=1766|to=2995|frame=-1|Fragment_5
PQC
>Locus_12205_Transcript_1/3_Confidence_0.714_Length_2995|.|sp|
P18168|SERR_DROME Protein serrate OS=Drosophila melanogaster GN=Ser
PE=1 SV=3|e=9.30868E-42|from=1766|to=2995|frame=-1|Fragment_6
EEPLDQCH
>Locus_12205_Transcript_1/3_Confidence_0.714_Length_2995|.|sp|
P18168|SERR_DROME Protein serrate OS=Drosophila melanogaster GN=Ser
PE=1 SV=3|e=9.30868E-42|from=1766|to=2995|frame=-1|Fragment_7
GEPCHNGGTCVGGPGWFRCECSRGFTGPDCRINVNECASSPCQGGAPCKDRIGGYTCLCP
PGRAGERCEIETSGSGCWAPPNSTWLNE
>Locus_12205_Transcript_1/3_Confidence_0.714_Length_2995|.|sp|
P18168|SERR_DROME Protein serrate OS=Drosophila melanogaster GN=Ser
PE=1 SV=3|e=9.30868E-42|from=1766|to=2995|frame=-1|Fragment_8
CNTCTCVQGIQR
>Locus_12205_Transcript_3/3_Confidence_0.714_Length_2995|.|sp|
P18168|SERR_DROME Protein serrate OS=Drosophila melanogaster GN=Ser
PE=1 SV=3|e=9.30868E-42|from=1766|to=2995|frame=-1|Fragment_1
NAANGYQCLCDAGWEGPNCETNIDDCVGNPCEHG
>Locus_12205_Transcript_3/3_Confidence_0.714_Length_2995|.|sp|
P18168|SERR_DROME Protein serrate OS=Drosophila melanogaster GN=Ser
PE=1 SV=3|e=9.30868E-42|from=1766|to=2995|frame=-1|Fragment_2
GSCIDLVDGFRCECPSEWTGEHCETDVDECLSSPCANAVSCSNTHGGFHCTCAPGWGGAL
CARNIDDC
>Locus_12205_Transcript_3/3_Confidence_0.714_Length_2995|.|sp|
P18168|SERR_DROME Protein serrate OS=Drosophila melanogaster GN=Ser
PE=1 SV=3|e=9.30868E-42|from=1766|to=2995|frame=-1|Fragment_3
VGQCLNGATCIDLVDDY
>Locus_12205_Transcript_3/3_Confidence_0.714_Length_2995|.|sp|
P18168|SERR_DROME Protein serrate OS=Drosophila melanogaster GN=Ser
PE=1 SV=3|e=9.30868E-42|from=1766|to=2995|frame=-1|Fragment_4
HCACASGYTGRDCSQDVDECQTNPCRHGGECVDLVGGFRCICPVGRSGTLCEDDIDRCAP
NPCQNGAECLNTPGGYYCHCPGGLTGRHCEGPRHPVLANDGCMPNPCQNNGTCLLGGPNG
AAFTCLCSAGTRGRLCSEMSNSGVIGGSEDDCEGVTGRCLNGGICDLQRGRCNCLAGWTG
>Locus_12205_Transcript_3/3_Confidence_0.714_Length_2995|.|sp|
P18168|SERR_DROME Protein serrate OS=Drosophila melanogaster GN=Ser
PE=1 SV=3|e=9.30868E-42|from=1766|to=2995|frame=-1|Fragment_5
PQC
>Locus_12205_Transcript_3/3_Confidence_0.714_Length_2995|.|sp|
P18168|SERR_DROME Protein serrate OS=Drosophila melanogaster GN=Ser
PE=1 SV=3|e=9.30868E-42|from=1766|to=2995|frame=-1|Fragment_6
EEPLDQCH

>Locus_12205_Transcript_3/3_Confidence_0.714_Length_2995|.|sp|
P18168|SERR_DROME Protein serrate OS=Drosophila melanogaster GN=Ser
PE=1 SV=3|e=9.30868E-42|from=1766|to=2995|frame=-1|Fragment_7
GEPCHNGGTCVGGPGWFRCECSRGFTGPDCRINVNECASSPCQGGAPCKDRIGGYTCLCP
PGRAGERCEIETSGSGCWAPPNSTWLNE
>Locus_12205_Transcript_3/3_Confidence_0.714_Length_2995|.|sp|
P18168|SERR_DROME Protein serrate OS=Drosophila melanogaster GN=Ser
PE=1 SV=3|e=9.30868E-42|from=1766|to=2995|frame=-1|Fragment_8
CNTCTCVQGIQR
>Locus_12205_Transcript_1/3_Confidence_0.714_Length_2995|.|sp|
P18168|SERR_DROME Protein serrate OS=Drosophila melanogaster GN=Ser
PE=1 SV=3|e=2.37268E-37|from=1655|to=2740|frame=-1|Fragment_1
CAPGWGG
>Locus_12205_Transcript_1/3_Confidence_0.714_Length_2995|.|sp|
P18168|SERR_DROME Protein serrate OS=Drosophila melanogaster GN=Ser
PE=1 SV=3|e=2.37268E-37|from=1655|to=2740|frame=-1|Fragment_2
ALCARNIDDCVGQCLNGATCIDLVDDYHCACASGYTGRDCSQDVDECQTNP
>Locus_12205_Transcript_1/3_Confidence_0.714_Length_2995|.|sp|
P18168|SERR_DROME Protein serrate OS=Drosophila melanogaster GN=Ser
PE=1 SV=3|e=2.37268E-37|from=1655|to=2740|frame=-1|Fragment_3
CRHGGECVDLVGGFRCICPVGRSGTLCEDDIDRCAPN
>Locus_12205_Transcript_1/3_Confidence_0.714_Length_2995|.|sp|
P18168|SERR_DROME Protein serrate OS=Drosophila melanogaster GN=Ser
PE=1 SV=3|e=2.37268E-37|from=1655|to=2740|frame=-1|Fragment_4
PCQNGAECLNT
>Locus_12205_Transcript_1/3_Confidence_0.714_Length_2995|.|sp|
P18168|SERR_DROME Protein serrate OS=Drosophila melanogaster GN=Ser
PE=1 SV=3|e=2.37268E-37|from=1655|to=2740|frame=-1|Fragment_5
PGGYYCHCPGGLTGRHCEGPRHPVLANDGCMPNPCQNNGTCLLGGPNGAAFTCLCSAGTR
GRLCSEMSNSGVIGGSEDDCEGVTGRCLNG
>Locus_12205_Transcript_1/3_Confidence_0.714_Length_2995|.|sp|
P18168|SERR_DROME Protein serrate OS=Drosophila melanogaster GN=Ser
PE=1 SV=3|e=2.37268E-37|from=1655|to=2740|frame=-1|Fragment_6
GICDLQRG
>Locus_12205_Transcript_1/3_Confidence_0.714_Length_2995|.|sp|
P18168|SERR_DROME Protein serrate OS=Drosophila melanogaster GN=Ser
PE=1 SV=3|e=2.37268E-37|from=1655|to=2740|frame=-1|Fragment_7
RCNCLAGWTGPQCEEPLDQCHGEPCHNGGTCVGGPGWFRCECSRGFTGPDCRINVNEC
>Locus_12205_Transcript_1/3_Confidence_0.714_Length_2995|.|sp|
P18168|SERR_DROME Protein serrate OS=Drosophila melanogaster GN=Ser
PE=1 SV=3|e=2.37268E-37|from=1655|to=2740|frame=-1|Fragment_8
ASSPCQGGAPCKDRIGGYTCLCPPGRAGERCEIETSGSGCWAPPNSTWLNECN
>Locus_12205_Transcript_1/3_Confidence_0.714_Length_2995|.|sp|
P18168|SERR_DROME Protein serrate OS=Drosophila melanogaster GN=Ser
PE=1 SV=3|e=2.37268E-37|from=1655|to=2740|frame=-1|Fragment_9
TCTCVQG
>Locus_12205_Transcript_1/3_Confidence_0.714_Length_2995|.|sp|
P18168|SERR_DROME Protein serrate OS=Drosophila melanogaster GN=Ser
PE=1 SV=3|e=2.37268E-37|from=1655|to=2740|frame=-1|Fragment_10
IQRCSNLWC
>Locus_12205_Transcript_1/3_Confidence_0.714_Length_2995|.|sp|
P18168|SERR_DROME Protein serrate OS=Drosophila melanogaster GN=Ser
PE=1 SV=3|e=2.37268E-37|from=1655|to=2740|frame=-1|Fragment_11
GLQDC

>Locus_12205_Transcript_1/3_Confidence_0.714_Length_2995|.|sp|
P18168|SERR_DROME Protein serrate OS=Drosophila melanogaster GN=Ser
PE=1 SV=3|e=2.37268E-37|from=1655|to=2740|frame=-1|Fragment_12
LGVNSTKC
>Locus_12205_Transcript_1/3_Confidence_0.714_Length_2995|.|sp|
P18168|SERR_DROME Protein serrate OS=Drosophila melanogaster GN=Ser
PE=1 SV=3|e=2.37268E-37|from=1655|to=2740|frame=-1|Fragment_13
APHEVCVPAP
>Locus_12205_Transcript_1/3_Confidence_0.714_Length_2995|.|sp|
P18168|SERR_DROME Protein serrate OS=Drosophila melanogaster GN=Ser
PE=1 SV=3|e=2.37268E-37|from=1655|to=2740|frame=-1|Fragment_14
QESCLSSP
>Locus_12205_Transcript_3/3_Confidence_0.714_Length_2995|.|sp|
P18168|SERR_DROME Protein serrate OS=Drosophila melanogaster GN=Ser
PE=1 SV=3|e=2.37268E-37|from=1655|to=2740|frame=-1|Fragment_1
CAPGWGG
>Locus_12205_Transcript_3/3_Confidence_0.714_Length_2995|.|sp|
P18168|SERR_DROME Protein serrate OS=Drosophila melanogaster GN=Ser
PE=1 SV=3|e=2.37268E-37|from=1655|to=2740|frame=-1|Fragment_2
ALCARNIDDCVGQCLNGATCIDLVDDYHCACASGYTGRDCSQDVDECQTNP
>Locus_12205_Transcript_3/3_Confidence_0.714_Length_2995|.|sp|
P18168|SERR_DROME Protein serrate OS=Drosophila melanogaster GN=Ser
PE=1 SV=3|e=2.37268E-37|from=1655|to=2740|frame=-1|Fragment_3
CRHGGECVDLVGGFRCICPVGRSGTLCEDDIDRCAPN
>Locus_12205_Transcript_3/3_Confidence_0.714_Length_2995|.|sp|
P18168|SERR_DROME Protein serrate OS=Drosophila melanogaster GN=Ser
PE=1 SV=3|e=2.37268E-37|from=1655|to=2740|frame=-1|Fragment_4
PCQNGAECLNT
>Locus_12205_Transcript_3/3_Confidence_0.714_Length_2995|.|sp|
P18168|SERR_DROME Protein serrate OS=Drosophila melanogaster GN=Ser
PE=1 SV=3|e=2.37268E-37|from=1655|to=2740|frame=-1|Fragment_5
PGGYYCHCPGGLTGRHCEGPRHPVLANDGCMPNPCQNNGTCLLGGPNGAAFTCLCSAGTR
GRLCSEMSNSGVIGGSEDDCEGVTGRCLNG
>Locus_12205_Transcript_3/3_Confidence_0.714_Length_2995|.|sp|
P18168|SERR_DROME Protein serrate OS=Drosophila melanogaster GN=Ser
PE=1 SV=3|e=2.37268E-37|from=1655|to=2740|frame=-1|Fragment_6
GICDLQRG
>Locus_12205_Transcript_3/3_Confidence_0.714_Length_2995|.|sp|
P18168|SERR_DROME Protein serrate OS=Drosophila melanogaster GN=Ser
PE=1 SV=3|e=2.37268E-37|from=1655|to=2740|frame=-1|Fragment_7
RCNCLAGWTGPQCEEPLDQCHGEPCHNGGTCVGGPGWFRCECSRGFTGPDCRINVNEC
>Locus_12205_Transcript_3/3_Confidence_0.714_Length_2995|.|sp|
P18168|SERR_DROME Protein serrate OS=Drosophila melanogaster GN=Ser
PE=1 SV=3|e=2.37268E-37|from=1655|to=2740|frame=-1|Fragment_8
ASSPCQGGAPCKDRIGGYTCLCPPGRAGERCEIETSGSGCWAPPNSTWLNECN
>Locus_12205_Transcript_3/3_Confidence_0.714_Length_2995|.|sp|
P18168|SERR_DROME Protein serrate OS=Drosophila melanogaster GN=Ser
PE=1 SV=3|e=2.37268E-37|from=1655|to=2740|frame=-1|Fragment_9
TCTCVQG
>Locus_12205_Transcript_3/3_Confidence_0.714_Length_2995|.|sp|
P18168|SERR_DROME Protein serrate OS=Drosophila melanogaster GN=Ser
PE=1 SV=3|e=2.37268E-37|from=1655|to=2740|frame=-1|Fragment_10
IQRCSNLWC
>Locus_12205_Transcript_3/3_Confidence_0.714_Length_2995|.|sp|

P18168|SERR_DROME Protein serrate OS=Drosophila melanogaster GN=Ser
PE=1 SV=3|e=2.37268E-37|from=1655|to=2740|frame=-1|Fragment_11
GLQDC
>Locus_12205_Transcript_3/3_Confidence_0.714_Length_2995|.|sp|
P18168|SERR_DROME Protein serrate OS=Drosophila melanogaster GN=Ser
PE=1 SV=3|e=2.37268E-37|from=1655|to=2740|frame=-1|Fragment_12
LGVNSTKC
>Locus_12205_Transcript_3/3_Confidence_0.714_Length_2995|.|sp|
P18168|SERR_DROME Protein serrate OS=Drosophila melanogaster GN=Ser
PE=1 SV=3|e=2.37268E-37|from=1655|to=2740|frame=-1|Fragment_13
APHEVCVPAP
>Locus_12205_Transcript_3/3_Confidence_0.714_Length_2995|.|sp|
P18168|SERR_DROME Protein serrate OS=Drosophila melanogaster GN=Ser
PE=1 SV=3|e=2.37268E-37|from=1655|to=2740|frame=-1|Fragment_14
QESCLSSP
>Locus_12205_Transcript_1/3_Confidence_0.714_Length_2995|.|sp|
P18168|SERR_DROME Protein serrate OS=Drosophila melanogaster GN=Ser
PE=1 SV=3|e=2.38925E-13|from=1922|to=2560|frame=-1|Fragment_1
HGGECVDLVGGFRCICPVGRSGTLCEDDIDRCAPNPCQNGAECLNTPGGYYCHCPGGLTG
RHCEGPRHPVLANDGCMPNPCQNNGTCLLGGPNGAAFTCLCSAGTRGRLCSEMSNSGVIG
GSEDDCEGVTGRCLNGGICDLQRGRCNCLAGWTGPQCEEPLDQCHG
>Locus_12205_Transcript_1/3_Confidence_0.714_Length_2995|.|sp|
P18168|SERR_DROME Protein serrate OS=Drosophila melanogaster GN=Ser
PE=1 SV=3|e=2.38925E-13|from=1922|to=2560|frame=-1|Fragment_2
EPCHNGGTCVG
>Locus_12205_Transcript_1/3_Confidence_0.714_Length_2995|.|sp|
P18168|SERR_DROME Protein serrate OS=Drosophila melanogaster GN=Ser
PE=1 SV=3|e=2.38925E-13|from=1922|to=2560|frame=-1|Fragment_3
GPGWFRCECSRGFTGPDCRINVNECASSPCQGGAPC
>Locus_12205_Transcript_3/3_Confidence_0.714_Length_2995|.|sp|
P18168|SERR_DROME Protein serrate OS=Drosophila melanogaster GN=Ser
PE=1 SV=3|e=2.38925E-13|from=1922|to=2560|frame=-1|Fragment_1
HGGECVDLVGGFRCICPVGRSGTLCEDDIDRCAPNPCQNGAECLNTPGGYYCHCPGGLTG
RHCEGPRHPVLANDGCMPNPCQNNGTCLLGGPNGAAFTCLCSAGTRGRLCSEMSNSGVIG
GSEDDCEGVTGRCLNGGICDLQRGRCNCLAGWTGPQCEEPLDQCHG
>Locus_12205_Transcript_3/3_Confidence_0.714_Length_2995|.|sp|
P18168|SERR_DROME Protein serrate OS=Drosophila melanogaster GN=Ser
PE=1 SV=3|e=2.38925E-13|from=1922|to=2560|frame=-1|Fragment_2
EPCHNGGTCVG
>Locus_12205_Transcript_3/3_Confidence_0.714_Length_2995|.|sp|
P18168|SERR_DROME Protein serrate OS=Drosophila melanogaster GN=Ser
PE=1 SV=3|e=2.38925E-13|from=1922|to=2560|frame=-1|Fragment_3
GPGWFRCECSRGFTGPDCRINVNECASSPCQGGAPC
>Locus_12205_Transcript_2/3_Confidence_0.571_Length_2677|.|sp|
P18168|SERR_DROME Protein serrate OS=Drosophila melanogaster GN=Ser
PE=1 SV=3|e=2.0851E-170|from=509|to=2674|frame=-1|Fragment_1
NGATCIDLVDDYHCACASGYTGRDCSQDVDECQTNPCRHGGECVDLVGGFRCICPVGRSG
TLCEDDIDRCAPNPCQNGAECLNTPGGYYCHCPGGLTGRHCEGPRHPVLA
>Locus_12205_Transcript_2/3_Confidence_0.571_Length_2677|.|sp|
P18168|SERR_DROME Protein serrate OS=Drosophila melanogaster GN=Ser
PE=1 SV=3|e=2.0851E-170|from=509|to=2674|frame=-1|Fragment_2
NDGCMPN
>Locus_12205_Transcript_2/3_Confidence_0.571_Length_2677|.|sp|
P18168|SERR_DROME Protein serrate OS=Drosophila melanogaster GN=Ser

PE=1 SV=3|e=2.0851E-170|from=509|to=2674|frame=-1|Fragment_3
PCQNNGTCLLGGPNGAAFTCLCSAGTRGRLC
>Locus_12205_Transcript_2/3_Confidence_0.571_Length_2677|.|sp|
P18168|SERR_DROME Protein serrate OS=Drosophila melanogaster GN=Ser
PE=1 SV=3|e=2.0851E-170|from=509|to=2674|frame=-1|Fragment_4
SEMSNSGVI
>Locus_12205_Transcript_2/3_Confidence_0.571_Length_2677|.|sp|
P18168|SERR_DROME Protein serrate OS=Drosophila melanogaster GN=Ser
PE=1 SV=3|e=2.0851E-170|from=509|to=2674|frame=-1|Fragment_5
GGSEDDCEGVT
>Locus_12205_Transcript_2/3_Confidence_0.571_Length_2677|.|sp|
P18168|SERR_DROME Protein serrate OS=Drosophila melanogaster GN=Ser
PE=1 SV=3|e=2.0851E-170|from=509|to=2674|frame=-1|Fragment_6
GRCLNGGIC
>Locus_12205_Transcript_2/3_Confidence_0.571_Length_2677|.|sp|
P18168|SERR_DROME Protein serrate OS=Drosophila melanogaster GN=Ser
PE=1 SV=3|e=2.0851E-170|from=509|to=2674|frame=-1|Fragment_7
DLQRGRCNCLAGWTGPQCEEPLDQCHGEPCHNGGTCVGGPGWFRCECSRGFTGPDCRINV
NECASSPCQGGAPCKDRIGGYTCLCPPGRAGERCEIETSG
>Locus_12205_Transcript_2/3_Confidence_0.571_Length_2677|.|sp|
P18168|SERR_DROME Protein serrate OS=Drosophila melanogaster GN=Ser
PE=1 SV=3|e=2.0851E-170|from=509|to=2674|frame=-1|Fragment_8
SGCWAPPNS
>Locus_12205_Transcript_2/3_Confidence_0.571_Length_2677|.|sp|
P18168|SERR_DROME Protein serrate OS=Drosophila melanogaster GN=Ser
PE=1 SV=3|e=2.0851E-170|from=509|to=2674|frame=-1|Fragment_9
TWLN
>Locus_12205_Transcript_2/3_Confidence_0.571_Length_2677|.|sp|
P18168|SERR_DROME Protein serrate OS=Drosophila melanogaster GN=Ser
PE=1 SV=3|e=2.0851E-170|from=509|to=2674|frame=-1|Fragment_10
ECNTCTCVQGIQRCSNLWCGLQDCLGVN
>Locus_12205_Transcript_2/3_Confidence_0.571_Length_2677|.|sp|
P18168|SERR_DROME Protein serrate OS=Drosophila melanogaster GN=Ser
PE=1 SV=3|e=2.0851E-170|from=509|to=2674|frame=-1|Fragment_11
STKCAPHEVCVPAPQESCLSSPCTARGDCRALEPSRRVAPPALPASPDCWPNQAALGEQC
ARVSALLETGKAPRGATSETLCDALRTLLGARLVREPTADLRH
>Locus_12205_Transcript_2/3_Confidence_0.571_Length_2677|.|sp|
P18168|SERR_DROME Protein serrate OS=Drosophila melanogaster GN=Ser
PE=1 SV=3|e=2.0851E-170|from=509|to=2674|frame=-1|Fragment_12
IVVVLCEMKTGSNDTVEVTVSSPPGGNAGAQIVALSVRLLGELISRGAAS
>Locus_12205_Transcript_2/3_Confidence_0.571_Length_2677|.|sp|
P18168|SERR_DROME Protein serrate OS=Drosophila melanogaster GN=Ser
PE=1 SV=3|e=2.0851E-170|from=509|to=2674|frame=-1|Fragment_13
SDGALPGAIREVKVETALLSSETQGGEYFIALICVMSIIVLAAVVLGIFVMRQRMRGRSI
SGMGLTPSPSDAFRAEEEKSNNLQNEENFRRYANPLKEE
>Locus_12205_Transcript_2/3_Confidence_0.571_Length_2677|.|sp|
P18168|SERR_DROME Protein serrate OS=Drosophila melanogaster GN=Ser
PE=1 SV=3|e=2.0851E-170|from=509|to=2674|frame=-1|Fragment_14
SIELSPKVSVEMHHHKSSQDLNPNVSLVRPIPGEALAGPSTTAQDFMHEYSQRLHKNKSL
NPDLVHVDADLLHVDCNLKTAH
>Locus_12205_Transcript_2/3_Confidence_0.571_Length_2677|.|sp|
P18168|SERR_DROME Protein serrate OS=Drosophila melanogaster GN=Ser
PE=1 SV=3|e=2.0851E-170|from=509|to=2674|frame=-1|Fragment_15
RNSQILLYKAQNPDMRKNTTGTFDNLNKDFGKRSINM

>Locus_12205_Transcript_2/3_Confidence_0.571_Length_2677|.|sp|
P18168|SERR_DROME Protein serrate OS=Drosophila melanogaster GN=Ser
PE=1 SV=3|e=2.0851E-170|from=509|to=2674|frame=-1|Fragment_16
STASSDSDVLTVLV
>Locus_12205_Transcript_2/3_Confidence_0.571_Length_2677|.|sp|
P18168|SERR_DROME Protein serrate OS=Drosophila melanogaster GN=Ser
PE=1 SV=3|e=3.33839E-43|from=1859|to=2653|frame=-1|Fragment_1
LVDDYHCACASGYTGRDCSQDVDECQTNPCRHGGECVDLVGGFRCICPVGRSGTLCEDDI
DRC
>Locus_12205_Transcript_2/3_Confidence_0.571_Length_2677|.|sp|
P18168|SERR_DROME Protein serrate OS=Drosophila melanogaster GN=Ser
PE=1 SV=3|e=3.33839E-43|from=1859|to=2653|frame=-1|Fragment_2
APNPCQNGAECLNTPGGYYCHCPGGLTGRHCEGPRHPVLANDGCMPNPCQNNGTCLLGGP
NGAAFTCLCSAGTRGRLCSEMSNSGVIGGSEDDCEGVTGRCLNGGIC
>Locus_12205_Transcript_2/3_Confidence_0.571_Length_2677|.|sp|
P18168|SERR_DROME Protein serrate OS=Drosophila melanogaster GN=Ser
PE=1 SV=3|e=3.33839E-43|from=1859|to=2653|frame=-1|Fragment_3
DLQRG
>Locus_12205_Transcript_2/3_Confidence_0.571_Length_2677|.|sp|
P18168|SERR_DROME Protein serrate OS=Drosophila melanogaster GN=Ser
PE=1 SV=3|e=3.33839E-43|from=1859|to=2653|frame=-1|Fragment_4
RCNCLAGWTGPQCEEPLDQCHGEPCHNGGTCVGGPGWFRCECSRGFTGPDCRINVNECAS
SPCQGGAPCKDRIGGYTCLCPPGRAGERCE
>Locus_12205_Transcript_2/3_Confidence_0.571_Length_2677|.|sp|
P18168|SERR_DROME Protein serrate OS=Drosophila melanogaster GN=Ser
PE=1 SV=3|e=1.77428E-36|from=1574|to=2674|frame=-1|Fragment_1
NGATCIDLVDDYHCACASGYTGRDCSQDVDECQT
>Locus_12205_Transcript_2/3_Confidence_0.571_Length_2677|.|sp|
P18168|SERR_DROME Protein serrate OS=Drosophila melanogaster GN=Ser
PE=1 SV=3|e=1.77428E-36|from=1574|to=2674|frame=-1|Fragment_2
NPCRHGGECVDLVGGFRCICPVGRSGTLCEDDIDRCAPNPCQNGAECLNTPGGYYCHCPG
GLTGRHCEGPRHPVLANDGCMPNPCQNNGTCLLGGPNGAAFTCLCSAGTRGRLCSEMSNS
GVIGGSEDDCEGVTGRCLNGGICDLQRG
>Locus_12205_Transcript_2/3_Confidence_0.571_Length_2677|.|sp|
P18168|SERR_DROME Protein serrate OS=Drosophila melanogaster GN=Ser
PE=1 SV=3|e=1.77428E-36|from=1574|to=2674|frame=-1|Fragment_3
RCNCLAGWTGPQCEEPLDQCHGEPCHNG
>Locus_12205_Transcript_2/3_Confidence_0.571_Length_2677|.|sp|
P18168|SERR_DROME Protein serrate OS=Drosophila melanogaster GN=Ser
PE=1 SV=3|e=1.77428E-36|from=1574|to=2674|frame=-1|Fragment_4
GTCVGGPGWFRCECSRGFTGPDCRINVNECASSPCQGGAPCKDRIGGYTCLCPPGRAGER
CEIETSGSGCWAPPNSTWLNEC
>Locus_12205_Transcript_2/3_Confidence_0.571_Length_2677|.|sp|
P18168|SERR_DROME Protein serrate OS=Drosophila melanogaster GN=Ser
PE=1 SV=3|e=1.77428E-36|from=1574|to=2674|frame=-1|Fragment_5
NTCTCVQGIQ
>Locus_12205_Transcript_2/3_Confidence_0.571_Length_2677|.|sp|
P18168|SERR_DROME Protein serrate OS=Drosophila melanogaster GN=Ser
PE=1 SV=3|e=1.77428E-36|from=1574|to=2674|frame=-1|Fragment_6
RCSNLWCGLQDCLGVNSTKCAPHEVCVPAPQESCLSSPCTARGDCRALEPSRRVAPPALP
ASPDC
>Locus_12205_Transcript_2/3_Confidence_0.571_Length_2677|.|sp|
P18168|SERR_DROME Protein serrate OS=Drosophila melanogaster GN=Ser
PE=1 SV=3|e=8.80565E-36|from=1655|to=2635|frame=-1|Fragment_1

CACASGYTGRDCSQDVDECQTNP
>Locus_12205_Transcript_2/3_Confidence_0.571_Length_2677|.|sp|
P18168|SERR_DROME Protein serrate OS=Drosophila melanogaster GN=Ser
PE=1 SV=3|e=8.80565E-36|from=1655|to=2635|frame=-1|Fragment_2
CRHGGECVDLVGGFRCICPVGRSGTLCEDDIDRCAPN
>Locus_12205_Transcript_2/3_Confidence_0.571_Length_2677|.|sp|
P18168|SERR_DROME Protein serrate OS=Drosophila melanogaster GN=Ser
PE=1 SV=3|e=8.80565E-36|from=1655|to=2635|frame=-1|Fragment_3
PCQNGAECLNT
>Locus_12205_Transcript_2/3_Confidence_0.571_Length_2677|.|sp|
P18168|SERR_DROME Protein serrate OS=Drosophila melanogaster GN=Ser
PE=1 SV=3|e=8.80565E-36|from=1655|to=2635|frame=-1|Fragment_4
PGGYYCHCPGGLTGRHCEGPRHPVLANDGCMPNPCQNNGTCLLGGPNGAAFTCLCSAGTR
GRLCSEMSNSGVIGGSEDDCEGVTGRCLNG
>Locus_12205_Transcript_2/3_Confidence_0.571_Length_2677|.|sp|
P18168|SERR_DROME Protein serrate OS=Drosophila melanogaster GN=Ser
PE=1 SV=3|e=8.80565E-36|from=1655|to=2635|frame=-1|Fragment_5
GICDLQRG
>Locus_12205_Transcript_2/3_Confidence_0.571_Length_2677|.|sp|
P18168|SERR_DROME Protein serrate OS=Drosophila melanogaster GN=Ser
PE=1 SV=3|e=8.80565E-36|from=1655|to=2635|frame=-1|Fragment_6
RCNCLAGWTGPQCEEPLDQCHGEPCHNGGTCVGGPGWFRCECSRGFTGPDCRINVNEC
>Locus_12205_Transcript_2/3_Confidence_0.571_Length_2677|.|sp|
P18168|SERR_DROME Protein serrate OS=Drosophila melanogaster GN=Ser
PE=1 SV=3|e=8.80565E-36|from=1655|to=2635|frame=-1|Fragment_7
ASSPCQGGAPCKDRIGGYTCLCPPGRAGERCEIETSGSGCWAPPNSTWLNECN
>Locus_12205_Transcript_2/3_Confidence_0.571_Length_2677|.|sp|
P18168|SERR_DROME Protein serrate OS=Drosophila melanogaster GN=Ser
PE=1 SV=3|e=8.80565E-36|from=1655|to=2635|frame=-1|Fragment_8
TCTCVQG
>Locus_12205_Transcript_2/3_Confidence_0.571_Length_2677|.|sp|
P18168|SERR_DROME Protein serrate OS=Drosophila melanogaster GN=Ser
PE=1 SV=3|e=8.80565E-36|from=1655|to=2635|frame=-1|Fragment_9
IQRCSNLWC
>Locus_12205_Transcript_2/3_Confidence_0.571_Length_2677|.|sp|
P18168|SERR_DROME Protein serrate OS=Drosophila melanogaster GN=Ser
PE=1 SV=3|e=8.80565E-36|from=1655|to=2635|frame=-1|Fragment_10
GLQDC
>Locus_12205_Transcript_2/3_Confidence_0.571_Length_2677|.|sp|
P18168|SERR_DROME Protein serrate OS=Drosophila melanogaster GN=Ser
PE=1 SV=3|e=8.80565E-36|from=1655|to=2635|frame=-1|Fragment_11
LGVNSTKC
>Locus_12205_Transcript_2/3_Confidence_0.571_Length_2677|.|sp|
P18168|SERR_DROME Protein serrate OS=Drosophila melanogaster GN=Ser
PE=1 SV=3|e=8.80565E-36|from=1655|to=2635|frame=-1|Fragment_12
APHEVCVPAP
>Locus_12205_Transcript_2/3_Confidence_0.571_Length_2677|.|sp|
P18168|SERR_DROME Protein serrate OS=Drosophila melanogaster GN=Ser
PE=1 SV=3|e=8.80565E-36|from=1655|to=2635|frame=-1|Fragment_13
QESCLSSP
>Locus_12205_Transcript_2/3_Confidence_0.571_Length_2677|.|sp|
P18168|SERR_DROME Protein serrate OS=Drosophila melanogaster GN=Ser
PE=1 SV=3|e=2.11052E-13|from=1922|to=2560|frame=-1|Fragment_1
HGGECVDLVGGFRCICPVGRSGTLCEDDIDRCAPNPCQNGAECLNTPGGYYCHCPGGLTG

RHCEGPRHPVLANDGCMPNPCQNNGTCLLGGPNGAAFTCLCSAGTRGRLCSEMSNSGVIG
GSEDDCEGVTGRCLNGGICDLQRGRCNCLAGWTGPQCEEPLDQCHG
>Locus_12205_Transcript_2/3_Confidence_0.571_Length_2677|.|sp|
P18168|SERR_DROME Protein serrate OS=Drosophila melanogaster GN=Ser
PE=1 SV=3|e=2.11052E-13|from=1922|to=2560|frame=-1|Fragment_2
EPCHNGGTCVG
>Locus_12205_Transcript_2/3_Confidence_0.571_Length_2677|.|sp|
P18168|SERR_DROME Protein serrate OS=Drosophila melanogaster GN=Ser
PE=1 SV=3|e=2.11052E-13|from=1922|to=2560|frame=-1|Fragment_3
GPGWFRCECSRGFTGPDCRINVNECASSPCQGGAPC

>Locus_621_Transcript_1/1_Confidence_1.000_Length_1216|UNIGENE
REPRESENTATIVE TRANSCRIPT|sp|P23380|VATL_DROME V-type proton ATPase
16 kDa proteolipid subunit OS=Drosophila melanogaster GN=Vha16-1
PE=2 SV=1|e=6.43404E-35|from=2|to=232|frame=2|Fragment_1
EEPKTYSLYKGFIHLGAGLAVGFSGLAAGFAIGIVGDAGVRGTAQQPRLFVGMILILIFA
EVLGLYGLIVAIYLYTK

>Locus_24204_Transcript_1/2_Confidence_0.750_Length_679|UNIGENE
REPRESENTATIVE TRANSCRIPT|sp|P25722|HS2ST_DROME Heparin sulfate O-
sulfotransferase OS=Drosophila melanogaster GN=Hs2st PE=2 SV=2|
e=2.47527E-73|from=1|to=555|frame=1|Fragment_1
KPLDRLVSYYYFLRNGDNFRPYLMRKKHGNKMTFDECVELKQPDCDPNNMWLQIPFLCGH
AAECWEPGSRWALEEAKRNLINNYFLVGLTEQLEDFIRILENSLPRIFKGATEYYQTSNK
SHLRQTVAKLEPMEKTVAAIKKSAIWQMENDLYQFAANHFAHVKKKFLTP
>Locus_24204_Transcript_1/2_Confidence_0.750_Length_679|UNIGENE
REPRESENTATIVE TRANSCRIPT|sp|P25722|HS2ST_DROME Heparin sulfate O-
sulfotransferase OS=Drosophila melanogaster GN=Hs2st PE=2 SV=2|
e=2.47527E-73|from=1|to=555|frame=1|Fragment_2
NGLPQTFFYEKIRPK
>Locus_24204_Transcript_2/2_Confidence_0.500_Length_559|.|sp|P25722|
HS2ST_DROME Heparin sulfate O-sulfotransferase OS=Drosophila
melanogaster GN=Hs2st PE=2 SV=2|e=8.88843E-54|from=1|to=435|frame=1|
Fragment_1
KQPDCDPNNMWLQIPFLCGHAAECWEPGSRWALEEAKRNLINNYFLVGLTEQLEDFIRIL
ENSLPRIFKGATEYYQTSNKSHLRQTVAKLEPMEKTVAAIKKSAIWQMENDLYQFAANHF
AHVKKKFLTP
>Locus_24204_Transcript_2/2_Confidence_0.500_Length_559|.|sp|P25722|
HS2ST_DROME Heparin sulfate O-sulfotransferase OS=Drosophila
melanogaster GN=Hs2st PE=2 SV=2|e=8.88843E-54|from=1|to=435|frame=1|
Fragment_2
NGLPQTFFYEKIRPK
>Locus_22709_Transcript_1/1_Confidence_1.000_Length_236|.|sp|P25722|
HS2ST_DROME Heparin sulfate O-sulfotransferase OS=Drosophila
melanogaster GN=Hs2st PE=2 SV=2|e=1.50874E-36|from=3|to=236|
frame=-1|Fragment_1
KPLDRLVSYYYFLRNGDNFRPYLMRKKHGNKMTFDECVELKQPDCDPNNMWLQIPFLCGH
AAECWEPGSRWALEEAKR

>Locus_5606_Transcript_1/4_Confidence_0.700_Length_721|UNIGENE
REPRESENTATIVE TRANSCRIPT|sp|P25931|NPYR_DROME Neuropeptide Y
receptor OS=Drosophila melanogaster GN=NepYr PE=2 SV=2|
e=2.56134E-71|from=116|to=721|frame=-1|Fragment_1
SVDRYMAIMWPLKPRLTKRSAKAIIVLVWAIAFLTALPIPAVSKLSQPAS

>Locus_5606_Transcript_1/4_Confidence_0.700_Length_721|UNIGENE REPRESENTATIVE TRANSCRIPT|sp|P25931|NPYR_DROME Neuropeptide Y receptor OS=Drosophila melanogaster GN=NepYr PE=2 SV=2|e=2.56134E-71|from=116|to=721|frame=-1|Fragment_2
WHQECNKVICREEWPSERHSYYYSMALMGLQFVAPLAVLMFTYTRIAIAVWGKRPPGEAV
DSRDQRMARSKR
>Locus_5606_Transcript_1/4_Confidence_0.700_Length_721|UNIGENE REPRESENTATIVE TRANSCRIPT|sp|P25931|NPYR_DROME Neuropeptide Y receptor OS=Drosophila melanogaster GN=NepYr PE=2 SV=2|e=2.56134E-71|from=116|to=721|frame=-1|Fragment_3
VCWMPFNILMVIWTNDESLGGWPPLPYVWFSFHWLAMSHSCYNPIIYCYMNARFRDGFAI
LLSPVP
>Locus_5605_Transcript_1/1_Confidence_1.000_Length_661|.|sp|P25931|NPYR_DROME Neuropeptide Y receptor OS=Drosophila melanogaster GN=NepYr PE=2 SV=2|e=1.12508E-51|from=207|to=659|frame=3|Fragment_1
SFNCDESFL
>Locus_5605_Transcript_1/1_Confidence_1.000_Length_661|.|sp|P25931|NPYR_DROME Neuropeptide Y receptor OS=Drosophila melanogaster GN=NepYr PE=2 SV=2|e=1.12508E-51|from=207|to=659|frame=3|Fragment_2
QQPLSSSYFRALVYFLYCSIFFLAVAGNGLVCFIVQRSPRMRTVTNLFIVNLAVGDILMS
VFCIPFTFVSILILHHWPFGMIMCHLVNFSQAVSVLVSAYTLVAISVDRYMAIMWPLKPR
LTKRSAKAIIVLVWAIAFLTAL
>Locus_5606_Transcript_2/4_Confidence_0.600_Length_611|.|sp|P25931|NPYR_DROME Neuropeptide Y receptor OS=Drosophila melanogaster GN=NepYr PE=2 SV=2|e=1.89475E-71|from=6|to=611|frame=-1|Fragment_1
SVDRYMAIMWPLKPRLTKRSAKAIIVLVWAIAFLTALPIPAVSKLSQPAS
>Locus_5606_Transcript_2/4_Confidence_0.600_Length_611|.|sp|P25931|NPYR_DROME Neuropeptide Y receptor OS=Drosophila melanogaster GN=NepYr PE=2 SV=2|e=1.89475E-71|from=6|to=611|frame=-1|Fragment_2
WHQECNKVICREEWPSERHSYYYSMALMGLQFVAPLAVLMFTYTRIAIAVWGKRPPGEAV
DSRDQRMARSKR
>Locus_5606_Transcript_2/4_Confidence_0.600_Length_611|.|sp|P25931|NPYR_DROME Neuropeptide Y receptor OS=Drosophila melanogaster GN=NepYr PE=2 SV=2|e=1.89475E-71|from=6|to=611|frame=-1|Fragment_3
VCWMPFNILMVIWTNDESLGGWPPLPYVWFSFHWLAMSHSCYNPIIYCYMNARFRDGFAI
LLSPVP
>Locus_5606_Transcript_3/4_Confidence_0.600_Length_611|.|sp|P25931|NPYR_DROME Neuropeptide Y receptor OS=Drosophila melanogaster GN=NepYr PE=2 SV=2|e=1.89475E-71|from=6|to=611|frame=-1|Fragment_1
SVDRYMAIMWPLKPRLTKRSAKAIIVLVWAIAFLTALPIPAVSKLSQPAS
>Locus_5606_Transcript_3/4_Confidence_0.600_Length_611|.|sp|P25931|NPYR_DROME Neuropeptide Y receptor OS=Drosophila melanogaster GN=NepYr PE=2 SV=2|e=1.89475E-71|from=6|to=611|frame=-1|Fragment_2
WHQECNKVICREEWPSERHSYYYSMALMGLQFVAPLAVLMFTYTRIAIAVWGKRPPGEAV
DSRDQRMARSKR
>Locus_5606_Transcript_3/4_Confidence_0.600_Length_611|.|sp|P25931|NPYR_DROME Neuropeptide Y receptor OS=Drosophila melanogaster GN=NepYr PE=2 SV=2|e=1.89475E-71|from=6|to=611|frame=-1|Fragment_3
VCWMPFNILMVIWTNDESLGGWPPLPYVWFSFHWLAMSHSCYNPIIYCYMNARFRDGFAI
LLSPVP
>Locus_5606_Transcript_4/4_Confidence_0.600_Length_611|.|sp|P25931|NPYR_DROME Neuropeptide Y receptor OS=Drosophila melanogaster GN=NepYr PE=2 SV=2|e=1.89475E-71|from=6|to=611|frame=-1|Fragment_1
SVDRYMAIMWPLKPRLTKRSAKAIIVLVWAIAFLTALPIPAVSKLSQPAS

>Locus_5606_Transcript_4/4_Confidence_0.600_Length_611|.|sp|P25931|
NPYR_DROME Neuropeptide Y receptor OS=Drosophila melanogaster
GN=NepYr PE=2 SV=2|e=1.89475E-71|from=6|to=611|frame=-1|Fragment_2
WHQECNKVICREEWPSERHSYYYSMALMGLQFVAPLAVLMFTYTRIAIAVWGKRPPGEAV
DSRDQRMARSKR
>Locus_5606_Transcript_4/4_Confidence_0.600_Length_611|.|sp|P25931|
NPYR_DROME Neuropeptide Y receptor OS=Drosophila melanogaster
GN=NepYr PE=2 SV=2|e=1.89475E-71|from=6|to=611|frame=-1|Fragment_3
VCWMPFNILMVIWTNDESLGGWPPLPYVWFSFHWLAMSHSCYNPIIYCYMNARFRDGFAI
LLSPVP
>Locus_14165_Transcript_3/5_Confidence_0.417_Length_437|.|sp|P25931|
NPYR_DROME Neuropeptide Y receptor OS=Drosophila melanogaster
GN=NepYr PE=2 SV=2|e=1.06637E-48|from=16|to=432|frame=1|Fragment_1
SFNCDESFL
>Locus_14165_Transcript_3/5_Confidence_0.417_Length_437|.|sp|P25931|
NPYR_DROME Neuropeptide Y receptor OS=Drosophila melanogaster
GN=NepYr PE=2 SV=2|e=1.06637E-48|from=16|to=432|frame=1|Fragment_2
QQPLSSSYFRALVYFLYCSIFFLAVAGNGLVCFIVQRSPRMRTVTNLFIVNLAVGDILMS
VFCIPFTFVSILILHHWPFGMIMCHLVNFSQAVSVLVSAYTLVAISVDRYMAIMWPLKPR
LTKRSAKAII
>Locus_14165_Transcript_2/5_Confidence_0.417_Length_379|.|sp|P25931|
NPYR_DROME Neuropeptide Y receptor OS=Drosophila melanogaster
GN=NepYr PE=2 SV=2|e=9.87922E-20|from=144|to=377|frame=3|Fragment_1
SFNCDESFL
>Locus_14165_Transcript_2/5_Confidence_0.417_Length_379|.|sp|P25931|
NPYR_DROME Neuropeptide Y receptor OS=Drosophila melanogaster
GN=NepYr PE=2 SV=2|e=9.87922E-20|from=144|to=377|frame=3|Fragment_2
QQPLSSSYFRALVYFLYCSIFFLAVAGNGLVCFIVQRSPRMRTVTNLFIVNLAVGDILMS
VFCIPFTFV
>Locus_14165_Transcript_4/5_Confidence_0.417_Length_379|.|sp|P25931|
NPYR_DROME Neuropeptide Y receptor OS=Drosophila melanogaster
GN=NepYr PE=2 SV=2|e=9.87922E-20|from=144|to=377|frame=3|Fragment_1
SFNCDESFL
>Locus_14165_Transcript_4/5_Confidence_0.417_Length_379|.|sp|P25931|
NPYR_DROME Neuropeptide Y receptor OS=Drosophila melanogaster
GN=NepYr PE=2 SV=2|e=9.87922E-20|from=144|to=377|frame=3|Fragment_2
QQPLSSSYFRALVYFLYCSIFFLAVAGNGLVCFIVQRSPRMRTVTNLFIVNLAVGDILMS
VFCIPFTFV
>Locus_11928_Transcript_1/1_Confidence_1.000_Length_281|.|sp|P25931|
NPYR_DROME Neuropeptide Y receptor OS=Drosophila melanogaster
GN=NepYr PE=2 SV=2|e=4.36867E-36|from=6|to=281|frame=-1|Fragment_1
VQRSPRMRTVTNLFIVNLAVGDILMSVFCIPFTFVSILILHHWPFGMIMCHLVNFSQAVS
VLVSAYTLVAISVDRYMAIMWPLKPRLTKRSA
>Locus_30558_Transcript_1/1_Confidence_1.000_Length_281|.|sp|P25931|
NPYR_DROME Neuropeptide Y receptor OS=Drosophila melanogaster
GN=NepYr PE=2 SV=2|e=4.36867E-36|from=6|to=281|frame=-1|Fragment_1
VQRSPRMRTVTNLFIVNLAVGDILMSVFCIPFTFVSILILHHWPFGMIMCHLVNFSQAVS
VLVSAYTLVAISVDRYMAIMWPLKPRLTKRSA
>Locus_14165_Transcript_5/5_Confidence_0.333_Length_251|.|sp|P25931|
NPYR_DROME Neuropeptide Y receptor OS=Drosophila melanogaster
GN=NepYr PE=2 SV=2|e=1.0062E-19|from=16|to=249|frame=1|Fragment_1
SFNCDESFL
>Locus_14165_Transcript_5/5_Confidence_0.333_Length_251|.|sp|P25931|
NPYR_DROME Neuropeptide Y receptor OS=Drosophila melanogaster

GN=NepYr PE=2 SV=2|e=1.0062E-19|from=16|to=249|frame=1|Fragment_2
QQPLSSSYFRALVYFLYCSIFFLAVAGNGLVCFIVQRSPRMRTVTNLFIVNLAVGDILMS
VFCIPFTFV
>Locus_25112_Transcript_1/1_Confidence_1.000_Length_461|UNIGENE
REPRESENTATIVE TRANSCRIPT|sp|P27716|INX1_DROME Innexin inx1
OS=Drosophila melanogaster GN=ogre PE=1 SV=1|e=1.26222E-69|from=21|
to=461|frame=3|Fragment_1
MYRLLGGLKDYFKVQDIKTDNAVFRLHNLFTTAILLTCSMIITATQFVGNPIQCIVNGIP
VHVVNTFCWVTSTFTMPDAHKRQVGLEVVQPGVGNDFGDEDAKKYYTYYQWVCFVLFFQA
IMCYTPKFLWDSFEGGLMRTIVMGLNV
>Locus_23801_Transcript_1/1_Confidence_1.000_Length_252|.|sp|P27716|
INX1_DROME Innexin inx1 OS=Drosophila melanogaster GN=ogre PE=1
SV=1|e=1.31346E-11|from=115|to=252|frame=-1|Fragment_1
AISRKTDVGDWWIIYMLGRNMDPLIYREVIAELAKKIETNSKETRA

>Locus_30475_Transcript_1/1_Confidence_1.000_Length_242|UNIGENE
REPRESENTATIVE TRANSCRIPT|"sp|P29993|ITPR_DROME Inositol 1,4,5-
trisphosphate receptor OS=Drosophila melanogaster GN=Itp-r83A PE=2
SV=3"|e=1.35697E-37|from=3|to=239|frame=-1|Fragment_1
TTSFLHLGDIVSLYAEGNVCGFLSTLGLVDDRTVVCPEAGDLGNPPKKFRDCLFKICPMN
RYSAQKQFWKAAKQSASAN

>Locus_6988_Transcript_1/6_Confidence_0.615_Length_3850|UNIGENE
REPRESENTATIVE TRANSCRIPT|"sp|P30430|FUR1C_DROME Furin-like protease
1, isoform 1-CRR OS=Drosophila melanogaster GN=Fur1 PE=2 SV=2"|e=0|
from=1976|to=3844|frame=-1|Fragment_1
ANLVARDHGFINLGEIFNDHYHFHHRAVPKRSKRSCDHRHRRLQSDSRVHWARQQRVKSR
QKRDLIALRP
>Locus_6988_Transcript_1/6_Confidence_0.615_Length_3850|UNIGENE
REPRESENTATIVE TRANSCRIPT|"sp|P30430|FUR1C_DROME Furin-like protease
1, isoform 1-CRR OS=Drosophila melanogaster GN=Fur1 PE=2 SV=2"|e=0|
from=1976|to=3844|frame=-1|Fragment_2
RTNLRT
>Locus_6988_Transcript_1/6_Confidence_0.615_Length_3850|UNIGENE
REPRESENTATIVE TRANSCRIPT|"sp|P30430|FUR1C_DROME Furin-like protease
1, isoform 1-CRR OS=Drosophila melanogaster GN=Fur1 PE=2 SV=2"|e=0|
from=1976|to=3844|frame=-1|Fragment_3
GIALNDQKWPQMWYLNRGSGLDMNVIPAWKEGITGKGVVVTILDDGLEKNHPDLIRNYDP
QASYDVNSHDEDPMPRYDMIDSNRHGTRCAGEVAATANNSLC
>Locus_6988_Transcript_1/6_Confidence_0.615_Length_3850|UNIGENE
REPRESENTATIVE TRANSCRIPT|"sp|P30430|FUR1C_DROME Furin-like protease
1, isoform 1-CRR OS=Drosophila melanogaster GN=Fur1 PE=2 SV=2"|e=0|
from=1976|to=3844|frame=-1|Fragment_4
VRMLDGDVTDAVEARSLSLNPQHIDIYSASWGPDDDGKTVDGPGELATRAFIEGVTKGRS
GKGSIFVWASGNGGREHDNCNCDGYTNSIWTLSISSATERGLVPWYSEMCSSTLATTYSS
GAQNEKQVITTDLHHACTSSHTGTSASAPLAAGICALALEANRDLTWRDMQHIVVRTAKP
ANLKSDDWATNGVGRNVSHSFGYGLMDAAAMVKLARTWKTVPEQRRCEVSAPHIDKLIPP
KSKVILQLPVSLCQGVNFLEHVQAKISLQASRRGDIQIHLTSPGGTRVTLLAARQNDASR
SGFSNWPFMSVHTWGESPQGLWHLEVHNEGRYMAKLTQWDLLFHGTQEPPQESDVE
>Locus_6988_Transcript_1/6_Confidence_0.615_Length_3850|UNIGENE
REPRESENTATIVE TRANSCRIPT|"sp|P30430|FUR1C_DROME Furin-like protease
1, isoform 1-CRR OS=Drosophila melanogaster GN=Fur1 PE=2 SV=2"|e=0|
from=1976|to=3844|frame=-1|Fragment_5
SVQYSANGGSNYPDGLEHNSLETDTASGQWRDLHHVREGHIDVQRTASDDVTSSACISYN

DNHNCL
>Locus_6988_Transcript_2/6_Confidence_0.615_Length_3850|.|"sp|
P30430|FUR1C_DROME Furin-like protease 1, isoform 1-CRR
OS=Drosophila melanogaster GN=Fur1 PE=2 SV=2"|e=0|from=1976|to=3844|
frame=-1|Fragment_1
ANLVARDHGFINLGEIFNDHYHFHHRAVPRRSKRSCDHRHRRLQSDSRVHWARQQRVKSR
QKRDLIALRP
>Locus_6988_Transcript_2/6_Confidence_0.615_Length_3850|.|"sp|
P30430|FUR1C_DROME Furin-like protease 1, isoform 1-CRR
OS=Drosophila melanogaster GN=Fur1 PE=2 SV=2"|e=0|from=1976|to=3844|
frame=-1|Fragment_2
RTNLRT
>Locus_6988_Transcript_2/6_Confidence_0.615_Length_3850|.|"sp|
P30430|FUR1C_DROME Furin-like protease 1, isoform 1-CRR
OS=Drosophila melanogaster GN=Fur1 PE=2 SV=2"|e=0|from=1976|to=3844|
frame=-1|Fragment_3
GIALNDQKWPQMWYLNRGSGLDMNVIPAWKEGITGKGVVVTILDDGLEKNHPDLIRNYDP
QASYDVNSHDEDPMPRYDMIDSNRHGTRCAGEVAATANNSLC
>Locus_6988_Transcript_2/6_Confidence_0.615_Length_3850|.|"sp|
P30430|FUR1C_DROME Furin-like protease 1, isoform 1-CRR
OS=Drosophila melanogaster GN=Fur1 PE=2 SV=2"|e=0|from=1976|to=3844|
frame=-1|Fragment_4
VRMLDGDVTDAVEARSLSLNPQHIDIYSASWGPDDDGKTVDGPGELATRAFIEGVTKGRS
GKGSIFVWASGNGGREHDNCNCDGYTNSIWTLSISSATERGLVPWYSEMCSSTLATTYSS
GAQNEKQVITTDLHHACTSSHTGTSASAPLAAGICALALEANRDLTWRDMQHIVVRTAKP
ANLKSDDWATNGVGRNVSHSFGYGLMDAAAMVKLARTWKTVPEQRRCEVSAPHIDKLIPP
KSKVILQLPVSLCQGVNFLEHVQAKISLQASRRGDIQIHLTSPGGTRVTLLAARQNDASR
SGFSNWPFMSVHTWGESPQGLWHLEVHNEGRYMAKLTQWDLLFHGTQEPPQESDVE
>Locus_6988_Transcript_2/6_Confidence_0.615_Length_3850|.|"sp|
P30430|FUR1C_DROME Furin-like protease 1, isoform 1-CRR
OS=Drosophila melanogaster GN=Fur1 PE=2 SV=2"|e=0|from=1976|to=3844|
frame=-1|Fragment_5
SVQYSANGGSNYPDGLEHNSLETDTASGQWRDLHHVREGHIDVQRTASDDVTSSACISYN
DNHNCL
>Locus_6988_Transcript_3/6_Confidence_0.615_Length_3850|.|"sp|
P30430|FUR1C_DROME Furin-like protease 1, isoform 1-CRR
OS=Drosophila melanogaster GN=Fur1 PE=2 SV=2"|e=0|from=1976|to=3844|
frame=-1|Fragment_1
ANLVARDHGFINLGEIFNDHYHFHHRAVPKRSKRSCDHRHRRLQSDSRVHWARQQRVKSR
QKRDLIALRP
>Locus_6988_Transcript_3/6_Confidence_0.615_Length_3850|.|"sp|
P30430|FUR1C_DROME Furin-like protease 1, isoform 1-CRR
OS=Drosophila melanogaster GN=Fur1 PE=2 SV=2"|e=0|from=1976|to=3844|
frame=-1|Fragment_2
RTNLRT
>Locus_6988_Transcript_3/6_Confidence_0.615_Length_3850|.|"sp|
P30430|FUR1C_DROME Furin-like protease 1, isoform 1-CRR
OS=Drosophila melanogaster GN=Fur1 PE=2 SV=2"|e=0|from=1976|to=3844|
frame=-1|Fragment_3
GIALNDQKWPQMWYLNRGSGLDMNVIPAWKEGITGKGVVVTILDDGLEKNHPDLIRNYDP
QASYDVNSHDEDPMPRYDMIDSNRHGTRCAGEVAATANNSLC
>Locus_6988_Transcript_3/6_Confidence_0.615_Length_3850|.|"sp|
P30430|FUR1C_DROME Furin-like protease 1, isoform 1-CRR
OS=Drosophila melanogaster GN=Fur1 PE=2 SV=2"|e=0|from=1976|to=3844|

frame=-1|Fragment_4
VRMLDGDVTDAVEARSLSLNPQHIDIYSASWGPDDDGKTVDGPGELATRAFIEGVTKGRS
GKGSIFVWASGNGGREHDNCNCDGYTNSIWTLSISSATERGLVPWYSEMCSSTLATTYSS
GAQNEKQVITTDLHHACTSSHTGTSASAPLAAGICALALEANRDLTWRDMQHIVVRTAKP
ANLKSDDWATNGVGRNVSHSFGYGLMDAAAMVKLARTWKTVPEQRRCEVSAPHIDKLIPP
KSKVILQLPVSLCQGVNFLEHVQAKISLQASRRGDIQIHLTSPGGTRVTLLAARQNDASR
SGFSNWPFMSVHTWGESPQGLWHLEVHNEGRYMAKLTQWDLLFHGTQEPPQESDVE
>Locus_6988_Transcript_3/6_Confidence_0.615_Length_3850|.|"sp|
P30430|FUR1C_DROME Furin-like protease 1, isoform 1-CRR
OS=Drosophila melanogaster GN=Fur1 PE=2 SV=2"|e=0|from=1976|to=3844|
frame=-1|Fragment_5
SVQYSANGGSNYPDGLEHNSLETDTASGQWRDLHHVREGHIDVQRTASDDVTSSACISYN
DNHNCL
>Locus_6988_Transcript_6/6_Confidence_0.615_Length_3850|.|"sp|
P30430|FUR1C_DROME Furin-like protease 1, isoform 1-CRR
OS=Drosophila melanogaster GN=Fur1 PE=2 SV=2"|e=0|from=1976|to=3844|
frame=-1|Fragment_1
ANLVARDHGFINLGEIFNDHYHFHHRAVPKRSKRSCDHRHRRLQSDSRVHWARQQRVKSR
QKRDLIALRP
>Locus_6988_Transcript_6/6_Confidence_0.615_Length_3850|.|"sp|
P30430|FUR1C_DROME Furin-like protease 1, isoform 1-CRR
OS=Drosophila melanogaster GN=Fur1 PE=2 SV=2"|e=0|from=1976|to=3844|
frame=-1|Fragment_2
RTNLRT
>Locus_6988_Transcript_6/6_Confidence_0.615_Length_3850|.|"sp|
P30430|FUR1C_DROME Furin-like protease 1, isoform 1-CRR
OS=Drosophila melanogaster GN=Fur1 PE=2 SV=2"|e=0|from=1976|to=3844|
frame=-1|Fragment_3
GIALNDQKWPQMWYLNRGSGLDMNVIPAWKEGITGKGVVVTILDDGLEKNHPDLIRNYDP
QASYDVNSHDEDPMPRYDMIDSNRHGTRCAGEVAATANNSLC
>Locus_6988_Transcript_6/6_Confidence_0.615_Length_3850|.|"sp|
P30430|FUR1C_DROME Furin-like protease 1, isoform 1-CRR
OS=Drosophila melanogaster GN=Fur1 PE=2 SV=2"|e=0|from=1976|to=3844|
frame=-1|Fragment_4
VRMLDGDVTDAVEARSLSLNPQHIDIYSASWGPDDDGKTVDGPGELATRAFIEGVTKGRS
GKGSIFVWASGNGGREHDNCNCDGYTNSIWTLSISSATERGLVPWYSEMCSSTLATTYSS
GAQNEKQVITTDLHHACTSSHTGTSASAPLAAGICALALEANRDLTWRDMQHIVVRTAKP
ANLKSDDWATNGVGRNVSHSFGYGLMDAAAMVKLARTWKTVPEQRRCEVSAPHIDKLIPP
KSKVILQLPVSLCQGVNFLEHVQAKISLQASRRGDIQIHLTSPGGTRVTLLAARQNDASR
SGFSNWPFMSVHTWGESPQGLWHLEVHNEGRYMAKLTQWDLLFHGTQEPPQESDVE
>Locus_6988_Transcript_6/6_Confidence_0.615_Length_3850|.|"sp|
P30430|FUR1C_DROME Furin-like protease 1, isoform 1-CRR
OS=Drosophila melanogaster GN=Fur1 PE=2 SV=2"|e=0|from=1976|to=3844|
frame=-1|Fragment_5
SVQYSANGGSNYPDGLEHNSLETDTASGQWRDLHHVREGHIDVQRTASDDVTSSACISYN
DNHNCL
>Locus_6988_Transcript_5/6_Confidence_0.615_Length_3793|.|"sp|
P30430|FUR1C_DROME Furin-like protease 1, isoform 1-CRR
OS=Drosophila melanogaster GN=Fur1 PE=2 SV=2"|e=0|from=1919|to=3787|
frame=-1|Fragment_1
ANLVARDHGFINLGEIFNDHYHFHHRAVPKRSKRSCDHRHRRLQSDSRVHWARQQRVKSR
QKRDLIALRP
>Locus_6988_Transcript_5/6_Confidence_0.615_Length_3793|.|"sp|
P30430|FUR1C_DROME Furin-like protease 1, isoform 1-CRR

OS=Drosophila melanogaster GN=Fur1 PE=2 SV=2"|e=0|from=1919|to=3787|
frame=-1|Fragment_2
RTNLRT
>Locus_6988_Transcript_5/6_Confidence_0.615_Length_3793|.|"sp|
P30430|FUR1C_DROME Furin-like protease 1, isoform 1-CRR
OS=Drosophila melanogaster GN=Fur1 PE=2 SV=2"|e=0|from=1919|to=3787|
frame=-1|Fragment_3
GIALNDQKWPQMWYLNRGSGLDMNVIPAWKEGITGKGVVVTILDDGLEKNHPDLIRNYDP
QASYDVNSHDEDPMPRYDMIDSNRHGTRCAGEVAATANNSLC
>Locus_6988_Transcript_5/6_Confidence_0.615_Length_3793|.|"sp|
P30430|FUR1C_DROME Furin-like protease 1, isoform 1-CRR
OS=Drosophila melanogaster GN=Fur1 PE=2 SV=2"|e=0|from=1919|to=3787|
frame=-1|Fragment_4
VRMLDGDVTDAVEARSLSLNPQHIDIYSASWGPDDDGKTVDGPGELATRAFIEGVTKGRS
GKGSIFVWASGNGGREHDNCNCDGYTNSIWTLSISSATERGLVPWYSEMCSSTLATTYSS
GAQNEKQVITTDLHHACTSSHTGTSASAPLAAGICALALEANRDLTWRDMQHIVVRTAKP
ANLKSDDWATNGVGRNVSHSFGYGLMDAAAMVKLARTWKTVPEQRRCEVSAPHIDKLIPP
KSKVILQLPVSLCQGVNFLEHVQAKISLQASRRGDIQIHLTSPGGTRVTLLAARQNDASR
SGFSNWPFMSVHTWGESPQGLWHLEVHNEGRYMAKLTQWDLLFHGTQEPPQESDVE
>Locus_6988_Transcript_5/6_Confidence_0.615_Length_3793|.|"sp|
P30430|FUR1C_DROME Furin-like protease 1, isoform 1-CRR
OS=Drosophila melanogaster GN=Fur1 PE=2 SV=2"|e=0|from=1919|to=3787|
frame=-1|Fragment_5
SVQYSANGGSNYPDGLEHNSLETDTASGQWRDLHHVREGHIDVQRTASDDVTSSACISYN
DNHNCL
>Locus_6988_Transcript_4/6_Confidence_0.538_Length_2275|.|"sp|
P30430|FUR1C_DROME Furin-like protease 1, isoform 1-CRR
OS=Drosophila melanogaster GN=Fur1 PE=2 SV=2"|e=0|from=56|to=2269|
frame=-1|Fragment_1
ANLVARDHGFINLGEIFNDHYHFHHRAVPKRSKRSCDHRHRRLQSDSRVHWARQQRVKSR
QKRDLIALRP
>Locus_6988_Transcript_4/6_Confidence_0.538_Length_2275|.|"sp|
P30430|FUR1C_DROME Furin-like protease 1, isoform 1-CRR
OS=Drosophila melanogaster GN=Fur1 PE=2 SV=2"|e=0|from=56|to=2269|
frame=-1|Fragment_2
RTNLRT
>Locus_6988_Transcript_4/6_Confidence_0.538_Length_2275|.|"sp|
P30430|FUR1C_DROME Furin-like protease 1, isoform 1-CRR
OS=Drosophila melanogaster GN=Fur1 PE=2 SV=2"|e=0|from=56|to=2269|
frame=-1|Fragment_3
GIALNDQKWPQMWYLNRGSGLDMNVIPAWKEGITGKGVVVTILDDGLEKNHPDLIRNYDP
QASYDVNSHDEDPMPRYDMIDSNRHGTRCAGEVAATANNSLC
>Locus_6988_Transcript_4/6_Confidence_0.538_Length_2275|.|"sp|
P30430|FUR1C_DROME Furin-like protease 1, isoform 1-CRR
OS=Drosophila melanogaster GN=Fur1 PE=2 SV=2"|e=0|from=56|to=2269|
frame=-1|Fragment_4
VRMLDGDVTDAVEARSLSLNPQHIDIYSASWGPDDDGKTVDGPGELATRAFIEGVTKGRS
GKGSIFVWASGNGGREHDNCNCDGYTNSIWTLSISSATERGLVPWYSEMCSSTLATTYSS
GAQNEKQVITTDLHHACTSSHTGTSASAPLAAGICALALEANRDLTWRDMQHIVVRTAKP
ANLKSDDWATNGVGRNVSHSFGYGLMDAAAMVKLARTWKTVPEQRRCEVSAPHIDKLIPP
KSKVILQLPVSLCQGVNFLEHVQAKISLQASRRGDIQIHLTSPGGTRVTLLAARQNDASR
SGFSNWPFMSVHTWGESPQGLWHLEVHNEGRYMAKLTQWDLLFHGTQEPPQESDVE
>Locus_6988_Transcript_4/6_Confidence_0.538_Length_2275|.|"sp|
P30430|FUR1C_DROME Furin-like protease 1, isoform 1-CRR

OS=Drosophila melanogaster GN=Fur1 PE=2 SV=2"|e=0|from=56|to=2269|
frame=-1|Fragment_5
SVQYSANGGSNYPDGLEHNSLETDTASGQWRDLHHVREGHIDVQRTASDDVTSSACISYN
DNHNCLECMPDTYLFEGHCYTNCPAKSFLSAK
>Locus_6988_Transcript_4/6_Confidence_0.538_Length_2275|.|"sp|
P30430|FUR1C_DROME Furin-like protease 1, isoform 1-CRR
OS=Drosophila melanogaster GN=Fur1 PE=2 SV=2"|e=0|from=56|to=2269|
frame=-1|Fragment_6
PTWLPATRNHNKKHLLENLWSSLAARNG
>Locus_6988_Transcript_4/6_Confidence_0.538_Length_2275|.|"sp|
P30430|FUR1C_DROME Furin-like protease 1, isoform 1-CRR
OS=Drosophila melanogaster GN=Fur1 PE=2 SV=2"|e=0|from=56|to=2269|
frame=-1|Fragment_7
HRVQRQVEEINRNSDM
>Locus_6988_Transcript_4/6_Confidence_0.538_Length_2275|.|"sp|
P30430|FUR1C_DROME Furin-like protease 1, isoform 1-CRR
OS=Drosophila melanogaster GN=Fur1 PE=2 SV=2"|e=0|from=56|to=2269|
frame=-1|Fragment_8
MVCLHCHYSCLTCRGPNDHECSTCNEDAVLTNKTNNENFCYPLTI

>Locus_4010_Transcript_1/3_Confidence_0.714_Length_4138|UNIGENE
REPRESENTATIVE TRANSCRIPT|sp|P30432|FUR2_DROME Furin-like protease 2
OS=Drosophila melanogaster GN=Fur2 PE=2 SV=2|e=0|from=72|to=3968|
frame=3|Fragment_1
IYGNEFAVHVPSGVEAADAIAVKHGFVNMGQIGSLKGYYLFQHNHVRKRSLSMSTEHHET
LNSEPEVYWVQQQHEKVRRKRDYGTIYNGQFSPLDTASELDALLLGG
>Locus_4010_Transcript_1/3_Confidence_0.714_Length_4138|UNIGENE
REPRESENTATIVE TRANSCRIPT|sp|P30432|FUR2_DROME Furin-like protease 2
OS=Drosophila melanogaster GN=Fur2 PE=2 SV=2|e=0|from=72|to=3968|
frame=3|Fragment_2
VAPSASRL
>Locus_4010_Transcript_1/3_Confidence_0.714_Length_4138|UNIGENE
REPRESENTATIVE TRANSCRIPT|sp|P30432|FUR2_DROME Furin-like protease 2
OS=Drosophila melanogaster GN=Fur2 PE=2 SV=2|e=0|from=72|to=3968|
frame=3|Fragment_3
HTARYRALGVGTQSIFPDPLFKEEWYLNGGAKDGLDMNVSPAWQKGYTGKGVVVSILDDG
IQTNHPDLALNYDPAAST
>Locus_4010_Transcript_1/3_Confidence_0.714_Length_4138|UNIGENE
REPRESENTATIVE TRANSCRIPT|sp|P30432|FUR2_DROME Furin-like protease 2
OS=Drosophila melanogaster GN=Fur2 PE=2 SV=2|e=0|from=72|to=3968|
frame=3|Fragment_4
KHGTRCAGEVAAVAYNNYCGIGVAYNASIGGVRMLDGVVNDAVEARALGLNPDHIDVYSA
SWGPEDDGKTVDGPGPLARRAFIYGVTSGRKGKGSIFVWASGNGGRHTDSCNCDGYTNSI
FTLSISSATQGGYKPWYLEECSSTLASTYSSGTPGHDKSVATVDMDARLRPDHICTVEHT
GTSASAPLAAGICALALEANPALTWRDMQYLVVLTSRSAPLEKEAGWILNGVKRKVSHKF
GYGLMDAGAMVSLAEQWTTVPPQHICKSQEIAEERLIEPTYGNTLVAHMDVTGCSGTLNE
VRFLEHVQCKITLRFFPRGNLKILLTSPMGTTSTLLFERPRDVVSSNFDDWPFLSVHFWG
EKADGRWTLQVINAGNRRVNQAGILKKWQLIFYGTNTNPIR
>Locus_4010_Transcript_1/3_Confidence_0.714_Length_4138|UNIGENE
REPRESENTATIVE TRANSCRIPT|sp|P30432|FUR2_DROME Furin-like protease 2
OS=Drosophila melanogaster GN=Fur2 PE=2 SV=2|e=0|from=72|to=3968|
frame=3|Fragment_5
LRSPNPS
>Locus_4010_Transcript_1/3_Confidence_0.714_Length_4138|UNIGENE

REPRESENTATIVE TRANSCRIPT|sp|P30432|FUR2_DROME Furin-like protease 2
OS=Drosophila melanogaster GN=Fur2 PE=2 SV=2|e=0|from=72|to=3968|
frame=3|Fragment_6
FQFPGQDALRRVVPQPPQYFTDFNT
>Locus_4010_Transcript_1/3_Confidence_0.714_Length_4138|UNIGENE
REPRESENTATIVE TRANSCRIPT|sp|P30432|FUR2_DROME Furin-like protease 2
OS=Drosophila melanogaster GN=Fur2 PE=2 SV=2|e=0|from=72|to=3968|
frame=3|Fragment_7
GTYRNYPDLF
>Locus_4010_Transcript_1/3_Confidence_0.714_Length_4138|UNIGENE
REPRESENTATIVE TRANSCRIPT|sp|P30432|FUR2_DROME Furin-like protease 2
OS=Drosophila melanogaster GN=Fur2 PE=2 SV=2|e=0|from=72|to=3968|
frame=3|Fragment_8
GAGSDPEVAIVSPD
>Locus_4010_Transcript_1/3_Confidence_0.714_Length_4138|UNIGENE
REPRESENTATIVE TRANSCRIPT|sp|P30432|FUR2_DROME Furin-like protease 2
OS=Drosophila melanogaster GN=Fur2 PE=2 SV=2|e=0|from=72|to=3968|
frame=3|Fragment_9
DGMLAQASATNVVVNNKT
>Locus_4010_Transcript_1/3_Confidence_0.714_Length_4138|UNIGENE
REPRESENTATIVE TRANSCRIPT|sp|P30432|FUR2_DROME Furin-like protease 2
OS=Drosophila melanogaster GN=Fur2 PE=2 SV=2|e=0|from=72|to=3968|
frame=3|Fragment_10
ETFHNNCDEQCDEQKGCYGPGPGQCIACKHYKLDQTCVSKCPPRSFPNQQGICWPCHASC
ETCAGAGMDSCLTCAPAHLRVLDLAVCLQQCPEGYFENTEQGTCVPCGPTCGSCQDRPDN
CTSCDHHLVMYENKCYAACPQDTYETEDYNCAQCHPSCGTCTGPSETQCVTCKPGLLHHE
GACLSSCPANFRADKKRRECVPCPSGCTSCESSGMCTDCAEGWDADKKGKCWPNGAEKCE
QGEYYA
>Locus_4010_Transcript_1/3_Confidence_0.714_Length_4138|UNIGENE
REPRESENTATIVE TRANSCRIPT|sp|P30432|FUR2_DROME Furin-like protease 2
OS=Drosophila melanogaster GN=Fur2 PE=2 SV=2|e=0|from=72|to=3968|
frame=3|Fragment_11
DGNCHPCHSTCESCFGPSKSDCLSCEQSLRLQNSNCVGECTDGHFPEGGG
>Locus_4010_Transcript_1/3_Confidence_0.714_Length_4138|UNIGENE
REPRESENTATIVE TRANSCRIPT|sp|P30432|FUR2_DROME Furin-like protease 2
OS=Drosophila melanogaster GN=Fur2 PE=2 SV=2|e=0|from=72|to=3968|
frame=3|Fragment_12
CSPCLHTCTQCVSRQNCTACAPGLQLQTGECRATCADGYYNDRGGCSRCYISCQTCSGPR
RDQCVRCPTGWQIAAGECRPECPAGFYRGAYNCQRCHHSCRNCKDEGPLSCTSCPPGHML
DGGLCMECLGSQYYDPPTQLCKTCDSSCRTCNGPGKFSCVSCTSPLHLDKLNNQCVPCC
>Locus_4010_Transcript_1/3_Confidence_0.714_Length_4138|UNIGENE
REPRESENTATIVE TRANSCRIPT|sp|P30432|FUR2_DROME Furin-like protease 2
OS=Drosophila melanogaster GN=Fur2 PE=2 SV=2|e=0|from=72|to=3968|
frame=3|Fragment_13
ALVDQT
>Locus_4010_Transcript_1/3_Confidence_0.714_Length_4138|UNIGENE
REPRESENTATIVE TRANSCRIPT|sp|P30432|FUR2_DROME Furin-like protease 2
OS=Drosophila melanogaster GN=Fur2 PE=2 SV=2|e=0|from=72|to=3968|
frame=3|Fragment_14
CCHCDPNTGSCKNSSPAGKRRIALGLIETEQMIQKSKDI
>Locus_4010_Transcript_1/3_Confidence_0.714_Length_4138|UNIGENE
REPRESENTATIVE TRANSCRIPT|sp|P30432|FUR2_DROME Furin-like protease 2
OS=Drosophila melanogaster GN=Fur2 PE=2 SV=2|e=0|from=72|to=3968|
frame=3|Fragment_15

NQQSVSSYI
>Locus_4010_Transcript_1/3_Confidence_0.714_Length_4138|UNIGENE REPRESENTATIVE TRANSCRIPT|sp|P30432|FUR2_DROME Furin-like protease 2 OS=Drosophila melanogaster GN=Fur2 PE=2 SV=2|e=0|from=72|to=3968|frame=3|Fragment_16
LNGLPSLTVAAVIVMILIFVTIFATFQR
>Locus_4010_Transcript_1/3_Confidence_0.714_Length_4138|UNIGENE REPRESENTATIVE TRANSCRIPT|sp|P30432|FUR2_DROME Furin-like protease 2 OS=Drosophila melanogaster GN=Fur2 PE=2 SV=2|e=0|from=72|to=3968|frame=3|Fragment_17
MHTNRVSYSKLPTINTSS
>Locus_4010_Transcript_1/3_Confidence_0.714_Length_4138|UNIGENE REPRESENTATIVE TRANSCRIPT|sp|P30432|FUR2_DROME Furin-like protease 2 OS=Drosophila melanogaster GN=Fur2 PE=2 SV=2|e=0|from=72|to=3968|frame=3|Fragment_18
HMIDEISECDKLDGDYLVSDTDEVD
>Locus_4010_Transcript_2/3_Confidence_0.714_Length_4138|.|sp|P30432|FUR2_DROME Furin-like protease 2 OS=Drosophila melanogaster GN=Fur2 PE=2 SV=2|e=0|from=72|to=3968|frame=3|Fragment_1
IYGNEFAVHVPSGVEAADAIAVKHGFVNMGQIGSLKGYYLFQHNHVRKRSLSMSTEHHET
LNSEPEVYWVQQQHEKVRRKRDYGTIYNGQFSPLDTASELDALLLGG
>Locus_4010_Transcript_2/3_Confidence_0.714_Length_4138|.|sp|P30432|FUR2_DROME Furin-like protease 2 OS=Drosophila melanogaster GN=Fur2 PE=2 SV=2|e=0|from=72|to=3968|frame=3|Fragment_2
VAPSASRL
>Locus_4010_Transcript_2/3_Confidence_0.714_Length_4138|.|sp|P30432|FUR2_DROME Furin-like protease 2 OS=Drosophila melanogaster GN=Fur2 PE=2 SV=2|e=0|from=72|to=3968|frame=3|Fragment_3
HTARYRALGVGTQSIFPDPLFKEEWYLNGGAKDGLDMNVSPAWQKGYTGKGVVVSILDDG
IQTNHPDLALNYDPAAST
>Locus_4010_Transcript_2/3_Confidence_0.714_Length_4138|.|sp|P30432|FUR2_DROME Furin-like protease 2 OS=Drosophila melanogaster GN=Fur2 PE=2 SV=2|e=0|from=72|to=3968|frame=3|Fragment_4
KHGTRCAGEVAAVAYNNYCGIGVAYNASIGGVRMLDGVVNDAVEARALGLNPDHIDVYSA
SWGPEDDGKTVDGPGPLARRAFIYGVTSGRKGKGSIFVWASGNGGRHTDSCNCDGYTNSI
FTLSISSATQGGYKPWYLEECSSTLASTYSSGTPGHDKSVATVDMDARLRPDHICTVEHT
GTSASAPLAAGICALALEANPALTWRDMQYLVVLTSRSAPLEKEAGWILNGVKRKVSHKF
GYGLMDAGAMVSLAEQWTTVPPQHICKSQEIAEERLIEPTYGNTLVAHMDVTGCSGTLNE
VRFLEHVQCKITLRFFPRGNLKILLTSPMGTTSTLLFERPRDVVSSNFDDWPFLSVHFWG
EKADGRWTLQVINAGNRRVNQAGILKKWQLIFYGTNTNPIR
>Locus_4010_Transcript_2/3_Confidence_0.714_Length_4138|.|sp|P30432|FUR2_DROME Furin-like protease 2 OS=Drosophila melanogaster GN=Fur2 PE=2 SV=2|e=0|from=72|to=3968|frame=3|Fragment_5
LRSPNPS
>Locus_4010_Transcript_2/3_Confidence_0.714_Length_4138|.|sp|P30432|FUR2_DROME Furin-like protease 2 OS=Drosophila melanogaster GN=Fur2 PE=2 SV=2|e=0|from=72|to=3968|frame=3|Fragment_6
FQFPGQDALRRVVPQPPQYFTDFNT
>Locus_4010_Transcript_2/3_Confidence_0.714_Length_4138|.|sp|P30432|FUR2_DROME Furin-like protease 2 OS=Drosophila melanogaster GN=Fur2 PE=2 SV=2|e=0|from=72|to=3968|frame=3|Fragment_7
GTYRNYPDLF
>Locus_4010_Transcript_2/3_Confidence_0.714_Length_4138|.|sp|P30432|FUR2_DROME Furin-like protease 2 OS=Drosophila melanogaster GN=Fur2

PE=2 SV=2|e=0|from=72|to=3968|frame=3|Fragment_8
GAGSDPEVAIVSPD
>Locus_4010_Transcript_2/3_Confidence_0.714_Length_4138|.|sp|P30432|
FUR2_DROME Furin-like protease 2 OS=Drosophila melanogaster GN=Fur2
PE=2 SV=2|e=0|from=72|to=3968|frame=3|Fragment_9
DGMLAQASATNVVVNNKT
>Locus_4010_Transcript_2/3_Confidence_0.714_Length_4138|.|sp|P30432|
FUR2_DROME Furin-like protease 2 OS=Drosophila melanogaster GN=Fur2
PE=2 SV=2|e=0|from=72|to=3968|frame=3|Fragment_10
ETFHNNCDEQCDEQKGCYGPGPGQCIACKHYKLDQTCVSKCPPRSFPNQQGICWPCHASC
ETCAGAGMDSCLTCAPAHLRVLDLAVCLQQCPEGYFENTEQGTCVPCGPTCGSCQDRPDN
CTSCDHHLVMYENKCYAACPQDTYETEDYNCAQCHPSCGTCTGPSETQCVTCKPGLLHHE
GACLSSCPANFRADKKRRECVPCPSGCTSCESSGMCTDCAEGWDADKKGKCWPNGAEKCE
QGEYYA
>Locus_4010_Transcript_2/3_Confidence_0.714_Length_4138|.|sp|P30432|
FUR2_DROME Furin-like protease 2 OS=Drosophila melanogaster GN=Fur2
PE=2 SV=2|e=0|from=72|to=3968|frame=3|Fragment_11
DGNCHPCHSTCESCFGPSKSDCLSCEQSLRLQNSNCVGECTDGHFPEGGG
>Locus_4010_Transcript_2/3_Confidence_0.714_Length_4138|.|sp|P30432|
FUR2_DROME Furin-like protease 2 OS=Drosophila melanogaster GN=Fur2
PE=2 SV=2|e=0|from=72|to=3968|frame=3|Fragment_12
CSPCLHTCTQCVSRQNCTACAPGLQLQTGECRATCADGYYNDRGGCSRCYISCQTCSGPR
RDQCVRCPTGWQIAAGECRPECPAGFYRGAYNCQRCHHSCRNCKDEGPLSCTSCPPGHML
DGGLCMECLGSQYYDPPTQLCKTCDSSCRTCNGPGKFSCVSCTSPLHLDKLNNQCVPCC
>Locus_4010_Transcript_2/3_Confidence_0.714_Length_4138|.|sp|P30432|
FUR2_DROME Furin-like protease 2 OS=Drosophila melanogaster GN=Fur2
PE=2 SV=2|e=0|from=72|to=3968|frame=3|Fragment_13
ALVDQT
>Locus_4010_Transcript_2/3_Confidence_0.714_Length_4138|.|sp|P30432|
FUR2_DROME Furin-like protease 2 OS=Drosophila melanogaster GN=Fur2
PE=2 SV=2|e=0|from=72|to=3968|frame=3|Fragment_14
CCHCDPNTGSCKNSSPAGKRRIALGLIETEQMIQKSKDI
>Locus_4010_Transcript_2/3_Confidence_0.714_Length_4138|.|sp|P30432|
FUR2_DROME Furin-like protease 2 OS=Drosophila melanogaster GN=Fur2
PE=2 SV=2|e=0|from=72|to=3968|frame=3|Fragment_15
NQQSVSSYI
>Locus_4010_Transcript_2/3_Confidence_0.714_Length_4138|.|sp|P30432|
FUR2_DROME Furin-like protease 2 OS=Drosophila melanogaster GN=Fur2
PE=2 SV=2|e=0|from=72|to=3968|frame=3|Fragment_16
LNGLPSLTVAAVIVMILIFVTIFATFQR
>Locus_4010_Transcript_2/3_Confidence_0.714_Length_4138|.|sp|P30432|
FUR2_DROME Furin-like protease 2 OS=Drosophila melanogaster GN=Fur2
PE=2 SV=2|e=0|from=72|to=3968|frame=3|Fragment_17
MHTNRVSYSKLPTINTSS
>Locus_4010_Transcript_2/3_Confidence_0.714_Length_4138|.|sp|P30432|
FUR2_DROME Furin-like protease 2 OS=Drosophila melanogaster GN=Fur2
PE=2 SV=2|e=0|from=72|to=3968|frame=3|Fragment_18
HMIDEISECDKLDGDYLVSDTDEVD
>Locus_4010_Transcript_3/3_Confidence_0.714_Length_4138|.|sp|P30432|
FUR2_DROME Furin-like protease 2 OS=Drosophila melanogaster GN=Fur2
PE=2 SV=2|e=0|from=72|to=3968|frame=3|Fragment_1
IYGNEFAVHVPSGVEAADAIAVKHGFVNMGQIGSLKGYYLFQHNHVRKRSLSMSTEHHET
LNSEPEVYWVQQQHEKVRRKRDYGTIYNGQFSPLDTASELDALLLGG
>Locus_4010_Transcript_3/3_Confidence_0.714_Length_4138|.|sp|P30432|

FUR2_DROME Furin-like protease 2 OS=Drosophila melanogaster GN=Fur2
PE=2 SV=2|e=0|from=72|to=3968|frame=3|Fragment_2
VAPSASRL
>Locus_4010_Transcript_3/3_Confidence_0.714_Length_4138|.|sp|P30432|
FUR2_DROME Furin-like protease 2 OS=Drosophila melanogaster GN=Fur2
PE=2 SV=2|e=0|from=72|to=3968|frame=3|Fragment_3
HTARYRALGVGTQSIFPDPLFKEEWYLNGGAKDGLDMNVSPAWQKGYTGKGVVVSILDDG
IQTNHPDLALNYDPAAST
>Locus_4010_Transcript_3/3_Confidence_0.714_Length_4138|.|sp|P30432|
FUR2_DROME Furin-like protease 2 OS=Drosophila melanogaster GN=Fur2
PE=2 SV=2|e=0|from=72|to=3968|frame=3|Fragment_4
KHGTRCAGEVAAVAYNNYCGIGVAYNASIGGVRMLDGVVNDAVEARALGLNPDHIDVYSA
SWGPEDDGKTVDGPGPLARRAFIYGVTSGRKGKGSIFVWASGNGGRHTDSCNCDGYTNSI
FTLSISSATQGGYKPWYLEECSSTLASTYSSGTPGHDKSVATVDMDARLRPDHICTVEHT
GTSASAPLAAGICALALEANPALTWRDMQYLVVLTSRSAPLEKEAGWILNGVKRKVSHKF
GYGLMDAGAMVSLAEQWTTVPPQHICKSQEIAEERLIEPTYGNTLVAHMDVTGCSGTLNE
VRFLEHVQCKITLRFFPRGNLKILLTSPMGTTSTLLFERPRDVVSSNFDDWPFLSVHFWG
EKADGRWTLQVINAGNRRVNQAGILKKWQLIFYGTNTNPIR
>Locus_4010_Transcript_3/3_Confidence_0.714_Length_4138|.|sp|P30432|
FUR2_DROME Furin-like protease 2 OS=Drosophila melanogaster GN=Fur2
PE=2 SV=2|e=0|from=72|to=3968|frame=3|Fragment_5
LRSPNPS
>Locus_4010_Transcript_3/3_Confidence_0.714_Length_4138|.|sp|P30432|
FUR2_DROME Furin-like protease 2 OS=Drosophila melanogaster GN=Fur2
PE=2 SV=2|e=0|from=72|to=3968|frame=3|Fragment_6
FQFPGQDALRRVVPQPPQYFTDFNT
>Locus_4010_Transcript_3/3_Confidence_0.714_Length_4138|.|sp|P30432|
FUR2_DROME Furin-like protease 2 OS=Drosophila melanogaster GN=Fur2
PE=2 SV=2|e=0|from=72|to=3968|frame=3|Fragment_7
GTYRNYPDLF
>Locus_4010_Transcript_3/3_Confidence_0.714_Length_4138|.|sp|P30432|
FUR2_DROME Furin-like protease 2 OS=Drosophila melanogaster GN=Fur2
PE=2 SV=2|e=0|from=72|to=3968|frame=3|Fragment_8
GAGSDPEVAIVSPD
>Locus_4010_Transcript_3/3_Confidence_0.714_Length_4138|.|sp|P30432|
FUR2_DROME Furin-like protease 2 OS=Drosophila melanogaster GN=Fur2
PE=2 SV=2|e=0|from=72|to=3968|frame=3|Fragment_9
DGMLAQASATNVVVNNKT
>Locus_4010_Transcript_3/3_Confidence_0.714_Length_4138|.|sp|P30432|
FUR2_DROME Furin-like protease 2 OS=Drosophila melanogaster GN=Fur2
PE=2 SV=2|e=0|from=72|to=3968|frame=3|Fragment_10
ETFHNNCDEQCDEQKGCYGPGPGQCIACKHYKLDQTCVSKCPPRSFPNQQGICWPCHASC
ETCAGAGMDSCLTCAPAHLRVLDLAVCLQQCPEGYFENTEQGTCVPCGPTCGSCQDRPDN
CTSCDHHLVMYENKCYAACPQDTYETEDYNCAQCHPSCGTCTGPSETQCVTCKPGLLHHE
GACLSSCPANFRADKKRRECVPCPSGCTSCESSGMCTDCAEGWDADKKGKCWPNGAEKCE
QGEYYA
>Locus_4010_Transcript_3/3_Confidence_0.714_Length_4138|.|sp|P30432|
FUR2_DROME Furin-like protease 2 OS=Drosophila melanogaster GN=Fur2
PE=2 SV=2|e=0|from=72|to=3968|frame=3|Fragment_11
DGNCHPCHSTCESCFGPSKSDCLSCEQSLRLQNSNCVGECTDGHFPEGGG
>Locus_4010_Transcript_3/3_Confidence_0.714_Length_4138|.|sp|P30432|
FUR2_DROME Furin-like protease 2 OS=Drosophila melanogaster GN=Fur2
PE=2 SV=2|e=0|from=72|to=3968|frame=3|Fragment_12
CSPCLHTCTQCVSRQNCTACAPGLQLQTGECRATCADGYYNDRGGCSRCYISCQTCSGPR

RDQCVRCPTGWQIAAGECRPECPAGFYRGAYNCQRCHHSCRNCKDEGPLSCTSCPPGHML
DGGLCMECLGSQYYDPPTQLCKTCDSSCRTCNGPGKFSCVSCTSPLHLDKLNNQCVPCC
>Locus_4010_Transcript_3/3_Confidence_0.714_Length_4138|.|sp|P30432|
FUR2_DROME Furin-like protease 2 OS=Drosophila melanogaster GN=Fur2
PE=2 SV=2|e=0|from=72|to=3968|frame=3|Fragment_13
ALVDQT
>Locus_4010_Transcript_3/3_Confidence_0.714_Length_4138|.|sp|P30432|
FUR2_DROME Furin-like protease 2 OS=Drosophila melanogaster GN=Fur2
PE=2 SV=2|e=0|from=72|to=3968|frame=3|Fragment_14
CCHCDPNTGSCKNSSPAGKRRIALGLIETEQMIQKSKDI
>Locus_4010_Transcript_3/3_Confidence_0.714_Length_4138|.|sp|P30432|
FUR2_DROME Furin-like protease 2 OS=Drosophila melanogaster GN=Fur2
PE=2 SV=2|e=0|from=72|to=3968|frame=3|Fragment_15
NQQSVSSYI
>Locus_4010_Transcript_3/3_Confidence_0.714_Length_4138|.|sp|P30432|
FUR2_DROME Furin-like protease 2 OS=Drosophila melanogaster GN=Fur2
PE=2 SV=2|e=0|from=72|to=3968|frame=3|Fragment_16
LNGLPSLTVAAVIVMILIFVTIFATFQR
>Locus_4010_Transcript_3/3_Confidence_0.714_Length_4138|.|sp|P30432|
FUR2_DROME Furin-like protease 2 OS=Drosophila melanogaster GN=Fur2
PE=2 SV=2|e=0|from=72|to=3968|frame=3|Fragment_17
MHTNRVSYSKLPTINTSS
>Locus_4010_Transcript_3/3_Confidence_0.714_Length_4138|.|sp|P30432|
FUR2_DROME Furin-like protease 2 OS=Drosophila melanogaster GN=Fur2
PE=2 SV=2|e=0|from=72|to=3968|frame=3|Fragment_18
HMIDEISECDKLDGDYLVSDTDEVD
>Locus_23471_Transcript_1/1_Confidence_1.000_Length_326|UNIGENE
REPRESENTATIVE TRANSCRIPT|sp|P30974|TLR1_DROME Tachykinin-like
peptides receptor 86C OS=Drosophila melanogaster GN=Takr86C PE=2
SV=2|e=6.05896E-41|from=7|to=321|frame=1|Fragment_1
WSSAFGFMLFIAVGGNAIVMWIVIAHRRMRTVTNYFLVNLSLADLLMSVLNCLFNFIYML
KSDWIFGDWYCTINNFIANVTVAASIFTLTGISFDRYMAIVRPLE

>Locus_8761_Transcript_1/5_Confidence_0.308_Length_1739|UNIGENE
REPRESENTATIVE TRANSCRIPT|sp|P32870|CYA1_DROME Ca(2+)/calmodulin-
responsive adenylate cyclase OS=Drosophila melanogaster GN=rut PE=1
SV=2|e=3.71316E-13|from=459|to=740|frame=-1|Fragment_1
ESNGYTTDEQGLENVSLLNEAGLTDAEGALSDVNSIYHHPDHENG
>Locus_8761_Transcript_1/5_Confidence_0.308_Length_1739|UNIGENE
REPRESENTATIVE TRANSCRIPT|sp|P32870|CYA1_DROME Ca(2+)/calmodulin-
responsive adenylate cyclase OS=Drosophila melanogaster GN=rut PE=1
SV=2|e=3.71316E-13|from=459|to=740|frame=-1|Fragment_2
SGMY
>Locus_8761_Transcript_1/5_Confidence_0.308_Length_1739|UNIGENE
REPRESENTATIVE TRANSCRIPT|sp|P32870|CYA1_DROME Ca(2+)/calmodulin-
responsive adenylate cyclase OS=Drosophila melanogaster GN=rut PE=1
SV=2|e=3.71316E-13|from=459|to=740|frame=-1|Fragment_3
CDSEGVDGWPGLALLDIRSVSESITKKFG

>Locus_19463_Transcript_1/1_Confidence_1.000_Length_349|UNIGENE
REPRESENTATIVE TRANSCRIPT|sp|P33085-4|SHAKB_DROME Isoform D of
Innexin shaking-B OS=Drosophila melanogaster GN=shakB|e=1.50702E-36|
from=2|to=298|frame=2|Fragment_1
FW

>Locus_19463_Transcript_1/1_Confidence_1.000_Length_349|UNIGENE
REPRESENTATIVE TRANSCRIPT|sp|P33085-4|SHAKB_DROME Isoform D of
Innexin shaking-B OS=Drosophila melanogaster GN=shakB|e=1.50702E-36|
from=2|to=298|frame=2|Fragment_2
YRVVIIFSPRMRVYLLRMRFRLIKRDAVEVIVRRSKMGDWFLLYMLGENVDSVIFRDVMQ
ELAIRLGQDQHHHVPGVKGEIQEA

>Locus_27253_Transcript_1/1_Confidence_1.000_Length_387|UNIGENE
REPRESENTATIVE TRANSCRIPT|sp|P33450|FAT_DROME Cadherin-related tumor
suppressor OS=Drosophila melanogaster GN=ft PE=1 SV=3|e=1.22924E-38|
from=2|to=385|frame=2|Fragment_1
WGSDVFSLNPVTGLLTLAGKLDYEEIQHYVLVVQAQDAGSPALTSTAMLYINVIDVNDNA
PAFEPRTYTKRIPENIPKGSEVIKLIATDKDSGANSIMKFSTINGDDTGDFSVDSNGIIR
TQRVLDRE
>Locus_27253_Transcript_1/1_Confidence_1.000_Length_387|.|sp|P33450|
FAT_DROME Cadherin-related tumor suppressor OS=Drosophila
melanogaster GN=ft PE=1 SV=3|e=2.57503E-20|from=17|to=385|frame=2|
Fragment_1
FSLNPVTGLLTLAGKLDYEEIQHYVLVVQAQDAGSPALTSTAMLYINVIDVNDNAPAFEP
RTYTKRIPENIPKGSEVIKLIATDKDSGANSIMKFSTINGDDTGDFSV
>Locus_27253_Transcript_1/1_Confidence_1.000_Length_387|.|sp|P33450|
FAT_DROME Cadherin-related tumor suppressor OS=Drosophila
melanogaster GN=ft PE=1 SV=3|e=2.57503E-20|from=17|to=385|frame=2|
Fragment_2
DSNGIIRTQRVLDRE
>Locus_27253_Transcript_1/1_Confidence_1.000_Length_387|.|sp|P33450|
FAT_DROME Cadherin-related tumor suppressor OS=Drosophila
melanogaster GN=ft PE=1 SV=3|e=3.36309E-20|from=8|to=385|frame=2|
Fragment_1
SDVFSLNPVTGLLTLAGKLDYEEIQHYVLVVQAQDAGSPALTSTAMLYINVIDVNDNAPA
FEPRTYTKRIPENIPKGSEVIKLIATDKDSGANSIMKFSTINGDDTGDFSVDS
>Locus_27253_Transcript_1/1_Confidence_1.000_Length_387|.|sp|P33450|
FAT_DROME Cadherin-related tumor suppressor OS=Drosophila
melanogaster GN=ft PE=1 SV=3|e=3.36309E-20|from=8|to=385|frame=2|
Fragment_2
NGIIRTQRVLDRE
>Locus_27253_Transcript_1/1_Confidence_1.000_Length_387|.|sp|P33450|
FAT_DROME Cadherin-related tumor suppressor OS=Drosophila
melanogaster GN=ft PE=1 SV=3|e=4.85629E-19|from=38|to=385|frame=2|
Fragment_1
GLLTLAGKLDYEEIQHYVLVVQAQDAGSPALTSTAMLYINVIDVNDNAPAFEPRTYTKRI
PENIPKGSEVIKLIATDKDSGANSIMKFSTINGDDTGDFSVDS
>Locus_27253_Transcript_1/1_Confidence_1.000_Length_387|.|sp|P33450|
FAT_DROME Cadherin-related tumor suppressor OS=Drosophila
melanogaster GN=ft PE=1 SV=3|e=4.85629E-19|from=38|to=385|frame=2|
Fragment_2
NGIIRTQRVLDRE
>Locus_27253_Transcript_1/1_Confidence_1.000_Length_387|.|sp|P33450|
FAT_DROME Cadherin-related tumor suppressor OS=Drosophila
melanogaster GN=ft PE=1 SV=3|e=1.19615E-17|from=41|to=385|frame=2|
Fragment_1
LLTLAGKLDYEEIQHYVLVVQAQDAGSPALTSTAMLYINVIDVNDNAPAFEPRTYTKRIP
ENIPKGSEVIKLIATDKDSGANSIMKFSTINGDDTGDFSVDS
>Locus_27253_Transcript_1/1_Confidence_1.000_Length_387|.|sp|P33450|

FAT_DROME Cadherin-related tumor suppressor OS=Drosophila melanogaster GN=ft PE=1 SV=3|e=1.19615E-17|from=41|to=385|frame=2|
Fragment_2
NGIIRTQRVLDRE
>Locus_27253_Transcript_1/1_Confidence_1.000_Length_387|.|sp|P33450|
FAT_DROME Cadherin-related tumor suppressor OS=Drosophila melanogaster GN=ft PE=1 SV=3|e=6.56348E-16|from=11|to=385|frame=2|
Fragment_1
DVFSLNPVTGLLTLAGKLDYEEIQHYVLVVQAQDAGSPALTSTAMLYINVIDVNDNAPAF
EPRTYTKRIPENIPKGSEVIKLIATDKDSGANSIMKFSTINGD
>Locus_27253_Transcript_1/1_Confidence_1.000_Length_387|.|sp|P33450|
FAT_DROME Cadherin-related tumor suppressor OS=Drosophila melanogaster GN=ft PE=1 SV=3|e=6.56348E-16|from=11|to=385|frame=2|
Fragment_2
DTGDFSVDS
>Locus_27253_Transcript_1/1_Confidence_1.000_Length_387|.|sp|P33450|
FAT_DROME Cadherin-related tumor suppressor OS=Drosophila melanogaster GN=ft PE=1 SV=3|e=6.56348E-16|from=11|to=385|frame=2|
Fragment_3
NGIIRTQRVLDRE
>Locus_27253_Transcript_1/1_Confidence_1.000_Length_387|.|sp|P33450|
FAT_DROME Cadherin-related tumor suppressor OS=Drosophila melanogaster GN=ft PE=1 SV=3|e=1.46219E-15|from=17|to=385|frame=2|
Fragment_1
FSLNPVTGLLTLAGKLDYEEIQHYVLVVQAQDAGSPALTSTAMLYINVIDVNDNAPAFEP
RTYTKRIPENIPKGSEVIKLIATDKDSGANSIMKFSTI
>Locus_27253_Transcript_1/1_Confidence_1.000_Length_387|.|sp|P33450|
FAT_DROME Cadherin-related tumor suppressor OS=Drosophila melanogaster GN=ft PE=1 SV=3|e=1.46219E-15|from=17|to=385|frame=2|
Fragment_2
NGDDTGDFSVD
>Locus_27253_Transcript_1/1_Confidence_1.000_Length_387|.|sp|P33450|
FAT_DROME Cadherin-related tumor suppressor OS=Drosophila melanogaster GN=ft PE=1 SV=3|e=1.46219E-15|from=17|to=385|frame=2|
Fragment_3
SNGIIRTQRVLDRE
>Locus_27253_Transcript_1/1_Confidence_1.000_Length_387|.|sp|P33450|
FAT_DROME Cadherin-related tumor suppressor OS=Drosophila melanogaster GN=ft PE=1 SV=3|e=1.46219E-15|from=17|to=385|frame=2|
Fragment_1
FSLNPVTGLLTLAGKLDYEEIQHYVLVVQAQDAGSPALTSTAMLYINVIDVNDNAPAFEP
RTYTKRIPENIPKGSEVIKLIATDKDSGAN
>Locus_27253_Transcript_1/1_Confidence_1.000_Length_387|.|sp|P33450|
FAT_DROME Cadherin-related tumor suppressor OS=Drosophila melanogaster GN=ft PE=1 SV=3|e=1.46219E-15|from=17|to=385|frame=2|
Fragment_2
SIMKFSTINGDDTGDFSVDSN
>Locus_27253_Transcript_1/1_Confidence_1.000_Length_387|.|sp|P33450|
FAT_DROME Cadherin-related tumor suppressor OS=Drosophila melanogaster GN=ft PE=1 SV=3|e=1.46219E-15|from=17|to=385|frame=2|
Fragment_3
GIIRTQRVLDRE
>Locus_27253_Transcript_1/1_Confidence_1.000_Length_387|.|sp|P33450|
FAT_DROME Cadherin-related tumor suppressor OS=Drosophila

melanogaster GN=ft PE=1 SV=3|e=1.36857E-13|from=8|to=373|frame=2|
Fragment_1
SDVFSLNPVTGLLTLAGKLDYEEIQHYVLVVQAQDAGSPALTSTAMLYINVIDVNDNAPA
FEPRTYTKRIPENIPKGSEVIKLIATDKDSGANSIMKFSTINGDDTGDFSVD
>Locus_27253_Transcript_1/1_Confidence_1.000_Length_387|.|sp|P33450|
FAT_DROME Cadherin-related tumor suppressor OS=Drosophila
melanogaster GN=ft PE=1 SV=3|e=1.36857E-13|from=8|to=373|frame=2|
Fragment_2
SNGIIRTQRV
>Locus_27253_Transcript_1/1_Confidence_1.000_Length_387|.|sp|P33450|
FAT_DROME Cadherin-related tumor suppressor OS=Drosophila
melanogaster GN=ft PE=1 SV=3|e=5.20055E-13|from=17|to=385|frame=2|
Fragment_1
FSLNPVTGLLTLAGKLDYEEIQHYVLVVQAQDAG
>Locus_27253_Transcript_1/1_Confidence_1.000_Length_387|.|sp|P33450|
FAT_DROME Cadherin-related tumor suppressor OS=Drosophila
melanogaster GN=ft PE=1 SV=3|e=5.20055E-13|from=17|to=385|frame=2|
Fragment_2
SPALTSTAMLYINVIDVNDNAPAFEPRTYTKRIPENIPKGSEVIKLIATDKDSGANSIMK
FSTINGDDTGDFSVDS
>Locus_27253_Transcript_1/1_Confidence_1.000_Length_387|.|sp|P33450|
FAT_DROME Cadherin-related tumor suppressor OS=Drosophila
melanogaster GN=ft PE=1 SV=3|e=5.20055E-13|from=17|to=385|frame=2|
Fragment_3
NGIIRTQRVLDRE
>Locus_27253_Transcript_1/1_Confidence_1.000_Length_387|.|sp|P33450|
FAT_DROME Cadherin-related tumor suppressor OS=Drosophila
melanogaster GN=ft PE=1 SV=3|e=6.79213E-13|from=17|to=385|frame=2|
Fragment_1
FSLNPVTGLLTLAGKLDYEEIQHYVLVVQAQDAGSPALTSTAMLYINVIDVNDNAPAFEP
RTYTKRIPENIPKGSEVIKLIATDKDSGANSIMKFSTINGDDTGDFSVDSNGIIRTQRVL
DRE
>Locus_27253_Transcript_1/1_Confidence_1.000_Length_387|.|sp|P33450|
FAT_DROME Cadherin-related tumor suppressor OS=Drosophila
melanogaster GN=ft PE=1 SV=3|e=6.79213E-13|from=17|to=367|frame=2|
Fragment_1
FSLNPVTGLLTLAGKLDYEEIQHYVLVVQAQDAG
>Locus_27253_Transcript_1/1_Confidence_1.000_Length_387|.|sp|P33450|
FAT_DROME Cadherin-related tumor suppressor OS=Drosophila
melanogaster GN=ft PE=1 SV=3|e=6.79213E-13|from=17|to=367|frame=2|
Fragment_2
SPALTSTAMLYINVIDVNDNAPAFEPRTYTKRIPENIPKGSEVIKLIATDKDSGANSIMK
FSTINGDDTGDFSVDS
>Locus_27253_Transcript_1/1_Confidence_1.000_Length_387|.|sp|P33450|
FAT_DROME Cadherin-related tumor suppressor OS=Drosophila
melanogaster GN=ft PE=1 SV=3|e=6.79213E-13|from=17|to=367|frame=2|
Fragment_3
NGIIRTQ
>Locus_27253_Transcript_1/1_Confidence_1.000_Length_387|.|sp|P33450|
FAT_DROME Cadherin-related tumor suppressor OS=Drosophila
melanogaster GN=ft PE=1 SV=3|e=1.51313E-12|from=11|to=385|frame=2|
Fragment_1
DVFSLNPVTGLLTLAGK
>Locus_27253_Transcript_1/1_Confidence_1.000_Length_387|.|sp|P33450|

FAT_DROME Cadherin-related tumor suppressor OS=Drosophila melanogaster GN=ft PE=1 SV=3|e=1.51313E-12|from=11|to=385|frame=2|
Fragment_2
LDYEEIQHYVLVVQAQDAGSPALTSTAMLYINVIDVNDNAPAFEPRTYTKRIPENIPKGS
EVIKLIATDKDSGANSIMKFSTINGDDTGDFSVDSNGIIRTQRVLDRE
>Locus_27253_Transcript_1/1_Confidence_1.000_Length_387|.|sp|P33450|
FAT_DROME Cadherin-related tumor suppressor OS=Drosophila melanogaster GN=ft PE=1 SV=3|e=7.50958E-12|from=17|to=346|frame=2|
Fragment_1
FSLNPVTGLLTLAGKLDYEEIQHYVLVVQAQDAGSPALTS
>Locus_27253_Transcript_1/1_Confidence_1.000_Length_387|.|sp|P33450|
FAT_DROME Cadherin-related tumor suppressor OS=Drosophila melanogaster GN=ft PE=1 SV=3|e=7.50958E-12|from=17|to=346|frame=2|
Fragment_2
TAMLYINVIDVNDNAPAFEPRTYTK
>Locus_27253_Transcript_1/1_Confidence_1.000_Length_387|.|sp|P33450|
FAT_DROME Cadherin-related tumor suppressor OS=Drosophila melanogaster GN=ft PE=1 SV=3|e=7.50958E-12|from=17|to=346|frame=2|
Fragment_3
RIPENIPKGSEVIKLIATDKDSGANSIMKFSTINGDDTGDFSVDS
>Locus_27253_Transcript_1/1_Confidence_1.000_Length_387|.|sp|P33450|
FAT_DROME Cadherin-related tumor suppressor OS=Drosophila melanogaster GN=ft PE=1 SV=3|e=1.67296E-11|from=17|to=385|frame=2|
Fragment_1
FSLNPVTGLLTLAGKLDYEEIQHYVLVVQAQDAG
>Locus_27253_Transcript_1/1_Confidence_1.000_Length_387|.|sp|P33450|
FAT_DROME Cadherin-related tumor suppressor OS=Drosophila melanogaster GN=ft PE=1 SV=3|e=1.67296E-11|from=17|to=385|frame=2|
Fragment_2
SPALTSTAMLYINVIDVNDNAPAFEPRTYTKRIPENI
>Locus_27253_Transcript_1/1_Confidence_1.000_Length_387|.|sp|P33450|
FAT_DROME Cadherin-related tumor suppressor OS=Drosophila melanogaster GN=ft PE=1 SV=3|e=1.67296E-11|from=17|to=385|frame=2|
Fragment_3
PKGSEVIKLIATDKDS
>Locus_27253_Transcript_1/1_Confidence_1.000_Length_387|.|sp|P33450|
FAT_DROME Cadherin-related tumor suppressor OS=Drosophila melanogaster GN=ft PE=1 SV=3|e=1.67296E-11|from=17|to=385|frame=2|
Fragment_4
GANSIMKFSTINGDDTGDFSVDSNGIIRTQRVLDRE
>Locus_27253_Transcript_1/1_Confidence_1.000_Length_387|.|sp|P33450|
FAT_DROME Cadherin-related tumor suppressor OS=Drosophila melanogaster GN=ft PE=1 SV=3|e=3.72697E-11|from=59|to=385|frame=2|
Fragment_1
KLDYEEIQHYVLVVQAQDAGSPALTSTAMLYINVIDVNDNAPAFEPRTYTKRIPENIPKG
SEVIKLIATDKDSGANSIMKFSTINGDDTGD
>Locus_27253_Transcript_1/1_Confidence_1.000_Length_387|.|sp|P33450|
FAT_DROME Cadherin-related tumor suppressor OS=Drosophila melanogaster GN=ft PE=1 SV=3|e=3.72697E-11|from=59|to=385|frame=2|
Fragment_2
FSVD
>Locus_27253_Transcript_1/1_Confidence_1.000_Length_387|.|sp|P33450|
FAT_DROME Cadherin-related tumor suppressor OS=Drosophila melanogaster GN=ft PE=1 SV=3|e=3.72697E-11|from=59|to=385|frame=2|

Fragment_3
SNGIIRTQRVLDRE

>Locus_23907_Transcript_1/1_Confidence_1.000_Length_469|UNIGENE REPRESENTATIVE TRANSCRIPT|sp|P34082|FAS2_DROME Fasciclin-2 OS=Drosophila melanogaster GN=Fas2 PE=1 SV=1|e=5.61408E-41|from=15|to=467|frame=3|Fragment_1
KTTKLVCIASGRPAPAVTFKRWGADVEMLPGPQPGDDRVVLEQEVNS
>Locus_23907_Transcript_1/1_Confidence_1.000_Length_469|UNIGENE REPRESENTATIVE TRANSCRIPT|sp|P34082|FAS2_DROME Fasciclin-2 OS=Drosophila melanogaster GN=Fas2 PE=1 SV=1|e=5.61408E-41|from=15|to=467|frame=3|Fragment_2
ITNVVRSDDGLYECIARNKGDTYYKTGHITVEFPPTFEASKNQPPVFSWDQRTANLSCLA
ESIPNATIEWRLNDRPI
>Locus_23907_Transcript_1/1_Confidence_1.000_Length_469|UNIGENE REPRESENTATIVE TRANSCRIPT|sp|P34082|FAS2_DROME Fasciclin-2 OS=Drosophila melanogaster GN=Fas2 PE=1 SV=1|e=5.61408E-41|from=15|to=467|frame=3|Fragment_3
IEDRFYRIEGSGPQSNL
>Locus_27787_Transcript_1/1_Confidence_1.000_Length_365|.|sp|P34082|FAS2_DROME Fasciclin-2 OS=Drosophila melanogaster GN=Fas2 PE=1 SV=1|e=3.66529E-30|from=1|to=363|frame=-3|Fragment_1
TIYHFRFAATNAVGPGPYSNNIAQATPRRSPPEEPKLLVG
>Locus_27787_Transcript_1/1_Confidence_1.000_Length_365|.|sp|P34082|FAS2_DROME Fasciclin-2 OS=Drosophila melanogaster GN=Fas2 PE=1 SV=1|e=3.66529E-30|from=1|to=363|frame=-3|Fragment_2
VSNEVDGGIVTSPYADHYELRWKIPADNGEPIDYYQLRYCPVQRQNGAWAELDSLCNARE
LRAFETTAHELNDLLADSHYK

>Locus_8734_Transcript_4/5_Confidence_0.692_Length_2062|UNIGENE REPRESENTATIVE TRANSCRIPT|sp|P42787|CBPD_DROME Carboxypeptidase D OS=Drosophila melanogaster GN=svr PE=1 SV=2|e=2.0915E-167|from=8|to=2059|frame=2|Fragment_1
SIGQSVKGHYLWVLEISANPGEHITGKPSIKYVGNMHGNEVVGREILLLFAKFLCENYLI
DEQVTRIVNTTRIHLLPSMNPDGYDMAKPEEINNVVGRNNAHNIDLNRNFPDQYRS
>Locus_8734_Transcript_4/5_Confidence_0.692_Length_2062|UNIGENE REPRESENTATIVE TRANSCRIPT|sp|P42787|CBPD_DROME Carboxypeptidase D OS=Drosophila melanogaster GN=svr PE=1 SV=2|e=2.0915E-167|from=8|to=2059|frame=2|Fragment_2
LLNVKPEPETEAVMAWTLSERFVLSANLHNGALVANYPFDENPSNI
>Locus_8734_Transcript_4/5_Confidence_0.692_Length_2062|UNIGENE REPRESENTATIVE TRANSCRIPT|sp|P42787|CBPD_DROME Carboxypeptidase D OS=Drosophila melanogaster GN=svr PE=1 SV=2|e=2.0915E-167|from=8|to=2059|frame=2|Fragment_3
ILQKVANPSPDDDVFQHLARIYSNAHRKMHLGQPCAEHPKEHFLNGITNGAAWYSVSGSM
QDWNYVEADCFEITLELGCVKYPGAEALPELWLDNKMALLDYIELAHIGVRGFVHSSIGN
PISSAEVLVKGRKHITKTGSKGEYHRLLLPGSYNITVSSYGYESQTVQIIVPDVKPYSVG
YNFTLMRDDPQHWSSAYDFRLIQHVVNTRYRTTNEIANYMAYLGNKYPNIAEFRTEYDDT
SMLLHSLKITDQIGSSEEKKFH
>Locus_8734_Transcript_4/5_Confidence_0.692_Length_2062|UNIGENE REPRESENTATIVE TRANSCRIPT|sp|P42787|CBPD_DROME Carboxypeptidase D OS=Drosophila melanogaster GN=svr PE=1 SV=2|e=2.0915E-167|from=8|to=2059|frame=2|Fragment_4
SEG

>Locus_8734_Transcript_4/5_Confidence_0.692_Length_2062|UNIGENE REPRESENTATIVE TRANSCRIPT|sp|P42787|CBPD_DROME Carboxypeptidase D OS=Drosophila melanogaster GN=svr PE=1 SV=2|e=2.0915E-167|from=8|to=2059|frame=2|Fragment_5
VGSEMVLNTAEHLMAAFKIGEQEHMQILRNAVVHVIPVLDDNFDSIIQNYRLHEDVCNPE
SFKQEFGERMIASNADKLVTAFKKLLWSGRYDFILNFGSGGCGLSYPRVNDSGFIYDKLL
NNMHNLINSTTSHETCCNNQGNQNHAEITERLTNLMYNEYNVPIVTVRNSCCKMPRQEQL
ANVWRNSLKPLMSFLELIETGIQGAILGNTMNPLRHAVVTLKQFNRTFNVTRNLAYYKIM
LPVGAYNV
>Locus_8734_Transcript_2/5_Confidence_0.692_Length_2062|.|sp|P42787|CBPD_DROME Carboxypeptidase D OS=Drosophila melanogaster GN=svr PE=1 SV=2|e=1.038E-166|from=8|to=2059|frame=2|Fragment_1
SIGQSVKGHYLWVLEISANPGEHITGKPSIKYVGNMHGNEVVGREILLLFAKFLCENYLI
DEQVTRIVNTTRIHLLPSMNPDGYDMAKPEEINNVVGRNNAHNIDLNRNFPDQYRS
>Locus_8734_Transcript_2/5_Confidence_0.692_Length_2062|.|sp|P42787|CBPD_DROME Carboxypeptidase D OS=Drosophila melanogaster GN=svr PE=1 SV=2|e=1.038E-166|from=8|to=2059|frame=2|Fragment_2
LLNVKPEPETEAVMAWTLSERFVLSANLHNGALVANYPFDENPSNI
>Locus_8734_Transcript_2/5_Confidence_0.692_Length_2062|.|sp|P42787|CBPD_DROME Carboxypeptidase D OS=Drosophila melanogaster GN=svr PE=1 SV=2|e=1.038E-166|from=8|to=2059|frame=2|Fragment_3
ILQKVANPSPDDDVFQHLARIYSNAHRKMHLGQPCAEHPKEHFLNGITNGAAWYSVSGSM
QDWNYVEADCFEITLELGCVKYPRAEALPELWLDNKMALLDYIELAHIGVRGFVHSSIGN
PISSAEVLVKGRKHITRTGSKGEYHRLLLPGLYNITVSSYGYESQTVQIIVPDVKPYSVG
YNFTLMRDDPQHWSSAYDFRLIQHVVNTRYRTTNEIANYMAYLGNKYPNIAEFRTEYDDT
SMLLHSLKITDQIGSSEEKKFH
>Locus_8734_Transcript_2/5_Confidence_0.692_Length_2062|.|sp|P42787|CBPD_DROME Carboxypeptidase D OS=Drosophila melanogaster GN=svr PE=1 SV=2|e=1.038E-166|from=8|to=2059|frame=2|Fragment_4
SEG
>Locus_8734_Transcript_2/5_Confidence_0.692_Length_2062|.|sp|P42787|CBPD_DROME Carboxypeptidase D OS=Drosophila melanogaster GN=svr PE=1 SV=2|e=1.038E-166|from=8|to=2059|frame=2|Fragment_5
VGSEMVLNTAEHLMAAFEIGEHKHIQILRNAVVHVIPVLDDNFDSIIQNYRLHEDVCNPE
SFKQEFGERMIASNADKLVTAFKKLLWFGRYDFILNFGSGGCGLSYPRVNDSGFIYDKLL
NNMHNLINSTTSHETCCNNQGNQNHAEITERLTNLMYNEYNVPIVTVRNSCCKMPRQEQL
ANVWRNSLKPLMSFLELIETGIQGAILGNTMNPLRHAVVTLKQFNRTFNVTRNLAYYKIM
LPVGAYNV
>Locus_8734_Transcript_1/5_Confidence_0.692_Length_2062|.|sp|P42787|CBPD_DROME Carboxypeptidase D OS=Drosophila melanogaster GN=svr PE=1 SV=2|e=2.3124E-166|from=8|to=2059|frame=2|Fragment_1
SIGQSVKGHYLWVLEISANPGEHITGKPSIKYVGNMHGNEVVGREILLLFAKFLCENYLI
DEQVTRIVNTTRIHLLPSMNPDGYDMAKPEEINNVVGRNNAHNIDLNRNFPDQYRS
>Locus_8734_Transcript_1/5_Confidence_0.692_Length_2062|.|sp|P42787|CBPD_DROME Carboxypeptidase D OS=Drosophila melanogaster GN=svr PE=1 SV=2|e=2.3124E-166|from=8|to=2059|frame=2|Fragment_2
LLNVKPEPETEAVMAWTLSERFVLSANLHNGALVANYPFDENPSNI
>Locus_8734_Transcript_1/5_Confidence_0.692_Length_2062|.|sp|P42787|CBPD_DROME Carboxypeptidase D OS=Drosophila melanogaster GN=svr PE=1 SV=2|e=2.3124E-166|from=8|to=2059|frame=2|Fragment_3
ILQKVANPSPDDDVFQHLARIYSNAHRKMHLGQPCAEHPKEHFLNGITNGAAWYSVSGSM
QDWNYVEADCFEITLELGCVKYPGAEALPELWLDNKMALLDYIELAHIGVRGFVHSSIGN
PISSAEVLVKGRKHITKTGSKGEYHRLLLPGSYNITVSSYGYESQTVQIIVPDVKPYSVG
YNFTLMRDDPQHWSSAYDFRLIQHVVNTRYRTTNEIANYMAYLGNKYPNIAEFRTEYDDT

SMLLHSLKITDQIGSSEEKKFH
>Locus_8734_Transcript_1/5_Confidence_0.692_Length_2062|.|sp|P42787|
CBPD_DROME Carboxypeptidase D OS=Drosophila melanogaster GN=svr PE=1
SV=2|e=2.3124E-166|from=8|to=2059|frame=2|Fragment_4
SEG
>Locus_8734_Transcript_1/5_Confidence_0.692_Length_2062|.|sp|P42787|
CBPD_DROME Carboxypeptidase D OS=Drosophila melanogaster GN=svr PE=1
SV=2|e=2.3124E-166|from=8|to=2059|frame=2|Fragment_5
VGSEMVLNTAEHLMAAFEIGEHKHIQILRNAVVHVIPVLDDNFDSIIQNYRLHEDVCNPE
SFKQEFGERMIASNADKLVTAFKKLLWFGRYDFILNFGSGGCGLSYPRVNDSGFIYDKLL
NNMHNLINSTTSHETCCNNQGNQNHAEITERLTNLMYNEYNVPIVTVRNSCCKMPRQEQL
ANVWRNSLKPLMSFLELIETGIQGAILGNTMNPLRHAVVTLKQFNRTFNVTRNLAYYKIM
LPVGAYNV
>Locus_8734_Transcript_5/5_Confidence_0.692_Length_2062|.|sp|P42787|
CBPD_DROME Carboxypeptidase D OS=Drosophila melanogaster GN=svr PE=1
SV=2|e=2.3124E-166|from=8|to=2059|frame=2|Fragment_1
SIGQSVKGHYLWVLEISANPGEHITGKPSIKYVGNMHGNEVVGREILLLFAKFLCENYLI
DEQVTRIVNTTRIHLLPSMNPDGYDMAKPEEINNVVGRNNAHNIDLNRNFPDQYRS
>Locus_8734_Transcript_5/5_Confidence_0.692_Length_2062|.|sp|P42787|
CBPD_DROME Carboxypeptidase D OS=Drosophila melanogaster GN=svr PE=1
SV=2|e=2.3124E-166|from=8|to=2059|frame=2|Fragment_2
LLNVKPEPETEAVMAWTLSERFVLSANLHNGALVANYPFDENPSNI
>Locus_8734_Transcript_5/5_Confidence_0.692_Length_2062|.|sp|P42787|
CBPD_DROME Carboxypeptidase D OS=Drosophila melanogaster GN=svr PE=1
SV=2|e=2.3124E-166|from=8|to=2059|frame=2|Fragment_3
ILQKVANPSPDDDVFQHLARIYSNAHRKMHLGQPCAEHPKEHFLNGITNGAAWYSVSGSM
QDWNYVEADCFEITLELGCVKYPGAEALPELWLDNKMALLDYIELAHIGVRGFVHSSIGN
PISSAEVLVKGRKHITKTGSKGEYHRLLLPGSYNITVSSYGYESQTVQIIVPDVKPYSVG
YNFTLMRDDPQHWSSAYDFRLIQHVVNTRYRTTNEIANYMAYLGNKYPNIAEFRTEYDDT
SMLLHSLKITDQIGSSEEKKFH
>Locus_8734_Transcript_5/5_Confidence_0.692_Length_2062|.|sp|P42787|
CBPD_DROME Carboxypeptidase D OS=Drosophila melanogaster GN=svr PE=1
SV=2|e=2.3124E-166|from=8|to=2059|frame=2|Fragment_4
SEG
>Locus_8734_Transcript_5/5_Confidence_0.692_Length_2062|.|sp|P42787|
CBPD_DROME Carboxypeptidase D OS=Drosophila melanogaster GN=svr PE=1
SV=2|e=2.3124E-166|from=8|to=2059|frame=2|Fragment_5
VGSEMVLNTAEHLMAAFEIGEHKHIQILRNAVVHVIPVLDDNFDSIIQNYRLHEDVCNPE
SFKQEFGERMIASNADKLVTAFKKLLWFGRYDFILNFGSGGCGLSYPRVNDSGFIYDKLL
NNMHNLINSTTSHETCCNNQGNQNHAEITERLTNLMYNEYNVPIVTVRNSCCKMPRQEQL
ANVWRNSLKPLMSFLELIETGIQGAILGNTMNPLRHAVVTLKQFNRTFNVTRNLAYYKIM
LPVGAYNV
>Locus_8734_Transcript_3/5_Confidence_0.692_Length_2062|.|sp|P42787|
CBPD_DROME Carboxypeptidase D OS=Drosophila melanogaster GN=svr PE=1
SV=2|e=3.0201E-166|from=8|to=2059|frame=2|Fragment_1
SIGQSVKGHYLWVLEISANPGEHITGKPSIKYVGNMHGNEVVGREILLLFAKFLCENYLI
DEQVTRIVNTTRIHLLPSMNPDGYDMAKPEEINNVVGRNNAHNIDLNRNFPDQYRS
>Locus_8734_Transcript_3/5_Confidence_0.692_Length_2062|.|sp|P42787|
CBPD_DROME Carboxypeptidase D OS=Drosophila melanogaster GN=svr PE=1
SV=2|e=3.0201E-166|from=8|to=2059|frame=2|Fragment_2
LLNVKPEPETEAVMAWTLSERFVLSANLHNGALVANYPFDENPSNI
>Locus_8734_Transcript_3/5_Confidence_0.692_Length_2062|.|sp|P42787|
CBPD_DROME Carboxypeptidase D OS=Drosophila melanogaster GN=svr PE=1
SV=2|e=3.0201E-166|from=8|to=2059|frame=2|Fragment_3

ILQKVANPSPDDDVFQHLARIYSNAHRKMHLGQPCAEHPKEHFLNGITNGAAWYSVSGSM
QDWNYVEADCFEITLELGCVKYPGAEALPELWLDNKMALLDYIELAHIGVRGFVHSSIGN
PISSAEVLVKGRKHITKTGSKGEYHRLLLPGSYNITVSSYGYESQTVQIIVPDVKPYSVG
YNFTLMRDDPQHWSSAYDFRLIQHVVNTRYRTTNEIANYMAYLGNKYPNIAEFRTEYDDT
SMLLHSLKITDQIGSSEEKKFH
>Locus_8734_Transcript_3/5_Confidence_0.692_Length_2062|.|sp|P42787|
CBPD_DROME Carboxypeptidase D OS=Drosophila melanogaster GN=svr PE=1
SV=2|e=3.0201E-166|from=8|to=2059|frame=2|Fragment_4
SEG
>Locus_8734_Transcript_3/5_Confidence_0.692_Length_2062|.|sp|P42787|
CBPD_DROME Carboxypeptidase D OS=Drosophila melanogaster GN=svr PE=1
SV=2|e=3.0201E-166|from=8|to=2059|frame=2|Fragment_5
VGSEMVLNTAEHLMAAFEIGEHKHIQILRNAVVHVIPVLDDNFDSIIQNYRLHEDVCNPE
SFKQEFGERMIASNADKLVTAFKKLLWFGRYDFILNFGSGGCGLSYPRVNDSGFIYDKLL
NNMHNLINSTTSHETCCNNQGNQNHAEITERLTNLMYNEYNVPIVTVRNSCCKMPRQEQL
ANVWRNSLKPLMSFLELIETGIEGTILGNTLNPLRHAVVTLKQFNRTFNVTRNLAYYKIM
LPVGAYNV
>Locus_8734_Transcript_2/5_Confidence_0.692_Length_2062|.|sp|P42787|
CBPD_DROME Carboxypeptidase D OS=Drosophila melanogaster GN=svr PE=1
SV=2|e=1.48581E-80|from=11|to=2062|frame=2|Fragment_1
IGQSVKGHYLWVLEISANPGEHITGKPSIKYVGNMHGNEVVGREILLLFAKFLCENYLID
EQVTRIVNTTRIHLLPSMNPDGYDMAKP
>Locus_8734_Transcript_2/5_Confidence_0.692_Length_2062|.|sp|P42787|
CBPD_DROME Carboxypeptidase D OS=Drosophila melanogaster GN=svr PE=1
SV=2|e=1.48581E-80|from=11|to=2062|frame=2|Fragment_2
EEINNVVGRNNAHNIDLNRNFPDQYRS
>Locus_8734_Transcript_2/5_Confidence_0.692_Length_2062|.|sp|P42787|
CBPD_DROME Carboxypeptidase D OS=Drosophila melanogaster GN=svr PE=1
SV=2|e=1.48581E-80|from=11|to=2062|frame=2|Fragment_3
LLNVKPEPETEAVMAWTLSERFVLSANLHNGALVANYPFDENPSNIILQKVANPSPDDDV
FQHLARIYSNAHRKMHLGQPCAEHPKEHFLNGITNGAAWYSVSGSMQDWNYVEADCFEIT
LELGCVKYPRAEALPELWLDNKMALLDYIELAHIGVRGFVHSSIGNPISSAEVLVKG
>Locus_8734_Transcript_2/5_Confidence_0.692_Length_2062|.|sp|P42787|
CBPD_DROME Carboxypeptidase D OS=Drosophila melanogaster GN=svr PE=1
SV=2|e=1.48581E-80|from=11|to=2062|frame=2|Fragment_4
RKHITRTGSKGEYHRLLLPGLYNITVSSYGYE
>Locus_8734_Transcript_2/5_Confidence_0.692_Length_2062|.|sp|P42787|
CBPD_DROME Carboxypeptidase D OS=Drosophila melanogaster GN=svr PE=1
SV=2|e=1.48581E-80|from=11|to=2062|frame=2|Fragment_5
SQTVQIIVPDVKPYSVGYNFTLMRDDPQHWSSAYDFRLIQ
>Locus_8734_Transcript_2/5_Confidence_0.692_Length_2062|.|sp|P42787|
CBPD_DROME Carboxypeptidase D OS=Drosophila melanogaster GN=svr PE=1
SV=2|e=1.48581E-80|from=11|to=2062|frame=2|Fragment_6
HVVNTRYRTTNEIA
>Locus_8734_Transcript_2/5_Confidence_0.692_Length_2062|.|sp|P42787|
CBPD_DROME Carboxypeptidase D OS=Drosophila melanogaster GN=svr PE=1
SV=2|e=1.48581E-80|from=11|to=2062|frame=2|Fragment_7
NYMAYLGNKYPNIAE
>Locus_8734_Transcript_2/5_Confidence_0.692_Length_2062|.|sp|P42787|
CBPD_DROME Carboxypeptidase D OS=Drosophila melanogaster GN=svr PE=1
SV=2|e=1.48581E-80|from=11|to=2062|frame=2|Fragment_8
FRTEYDDTSMLLHSLKITDQIGSSEEKKFH
>Locus_8734_Transcript_2/5_Confidence_0.692_Length_2062|.|sp|P42787|
CBPD_DROME Carboxypeptidase D OS=Drosophila melanogaster GN=svr PE=1

SV=2|e=1.48581E-80|from=11|to=2062|frame=2|Fragment_9
SEGVGSEMVLNTAEHLMAAFEIGEHKHIQILRNAVVHVIPVLD
>Locus_8734_Transcript_2/5_Confidence_0.692_Length_2062|.|sp|P42787|
CBPD_DROME Carboxypeptidase D OS=Drosophila melanogaster GN=svr PE=1
SV=2|e=1.48581E-80|from=11|to=2062|frame=2|Fragment_10
DNFDSIIQNYRL
>Locus_8734_Transcript_2/5_Confidence_0.692_Length_2062|.|sp|P42787|
CBPD_DROME Carboxypeptidase D OS=Drosophila melanogaster GN=svr PE=1
SV=2|e=1.48581E-80|from=11|to=2062|frame=2|Fragment_11
HEDVCNPESFKQEFGERMIASNADKLVTAFKKLLWFGRYDFIL
>Locus_8734_Transcript_2/5_Confidence_0.692_Length_2062|.|sp|P42787|
CBPD_DROME Carboxypeptidase D OS=Drosophila melanogaster GN=svr PE=1
SV=2|e=1.48581E-80|from=11|to=2062|frame=2|Fragment_12
NFGSGGCGLSYPRVNDSGFIYDKLLNNMHNLINS
>Locus_8734_Transcript_2/5_Confidence_0.692_Length_2062|.|sp|P42787|
CBPD_DROME Carboxypeptidase D OS=Drosophila melanogaster GN=svr PE=1
SV=2|e=1.48581E-80|from=11|to=2062|frame=2|Fragment_13
TTSHETCCNNQ
>Locus_8734_Transcript_2/5_Confidence_0.692_Length_2062|.|sp|P42787|
CBPD_DROME Carboxypeptidase D OS=Drosophila melanogaster GN=svr PE=1
SV=2|e=1.48581E-80|from=11|to=2062|frame=2|Fragment_14
GNQNHAE
>Locus_8734_Transcript_2/5_Confidence_0.692_Length_2062|.|sp|P42787|
CBPD_DROME Carboxypeptidase D OS=Drosophila melanogaster GN=svr PE=1
SV=2|e=1.48581E-80|from=11|to=2062|frame=2|Fragment_15
ITERLTNLMYNEYNVPIVTVRNSCCKMPRQEQLANVWRNSLKPLMSFLELIETGIQGAIL
GNTMNPLRHAVVTLKQFNRTFNVTRNLAYYKIMLPVGAYNVT
>Locus_8734_Transcript_4/5_Confidence_0.692_Length_2062|.|sp|P42787|
CBPD_DROME Carboxypeptidase D OS=Drosophila melanogaster GN=svr PE=1
SV=2|e=4.32303E-80|from=11|to=2062|frame=2|Fragment_1
IGQSVKGHYLWVLEISANPGEHITGKPSIKYVGNMHGNEVVGREILLLFAKFLCENYLID
EQVTRIVNTTRIHLLPSMNPDGYDMAKP
>Locus_8734_Transcript_4/5_Confidence_0.692_Length_2062|.|sp|P42787|
CBPD_DROME Carboxypeptidase D OS=Drosophila melanogaster GN=svr PE=1
SV=2|e=4.32303E-80|from=11|to=2062|frame=2|Fragment_2
EEINNVVGRNNAHNIDLNRNFPDQYRS
>Locus_8734_Transcript_4/5_Confidence_0.692_Length_2062|.|sp|P42787|
CBPD_DROME Carboxypeptidase D OS=Drosophila melanogaster GN=svr PE=1
SV=2|e=4.32303E-80|from=11|to=2062|frame=2|Fragment_3
LLNVKPEPETEAVMAWTLSERFVLSANLHNGALVANYPFDENPSNIILQKVANPSPDDDV
FQHLARIYSNAHRKMHLGQPCAEHPKEHFLNGITNGAAWYSVSGSMQDWNYVEADCFEIT
LELGCVKYPGAEALPELWLDNKMALLDYIELAHIGVRGFVHSSIGNPISSAEVLVKG
>Locus_8734_Transcript_4/5_Confidence_0.692_Length_2062|.|sp|P42787|
CBPD_DROME Carboxypeptidase D OS=Drosophila melanogaster GN=svr PE=1
SV=2|e=4.32303E-80|from=11|to=2062|frame=2|Fragment_4
RKHITKTGSKGEYHRLLLPGSYNITVSSYGYE
>Locus_8734_Transcript_4/5_Confidence_0.692_Length_2062|.|sp|P42787|
CBPD_DROME Carboxypeptidase D OS=Drosophila melanogaster GN=svr PE=1
SV=2|e=4.32303E-80|from=11|to=2062|frame=2|Fragment_5
SQTVQIIVPDVKPYSVGYNFTLMRDDPQHWSSAYDFRLIQ
>Locus_8734_Transcript_4/5_Confidence_0.692_Length_2062|.|sp|P42787|
CBPD_DROME Carboxypeptidase D OS=Drosophila melanogaster GN=svr PE=1
SV=2|e=4.32303E-80|from=11|to=2062|frame=2|Fragment_6
HVVNTRYRTTNEIA

>Locus_8734_Transcript_4/5_Confidence_0.692_Length_2062|.|sp|P42787|
CBPD_DROME Carboxypeptidase D OS=Drosophila melanogaster GN=svr PE=1
SV=2|e=4.32303E-80|from=11|to=2062|frame=2|Fragment_7
NYMAYLGNKYPNIAE
>Locus_8734_Transcript_4/5_Confidence_0.692_Length_2062|.|sp|P42787|
CBPD_DROME Carboxypeptidase D OS=Drosophila melanogaster GN=svr PE=1
SV=2|e=4.32303E-80|from=11|to=2062|frame=2|Fragment_8
FRTEYDDTSMLLHSLKITDQIGSSEEKKFH
>Locus_8734_Transcript_4/5_Confidence_0.692_Length_2062|.|sp|P42787|
CBPD_DROME Carboxypeptidase D OS=Drosophila melanogaster GN=svr PE=1
SV=2|e=4.32303E-80|from=11|to=2062|frame=2|Fragment_9
SEGVGSEMVLNTAEHLMAAFKIGEQEHMQILRNAV
>Locus_8734_Transcript_4/5_Confidence_0.692_Length_2062|.|sp|P42787|
CBPD_DROME Carboxypeptidase D OS=Drosophila melanogaster GN=svr PE=1
SV=2|e=4.32303E-80|from=11|to=2062|frame=2|Fragment_10
VHVIPVLD
>Locus_8734_Transcript_4/5_Confidence_0.692_Length_2062|.|sp|P42787|
CBPD_DROME Carboxypeptidase D OS=Drosophila melanogaster GN=svr PE=1
SV=2|e=4.32303E-80|from=11|to=2062|frame=2|Fragment_11
DNFDSIIQNYRL
>Locus_8734_Transcript_4/5_Confidence_0.692_Length_2062|.|sp|P42787|
CBPD_DROME Carboxypeptidase D OS=Drosophila melanogaster GN=svr PE=1
SV=2|e=4.32303E-80|from=11|to=2062|frame=2|Fragment_12
HEDVCNPESFKQEFGERMIASNADKLVTAFKKLLWSGRYDFIL
>Locus_8734_Transcript_4/5_Confidence_0.692_Length_2062|.|sp|P42787|
CBPD_DROME Carboxypeptidase D OS=Drosophila melanogaster GN=svr PE=1
SV=2|e=4.32303E-80|from=11|to=2062|frame=2|Fragment_13
NFGSGGCGLSYPRVNDSGFIYDKLLNNMHNLINS
>Locus_8734_Transcript_4/5_Confidence_0.692_Length_2062|.|sp|P42787|
CBPD_DROME Carboxypeptidase D OS=Drosophila melanogaster GN=svr PE=1
SV=2|e=4.32303E-80|from=11|to=2062|frame=2|Fragment_14
TTSHETCCNNQ
>Locus_8734_Transcript_4/5_Confidence_0.692_Length_2062|.|sp|P42787|
CBPD_DROME Carboxypeptidase D OS=Drosophila melanogaster GN=svr PE=1
SV=2|e=4.32303E-80|from=11|to=2062|frame=2|Fragment_15
GNQNHAE
>Locus_8734_Transcript_4/5_Confidence_0.692_Length_2062|.|sp|P42787|
CBPD_DROME Carboxypeptidase D OS=Drosophila melanogaster GN=svr PE=1
SV=2|e=4.32303E-80|from=11|to=2062|frame=2|Fragment_16
ITERLTNLMYNEYNVPIVTVRNSCCKMPRQEQLANVWRNSLKPLMSFLELIETGIQGAIL
GNTMNPLRHAVVTLKQFNRTFNVTRNLAYYKIMLPVGAYNVT
>Locus_8734_Transcript_1/5_Confidence_0.692_Length_2062|.|sp|P42787|
CBPD_DROME Carboxypeptidase D OS=Drosophila melanogaster GN=svr PE=1
SV=2|e=1.25781E-79|from=11|to=2062|frame=2|Fragment_1
IGQSVKGHYLWVLEISANPGEHITGKPSIKYVGNMHGNEVVGREILLLFAKFLCENYLID
EQVTRIVNTTRIHLLPSMNPDGYDMAKP
>Locus_8734_Transcript_1/5_Confidence_0.692_Length_2062|.|sp|P42787|
CBPD_DROME Carboxypeptidase D OS=Drosophila melanogaster GN=svr PE=1
SV=2|e=1.25781E-79|from=11|to=2062|frame=2|Fragment_2
EEINNVVGRNNAHNIDLNRNFPDQYRS
>Locus_8734_Transcript_1/5_Confidence_0.692_Length_2062|.|sp|P42787|
CBPD_DROME Carboxypeptidase D OS=Drosophila melanogaster GN=svr PE=1
SV=2|e=1.25781E-79|from=11|to=2062|frame=2|Fragment_3
LLNVKPEPETEAVMAWTLSERFVLSANLHNGALVANYPFDENPSNIILQKVANPSPDDDV

FQHLARIYSNAHRKMHLGQPCAEHPKEHFLNGITNGAAWYSVSGSMQDWNYVEADCFEIT
LELGCVKYPGAEALPELWLDNKMALLDYIELAHIGVRGFVHSSIGNPISSAEVLVKG
>Locus_8734_Transcript_1/5_Confidence_0.692_Length_2062|.|sp|P42787|
CBPD_DROME Carboxypeptidase D OS=Drosophila melanogaster GN=svr PE=1
SV=2|e=1.25781E-79|from=11|to=2062|frame=2|Fragment_4
RKHITKTGSKGEYHRLLLPGSYNITVSSYGYE
>Locus_8734_Transcript_1/5_Confidence_0.692_Length_2062|.|sp|P42787|
CBPD_DROME Carboxypeptidase D OS=Drosophila melanogaster GN=svr PE=1
SV=2|e=1.25781E-79|from=11|to=2062|frame=2|Fragment_5
SQTVQIIVPDVKPYSVGYNFTLMRDDPQHWSSAYDFRLIQ
>Locus_8734_Transcript_1/5_Confidence_0.692_Length_2062|.|sp|P42787|
CBPD_DROME Carboxypeptidase D OS=Drosophila melanogaster GN=svr PE=1
SV=2|e=1.25781E-79|from=11|to=2062|frame=2|Fragment_6
HVVNTRYRTTNEIA
>Locus_8734_Transcript_1/5_Confidence_0.692_Length_2062|.|sp|P42787|
CBPD_DROME Carboxypeptidase D OS=Drosophila melanogaster GN=svr PE=1
SV=2|e=1.25781E-79|from=11|to=2062|frame=2|Fragment_7
NYMAYLGNKYPNIAE
>Locus_8734_Transcript_1/5_Confidence_0.692_Length_2062|.|sp|P42787|
CBPD_DROME Carboxypeptidase D OS=Drosophila melanogaster GN=svr PE=1
SV=2|e=1.25781E-79|from=11|to=2062|frame=2|Fragment_8
FRTEYDDTSMLLHSLKITDQIGSSEEKKFH
>Locus_8734_Transcript_1/5_Confidence_0.692_Length_2062|.|sp|P42787|
CBPD_DROME Carboxypeptidase D OS=Drosophila melanogaster GN=svr PE=1
SV=2|e=1.25781E-79|from=11|to=2062|frame=2|Fragment_9
SEGVGSEMVLNTAEHLMAAFEIGEHKHIQILRNAVVHVIPVLD
>Locus_8734_Transcript_1/5_Confidence_0.692_Length_2062|.|sp|P42787|
CBPD_DROME Carboxypeptidase D OS=Drosophila melanogaster GN=svr PE=1
SV=2|e=1.25781E-79|from=11|to=2062|frame=2|Fragment_10
DNFDSIIQNYRL
>Locus_8734_Transcript_1/5_Confidence_0.692_Length_2062|.|sp|P42787|
CBPD_DROME Carboxypeptidase D OS=Drosophila melanogaster GN=svr PE=1
SV=2|e=1.25781E-79|from=11|to=2062|frame=2|Fragment_11
HEDVCNPESFKQEFGERMIASNADKLVTAFKKLLWFGRYDFIL
>Locus_8734_Transcript_1/5_Confidence_0.692_Length_2062|.|sp|P42787|
CBPD_DROME Carboxypeptidase D OS=Drosophila melanogaster GN=svr PE=1
SV=2|e=1.25781E-79|from=11|to=2062|frame=2|Fragment_12
NFGSGGCGLSYPRVNDSGFIYDKLLNNMHNLINS
>Locus_8734_Transcript_1/5_Confidence_0.692_Length_2062|.|sp|P42787|
CBPD_DROME Carboxypeptidase D OS=Drosophila melanogaster GN=svr PE=1
SV=2|e=1.25781E-79|from=11|to=2062|frame=2|Fragment_13
TTSHETCCNNQ
>Locus_8734_Transcript_1/5_Confidence_0.692_Length_2062|.|sp|P42787|
CBPD_DROME Carboxypeptidase D OS=Drosophila melanogaster GN=svr PE=1
SV=2|e=1.25781E-79|from=11|to=2062|frame=2|Fragment_14
GNQNHAE
>Locus_8734_Transcript_1/5_Confidence_0.692_Length_2062|.|sp|P42787|
CBPD_DROME Carboxypeptidase D OS=Drosophila melanogaster GN=svr PE=1
SV=2|e=1.25781E-79|from=11|to=2062|frame=2|Fragment_15
ITERLTNLMYNEYNVPIVTVRNSCCKMPRQEQLANVWRNSLKPLMSFLELIETGIQGAIL
GNTMNPLRHAVVTLKQFNRTFNVTRNLAYYKIMLPVGAYNVT
>Locus_8734_Transcript_5/5_Confidence_0.692_Length_2062|.|sp|P42787|
CBPD_DROME Carboxypeptidase D OS=Drosophila melanogaster GN=svr PE=1
SV=2|e=1.25781E-79|from=11|to=2062|frame=2|Fragment_1

IGQSVKGHYLWVLEISANPGEHITGKPSIKYVGNMHGNEVVGREILLLFAKFLCENYLID
EQVTRIVNTTRIHLLPSMNPDGYDMAKP
>Locus_8734_Transcript_5/5_Confidence_0.692_Length_2062|.|sp|P42787|
CBPD_DROME Carboxypeptidase D OS=Drosophila melanogaster GN=svr PE=1
SV=2|e=1.25781E-79|from=11|to=2062|frame=2|Fragment_2
EEINNVVGRNNAHNIDLNRNFPDQYRS
>Locus_8734_Transcript_5/5_Confidence_0.692_Length_2062|.|sp|P42787|
CBPD_DROME Carboxypeptidase D OS=Drosophila melanogaster GN=svr PE=1
SV=2|e=1.25781E-79|from=11|to=2062|frame=2|Fragment_3
LLNVKPEPETEAVMAWTLSERFVLSANLHNGALVANYPFDENPSNIILQKVANPSPDDDV
FQHLARIYSNAHRKMHLGQPCAEHPKEHFLNGITNGAAWYSVSGSMQDWNYVEADCFEIT
LELGCVKYPGAEALPELWLDNKMALLDYIELAHIGVRGFVHSSIGNPISSAEVLVKG
>Locus_8734_Transcript_5/5_Confidence_0.692_Length_2062|.|sp|P42787|
CBPD_DROME Carboxypeptidase D OS=Drosophila melanogaster GN=svr PE=1
SV=2|e=1.25781E-79|from=11|to=2062|frame=2|Fragment_4
RKHITKTGSKGEYHRLLLPGSYNITVSSYGYE
>Locus_8734_Transcript_5/5_Confidence_0.692_Length_2062|.|sp|P42787|
CBPD_DROME Carboxypeptidase D OS=Drosophila melanogaster GN=svr PE=1
SV=2|e=1.25781E-79|from=11|to=2062|frame=2|Fragment_5
SQTVQIIVPDVKPYSVGYNFTLMRDDPQHWSSAYDFRLIQ
>Locus_8734_Transcript_5/5_Confidence_0.692_Length_2062|.|sp|P42787|
CBPD_DROME Carboxypeptidase D OS=Drosophila melanogaster GN=svr PE=1
SV=2|e=1.25781E-79|from=11|to=2062|frame=2|Fragment_6
HVVNTRYRTTNEIA
>Locus_8734_Transcript_5/5_Confidence_0.692_Length_2062|.|sp|P42787|
CBPD_DROME Carboxypeptidase D OS=Drosophila melanogaster GN=svr PE=1
SV=2|e=1.25781E-79|from=11|to=2062|frame=2|Fragment_7
NYMAYLGNKYPNIAE
>Locus_8734_Transcript_5/5_Confidence_0.692_Length_2062|.|sp|P42787|
CBPD_DROME Carboxypeptidase D OS=Drosophila melanogaster GN=svr PE=1
SV=2|e=1.25781E-79|from=11|to=2062|frame=2|Fragment_8
FRTEYDDTSMLLHSLKITDQIGSSEEKKFH
>Locus_8734_Transcript_5/5_Confidence_0.692_Length_2062|.|sp|P42787|
CBPD_DROME Carboxypeptidase D OS=Drosophila melanogaster GN=svr PE=1
SV=2|e=1.25781E-79|from=11|to=2062|frame=2|Fragment_9
SEGVGSEMVLNTAEHLMAAFEIGEHKHIQILRNAVVHVIPVLD
>Locus_8734_Transcript_5/5_Confidence_0.692_Length_2062|.|sp|P42787|
CBPD_DROME Carboxypeptidase D OS=Drosophila melanogaster GN=svr PE=1
SV=2|e=1.25781E-79|from=11|to=2062|frame=2|Fragment_10
DNFDSIIQNYRL
>Locus_8734_Transcript_5/5_Confidence_0.692_Length_2062|.|sp|P42787|
CBPD_DROME Carboxypeptidase D OS=Drosophila melanogaster GN=svr PE=1
SV=2|e=1.25781E-79|from=11|to=2062|frame=2|Fragment_11
HEDVCNPESFKQEFGERMIASNADKLVTAFKKLLWFGRYDFIL
>Locus_8734_Transcript_5/5_Confidence_0.692_Length_2062|.|sp|P42787|
CBPD_DROME Carboxypeptidase D OS=Drosophila melanogaster GN=svr PE=1
SV=2|e=1.25781E-79|from=11|to=2062|frame=2|Fragment_12
NFGSGGCGLSYPRVNDSGFIYDKLLNNMHNLINS
>Locus_8734_Transcript_5/5_Confidence_0.692_Length_2062|.|sp|P42787|
CBPD_DROME Carboxypeptidase D OS=Drosophila melanogaster GN=svr PE=1
SV=2|e=1.25781E-79|from=11|to=2062|frame=2|Fragment_13
TTSHETCCNNQ
>Locus_8734_Transcript_5/5_Confidence_0.692_Length_2062|.|sp|P42787|
CBPD_DROME Carboxypeptidase D OS=Drosophila melanogaster GN=svr PE=1

SV=2|e=1.25781E-79|from=11|to=2062|frame=2|Fragment_14
GNQNHAE
>Locus_8734_Transcript_5/5_Confidence_0.692_Length_2062|.|sp|P42787|
CBPD_DROME Carboxypeptidase D OS=Drosophila melanogaster GN=svr PE=1
SV=2|e=1.25781E-79|from=11|to=2062|frame=2|Fragment_15
ITERLTNLMYNEYNVPIVTVRNSCCKMPRQEQLANVWRNSLKPLMSFLELIETGIQGAIL
GNTMNPLRHAVVTLKQFNRTFNVTRNLAYYKIMLPVGAYNVT
>Locus_8734_Transcript_3/5_Confidence_0.692_Length_2062|.|sp|P42787|
CBPD_DROME Carboxypeptidase D OS=Drosophila melanogaster GN=svr PE=1
SV=2|e=1.64275E-79|from=11|to=2062|frame=2|Fragment_1
IGQSVKGHYLWVLEISANPGEHITGKPSIKYVGNMHGNEVVGREILLLFAKFLCENYLID
EQVTRIVNTTRIHLLPSMNPDGYDMAKP
>Locus_8734_Transcript_3/5_Confidence_0.692_Length_2062|.|sp|P42787|
CBPD_DROME Carboxypeptidase D OS=Drosophila melanogaster GN=svr PE=1
SV=2|e=1.64275E-79|from=11|to=2062|frame=2|Fragment_2
EEINNVVGRNNAHNIDLNRNFPDQYRS
>Locus_8734_Transcript_3/5_Confidence_0.692_Length_2062|.|sp|P42787|
CBPD_DROME Carboxypeptidase D OS=Drosophila melanogaster GN=svr PE=1
SV=2|e=1.64275E-79|from=11|to=2062|frame=2|Fragment_3
LLNVKPEPETEAVMAWTLSERFVLSANLHNGALVANYPFDENPSNIILQKVANPSPDDDV
FQHLARIYSNAHRKMHLGQPCAEHPKEHFLNGITNGAAWYSVSGSMQDWNYVEADCFEIT
LELGCVKYPGAEALPELWLDNKMALLDYIELAHIGVRGFVHSSIGNPISSAEVLVKG
>Locus_8734_Transcript_3/5_Confidence_0.692_Length_2062|.|sp|P42787|
CBPD_DROME Carboxypeptidase D OS=Drosophila melanogaster GN=svr PE=1
SV=2|e=1.64275E-79|from=11|to=2062|frame=2|Fragment_4
RKHITKTGSKGEYHRLLLPGSYNITVSSYGYE
>Locus_8734_Transcript_3/5_Confidence_0.692_Length_2062|.|sp|P42787|
CBPD_DROME Carboxypeptidase D OS=Drosophila melanogaster GN=svr PE=1
SV=2|e=1.64275E-79|from=11|to=2062|frame=2|Fragment_5
SQTVQIIVPDVKPYSVGYNFTLMRDDPQHWSSAYDFRLIQ
>Locus_8734_Transcript_3/5_Confidence_0.692_Length_2062|.|sp|P42787|
CBPD_DROME Carboxypeptidase D OS=Drosophila melanogaster GN=svr PE=1
SV=2|e=1.64275E-79|from=11|to=2062|frame=2|Fragment_6
HVVNTRYRTTNEIA
>Locus_8734_Transcript_3/5_Confidence_0.692_Length_2062|.|sp|P42787|
CBPD_DROME Carboxypeptidase D OS=Drosophila melanogaster GN=svr PE=1
SV=2|e=1.64275E-79|from=11|to=2062|frame=2|Fragment_7
NYMAYLGNKYPNIAE
>Locus_8734_Transcript_3/5_Confidence_0.692_Length_2062|.|sp|P42787|
CBPD_DROME Carboxypeptidase D OS=Drosophila melanogaster GN=svr PE=1
SV=2|e=1.64275E-79|from=11|to=2062|frame=2|Fragment_8
FRTEYDDTSMLLHSLKITDQIGSSEEKKFH
>Locus_8734_Transcript_3/5_Confidence_0.692_Length_2062|.|sp|P42787|
CBPD_DROME Carboxypeptidase D OS=Drosophila melanogaster GN=svr PE=1
SV=2|e=1.64275E-79|from=11|to=2062|frame=2|Fragment_9
SEGVGSEMVLNTAEHLMAAFEIGEHKHIQILRNAVVHVIPVLD
>Locus_8734_Transcript_3/5_Confidence_0.692_Length_2062|.|sp|P42787|
CBPD_DROME Carboxypeptidase D OS=Drosophila melanogaster GN=svr PE=1
SV=2|e=1.64275E-79|from=11|to=2062|frame=2|Fragment_10
DNFDSIIQNYRL
>Locus_8734_Transcript_3/5_Confidence_0.692_Length_2062|.|sp|P42787|
CBPD_DROME Carboxypeptidase D OS=Drosophila melanogaster GN=svr PE=1
SV=2|e=1.64275E-79|from=11|to=2062|frame=2|Fragment_11
HEDVCNPESFKQEFGERMIASNADKLVTAFKKLLWFGRYDFIL

>Locus_8734_Transcript_3/5_Confidence_0.692_Length_2062|.|sp|P42787|
CBPD_DROME Carboxypeptidase D OS=Drosophila melanogaster GN=svr PE=1
SV=2|e=1.64275E-79|from=11|to=2062|frame=2|Fragment_12
NFGSGGCGLSYPRVNDSGFIYDKLLNNMHNLINS
>Locus_8734_Transcript_3/5_Confidence_0.692_Length_2062|.|sp|P42787|
CBPD_DROME Carboxypeptidase D OS=Drosophila melanogaster GN=svr PE=1
SV=2|e=1.64275E-79|from=11|to=2062|frame=2|Fragment_13
TTSHETCCNNQ
>Locus_8734_Transcript_3/5_Confidence_0.692_Length_2062|.|sp|P42787|
CBPD_DROME Carboxypeptidase D OS=Drosophila melanogaster GN=svr PE=1
SV=2|e=1.64275E-79|from=11|to=2062|frame=2|Fragment_14
GNQNHAE
>Locus_8734_Transcript_3/5_Confidence_0.692_Length_2062|.|sp|P42787|
CBPD_DROME Carboxypeptidase D OS=Drosophila melanogaster GN=svr PE=1
SV=2|e=1.64275E-79|from=11|to=2062|frame=2|Fragment_15
ITERLTNLMYNEYNVPIVTVRNSCCKMPRQEQLANVWRNSLKPLMSFLELIETGIEGTIL
GNTLNPLRHAVVTLKQFNRTFNVTRNLAYYKIMLPVGAYNVT

>Locus_28664_Transcript_1/1_Confidence_1.000_Length_379|UNIGENE
REPRESENTATIVE TRANSCRIPT|sp|P48994|TRPL_DROME Transient-receptor-
potential-like protein OS=Drosophila melanogaster GN=trpl PE=1 SV=2|
e=2.03619E-57|from=9|to=377|frame=3|Fragment_1
AYVQQIREINRNPDTIYIPREEWDDFDPQLIAEGLFAAANIFSALKLVHLFSINPHLGPL
QISLGRMVIDIIKFFFIYSLVLFAFACGLNQLLWYFADLERSKCYILPGGLPDWANHADA
CMK
>Locus_17161_Transcript_1/1_Confidence_1.000_Length_337|.|sp|P48994|
TRPL_DROME Transient-receptor-potential-like protein OS=Drosophila
melanogaster GN=trpl PE=1 SV=2|e=1.33446E-48|from=3|to=335|frame=3|
Fragment_1
ERSKCYILPGGLPDWANHADACMKWRSFGNLFESTQSLFWASFGMIGLENFELSGIKSYT
RFWGLLMFGSYS
>Locus_17161_Transcript_1/1_Confidence_1.000_Length_337|.|sp|P48994|
TRPL_DROME Transient-receptor-potential-like protein OS=Drosophila
melanogaster GN=trpl PE=1 SV=2|e=1.33446E-48|from=3|to=335|frame=3|
Fragment_2
AMMSNSYAMIDEHSDTEWKFARTRLWM

>Locus_1548_Transcript_2/11_Confidence_0.600_Length_2317|UNIGENE
REPRESENTATIVE TRANSCRIPT|"sp|P53624|MA121_DROME Mannosyl-
oligosaccharide alpha-1,2-mannosidase isoform A OS=Drosophila
melanogaster GN=alpha-Man-I PE=1 SV=2"|e=0|from=597|to=2036|frame=3|
Fragment_1
PSAPTITNGEDSDPET
>Locus_1548_Transcript_2/11_Confidence_0.600_Length_2317|UNIGENE
REPRESENTATIVE TRANSCRIPT|"sp|P53624|MA121_DROME Mannosyl-
oligosaccharide alpha-1,2-mannosidase isoform A OS=Drosophila
melanogaster GN=alpha-Man-I PE=1 SV=2"|e=0|from=597|to=2036|frame=3|
Fragment_2
RARRDKVKEMMRLAWAGYERHAWGANEVRPVSGAAHTSSIFGSASLGATIIDSMDTLLVM
GMTEEYERARKWVEEEFTVDNLNIEVSVFEMNIRFIGGFLACYALTGDELFKEKAKEVAD
KLLPAFQTPTGIPHALVNLKTGESKKYPWTGGFSSILAEFGTLHLEFTYLSDITGDEIYR
DKVQHIRSIIKQIDKVNGLYPNYLNPKTGKWGQHHMSLGALGDSFFEYLLKAWLQSGRQD
AEARVMYDEAMAAITEKMIKRSHQSGLTYVAELKYEKVEHKMDHLACFAGGLYGLGAQTL
QNDVSDSYLLIGEQLAATCHESYDRTPTKLGPESFRFTEAVEARAIKTTEKYYILRPETI

ESYFVLWRLTKNQKYRDWGWEAVQAIEKNCRASGGYTGIKNVYLEDSPKDDVQQSFFLAE
TLKYLYLLFSDDSLIPLDKWVFNTEAHPLPIKGANPLYRSHTAT
>Locus_1548_Transcript_3/11_Confidence_0.600_Length_2298|.|"sp|
P53624|MA121_DROME Mannosyl-oligosaccharide alpha-1,2-mannosidase
isoform A OS=Drosophila melanogaster GN=alpha-Man-I PE=1 SV=2"|e=0|
from=597|to=2036|frame=3|Fragment_1
PSAPTITNGEDSDPET
>Locus_1548_Transcript_3/11_Confidence_0.600_Length_2298|.|"sp|
P53624|MA121_DROME Mannosyl-oligosaccharide alpha-1,2-mannosidase
isoform A OS=Drosophila melanogaster GN=alpha-Man-I PE=1 SV=2"|e=0|
from=597|to=2036|frame=3|Fragment_2
RARRDKVKEMMRLAWAGYERHAWGANEVRPVSGAAHTSSIFGSASLGATIIDSMDTLLVM
GMTEEYERARKWVEEEFTVDNLNIEVSVFEMNIRFIGGFLACYALTGDELFKEKAKEVAD
KLLPAFQTPTGIPHALVNLKTGESKKYPWTGGFSSILAEFGTLHLEFTYLSDITGDEIYR
DKVQHIRSIIKQIDKVNGLYPNYLNPKTGKWGQHHMSLGALGDSFFEYLLKAWLQSGRQD
AEARVMYDEAMAAITEKMIKRSHQSGLTYVAELKYEKVEHKMDHLACFAGGLYGLGAQTL
QNDVSDSYLLIGEQLAATCHESYDRTPTKLGPESFRFTEAVEARAIKTTEKYYILRPETI
ESYFVLWRLTKNQKYRDWGWEAVQAIEKNCRASGGYTGIKNVYLEDSPKDDVQQSFFLAE
TLKYLYLLFSDDSLIPLDKWVFNTEAHPLPIKGANPLYRSHTAT
>Locus_1548_Transcript_4/11_Confidence_0.600_Length_2298|.|"sp|
P53624|MA121_DROME Mannosyl-oligosaccharide alpha-1,2-mannosidase
isoform A OS=Drosophila melanogaster GN=alpha-Man-I PE=1 SV=2"|e=0|
from=597|to=2036|frame=3|Fragment_1
PSAPTITNGEDSDPET
>Locus_1548_Transcript_4/11_Confidence_0.600_Length_2298|.|"sp|
P53624|MA121_DROME Mannosyl-oligosaccharide alpha-1,2-mannosidase
isoform A OS=Drosophila melanogaster GN=alpha-Man-I PE=1 SV=2"|e=0|
from=597|to=2036|frame=3|Fragment_2
RARRDKVKEMMRLAWAGYERHAWGANEVRPVSGAAHTSSIFGSASLGATIIDSMDTLLVM
GMTEEYERARKWVEEEFTVDNLNIEVSVFEMNIRFIGGFLACYALTGDELFKEKAKEVAD
KLLPAFQTPTGIPHALVNLKTGESKKYPWTGGFSSILAEFGTLHLEFTYLSDITGDEIYR
DKVQHIRSIIKQIDKVNGLYPNYLNPKTGKWGQHHMSLGALGDSFFEYLLKAWLQSGRQD
AEARVMYDEAMAAITEKMIKRSHQSGLTYVAELKYEKVEHKMDHLACFAGGLYGLGAQTL
QNDVSDSYLLIGEQLAATCHESYDRTPTKLGPESFRFTEAVEARAIKTTEKYYILRPETI
ESYFVLWRLTKNQKYRDWGWEAVQAIEKNCRASGGYTGIKNVYLEDSPKDDVQQSFFLAE
TLKYLYLLFSDDSLIPLDKWVFNTEAHPLPIKGANPLYRSHTAT
>Locus_1548_Transcript_5/11_Confidence_0.571_Length_2298|.|"sp|
P53624|MA121_DROME Mannosyl-oligosaccharide alpha-1,2-mannosidase
isoform A OS=Drosophila melanogaster GN=alpha-Man-I PE=1 SV=2"|e=0|
from=597|to=2036|frame=3|Fragment_1
PSAPTITNGEDSDPET
>Locus_1548_Transcript_5/11_Confidence_0.571_Length_2298|.|"sp|
P53624|MA121_DROME Mannosyl-oligosaccharide alpha-1,2-mannosidase
isoform A OS=Drosophila melanogaster GN=alpha-Man-I PE=1 SV=2"|e=0|
from=597|to=2036|frame=3|Fragment_2
RARRDKVKEMMRLAWAGYERHAWGANEVRPVSGAAHTSSIFGSASLGATIIDSMDTLLVM
GMTEEYERARKWVEEEFTVDNLNIEVSVFEMNIRFIGGFLACYALTGDELFKEKAKEVAD
KLLPAFQTPTGIPHALVNLKTGESKKYPWTGGFSSILAEFGTLHLEFTYLSDITGDEIYR
DKVQHIRSIIKQIDKVNGLYPNYLNPKTGKWGQHHMSLGALGDSFFEYLLKAWLQSGRQD
AEARVMYDEAMAAITEKMIKRSHQSGLTYVAELKYEKVEHKMDHLACFAGGLYGLGAQTL
QNDVSDSYLLIGEQLAATCHESYDRTPTKLGPESFRFTEAVEARAIKTTEKYYILRPETI
ESYFVLWRLTKNQKYRDWGWEAVQAIEKNCRASGGYTGIKNVYLEDSPKDDVQQSFFLAE
TLKYLYLLFSDDSLIPLDKWVFNTEAHPLPIKGANPLYRSHTAT
>Locus_1548_Transcript_6/11_Confidence_0.600_Length_2298|.|"sp|

P53624|MA121_DROME Mannosyl-oligosaccharide alpha-1,2-mannosidase isoform A OS=Drosophila melanogaster GN=alpha-Man-I PE=1 SV=2"|e=0|from=597|to=2036|frame=3|Fragment_1
PSAPTITNGEDSDPET
>Locus_1548_Transcript_6/11_Confidence_0.600_Length_2298|.|"sp|P53624|MA121_DROME Mannosyl-oligosaccharide alpha-1,2-mannosidase isoform A OS=Drosophila melanogaster GN=alpha-Man-I PE=1 SV=2"|e=0|from=597|to=2036|frame=3|Fragment_2
RARRDKVKEMMRLAWAGYERHAWGANEVRPVSGAAHTSSIFGSASLGATIIDSMDTLLVM
GMTEEYERARKWVEEEFTVDNLNIEVSVFEMNIRFIGGFLACYALTGDELFKEKAKEVAD
KLLPAFQTPTGIPHALVNLKTGESKKYPWTGGFSSILAEFGTLHLEFTYLSDITGDEIYR
DKVQHIRSIIKQIDKVNGLYPNYLNPKTGKWGQHHMSLGALGDSFFEYLLKAWLQSGRQD
AEARVMYDEAMAAITEKMIKRSHQSGLTYVAELKYEKVEHKMDHLACFAGGLYGLGAQTL
QNDVSDSYLLIGEQLAATCHESYDRTPTKLGPESFRFTEAVEARAIKTTEKYYILRPETI
ESYFVLWRLTKNQKYRDWGWEAVQAIEKNCRASGGYTGIKNVYLEDSPKDDVQQSFFLAE
TLKYLYLLFSDDSLIPLDKWVFNTEAHPLPIKGANPLYRSHTAT
>Locus_1548_Transcript_7/11_Confidence_0.543_Length_2298|.|"sp|P53624|MA121_DROME Mannosyl-oligosaccharide alpha-1,2-mannosidase isoform A OS=Drosophila melanogaster GN=alpha-Man-I PE=1 SV=2"|e=0|from=597|to=2036|frame=3|Fragment_1
PSAPTITNGEDSDPET
>Locus_1548_Transcript_7/11_Confidence_0.543_Length_2298|.|"sp|P53624|MA121_DROME Mannosyl-oligosaccharide alpha-1,2-mannosidase isoform A OS=Drosophila melanogaster GN=alpha-Man-I PE=1 SV=2"|e=0|from=597|to=2036|frame=3|Fragment_2
RARRDKVKEMMRLAWAGYERHAWGANEVRPVSGAAHTSSIFGSASLGATIIDSMDTLLVM
GMTEEYERARKWVEEEFTVDNLNIEVSVFEMNIRFIGGFLACYALTGDELFKEKAKEVAD
KLLPAFQTPTGIPHALVNLKTGESKKYPWTGGFSSILAEFGTLHLEFTYLSDITGDEIYR
DKVQHIRSIIKQIDKVNGLYPNYLNPKTGKWGQHHMSLGALGDSFFEYLLKAWLQSGRQD
AEARVMYDEAMAAITEKMIKRSHQSGLTYVAELKYEKVEHKMDHLACFAGGLYGLGAQTL
QNDVSDSYLLIGEQLAATCHESYDRTPTKLGPESFRFTEAVEARAIKTTEKYYILRPETI
ESYFVLWRLTKNQKYRDWGWEAVQAIEKNCRASGGYTGIKNVYLEDSPKDDVQQSFFLAE
TLKYLYLLFSDDSLIPLDKWVFNTEAHPLPIKGANPLYRSHTAT
>Locus_1548_Transcript_8/11_Confidence_0.571_Length_2298|.|"sp|P53624|MA121_DROME Mannosyl-oligosaccharide alpha-1,2-mannosidase isoform A OS=Drosophila melanogaster GN=alpha-Man-I PE=1 SV=2"|e=0|from=597|to=2036|frame=3|Fragment_1
PSAPTITNGEDSDPET
>Locus_1548_Transcript_8/11_Confidence_0.571_Length_2298|.|"sp|P53624|MA121_DROME Mannosyl-oligosaccharide alpha-1,2-mannosidase isoform A OS=Drosophila melanogaster GN=alpha-Man-I PE=1 SV=2"|e=0|from=597|to=2036|frame=3|Fragment_2
RARRDKVKEMMRLAWAGYERHAWGANEVRPVSGAAHTSSIFGSASLGATIIDSMDTLLIM
GMTEEYERARKWVEEEFTVDNLNIEVSVFEMNIRFIGGFLACYALTGDELFKEKAKEVAD
KLLPAFQTPTGIPHALVNLKTGESKKYPWTGGFSSILAEFGTLHLEFTYLSDITGDEIYR
DKVQHIRSIIKQIDKVNGLYPNYLNPKTGKWGQHHMSLGALGDSFFEYLLKAWLQSGRQD
AEARVMYDEAMAAITEKMIKRSHQSGLTYVAELKYEKVEHKMDHLACFAGGLYGLGAQTL
QNDVSDSYLLIGEQLAATCHESYDRTPTKLGPESFRFTEAVEARAIKTTEKYYILRPETI
ESYFVLWRLTKNQKYRDWGWEAVQAIEKNCRASGGYTGIKNVYLEDSPKDDVQQSFFLAE
TLKYLYLLFSDDSLIPLDKWVFNTEAHPLPIKGANPLYRSHTAT
>Locus_1548_Transcript_9/11_Confidence_0.543_Length_2298|.|"sp|P53624|MA121_DROME Mannosyl-oligosaccharide alpha-1,2-mannosidase isoform A OS=Drosophila melanogaster GN=alpha-Man-I PE=1 SV=2"|e=0|from=597|to=2036|frame=3|Fragment_1

PSAPTITNGEDSDPET
>Locus_1548_Transcript_9/11_Confidence_0.543_Length_2298|.|"sp|
P53624|MA121_DROME Mannosyl-oligosaccharide alpha-1,2-mannosidase
isoform A OS=Drosophila melanogaster GN=alpha-Man-I PE=1 SV=2"|e=0|
from=597|to=2036|frame=3|Fragment_2
RARRDKVKEMMRHAWAGYERHAWGANEVRPVSGAAHTSSIFGSASLGATIIDSMDTLLVM
GMTEEYERARKWVEEEFTVDNLNIEVSVFEMNIRFIGGFLACYALTGDELFKEKAKEVAD
KLLPAFQTPTGIPHALVNLKTGESKKYPWTGGFSSILAEFGTLHLEFTYLSDITGDEIYR
DKVQHIRSIIKQIDKVNGLYPNYLNPKTGKWGQHHMSLGALGDSFFEYLLKAWLQSGRQD
AEARVMYDEAMAAITEKMIKRSHQSGLTYVAELKYEKVEHKMDHLACFAGGLYGLGAQTL
QNDVSDSYLLIGEQLAATCHESYDRTPTKLGPESFRFTEAVEARAIKTTEKYYILRPETI
ESYFVLWRLTKNQKYRDWGWEAVQAIEKNCRASGGYTGIKNVYLEDSPKDDVQQSFFLAE
TLKYLYLLFSDDSLIPLDKWVFNTEAHPLPIKGANPLYRSHTAT
>Locus_1548_Transcript_10/11_Confidence_0.571_Length_2298|.|"sp|
P53624|MA121_DROME Mannosyl-oligosaccharide alpha-1,2-mannosidase
isoform A OS=Drosophila melanogaster GN=alpha-Man-I PE=1 SV=2"|e=0|
from=597|to=2036|frame=3|Fragment_1
PSAPTITNGEDSDPET
>Locus_1548_Transcript_10/11_Confidence_0.571_Length_2298|.|"sp|
P53624|MA121_DROME Mannosyl-oligosaccharide alpha-1,2-mannosidase
isoform A OS=Drosophila melanogaster GN=alpha-Man-I PE=1 SV=2"|e=0|
from=597|to=2036|frame=3|Fragment_2
RARRDKVKEMMRLAWAGYERHAWGANEVRPVSGAAHTSSIFGSASLGATIIDSMDTLLVM
GMTEEYERARKWVEEEFTVDNLNIEVSVFEMNIRFIGGFLACYALTGDELFKEKAKEVAD
KLLPAFQTPTGIPHALVNLKTGESKKYPWTGGFSSILAEFGTLHLEFTYLSDITGDEIYR
DKVQHIRSIIKQIDKVNGLYPNYLNPKTGKWGQHHMSLGALGDSFFEYLLKAWLQSGRQD
AEARVMYDEAMAAITEKMIKRSHQSGLTYVAELKYEKVEHKMDHLACFAGGLYGLGAQTL
QNDVSDSYLLIGEQLAATCHESYDRTPTKLGPESFRFTEAVEARAIKTTEKYYILRPETI
ESYFVLWRLTKNQKYRDWGWEAVQAIEKNCRASGGYTGIKNVYLEDSPKDDVQQSFFLAE
TLKYLYLLFSDDSLIPLDKWVFNTEAHPLPIKGANPLYRSHTAT
>Locus_1548_Transcript_11/11_Confidence_0.600_Length_2298|.|"sp|
P53624|MA121_DROME Mannosyl-oligosaccharide alpha-1,2-mannosidase
isoform A OS=Drosophila melanogaster GN=alpha-Man-I PE=1 SV=2"|e=0|
from=597|to=2036|frame=3|Fragment_1
PSAPTITNGEDSDPET
>Locus_1548_Transcript_11/11_Confidence_0.600_Length_2298|.|"sp|
P53624|MA121_DROME Mannosyl-oligosaccharide alpha-1,2-mannosidase
isoform A OS=Drosophila melanogaster GN=alpha-Man-I PE=1 SV=2"|e=0|
from=597|to=2036|frame=3|Fragment_2
RARRDKVKEMMRLAWAGYERHAWGANEVRPVSGAAHTSSIFGSASLGATIIDSMDTLLVM
GMTEEYERARKWVEEEFTVDNLNIEVSVFEMNIRFIGGFLACYALTGDELFKEKAKEVAD
KLLPAFQTPTGIPHALVNLKTGESKKYPWTGGFSSILAEFGTLHLEFTYLSDITGDEIYR
DKVQHIRSIIKQIDKVNGLYPNYLNPKTGKWGQHHMSLGALGDSFFEYLLKAWLQSGRQD
AEARVMYDEAMAAITEKMIKRSHQSGLTYVAELKYEKVEHKMDHLACFAGGLYGLGAQTL
QNDVSDSYLLIGEQLAATCHESYDRTPTKLGPESFRFTEAVEARAIKTTEKYYILRPETI
ESYFVLWRLTKNQKYRDWGWEAVQAIEKNCRASGGYTGIKNVYLEDSPKDDVQQSFFLAE
TLKYLYLLFSDDSLIPLDKWVFNTEAHPLPIKGANPLYRSHTAT
>Locus_23182_Transcript_1/2_Confidence_0.750_Length_317|.|"sp|
P53624|MA121_DROME Mannosyl-oligosaccharide alpha-1,2-mannosidase
isoform A OS=Drosophila melanogaster GN=alpha-Man-I PE=1 SV=2"|
e=1.66626E-27|from=19|to=312|frame=1|Fragment_1
GEDSDPET
>Locus_23182_Transcript_1/2_Confidence_0.750_Length_317|.|"sp|
P53624|MA121_DROME Mannosyl-oligosaccharide alpha-1,2-mannosidase

isoform A OS=Drosophila melanogaster GN=alpha-Man-I PE=1 SV=2"|
e=1.66626E-27|from=19|to=312|frame=1|Fragment_2
RARRDKVKEMMRHAWAGYERHAWGANEVRPVSGAAHTSSIFGSASLGATIIDSMDTLLVM
GMTEEYERARKWVEEEFTVDNLNIEVSVFE
>Locus_23182_Transcript_2/2_Confidence_0.750_Length_317|.|"sp|
P53624|MA121_DROME Mannosyl-oligosaccharide alpha-1,2-mannosidase
isoform A OS=Drosophila melanogaster GN=alpha-Man-I PE=1 SV=2"|
e=1.66626E-27|from=19|to=312|frame=1|Fragment_1
GEDSDPET
>Locus_23182_Transcript_2/2_Confidence_0.750_Length_317|.|"sp|
P53624|MA121_DROME Mannosyl-oligosaccharide alpha-1,2-mannosidase
isoform A OS=Drosophila melanogaster GN=alpha-Man-I PE=1 SV=2"|
e=1.66626E-27|from=19|to=312|frame=1|Fragment_2
RARRDKVKEMMRHAWAGYERHAWGANEVRPVSGAAHTSSIFGSASLGATIIDSMDTLLVM
GMTEEYERARKWVEEEFTVDNLNIEVSVFE
>Locus_1218_Transcript_1/3_Confidence_0.667_Length_878|UNIGENE
REPRESENTATIVE TRANSCRIPT|sp|P81928|140U_DROME RPII140-upstream gene
protein OS=Drosophila melanogaster GN=140up PE=2 SV=2|e=9.93193E-50|
from=22|to=699|frame=1|Fragment_1
LIAGVMPI
>Locus_1218_Transcript_1/3_Confidence_0.667_Length_878|UNIGENE
REPRESENTATIVE TRANSCRIPT|sp|P81928|140U_DROME RPII140-upstream gene
protein OS=Drosophila melanogaster GN=140up PE=2 SV=2|e=9.93193E-50|
from=22|to=699|frame=1|Fragment_2
TDHDILPDTKTTAEMIKKKLESETPKDRLYKMFSMDEFGIISPELTSIVQATSFGALTGA
VYGGFIKSKMAYEDFMSNNQATSFTSMQEAKQKLQNQVTIGFGKGAFMWGWRLALFTCSY
ITITTVISVYRDRSSIWEYLAAGSLTGALYKFRMGPKGMI
>Locus_1218_Transcript_1/3_Confidence_0.667_Length_878|UNIGENE
REPRESENTATIVE TRANSCRIPT|sp|P81928|140U_DROME RPII140-upstream gene
protein OS=Drosophila melanogaster GN=140up PE=2 SV=2|e=9.93193E-50|
from=22|to=699|frame=1|Fragment_3
IAGTISLTVLYLTGTTVQELRHWSYQLEETRN
>Locus_1218_Transcript_1/3_Confidence_0.667_Length_878|UNIGENE
REPRESENTATIVE TRANSCRIPT|sp|P81928|140U_DROME RPII140-upstream gene
protein OS=Drosophila melanogaster GN=140up PE=2 SV=2|e=9.93193E-50|
from=22|to=699|frame=1|Fragment_4
NAKRQMQSGPRTEDP
>Locus_1218_Transcript_2/3_Confidence_0.667_Length_710|.|sp|P81928|
140U_DROME RPII140-upstream gene protein OS=Drosophila melanogaster
GN=140up PE=2 SV=2|e=1.01778E-40|from=1|to=531|frame=1|Fragment_1
SPELTSIVQATSFGALTGAVYGGFIKSKMAYEDFMSNNQATSFTSMQEAKQKLQNQVTIG
FGKGAFMWGWRLALFTCSYITITTVISVYRDRSSIWEYLAAGSLTGALYKFRMGPKGMI
>Locus_1218_Transcript_2/3_Confidence_0.667_Length_710|.|sp|P81928|
140U_DROME RPII140-upstream gene protein OS=Drosophila melanogaster
GN=140up PE=2 SV=2|e=1.01778E-40|from=1|to=531|frame=1|Fragment_2
IAGTISLTVLYLTGTTVQELRHWSYQLEETRN
>Locus_1218_Transcript_2/3_Confidence_0.667_Length_710|.|sp|P81928|
140U_DROME RPII140-upstream gene protein OS=Drosophila melanogaster
GN=140up PE=2 SV=2|e=1.01778E-40|from=1|to=531|frame=1|Fragment_3
NAKRQMQSGPRTEDP
>Locus_1218_Transcript_3/3_Confidence_0.667_Length_710|.|sp|P81928|
140U_DROME RPII140-upstream gene protein OS=Drosophila melanogaster
GN=140up PE=2 SV=2|e=1.01778E-40|from=1|to=531|frame=1|Fragment_1
SPELTSIVQATSFGALTGAVYGGFIKSKMAYEDFMSNNQATSFTSMQEAKQKLQNQVTIG

FGKGAFMWGWRLALFTCSYITITTVISVYRDRSSIWEYLAAGSLTGALYKFRMGPKGMI
>Locus_1218_Transcript_3/3_Confidence_0.667_Length_710|.|sp|P81928|140U_DROME RPII140-upstream gene protein OS=Drosophila melanogaster GN=140up PE=2 SV=2|e=1.01778E-40|from=1|to=531|frame=1|Fragment_2
IAGTISLTVLYLTGTTVQELRHWSYQLEETRN
>Locus_1218_Transcript_3/3_Confidence_0.667_Length_710|.|sp|P81928|140U_DROME RPII140-upstream gene protein OS=Drosophila melanogaster GN=140up PE=2 SV=2|e=1.01778E-40|from=1|to=531|frame=1|Fragment_3
NAKRQMQSGPRTEDP
>Locus_10462_Transcript_1/1_Confidence_1.000_Length_302|.|sp|P81928|140U_DROME RPII140-upstream gene protein OS=Drosophila melanogaster GN=140up PE=2 SV=2|e=5.28198E-13|from=43|to=300|frame=1|Fragment_1
LIAGVMPI
>Locus_10462_Transcript_1/1_Confidence_1.000_Length_302|.|sp|P81928|140U_DROME RPII140-upstream gene protein OS=Drosophila melanogaster GN=140up PE=2 SV=2|e=5.28198E-13|from=43|to=300|frame=1|Fragment_2
TDHDILPDTKTTAEMIKKKLESETPRDRLYKMFSMDEFGIISPELTSIVQATSFGALTGA
VYGGFIKSKMAYEDFMSN
>Locus_29945_Transcript_1/1_Confidence_1.000_Length_281|.|sp|P81928|140U_DROME RPII140-upstream gene protein OS=Drosophila melanogaster GN=140up PE=2 SV=2|e=5.19659E-13|from=22|to=279|frame=1|Fragment_1
LIAGVMPI
>Locus_29945_Transcript_1/1_Confidence_1.000_Length_281|.|sp|P81928|140U_DROME RPII140-upstream gene protein OS=Drosophila melanogaster GN=140up PE=2 SV=2|e=5.19659E-13|from=22|to=279|frame=1|Fragment_2
TDHDILPDTKTTAEMIKKKLESETPKDRLYKMFSMDEFGIISPELTSIVQATSFGALTGA
VYGGFIKSKMAYEDFMSN
>Locus_21596_Transcript_1/1_Confidence_1.000_Length_243|UNIGENE REPRESENTATIVE TRANSCRIPT|sp|P91645|CAC1A_DROME Voltage-dependent calcium channel type A subunit alpha-1 OS=Drosophila melanogaster GN=cac PE=2 SV=3|e=1.23408E-25|from=1|to=243|frame=1|Fragment_1
LVISLLNSMR
>Locus_21596_Transcript_1/1_Confidence_1.000_Length_243|UNIGENE REPRESENTATIVE TRANSCRIPT|sp|P91645|CAC1A_DROME Voltage-dependent calcium channel type A subunit alpha-1 OS=Drosophila melanogaster GN=cac PE=2 SV=3|e=1.23408E-25|from=1|to=243|frame=1|Fragment_2
GMQLFGGQFNFPDGTPATNFNTFPIALLTVFQILTGEDWNEVMYQGIASQGG
>Locus_4079_Transcript_1/2_Confidence_0.750_Length_3495|UNIGENE REPRESENTATIVE TRANSCRIPT|sp|P91682|SMO_DROME Protein smoothened OS=Drosophila melanogaster GN=smo PE=1 SV=1|e=0|from=385|to=3219|frame=-1|Fragment_1
ENSEKILEAINDSINYRIQNLKGKDEKPWFSSRDTALSYCVRPGKCEKLEY
>Locus_4079_Transcript_1/2_Confidence_0.750_Length_3495|UNIGENE REPRESENTATIVE TRANSCRIPT|sp|P91682|SMO_DROME Protein smoothened OS=Drosophila melanogaster GN=smo PE=1 SV=1|e=0|from=385|to=3219|frame=-1|Fragment_2
NMCFNAKIPYSYTSLDLTY
>Locus_4079_Transcript_1/2_Confidence_0.750_Length_3495|UNIGENE REPRESENTATIVE TRANSCRIPT|sp|P91682|SMO_DROME Protein smoothened OS=Drosophila melanogaster GN=smo PE=1 SV=1|e=0|from=385|to=3219|frame=-1|Fragment_3
QTLIHIPKCWAVIQPFLCATYFPKCENISNTEIVYIPSLEMCKIIMEPCRLVYNTTFFPD
FLKCNPSLYSSSCKNDVRDMKFNITGQCLPPLVKTDSIKHFYDGIDGCGLQCNDALYTVN
EHQQIHKLIAWGATICLLCNMFTILTFLIDWQNANKYPALVIFYINCWFLISCLGFIAQF

IPGAREDIVCRKDGTLRQSEPSAGENLSC
>Locus_4079_Transcript_1/2_Confidence_0.750_Length_3495|UNIGENE REPRESENTATIVE TRANSCRIPT|sp|P91682|SMO_DROME Protein smoothened OS=Drosophila melanogaster GN=smo PE=1 SV=1|e=0|from=385|to=3219|frame=-1|Fragment_4
VYYSLIAAMVWFVIFTYVWHISFQAIGKIQERIDKKGAYFHLIACSLPLVLTITTMALSE
IDGNSIVGICFLGYKNHSIRAGLLLGPICGVIIVGGYFLSRGFLTLITLKITSKDIISAR
ASKKIRETIARMGLCCLLMVVLIIGTFSCHINEFQSTQAWASDLKNYIICLIMYKYDTSN
SAESCRLTSRPSVALLQLHLLCWFATGIIMSSWVWTKSSAEAWKRYIRKKFDCEIEEPLK
LQKHKVIAQAFAKRKEFHDAGRLSITFHNTHTDPLGLNF
>Locus_4079_Transcript_1/2_Confidence_0.750_Length_3495|UNIGENE REPRESENTATIVE TRANSCRIPT|sp|P91682|SMO_DROME Protein smoothened OS=Drosophila melanogaster GN=smo PE=1 SV=1|e=0|from=385|to=3219|frame=-1|Fragment_5
ELNSVASHDFSSTWANNLPRFVNRRCALTCGPNTGSASSSHCGNHRRNSLDSEISFSVRH
VSVESRRNSLDSQISFKIAEMKTKVASRRCSKGKR
>Locus_4079_Transcript_1/2_Confidence_0.750_Length_3495|UNIGENE REPRESENTATIVE TRANSCRIPT|sp|P91682|SMO_DROME Protein smoothened OS=Drosophila melanogaster GN=smo PE=1 SV=1|e=0|from=385|to=3219|frame=-1|Fragment_6
IRKRDFF
>Locus_4079_Transcript_1/2_Confidence_0.750_Length_3495|UNIGENE REPRESENTATIVE TRANSCRIPT|sp|P91682|SMO_DROME Protein smoothened OS=Drosophila melanogaster GN=smo PE=1 SV=1|e=0|from=385|to=3219|frame=-1|Fragment_7
STSLETQ
>Locus_4079_Transcript_1/2_Confidence_0.750_Length_3495|UNIGENE REPRESENTATIVE TRANSCRIPT|sp|P91682|SMO_DROME Protein smoothened OS=Drosophila melanogaster GN=smo PE=1 SV=1|e=0|from=385|to=3219|frame=-1|Fragment_8
IGAQIIKVLNADHNSRPSLKRRTGNAGLDSDNINSLL
>Locus_4079_Transcript_1/2_Confidence_0.750_Length_3495|UNIGENE REPRESENTATIVE TRANSCRIPT|sp|P91682|SMO_DROME Protein smoothened OS=Drosophila melanogaster GN=smo PE=1 SV=1|e=0|from=385|to=3219|frame=-1|Fragment_9
CDGKLVLPFLHNQGLTTSDDECHSVTSLTIKDSRLDVVLRRKSHISSDDNLNQVSINIEE
YHSSDDKCSPNNYEKSDPANEKCIMSSSHKDRKEKEGKITKSTSKY
>Locus_4079_Transcript_1/2_Confidence_0.750_Length_3495|UNIGENE REPRESENTATIVE TRANSCRIPT|sp|P91682|SMO_DROME Protein smoothened OS=Drosophila melanogaster GN=smo PE=1 SV=1|e=0|from=385|to=3219|frame=-1|Fragment_10
NDKVSRSNKPIESESGADHDLCESRAP
>Locus_4079_Transcript_1/2_Confidence_0.750_Length_3495|UNIGENE REPRESENTATIVE TRANSCRIPT|sp|P91682|SMO_DROME Protein smoothened OS=Drosophila melanogaster GN=smo PE=1 SV=1|e=0|from=385|to=3219|frame=-1|Fragment_11
SYCSELSPLAVQSSFSGISVGKTNSRNSKLSCDVGIQANAHEI
>Locus_4079_Transcript_2/2_Confidence_0.750_Length_3495|.|sp|P91682|SMO_DROME Protein smoothened OS=Drosophila melanogaster GN=smo PE=1 SV=1|e=0|from=385|to=3219|frame=-1|Fragment_1
ENSEKILEAINDSINYRIQNLKGKDEKPWFSSRDTALSYCVRPGKCEKLEY
>Locus_4079_Transcript_2/2_Confidence_0.750_Length_3495|.|sp|P91682|SMO_DROME Protein smoothened OS=Drosophila melanogaster GN=smo PE=1 SV=1|e=0|from=385|to=3219|frame=-1|Fragment_2

NMCFNAKIPYSYTSLDLTY
>Locus_4079_Transcript_2/2_Confidence_0.750_Length_3495|.|sp|P91682|SMO_DROME Protein smoothened OS=Drosophila melanogaster GN=smo PE=1 SV=1|e=0|from=385|to=3219|frame=-1|Fragment_3
QTLIHIPKCWAVIQPFLCATYFPKCENISNTEIVYIPSLEMCKIIMEPCRLVYNTTFFPD
FLKCNPSLYSSSCKNDVRDMKFNITGQCLPPLVKTDSIKHFYDGIDGCGLQCNDALYTVN
EHQQIHKLIAWGATICLLCNMFTILTFLIDWQNANKYPALVIFYINCWFLISCLGFIAQF
IPGAREDIVCRKDGTLRQSEPSAGENLSC
>Locus_4079_Transcript_2/2_Confidence_0.750_Length_3495|.|sp|P91682|SMO_DROME Protein smoothened OS=Drosophila melanogaster GN=smo PE=1 SV=1|e=0|from=385|to=3219|frame=-1|Fragment_4
VYYSLIAAMVWFVIFTYVWHISFQAIGKIQERIDKKGAYFHLIACSLPLVLTITTMALSE
IDGNSIVGICFLGYKNHSIRAGLLLGPICGVIIVGGYFLSRGFLTLITLKITSKDIISAR
ASKKIRETIARMGLCCLLMVVLIIGTFSCHINEFQSTQAWASDLKNYIICLIMYKYDTSN
SAESCRLTSRPSVALLQLHLLCWFATGIIMSSWVWTKSSAEAWKRYIRKKFDCEIEEPLK
LQKHKVIAQAFAKRKEFHDAGRLSITFHNTHTDPLGLNF
>Locus_4079_Transcript_2/2_Confidence_0.750_Length_3495|.|sp|P91682|SMO_DROME Protein smoothened OS=Drosophila melanogaster GN=smo PE=1 SV=1|e=0|from=385|to=3219|frame=-1|Fragment_5
ELNSVASHDFSSTWANNLPRFVNRRCALTCGPNTGSASSSHCGNHRRNSLDSEISFSVRH
VSVESRRNSLDSQISFKIAEMKTKVASRRCSKGKR
>Locus_4079_Transcript_2/2_Confidence_0.750_Length_3495|.|sp|P91682|SMO_DROME Protein smoothened OS=Drosophila melanogaster GN=smo PE=1 SV=1|e=0|from=385|to=3219|frame=-1|Fragment_6
IRKRDFF
>Locus_4079_Transcript_2/2_Confidence_0.750_Length_3495|.|sp|P91682|SMO_DROME Protein smoothened OS=Drosophila melanogaster GN=smo PE=1 SV=1|e=0|from=385|to=3219|frame=-1|Fragment_7
STSLETQ
>Locus_4079_Transcript_2/2_Confidence_0.750_Length_3495|.|sp|P91682|SMO_DROME Protein smoothened OS=Drosophila melanogaster GN=smo PE=1 SV=1|e=0|from=385|to=3219|frame=-1|Fragment_8
IGAQIIKVLNADHNSRPSLKRRTGNAGLDSDNINSLL
>Locus_4079_Transcript_2/2_Confidence_0.750_Length_3495|.|sp|P91682|SMO_DROME Protein smoothened OS=Drosophila melanogaster GN=smo PE=1 SV=1|e=0|from=385|to=3219|frame=-1|Fragment_9
CDGKLVLPFLHNQGLTTSDDECHSVTSLTIKDSRLDVVLRRKSHISSDDNLNQVSINIEE
YHSSDDKCSPNNYEKSDPANEKCIMSSSHKDRKEKEGKITKSTSKY
>Locus_4079_Transcript_2/2_Confidence_0.750_Length_3495|.|sp|P91682|SMO_DROME Protein smoothened OS=Drosophila melanogaster GN=smo PE=1 SV=1|e=0|from=385|to=3219|frame=-1|Fragment_10
NDKVSRSNKPIESESGADHDLCESRAP
>Locus_4079_Transcript_2/2_Confidence_0.750_Length_3495|.|sp|P91682|SMO_DROME Protein smoothened OS=Drosophila melanogaster GN=smo PE=1 SV=1|e=0|from=385|to=3219|frame=-1|Fragment_11
SYCSELSPLAVQSSFSGISVGKTNSRNSKLSCDVGIQANAHEI
>Locus_1925_Transcript_1/5_Confidence_0.600_Length_895|UNIGENE REPRESENTATIVE TRANSCRIPT|sp|P92029|DNJ60_DROME DnaJ-like protein 60 OS=Drosophila melanogaster GN=DnaJ-60 PE=2 SV=1|e=1.64937E-23|from=4|to=555|frame=1|Fragment_1
SHYDVLKLRKNCSTREIKASFIELSKKFHPDVNSG
>Locus_1925_Transcript_1/5_Confidence_0.600_Length_895|UNIGENE REPRESENTATIVE TRANSCRIPT|sp|P92029|DNJ60_DROME DnaJ-like protein 60 OS=Drosophila melanogaster GN=DnaJ-60 PE=2 SV=1|e=1.64937E-23|

from=4|to=555|frame=1|Fragment_2
KRSHIDFLQISEAYKILSRPVTRRKYDAELAIKLRETTWPRRDMDPETWSDSTIWNSKNK
TSQKPKPYYGIPGVKRLPNAWIVAMCLTTAGIALAAEVFLISTASRHTRQKITMQSMANE
ET
>Locus_1925_Transcript_1/5_Confidence_0.600_Length_895|UNIGENE
REPRESENTATIVE TRANSCRIPT|sp|P92029|DNJ60_DROME DnaJ-like protein 60
OS=Drosophila melanogaster GN=DnaJ-60 PE=2 SV=1|e=1.64937E-23|
from=4|to=555|frame=1|Fragment_3
LATVQAEARRFGNEIQLERLIARAKLS
>Locus_1925_Transcript_5/5_Confidence_0.500_Length_895|.|sp|P92029|
DNJ60_DROME DnaJ-like protein 60 OS=Drosophila melanogaster
GN=DnaJ-60 PE=2 SV=1|e=1.64937E-23|from=4|to=555|frame=1|Fragment_1
SHYDVLKLRKNCSTREIKASFIELSKKFHPDVNSG
>Locus_1925_Transcript_5/5_Confidence_0.500_Length_895|.|sp|P92029|
DNJ60_DROME DnaJ-like protein 60 OS=Drosophila melanogaster
GN=DnaJ-60 PE=2 SV=1|e=1.64937E-23|from=4|to=555|frame=1|Fragment_2
KRSHIDFLQISEAYKILSRPVTRRKYDAELAIKLRETTWPRRDMDPETWSDSTIWNSKNK
TSQKPKPYYGIPGVKRLPNAWIVAMCLTTAGIALAAEVFLISTASRHTRQKITMQSMANE
ET
>Locus_1925_Transcript_5/5_Confidence_0.500_Length_895|.|sp|P92029|
DNJ60_DROME DnaJ-like protein 60 OS=Drosophila melanogaster
GN=DnaJ-60 PE=2 SV=1|e=1.64937E-23|from=4|to=555|frame=1|Fragment_3
LATVQAEARRFGNEIQLERLIARAKLS
>Locus_1925_Transcript_4/5_Confidence_0.500_Length_796|.|sp|P92029|
DNJ60_DROME DnaJ-like protein 60 OS=Drosophila melanogaster
GN=DnaJ-60 PE=2 SV=1|e=1.37322E-23|from=4|to=555|frame=1|Fragment_1
SHYDVLKLRKNCSTREIKASFIELSKKFHPDVNSG
>Locus_1925_Transcript_4/5_Confidence_0.500_Length_796|.|sp|P92029|
DNJ60_DROME DnaJ-like protein 60 OS=Drosophila melanogaster
GN=DnaJ-60 PE=2 SV=1|e=1.37322E-23|from=4|to=555|frame=1|Fragment_2
KRSHIDFLQISEAYKILSRPVTRRKYDAELAIKLRETTWPRRDMDPETWSDSTIWNSKNK
TSQKPKPYYGIPGVKRLPNAWIVAMCLTTAGIALAAEVFLISTASRHTRQKITMQSMANE
ET
>Locus_539_Transcript_2/3_Confidence_0.714_Length_5667|UNIGENE
REPRESENTATIVE TRANSCRIPT|sp|P98163|YL_DROME Putative vitellogenin
receptor OS=Drosophila melanogaster GN=yl PE=1 SV=2|e=0|from=436|
to=5412|frame=-1|Fragment_1
CLTSEYTCGDGECISKTRICDGIKDCKDDSDEHHCEMYRCEEPDWYRCAD
>Locus_539_Transcript_2/3_Confidence_0.714_Length_5667|UNIGENE
REPRESENTATIVE TRANSCRIPT|sp|P98163|YL_DROME Putative vitellogenin
receptor OS=Drosophila melanogaster GN=yl PE=1 SV=2|e=0|from=436|
to=5412|frame=-1|Fragment_2
GECISSSFRCDKSMDCPSGSDEQNCE
>Locus_539_Transcript_2/3_Confidence_0.714_Length_5667|UNIGENE
REPRESENTATIVE TRANSCRIPT|sp|P98163|YL_DROME Putative vitellogenin
receptor OS=Drosophila melanogaster GN=yl PE=1 SV=2|e=0|from=436|
to=5412|frame=-1|Fragment_3
DFVYTPPSTN
>Locus_539_Transcript_2/3_Confidence_0.714_Length_5667|UNIGENE
REPRESENTATIVE TRANSCRIPT|sp|P98163|YL_DROME Putative vitellogenin
receptor OS=Drosophila melanogaster GN=yl PE=1 SV=2|e=0|from=436|
to=5412|frame=-1|Fragment_4
HSCEHNEWMCA
>Locus_539_Transcript_2/3_Confidence_0.714_Length_5667|UNIGENE

REPRESENTATIVE TRANSCRIPT|sp|P98163|YL_DROME Putative vitellogenin receptor OS=Drosophila melanogaster GN=yl PE=1 SV=2|e=0|from=436|to=5412|frame=-1|Fragment_5
DRMCIPLEFVCNGDFDCMDKSDEDMGCKDQHAC
>Locus_539_Transcript_2/3_Confidence_0.714_Length_5667|UNIGENE REPRESENTATIVE TRANSCRIPT|sp|P98163|YL_DROME Putative vitellogenin receptor OS=Drosophila melanogaster GN=yl PE=1 SV=2|e=0|from=436|to=5412|frame=-1|Fragment_6
DGFRCRNGHCIS
>Locus_539_Transcript_2/3_Confidence_0.714_Length_5667|UNIGENE REPRESENTATIVE TRANSCRIPT|sp|P98163|YL_DROME Putative vitellogenin receptor OS=Drosophila melanogaster GN=yl PE=1 SV=2|e=0|from=436|to=5412|frame=-1|Fragment_7
EDWVCDGAIDCADGSDEIDCDSRPVTFMCILEKDHYPCRDNSSCIPINKVCDNIPHCKDK
SDEGVGCQQ
>Locus_539_Transcript_2/3_Confidence_0.714_Length_5667|UNIGENE REPRESENTATIVE TRANSCRIPT|sp|P98163|YL_DROME Putative vitellogenin receptor OS=Drosophila melanogaster GN=yl PE=1 SV=2|e=0|from=436|to=5412|frame=-1|Fragment_8
DCTSLSCA
>Locus_539_Transcript_2/3_Confidence_0.714_Length_5667|UNIGENE REPRESENTATIVE TRANSCRIPT|sp|P98163|YL_DROME Putative vitellogenin receptor OS=Drosophila melanogaster GN=yl PE=1 SV=2|e=0|from=436|to=5412|frame=-1|Fragment_9
KHCVKTP
>Locus_539_Transcript_2/3_Confidence_0.714_Length_5667|UNIGENE REPRESENTATIVE TRANSCRIPT|sp|P98163|YL_DROME Putative vitellogenin receptor OS=Drosophila melanogaster GN=yl PE=1 SV=2|e=0|from=436|to=5412|frame=-1|Fragment_10
EGPRCVCPSGFHNPLNSDFSHMHKNWQYNNNDNCTDINECAIHG
>Locus_539_Transcript_2/3_Confidence_0.714_Length_5667|UNIGENE REPRESENTATIVE TRANSCRIPT|sp|P98163|YL_DROME Putative vitellogenin receptor OS=Drosophila melanogaster GN=yl PE=1 SV=2|e=0|from=436|to=5412|frame=-1|Fragment_11
MCSQRCENTIGSYKCLCDMKYTLSSDKKSCK
>Locus_539_Transcript_2/3_Confidence_0.714_Length_5667|UNIGENE REPRESENTATIVE TRANSCRIPT|sp|P98163|YL_DROME Putative vitellogenin receptor OS=Drosophila melanogaster GN=yl PE=1 SV=2|e=0|from=436|to=5412|frame=-1|Fragment_12
VNGG
>Locus_539_Transcript_2/3_Confidence_0.714_Length_5667|UNIGENE REPRESENTATIVE TRANSCRIPT|sp|P98163|YL_DROME Putative vitellogenin receptor OS=Drosophila melanogaster GN=yl PE=1 SV=2|e=0|from=436|to=5412|frame=-1|Fragment_13
EALLLFSSRSNIRGLYLES
>Locus_539_Transcript_2/3_Confidence_0.714_Length_5667|UNIGENE REPRESENTATIVE TRANSCRIPT|sp|P98163|YL_DROME Putative vitellogenin receptor OS=Drosophila melanogaster GN=yl PE=1 SV=2|e=0|from=436|to=5412|frame=-1|Fragment_14
KILFQVIKNQRQIIGVEYDGNHVYWTDVHLGTESILRSLEDGSQMEVLLTSGLSAPEDLA
LDWLTGNIYFTDAELMHIGVCSNDGKHCTIIVNENIHKPRAIALYPGRGQMFWSDWGDNP
QIATSSMDGSSPKLFVSTNLHWPNGISIDIPNKRLYWVDAKLQTIESINLDGTDRRIVLK
GVTKHPYAIAIFEDQIYWSDWDSKSIQSCNKFTCKNRVTVAKESKEYIYGIHIYHSALQP
KHDHPCMKSSCSHLCLIKSAH

>Locus_539_Transcript_2/3_Confidence_0.714_Length_5667|UNIGENE REPRESENTATIVE TRANSCRIPT|sp|P98163|YL_DROME Putative vitellogenin receptor OS=Drosophila melanogaster GN=yl PE=1 SV=2|e=0|from=436|to=5412|frame=-1|Fragment_15
EYSCACPEGKELSADKTSCRETQRQSELIIGAGNLMVSLMHQKLGRHEL
>Locus_539_Transcript_2/3_Confidence_0.714_Length_5667|UNIGENE REPRESENTATIVE TRANSCRIPT|sp|P98163|YL_DROME Putative vitellogenin receptor OS=Drosophila melanogaster GN=yl PE=1 SV=2|e=0|from=436|to=5412|frame=-1|Fragment_16
HGVPMKINMDVLTYNSLNGEIIVADNNKKFIYTVDMYTTKSSRLTDIKSGEVEAMHFDNL
ANNLFWIDSVQQTIEVLHLDTGVKGFILHGLGDQVPVGFAIVPSHGIMFVAFRSHDHTHI
DRMLLDGSGIRTHVVEDGLGRWGKISMVYDHDLQRVFWAVEETGRIESSSVDGDDRHGFR
HTKDKPIAIAILGNDLFWTTAGNRRLYWADKHNLGNGFNKKLTIDIER
>Locus_539_Transcript_2/3_Confidence_0.714_Length_5667|UNIGENE REPRESENTATIVE TRANSCRIPT|sp|P98163|YL_DROME Putative vitellogenin receptor OS=Drosophila melanogaster GN=yl PE=1 SV=2|e=0|from=436|to=5412|frame=-1|Fragment_17
NLKSVPLTAITPRTISNHLCIHENGGCSHICIPMMQGKISCHCPVGLSLVPPYNMSCETI
IHCTGEQFRCSDTNKCIPMILRCNGNKDCDFGEDESDCE
>Locus_539_Transcript_2/3_Confidence_0.714_Length_5667|UNIGENE REPRESENTATIVE TRANSCRIPT|sp|P98163|YL_DROME Putative vitellogenin receptor OS=Drosophila melanogaster GN=yl PE=1 SV=2|e=0|from=436|to=5412|frame=-1|Fragment_18
RYKDRQSCAVNEFQCNDG
>Locus_539_Transcript_2/3_Confidence_0.714_Length_5667|UNIGENE REPRESENTATIVE TRANSCRIPT|sp|P98163|YL_DROME Putative vitellogenin receptor OS=Drosophila melanogaster GN=yl PE=1 SV=2|e=0|from=436|to=5412|frame=-1|Fragment_19
ICIEYHYKCDKHFDCPDKSDEENCENYVSSSKCTHAQFECANGQCIDNRLLCDVLPDCLD
GSDEKMCDHVT
>Locus_539_Transcript_2/3_Confidence_0.714_Length_5667|UNIGENE REPRESENTATIVE TRANSCRIPT|sp|P98163|YL_DROME Putative vitellogenin receptor OS=Drosophila melanogaster GN=yl PE=1 SV=2|e=0|from=436|to=5412|frame=-1|Fragment_20
CESNMFQCQSGSCIPSSWECDGEIDCTDASDEHTFCEPNTCKDNQFTCTNGKCIDMDLKC
DHEDDCGDSSDEIRCPSKEKESTTETDFIDLITCEPTQYTCEES
>Locus_539_Transcript_2/3_Confidence_0.714_Length_5667|UNIGENE REPRESENTATIVE TRANSCRIPT|sp|P98163|YL_DROME Putative vitellogenin receptor OS=Drosophila melanogaster GN=yl PE=1 SV=2|e=0|from=436|to=5412|frame=-1|Fragment_21
GACLPEIARCNGTAECKYGEDERECTN
>Locus_539_Transcript_2/3_Confidence_0.714_Length_5667|UNIGENE REPRESENTATIVE TRANSCRIPT|sp|P98163|YL_DROME Putative vitellogenin receptor OS=Drosophila melanogaster GN=yl PE=1 SV=2|e=0|from=436|to=5412|frame=-1|Fragment_22
CQLDEFTCNSG
>Locus_539_Transcript_2/3_Confidence_0.714_Length_5667|UNIGENE REPRESENTATIVE TRANSCRIPT|sp|P98163|YL_DROME Putative vitellogenin receptor OS=Drosophila melanogaster GN=yl PE=1 SV=2|e=0|from=436|to=5412|frame=-1|Fragment_23
QCIQRKWVCDKTRDCNDGSDETS
>Locus_539_Transcript_2/3_Confidence_0.714_Length_5667|UNIGENE REPRESENTATIVE TRANSCRIPT|sp|P98163|YL_DROME Putative vitellogenin receptor OS=Drosophila melanogaster GN=yl PE=1 SV=2|e=0|from=436|

to=5412|frame=-1|Fragment_24
ESCSAVTTVQPVHSQFLPVCDEDSFRCKSGGCVDFLFVCNGIPDCFDGSDENGACNTSCD
AIK
>Locus_539_Transcript_2/3_Confidence_0.714_Length_5667|UNIGENE
REPRESENTATIVE TRANSCRIPT|sp|P98163|YL_DROME Putative vitellogenin
receptor OS=Drosophila melanogaster GN=yl PE=1 SV=2|e=0|from=436|
to=5412|frame=-1|Fragment_25
CKHKCSPTPNGPQCSCHEGYHLAGDHLSCTDINECKAELQQCGQICTNSAGSFHCSCYPE
FMLRSDKRSCKATGDQMSILYSTSNQIRELTLSPPSVKILFNRNIPKILSLDVSKHTSTL
YFSLHLISQIYRLDIKSGDVQYIMNIGKPMKLAVDWITQNIYFISEDDLHKTLRVCHIEN
AACAILMKFDKNIAVPELVVDPISKYIFYTVTHWWVYDNPITILYRANLDGSHVIELRNE
KLNLISGIGIDHHQKTVYYCDKHKNTIESVSY
>Locus_539_Transcript_2/3_Confidence_0.714_Length_5667|UNIGENE
REPRESENTATIVE TRANSCRIPT|sp|P98163|YL_DROME Putative vitellogenin
receptor OS=Drosophila melanogaster GN=yl PE=1 SV=2|e=0|from=436|
to=5412|frame=-1|Fragment_26
NGSDHR
>Locus_539_Transcript_2/3_Confidence_0.714_Length_5667|UNIGENE
REPRESENTATIVE TRANSCRIPT|sp|P98163|YL_DROME Putative vitellogenin
receptor OS=Drosophila melanogaster GN=yl PE=1 SV=2|e=0|from=436|
to=5412|frame=-1|Fragment_27
ILVKEQKLLTAPNGLDVFENYAYTLVYGSTRMIKCRLFGNYACTVIDAYISNADSFKISQ
YSKQ
>Locus_539_Transcript_2/3_Confidence_0.714_Length_5667|UNIGENE
REPRESENTATIVE TRANSCRIPT|sp|P98163|YL_DROME Putative vitellogenin
receptor OS=Drosophila melanogaster GN=yl PE=1 SV=2|e=0|from=436|
to=5412|frame=-1|Fragment_28
LLHENACENSNCMYLCVPADSGPKCICKNGLMIAPGSSC
>Locus_539_Transcript_3/3_Confidence_0.714_Length_5667|.|sp|P98163|
YL_DROME Putative vitellogenin receptor OS=Drosophila melanogaster
GN=yl PE=1 SV=2|e=0|from=436|to=5412|frame=-1|Fragment_1
CLTSEYTCGDGECISKTRICDGIKDCKDDSDEHHCEMYRCEEPDWYRCAD
>Locus_539_Transcript_3/3_Confidence_0.714_Length_5667|.|sp|P98163|
YL_DROME Putative vitellogenin receptor OS=Drosophila melanogaster
GN=yl PE=1 SV=2|e=0|from=436|to=5412|frame=-1|Fragment_2
GECISSSFRCDKSMDCPSGSDEQNCE
>Locus_539_Transcript_3/3_Confidence_0.714_Length_5667|.|sp|P98163|
YL_DROME Putative vitellogenin receptor OS=Drosophila melanogaster
GN=yl PE=1 SV=2|e=0|from=436|to=5412|frame=-1|Fragment_3
DFVYTPPSTN
>Locus_539_Transcript_3/3_Confidence_0.714_Length_5667|.|sp|P98163|
YL_DROME Putative vitellogenin receptor OS=Drosophila melanogaster
GN=yl PE=1 SV=2|e=0|from=436|to=5412|frame=-1|Fragment_4
HSCEHNEWMCA
>Locus_539_Transcript_3/3_Confidence_0.714_Length_5667|.|sp|P98163|
YL_DROME Putative vitellogenin receptor OS=Drosophila melanogaster
GN=yl PE=1 SV=2|e=0|from=436|to=5412|frame=-1|Fragment_5
DRMCIPLEFVCNGDFDCMDKSDEDMGCKDQHAC
>Locus_539_Transcript_3/3_Confidence_0.714_Length_5667|.|sp|P98163|
YL_DROME Putative vitellogenin receptor OS=Drosophila melanogaster
GN=yl PE=1 SV=2|e=0|from=436|to=5412|frame=-1|Fragment_6
DGFRCRNGHCIS
>Locus_539_Transcript_3/3_Confidence_0.714_Length_5667|.|sp|P98163|
YL_DROME Putative vitellogenin receptor OS=Drosophila melanogaster

GN=yl PE=1 SV=2|e=0|from=436|to=5412|frame=-1|Fragment_7
EDWVCDGAIDCADGSDEIDCDSRPVTFMCILEKDHYPCRDNSSCIPINKVCDNIPHCKDK
SDEGVGCQQ
>Locus_539_Transcript_3/3_Confidence_0.714_Length_5667|.|sp|P98163|
YL_DROME Putative vitellogenin receptor OS=Drosophila melanogaster
GN=yl PE=1 SV=2|e=0|from=436|to=5412|frame=-1|Fragment_8
DCTSLSCA
>Locus_539_Transcript_3/3_Confidence_0.714_Length_5667|.|sp|P98163|
YL_DROME Putative vitellogenin receptor OS=Drosophila melanogaster
GN=yl PE=1 SV=2|e=0|from=436|to=5412|frame=-1|Fragment_9
KHCVKTP
>Locus_539_Transcript_3/3_Confidence_0.714_Length_5667|.|sp|P98163|
YL_DROME Putative vitellogenin receptor OS=Drosophila melanogaster
GN=yl PE=1 SV=2|e=0|from=436|to=5412|frame=-1|Fragment_10
EGPRCVCPSGFHNPLNSDFSHMHKNWQYNNNDNCTDINECAIHG
>Locus_539_Transcript_3/3_Confidence_0.714_Length_5667|.|sp|P98163|
YL_DROME Putative vitellogenin receptor OS=Drosophila melanogaster
GN=yl PE=1 SV=2|e=0|from=436|to=5412|frame=-1|Fragment_11
MCSQRCENTIGSYKCLCDMKYTLSSDKKSCK
>Locus_539_Transcript_3/3_Confidence_0.714_Length_5667|.|sp|P98163|
YL_DROME Putative vitellogenin receptor OS=Drosophila melanogaster
GN=yl PE=1 SV=2|e=0|from=436|to=5412|frame=-1|Fragment_12
VNGG
>Locus_539_Transcript_3/3_Confidence_0.714_Length_5667|.|sp|P98163|
YL_DROME Putative vitellogenin receptor OS=Drosophila melanogaster
GN=yl PE=1 SV=2|e=0|from=436|to=5412|frame=-1|Fragment_13
EALLLFSSRSNIRGLYLES
>Locus_539_Transcript_3/3_Confidence_0.714_Length_5667|.|sp|P98163|
YL_DROME Putative vitellogenin receptor OS=Drosophila melanogaster
GN=yl PE=1 SV=2|e=0|from=436|to=5412|frame=-1|Fragment_14
KILFQVIKNQRQIIGVEYDGNHVYWTDVHLGTESILRSLEDGSQMEVLLTSGLSAPEDLA
LDWLTGNIYFTDAELMHIGVCSNDGKHCTIIVNENIHKPRAIALYPGRGQMFWSDWGDNP
QIATSSMDGSSPKLFVSTNLHWPNGISIDIPNKRLYWVDAKLQTIESINLDGTDRRIVLK
GVTKHPYAIAIFEDQIYWSDWDSKSIQSCNKFTCKNRVTVAKESKEYIYGIHIYHSALQP
KHDHPCMKSSCSHLCLIKSAH
>Locus_539_Transcript_3/3_Confidence_0.714_Length_5667|.|sp|P98163|
YL_DROME Putative vitellogenin receptor OS=Drosophila melanogaster
GN=yl PE=1 SV=2|e=0|from=436|to=5412|frame=-1|Fragment_15
EYSCACPEGKELSADKTSCRETQRQSELIIGAGNLMVSLMHQKLGRHEL
>Locus_539_Transcript_3/3_Confidence_0.714_Length_5667|.|sp|P98163|
YL_DROME Putative vitellogenin receptor OS=Drosophila melanogaster
GN=yl PE=1 SV=2|e=0|from=436|to=5412|frame=-1|Fragment_16
HGVPMKINMDVLTYNSLNGEIIVADNNKKFIYTVDMYTTKSSRLTDIKSGEVEAMHFDNL
ANNLFWIDSVQQTIEVLHLDTGVKGFILHGLGDQVPVGFAIVPSHGIMFVAFRSHDHTHI
DRMLLDGSGIRTHVVEDGLGRWGKISMVYDHDLQRVFWAVEETGRIESSSVDGDDRHGFR
HTKDKPIAIAILGNDLFWTTAGNRRLYWADKHNLGNGFNKKLTIDIER
>Locus_539_Transcript_3/3_Confidence_0.714_Length_5667|.|sp|P98163|
YL_DROME Putative vitellogenin receptor OS=Drosophila melanogaster
GN=yl PE=1 SV=2|e=0|from=436|to=5412|frame=-1|Fragment_17
NLKSVPLTAITPRTISNHLCIHENGGCSHICIPMMQGKISCHCPVGLSLVPPYNMSCETI
IHCTGEQFRCSDTNKCIPMIFRCNGNKDCDFGEDESDCE
>Locus_539_Transcript_3/3_Confidence_0.714_Length_5667|.|sp|P98163|
YL_DROME Putative vitellogenin receptor OS=Drosophila melanogaster
GN=yl PE=1 SV=2|e=0|from=436|to=5412|frame=-1|Fragment_18

RYKDRQSCAVNEFQCNDG
>Locus_539_Transcript_3/3_Confidence_0.714_Length_5667|.|sp|P98163|
YL_DROME Putative vitellogenin receptor OS=Drosophila melanogaster
GN=yl PE=1 SV=2|e=0|from=436|to=5412|frame=-1|Fragment_19
ICIEYHYKCDKHFDCPDKSDEENCENYVSSSKCTHAQFECANGQCIDNRLLCDVLPDCLD
GSDEKMCDHVT
>Locus_539_Transcript_3/3_Confidence_0.714_Length_5667|.|sp|P98163|
YL_DROME Putative vitellogenin receptor OS=Drosophila melanogaster
GN=yl PE=1 SV=2|e=0|from=436|to=5412|frame=-1|Fragment_20
CESNMFQCQSGSCIPSSWECDGEIDCTDASDEHTFCEPNTCKDNQFTCTNGKCIDMDLKC
DHEDDCGDSSDEIRCPSKEKESTTETDFIDLITCEPTQYTCEES
>Locus_539_Transcript_3/3_Confidence_0.714_Length_5667|.|sp|P98163|
YL_DROME Putative vitellogenin receptor OS=Drosophila melanogaster
GN=yl PE=1 SV=2|e=0|from=436|to=5412|frame=-1|Fragment_21
GACLPEIARCNGTAECKYGEDERECTN
>Locus_539_Transcript_3/3_Confidence_0.714_Length_5667|.|sp|P98163|
YL_DROME Putative vitellogenin receptor OS=Drosophila melanogaster
GN=yl PE=1 SV=2|e=0|from=436|to=5412|frame=-1|Fragment_22
CQLDEFTCNSG
>Locus_539_Transcript_3/3_Confidence_0.714_Length_5667|.|sp|P98163|
YL_DROME Putative vitellogenin receptor OS=Drosophila melanogaster
GN=yl PE=1 SV=2|e=0|from=436|to=5412|frame=-1|Fragment_23
QCIQRKWVCDKTRDCNDGSDETS
>Locus_539_Transcript_3/3_Confidence_0.714_Length_5667|.|sp|P98163|
YL_DROME Putative vitellogenin receptor OS=Drosophila melanogaster
GN=yl PE=1 SV=2|e=0|from=436|to=5412|frame=-1|Fragment_24
ESCSAVTTVQPVHSQFLPVCDEDSFRCKSGGCVDFLFVCNGIPDCFDGSDENGACNTSCD
AIK
>Locus_539_Transcript_3/3_Confidence_0.714_Length_5667|.|sp|P98163|
YL_DROME Putative vitellogenin receptor OS=Drosophila melanogaster
GN=yl PE=1 SV=2|e=0|from=436|to=5412|frame=-1|Fragment_25
CKHKCSPTPNGPQCSCHEGYHLAGDHLSCTDINECKAELQQCGQICTNSAGSFHCSCYPE
FMLRSDKRSCKATGDQMSILYSTSNQIRELTLSPPSVKILFNRNIPKILSLDVSKHTSTL
YFSLHLISQIYRLDIKSGDVQYIMNIGKPMKLAVDWITQNIYFISEDDLHKTLRVCHIEN
AACAILMKFDKNIAVPELVVDPISKYIFYTVTHWWVYDNPITILYRANLDGSHVIELRNE
KLNLISGIGIDHHQKTVYYCDKHKNTIESVSY
>Locus_539_Transcript_3/3_Confidence_0.714_Length_5667|.|sp|P98163|
YL_DROME Putative vitellogenin receptor OS=Drosophila melanogaster
GN=yl PE=1 SV=2|e=0|from=436|to=5412|frame=-1|Fragment_26
NGSDHR
>Locus_539_Transcript_3/3_Confidence_0.714_Length_5667|.|sp|P98163|
YL_DROME Putative vitellogenin receptor OS=Drosophila melanogaster
GN=yl PE=1 SV=2|e=0|from=436|to=5412|frame=-1|Fragment_27
ILVKEQKLLTAPNGLDVFENYAYTLVYGSTRMIKCRLFGNYACTVIDAYISNADSFKISQ
YSKQ
>Locus_539_Transcript_3/3_Confidence_0.714_Length_5667|.|sp|P98163|
YL_DROME Putative vitellogenin receptor OS=Drosophila melanogaster
GN=yl PE=1 SV=2|e=0|from=436|to=5412|frame=-1|Fragment_28
LLHENACENSNCMYLCVPADSGPKCICKNGLMIAPGSSC
>Locus_539_Transcript_2/3_Confidence_0.714_Length_5667|.|sp|P98163|
YL_DROME Putative vitellogenin receptor OS=Drosophila melanogaster
GN=yl PE=1 SV=2|e=1.95083E-67|from=3505|to=5418|frame=-1|Fragment_1
KECLTSEYTCGDGECISKTRICDGIKDCKDDSDEHHCEMYRCEEPDWYRCADGECISSSF
RCDKSMDCPSGSDEQNCEDFVYTPP

>Locus_539_Transcript_2/3_Confidence_0.714_Length_5667|.|sp|P98163|
YL_DROME Putative vitellogenin receptor OS=Drosophila melanogaster
GN=yl PE=1 SV=2|e=1.95083E-67|from=3505|to=5418|frame=-1|Fragment_2
STNHSCEHNEWMCAD
>Locus_539_Transcript_2/3_Confidence_0.714_Length_5667|.|sp|P98163|
YL_DROME Putative vitellogenin receptor OS=Drosophila melanogaster
GN=yl PE=1 SV=2|e=1.95083E-67|from=3505|to=5418|frame=-1|Fragment_3
RMCIPLEFVCNGDFDCMDKSDEDMGCKDQHACDGFRCRNG
>Locus_539_Transcript_2/3_Confidence_0.714_Length_5667|.|sp|P98163|
YL_DROME Putative vitellogenin receptor OS=Drosophila melanogaster
GN=yl PE=1 SV=2|e=1.95083E-67|from=3505|to=5418|frame=-1|Fragment_4
HCISEDWVCDGAIDCADGSDEIDCDSRPVTFMCILEKDHY
>Locus_539_Transcript_2/3_Confidence_0.714_Length_5667|.|sp|P98163|
YL_DROME Putative vitellogenin receptor OS=Drosophila melanogaster
GN=yl PE=1 SV=2|e=1.95083E-67|from=3505|to=5418|frame=-1|Fragment_5
PCR
>Locus_539_Transcript_2/3_Confidence_0.714_Length_5667|.|sp|P98163|
YL_DROME Putative vitellogenin receptor OS=Drosophila melanogaster
GN=yl PE=1 SV=2|e=1.95083E-67|from=3505|to=5418|frame=-1|Fragment_6
DNSSCIPINKVCDNIPHCKDKSDEGVGCQQDCTSLS
>Locus_539_Transcript_2/3_Confidence_0.714_Length_5667|.|sp|P98163|
YL_DROME Putative vitellogenin receptor OS=Drosophila melanogaster
GN=yl PE=1 SV=2|e=1.95083E-67|from=3505|to=5418|frame=-1|Fragment_7
CAKHCVKTPEGPRCVCPSGFHNPLNSDFSHMHKNWQYNNNDNCTDINECAIHGMCSQRCE
NTIGSYKCLCDMKYTLSSDKKSCKVNGGEALLLFSSRSNIRGLYLESKILFQVI
>Locus_539_Transcript_2/3_Confidence_0.714_Length_5667|.|sp|P98163|
YL_DROME Putative vitellogenin receptor OS=Drosophila melanogaster
GN=yl PE=1 SV=2|e=1.95083E-67|from=3505|to=5418|frame=-1|Fragment_8
KNQRQIIGVEY
>Locus_539_Transcript_2/3_Confidence_0.714_Length_5667|.|sp|P98163|
YL_DROME Putative vitellogenin receptor OS=Drosophila melanogaster
GN=yl PE=1 SV=2|e=1.95083E-67|from=3505|to=5418|frame=-1|Fragment_9
DGNHVYWTDVHLGTESILRSLEDGSQMEVLLTSGLSAPEDLALDWLTGNIYFTDAELMHI
GVCSNDGKHCTIIVNENIHKPRAI
>Locus_539_Transcript_2/3_Confidence_0.714_Length_5667|.|sp|P98163|
YL_DROME Putative vitellogenin receptor OS=Drosophila melanogaster
GN=yl PE=1 SV=2|e=1.95083E-67|from=3505|to=5418|frame=-1|Fragment_10
ALYPGRGQMFW
>Locus_539_Transcript_2/3_Confidence_0.714_Length_5667|.|sp|P98163|
YL_DROME Putative vitellogenin receptor OS=Drosophila melanogaster
GN=yl PE=1 SV=2|e=1.95083E-67|from=3505|to=5418|frame=-1|Fragment_11
SDWGD
>Locus_539_Transcript_2/3_Confidence_0.714_Length_5667|.|sp|P98163|
YL_DROME Putative vitellogenin receptor OS=Drosophila melanogaster
GN=yl PE=1 SV=2|e=1.95083E-67|from=3505|to=5418|frame=-1|Fragment_12
NPQIATSSMDGSSPKLFVSTNLHWPNGISIDIPNKRLYWVDAKLQTIESINL
>Locus_539_Transcript_2/3_Confidence_0.714_Length_5667|.|sp|P98163|
YL_DROME Putative vitellogenin receptor OS=Drosophila melanogaster
GN=yl PE=1 SV=2|e=1.95083E-67|from=3505|to=5418|frame=-1|Fragment_13
DGTDRR
>Locus_539_Transcript_2/3_Confidence_0.714_Length_5667|.|sp|P98163|
YL_DROME Putative vitellogenin receptor OS=Drosophila melanogaster
GN=yl PE=1 SV=2|e=1.95083E-67|from=3505|to=5418|frame=-1|Fragment_14
IVLK

>Locus_539_Transcript_2/3_Confidence_0.714_Length_5667|.|sp|P98163|
YL_DROME Putative vitellogenin receptor OS=Drosophila melanogaster
GN=yl PE=1 SV=2|e=1.95083E-67|from=3505|to=5418|frame=-1|Fragment_15
GVTKHPYAIAIFEDQIYWSDWDSKSIQSC
>Locus_539_Transcript_2/3_Confidence_0.714_Length_5667|.|sp|P98163|
YL_DROME Putative vitellogenin receptor OS=Drosophila melanogaster
GN=yl PE=1 SV=2|e=1.95083E-67|from=3505|to=5418|frame=-1|Fragment_16
NKFTCKNRVTVAKESKEYIYGIHIYHSALQPKHDHPCMKSSCSHLCLIKSAHEYSCACPE
GKELSADKTSCRETQRQSELIIGAGNLMVSLMHQKLGRHELHG
>Locus_539_Transcript_3/3_Confidence_0.714_Length_5667|.|sp|P98163|
YL_DROME Putative vitellogenin receptor OS=Drosophila melanogaster
GN=yl PE=1 SV=2|e=1.95083E-67|from=3505|to=5418|frame=-1|Fragment_1
KECLTSEYTCGDGECISKTRICDGIKDCKDDSDEHHCEMYRCEEPDWYRCADGECISSSF
RCDKSMDCPSGSDEQNCEDFVYTPP
>Locus_539_Transcript_3/3_Confidence_0.714_Length_5667|.|sp|P98163|
YL_DROME Putative vitellogenin receptor OS=Drosophila melanogaster
GN=yl PE=1 SV=2|e=1.95083E-67|from=3505|to=5418|frame=-1|Fragment_2
STNHSCEHNEWMCAD
>Locus_539_Transcript_3/3_Confidence_0.714_Length_5667|.|sp|P98163|
YL_DROME Putative vitellogenin receptor OS=Drosophila melanogaster
GN=yl PE=1 SV=2|e=1.95083E-67|from=3505|to=5418|frame=-1|Fragment_3
RMCIPLEFVCNGDFDCMDKSDEDMGCKDQHACDGFRCRNG
>Locus_539_Transcript_3/3_Confidence_0.714_Length_5667|.|sp|P98163|
YL_DROME Putative vitellogenin receptor OS=Drosophila melanogaster
GN=yl PE=1 SV=2|e=1.95083E-67|from=3505|to=5418|frame=-1|Fragment_4
HCISEDWVCDGAIDCADGSDEIDCDSRPVTFMCILEKDHY
>Locus_539_Transcript_3/3_Confidence_0.714_Length_5667|.|sp|P98163|
YL_DROME Putative vitellogenin receptor OS=Drosophila melanogaster
GN=yl PE=1 SV=2|e=1.95083E-67|from=3505|to=5418|frame=-1|Fragment_5
PCR
>Locus_539_Transcript_3/3_Confidence_0.714_Length_5667|.|sp|P98163|
YL_DROME Putative vitellogenin receptor OS=Drosophila melanogaster
GN=yl PE=1 SV=2|e=1.95083E-67|from=3505|to=5418|frame=-1|Fragment_6
DNSSCIPINKVCDNIPHCKDKSDEGVGCQQDCTSLS
>Locus_539_Transcript_3/3_Confidence_0.714_Length_5667|.|sp|P98163|
YL_DROME Putative vitellogenin receptor OS=Drosophila melanogaster
GN=yl PE=1 SV=2|e=1.95083E-67|from=3505|to=5418|frame=-1|Fragment_7
CAKHCVKTPEGPRCVCPSGFHNPLNSDFSHMHKNWQYNNNDNCTDINECAIHGMCSQRCE
NTIGSYKCLCDMKYTLSSDKKSCKVNGGEALLLFSSRSNIRGLYLESKILFQVI
>Locus_539_Transcript_3/3_Confidence_0.714_Length_5667|.|sp|P98163|
YL_DROME Putative vitellogenin receptor OS=Drosophila melanogaster
GN=yl PE=1 SV=2|e=1.95083E-67|from=3505|to=5418|frame=-1|Fragment_8
KNQRQIIGVEY
>Locus_539_Transcript_3/3_Confidence_0.714_Length_5667|.|sp|P98163|
YL_DROME Putative vitellogenin receptor OS=Drosophila melanogaster
GN=yl PE=1 SV=2|e=1.95083E-67|from=3505|to=5418|frame=-1|Fragment_9
DGNHVYWTDVHLGTESILRSLEDGSQMEVLLTSGLSAPEDLALDWLTGNIYFTDAELMHI
GVCSNDGKHCTIIVNENIHKPRAI
>Locus_539_Transcript_3/3_Confidence_0.714_Length_5667|.|sp|P98163|
YL_DROME Putative vitellogenin receptor OS=Drosophila melanogaster
GN=yl PE=1 SV=2|e=1.95083E-67|from=3505|to=5418|frame=-1|Fragment_10
ALYPGRGQMFW
>Locus_539_Transcript_3/3_Confidence_0.714_Length_5667|.|sp|P98163|
YL_DROME Putative vitellogenin receptor OS=Drosophila melanogaster

GN=yl PE=1 SV=2|e=1.95083E-67|from=3505|to=5418|frame=-1|Fragment_11
SDWGD
>Locus_539_Transcript_3/3_Confidence_0.714_Length_5667|.|sp|P98163|
YL_DROME Putative vitellogenin receptor OS=Drosophila melanogaster
GN=yl PE=1 SV=2|e=1.95083E-67|from=3505|to=5418|frame=-1|Fragment_12
NPQIATSSMDGSSPKLFVSTNLHWPNGISIDIPNKRLYWVDAKLQTIESINL
>Locus_539_Transcript_3/3_Confidence_0.714_Length_5667|.|sp|P98163|
YL_DROME Putative vitellogenin receptor OS=Drosophila melanogaster
GN=yl PE=1 SV=2|e=1.95083E-67|from=3505|to=5418|frame=-1|Fragment_13
DGTDRR
>Locus_539_Transcript_3/3_Confidence_0.714_Length_5667|.|sp|P98163|
YL_DROME Putative vitellogenin receptor OS=Drosophila melanogaster
GN=yl PE=1 SV=2|e=1.95083E-67|from=3505|to=5418|frame=-1|Fragment_14
IVLK
>Locus_539_Transcript_3/3_Confidence_0.714_Length_5667|.|sp|P98163|
YL_DROME Putative vitellogenin receptor OS=Drosophila melanogaster
GN=yl PE=1 SV=2|e=1.95083E-67|from=3505|to=5418|frame=-1|Fragment_15
GVTKHPYAIAIFEDQIYWSDWDSKSIQSC
>Locus_539_Transcript_3/3_Confidence_0.714_Length_5667|.|sp|P98163|
YL_DROME Putative vitellogenin receptor OS=Drosophila melanogaster
GN=yl PE=1 SV=2|e=1.95083E-67|from=3505|to=5418|frame=-1|Fragment_16
NKFTCKNRVTVAKESKEYIYGIHIYHSALQPKHDHPCMKSSCSHLCLIKSAHEYSCACPE
GKELSADKTSCRETQRQSELIIGAGNLMVSLMHQKLGRHELHG
>Locus_539_Transcript_2/3_Confidence_0.714_Length_5667|.|sp|P98163|
YL_DROME Putative vitellogenin receptor OS=Drosophila melanogaster
GN=yl PE=1 SV=2|e=2.46781E-62|from=316|to=2391|frame=-1|Fragment_1
SSSKCTHAQFECANGQCIDNRLLCDVLPDCLDGSDEKMCDHVTCE
>Locus_539_Transcript_2/3_Confidence_0.714_Length_5667|.|sp|P98163|
YL_DROME Putative vitellogenin receptor OS=Drosophila melanogaster
GN=yl PE=1 SV=2|e=2.46781E-62|from=316|to=2391|frame=-1|Fragment_2
SNMFQCQS
>Locus_539_Transcript_2/3_Confidence_0.714_Length_5667|.|sp|P98163|
YL_DROME Putative vitellogenin receptor OS=Drosophila melanogaster
GN=yl PE=1 SV=2|e=2.46781E-62|from=316|to=2391|frame=-1|Fragment_3
GSCIPSSWECDGEIDCTDASDE
>Locus_539_Transcript_2/3_Confidence_0.714_Length_5667|.|sp|P98163|
YL_DROME Putative vitellogenin receptor OS=Drosophila melanogaster
GN=yl PE=1 SV=2|e=2.46781E-62|from=316|to=2391|frame=-1|Fragment_4
HTFCEPNTCKDNQFTCTNGK
>Locus_539_Transcript_2/3_Confidence_0.714_Length_5667|.|sp|P98163|
YL_DROME Putative vitellogenin receptor OS=Drosophila melanogaster
GN=yl PE=1 SV=2|e=2.46781E-62|from=316|to=2391|frame=-1|Fragment_5
CIDMDLKCDHEDDCGDSSDEIR
>Locus_539_Transcript_2/3_Confidence_0.714_Length_5667|.|sp|P98163|
YL_DROME Putative vitellogenin receptor OS=Drosophila melanogaster
GN=yl PE=1 SV=2|e=2.46781E-62|from=316|to=2391|frame=-1|Fragment_6
CPSKEKESTTETDFIDLITCEPTQYTCEESGACLPEIA
>Locus_539_Transcript_2/3_Confidence_0.714_Length_5667|.|sp|P98163|
YL_DROME Putative vitellogenin receptor OS=Drosophila melanogaster
GN=yl PE=1 SV=2|e=2.46781E-62|from=316|to=2391|frame=-1|Fragment_7
RCNGTAECKYGEDERECTNC
>Locus_539_Transcript_2/3_Confidence_0.714_Length_5667|.|sp|P98163|
YL_DROME Putative vitellogenin receptor OS=Drosophila melanogaster
GN=yl PE=1 SV=2|e=2.46781E-62|from=316|to=2391|frame=-1|Fragment_8

QLDEFTC
>Locus_539_Transcript_2/3_Confidence_0.714_Length_5667|.|sp|P98163|YL_DROME Putative vitellogenin receptor OS=Drosophila melanogaster GN=yl PE=1 SV=2|e=2.46781E-62|from=316|to=2391|frame=-1|Fragment_9
NSGQCIQRKWVCDKTRDCNDGSDETSESCSAVTTVQPVHSQFLPVCDEDSFRCKSGGCVD
FLFVCNGIPDCFDGSDENGACNTSCDAIKCK
>Locus_539_Transcript_2/3_Confidence_0.714_Length_5667|.|sp|P98163|YL_DROME Putative vitellogenin receptor OS=Drosophila melanogaster GN=yl PE=1 SV=2|e=2.46781E-62|from=316|to=2391|frame=-1|Fragment_10
HKCSPTP
>Locus_539_Transcript_2/3_Confidence_0.714_Length_5667|.|sp|P98163|YL_DROME Putative vitellogenin receptor OS=Drosophila melanogaster GN=yl PE=1 SV=2|e=2.46781E-62|from=316|to=2391|frame=-1|Fragment_11
NGPQCSCHEGYHLAGDHLSCTDINECKAELQQCGQICTNSAGSFHCSCYPEFMLRSDKRS
CKAT
>Locus_539_Transcript_2/3_Confidence_0.714_Length_5667|.|sp|P98163|YL_DROME Putative vitellogenin receptor OS=Drosophila melanogaster GN=yl PE=1 SV=2|e=2.46781E-62|from=316|to=2391|frame=-1|Fragment_12
GDQMSILYSTSNQIRELTLSPPSVKILFNRNIPKILSLDVSKHTSTLYFSLHL
>Locus_539_Transcript_2/3_Confidence_0.714_Length_5667|.|sp|P98163|YL_DROME Putative vitellogenin receptor OS=Drosophila melanogaster GN=yl PE=1 SV=2|e=2.46781E-62|from=316|to=2391|frame=-1|Fragment_13
ISQIYRLDIKSGDVQYIMNIG
>Locus_539_Transcript_2/3_Confidence_0.714_Length_5667|.|sp|P98163|YL_DROME Putative vitellogenin receptor OS=Drosophila melanogaster GN=yl PE=1 SV=2|e=2.46781E-62|from=316|to=2391|frame=-1|Fragment_14
KPMKLAVDWITQNIYFISEDDLHKTLRVCHIENAACAILMKFDKNIAVPELVVDPISKYI
FYTVTHWWVYDNPITILYRANLDGSHVIELRNEKLNLISGIGIDHHQKTVYYCDKHKNTI
ESVSYNGSDHRILVKEQKLLTAPNGLDVFENYAYTLVYGSTRMIKCRLFGNYACTVIDAY
ISNADSFKISQYSKQLLHENACENSNCMYLCV
>Locus_539_Transcript_2/3_Confidence_0.714_Length_5667|.|sp|P98163|YL_DROME Putative vitellogenin receptor OS=Drosophila melanogaster GN=yl PE=1 SV=2|e=2.46781E-62|from=316|to=2391|frame=-1|Fragment_15
PADSGPKCICKNGLMIAPGSSCVDDMD
>Locus_539_Transcript_2/3_Confidence_0.714_Length_5667|.|sp|P98163|YL_DROME Putative vitellogenin receptor OS=Drosophila melanogaster GN=yl PE=1 SV=2|e=2.46781E-62|from=316|to=2391|frame=-1|Fragment_16
DHPQFGTMASNEQSTSVAVPIFLSLLLFGAVAGSI
>Locus_539_Transcript_3/3_Confidence_0.714_Length_5667|.|sp|P98163|YL_DROME Putative vitellogenin receptor OS=Drosophila melanogaster GN=yl PE=1 SV=2|e=2.46781E-62|from=316|to=2391|frame=-1|Fragment_1
SSSKCTHAQFECANGQCIDNRLLCDVLPDCLDGSDEKMCDHVTCE
>Locus_539_Transcript_3/3_Confidence_0.714_Length_5667|.|sp|P98163|YL_DROME Putative vitellogenin receptor OS=Drosophila melanogaster GN=yl PE=1 SV=2|e=2.46781E-62|from=316|to=2391|frame=-1|Fragment_2
SNMFQCQS
>Locus_539_Transcript_3/3_Confidence_0.714_Length_5667|.|sp|P98163|YL_DROME Putative vitellogenin receptor OS=Drosophila melanogaster GN=yl PE=1 SV=2|e=2.46781E-62|from=316|to=2391|frame=-1|Fragment_3
GSCIPSSWECDGEIDCTDASDE
>Locus_539_Transcript_3/3_Confidence_0.714_Length_5667|.|sp|P98163|YL_DROME Putative vitellogenin receptor OS=Drosophila melanogaster GN=yl PE=1 SV=2|e=2.46781E-62|from=316|to=2391|frame=-1|Fragment_4
HTFCEPNTCKDNQFTCTNGK

>Locus_539_Transcript_3/3_Confidence_0.714_Length_5667|.|sp|P98163|
YL_DROME Putative vitellogenin receptor OS=Drosophila melanogaster
GN=yl PE=1 SV=2|e=2.46781E-62|from=316|to=2391|frame=-1|Fragment_5
CIDMDLKCDHEDDCGDSSDEIR
>Locus_539_Transcript_3/3_Confidence_0.714_Length_5667|.|sp|P98163|
YL_DROME Putative vitellogenin receptor OS=Drosophila melanogaster
GN=yl PE=1 SV=2|e=2.46781E-62|from=316|to=2391|frame=-1|Fragment_6
CPSKEKESTTETDFIDLITCEPTQYTCEESGACLPEIA
>Locus_539_Transcript_3/3_Confidence_0.714_Length_5667|.|sp|P98163|
YL_DROME Putative vitellogenin receptor OS=Drosophila melanogaster
GN=yl PE=1 SV=2|e=2.46781E-62|from=316|to=2391|frame=-1|Fragment_7
RCNGTAECKYGEDERECTNC
>Locus_539_Transcript_3/3_Confidence_0.714_Length_5667|.|sp|P98163|
YL_DROME Putative vitellogenin receptor OS=Drosophila melanogaster
GN=yl PE=1 SV=2|e=2.46781E-62|from=316|to=2391|frame=-1|Fragment_8
QLDEFTC
>Locus_539_Transcript_3/3_Confidence_0.714_Length_5667|.|sp|P98163|
YL_DROME Putative vitellogenin receptor OS=Drosophila melanogaster
GN=yl PE=1 SV=2|e=2.46781E-62|from=316|to=2391|frame=-1|Fragment_9
NSGQCIQRKWVCDKTRDCNDGSDETSESCSAVTTVQPVHSQFLPVCDEDSFRCKSGGCVD
FLFVCNGIPDCFDGSDENGACNTSCDAIKCK
>Locus_539_Transcript_3/3_Confidence_0.714_Length_5667|.|sp|P98163|
YL_DROME Putative vitellogenin receptor OS=Drosophila melanogaster
GN=yl PE=1 SV=2|e=2.46781E-62|from=316|to=2391|frame=-1|Fragment_10
HKCSPTP
>Locus_539_Transcript_3/3_Confidence_0.714_Length_5667|.|sp|P98163|
YL_DROME Putative vitellogenin receptor OS=Drosophila melanogaster
GN=yl PE=1 SV=2|e=2.46781E-62|from=316|to=2391|frame=-1|Fragment_11
NGPQCSCHEGYHLAGDHLSCTDINECKAELQQCGQICTNSAGSFHCSCYPEFMLRSDKRS
CKAT
>Locus_539_Transcript_3/3_Confidence_0.714_Length_5667|.|sp|P98163|
YL_DROME Putative vitellogenin receptor OS=Drosophila melanogaster
GN=yl PE=1 SV=2|e=2.46781E-62|from=316|to=2391|frame=-1|Fragment_12
GDQMSILYSTSNQIRELTLSPPSVKILFNRNIPKILSLDVSKHTSTLYFSLHL
>Locus_539_Transcript_3/3_Confidence_0.714_Length_5667|.|sp|P98163|
YL_DROME Putative vitellogenin receptor OS=Drosophila melanogaster
GN=yl PE=1 SV=2|e=2.46781E-62|from=316|to=2391|frame=-1|Fragment_13
ISQIYRLDIKSGDVQYIMNIG
>Locus_539_Transcript_3/3_Confidence_0.714_Length_5667|.|sp|P98163|
YL_DROME Putative vitellogenin receptor OS=Drosophila melanogaster
GN=yl PE=1 SV=2|e=2.46781E-62|from=316|to=2391|frame=-1|Fragment_14
KPMKLAVDWITQNIYFISEDDLHKTLRVCHIENAACAILMKFDKNIAVPELVVDPISKYI
FYTVTHWWVYDNPITILYRANLDGSHVIELRNEKLNLISGIGIDHHQKTVYYCDKHKNTI
ESVSYNGSDHRILVKEQKLLTAPNGLDVFENYAYTLVYGSTRMIKCRLFGNYACTVIDAY
ISNADSFKISQYSKQLLHENACENSNCMYLCV
>Locus_539_Transcript_3/3_Confidence_0.714_Length_5667|.|sp|P98163|
YL_DROME Putative vitellogenin receptor OS=Drosophila melanogaster
GN=yl PE=1 SV=2|e=2.46781E-62|from=316|to=2391|frame=-1|Fragment_15
PADSGPKCICKNGLMIAPGSSCVDDMD
>Locus_539_Transcript_3/3_Confidence_0.714_Length_5667|.|sp|P98163|
YL_DROME Putative vitellogenin receptor OS=Drosophila melanogaster
GN=yl PE=1 SV=2|e=2.46781E-62|from=316|to=2391|frame=-1|Fragment_16
DHPQFGTMASNEQSTSVAVPIFLSLLLFGAVAGSI
>Locus_539_Transcript_2/3_Confidence_0.714_Length_5667|.|sp|P98163|

YL_DROME Putative vitellogenin receptor OS=Drosophila melanogaster
GN=yl PE=1 SV=2|e=3.14358E-33|from=4708|to=5451|frame=-1|Fragment_1
VCPRSGFVFDHK
>Locus_539_Transcript_2/3_Confidence_0.714_Length_5667|.|sp|P98163|
YL_DROME Putative vitellogenin receptor OS=Drosophila melanogaster
GN=yl PE=1 SV=2|e=3.14358E-33|from=4708|to=5451|frame=-1|Fragment_2
ECLTSEYTCGDGECISKTRICDGIKDCKDDSDEHHC
>Locus_539_Transcript_2/3_Confidence_0.714_Length_5667|.|sp|P98163|
YL_DROME Putative vitellogenin receptor OS=Drosophila melanogaster
GN=yl PE=1 SV=2|e=3.14358E-33|from=4708|to=5451|frame=-1|Fragment_3
EMYRCE
>Locus_539_Transcript_2/3_Confidence_0.714_Length_5667|.|sp|P98163|
YL_DROME Putative vitellogenin receptor OS=Drosophila melanogaster
GN=yl PE=1 SV=2|e=3.14358E-33|from=4708|to=5451|frame=-1|Fragment_4
EPDWYRCADGE
>Locus_539_Transcript_2/3_Confidence_0.714_Length_5667|.|sp|P98163|
YL_DROME Putative vitellogenin receptor OS=Drosophila melanogaster
GN=yl PE=1 SV=2|e=3.14358E-33|from=4708|to=5451|frame=-1|Fragment_5
CISSSFRCDKSMDCPSGSDEQNCEDFVYTPPSTNHSCEHNEWMCADRMCIPLEFVCNGDF
DCMDKSDEDMGCKDQHACDG
>Locus_539_Transcript_2/3_Confidence_0.714_Length_5667|.|sp|P98163|
YL_DROME Putative vitellogenin receptor OS=Drosophila melanogaster
GN=yl PE=1 SV=2|e=3.14358E-33|from=4708|to=5451|frame=-1|Fragment_6
FRCRNGHCISEDWVCDGAIDCADGSDEID
>Locus_539_Transcript_2/3_Confidence_0.714_Length_5667|.|sp|P98163|
YL_DROME Putative vitellogenin receptor OS=Drosophila melanogaster
GN=yl PE=1 SV=2|e=3.14358E-33|from=4708|to=5451|frame=-1|Fragment_7
CDSR
>Locus_539_Transcript_2/3_Confidence_0.714_Length_5667|.|sp|P98163|
YL_DROME Putative vitellogenin receptor OS=Drosophila melanogaster
GN=yl PE=1 SV=2|e=3.14358E-33|from=4708|to=5451|frame=-1|Fragment_8
PVTFMCILEKDHYPCRDNSSCIPINKVCDNIPHCKDKSDE
>Locus_539_Transcript_2/3_Confidence_0.714_Length_5667|.|sp|P98163|
YL_DROME Putative vitellogenin receptor OS=Drosophila melanogaster
GN=yl PE=1 SV=2|e=3.14358E-33|from=4708|to=5451|frame=-1|Fragment_9
GVGCQQDCTSLSCAKHCVKTPEGPRCVCPS
>Locus_539_Transcript_3/3_Confidence_0.714_Length_5667|.|sp|P98163|
YL_DROME Putative vitellogenin receptor OS=Drosophila melanogaster
GN=yl PE=1 SV=2|e=3.14358E-33|from=4708|to=5451|frame=-1|Fragment_1
VCPRSGFVFDHK
>Locus_539_Transcript_3/3_Confidence_0.714_Length_5667|.|sp|P98163|
YL_DROME Putative vitellogenin receptor OS=Drosophila melanogaster
GN=yl PE=1 SV=2|e=3.14358E-33|from=4708|to=5451|frame=-1|Fragment_2
ECLTSEYTCGDGECISKTRICDGIKDCKDDSDEHHC
>Locus_539_Transcript_3/3_Confidence_0.714_Length_5667|.|sp|P98163|
YL_DROME Putative vitellogenin receptor OS=Drosophila melanogaster
GN=yl PE=1 SV=2|e=3.14358E-33|from=4708|to=5451|frame=-1|Fragment_3
EMYRCE
>Locus_539_Transcript_3/3_Confidence_0.714_Length_5667|.|sp|P98163|
YL_DROME Putative vitellogenin receptor OS=Drosophila melanogaster
GN=yl PE=1 SV=2|e=3.14358E-33|from=4708|to=5451|frame=-1|Fragment_4
EPDWYRCADGE
>Locus_539_Transcript_3/3_Confidence_0.714_Length_5667|.|sp|P98163|
YL_DROME Putative vitellogenin receptor OS=Drosophila melanogaster

GN=yl PE=1 SV=2|e=3.14358E-33|from=4708|to=5451|frame=-1|Fragment_5
CISSSFRCDKSMDCPSGSDEQNCEDFVYTPPSTNHSCEHNEWMCADRMCIPLEFVCNGDF
DCMDKSDEDMGCKDQHACDG
>Locus_539_Transcript_3/3_Confidence_0.714_Length_5667|.|sp|P98163|
YL_DROME Putative vitellogenin receptor OS=Drosophila melanogaster
GN=yl PE=1 SV=2|e=3.14358E-33|from=4708|to=5451|frame=-1|Fragment_6
FRCRNGHCISEDWVCDGAIDCADGSDEID
>Locus_539_Transcript_3/3_Confidence_0.714_Length_5667|.|sp|P98163|
YL_DROME Putative vitellogenin receptor OS=Drosophila melanogaster
GN=yl PE=1 SV=2|e=3.14358E-33|from=4708|to=5451|frame=-1|Fragment_7
CDSR
>Locus_539_Transcript_3/3_Confidence_0.714_Length_5667|.|sp|P98163|
YL_DROME Putative vitellogenin receptor OS=Drosophila melanogaster
GN=yl PE=1 SV=2|e=3.14358E-33|from=4708|to=5451|frame=-1|Fragment_8
PVTFMCILEKDHYPCRDNSSCIPINKVCDNIPHCKDKSDE
>Locus_539_Transcript_3/3_Confidence_0.714_Length_5667|.|sp|P98163|
YL_DROME Putative vitellogenin receptor OS=Drosophila melanogaster
GN=yl PE=1 SV=2|e=3.14358E-33|from=4708|to=5451|frame=-1|Fragment_9
GVGCQQDCTSLSCAKHCVKTPEGPRCVCPS
>Locus_539_Transcript_2/3_Confidence_0.714_Length_5667|.|sp|P98163|
YL_DROME Putative vitellogenin receptor OS=Drosophila melanogaster
GN=yl PE=1 SV=2|e=1.67462E-18|from=3829|to=5172|frame=-1|Fragment_1
TPPSTNHSCEHNEWMCADRMCIPLEFVCNGDFDCMDKSDEDMGCKDQHAC
>Locus_539_Transcript_2/3_Confidence_0.714_Length_5667|.|sp|P98163|
YL_DROME Putative vitellogenin receptor OS=Drosophila melanogaster
GN=yl PE=1 SV=2|e=1.67462E-18|from=3829|to=5172|frame=-1|Fragment_2
DGFRCRNGH
>Locus_539_Transcript_2/3_Confidence_0.714_Length_5667|.|sp|P98163|
YL_DROME Putative vitellogenin receptor OS=Drosophila melanogaster
GN=yl PE=1 SV=2|e=1.67462E-18|from=3829|to=5172|frame=-1|Fragment_3
CISEDWVCDGAIDCADGSDEIDCDSRP
>Locus_539_Transcript_2/3_Confidence_0.714_Length_5667|.|sp|P98163|
YL_DROME Putative vitellogenin receptor OS=Drosophila melanogaster
GN=yl PE=1 SV=2|e=1.67462E-18|from=3829|to=5172|frame=-1|Fragment_4
VTFMCILEKDHYPCRDNSSCIPINKVCDNIPHCKDKSDEGVGCQQD
>Locus_539_Transcript_2/3_Confidence_0.714_Length_5667|.|sp|P98163|
YL_DROME Putative vitellogenin receptor OS=Drosophila melanogaster
GN=yl PE=1 SV=2|e=1.67462E-18|from=3829|to=5172|frame=-1|Fragment_5
CTSLSCAKHCVKTPEG
>Locus_539_Transcript_2/3_Confidence_0.714_Length_5667|.|sp|P98163|
YL_DROME Putative vitellogenin receptor OS=Drosophila melanogaster
GN=yl PE=1 SV=2|e=1.67462E-18|from=3829|to=5172|frame=-1|Fragment_6
PRCVCPSGFHNPLNSDFSHMHKNWQYNNNDNCTDINE
>Locus_539_Transcript_2/3_Confidence_0.714_Length_5667|.|sp|P98163|
YL_DROME Putative vitellogenin receptor OS=Drosophila melanogaster
GN=yl PE=1 SV=2|e=1.67462E-18|from=3829|to=5172|frame=-1|Fragment_7
CAIHGMCSQRCENT
>Locus_539_Transcript_2/3_Confidence_0.714_Length_5667|.|sp|P98163|
YL_DROME Putative vitellogenin receptor OS=Drosophila melanogaster
GN=yl PE=1 SV=2|e=1.67462E-18|from=3829|to=5172|frame=-1|Fragment_8
IGSYKCLCDMK
>Locus_539_Transcript_2/3_Confidence_0.714_Length_5667|.|sp|P98163|
YL_DROME Putative vitellogenin receptor OS=Drosophila melanogaster
GN=yl PE=1 SV=2|e=1.67462E-18|from=3829|to=5172|frame=-1|Fragment_9

YTLSSDKKSCKVNGGEALLLFSSR
>Locus_539_Transcript_2/3_Confidence_0.714_Length_5667|.|sp|P98163|
YL_DROME Putative vitellogenin receptor OS=Drosophila melanogaster
GN=yl PE=1 SV=2|e=1.67462E-18|from=3829|to=5172|frame=-1|Fragment_10
SNIRGLYLESKILFQVIKNQRQIIGVEYDGNHVYWTDVHLGTESILRSLEDGSQMEVLLT
SGLSAPEDLALDWLTGNIYFTDAELMHIGVCSNDGKHC
>Locus_539_Transcript_2/3_Confidence_0.714_Length_5667|.|sp|P98163|
YL_DROME Putative vitellogenin receptor OS=Drosophila melanogaster
GN=yl PE=1 SV=2|e=1.67462E-18|from=3829|to=5172|frame=-1|Fragment_11
TIIVNENIHKPRAIALYPGRGQMFWSDWGDNPQIATSSMDGSSPKLFVSTNLHWPNGISI
DIPNKRLYWVDAKLQTIESINLDGTDRRIVLKGVTKHPYAIAIFEDQ
>Locus_539_Transcript_2/3_Confidence_0.714_Length_5667|.|sp|P98163|
YL_DROME Putative vitellogenin receptor OS=Drosophila melanogaster
GN=yl PE=1 SV=2|e=1.67462E-18|from=3829|to=5172|frame=-1|Fragment_12
IYWSDWDSK
>Locus_539_Transcript_3/3_Confidence_0.714_Length_5667|.|sp|P98163|
YL_DROME Putative vitellogenin receptor OS=Drosophila melanogaster
GN=yl PE=1 SV=2|e=1.67462E-18|from=3829|to=5172|frame=-1|Fragment_1
TPPSTNHSCEHNEWMCADRMCIPLEFVCNGDFDCMDKSDEDMGCKDQHAC
>Locus_539_Transcript_3/3_Confidence_0.714_Length_5667|.|sp|P98163|
YL_DROME Putative vitellogenin receptor OS=Drosophila melanogaster
GN=yl PE=1 SV=2|e=1.67462E-18|from=3829|to=5172|frame=-1|Fragment_2
DGFRCRNGH
>Locus_539_Transcript_3/3_Confidence_0.714_Length_5667|.|sp|P98163|
YL_DROME Putative vitellogenin receptor OS=Drosophila melanogaster
GN=yl PE=1 SV=2|e=1.67462E-18|from=3829|to=5172|frame=-1|Fragment_3
CISEDWVCDGAIDCADGSDEIDCDSRP
>Locus_539_Transcript_3/3_Confidence_0.714_Length_5667|.|sp|P98163|
YL_DROME Putative vitellogenin receptor OS=Drosophila melanogaster
GN=yl PE=1 SV=2|e=1.67462E-18|from=3829|to=5172|frame=-1|Fragment_4
VTFMCILEKDHYPCRDNSSCIPINKVCDNIPHCKDKSDEGVGCQQD
>Locus_539_Transcript_3/3_Confidence_0.714_Length_5667|.|sp|P98163|
YL_DROME Putative vitellogenin receptor OS=Drosophila melanogaster
GN=yl PE=1 SV=2|e=1.67462E-18|from=3829|to=5172|frame=-1|Fragment_5
CTSLSCAKHCVKTPEG
>Locus_539_Transcript_3/3_Confidence_0.714_Length_5667|.|sp|P98163|
YL_DROME Putative vitellogenin receptor OS=Drosophila melanogaster
GN=yl PE=1 SV=2|e=1.67462E-18|from=3829|to=5172|frame=-1|Fragment_6
PRCVCPSGFHNPLNSDFSHMHKNWQYNNNDNCTDINE
>Locus_539_Transcript_3/3_Confidence_0.714_Length_5667|.|sp|P98163|
YL_DROME Putative vitellogenin receptor OS=Drosophila melanogaster
GN=yl PE=1 SV=2|e=1.67462E-18|from=3829|to=5172|frame=-1|Fragment_7
CAIHGMCSQRCENT
>Locus_539_Transcript_3/3_Confidence_0.714_Length_5667|.|sp|P98163|
YL_DROME Putative vitellogenin receptor OS=Drosophila melanogaster
GN=yl PE=1 SV=2|e=1.67462E-18|from=3829|to=5172|frame=-1|Fragment_8
IGSYKCLCDMK
>Locus_539_Transcript_3/3_Confidence_0.714_Length_5667|.|sp|P98163|
YL_DROME Putative vitellogenin receptor OS=Drosophila melanogaster
GN=yl PE=1 SV=2|e=1.67462E-18|from=3829|to=5172|frame=-1|Fragment_9
YTLSSDKKSCKVNGGEALLLFSSR
>Locus_539_Transcript_3/3_Confidence_0.714_Length_5667|.|sp|P98163|
YL_DROME Putative vitellogenin receptor OS=Drosophila melanogaster
GN=yl PE=1 SV=2|e=1.67462E-18|from=3829|to=5172|frame=-1|Fragment_10

SNIRGLYLESKILFQVIKNQRQIIGVEYDGNHVYWTDVHLGTESILRSLEDGSQMEVLLT
SGLSAPEDLALDWLTGNIYFTDAELMHIGVCSNDGKHC
>Locus_539_Transcript_3/3_Confidence_0.714_Length_5667|.|sp|P98163|
YL_DROME Putative vitellogenin receptor OS=Drosophila melanogaster
GN=yl PE=1 SV=2|e=1.67462E-18|from=3829|to=5172|frame=-1|Fragment_11
TIIVNENIHKPRAIALYPGRGQMFWSDWGDNPQIATSSMDGSSPKLFVSTNLHWPNGISI
DIPNKRLYWVDAKLQTIESINLDGTDRRIVLKGVTKHPYAIAIFEDQ
>Locus_539_Transcript_3/3_Confidence_0.714_Length_5667|.|sp|P98163|
YL_DROME Putative vitellogenin receptor OS=Drosophila melanogaster
GN=yl PE=1 SV=2|e=1.67462E-18|from=3829|to=5172|frame=-1|Fragment_12
IYWSDWDSK
>Locus_539_Transcript_1/3_Confidence_0.714_Length_5649|.|sp|P98163|
YL_DROME Putative vitellogenin receptor OS=Drosophila melanogaster
GN=yl PE=1 SV=2|e=0|from=436|to=5394|frame=-1|Fragment_1
CLTSEYTCGDGECISKTRICDGIKDCKDDSDEHHCEMYRCEEPDWYRCAD
>Locus_539_Transcript_1/3_Confidence_0.714_Length_5649|.|sp|P98163|
YL_DROME Putative vitellogenin receptor OS=Drosophila melanogaster
GN=yl PE=1 SV=2|e=0|from=436|to=5394|frame=-1|Fragment_2
GECISSSFRCDKSMDCPSGSDEQNCE
>Locus_539_Transcript_1/3_Confidence_0.714_Length_5649|.|sp|P98163|
YL_DROME Putative vitellogenin receptor OS=Drosophila melanogaster
GN=yl PE=1 SV=2|e=0|from=436|to=5394|frame=-1|Fragment_3
DFVYTPPSTN
>Locus_539_Transcript_1/3_Confidence_0.714_Length_5649|.|sp|P98163|
YL_DROME Putative vitellogenin receptor OS=Drosophila melanogaster
GN=yl PE=1 SV=2|e=0|from=436|to=5394|frame=-1|Fragment_4
HSCEHNEWMCA
>Locus_539_Transcript_1/3_Confidence_0.714_Length_5649|.|sp|P98163|
YL_DROME Putative vitellogenin receptor OS=Drosophila melanogaster
GN=yl PE=1 SV=2|e=0|from=436|to=5394|frame=-1|Fragment_5
DRMCIPLEFVCNGDFDCMDKSDEDMGCKDQHAC
>Locus_539_Transcript_1/3_Confidence_0.714_Length_5649|.|sp|P98163|
YL_DROME Putative vitellogenin receptor OS=Drosophila melanogaster
GN=yl PE=1 SV=2|e=0|from=436|to=5394|frame=-1|Fragment_6
DGFRCRNGHCIS
>Locus_539_Transcript_1/3_Confidence_0.714_Length_5649|.|sp|P98163|
YL_DROME Putative vitellogenin receptor OS=Drosophila melanogaster
GN=yl PE=1 SV=2|e=0|from=436|to=5394|frame=-1|Fragment_7
EDWVCDGAIDCADGSDEIDCDSRPVTFMCILEKDHYPCRDNSSCIPINKVCDNIPHCKDK
SDEGVGCQQ
>Locus_539_Transcript_1/3_Confidence_0.714_Length_5649|.|sp|P98163|
YL_DROME Putative vitellogenin receptor OS=Drosophila melanogaster
GN=yl PE=1 SV=2|e=0|from=436|to=5394|frame=-1|Fragment_8
DCTSLSCA
>Locus_539_Transcript_1/3_Confidence_0.714_Length_5649|.|sp|P98163|
YL_DROME Putative vitellogenin receptor OS=Drosophila melanogaster
GN=yl PE=1 SV=2|e=0|from=436|to=5394|frame=-1|Fragment_9
KHCVKTP
>Locus_539_Transcript_1/3_Confidence_0.714_Length_5649|.|sp|P98163|
YL_DROME Putative vitellogenin receptor OS=Drosophila melanogaster
GN=yl PE=1 SV=2|e=0|from=436|to=5394|frame=-1|Fragment_10
EGPRCVCPSGFHNPLNSDFSHMHKNWQYNNNDNCTDINECAIHG
>Locus_539_Transcript_1/3_Confidence_0.714_Length_5649|.|sp|P98163|
YL_DROME Putative vitellogenin receptor OS=Drosophila melanogaster

GN=yl PE=1 SV=2|e=0|from=436|to=5394|frame=-1|Fragment_11
MCSQRCENTIGSYKCLCDMKYTLSSDKKSCK
>Locus_539_Transcript_1/3_Confidence_0.714_Length_5649|.|sp|P98163|
YL_DROME Putative vitellogenin receptor OS=Drosophila melanogaster
GN=yl PE=1 SV=2|e=0|from=436|to=5394|frame=-1|Fragment_12
VNGG
>Locus_539_Transcript_1/3_Confidence_0.714_Length_5649|.|sp|P98163|
YL_DROME Putative vitellogenin receptor OS=Drosophila melanogaster
GN=yl PE=1 SV=2|e=0|from=436|to=5394|frame=-1|Fragment_13
EALLLFSSRSNIRGLYLES
>Locus_539_Transcript_1/3_Confidence_0.714_Length_5649|.|sp|P98163|
YL_DROME Putative vitellogenin receptor OS=Drosophila melanogaster
GN=yl PE=1 SV=2|e=0|from=436|to=5394|frame=-1|Fragment_14
KILFQVIKNQRQIIGVEYDGNHVYWTDVHLGTESILRSLEDGSQMEVLLTSGLSAPEDLA
LDWLTGNIYFTDAELMHIGVCSNDGKHCTIIVNENIHKPRAIALYPGRGQMFWSDWGDNP
QIATSSMDGSSPKLFVSTNLHWPNGISIDIPNKRLYWVDAKLQTIESINLDGTDRRIVLK
GVTKHPYAIAIFEDQIYWSDWDSKSIQSCNKFTCKNRVTVAKESKEYIYGIHIYHSALQP
KHDHPCMKSSCSHLCLIKSAH
>Locus_539_Transcript_1/3_Confidence_0.714_Length_5649|.|sp|P98163|
YL_DROME Putative vitellogenin receptor OS=Drosophila melanogaster
GN=yl PE=1 SV=2|e=0|from=436|to=5394|frame=-1|Fragment_15
EYSCACPEGKELSADKTSCRETQRQSELIIGAGNLMVSLMHQKLGRHEL
>Locus_539_Transcript_1/3_Confidence_0.714_Length_5649|.|sp|P98163|
YL_DROME Putative vitellogenin receptor OS=Drosophila melanogaster
GN=yl PE=1 SV=2|e=0|from=436|to=5394|frame=-1|Fragment_16
HGVPMKINMDVLTYNSLNGEIIVADNNKKFIYTVDMYTTKSSRLTDIKSGEVEAMHFDNL
ANNLFWIDSVQQTIEVLHLDTGVKGFILHGLGDQVPVGFAIVPSHGIMFVAFRSHDHTHI
DRMLLDGSGIRTHVVEDGLGRWGKISMVYDHDLQRVFWAVEETGRIESSSVDGDDRHGFR
HTKDKPIAIAILGNDLFWTTAGNRRLYWADKHNLGNGFNKKLTIDIER
>Locus_539_Transcript_1/3_Confidence_0.714_Length_5649|.|sp|P98163|
YL_DROME Putative vitellogenin receptor OS=Drosophila melanogaster
GN=yl PE=1 SV=2|e=0|from=436|to=5394|frame=-1|Fragment_17
NLKSVPLTAITPRTISNHLCIHENGGCSHICIPMMQGKISCHCPVGLSLVPPYNMSCETI
IHCTGEQFRCSDTNKCIPMIFRCNGNKDCDFGEDESDCE
>Locus_539_Transcript_1/3_Confidence_0.714_Length_5649|.|sp|P98163|
YL_DROME Putative vitellogenin receptor OS=Drosophila melanogaster
GN=yl PE=1 SV=2|e=0|from=436|to=5394|frame=-1|Fragment_18
RYKDRQSCAVNEFQCNDG
>Locus_539_Transcript_1/3_Confidence_0.714_Length_5649|.|sp|P98163|
YL_DROME Putative vitellogenin receptor OS=Drosophila melanogaster
GN=yl PE=1 SV=2|e=0|from=436|to=5394|frame=-1|Fragment_19
ICIEYHYKCDKHFDCPDKSDEENCENYVSSSKCTHAQFECANGQCIDNRLLCDVLPDCLD
GSDEKMCDHVT
>Locus_539_Transcript_1/3_Confidence_0.714_Length_5649|.|sp|P98163|
YL_DROME Putative vitellogenin receptor OS=Drosophila melanogaster
GN=yl PE=1 SV=2|e=0|from=436|to=5394|frame=-1|Fragment_20
CESNMFQCQSGSCIPSSWECDGEIDCTDASDEHTFCEPNTCKDNQFTCTNGKCIDMDLKC
DHEDDCGDSSDEIRCPSKEKESTTETDFIDLITCEPTQYTCEES
>Locus_539_Transcript_1/3_Confidence_0.714_Length_5649|.|sp|P98163|
YL_DROME Putative vitellogenin receptor OS=Drosophila melanogaster
GN=yl PE=1 SV=2|e=0|from=436|to=5394|frame=-1|Fragment_21
GACLPEIARCNGTAECKYGEDERECTN
>Locus_539_Transcript_1/3_Confidence_0.714_Length_5649|.|sp|P98163|
YL_DROME Putative vitellogenin receptor OS=Drosophila melanogaster

GN=yl PE=1 SV=2|e=0|from=436|to=5394|frame=-1|Fragment_22
CQLDEFTCNSG
>Locus_539_Transcript_1/3_Confidence_0.714_Length_5649|.|sp|P98163|
YL_DROME Putative vitellogenin receptor OS=Drosophila melanogaster
GN=yl PE=1 SV=2|e=0|from=436|to=5394|frame=-1|Fragment_23
QCIQRKWVCDKTRDCNDGSDETITTVQPVH
>Locus_539_Transcript_1/3_Confidence_0.714_Length_5649|.|sp|P98163|
YL_DROME Putative vitellogenin receptor OS=Drosophila melanogaster
GN=yl PE=1 SV=2|e=0|from=436|to=5394|frame=-1|Fragment_24
SQFLP
>Locus_539_Transcript_1/3_Confidence_0.714_Length_5649|.|sp|P98163|
YL_DROME Putative vitellogenin receptor OS=Drosophila melanogaster
GN=yl PE=1 SV=2|e=0|from=436|to=5394|frame=-1|Fragment_25
VCDEDSFRCKSGGCVDFLFVCNGIPDCFDGSDENGACNTSCDAIK
>Locus_539_Transcript_1/3_Confidence_0.714_Length_5649|.|sp|P98163|
YL_DROME Putative vitellogenin receptor OS=Drosophila melanogaster
GN=yl PE=1 SV=2|e=0|from=436|to=5394|frame=-1|Fragment_26
CKHKCSPTPNGPQCSCHEGYHLAGDHLSCTDINECKAELQQCGQICTNSAGSFHCSCYPE
FMLRSDKRSCKATGDQMSILYSTSNQIRELTLSPPSVKILFNRNIPKILSLDVSKHTSTL
YFSLHLISQIYRLDIKSGDVQYIMNIGKPMKLAVDWITQNIYFISEDDLHKTLRVCHIEN
AACAILMKFDKNIAVPELVVDPISKYIFYTVTHWWVYDNPITILYRANLDGSHVIELRNE
KLNLISGIGIDHHQKTVYYCDKHKNTIESVSY
>Locus_539_Transcript_1/3_Confidence_0.714_Length_5649|.|sp|P98163|
YL_DROME Putative vitellogenin receptor OS=Drosophila melanogaster
GN=yl PE=1 SV=2|e=0|from=436|to=5394|frame=-1|Fragment_27
NGSDHR
>Locus_539_Transcript_1/3_Confidence_0.714_Length_5649|.|sp|P98163|
YL_DROME Putative vitellogenin receptor OS=Drosophila melanogaster
GN=yl PE=1 SV=2|e=0|from=436|to=5394|frame=-1|Fragment_28
ILVKEQKLLTAPNGLDVFENYAYTLVYGSTRMIKCRLFGNYACTVIDAYISNADSFKISQ
YSKQ
>Locus_539_Transcript_1/3_Confidence_0.714_Length_5649|.|sp|P98163|
YL_DROME Putative vitellogenin receptor OS=Drosophila melanogaster
GN=yl PE=1 SV=2|e=0|from=436|to=5394|frame=-1|Fragment_29
LLHENACENSNCMYLCVPADSGPKCICKNGLMIAPGSSC
>Locus_539_Transcript_1/3_Confidence_0.714_Length_5649|.|sp|P98163|
YL_DROME Putative vitellogenin receptor OS=Drosophila melanogaster
GN=yl PE=1 SV=2|e=1.94419E-67|from=3487|to=5400|frame=-1|Fragment_1
KECLTSEYTCGDGECISKTRICDGIKDCKDDSDEHHCEMYRCEEPDWYRCADGECISSSF
RCDKSMDCPSGSDEQNCEDFVYTPP
>Locus_539_Transcript_1/3_Confidence_0.714_Length_5649|.|sp|P98163|
YL_DROME Putative vitellogenin receptor OS=Drosophila melanogaster
GN=yl PE=1 SV=2|e=1.94419E-67|from=3487|to=5400|frame=-1|Fragment_2
STNHSCEHNEWMCAD
>Locus_539_Transcript_1/3_Confidence_0.714_Length_5649|.|sp|P98163|
YL_DROME Putative vitellogenin receptor OS=Drosophila melanogaster
GN=yl PE=1 SV=2|e=1.94419E-67|from=3487|to=5400|frame=-1|Fragment_3
RMCIPLEFVCNGDFDCMDKSDEDMGCKDQHACDGFRCRNG
>Locus_539_Transcript_1/3_Confidence_0.714_Length_5649|.|sp|P98163|
YL_DROME Putative vitellogenin receptor OS=Drosophila melanogaster
GN=yl PE=1 SV=2|e=1.94419E-67|from=3487|to=5400|frame=-1|Fragment_4
HCISEDWVCDGAIDCADGSDEIDCDSRPVTFMCILEKDHY
>Locus_539_Transcript_1/3_Confidence_0.714_Length_5649|.|sp|P98163|
YL_DROME Putative vitellogenin receptor OS=Drosophila melanogaster

GN=yl PE=1 SV=2|e=1.94419E-67|from=3487|to=5400|frame=-1|Fragment_5
PCR
>Locus_539_Transcript_1/3_Confidence_0.714_Length_5649|.|sp|P98163|
YL_DROME Putative vitellogenin receptor OS=Drosophila melanogaster
GN=yl PE=1 SV=2|e=1.94419E-67|from=3487|to=5400|frame=-1|Fragment_6
DNSSCIPINKVCDNIPHCKDKSDEGVGCQQDCTSLS
>Locus_539_Transcript_1/3_Confidence_0.714_Length_5649|.|sp|P98163|
YL_DROME Putative vitellogenin receptor OS=Drosophila melanogaster
GN=yl PE=1 SV=2|e=1.94419E-67|from=3487|to=5400|frame=-1|Fragment_7
CAKHCVKTPEGPRCVCPSGFHNPLNSDFSHMHKNWQYNNNDNCTDINECAIHGMCSQRCE
NTIGSYKCLCDMKYTLSSDKKSCKVNGGEALLLFSSRSNIRGLYLESKILFQVI
>Locus_539_Transcript_1/3_Confidence_0.714_Length_5649|.|sp|P98163|
YL_DROME Putative vitellogenin receptor OS=Drosophila melanogaster
GN=yl PE=1 SV=2|e=1.94419E-67|from=3487|to=5400|frame=-1|Fragment_8
KNQRQIIGVEY
>Locus_539_Transcript_1/3_Confidence_0.714_Length_5649|.|sp|P98163|
YL_DROME Putative vitellogenin receptor OS=Drosophila melanogaster
GN=yl PE=1 SV=2|e=1.94419E-67|from=3487|to=5400|frame=-1|Fragment_9
DGNHVYWTDVHLGTESILRSLEDGSQMEVLLTSGLSAPEDLALDWLTGNIYFTDAELMHI
GVCSNDGKHCTIIVNENIHKPRAI
>Locus_539_Transcript_1/3_Confidence_0.714_Length_5649|.|sp|P98163|
YL_DROME Putative vitellogenin receptor OS=Drosophila melanogaster
GN=yl PE=1 SV=2|e=1.94419E-67|from=3487|to=5400|frame=-1|Fragment_10
ALYPGRGQMFW
>Locus_539_Transcript_1/3_Confidence_0.714_Length_5649|.|sp|P98163|
YL_DROME Putative vitellogenin receptor OS=Drosophila melanogaster
GN=yl PE=1 SV=2|e=1.94419E-67|from=3487|to=5400|frame=-1|Fragment_11
SDWGD
>Locus_539_Transcript_1/3_Confidence_0.714_Length_5649|.|sp|P98163|
YL_DROME Putative vitellogenin receptor OS=Drosophila melanogaster
GN=yl PE=1 SV=2|e=1.94419E-67|from=3487|to=5400|frame=-1|Fragment_12
NPQIATSSMDGSSPKLFVSTNLHWPNGISIDIPNKRLYWVDAKLQTIESINL
>Locus_539_Transcript_1/3_Confidence_0.714_Length_5649|.|sp|P98163|
YL_DROME Putative vitellogenin receptor OS=Drosophila melanogaster
GN=yl PE=1 SV=2|e=1.94419E-67|from=3487|to=5400|frame=-1|Fragment_13
DGTDRR
>Locus_539_Transcript_1/3_Confidence_0.714_Length_5649|.|sp|P98163|
YL_DROME Putative vitellogenin receptor OS=Drosophila melanogaster
GN=yl PE=1 SV=2|e=1.94419E-67|from=3487|to=5400|frame=-1|Fragment_14
IVLK
>Locus_539_Transcript_1/3_Confidence_0.714_Length_5649|.|sp|P98163|
YL_DROME Putative vitellogenin receptor OS=Drosophila melanogaster
GN=yl PE=1 SV=2|e=1.94419E-67|from=3487|to=5400|frame=-1|Fragment_15
GVTKHPYAIAIFEDQIYWSDWDSKSIQSC
>Locus_539_Transcript_1/3_Confidence_0.714_Length_5649|.|sp|P98163|
YL_DROME Putative vitellogenin receptor OS=Drosophila melanogaster
GN=yl PE=1 SV=2|e=1.94419E-67|from=3487|to=5400|frame=-1|Fragment_16
NKFTCKNRVTVAKESKEYIYGIHIYHSALQPKHDHPCMKSSCSHLCLIKSAHEYSCACPE
GKELSADKTSCRETQRQSELIIGAGNLMVSLMHQKLGRHELHG
>Locus_539_Transcript_1/3_Confidence_0.714_Length_5649|.|sp|P98163|
YL_DROME Putative vitellogenin receptor OS=Drosophila melanogaster
GN=yl PE=1 SV=2|e=1.10398E-62|from=316|to=2373|frame=-1|Fragment_1
SSSKCTHAQFECANGQCIDNRLLCDVLPDCLDGSDEKMCDHVTCE
>Locus_539_Transcript_1/3_Confidence_0.714_Length_5649|.|sp|P98163|

YL_DROME Putative vitellogenin receptor OS=Drosophila melanogaster
GN=yl PE=1 SV=2|e=1.10398E-62|from=316|to=2373|frame=-1|Fragment_2
SNMFQCQS
>Locus_539_Transcript_1/3_Confidence_0.714_Length_5649|.|sp|P98163|
YL_DROME Putative vitellogenin receptor OS=Drosophila melanogaster
GN=yl PE=1 SV=2|e=1.10398E-62|from=316|to=2373|frame=-1|Fragment_3
GSCIPSSWECDGEIDCTDASDE
>Locus_539_Transcript_1/3_Confidence_0.714_Length_5649|.|sp|P98163|
YL_DROME Putative vitellogenin receptor OS=Drosophila melanogaster
GN=yl PE=1 SV=2|e=1.10398E-62|from=316|to=2373|frame=-1|Fragment_4
HTFCEPNTCKDNQFTCTNGK
>Locus_539_Transcript_1/3_Confidence_0.714_Length_5649|.|sp|P98163|
YL_DROME Putative vitellogenin receptor OS=Drosophila melanogaster
GN=yl PE=1 SV=2|e=1.10398E-62|from=316|to=2373|frame=-1|Fragment_5
CIDMDLKCDHEDDCGDSSDEIR
>Locus_539_Transcript_1/3_Confidence_0.714_Length_5649|.|sp|P98163|
YL_DROME Putative vitellogenin receptor OS=Drosophila melanogaster
GN=yl PE=1 SV=2|e=1.10398E-62|from=316|to=2373|frame=-1|Fragment_6
CPSKEKESTTETDFIDLITCEPTQYTCEESGACLPEIA
>Locus_539_Transcript_1/3_Confidence_0.714_Length_5649|.|sp|P98163|
YL_DROME Putative vitellogenin receptor OS=Drosophila melanogaster
GN=yl PE=1 SV=2|e=1.10398E-62|from=316|to=2373|frame=-1|Fragment_7
RCNGTAECKYGEDERECTNC
>Locus_539_Transcript_1/3_Confidence_0.714_Length_5649|.|sp|P98163|
YL_DROME Putative vitellogenin receptor OS=Drosophila melanogaster
GN=yl PE=1 SV=2|e=1.10398E-62|from=316|to=2373|frame=-1|Fragment_8
QLDEFTC
>Locus_539_Transcript_1/3_Confidence_0.714_Length_5649|.|sp|P98163|
YL_DROME Putative vitellogenin receptor OS=Drosophila melanogaster
GN=yl PE=1 SV=2|e=1.10398E-62|from=316|to=2373|frame=-1|Fragment_9
NSGQCIQRKWVCDKTRDCNDGSDETITTVQPVHSQFLPVCDEDSFRCKSGGCVDFLFVCN
GIPDCFDGSDENGACNTSCDAIKCK
>Locus_539_Transcript_1/3_Confidence_0.714_Length_5649|.|sp|P98163|
YL_DROME Putative vitellogenin receptor OS=Drosophila melanogaster
GN=yl PE=1 SV=2|e=1.10398E-62|from=316|to=2373|frame=-1|Fragment_10
HKCSPTP
>Locus_539_Transcript_1/3_Confidence_0.714_Length_5649|.|sp|P98163|
YL_DROME Putative vitellogenin receptor OS=Drosophila melanogaster
GN=yl PE=1 SV=2|e=1.10398E-62|from=316|to=2373|frame=-1|Fragment_11
NGPQCSCHEGYHLAGDHLSCTDINECKAELQQCGQICTNSAGSFHCSCYPEFMLRSDKRS
CKAT
>Locus_539_Transcript_1/3_Confidence_0.714_Length_5649|.|sp|P98163|
YL_DROME Putative vitellogenin receptor OS=Drosophila melanogaster
GN=yl PE=1 SV=2|e=1.10398E-62|from=316|to=2373|frame=-1|Fragment_12
GDQMSILYSTSNQIRELTLSPPSVKILFNRNIPKILSLDVSKHTSTLYFSLHL
>Locus_539_Transcript_1/3_Confidence_0.714_Length_5649|.|sp|P98163|
YL_DROME Putative vitellogenin receptor OS=Drosophila melanogaster
GN=yl PE=1 SV=2|e=1.10398E-62|from=316|to=2373|frame=-1|Fragment_13
ISQIYRLDIKSGDVQYIMNIG
>Locus_539_Transcript_1/3_Confidence_0.714_Length_5649|.|sp|P98163|
YL_DROME Putative vitellogenin receptor OS=Drosophila melanogaster
GN=yl PE=1 SV=2|e=1.10398E-62|from=316|to=2373|frame=-1|Fragment_14
KPMKLAVDWITQNIYFISEDDLHKTLRVCHIENAACAILMKFDKNIAVPELVVDPISKYI
FYTVTHWWVYDNPITILYRANLDGSHVIELRNEKLNLISGIGIDHHQKTVYYCDKHKNTI

ESVSYNGSDHRILVKEQKLLTAPNGLDVFENYAYTLVYGSTRMIKCRLFGNYACTVIDAY
ISNADSFKISQYSKQLLHENACENSNCMYLCV
>Locus_539_Transcript_1/3_Confidence_0.714_Length_5649|.|sp|P98163|
YL_DROME Putative vitellogenin receptor OS=Drosophila melanogaster
GN=yl PE=1 SV=2|e=1.10398E-62|from=316|to=2373|frame=-1|Fragment_15
PADSGPKCICKNGLMIAPGSSCVDDMD
>Locus_539_Transcript_1/3_Confidence_0.714_Length_5649|.|sp|P98163|
YL_DROME Putative vitellogenin receptor OS=Drosophila melanogaster
GN=yl PE=1 SV=2|e=1.10398E-62|from=316|to=2373|frame=-1|Fragment_16
DHPQFGTMASNEQSTSVAVPIFLSLLLFGAVAGSI
>Locus_539_Transcript_1/3_Confidence_0.714_Length_5649|.|sp|P98163|
YL_DROME Putative vitellogenin receptor OS=Drosophila melanogaster
GN=yl PE=1 SV=2|e=3.13288E-33|from=4690|to=5433|frame=-1|Fragment_1
VCPRSGFVFDHK
>Locus_539_Transcript_1/3_Confidence_0.714_Length_5649|.|sp|P98163|
YL_DROME Putative vitellogenin receptor OS=Drosophila melanogaster
GN=yl PE=1 SV=2|e=3.13288E-33|from=4690|to=5433|frame=-1|Fragment_2
ECLTSEYTCGDGECISKTRICDGIKDCKDDSDEHHC
>Locus_539_Transcript_1/3_Confidence_0.714_Length_5649|.|sp|P98163|
YL_DROME Putative vitellogenin receptor OS=Drosophila melanogaster
GN=yl PE=1 SV=2|e=3.13288E-33|from=4690|to=5433|frame=-1|Fragment_3
EMYRCE
>Locus_539_Transcript_1/3_Confidence_0.714_Length_5649|.|sp|P98163|
YL_DROME Putative vitellogenin receptor OS=Drosophila melanogaster
GN=yl PE=1 SV=2|e=3.13288E-33|from=4690|to=5433|frame=-1|Fragment_4
EPDWYRCADGE
>Locus_539_Transcript_1/3_Confidence_0.714_Length_5649|.|sp|P98163|
YL_DROME Putative vitellogenin receptor OS=Drosophila melanogaster
GN=yl PE=1 SV=2|e=3.13288E-33|from=4690|to=5433|frame=-1|Fragment_5
CISSSFRCDKSMDCPSGSDEQNCEDFVYTPPSTNHSCEHNEWMCADRMCIPLEFVCNGDF
DCMDKSDEDMGCKDQHACDG
>Locus_539_Transcript_1/3_Confidence_0.714_Length_5649|.|sp|P98163|
YL_DROME Putative vitellogenin receptor OS=Drosophila melanogaster
GN=yl PE=1 SV=2|e=3.13288E-33|from=4690|to=5433|frame=-1|Fragment_6
FRCRNGHCISEDWVCDGAIDCADGSDEID
>Locus_539_Transcript_1/3_Confidence_0.714_Length_5649|.|sp|P98163|
YL_DROME Putative vitellogenin receptor OS=Drosophila melanogaster
GN=yl PE=1 SV=2|e=3.13288E-33|from=4690|to=5433|frame=-1|Fragment_7
CDSR
>Locus_539_Transcript_1/3_Confidence_0.714_Length_5649|.|sp|P98163|
YL_DROME Putative vitellogenin receptor OS=Drosophila melanogaster
GN=yl PE=1 SV=2|e=3.13288E-33|from=4690|to=5433|frame=-1|Fragment_8
PVTFMCILEKDHYPCRDNSSCIPINKVCDNIPHCKDKSDE
>Locus_539_Transcript_1/3_Confidence_0.714_Length_5649|.|sp|P98163|
YL_DROME Putative vitellogenin receptor OS=Drosophila melanogaster
GN=yl PE=1 SV=2|e=3.13288E-33|from=4690|to=5433|frame=-1|Fragment_9
GVGCQQDCTSLSCAKHCVKTPEGPRCVCPS
>Locus_539_Transcript_1/3_Confidence_0.714_Length_5649|.|sp|P98163|
YL_DROME Putative vitellogenin receptor OS=Drosophila melanogaster
GN=yl PE=1 SV=2|e=1.66892E-18|from=3811|to=5154|frame=-1|Fragment_1
TPPSTNHSCEHNEWMCADRMCIPLEFVCNGDFDCMDKSDEDMGCKDQHAC
>Locus_539_Transcript_1/3_Confidence_0.714_Length_5649|.|sp|P98163|
YL_DROME Putative vitellogenin receptor OS=Drosophila melanogaster
GN=yl PE=1 SV=2|e=1.66892E-18|from=3811|to=5154|frame=-1|Fragment_2

DGFRCRNGH
>Locus_539_Transcript_1/3_Confidence_0.714_Length_5649|.|sp|P98163|
YL_DROME Putative vitellogenin receptor OS=Drosophila melanogaster
GN=yl PE=1 SV=2|e=1.66892E-18|from=3811|to=5154|frame=-1|Fragment_3
CISEDWVCDGAIDCADGSDEIDCDSRP
>Locus_539_Transcript_1/3_Confidence_0.714_Length_5649|.|sp|P98163|
YL_DROME Putative vitellogenin receptor OS=Drosophila melanogaster
GN=yl PE=1 SV=2|e=1.66892E-18|from=3811|to=5154|frame=-1|Fragment_4
VTFMCILEKDHYPCRDNSSCIPINKVCDNIPHCKDKSDEGVGCQQD
>Locus_539_Transcript_1/3_Confidence_0.714_Length_5649|.|sp|P98163|
YL_DROME Putative vitellogenin receptor OS=Drosophila melanogaster
GN=yl PE=1 SV=2|e=1.66892E-18|from=3811|to=5154|frame=-1|Fragment_5
CTSLSCAKHCVKTPEG
>Locus_539_Transcript_1/3_Confidence_0.714_Length_5649|.|sp|P98163|
YL_DROME Putative vitellogenin receptor OS=Drosophila melanogaster
GN=yl PE=1 SV=2|e=1.66892E-18|from=3811|to=5154|frame=-1|Fragment_6
PRCVCPSGFHNPLNSDFSHMHKNWQYNNNDNCTDINE
>Locus_539_Transcript_1/3_Confidence_0.714_Length_5649|.|sp|P98163|
YL_DROME Putative vitellogenin receptor OS=Drosophila melanogaster
GN=yl PE=1 SV=2|e=1.66892E-18|from=3811|to=5154|frame=-1|Fragment_7
CAIHGMCSQRCENT
>Locus_539_Transcript_1/3_Confidence_0.714_Length_5649|.|sp|P98163|
YL_DROME Putative vitellogenin receptor OS=Drosophila melanogaster
GN=yl PE=1 SV=2|e=1.66892E-18|from=3811|to=5154|frame=-1|Fragment_8
IGSYKCLCDMK
>Locus_539_Transcript_1/3_Confidence_0.714_Length_5649|.|sp|P98163|
YL_DROME Putative vitellogenin receptor OS=Drosophila melanogaster
GN=yl PE=1 SV=2|e=1.66892E-18|from=3811|to=5154|frame=-1|Fragment_9
YTLSSDKKSCKVNGGEALLLFSSR
>Locus_539_Transcript_1/3_Confidence_0.714_Length_5649|.|sp|P98163|
YL_DROME Putative vitellogenin receptor OS=Drosophila melanogaster
GN=yl PE=1 SV=2|e=1.66892E-18|from=3811|to=5154|frame=-1|Fragment_10
SNIRGLYLESKILFQVIKNQRQIIGVEYDGNHVYWTDVHLGTESILRSLEDGSQMEVLLT
SGLSAPEDLALDWLTGNIYFTDAELMHIGVCSNDGKHC
>Locus_539_Transcript_1/3_Confidence_0.714_Length_5649|.|sp|P98163|
YL_DROME Putative vitellogenin receptor OS=Drosophila melanogaster
GN=yl PE=1 SV=2|e=1.66892E-18|from=3811|to=5154|frame=-1|Fragment_11
TIIVNENIHKPRAIALYPGRGQMFWSDWGDNPQIATSSMDGSSPKLFVSTNLHWPNGISI
DIPNKRLYWVDAKLQTIESINLDGTDRRIVLKGVTKHPYAIAIFEDQ
>Locus_539_Transcript_1/3_Confidence_0.714_Length_5649|.|sp|P98163|
YL_DROME Putative vitellogenin receptor OS=Drosophila melanogaster
GN=yl PE=1 SV=2|e=1.66892E-18|from=3811|to=5154|frame=-1|Fragment_12
IYWSDWDSK

>Locus_26707_Transcript_1/1_Confidence_1.000_Length_258|UNIGENE
REPRESENTATIVE TRANSCRIPT|sp|Q04164|SAS_DROME Putative epidermal
cell surface receptor OS=Drosophila melanogaster GN=sas PE=2 SV=2|
e=4.04109E-29|from=7|to=258|frame=-1|Fragment_1
IPFAGQNIELKAENMLRVKTEREIDVYGFEIKINATKIKSTTMEISWSGVPYPEDKFVNV
YRAIYQSETGKEDSSVFKVAKRES
>Locus_6199_Transcript_1/1_Confidence_1.000_Length_3685|UNIGENE
REPRESENTATIVE TRANSCRIPT|sp|Q05319|STUB_DROME Serine proteinase
stubble OS=Drosophila melanogaster GN=Sb PE=2 SV=2|e=3.5049E-155|
from=757|to=2418|frame=1|Fragment_1

EQHQRYKISPKPCSVNGHEGTCMFVYECIRSEGKHVGVCVDTFMFGSCCSHNLTDNAILP
>Locus_6199_Transcript_1/1_Confidence_1.000_Length_3685|UNIGENE REPRESENTATIVE TRANSCRIPT|sp|Q05319|STUB_DROME Serine proteinase stubble OS=Drosophila melanogaster GN=Sb PE=2 SV=2|e=3.5049E-155|from=757|to=2418|frame=1|Fragment_2
PATSLL
>Locus_9565_Transcript_2/2_Confidence_0.875_Length_1216|UNIGENE REPRESENTATIVE TRANSCRIPT|sp|Q17292|OPSD_CAMAT Rhodopsin OS=Camponotus atriceps PE=2 SV=1|e=5.0778E-149|from=9|to=1130|frame=-3|Fragment_1
GPEYSAYKWDRFNADPWANMTVVDKVPPEILHMVDVHWYQFPPMNPMWH
>Locus_9565_Transcript_2/2_Confidence_0.875_Length_1216|UNIGENE REPRESENTATIVE TRANSCRIPT|sp|Q17292|OPSD_CAMAT Rhodopsin OS=Camponotus atriceps PE=2 SV=1|e=5.0778E-149|from=9|to=1130|frame=-3|Fragment_2
GNGMVIYIFTSTKQLRTPSNLLVINLAVSDFLMMFWMSPPMVINCYYETWILGPLMCELY
GMFGSLCGCVSIWTMTMIAFDRYNVIVKGLAAKPLTNKGAVLRILLVWISSGLWGIAPFF
GWNRYVPEGNMTACGTDYLNKDWFSRSYILVYSVFVYYLPLFCIIYSYFFIVRAVAEHEK
SMREQAKKMKV
>Locus_9565_Transcript_2/2_Confidence_0.875_Length_1216|UNIGENE REPRESENTATIVE TRANSCRIPT|sp|Q17292|OPSD_CAMAT Rhodopsin OS=Camponotus atriceps PE=2 SV=1|e=5.0778E-149|from=9|to=1130|frame=-3|Fragment_3
AEFKLAKVALMTIALWFFAWTPYLVINYTGIFETFTISPLATIWGSLFAKANAVYNPIVY
GISHPKYRAALFKKFPSLMCAPEPQPEVDLQSLASGVTAVSE
>Locus_10514_Transcript_1/3_Confidence_0.750_Length_2925|UNIGENE REPRESENTATIVE TRANSCRIPT|sp|Q24025|SOG_DROME Dorsal-ventral patterning protein Sog OS=Drosophila melanogaster GN=sog PE=1 SV=1|e=4.6474E-155|from=3|to=1784|frame=3|Fragment_1
IILDEVRRVRKPGHELNVIEVSSPVSAADMRLLTRGRLALTVESRRRPEALRLQGPVVTR
ATCEIFQ
>Locus_10514_Transcript_1/3_Confidence_0.750_Length_2925|UNIGENE REPRESENTATIVE TRANSCRIPT|sp|Q24025|SOG_DROME Dorsal-ventral patterning protein Sog OS=Drosophila melanogaster GN=sog PE=1 SV=1|e=4.6474E-155|from=3|to=1784|frame=3|Fragment_2
WLYLNRDGALVYNIQVDELDRTQTPMITLVDDSGKRRTELEDLTPSLTADGRATGTLERL
GPRVLEPLYAGNLAVNVATKTHQESLVRGRLVARPVADARDSPAGAILLKRPNLNTPAN
>Locus_10514_Transcript_1/3_Confidence_0.750_Length_2925|UNIGENE REPRESENTATIVE TRANSCRIPT|sp|Q24025|SOG_DROME Dorsal-ventral patterning protein Sog OS=Drosophila melanogaster GN=sog PE=1 SV=1|e=4.6474E-155|from=3|to=1784|frame=3|Fragment_3
IVGVAWLAVDAADCSLHYDLSIAGRGSHQRTMELYLEEVPFIAPGAPVSRALLEEEFTTT
NVEGTLAGVTQGDLARLETGVVFLDLKDKIKNETMLKATLKHIKVPPNCLPHYTDNDVSA
MSPTGGASYDPESNSLQGHSPVVISVDPGQRCFHSGRFYVEGAQWRSAAESCTMCSCVHG
RVKCDAIKCPPLSKCRLEDRRI
>Locus_10514_Transcript_1/3_Confidence_0.750_Length_2925|UNIGENE REPRESENTATIVE TRANSCRIPT|sp|Q24025|SOG_DROME Dorsal-ventral patterning protein Sog OS=Drosophila melanogaster GN=sog PE=1 SV=1|e=4.6474E-155|from=3|to=1784|frame=3|Fragment_4
REGECCPVCTNNTVLPEPTNSSAP
>Locus_10514_Transcript_1/3_Confidence_0.750_Length_2925|UNIGENE REPRESENTATIVE TRANSCRIPT|sp|Q24025|SOG_DROME Dorsal-ventral patterning protein Sog OS=Drosophila melanogaster GN=sog PE=1 SV=1|e=4.6474E-155|from=3|to=1784|frame=3|Fragment_5

RGCYLGDQFHKAGSSWHPYLPPNGFDTCAVCTCDMITLDIRCPRTQCPPLRCDEKIAIRP
DKKACCKVCPEIKNEAV
>Locus_10514_Transcript_1/3_Confidence_0.750_Length_2925|UNIGENE
REPRESENTATIVE TRANSCRIPT|sp|Q24025|SOG_DROME Dorsal-ventral
patterning protein Sog OS=Drosophila melanogaster GN=sog PE=1 SV=1|
e=4.6474E-155|from=3|to=1784|frame=3|Fragment_6
PMVISDPGTVQDQGTIEI
>Locus_10514_Transcript_1/3_Confidence_0.750_Length_2925|UNIGENE
REPRESENTATIVE TRANSCRIPT|sp|Q24025|SOG_DROME Dorsal-ventral
patterning protein Sog OS=Drosophila melanogaster GN=sog PE=1 SV=1|
e=4.6474E-155|from=3|to=1784|frame=3|Fragment_7
ARTNEQIMQDGGCKNPLGAPYENGKEWHPWLSSHGEQKCVTCRCKDGAIKCERKRCPRMS
CANHRRAT
>Locus_10514_Transcript_2/3_Confidence_0.750_Length_2925|.|sp|
Q24025|SOG_DROME Dorsal-ventral patterning protein Sog OS=Drosophila
melanogaster GN=sog PE=1 SV=1|e=4.6474E-155|from=3|to=1784|frame=3|
Fragment_1
IILDEVRRVRKPGHELNVIEVSSPVSAADMRLLTRGRLALTVESRRRPEALRLQGPVVTR
ATCEIFQ
>Locus_10514_Transcript_2/3_Confidence_0.750_Length_2925|.|sp|
Q24025|SOG_DROME Dorsal-ventral patterning protein Sog OS=Drosophila
melanogaster GN=sog PE=1 SV=1|e=4.6474E-155|from=3|to=1784|frame=3|
Fragment_2
WLYLNRDGALVYNIQVDELDRTQTPMITLVDDSGKRRTELEDLTPSLTADGRATGTLERL
GPRVLEPLYAGNLAVNVATKTHQESLVRGRLVARPVADARDSPAGAILLKRPNLNTPAN
>Locus_10514_Transcript_2/3_Confidence_0.750_Length_2925|.|sp|
Q24025|SOG_DROME Dorsal-ventral patterning protein Sog OS=Drosophila
melanogaster GN=sog PE=1 SV=1|e=4.6474E-155|from=3|to=1784|frame=3|
Fragment_3
IVGVAWLAVDAADCSLHYDLSIAGRGSHQRTMELYLEEVPFIAPGAPVSRALLEEEFTTT
NVEGTLAGVTQGDLARLETGVVFLDLKDKIKNETMLKATLKHIKVPPNCLPHYTDNDVSA
MSPTGGASYDPESNSLQGHSPVVISVDPGQRCFHSGRFYVEGAQWRSAAESCTMCSCVHG
RVKCDAIKCPPLSKCRLEDRRI
>Locus_10514_Transcript_2/3_Confidence_0.750_Length_2925|.|sp|
Q24025|SOG_DROME Dorsal-ventral patterning protein Sog OS=Drosophila
melanogaster GN=sog PE=1 SV=1|e=4.6474E-155|from=3|to=1784|frame=3|
Fragment_4
REGECCPVCTNNTVLPEPTNSSAP
>Locus_10514_Transcript_2/3_Confidence_0.750_Length_2925|.|sp|
Q24025|SOG_DROME Dorsal-ventral patterning protein Sog OS=Drosophila
melanogaster GN=sog PE=1 SV=1|e=4.6474E-155|from=3|to=1784|frame=3|
Fragment_5
RGCYLGDQFHKAGSSWHPYLPPNGFDTCAVCTCDMITLDIRCPRTQCPPLRCDEKIAIRP
DKKACCKVCPEIKNEAV
>Locus_10514_Transcript_2/3_Confidence_0.750_Length_2925|.|sp|
Q24025|SOG_DROME Dorsal-ventral patterning protein Sog OS=Drosophila
melanogaster GN=sog PE=1 SV=1|e=4.6474E-155|from=3|to=1784|frame=3|
Fragment_6
PMVISDPGTVQDQGTIEI
>Locus_10514_Transcript_2/3_Confidence_0.750_Length_2925|.|sp|
Q24025|SOG_DROME Dorsal-ventral patterning protein Sog OS=Drosophila
melanogaster GN=sog PE=1 SV=1|e=4.6474E-155|from=3|to=1784|frame=3|
Fragment_7
ARTNEQIMQDGGCKNPLGAPYENGKEWHPWLSSHGEQKCVTCRCKDGAIKCERKRCPRMS

CANHRRAT
>Locus_10514_Transcript_3/3_Confidence_0.750_Length_2925|.|sp|
Q24025|SOG_DROME Dorsal-ventral patterning protein Sog OS=Drosophila
melanogaster GN=sog PE=1 SV=1|e=4.6474E-155|from=3|to=1784|frame=3|
Fragment_1
IILDEVRRVRKPGHELNVIEVSSPVSAADMRLLTRGRLALTVESRRRPEALRLQGPVVTR
ATCEIFQ
>Locus_10514_Transcript_3/3_Confidence_0.750_Length_2925|.|sp|
Q24025|SOG_DROME Dorsal-ventral patterning protein Sog OS=Drosophila
melanogaster GN=sog PE=1 SV=1|e=4.6474E-155|from=3|to=1784|frame=3|
Fragment_2
WLYLNRDGALVYNIQVDELDRTQTPMITLVDDSGKRRTELEDLTPSLTADGRATGTLERL
GPRVLEPLYAGNLAVNVATKTHQESLVRGRLVARPVADARDSPAGAILLKRPNLNTPAN
>Locus_10514_Transcript_3/3_Confidence_0.750_Length_2925|.|sp|
Q24025|SOG_DROME Dorsal-ventral patterning protein Sog OS=Drosophila
melanogaster GN=sog PE=1 SV=1|e=4.6474E-155|from=3|to=1784|frame=3|
Fragment_3
IVGVAWLAVDAADCSLHYDLSIAGRGSHQRTMELYLEEVPFIAPGAPVSRALLEEEFTTT
NVEGTLAGVTQGDLARLETGVVFLDLKDKIKNETMLKATLKHIKVPPNCLPHYTDNDVSA
MSPTGGASYDPESNSLQGHSPVVISVDPGQRCFHSGRFYVEGAQWRSAAESCTMCSCVHG
RVKCDAIKCPPLSKCRLEDRRI
>Locus_10514_Transcript_3/3_Confidence_0.750_Length_2925|.|sp|
Q24025|SOG_DROME Dorsal-ventral patterning protein Sog OS=Drosophila
melanogaster GN=sog PE=1 SV=1|e=4.6474E-155|from=3|to=1784|frame=3|
Fragment_4
REGECCPVCTNNTVLPEPTNSSAP
>Locus_10514_Transcript_3/3_Confidence_0.750_Length_2925|.|sp|
Q24025|SOG_DROME Dorsal-ventral patterning protein Sog OS=Drosophila
melanogaster GN=sog PE=1 SV=1|e=4.6474E-155|from=3|to=1784|frame=3|
Fragment_5
RGCYLGDQFHKAGSSWHPYLPPNGFDTCAVCTCDMITLDIRCPRTQCPPLRCDEKIAIRP
DKKACCKVCPEIKNEAV
>Locus_10514_Transcript_3/3_Confidence_0.750_Length_2925|.|sp|
Q24025|SOG_DROME Dorsal-ventral patterning protein Sog OS=Drosophila
melanogaster GN=sog PE=1 SV=1|e=4.6474E-155|from=3|to=1784|frame=3|
Fragment_6
PMVISDPGTVQDQGTIEI
>Locus_10514_Transcript_3/3_Confidence_0.750_Length_2925|.|sp|
Q24025|SOG_DROME Dorsal-ventral patterning protein Sog OS=Drosophila
melanogaster GN=sog PE=1 SV=1|e=4.6474E-155|from=3|to=1784|frame=3|
Fragment_7
ARTNEQIMQDGGCKNPLGAPYENGKEWHPWLSSHGEQKCVTCRCKDGAIKCERKRCPRMS
CANHRRAT
>Locus_8703_Transcript_4/4_Confidence_0.556_Length_1512|.|sp|Q24025|
SOG_DROME Dorsal-ventral patterning protein Sog OS=Drosophila
melanogaster GN=sog PE=1 SV=1|e=8.5658E-120|from=404|to=1510|
frame=2|Fragment_1
QARRQSPLMVEE
>Locus_8703_Transcript_4/4_Confidence_0.556_Length_1512|.|sp|Q24025|
SOG_DROME Dorsal-ventral patterning protein Sog OS=Drosophila
melanogaster GN=sog PE=1 SV=1|e=8.5658E-120|from=404|to=1510|
frame=2|Fragment_2
DGFRKNHRAA
>Locus_8703_Transcript_4/4_Confidence_0.556_Length_1512|.|sp|Q24025|

SOG_DROME Dorsal-ventral patterning protein Sog OS=Drosophila
melanogaster GN=sog PE=1 SV=1|e=8.5658E-120|from=404|to=1510|
frame=2|Fragment_3
ECTFGKQTRELGSTWFADLGPPFGVMYCIRCECTPVQKKRRIVARVQCRNIKNECPKPTC
DEPVLHPGRCCKTCPGDIYSPDIPQDVSPTNTNEEEEKSMKHFAALLTGRSPLIHRREDL
SAIIDSHNPRNVVATGRFDFHRKNLHYSFYVSQPASRPRALQFLDKSGSILEEHSLVTH
>Locus_8703_Transcript_4/4_Confidence_0.556_Length_1512|.|sp|Q24025|
SOG_DROME Dorsal-ventral patterning protein Sog OS=Drosophila
melanogaster GN=sog PE=1 SV=1|e=8.5658E-120|from=404|to=1510|
frame=2|Fragment_4
GEYQNATGKVCGVWRRVPKEYRRLLKDEKLFVALLWNSDGVNHTGGELILSGRIGRYASL
GTELFSSLLE
>Locus_8703_Transcript_4/4_Confidence_0.556_Length_1512|.|sp|Q24025|
SOG_DROME Dorsal-ventral patterning protein Sog OS=Drosophila
melanogaster GN=sog PE=1 SV=1|e=8.5658E-120|from=404|to=1510|
frame=2|Fragment_5
PASGTDRERMSGAGGTAVVSTSVGATPSIHLTILFNGIFSPDDGNDVPITVRLELVERGL
IILDEVRRVRKPGHELNVIEVSSPVSAADMRLLTRGRL
>Locus_8703_Transcript_3/4_Confidence_0.444_Length_1474|.|sp|Q24025|
SOG_DROME Dorsal-ventral patterning protein Sog OS=Drosophila
melanogaster GN=sog PE=1 SV=1|e=2.4993E-116|from=404|to=1474|
frame=2|Fragment_1
QARRQSPLMVEE
>Locus_8703_Transcript_3/4_Confidence_0.444_Length_1474|.|sp|Q24025|
SOG_DROME Dorsal-ventral patterning protein Sog OS=Drosophila
melanogaster GN=sog PE=1 SV=1|e=2.4993E-116|from=404|to=1474|
frame=2|Fragment_2
DGFRKNHRAA
>Locus_8703_Transcript_3/4_Confidence_0.444_Length_1474|.|sp|Q24025|
SOG_DROME Dorsal-ventral patterning protein Sog OS=Drosophila
melanogaster GN=sog PE=1 SV=1|e=2.4993E-116|from=404|to=1474|
frame=2|Fragment_3
ECTFGKQTRELGSTWFADLGPPFGVMYCIRCECTPVQKKRRIVARVQCRNIKNECPKPTC
DEPVLHPGRCCKTCPGDIYSPDIPQDVSPTNTNEEEEKSMKHFAALLTGRSPLIHRREDL
SAIIDSHNPRNVVATGRFDFHRKNLHYSFYVSQPASRPRALQFLDKSGSILEEHSLVTH
>Locus_8703_Transcript_3/4_Confidence_0.444_Length_1474|.|sp|Q24025|
SOG_DROME Dorsal-ventral patterning protein Sog OS=Drosophila
melanogaster GN=sog PE=1 SV=1|e=2.4993E-116|from=404|to=1474|
frame=2|Fragment_4
GEYQNATGKVCGVWRRVPKEYRRLLKDEKLFVALLWNSDGVNHTGGELILSGRIGRYASL
GTELFSSLLE
>Locus_8703_Transcript_3/4_Confidence_0.444_Length_1474|.|sp|Q24025|
SOG_DROME Dorsal-ventral patterning protein Sog OS=Drosophila
melanogaster GN=sog PE=1 SV=1|e=2.4993E-116|from=404|to=1474|
frame=2|Fragment_5
PAAGTDRERMSGAGGTAVVSTSVGATPSIHLTILFNGIFSPDDGNDVPITVRLELVERGL
IILDEVRRVRKPGHELNVIEVSSPVS
>Locus_8703_Transcript_1/4_Confidence_0.444_Length_1453|.|sp|Q24025|
SOG_DROME Dorsal-ventral patterning protein Sog OS=Drosophila
melanogaster GN=sog PE=1 SV=1|e=4.3296E-113|from=404|to=1453|
frame=2|Fragment_1
QARRQSPLMVEE
>Locus_8703_Transcript_1/4_Confidence_0.444_Length_1453|.|sp|Q24025|
SOG_DROME Dorsal-ventral patterning protein Sog OS=Drosophila

melanogaster GN=sog PE=1 SV=1|e=4.3296E-113|from=404|to=1453|
frame=2|Fragment_2
DGFRKNHRAA
>Locus_8703_Transcript_1/4_Confidence_0.444_Length_1453|.|sp|Q24025|
SOG_DROME Dorsal-ventral patterning protein Sog OS=Drosophila
melanogaster GN=sog PE=1 SV=1|e=4.3296E-113|from=404|to=1453|
frame=2|Fragment_3
ECTFGKQTRELGSTWFADLGPPFGVMYCIRCECTPVQKKRRIVARVQCRNIKNECPKPTC
DEPVLHPGRCCKTCPGDIYSPDIPQDVSPTNTNEEEEKSMKHFAALLTGRSPLIHRREDL
SAIIDSHNPRNVVATGRFDFHRKNLHYSFYVSQPASRPRALQFLDKSGSILEEHSLVTH
>Locus_8703_Transcript_1/4_Confidence_0.444_Length_1453|.|sp|Q24025|
SOG_DROME Dorsal-ventral patterning protein Sog OS=Drosophila
melanogaster GN=sog PE=1 SV=1|e=4.3296E-113|from=404|to=1453|
frame=2|Fragment_4
GEYQNATGKVCGVWRRVPKEYRRLLKDEKLFVALLWNSDGVNHTGGELILSGRIGRYASL
GTELFSSLLE
>Locus_8703_Transcript_1/4_Confidence_0.444_Length_1453|.|sp|Q24025|
SOG_DROME Dorsal-ventral patterning protein Sog OS=Drosophila
melanogaster GN=sog PE=1 SV=1|e=4.3296E-113|from=404|to=1453|
frame=2|Fragment_5
PASGTDRERMSGAGGTAVVSTSVGATPSIHLTILFNGIFSPDDGNDVPITVRLELVERGL
IILDEVRRVRKPGHELNVI
>Locus_4007_Transcript_2/2_Confidence_0.667_Length_2248|UNIGENE
REPRESENTATIVE TRANSCRIPT|sp|Q24048|ATPB2_DROME Sodium/potassium-
transporting ATPase subunit beta-2 OS=Drosophila melanogaster
GN=nrv2 PE=1 SV=2|e=1.1971E-123|from=1113|to=2102|frame=-3|
Fragment_1
PTMNQKDQDKQLEFYQHYYKKPKKYDFTTWAGIKLFAYDKKTGEIFGRTGSSWSKIGIFY
TIFYGVLAALVAICMWVFFQTLDPRIPKWQLDESIIGTNPGLGFRPLPPDENIESTLIWY
KGTDLDNYSRWTKSLSEFLQFYKTPGLTYNRGQNIYSCDYNSPPPKGMVCDVDVKAWGPC
IEENNFNYHKSAPCVILKLNKIFGWVPEYYNDTNSLPEKMPEELKNYIRNDPEIVANPAM
RNTVWVSCEGENPADVEHIGTIKYYPKRGF
>Locus_4007_Transcript_2/2_Confidence_0.667_Length_2248|UNIGENE
REPRESENTATIVE TRANSCRIPT|sp|Q24048|ATPB2_DROME Sodium/potassium-
transporting ATPase subunit beta-2 OS=Drosophila melanogaster
GN=nrv2 PE=1 SV=2|e=1.1971E-123|from=1113|to=2102|frame=-3|
Fragment_2
YLSPLVAVHFERPKTGILINIECKAWAHNVIHDRHDRIGSVHFELMVD
>Locus_18836_Transcript_1/2_Confidence_0.750_Length_1142|UNIGENE
REPRESENTATIVE TRANSCRIPT|sp|Q24247|ITA1_DROME Integrin alpha-PS1
OS=Drosophila melanogaster GN=mew PE=1 SV=2|e=3.40138E-83|from=2|
to=1132|frame=-2|Fragment_1
EFKGDHVTSWLLAGAPLGQNLQTGSNRSGAFFKCPLTPNKDDCMQVMTDG
>Locus_18836_Transcript_1/2_Confidence_0.750_Length_1142|UNIGENE
REPRESENTATIVE TRANSCRIPT|sp|Q24247|ITA1_DROME Integrin alpha-PS1
OS=Drosophila melanogaster GN=mew PE=1 SV=2|e=3.40138E-83|from=2|
to=1132|frame=-2|Fragment_2
TTKSEDQIKTGQWLGVSVSS
>Locus_18836_Transcript_1/2_Confidence_0.750_Length_1142|UNIGENE
REPRESENTATIVE TRANSCRIPT|sp|Q24247|ITA1_DROME Integrin alpha-PS1
OS=Drosophila melanogaster GN=mew PE=1 SV=2|e=3.40138E-83|from=2|
to=1132|frame=-2|Fragment_3
ASTDFTGGQAAVCAHRYAEIINENVVVGKGLCFVFDQDLGVPEQFEPCKGKPHVRGHLQY
GLCQAGISTSFYNN

>Locus_18836_Transcript_1/2_Confidence_0.750_Length_1142|UNIGENE REPRESENTATIVE TRANSCRIPT|sp|Q24247|ITA1_DROME Integrin alpha-PS1 OS=Drosophila melanogaster GN=mew PE=1 SV=2|e=3.40138E-83|from=2|to=1132|frame=-2|Fragment_4
SLLIGAPGAYTWTGMVFVLKPTNDLFSE
>Locus_18836_Transcript_1/2_Confidence_0.750_Length_1142|UNIGENE REPRESENTATIVE TRANSCRIPT|sp|Q24247|ITA1_DROME Integrin alpha-PS1 OS=Drosophila melanogaster GN=mew PE=1 SV=2|e=3.40138E-83|from=2|to=1132|frame=-2|Fragment_5
TWETKVEQLGNEKE
>Locus_18836_Transcript_1/2_Confidence_0.750_Length_1142|UNIGENE REPRESENTATIVE TRANSCRIPT|sp|Q24247|ITA1_DROME Integrin alpha-PS1 OS=Drosophila melanogaster GN=mew PE=1 SV=2|e=3.40138E-83|from=2|to=1132|frame=-2|Fragment_6
YGYRGMSVVGGNFLNVPSIAIGAPRDNNYGSILIAVESNIPSRTLEVKMILYGEQFGSGF
GHEIIRADVNGDKRDDLIVGAPYYFEQNNGGAVYIYLNGEEGVTNTYFKRLLGPRPESRF
GIALACLGDLNKDGYEDIAVGAPEDGPGGAVYIFLGSKDGLQESPSQVIHAHT
>Locus_18836_Transcript_1/2_Confidence_0.750_Length_1142|UNIGENE REPRESENTATIVE TRANSCRIPT|sp|Q24247|ITA1_DROME Integrin alpha-PS1 OS=Drosophila melanogaster GN=mew PE=1 SV=2|e=3.40138E-83|from=2|to=1132|frame=-2|Fragment_7
GLL
>Locus_18836_Transcript_1/2_Confidence_0.750_Length_1142|UNIGENE REPRESENTATIVE TRANSCRIPT|sp|Q24247|ITA1_DROME Integrin alpha-PS1 OS=Drosophila melanogaster GN=mew PE=1 SV=2|e=3.40138E-83|from=2|to=1132|frame=-2|Fragment_8
KGIKTFGYSLSGGLD
>Locus_31460_Transcript_1/1_Confidence_1.000_Length_428|.|sp|Q24247|ITA1_DROME Integrin alpha-PS1 OS=Drosophila melanogaster GN=mew PE=1 SV=2|e=6.90854E-42|from=1|to=426|frame=1|Fragment_1
GTGQVVIFKKTKNVNPMEVIYVIPGEQFGSSFGYEIARADINGDGWDDMIVGAPFYFSKD
KGGAVYVFINNKGRFGENYSSRLLGPKYDSRFGFALAAVGDLNKDGCEDIAVGAPYEGSG
AVYIFFGSRQGIQEKPSQVIHA
>Locus_12799_Transcript_2/4_Confidence_0.667_Length_2171|UNIGENE REPRESENTATIVE TRANSCRIPT|sp|Q24270|CAC1D_DROME Voltage-dependent calcium channel type D subunit alpha-1 OS=Drosophila melanogaster GN=Ca-alpha1D PE=1 SV=2|e=0|from=1|to=2166|frame=1|Fragment_1
SLGFQGYFVSLFNRFDCFVVIGSIGEMILTNTKVMPPLGVS
>Locus_12799_Transcript_2/4_Confidence_0.667_Length_2171|UNIGENE REPRESENTATIVE TRANSCRIPT|sp|Q24270|CAC1D_DROME Voltage-dependent calcium channel type D subunit alpha-1 OS=Drosophila melanogaster GN=Ca-alpha1D PE=1 SV=2|e=0|from=1|to=2166|frame=1|Fragment_2
FKVTKYWR
>Locus_12799_Transcript_2/4_Confidence_0.667_Length_2171|UNIGENE REPRESENTATIVE TRANSCRIPT|sp|Q24270|CAC1D_DROME Voltage-dependent calcium channel type D subunit alpha-1 OS=Drosophila melanogaster GN=Ca-alpha1D PE=1 SV=2|e=0|from=1|to=2166|frame=1|Fragment_3
GMQVFGGKFNFDPTKDKPRGNFDSFWQSLLTVFQILTGEDWNVVMYDGIQAYGGVASIGI
LACIYFIILFICGNYILLNVFLAIAVDNLADAESLTAI
>Locus_12799_Transcript_2/4_Confidence_0.667_Length_2171|UNIGENE REPRESENTATIVE TRANSCRIPT|sp|Q24270|CAC1D_DROME Voltage-dependent calcium channel type D subunit alpha-1 OS=Drosophila melanogaster GN=Ca-alpha1D PE=1 SV=2|e=0|from=1|to=2166|frame=1|Fragment_4
SHSPTPVLDQDEECLDEDMDGDGERNMYDGEMSEQGDETASEAKVRL

>Locus_12799_Transcript_2/4_Confidence_0.667_Length_2171|UNIGENE REPRESENTATIVE TRANSCRIPT|sp|Q24270|CAC1D_DROME Voltage-dependent calcium channel type D subunit alpha-1 OS=Drosophila melanogaster GN=Ca-alpha1D PE=1 SV=2|e=0|from=1|to=2166|frame=1|Fragment_5
IIVESARPRRMSD
>Locus_12799_Transcript_2/4_Confidence_0.667_Length_2171|UNIGENE REPRESENTATIVE TRANSCRIPT|sp|Q24270|CAC1D_DROME Voltage-dependent calcium channel type D subunit alpha-1 OS=Drosophila melanogaster GN=Ca-alpha1D PE=1 SV=2|e=0|from=1|to=2166|frame=1|Fragment_6
GSAFFILSQTNRFRVFCHWLCNHSYFGNIILVCIMFSSAMLAAEDPLNAASERNQILNYF
DYFFTGAFTVELLLKLVSYGFVLHKGAFCRSAFNLLDLLVVCVSLISIYFSSGAIS
>Locus_12799_Transcript_2/4_Confidence_0.667_Length_2171|UNIGENE REPRESENTATIVE TRANSCRIPT|sp|Q24270|CAC1D_DROME Voltage-dependent calcium channel type D subunit alpha-1 OS=Drosophila melanogaster GN=Ca-alpha1D PE=1 SV=2|e=0|from=1|to=2166|frame=1|Fragment_7
AINRAKGLKHVVQCVIVAVKTIGNIVLVTCLLQFMFAVIGVQLFKGKFFYCSDGSKMIES
ECQGTYVVFEDGDINKPEMRERIWIRNKFHFDDVAKAMLTLFTVSTFEGWPNLLYVSIDS
NNENVGPIHNYRP
>Locus_12799_Transcript_2/4_Confidence_0.667_Length_2171|UNIGENE REPRESENTATIVE TRANSCRIPT|sp|Q24270|CAC1D_DROME Voltage-dependent calcium channel type D subunit alpha-1 OS=Drosophila melanogaster GN=Ca-alpha1D PE=1 SV=2|e=0|from=1|to=2166|frame=1|Fragment_8
GFVIVTFQNEGEQEYKNCELDKNQRNCIEFALKAKPVRRYIPKHRIQYKVWWFVTSQPFE
YTIFILIMINTITLAMKFHRQPDMYTHALDVLNMIFTAVFALEFVFKLAAFRFKNYFGDA
WNVFDFIIVLGSFIDIVYSEV
>Locus_12799_Transcript_4/4_Confidence_0.667_Length_2171|.|sp|Q24270|CAC1D_DROME Voltage-dependent calcium channel type D subunit alpha-1 OS=Drosophila melanogaster GN=Ca-alpha1D PE=1 SV=2|e=0|from=1|to=2166|frame=1|Fragment_1
SLGFQGYFVSLFNRFDCFVVIGSIGEMILTNTKVMPPLGVS
>Locus_12799_Transcript_4/4_Confidence_0.667_Length_2171|.|sp|Q24270|CAC1D_DROME Voltage-dependent calcium channel type D subunit alpha-1 OS=Drosophila melanogaster GN=Ca-alpha1D PE=1 SV=2|e=0|from=1|to=2166|frame=1|Fragment_2
FKVTKYWR
>Locus_12799_Transcript_4/4_Confidence_0.667_Length_2171|.|sp|Q24270|CAC1D_DROME Voltage-dependent calcium channel type D subunit alpha-1 OS=Drosophila melanogaster GN=Ca-alpha1D PE=1 SV=2|e=0|from=1|to=2166|frame=1|Fragment_3
GMQVFGGKFNFDPTKDKPRGNFDSFWQSLLTVFQILTGEDWNVVMYDGIQAYGGVASIGI
LACIYFIILFICGNYILLNVFLAIAVDNLADAESLTAI
>Locus_12799_Transcript_4/4_Confidence_0.667_Length_2171|.|sp|Q24270|CAC1D_DROME Voltage-dependent calcium channel type D subunit alpha-1 OS=Drosophila melanogaster GN=Ca-alpha1D PE=1 SV=2|e=0|from=1|to=2166|frame=1|Fragment_4
SHSPTPVLDQDEECLDEDMDGDGERNMYDGEMSEQGDETASEAKVRL
>Locus_12799_Transcript_4/4_Confidence_0.667_Length_2171|.|sp|Q24270|CAC1D_DROME Voltage-dependent calcium channel type D subunit alpha-1 OS=Drosophila melanogaster GN=Ca-alpha1D PE=1 SV=2|e=0|from=1|to=2166|frame=1|Fragment_5
IIVESARPRRMSD
>Locus_12799_Transcript_4/4_Confidence_0.667_Length_2171|.|sp|Q24270|CAC1D_DROME Voltage-dependent calcium channel type D subunit alpha-1 OS=Drosophila melanogaster GN=Ca-alpha1D PE=1 SV=2|e=0|

from=1|to=2166|frame=1|Fragment_6
GSAFFILSQTNRFRVFCHWLCNHSYFGNIILVCIMFSSAMLAAEDPLNAASERNQILNYF
DYFFTGAFTVELLLKLVSYGFVLHKGAFCRSAFNLLDLLVVCVSLISIYFSSGAIS
>Locus_12799_Transcript_4/4_Confidence_0.667_Length_2171|.|sp|
Q24270|CAC1D_DROME Voltage-dependent calcium channel type D subunit
alpha-1 OS=Drosophila melanogaster GN=Ca-alpha1D PE=1 SV=2|e=0|
from=1|to=2166|frame=1|Fragment_7
AINRAKGLKHVVQCVIVAVKTIGNIVLVTCLLQFMFAVIGVQLFKGKFFYCSDGSKMIES
ECQGTYVVFEDGDINKPEMRERIWIRNKFHFDDVAKAMLTLFTVSTFEGWPNLLYVSIDS
NNENVGPIHNYRP
>Locus_12799_Transcript_4/4_Confidence_0.667_Length_2171|.|sp|
Q24270|CAC1D_DROME Voltage-dependent calcium channel type D subunit
alpha-1 OS=Drosophila melanogaster GN=Ca-alpha1D PE=1 SV=2|e=0|
from=1|to=2166|frame=1|Fragment_8
GFVIVTFQNEGEQEYKNCELDKNQRNCIEFALKAKPVRRYIPKHRIQYKVWWFVTSQPFE
YTIFILIMINTITLAMKFHRQPDMYTHALDVLNMIFTAVFALEFVFKLAAFRFKNYFGDA
WNVFDFIIVLGSFIDIVYSEV
>Locus_12799_Transcript_2/4_Confidence_0.667_Length_2171|.|sp|
Q24270|CAC1D_DROME Voltage-dependent calcium channel type D subunit
alpha-1 OS=Drosophila melanogaster GN=Ca-alpha1D PE=1 SV=2|
e=1.11454E-17|from=892|to=1623|frame=1|Fragment_1
AFFILSQTNRFRVFCHWLCNHSYFGNIILVCIMFSSAMLAAEDPLNAASER
>Locus_12799_Transcript_2/4_Confidence_0.667_Length_2171|.|sp|
Q24270|CAC1D_DROME Voltage-dependent calcium channel type D subunit
alpha-1 OS=Drosophila melanogaster GN=Ca-alpha1D PE=1 SV=2|
e=1.11454E-17|from=892|to=1623|frame=1|Fragment_2
NQILNYFDYFFTGAFTVELLLKLVSYGFVLHKGAFCRSAFNLLDLLVVCVSLISIYFSS
>Locus_12799_Transcript_2/4_Confidence_0.667_Length_2171|.|sp|
Q24270|CAC1D_DROME Voltage-dependent calcium channel type D subunit
alpha-1 OS=Drosophila melanogaster GN=Ca-alpha1D PE=1 SV=2|
e=1.11454E-17|from=892|to=1623|frame=1|Fragment_3
GAIS
>Locus_12799_Transcript_2/4_Confidence_0.667_Length_2171|.|sp|
Q24270|CAC1D_DROME Voltage-dependent calcium channel type D subunit
alpha-1 OS=Drosophila melanogaster GN=Ca-alpha1D PE=1 SV=2|
e=1.11454E-17|from=892|to=1623|frame=1|Fragment_4
AINRAKGLKHVVQCVIVAVKTIGNIVLVTCLLQFMFAVIGVQLFKGKF
>Locus_12799_Transcript_2/4_Confidence_0.667_Length_2171|.|sp|
Q24270|CAC1D_DROME Voltage-dependent calcium channel type D subunit
alpha-1 OS=Drosophila melanogaster GN=Ca-alpha1D PE=1 SV=2|
e=1.11454E-17|from=892|to=1623|frame=1|Fragment_5
FYCSDGSKMIESECQGTYVVFEDGDINKPEMRERIWIRNKFHFDDVAKAMLTLFTVSTFE
GWPNLLY
>Locus_12799_Transcript_4/4_Confidence_0.667_Length_2171|.|sp|
Q24270|CAC1D_DROME Voltage-dependent calcium channel type D subunit
alpha-1 OS=Drosophila melanogaster GN=Ca-alpha1D PE=1 SV=2|
e=1.11454E-17|from=892|to=1623|frame=1|Fragment_1
AFFILSQTNRFRVFCHWLCNHSYFGNIILVCIMFSSAMLAAEDPLNAASER
>Locus_12799_Transcript_4/4_Confidence_0.667_Length_2171|.|sp|
Q24270|CAC1D_DROME Voltage-dependent calcium channel type D subunit
alpha-1 OS=Drosophila melanogaster GN=Ca-alpha1D PE=1 SV=2|
e=1.11454E-17|from=892|to=1623|frame=1|Fragment_2
NQILNYFDYFFTGAFTVELLLKLVSYGFVLHKGAFCRSAFNLLDLLVVCVSLISIYFSS
>Locus_12799_Transcript_4/4_Confidence_0.667_Length_2171|.|sp|

Q24270|CAC1D_DROME Voltage-dependent calcium channel type D subunit alpha-1 OS=Drosophila melanogaster GN=Ca-alpha1D PE=1 SV=2|
e=1.11454E-17|from=892|to=1623|frame=1|Fragment_3
GAIS
>Locus_12799_Transcript_4/4_Confidence_0.667_Length_2171|.|sp|
Q24270|CAC1D_DROME Voltage-dependent calcium channel type D subunit alpha-1 OS=Drosophila melanogaster GN=Ca-alpha1D PE=1 SV=2|
e=1.11454E-17|from=892|to=1623|frame=1|Fragment_4
AINRAKGLKHVVQCVIVAVKTIGNIVLVTCLLQFMFAVIGVQLFKGKF
>Locus_12799_Transcript_4/4_Confidence_0.667_Length_2171|.|sp|
Q24270|CAC1D_DROME Voltage-dependent calcium channel type D subunit alpha-1 OS=Drosophila melanogaster GN=Ca-alpha1D PE=1 SV=2|
e=1.11454E-17|from=892|to=1623|frame=1|Fragment_5
FYCSDGSKMIESECQGTYVVFEDGDINKPEMRERIWIRNKFHFDDVAKAMLTLFTVSTFE
GWPNLLY
>Locus_12799_Transcript_1/4_Confidence_0.556_Length_2123|.|sp|
Q24270|CAC1D_DROME Voltage-dependent calcium channel type D subunit alpha-1 OS=Drosophila melanogaster GN=Ca-alpha1D PE=1 SV=2|e=0|
from=1|to=2118|frame=1|Fragment_1
CFVVIGSIGEMILTNTKVMPPLGVS
>Locus_12799_Transcript_1/4_Confidence_0.556_Length_2123|.|sp|
Q24270|CAC1D_DROME Voltage-dependent calcium channel type D subunit alpha-1 OS=Drosophila melanogaster GN=Ca-alpha1D PE=1 SV=2|e=0|
from=1|to=2118|frame=1|Fragment_2
FKVTKYWR
>Locus_12799_Transcript_1/4_Confidence_0.556_Length_2123|.|sp|
Q24270|CAC1D_DROME Voltage-dependent calcium channel type D subunit alpha-1 OS=Drosophila melanogaster GN=Ca-alpha1D PE=1 SV=2|e=0|
from=1|to=2118|frame=1|Fragment_3
GMQVFGGKFNFDPTKDKPRGNFDSFWQSLLTVFQILTGEDWNVVMYDGIQAYGGVASIGI
LACIYFIILFICGNYILLNVFLAIAVDNLADAESLTAI
>Locus_12799_Transcript_1/4_Confidence_0.556_Length_2123|.|sp|
Q24270|CAC1D_DROME Voltage-dependent calcium channel type D subunit alpha-1 OS=Drosophila melanogaster GN=Ca-alpha1D PE=1 SV=2|e=0|
from=1|to=2118|frame=1|Fragment_4
SHSPTPVLDQDEECLDEDMDGDGERNMYDGEMSEQGDETASEAKVRL
>Locus_12799_Transcript_1/4_Confidence_0.556_Length_2123|.|sp|
Q24270|CAC1D_DROME Voltage-dependent calcium channel type D subunit alpha-1 OS=Drosophila melanogaster GN=Ca-alpha1D PE=1 SV=2|e=0|
from=1|to=2118|frame=1|Fragment_5
IIVESARPRRMSD
>Locus_12799_Transcript_1/4_Confidence_0.556_Length_2123|.|sp|
Q24270|CAC1D_DROME Voltage-dependent calcium channel type D subunit alpha-1 OS=Drosophila melanogaster GN=Ca-alpha1D PE=1 SV=2|e=0|
from=1|to=2118|frame=1|Fragment_6
GSAFFILSQTNRFRVFCHWLCNHSYFGNIILVCIMFSSAMLAAEDPLNAASERNQILNYF
DYFFTGAFTVELLLKLVSYGFVLHKGAFCRSAFNLLDLLVVCVSLISIYFSSGAIS
>Locus_12799_Transcript_1/4_Confidence_0.556_Length_2123|.|sp|
Q24270|CAC1D_DROME Voltage-dependent calcium channel type D subunit alpha-1 OS=Drosophila melanogaster GN=Ca-alpha1D PE=1 SV=2|e=0|
from=1|to=2118|frame=1|Fragment_7
AINRAKGLKHVVQCVIVAVKTIGNIVLVTCLLQFMFAVIGVQLFKGKFFYCSDGSKMIES
ECQGTYVVFEDGDINKPEMRERIWIRNKFHFDDVAKAMLTLFTVSTFEGWPNLLYVSIDS
NNENVGPIHNYRP

>Locus_12799_Transcript_1/4_Confidence_0.556_Length_2123|.|sp|
Q24270|CAC1D_DROME Voltage-dependent calcium channel type D subunit
alpha-1 OS=Drosophila melanogaster GN=Ca-alpha1D PE=1 SV=2|e=0|
from=1|to=2118|frame=1|Fragment_8
GFVIVTFQNEGEQEYKNCELDKNQRNCIEFALKAKPVRRYIPKHRIQYKVWWFVTSQPFE
YTIFILIMINTITLAMKFHRQPDMYTHALDVLNMIFTAVFALEFVFKLAAFRFKNYFGDA
WNVFDFIIVLGSFIDIVYSEV
>Locus_12799_Transcript_1/4_Confidence_0.556_Length_2123|.|sp|
Q24270|CAC1D_DROME Voltage-dependent calcium channel type D subunit
alpha-1 OS=Drosophila melanogaster GN=Ca-alpha1D PE=1 SV=2|
e=1.08507E-17|from=844|to=1575|frame=1|Fragment_1
AFFILSQTNRFRVFCHWLCNHSYFGNIILVCIMFSSAMLAAEDPLNAASER
>Locus_12799_Transcript_1/4_Confidence_0.556_Length_2123|.|sp|
Q24270|CAC1D_DROME Voltage-dependent calcium channel type D subunit
alpha-1 OS=Drosophila melanogaster GN=Ca-alpha1D PE=1 SV=2|
e=1.08507E-17|from=844|to=1575|frame=1|Fragment_2
NQILNYFDYFFTGAFTVELLLKLVSYGFVLHKGAFCRSAFNLLDLLVVCVSLISIYFSS
>Locus_12799_Transcript_1/4_Confidence_0.556_Length_2123|.|sp|
Q24270|CAC1D_DROME Voltage-dependent calcium channel type D subunit
alpha-1 OS=Drosophila melanogaster GN=Ca-alpha1D PE=1 SV=2|
e=1.08507E-17|from=844|to=1575|frame=1|Fragment_3
GAIS
>Locus_12799_Transcript_1/4_Confidence_0.556_Length_2123|.|sp|
Q24270|CAC1D_DROME Voltage-dependent calcium channel type D subunit
alpha-1 OS=Drosophila melanogaster GN=Ca-alpha1D PE=1 SV=2|
e=1.08507E-17|from=844|to=1575|frame=1|Fragment_4
AINRAKGLKHVVQCVIVAVKTIGNIVLVTCLLQFMFAVIGVQLFKGKF
>Locus_12799_Transcript_1/4_Confidence_0.556_Length_2123|.|sp|
Q24270|CAC1D_DROME Voltage-dependent calcium channel type D subunit
alpha-1 OS=Drosophila melanogaster GN=Ca-alpha1D PE=1 SV=2|
e=1.08507E-17|from=844|to=1575|frame=1|Fragment_5
FYCSDGSKMIESECQGTYVVFEDGDINKPEMRERIWIRNKFHFDDVAKAMLTLFTVSTFE
GWPNLLY
>Locus_15291_Transcript_2/3_Confidence_0.667_Length_2247|UNIGENE
REPRESENTATIVE TRANSCRIPT|sp|Q24278|CNG_DROME Cyclic nucleotide-
gated cation channel OS=Drosophila melanogaster GN=Cng PE=2 SV=2|
e=0|from=449|to=2233|frame=2|Fragment_1
ISDTPNQNIQDEESRASRWYQEFRTFISERILPKGSVKSAAKAPDWFLDK
>Locus_15291_Transcript_2/3_Confidence_0.667_Length_2247|UNIGENE
REPRESENTATIVE TRANSCRIPT|sp|Q24278|CNG_DROME Cyclic nucleotide-
gated cation channel OS=Drosophila melanogaster GN=Cng PE=2 SV=2|
e=0|from=449|to=2233|frame=2|Fragment_2
QDNKNQNN
>Locus_15291_Transcript_2/3_Confidence_0.667_Length_2247|UNIGENE
REPRESENTATIVE TRANSCRIPT|sp|Q24278|CNG_DROME Cyclic nucleotide-
gated cation channel OS=Drosophila melanogaster GN=Cng PE=2 SV=2|
e=0|from=449|to=2233|frame=2|Fragment_3
EDATVICGYPLVVDTTLNSHYRWLALVSLAVLYNIIFVVGRAVFWEINNKIPAFWFTMDY
FCDLIYWLDVAVHAHEGYLDQGLMVRDSSKLRSHYIASGSWRLDLLSLLPTDIMYIWWQP
STCQ
>Locus_15291_Transcript_2/3_Confidence_0.667_Length_2247|UNIGENE
REPRESENTATIVE TRANSCRIPT|sp|Q24278|CNG_DROME Cyclic nucleotide-
gated cation channel OS=Drosophila melanogaster GN=Cng PE=2 SV=2|
e=0|from=449|to=2233|frame=2|Fragment_4

LRVPCPVIVRLNRLLRAPRMWEWFDRTETATGYPNAFRICKVLIAILILIHWNACLYFAI
SYGLGFGTDHWVYNLNGPRNSSLSRQYIYSFYWS
>Locus_15291_Transcript_2/3_Confidence_0.667_Length_2247|UNIGENE
REPRESENTATIVE TRANSCRIPT|sp|Q24278|CNG_DROME Cyclic nucleotide-
gated cation channel OS=Drosophila melanogaster GN=Cng PE=2 SV=2|
e=0|from=449|to=2233|frame=2|Fragment_5
NDAEYLFVVCDFLAGVLIFATIVGNIGSMISNMNVAKVDFQNRMDGVKQYMAFRKISGEL
EARVIRWFAYTWSQSGALDEEKVLSALPDKLKAEIAIVVHMETLRKVTIFQDCEPGLLEA
LVLKLRLQIFSPADYICRKGDVGKEMYIVKR
>Locus_15291_Transcript_2/3_Confidence_0.667_Length_2247|UNIGENE
REPRESENTATIVE TRANSCRIPT|sp|Q24278|CNG_DROME Cyclic nucleotide-
gated cation channel OS=Drosophila melanogaster GN=Cng PE=2 SV=2|
e=0|from=449|to=2233|frame=2|Fragment_6
ATLSAGSVFGELSVLEISGNRTGNRRSANVKSLGYSDLFCLAKKDLWEVLENFQEAKEML
IEKGVELLRKDGLLDEQALERSRRGKDSLKHEVDKLGQTVDSLGTRLARLMAECSAAQAK
LKQRLTRLE
>Locus_15291_Transcript_3/3_Confidence_0.667_Length_2247|.|sp|
Q24278|CNG_DROME Cyclic nucleotide-gated cation channel
OS=Drosophila melanogaster GN=Cng PE=2 SV=2|e=0|from=449|to=2233|
frame=2|Fragment_1
ISDTPNQNIQDEESRASRWYQEFRTFISERILPKGSVKSAAKAPDWFLDK
>Locus_15291_Transcript_3/3_Confidence_0.667_Length_2247|.|sp|
Q24278|CNG_DROME Cyclic nucleotide-gated cation channel
OS=Drosophila melanogaster GN=Cng PE=2 SV=2|e=0|from=449|to=2233|
frame=2|Fragment_2
QDNKNQNN
>Locus_15291_Transcript_3/3_Confidence_0.667_Length_2247|.|sp|
Q24278|CNG_DROME Cyclic nucleotide-gated cation channel
OS=Drosophila melanogaster GN=Cng PE=2 SV=2|e=0|from=449|to=2233|
frame=2|Fragment_3
EDATVICGYPLVVDTTLNSHYRWLALVSLAVLYNIIFVVGRAVFWEINNKIPAFWFTMDY
FCDLIYWLDVAVHAHEGYLDQGLMVRDSSKLRSHYIASGSWRLDLLSLLPTDIMYIWWQP
STCQ
>Locus_15291_Transcript_3/3_Confidence_0.667_Length_2247|.|sp|
Q24278|CNG_DROME Cyclic nucleotide-gated cation channel
OS=Drosophila melanogaster GN=Cng PE=2 SV=2|e=0|from=449|to=2233|
frame=2|Fragment_4
LRVPCPVIVRLNRLLRAPRMWEWFDRTETATGYPNAFRICKVLIAILILIHWNACLYFAI
SYGLGFGTDHWVYNLNGPRNSSLSRQYIYSFYWS
>Locus_15291_Transcript_3/3_Confidence_0.667_Length_2247|.|sp|
Q24278|CNG_DROME Cyclic nucleotide-gated cation channel
OS=Drosophila melanogaster GN=Cng PE=2 SV=2|e=0|from=449|to=2233|
frame=2|Fragment_5
NDAEYLFVVCDFLAGVLIFATIVGNIGSMISNMNVAKVDFQNRMDGVKQYMAFRKISGEL
EARVIRWFAYTWSQSGALDEEKVLSALPDKLKAEIAIVVHMETLRKVTIFQDCEPGLLEA
LVLKLRLQIFSPADYICRKGDVGKEMYIVKR
>Locus_15291_Transcript_3/3_Confidence_0.667_Length_2247|.|sp|
Q24278|CNG_DROME Cyclic nucleotide-gated cation channel
OS=Drosophila melanogaster GN=Cng PE=2 SV=2|e=0|from=449|to=2233|
frame=2|Fragment_6
ATLSAGSVFGELSVLEISGNRTGNRRSANVKSLGYSDLFCLAKKDLWEVLENFQEAKEML
IEKGVELLRKDGLLDEQALERSRRGKDSLKHEVDKLGQTVDSLGTRLARLMAECSAAQAK
LKQRLTRLE
>Locus_9542_Transcript_2/4_Confidence_0.700_Length_1254|UNIGENE

REPRESENTATIVE TRANSCRIPT|sp|Q24451|MAN2_DROME Alpha-mannosidase 2
OS=Drosophila melanogaster GN=alpha-Man-II PE=1 SV=2|e=2.67907E-76|
from=1|to=1161|frame=1|Fragment_1
PYVKVYDSKDRPVHCQLSPVWLWSEGV
>Locus_9542_Transcript_2/4_Confidence_0.700_Length_1254|UNIGENE
REPRESENTATIVE TRANSCRIPT|sp|Q24451|MAN2_DROME Alpha-mannosidase 2
OS=Drosophila melanogaster GN=alpha-Man-II PE=1 SV=2|e=2.67907E-76|
from=1|to=1161|frame=1|Fragment_2
PQASPVRYQLGCMIEVPALTLIALAVQAAEASDNGLPSFASVTLLTDENSVLHQQVTS
>Locus_9542_Transcript_2/4_Confidence_0.700_Length_1254|UNIGENE
REPRESENTATIVE TRANSCRIPT|sp|Q24451|MAN2_DROME Alpha-mannosidase 2
OS=Drosophila melanogaster GN=alpha-Man-II PE=1 SV=2|e=2.67907E-76|
from=1|to=1161|frame=1|Fragment_3
ELGEYPGALTLQPQRDFTVDVALDSDGVVTQSISFASDGTLKSITKPDDEP
>Locus_9542_Transcript_2/4_Confidence_0.700_Length_1254|UNIGENE
REPRESENTATIVE TRANSCRIPT|sp|Q24451|MAN2_DROME Alpha-mannosidase 2
OS=Drosophila melanogaster GN=alpha-Man-II PE=1 SV=2|e=2.67907E-76|
from=1|to=1161|frame=1|Fragment_4
MPIHLSFVKYGARKGGERSGAYLFLPDLPAQTVQNSLDSKSVVVIIKGPLESRVQVNLPY
VLHETIMRTIQSSEPRNEESTILEIRNLVDVANTHNAELAMRIATDISNKDEFFTDLNGL
QMIRRKRFDKLPLQANYYPVPSAMFLQDETRRMTLISGQPLGGTSPRSGTLEIMQDRRLN
QDDNRGLDQGVLDNKPVLNHFKLILE
>Locus_9542_Transcript_2/4_Confidence_0.700_Length_1254|UNIGENE
REPRESENTATIVE TRANSCRIPT|sp|Q24451|MAN2_DROME Alpha-mannosidase 2
OS=Drosophila melanogaster GN=alpha-Man-II PE=1 SV=2|e=2.67907E-76|
from=1|to=1161|frame=1|Fragment_5
TRKCAGEQHDEEPANPAGYLSKEALLQRDLMHYPIDKMLYSQDGW
>Locus_9542_Transcript_3/4_Confidence_0.700_Length_1254|.|sp|Q24451|
MAN2_DROME Alpha-mannosidase 2 OS=Drosophila melanogaster GN=alpha-
Man-II PE=1 SV=2|e=2.67907E-76|from=1|to=1161|frame=1|Fragment_1
PYVKVYDSKDRPVHCQLSPVWLWSEGV
>Locus_9542_Transcript_3/4_Confidence_0.700_Length_1254|.|sp|Q24451|
MAN2_DROME Alpha-mannosidase 2 OS=Drosophila melanogaster GN=alpha-
Man-II PE=1 SV=2|e=2.67907E-76|from=1|to=1161|frame=1|Fragment_2
PQASPVRYQLGCMIEVPALTLIALAVQAAEASDNGLPSFASVTLLTDENSVLHQQVTS
>Locus_9542_Transcript_3/4_Confidence_0.700_Length_1254|.|sp|Q24451|
MAN2_DROME Alpha-mannosidase 2 OS=Drosophila melanogaster GN=alpha-
Man-II PE=1 SV=2|e=2.67907E-76|from=1|to=1161|frame=1|Fragment_3
ELGEYPGALTLQPQRDFTVDVALDSDGVVTQSISFASDGTLKSITKPDDEP
>Locus_9542_Transcript_3/4_Confidence_0.700_Length_1254|.|sp|Q24451|
MAN2_DROME Alpha-mannosidase 2 OS=Drosophila melanogaster GN=alpha-
Man-II PE=1 SV=2|e=2.67907E-76|from=1|to=1161|frame=1|Fragment_4
MPIHLSFVKYGARKGGERSGAYLFLPDLPAQTVQNSLDSKSVVVIIKGPLESRVQVNLPY
VLHETIMRIIQSSEPRNEESTILEIRNLVDVANTHNAELAMRIATDISNKDEFFTDLNGL
QMIRRKRFDKLPLQANYYPVPSAMFLQDETRRMTLISGQPLGGTSPRSGTLEIMQDRRLN
QDDNRGLDQGVLDNKPVLNHFKLILE
>Locus_9542_Transcript_3/4_Confidence_0.700_Length_1254|.|sp|Q24451|
MAN2_DROME Alpha-mannosidase 2 OS=Drosophila melanogaster GN=alpha-
Man-II PE=1 SV=2|e=2.67907E-76|from=1|to=1161|frame=1|Fragment_5
TRKCAGEQHDEEPANPAGYLSKEALLQRDLMHYPIDKMLYSQDGW
>Locus_9542_Transcript_4/4_Confidence_0.700_Length_1254|.|sp|Q24451|
MAN2_DROME Alpha-mannosidase 2 OS=Drosophila melanogaster GN=alpha-
Man-II PE=1 SV=2|e=2.67907E-76|from=1|to=1161|frame=1|Fragment_1
PYVKVYDSKDRPVHCQLSPVWLWSEGV

>Locus_9542_Transcript_4/4_Confidence_0.700_Length_1254|.|sp|Q24451|
MAN2_DROME Alpha-mannosidase 2 OS=Drosophila melanogaster GN=alpha-
Man-II PE=1 SV=2|e=2.67907E-76|from=1|to=1161|frame=1|Fragment_2
PQASPVRYQLGCMIEVPALTLIALAVQAAEASDNGLPSFASVTLLTDENSVLHQQVTS
>Locus_9542_Transcript_4/4_Confidence_0.700_Length_1254|.|sp|Q24451|
MAN2_DROME Alpha-mannosidase 2 OS=Drosophila melanogaster GN=alpha-
Man-II PE=1 SV=2|e=2.67907E-76|from=1|to=1161|frame=1|Fragment_3
ELGEYPGALTLQPQRDFTVDVALDSDGVVTQSISFASDGTLKSITKPDDEP
>Locus_9542_Transcript_4/4_Confidence_0.700_Length_1254|.|sp|Q24451|
MAN2_DROME Alpha-mannosidase 2 OS=Drosophila melanogaster GN=alpha-
Man-II PE=1 SV=2|e=2.67907E-76|from=1|to=1161|frame=1|Fragment_4
MPIHLSFVKYGARKGGERSGAYLFLPDLPAQTVQNSLDSKSVVVIIKGPLESRVQVNLPY
VLHETIMRTIQSSEPRNEESTILEIRNLVDVANTHNAELAMRIATDISNKDEFFTDLNGL
QMIRRKRFDKLPLQANYYPVPSAMFLQDETRRMTLISGQPLGGTSPRSGTLEIMQDRRLN
QDDNRGLDQGVLDNKPVLNHFKLILE
>Locus_9542_Transcript_4/4_Confidence_0.700_Length_1254|.|sp|Q24451|
MAN2_DROME Alpha-mannosidase 2 OS=Drosophila melanogaster GN=alpha-
Man-II PE=1 SV=2|e=2.67907E-76|from=1|to=1161|frame=1|Fragment_5
TRKCAGEQHDEEPANPAGYLSKEALLQRDLMHYPIDKMLYSQDGW
>Locus_9542_Transcript_1/4_Confidence_0.700_Length_1254|.|sp|Q24451|
MAN2_DROME Alpha-mannosidase 2 OS=Drosophila melanogaster GN=alpha-
Man-II PE=1 SV=2|e=2.26797E-75|from=1|to=1161|frame=1|Fragment_1
PYVKVYDSKDRPVHCQLSPVWLWSEGV
>Locus_9542_Transcript_1/4_Confidence_0.700_Length_1254|.|sp|Q24451|
MAN2_DROME Alpha-mannosidase 2 OS=Drosophila melanogaster GN=alpha-
Man-II PE=1 SV=2|e=2.26797E-75|from=1|to=1161|frame=1|Fragment_2
PQASPVRYQLGCMIEVSALTLIALAVQAAEASDNGLPSFASVTLLTDENSVLHQQVTS
>Locus_9542_Transcript_1/4_Confidence_0.700_Length_1254|.|sp|Q24451|
MAN2_DROME Alpha-mannosidase 2 OS=Drosophila melanogaster GN=alpha-
Man-II PE=1 SV=2|e=2.26797E-75|from=1|to=1161|frame=1|Fragment_3
ELGEYPGALTLQPQRDFTVDVALDSDGVVTQSISFASDGTLKSITKPDDEP
>Locus_9542_Transcript_1/4_Confidence_0.700_Length_1254|.|sp|Q24451|
MAN2_DROME Alpha-mannosidase 2 OS=Drosophila melanogaster GN=alpha-
Man-II PE=1 SV=2|e=2.26797E-75|from=1|to=1161|frame=1|Fragment_4
MPIHLSFVKYGARKGGERSGAYLFLPDLPAQTVQNSLDSKSVVVIIKGPLESRVQVNLPY
VLHETIMRTIQSSEPRNEESTILEIRNLVDVANTHNAELAMRIATDISNKDEFFTDLNGL
QMIRRKRFDKLPLQANYYPVPSAMFLQDETRRMTLISGQPLGGTSPRSGTLEIMQDRRLN
QDDNRGLDQGVLDNKPVLNHFKLILE
>Locus_9542_Transcript_1/4_Confidence_0.700_Length_1254|.|sp|Q24451|
MAN2_DROME Alpha-mannosidase 2 OS=Drosophila melanogaster GN=alpha-
Man-II PE=1 SV=2|e=2.26797E-75|from=1|to=1161|frame=1|Fragment_5
TRKCAGEQHDEEPANPAGYLSKEALLQRDLMHYPIDKMLYSQDGW
>Locus_10403_Transcript_1/3_Confidence_0.667_Length_3725|UNIGENE
REPRESENTATIVE TRANSCRIPT|sp|Q24498|RY44_DROME Ryanodine receptor
44F OS=Drosophila melanogaster GN=Rya-r44F PE=1 SV=3|e=0|from=3|
to=3578|frame=-1|Fragment_1
MAEAEGGSEQDDVSFLRTEDMVCLSCTATGERVCLAAEGFGNRHCFLENIADKNIPPDLS
QCVFVIEQALSVRALQELV
>Locus_10403_Transcript_1/3_Confidence_0.667_Length_3725|UNIGENE
REPRESENTATIVE TRANSCRIPT|sp|Q24498|RY44_DROME Ryanodine receptor
44F OS=Drosophila melanogaster GN=Rya-r44F PE=1 SV=3|e=0|from=3|
to=3578|frame=-1|Fragment_2
GHRTLLYGNAILLRHQNSDMYLACLSTSSSNDKLSFDVGLQEHSKGEACWWTVHPASKQR
SEGEKVRVGDDLILVSVATERYLHTTKENDLSVVNASFHVTHWSVQPYGTGISKMKYVGY

VFGGDVLRFFHGGDECLTIPSTWSETLGQNIVVYEGGSVMSQARSLWRLELARTKWAGGF
INWYHPMRIRHITTGRYLGVNENNELYLVRREEATTASTAFCLRQEKDDQKVVLEDKDLE
VIGSPIIKYGDSTVIMQHSESGLWLSYKSYET
>Locus_10403_Transcript_1/3_Confidence_0.667_Length_3725|UNIGENE REPRESENTATIVE TRANSCRIPT|sp|Q24498|RY44_DROME Ryanodine receptor 44F OS=Drosophila melanogaster GN=Rya-r44F PE=1 SV=3|e=0|from=3|to=3578|frame=-1|Fragment_3
KQAILHEEGKMDDGLDFSRSQEEESRTARVIRKCSSLFTKFINGLETLQTNRRHSLFFAS
VNLSEMVMCLEDLINYFAQPDEEMEHEEKQNRFRALRNRQDLFQEEGILNLILEAIDKIN
VITSQGFLAGFLAGDDAGQSWEMISGYLYQLLAAIIKGNHTNCAQFANSNRLNWLFSRLG
SQASSEGTGMLDVLHCVLIDSPEALNMMRDEHIKVIISLLEKHGRDPKVLDVLCSLCVGN
GVAVRSSQNNICDFLLPGKNLLLQTQLVDHVASIRPNIFVGKVEGSAVYQKWYFEVTMDH
LEQTTHMMPHLRIGWANTTGYVPYP
>Locus_10403_Transcript_1/3_Confidence_0.667_Length_3725|UNIGENE REPRESENTATIVE TRANSCRIPT|sp|Q24498|RY44_DROME Ryanodine receptor 44F OS=Drosophila melanogaster GN=Rya-r44F PE=1 SV=3|e=0|from=3|to=3578|frame=-1|Fragment_4
GDDLYSFGFDGAYLWTGGRKSLVLPG
>Locus_10403_Transcript_1/3_Confidence_0.667_Length_3725|UNIGENE REPRESENTATIVE TRANSCRIPT|sp|Q24498|RY44_DROME Ryanodine receptor 44F OS=Drosophila melanogaster GN=Rya-r44F PE=1 SV=3|e=0|from=3|to=3578|frame=-1|Fragment_5
AEEPYIRKGDVVGCALDLSIPLITFTMNGMRIHGSFTDFNLDGMFHPVMSCSSKLSCRFL
LGGDHGRLKYAPPEGFSPLVECLQPQQILSLDPCFYFGNLNKSVLAGPWLVEDDTAFVPK
PVDTTSVTMPSYVETIRDKLAENIHEMWALNKVESGWCWGERRDDVHKIHPCLTQFDNLP
AAEKKYDSQLAIQTLKTIIALGYYISMDKPPARIRQIRLPNEPFLQANGYKPAPLDLSAV
TLTPKMDELVDQLAENTHNLWAKERIQQGWTYGLNEDSDNLRSPHLVPYAKVDEAIKKAN
RDTASETVRTLLVYGYNLEPPQGEANEALLAEAHRLRHSGFRTYRVEKTYAVTSGKWYFE
FEILTAGPMRVGWARSDCPPGQMLGQDEYTWAFDGYNEEKVFSGNAESFGKQWAVGDVVG
VFLDLIDHTISFSLNGELLMDALGGETSFADVQGEG
>Locus_10403_Transcript_3/3_Confidence_0.667_Length_3725|.|sp|Q24498|RY44_DROME Ryanodine receptor 44F OS=Drosophila melanogaster GN=Rya-r44F PE=1 SV=3|e=0|from=3|to=3578|frame=-1|Fragment_1
MAEAEGGSEQDDVSFLRTEDMVCLSCTATGERVCLAAEGFGNRHCFLENIADKNIPPDLS
QCVFVIEQALSVRALQELV
>Locus_10403_Transcript_3/3_Confidence_0.667_Length_3725|.|sp|Q24498|RY44_DROME Ryanodine receptor 44F OS=Drosophila melanogaster GN=Rya-r44F PE=1 SV=3|e=0|from=3|to=3578|frame=-1|Fragment_2
GHRTLLYGNAILLRHQNSDMYLACLSTSSSNDKLSFDVGLQEHSKGEACWWTVHPASKQR
SEGEKVRVGDDLILVSVATERYLHTTKENDLSVVNASFHVTHWSVQPYGTGISKMKYVGY
VFGGDVLRFFHGGDECLTIPSTWSETLGQNIVVYEGGSVMSQARSLWRLELARTKWAGGF
INWYHPMRIRHITTGRYLGVNENNELYLVRREEATTASTAFCLRQEKDDQKVVLEDKDLE
VIGSPIIKYGDSTVIMQHSESGLWLSYKSYET
>Locus_10403_Transcript_3/3_Confidence_0.667_Length_3725|.|sp|Q24498|RY44_DROME Ryanodine receptor 44F OS=Drosophila melanogaster GN=Rya-r44F PE=1 SV=3|e=0|from=3|to=3578|frame=-1|Fragment_3
KQAILHEEGKMDDGLDFSRSQEEESRTARVIRKCSSLFTKFINGLETLQTNRRHSLFFAS
VNLSEMVMCLEDLINYFAQPDEEMEHEEKQNRFRALRNRQDLFQEEGILNLILEAIDKIN
VITSQGFLAGFLAGDDAGQSWEMISGYLYQLLAAIIKGNHTNCAQFANSNRLNWLFSRLG
SQASSEGTGMLDVLHCVLIDSPEALNMMRDEHIKVIISLLEKHGRDPKVLDVLCSLCVGN
GVAVRSSQNNICDFLLPGKNLLLQTQLVDHVASIRPNIFVGKVEGSAVYQKWYFEVTMDH
LEQTTHMMPHLRIGWANTTGYVPYP
>Locus_10403_Transcript_3/3_Confidence_0.667_Length_3725|.|sp|Q24498|RY44_DROME Ryanodine receptor 44F OS=Drosophila melanogaster

GN=Rya-r44F PE=1 SV=3|e=0|from=3|to=3578|frame=-1|Fragment_4
GDDLYSFGFDGAYLWTGGRKSLVLPG
>Locus_10403_Transcript_3/3_Confidence_0.667_Length_3725|.|sp|
Q24498|RY44_DROME Ryanodine receptor 44F OS=Drosophila melanogaster
GN=Rya-r44F PE=1 SV=3|e=0|from=3|to=3578|frame=-1|Fragment_5
AEEPYIRKGDVVGCALDLSIPLITFTMNGMRIHGSFTDFNLDGMFHPVMSCSSKLSCRFL
LGGDHGRLKYAPPEGFSPLVECLQPQQILSLDPCFYFGNLNKSVLAGPWLVEDDTAFVPK
PVDTTSVTMPSYVETIRDKLAENIHEMWALNKVESGWCWGERRDDVHKIHPCLTQFDNLP
AAEKKYDSQLAIQTLKTIIALGYYISMDKPPARIRQIRLPNEPFLQANGYKPAPLDLSAV
TLTPKMDELVDQLAENTHNLWAKERIQQGWTYGLNEDSDNLRSPHLVPYAKVDEAIKKAN
RDTASETVRTLLVYGYNLEPPQGEANEALLAEAHRLRHSGFRTYRVEKTYAVTSGKWYFE
FEILTAGPMRVGWARSDCPPGQMLGQDEYTWAFDGYNEEKVFSGNAESFGKQWAVGDVVG
VFLDLIDHTISFSLNGELLMDALGGETSFADVQGEG
>Locus_10403_Transcript_1/3_Confidence_0.667_Length_3725|.|sp|
Q24498|RY44_DROME Ryanodine receptor 44F OS=Drosophila melanogaster
GN=Rya-r44F PE=1 SV=3|e=2.08974E-22|from=408|to=1034|frame=-1|
Fragment_1
DDTAFVPKPVDTTSVTMPSYVETIRDKLAENIHEMWALNKVESGWCWGERRDDVHKIHPC
LTQFDNLPAAEKKYDSQLAIQTLKTIIALGYYIS
>Locus_10403_Transcript_1/3_Confidence_0.667_Length_3725|.|sp|
Q24498|RY44_DROME Ryanodine receptor 44F OS=Drosophila melanogaster
GN=Rya-r44F PE=1 SV=3|e=2.08974E-22|from=408|to=1034|frame=-1|
Fragment_2
MDKPPARIRQIRLPNEPFLQANGYKPAPLDLSAVTLTPKMDELVDQLAENTHNLWAKERI
QQGWTYGLNEDSDNLRSPHLVPYAKVDEAIKKANRDTASETVRTLLVYGYNLEPP
>Locus_10403_Transcript_3/3_Confidence_0.667_Length_3725|.|sp|
Q24498|RY44_DROME Ryanodine receptor 44F OS=Drosophila melanogaster
GN=Rya-r44F PE=1 SV=3|e=2.08974E-22|from=408|to=1034|frame=-1|
Fragment_1
DDTAFVPKPVDTTSVTMPSYVETIRDKLAENIHEMWALNKVESGWCWGERRDDVHKIHPC
LTQFDNLPAAEKKYDSQLAIQTLKTIIALGYYIS
>Locus_10403_Transcript_3/3_Confidence_0.667_Length_3725|.|sp|
Q24498|RY44_DROME Ryanodine receptor 44F OS=Drosophila melanogaster
GN=Rya-r44F PE=1 SV=3|e=2.08974E-22|from=408|to=1034|frame=-1|
Fragment_2
MDKPPARIRQIRLPNEPFLQANGYKPAPLDLSAVTLTPKMDELVDQLAENTHNLWAKERI
QQGWTYGLNEDSDNLRSPHLVPYAKVDEAIKKANRDTASETVRTLLVYGYNLEPP
>Locus_10403_Transcript_1/3_Confidence_0.667_Length_3725|.|sp|
Q24498|RY44_DROME Ryanodine receptor 44F OS=Drosophila melanogaster
GN=Rya-r44F PE=1 SV=3|e=4.36749E-12|from=399|to=845|frame=-1|
Fragment_1
FDNLPAAEKKYDSQLAIQTLKTIIALGYYISMDKPPARI
>Locus_10403_Transcript_1/3_Confidence_0.667_Length_3725|.|sp|
Q24498|RY44_DROME Ryanodine receptor 44F OS=Drosophila melanogaster
GN=Rya-r44F PE=1 SV=3|e=4.36749E-12|from=399|to=845|frame=-1|
Fragment_2
RQIRLPNEPFLQANGYKPAPLDLSAVTLTPKMDELVDQLAENTHNLWAKERIQQGWTYGL
NEDSDNLRSPHLVPYAKVDEAIKKANRDTASETVRTLLVYGYNLEPPQGE
>Locus_10403_Transcript_3/3_Confidence_0.667_Length_3725|.|sp|
Q24498|RY44_DROME Ryanodine receptor 44F OS=Drosophila melanogaster
GN=Rya-r44F PE=1 SV=3|e=4.36749E-12|from=399|to=845|frame=-1|
Fragment_1
FDNLPAAEKKYDSQLAIQTLKTIIALGYYISMDKPPARI
>Locus_10403_Transcript_3/3_Confidence_0.667_Length_3725|.|sp|

```
Q24498|RY44_DROME Ryanodine receptor 44F OS=Drosophila melanogaster
GN=Rya-r44F PE=1 SV=3|e=4.36749E-12|from=399|to=845|frame=-1|
Fragment_2
RQIRLPNEPFLQANGYKPAPLDLSAVTLTPKMDELVDQLAENTHNLWAKERIQQGWTYGL
NEDSDNLRSPHLVPYAKVDEAIKKANRDTASETVRTLLVYGYNLEPPQGE
>Locus_10403_Transcript_2/3_Confidence_0.333_Length_2537|.|sp|
Q24498|RY44_DROME Ryanodine receptor 44F OS=Drosophila melanogaster
GN=Rya-r44F PE=1 SV=3|e=0|from=3|to=2537|frame=-1|Fragment_1
MQHSESGLWLSYKSYET
>Locus_10403_Transcript_2/3_Confidence_0.333_Length_2537|.|sp|
Q24498|RY44_DROME Ryanodine receptor 44F OS=Drosophila melanogaster
GN=Rya-r44F PE=1 SV=3|e=0|from=3|to=2537|frame=-1|Fragment_2
KQAILHEEGKMDDGLDFSRSQEEESRTARVIRKCSSLFTKFINGLETLQTNRRHSLFFAS
VNLSEMVMCLEDLINYFAQPDEEMEHEEKQNRFRALRNRQDLFQEEGILNLILEAIDKIN
VITSQGFLAGFLAGDDAGQSWEMISGYLYQLLAAIIKGNHTNCAQFANSNRLNWLFSRLG
SQASSEGTGMLDVLHCVLIDSPEALNMMRDEHIKVIISLLEKHGRDPKVLDVLCSLCVGN
GVAVRSSQNNICDFLLPGKNLLLQTQLVDHVASIRPNIFVGKVEGSAVYQKWYFEVTMDH
LEQTTHMMPHLRIGWANTTGYVPYP
>Locus_10403_Transcript_2/3_Confidence_0.333_Length_2537|.|sp|
Q24498|RY44_DROME Ryanodine receptor 44F OS=Drosophila melanogaster
GN=Rya-r44F PE=1 SV=3|e=0|from=3|to=2537|frame=-1|Fragment_3
GDDLYSFGFDGAYLWTGGRKSLVLPGA
>Locus_10403_Transcript_2/3_Confidence_0.333_Length_2537|.|sp|
Q24498|RY44_DROME Ryanodine receptor 44F OS=Drosophila melanogaster
GN=Rya-r44F PE=1 SV=3|e=0|from=3|to=2537|frame=-1|Fragment_4
EEPYIRKGDVVGCALDLSIPLITFTMNGMRIHGSFTDFNLDGMFHPVMSCSSKLSCRFLL
GGDHGRLKYAPPEGFSPLVECLQPQQILSLDPCFYFGNLNKSVLAGPWLVEDDTAFVPKP
VDTTSVTMPSYVETIRDKLAENIHEMWALNKVESGWCWGERRDDVHKIHPCLTQFDNLPA
AEKKYDSQLAIQTLKTIIALGYYISMDKPPARIRQIRLPNEPFLQANGYKPAPLDLSAVT
LTPKMDELVDQLAENTHNLWAKERIQQGWTYGLNEDSDNLRSPHLVPYAKVDEAIKKANR
DTASETVRTLLVYGYNLEPPQGEANEALLAEAHRLRHSGFRTYRVEKTYAVTSGKWYFEF
EILTAGPMRVGWARSDCPPGQMLGQDEYTWAFDGYNEEKVFSGNAESFGKQWAVGDVVGV
FLDLIDHTISFSLNGELLMDALGGETSFADVQGEG
>Locus_10403_Transcript_2/3_Confidence_0.333_Length_2537|.|sp|
Q24498|RY44_DROME Ryanodine receptor 44F OS=Drosophila melanogaster
GN=Rya-r44F PE=1 SV=3|e=1.36942E-22|from=408|to=1034|frame=-1|
Fragment_1
DDTAFVPKPVDTTSVTMPSYVETIRDKLAENIHEMWALNKVESGWCWGERRDDVHKIHPC
LTQFDNLPAAEKKYDSQLAIQTLKTIIALGYYIS
>Locus_10403_Transcript_2/3_Confidence_0.333_Length_2537|.|sp|
Q24498|RY44_DROME Ryanodine receptor 44F OS=Drosophila melanogaster
GN=Rya-r44F PE=1 SV=3|e=1.36942E-22|from=408|to=1034|frame=-1|
Fragment_2
MDKPPARIRQIRLPNEPFLQANGYKPAPLDLSAVTLTPKMDELVDQLAENTHNLWAKERI
QQGWTYGLNEDSDNLRSPHLVPYAKVDEAIKKANRDTASETVRTLLVYGYNLEPP
>Locus_10403_Transcript_2/3_Confidence_0.333_Length_2537|.|sp|
Q24498|RY44_DROME Ryanodine receptor 44F OS=Drosophila melanogaster
GN=Rya-r44F PE=1 SV=3|e=2.86205E-12|from=399|to=845|frame=-1|
Fragment_1
FDNLPAAEKKYDSQLAIQTLKTIIALGYYISMDKPPARI
>Locus_10403_Transcript_2/3_Confidence_0.333_Length_2537|.|sp|
Q24498|RY44_DROME Ryanodine receptor 44F OS=Drosophila melanogaster
GN=Rya-r44F PE=1 SV=3|e=2.86205E-12|from=399|to=845|frame=-1|
Fragment_2
```

RQIRLPNEPFLQANGYKPAPLDLSAVTLTPKMDELVDQLAENTHNLWAKERIQQGWTYGL
NEDSDNLRSPHLVPYAKVDEAIKKANRDTASETVRTLLVYGYNLEPPQGE
>Locus_29700_Transcript_1/1_Confidence_1.000_Length_286|.|sp|Q24498|RY44_DROME Ryanodine receptor 44F OS=Drosophila melanogaster GN=Rya-r44F PE=1 SV=3|e=5.87161E-44|from=1|to=285|frame=-2|Fragment_1
GMFVGCFVDTATGCITFTCEGKETSHKWHMEPETKLFPAIFVEATSKDMLQIELGRTATT
LPLSAAVLPTGDKHAIPQFPPRLKVQCLKPHQWAR
>Locus_2665_Transcript_4/20_Confidence_1.000_Length_248|.|sp|Q24498|RY44_DROME Ryanodine receptor 44F OS=Drosophila melanogaster GN=Rya-r44F PE=1 SV=3|e=2.17728E-14|from=3|to=248|frame=-1|Fragment_1
SLSALQEPEPEE
>Locus_2665_Transcript_4/20_Confidence_1.000_Length_248|.|sp|Q24498|RY44_DROME Ryanodine receptor 44F OS=Drosophila melanogaster GN=Rya-r44F PE=1 SV=3|e=2.17728E-14|from=3|to=248|frame=-1|Fragment_2
PAVDESRTGKIKKLFNFINTV
>Locus_2665_Transcript_4/20_Confidence_1.000_Length_248|.|sp|Q24498|RY44_DROME Ryanodine receptor 44F OS=Drosophila melanogaster GN=Rya-r44F PE=1 SV=3|e=2.17728E-14|from=3|to=248|frame=-1|Fragment_3
EIFRKVLISTIVSWAEESQIETPKLVREM
>Locus_10437_Transcript_1/2_Confidence_0.750_Length_2925|UNIGENE REPRESENTATIVE TRANSCRIPT|sp|Q26972|SEM1A_TRICF Semaphorin-1A OS=Tribolium confusum GN=SEMA-1A PE=2 SV=1|e=6.1121E-131|from=20|to=2296|frame=2|Fragment_1
SFNNNLNDKDTKQYFKLLQHETQDSILVGARNAVYNLSLPDLIENKNQRIEWPSSGAHRE
LCYLKGKSEIDCQNYIRIGALDTSNALLVCGTNSFKPLCRRYR
>Locus_10437_Transcript_1/2_Confidence_0.750_Length_2925|UNIGENE REPRESENTATIVE TRANSCRIPT|sp|Q26972|SEM1A_TRICF Semaphorin-1A OS=Tribolium confusum GN=SEMA-1A PE=2 SV=1|e=6.1121E-131|from=20|to=2296|frame=2|Fragment_2
PQTNITASSEAQASKLHEQFEGIGVCPYDPTHNSTAIYADGALFSATAADFSGGDPLIYR
SPQRTERYDLRQLNDPAFVGSVTYGEHVYVFFRETAVEHMNCGKVVYSRVGRICASDRGG
PRHFGDRWTSFVKARLNCSVAGDYPFYFDEVQAVSGPHKGVRAGGVESSVVYLVATTAPS
AIGGSAVCAFDMDHVAKLFDTASFKHQPHGINSNWL
>Locus_10437_Transcript_1/2_Confidence_0.750_Length_2925|UNIGENE REPRESENTATIVE TRANSCRIPT|sp|Q26972|SEM1A_TRICF Semaphorin-1A OS=Tribolium confusum GN=SEMA-1A PE=2 SV=1|e=6.1121E-131|from=20|to=2296|frame=2|Fragment_3
GSCLEDSRTLSDNNVNFIRAHSLLDPAIPGLTRHPLITRVSLSHRMSSIAVQPQIRALND
KTYDMLYIGTDDGRVLQALNTALAVSLNASNPILVSEWQLA
>Locus_10437_Transcript_1/2_Confidence_0.750_Length_2925|UNIGENE REPRESENTATIVE TRANSCRIPT|sp|Q26972|SEM1A_TRICF Semaphorin-1A OS=Tribolium confusum GN=SEMA-1A PE=2 SV=1|e=6.1121E-131|from=20|to=2296|frame=2|Fragment_4
PQGEPIRELRMAGAANNRFLVAVTDERVLAADTRFVCKSASNCRECLQLRNPECAYDRRN
QMCISLQDHPYPNKERFLQSLDPEDYQSVANSDAELCKKSQTWPGVPASRRRPDANKGRD
ETGHSGDGSGENKRISSDE
>Locus_10437_Transcript_1/2_Confidence_0.750_Length_2925|UNIGENE REPRESENTATIVE TRANSCRIPT|sp|Q26972|SEM1A_TRICF Semaphorin-1A OS=Tribolium confusum GN=SEMA-1A PE=2 SV=1|e=6.1121E-131|from=20|to=2296|frame=2|Fragment_5
LDNEIVIELVGSRALA
>Locus_10437_Transcript_1/2_Confidence_0.750_Length_2925|UNIGENE REPRESENTATIVE TRANSCRIPT|sp|Q26972|SEM1A_TRICF Semaphorin-1A OS=Tribolium confusum GN=SEMA-1A PE=2 SV=1|e=6.1121E-131|from=20|

to=2296|frame=2|Fragment_6
IGAVHPDVGAIKYSSGTMHSVIIISSLLTLVCGFALGFLFSRRMR
>Locus_10437_Transcript_1/2_Confidence_0.750_Length_2925|UNIGENE
REPRESENTATIVE TRANSCRIPT|sp|Q26972|SEM1A_TRICF Semaphorin-1A
OS=Tribolium confusum GN=SEMA-1A PE=2 SV=1|e=6.1121E-131|from=20|
to=2296|frame=2|Fragment_7
YHRQGPYGDQHNHRHLESNFSTSAQCSQY
>Locus_10437_Transcript_1/2_Confidence_0.750_Length_2925|UNIGENE
REPRESENTATIVE TRANSCRIPT|sp|Q26972|SEM1A_TRICF Semaphorin-1A
OS=Tribolium confusum GN=SEMA-1A PE=2 SV=1|e=6.1121E-131|from=20|
to=2296|frame=2|Fragment_8
LLKNVPTCEKQDNLQHELAKHHELKNSTESLDRMGTLQKAKKTYI
>Locus_10437_Transcript_2/2_Confidence_0.750_Length_2925|.|sp|
Q26972|SEM1A_TRICF Semaphorin-1A OS=Tribolium confusum GN=SEMA-1A
PE=2 SV=1|e=6.1121E-131|from=20|to=2296|frame=2|Fragment_1
SFNNNLNDKDTKQYFKLLQHETQDSILVGARNAVYNLSLPDLIENKNQRIEWPSSGAHRE
LCYLKGKSEIDCQNYIRIGALDTSNALLVCGTNSFKPLCRRYR
>Locus_10437_Transcript_2/2_Confidence_0.750_Length_2925|.|sp|
Q26972|SEM1A_TRICF Semaphorin-1A OS=Tribolium confusum GN=SEMA-1A
PE=2 SV=1|e=6.1121E-131|from=20|to=2296|frame=2|Fragment_2
PQTNITASSEAQASKLHEQFEGIGVCPYDPTHNSTAIYADGALFSATAADFSGGDPLIYR
SPQRTERYDLRQLNDPAFVGSVTYGEHVYVFFRETAVEHMNCGKVVYSRVGRICASDRGG
PRHFGDRWTSFVKARLNCSVAGDYPFYFDEVQAVSGPHKGVRAGGVESSVVYLVATTAPS
AIGGSAVCAFDMDHVAKLFDTASFKHQPHGINSNWL
>Locus_10437_Transcript_2/2_Confidence_0.750_Length_2925|.|sp|
Q26972|SEM1A_TRICF Semaphorin-1A OS=Tribolium confusum GN=SEMA-1A
PE=2 SV=1|e=6.1121E-131|from=20|to=2296|frame=2|Fragment_3
GSCLEDSRTLSDNNVNFIRAHSLLDPAIPGLTRHPLITRVSLSHRMSSIAVQPQIRALND
KTYDMLYIGTDDGRVLQALNTALAVSLNASNPILVSEWQLA
>Locus_10437_Transcript_2/2_Confidence_0.750_Length_2925|.|sp|
Q26972|SEM1A_TRICF Semaphorin-1A OS=Tribolium confusum GN=SEMA-1A
PE=2 SV=1|e=6.1121E-131|from=20|to=2296|frame=2|Fragment_4
PQGEPIRELRMAGAANNRFLVAVTDERVLAADTRFVCKSASNCRECLQLRNPECAYDRRN
QMCISLQDHPYPNKERFLQSLDPEDYQSVANSDAELCKKSQTWPGVPASRRRPDANKGRD
ETGHSGDGSGENKRISSDE
>Locus_10437_Transcript_2/2_Confidence_0.750_Length_2925|.|sp|
Q26972|SEM1A_TRICF Semaphorin-1A OS=Tribolium confusum GN=SEMA-1A
PE=2 SV=1|e=6.1121E-131|from=20|to=2296|frame=2|Fragment_5
LDNEIVIELVGSRALA
>Locus_10437_Transcript_2/2_Confidence_0.750_Length_2925|.|sp|
Q26972|SEM1A_TRICF Semaphorin-1A OS=Tribolium confusum GN=SEMA-1A
PE=2 SV=1|e=6.1121E-131|from=20|to=2296|frame=2|Fragment_6
IGAVHPDVGAIKYSSGTMHSVIIISSLLTLVCGFALGFLFSRRMR
>Locus_10437_Transcript_2/2_Confidence_0.750_Length_2925|.|sp|
Q26972|SEM1A_TRICF Semaphorin-1A OS=Tribolium confusum GN=SEMA-1A
PE=2 SV=1|e=6.1121E-131|from=20|to=2296|frame=2|Fragment_7
YHRQGPYGDQHNHRHLESNFSTSAQCSQY
>Locus_10437_Transcript_2/2_Confidence_0.750_Length_2925|.|sp|
Q26972|SEM1A_TRICF Semaphorin-1A OS=Tribolium confusum GN=SEMA-1A
PE=2 SV=1|e=6.1121E-131|from=20|to=2296|frame=2|Fragment_8
LLKNVPTCEKQDNLQHELAKHHELKNSTESLDRMGTLQKAKKTYI
>Locus_4048_Transcript_2/6_Confidence_0.625_Length_1355|UNIGENE
REPRESENTATIVE TRANSCRIPT|sp|Q27333|NT56_DROME Lethal(2)neighbour of
tid protein OS=Drosophila melanogaster GN=l(2)not PE=1 SV=1|

e=7.05245E-86|from=426|to=1355|frame=-1|Fragment_1
NDPIAILFLYLSLNLYMDSKWTLASVALSLAVSIKMN
>Locus_4048_Transcript_2/6_Confidence_0.625_Length_1355|UNIGENE
REPRESENTATIVE TRANSCRIPT|sp|Q27333|NT56_DROME Lethal(2)neighbour of
tid protein OS=Drosophila melanogaster GN=l(2)not PE=1 SV=1|
e=7.05245E-86|from=426|to=1355|frame=-1|Fragment_2
IVNLGLVKTIVQLSICGIIQLILGLPFLLVNAVAYIKGSFDFGRVFEHKWTVNYRFLHRD
IFESGKFHIS
>Locus_4048_Transcript_2/6_Confidence_0.625_Length_1355|UNIGENE
REPRESENTATIVE TRANSCRIPT|sp|Q27333|NT56_DROME Lethal(2)neighbour of
tid protein OS=Drosophila melanogaster GN=l(2)not PE=1 SV=1|
e=7.05245E-86|from=426|to=1355|frame=-1|Fragment_3
LAAFFPISFKYMQSYVRLRTVQKQFEPQIN
>Locus_4048_Transcript_2/6_Confidence_0.625_Length_1355|UNIGENE
REPRESENTATIVE TRANSCRIPT|sp|Q27333|NT56_DROME Lethal(2)neighbour of
tid protein OS=Drosophila melanogaster GN=l(2)not PE=1 SV=1|
e=7.05245E-86|from=426|to=1355|frame=-1|Fragment_4
A
>Locus_4048_Transcript_2/6_Confidence_0.625_Length_1355|UNIGENE
REPRESENTATIVE TRANSCRIPT|sp|Q27333|NT56_DROME Lethal(2)neighbour of
tid protein OS=Drosophila melanogaster GN=l(2)not PE=1 SV=1|
e=7.05245E-86|from=426|to=1355|frame=-1|Fragment_5
EPLTAEQHKFLKDFEKGINKTSTPNNRAVTVEPKQDDNVKTFAVHFDSVTQLALLPIFLC
NFIGVACSRSLHYQFYVWYFHSLPHLLWCTTYSIKVRLLILALIEYSWNTYPSTDLSSCV
LHLCHVAVIWGIYKNI
>Locus_4048_Transcript_3/6_Confidence_0.625_Length_1355|.|sp|Q27333|
NT56_DROME Lethal(2)neighbour of tid protein OS=Drosophila
melanogaster GN=l(2)not PE=1 SV=1|e=7.05245E-86|from=426|to=1355|
frame=-1|Fragment_1
NDPIAILFLYLSLNLYMDSKWTLASVALSLAVSIKMN
>Locus_4048_Transcript_3/6_Confidence_0.625_Length_1355|.|sp|Q27333|
NT56_DROME Lethal(2)neighbour of tid protein OS=Drosophila
melanogaster GN=l(2)not PE=1 SV=1|e=7.05245E-86|from=426|to=1355|
frame=-1|Fragment_2
IVNLGLVKTIVQLSICGIIQLILGLPFLLVNAVAYIKGSFDFGRVFEHKWTVNYRFLHRD
IFESGKFHIS
>Locus_4048_Transcript_3/6_Confidence_0.625_Length_1355|.|sp|Q27333|
NT56_DROME Lethal(2)neighbour of tid protein OS=Drosophila
melanogaster GN=l(2)not PE=1 SV=1|e=7.05245E-86|from=426|to=1355|
frame=-1|Fragment_3
LAAFFPISFKYMQSYVRLRTVQKQFEPQIN
>Locus_4048_Transcript_3/6_Confidence_0.625_Length_1355|.|sp|Q27333|
NT56_DROME Lethal(2)neighbour of tid protein OS=Drosophila
melanogaster GN=l(2)not PE=1 SV=1|e=7.05245E-86|from=426|to=1355|
frame=-1|Fragment_4
A
>Locus_4048_Transcript_3/6_Confidence_0.625_Length_1355|.|sp|Q27333|
NT56_DROME Lethal(2)neighbour of tid protein OS=Drosophila
melanogaster GN=l(2)not PE=1 SV=1|e=7.05245E-86|from=426|to=1355|
frame=-1|Fragment_5
EPLTAEQHKFLKDFEKGINKTSTPNNRAVTVEPKQDDNVKTFAVHFDSVTQLALLPIFLC
NFIGVACSRSLHYQFYVWYFHSLPHLLWCTTYSIKVRLLILALIEYSWNTYPSTDLSSCV
LHLCHVAVIWGIYKNI
>Locus_4048_Transcript_5/6_Confidence_0.625_Length_1355|.|sp|Q27333|

NT56_DROME Lethal(2)neighbour of tid protein OS=Drosophila melanogaster GN=l(2)not PE=1 SV=1|e=7.05245E-86|from=426|to=1355|frame=-1|Fragment_1
NDPIAILFLYLSLNLYMDSKWTLASVALSLAVSIKMN
>Locus_4048_Transcript_5/6_Confidence_0.625_Length_1355|.|sp|Q27333|NT56_DROME Lethal(2)neighbour of tid protein OS=Drosophila melanogaster GN=l(2)not PE=1 SV=1|e=7.05245E-86|from=426|to=1355|frame=-1|Fragment_2
IVNLGLVKTIVQLSICGIIQLILGLPFLLVNAVAYIKGSFDFGRVFEHKWTVNYRFLHRD
IFESGKFHIS
>Locus_4048_Transcript_5/6_Confidence_0.625_Length_1355|.|sp|Q27333|NT56_DROME Lethal(2)neighbour of tid protein OS=Drosophila melanogaster GN=l(2)not PE=1 SV=1|e=7.05245E-86|from=426|to=1355|frame=-1|Fragment_3
LAAFFPISFKYMQSYVRLRTVQKQFEPQIN
>Locus_4048_Transcript_5/6_Confidence_0.625_Length_1355|.|sp|Q27333|NT56_DROME Lethal(2)neighbour of tid protein OS=Drosophila melanogaster GN=l(2)not PE=1 SV=1|e=7.05245E-86|from=426|to=1355|frame=-1|Fragment_4
A
>Locus_4048_Transcript_5/6_Confidence_0.625_Length_1355|.|sp|Q27333|NT56_DROME Lethal(2)neighbour of tid protein OS=Drosophila melanogaster GN=l(2)not PE=1 SV=1|e=7.05245E-86|from=426|to=1355|frame=-1|Fragment_5
EPLTAEQHKFLKDFEKGINKTSTPNNRAVTVEPKQDDNVKTFAVHFDSVTQLALLPIFLC
NFIGVACSRSLHYQFYVWYFHSLPHLLWCTTYSIKVRLLILALIEYSWNTYPSTDLSSCV
LHLCHVAVIWGIYKNI
>Locus_4048_Transcript_6/6_Confidence_0.688_Length_1355|.|sp|Q27333|NT56_DROME Lethal(2)neighbour of tid protein OS=Drosophila melanogaster GN=l(2)not PE=1 SV=1|e=7.05245E-86|from=426|to=1355|frame=-1|Fragment_1
NDPIAILFLYLSLNLYMDSKWTLASVALSLAVSIKMN
>Locus_4048_Transcript_6/6_Confidence_0.688_Length_1355|.|sp|Q27333|NT56_DROME Lethal(2)neighbour of tid protein OS=Drosophila melanogaster GN=l(2)not PE=1 SV=1|e=7.05245E-86|from=426|to=1355|frame=-1|Fragment_2
IVNLGLVKTIVQLSICGIIQLILGLPFLLVNAVAYIKGSFDFGRVFEHKWTVNYRFLHRD
IFESGKFHIS
>Locus_4048_Transcript_6/6_Confidence_0.688_Length_1355|.|sp|Q27333|NT56_DROME Lethal(2)neighbour of tid protein OS=Drosophila melanogaster GN=l(2)not PE=1 SV=1|e=7.05245E-86|from=426|to=1355|frame=-1|Fragment_3
LAAFFPISFKYMQSYVRLRTVQKQFEPQIN
>Locus_4048_Transcript_6/6_Confidence_0.688_Length_1355|.|sp|Q27333|NT56_DROME Lethal(2)neighbour of tid protein OS=Drosophila melanogaster GN=l(2)not PE=1 SV=1|e=7.05245E-86|from=426|to=1355|frame=-1|Fragment_4
A
>Locus_4048_Transcript_6/6_Confidence_0.688_Length_1355|.|sp|Q27333|NT56_DROME Lethal(2)neighbour of tid protein OS=Drosophila melanogaster GN=l(2)not PE=1 SV=1|e=7.05245E-86|from=426|to=1355|frame=-1|Fragment_5
EPLTAEQHKFLKDFEKGINKTSTPNNRAVTVEPKQDDNVKTFAVHFDSVTQLALLPIFLC
NFIGVACSRSLHYQFYVWYFHSLPHLLWCTTYSIKVRLLILALIEYSWNTYPSTDLSSCV

LHLCHVAVIWGIYKNI
>Locus_4048_Transcript_1/6_Confidence_0.688_Length_1355|.|sp|Q27333|
NT56_DROME Lethal(2)neighbour of tid protein OS=Drosophila
melanogaster GN=l(2)not PE=1 SV=1|e=9.21079E-86|from=426|to=1355|
frame=-1|Fragment_1
NDPIAILFLYLSLNLYMDSKWTLASVALSLAVSIKMN
>Locus_4048_Transcript_1/6_Confidence_0.688_Length_1355|.|sp|Q27333|
NT56_DROME Lethal(2)neighbour of tid protein OS=Drosophila
melanogaster GN=l(2)not PE=1 SV=1|e=9.21079E-86|from=426|to=1355|
frame=-1|Fragment_2
IVNLGLVKTIVQLSICGIIQLILGLPFLLVNAVAYIKGSFDFGRVFEHKWTVNYRFLHRD
IFESGKFHIS
>Locus_4048_Transcript_1/6_Confidence_0.688_Length_1355|.|sp|Q27333|
NT56_DROME Lethal(2)neighbour of tid protein OS=Drosophila
melanogaster GN=l(2)not PE=1 SV=1|e=9.21079E-86|from=426|to=1355|
frame=-1|Fragment_3
LAAFFPISFKYMQSYVRLRTVQKQFEPQIN
>Locus_4048_Transcript_1/6_Confidence_0.688_Length_1355|.|sp|Q27333|
NT56_DROME Lethal(2)neighbour of tid protein OS=Drosophila
melanogaster GN=l(2)not PE=1 SV=1|e=9.21079E-86|from=426|to=1355|
frame=-1|Fragment_4
A
>Locus_4048_Transcript_1/6_Confidence_0.688_Length_1355|.|sp|Q27333|
NT56_DROME Lethal(2)neighbour of tid protein OS=Drosophila
melanogaster GN=l(2)not PE=1 SV=1|e=9.21079E-86|from=426|to=1355|
frame=-1|Fragment_5
EPLTAEQHKFLKDFEKGINKTSTPNNRAVTVEPKQDDNVKTFAVHFDSVTQLALLPIFLC
NFIGVACSRSLHYQFYVWYFHSLPHLLWCTTYSIKVRLLILALIEYSWNTYPSTDLSSCV
LHLCHVAVIWGIYKNV
>Locus_4048_Transcript_4/6_Confidence_0.625_Length_1355|.|sp|Q27333|
NT56_DROME Lethal(2)neighbour of tid protein OS=Drosophila
melanogaster GN=l(2)not PE=1 SV=1|e=3.50009E-85|from=426|to=1355|
frame=-1|Fragment_1
NDPIAILFLYLSLNLYMDSKWTLASVALSLAVSIKMN
>Locus_4048_Transcript_4/6_Confidence_0.625_Length_1355|.|sp|Q27333|
NT56_DROME Lethal(2)neighbour of tid protein OS=Drosophila
melanogaster GN=l(2)not PE=1 SV=1|e=3.50009E-85|from=426|to=1355|
frame=-1|Fragment_2
IVNLGLVKTIVQLSICGIIQLILGLPFLLVNAVAYIKGSFDFGRVFEHKWTVNYRFLHRD
IFESGKFHIS
>Locus_4048_Transcript_4/6_Confidence_0.625_Length_1355|.|sp|Q27333|
NT56_DROME Lethal(2)neighbour of tid protein OS=Drosophila
melanogaster GN=l(2)not PE=1 SV=1|e=3.50009E-85|from=426|to=1355|
frame=-1|Fragment_3
LAAFFPISFKYMQSYVRLRTVQKQFEPQIN
>Locus_4048_Transcript_4/6_Confidence_0.625_Length_1355|.|sp|Q27333|
NT56_DROME Lethal(2)neighbour of tid protein OS=Drosophila
melanogaster GN=l(2)not PE=1 SV=1|e=3.50009E-85|from=426|to=1355|
frame=-1|Fragment_4
A
>Locus_4048_Transcript_4/6_Confidence_0.625_Length_1355|.|sp|Q27333|
NT56_DROME Lethal(2)neighbour of tid protein OS=Drosophila
melanogaster GN=l(2)not PE=1 SV=1|e=3.50009E-85|from=426|to=1355|
frame=-1|Fragment_5

EPLTAEQHKFLKDFEKGINKTSTPNNRAVTVEPKQDDNVKTFAVHFDSVIQLALLPIFLC
NFIGVACSRSLHYQFYVWYFHSLPHLLWCTTYSIKVRLLILALIEYSWNTYPSTDLSSCV
LHLCHVAVIWGIYKNI
>Locus_1899_Transcript_2/4_Confidence_0.700_Length_818|.|sp|Q27333|NT56_DROME Lethal(2)neighbour of tid protein OS=Drosophila melanogaster GN=l(2)not PE=1 SV=1|e=1.12118E-68|from=258|to=818|frame=3|Fragment_1
YLSLQFGKELLTSSEKLPIISFLILAVELVFNIFIINTRKYTEIDWIAYMQECEGFLNGT
LDYSQLRGDTGPLVYPAGFVYIYSILYYITAAGKDIYVGQCIFIGIYLCQMALVLRLYTK
SRKVPPYVLILCALTSYRIHSIYMLRLFNDPIAILFLYLSLNLYMDSKWTLASVALSLAV
SIKMNIL
>Locus_1899_Transcript_1/4_Confidence_0.600_Length_818|.|sp|Q27333|NT56_DROME Lethal(2)neighbour of tid protein OS=Drosophila melanogaster GN=l(2)not PE=1 SV=1|e=1.91244E-68|from=258|to=818|frame=3|Fragment_1
YLSFQFGKELLTSPEKLTIISFLILAVELVFNIFIINTRKYTEIDWIAYMQECEGFLNGT
LDYSQLRGDTGPLVYPAGFVYIYSILYYITAAGKDIYVGQCIFIGIYLCQMALVLRLYTK
SRKVPPYVLILCALTSYRIHSIYMLRLFNDPIAILFLYLSLNLYMDSKWTLASVALSLAV
SIKMNIL
>Locus_1899_Transcript_3/4_Confidence_0.600_Length_818|.|sp|Q27333|NT56_DROME Lethal(2)neighbour of tid protein OS=Drosophila melanogaster GN=l(2)not PE=1 SV=1|e=1.91244E-68|from=258|to=818|frame=3|Fragment_1
YLSFQFGKELLTSPEKLTIISFLILAVELVFNIFIINTRKYTEIDWIAYMQECEGFLNGT
LDYSQLRGDTGPLVYPAGFVYIYSILYYITAAGKDIYVGQCIFIGIYLCQMALVLRLYTK
SRKVPPYVLILCALTSYRIHSIYMLRLFNDPIAILFLYLSLNLYMDSKWTLASVALSLAV
SIKMNIL
>Locus_1899_Transcript_4/4_Confidence_0.700_Length_818|.|sp|Q27333|NT56_DROME Lethal(2)neighbour of tid protein OS=Drosophila melanogaster GN=l(2)not PE=1 SV=1|e=1.91244E-68|from=258|to=818|frame=3|Fragment_1
YLSFQFGKELLTSPEKLTIISFLILAVELVFNIFIINTRKYTEIDWIAYMQECEGFLNGT
LDYSQLRGDTGPLVYPAGFVYIYSILYYITAAGKDIYVGQCIFIGIYLCQMALVLRLYTK
SRKVPPYVLILCALTSYRIHSIYMLRLFNDPIAILFLYLSLNLYMDSKWTLASVALSLAV
SIKMNIL
>Locus_9676_Transcript_1/2_Confidence_0.750_Length_569|UNIGENE REPRESENTATIVE TRANSCRIPT|sp|Q27367|CRQ_DROME Protein croquemort OS=Drosophila melanogaster GN=crq PE=1 SV=2|e=3.82172E-44|from=16|to=564|frame=1|Fragment_1
CSLRCRRCLLFSCSGIFVILALVCGLLWPMIADPIMRSQLVLQPGSKNFEMWKETPIPIY
LELYLFNWTNPDDINNHDIKPHFEEMGPYVFHEKHVRVDLNWNQNSTITFKQKRIWNFMP
DMSKGSLDDMITNLNVIVSTVAYTVRNKHYLIQKAVDLLMREKREKLVITKSVRELLFDG
YQD
>Locus_9676_Transcript_2/2_Confidence_0.750_Length_569|.|sp|Q27367|CRQ_DROME Protein croquemort OS=Drosophila melanogaster GN=crq PE=1 SV=2|e=3.82172E-44|from=16|to=564|frame=1|Fragment_1
CSLRCRRCLLFSCSGIFVILALVCGLLWPMIADPIMRSQLVLQPGSKNFEMWKETPIPIY
LELYLFNWTNPDDINNHDIKPHFEEMGPYVFHEKHVRVDLNWNQNSTITFKQKRIWNFMP
DMSKGSLDDMITNLNVIVSTVAYTVRNKHYLIQKAVDLLMREKREKLVITKSVRELLFDG
YQD
>Locus_8114_Transcript_1/1_Confidence_1.000_Length_396|UNIGENE REPRESENTATIVE TRANSCRIPT|sp|Q4LBB6|OCTB3_DROME Octopamine receptor beta-3R OS=Drosophila melanogaster GN=Octbeta3R PE=2 SV=4|e=5.47743E-21|from=1|to=180|frame=1|Fragment_1

CPCPDVVVATLFWIGYFNSALNPLIYAYFNRDFREAFKNTLKCVFPCAVRQKDSSHLHYV
>Locus_6430_Transcript_1/2_Confidence_0.667_Length_2629|UNIGENE REPRESENTATIVE TRANSCRIPT|sp|Q7KRW1|TRC8_DROME Protein TRC8 homolog OS=Drosophila melanogaster GN=Trc8 PE=1 SV=1|e=0|from=600|to=2402|frame=-3|Fragment_1
MSVRTKILGFVDVILRVPPLFLLDEIFKIGLGLPL
>Locus_6430_Transcript_1/2_Confidence_0.667_Length_2629|UNIGENE REPRESENTATIVE TRANSCRIPT|sp|Q7KRW1|TRC8_DROME Protein TRC8 homolog OS=Drosophila melanogaster GN=Trc8 PE=1 SV=1|e=0|from=600|to=2402|frame=-3|Fragment_2
ANEEPYLDLNVTKVILTDAEQ
>Locus_6430_Transcript_1/2_Confidence_0.667_Length_2629|UNIGENE REPRESENTATIVE TRANSCRIPT|sp|Q7KRW1|TRC8_DROME Protein TRC8 homolog OS=Drosophila melanogaster GN=Trc8 PE=1 SV=1|e=0|from=600|to=2402|frame=-3|Fragment_3
ITYDNDFYRVLLTTACKFIVCCLGCISALCIFTLWTKHLVVVYMYLLSIAIIVLSYLGNV
SLIKALVDKPSLVEMVIHSNYSVLLETEGPVFQLCSHLLGQLIMGFSFQYVHMGPRYSVM
QKLLPYTFLFPLTIALFPIPRELLKHVAMIATLVPLALVKIALWVSAFDVVETLYGGFQH
ARNYASNYGLSALVESEWQRLNVPCVLRAFWMIRLFE
>Locus_6430_Transcript_1/2_Confidence_0.667_Length_2629|UNIGENE REPRESENTATIVE TRANSCRIPT|sp|Q7KRW1|TRC8_DROME Protein TRC8 homolog OS=Drosophila melanogaster GN=Trc8 PE=1 SV=1|e=0|from=600|to=2402|frame=-3|Fragment_4
QLSNIIEENNITFMGAVQTLLVCGCETVTAVLGMTSVVSLISHYIGKFFQMFLLTDDDED
KSMATVSAIVFYILALQTGLTSLSPEKRFVRLCRNLCLLVTALLHYIHNNVSPLLMSLSA
ARNPSRNRHIR
>Locus_6430_Transcript_1/2_Confidence_0.667_Length_2629|UNIGENE REPRESENTATIVE TRANSCRIPT|sp|Q7KRW1|TRC8_DROME Protein TRC8 homolog OS=Drosophila melanogaster GN=Trc8 PE=1 SV=1|e=0|from=600|to=2402|frame=-3|Fragment_5
LWSRHPTSTWLLAVTAFSIELIVKVLVSLATYTLFLLDARRTTFWEPLDDYVYYIRGFGN
GVEFLFGIFLFFNGAWILIFES
>Locus_6430_Transcript_1/2_Confidence_0.667_Length_2629|UNIGENE REPRESENTATIVE TRANSCRIPT|sp|Q7KRW1|TRC8_DROME Protein TRC8 homolog OS=Drosophila melanogaster GN=Trc8 PE=1 SV=1|e=0|from=600|to=2402|frame=-3|Fragment_6
GGAIRAVMMCVHAYFNIWCEARAGWGVFMKRRTAVHKIRSLPEATKEDLEQLADVCAICY
QEMTAAKITRCRHFFHGVCLRKWLYVQDSCPLCHELL
>Locus_7677_Transcript_2/2_Confidence_0.667_Length_1080|UNIGENE REPRESENTATIVE TRANSCRIPT|sp|Q7KT91|C3390_DROME Non-lysosomal glucosylceramidase OS=Drosophila melanogaster GN=CG33090 PE=1 SV=1|e=1.3632E-102|from=5|to=1045|frame=2|Fragment_1
ENGKR
>Locus_7677_Transcript_2/2_Confidence_0.667_Length_1080|UNIGENE REPRESENTATIVE TRANSCRIPT|sp|Q7KT91|C3390_DROME Non-lysosomal glucosylceramidase OS=Drosophila melanogaster GN=CG33090 PE=1 SV=1|e=1.3632E-102|from=5|to=1045|frame=2|Fragment_2
EFCRFQLRPGKYEYNTVIANQFIINIKDSNKKTIFQSVLS
>Locus_7677_Transcript_2/2_Confidence_0.667_Length_1080|UNIGENE REPRESENTATIVE TRANSCRIPT|sp|Q7KT91|C3390_DROME Non-lysosomal glucosylceramidase OS=Drosophila melanogaster GN=CG33090 PE=1 SV=1|e=1.3632E-102|from=5|to=1045|frame=2|Fragment_3
TYEKPS
>Locus_7677_Transcript_2/2_Confidence_0.667_Length_1080|UNIGENE

REPRESENTATIVE TRANSCRIPT|sp|Q7KT91|C3390_DROME Non-lysosomal glucosylceramidase OS=Drosophila melanogaster GN=CG33090 PE=1 SV=1|
e=1.3632E-102|from=5|to=1045|frame=2|Fragment_4
KPLECWQWQLNPDKCSYTGLYPRSWTEYDLSEYGIKLTCRQVSPVIPHEYKDSCLPCAVF
VWSVDNICSEERTVSITFSFKNGTGTAKQDAEGNASSFKVSNENITSVCISQAIDNMICT
YNLSTRNNVKNIQVSTCDKFDPCGNGGKFWQELYENGCFTQRIDDGAFKGKDVGVAICAQ
SKIAPNNSCDFEFALSWDMPSIHFMGK
>Locus_7677_Transcript_2/2_Confidence_0.667_Length_1080|UNIGENE
REPRESENTATIVE TRANSCRIPT|sp|Q7KT91|C3390_DROME Non-lysosomal
glucosylceramidase OS=Drosophila melanogaster GN=CG33090 PE=1 SV=1|
e=1.3632E-102|from=5|to=1045|frame=2|Fragment_5
NLYKRYYTKYFEGK
>Locus_7677_Transcript_2/2_Confidence_0.667_Length_1080|UNIGENE
REPRESENTATIVE TRANSCRIPT|sp|Q7KT91|C3390_DROME Non-lysosomal
glucosylceramidase OS=Drosophila melanogaster GN=CG33090 PE=1 SV=1|
e=1.3632E-102|from=5|to=1045|frame=2|Fragment_6
NAGILIGEHALTNYKMWEEQIYNWQSPILNDSELPDWYKGSLFNELYYVSDGGTV
>Locus_7677_Transcript_1/2_Confidence_0.667_Length_942|.|sp|Q7KT91|
C3390_DROME Non-lysosomal glucosylceramidase OS=Drosophila
melanogaster GN=CG33090 PE=1 SV=1|e=4.27044E-86|from=5|to=934|
frame=2|Fragment_1
ENGKR
>Locus_7677_Transcript_1/2_Confidence_0.667_Length_942|.|sp|Q7KT91|
C3390_DROME Non-lysosomal glucosylceramidase OS=Drosophila
melanogaster GN=CG33090 PE=1 SV=1|e=4.27044E-86|from=5|to=934|
frame=2|Fragment_2
EFCRFQLRPGKYEYNTVIANQFIINIKDSNKKTIFQSVLS
>Locus_7677_Transcript_1/2_Confidence_0.667_Length_942|.|sp|Q7KT91|
C3390_DROME Non-lysosomal glucosylceramidase OS=Drosophila
melanogaster GN=CG33090 PE=1 SV=1|e=4.27044E-86|from=5|to=934|
frame=2|Fragment_3
TYEKPS
>Locus_7677_Transcript_1/2_Confidence_0.667_Length_942|.|sp|Q7KT91|
C3390_DROME Non-lysosomal glucosylceramidase OS=Drosophila
melanogaster GN=CG33090 PE=1 SV=1|e=4.27044E-86|from=5|to=934|
frame=2|Fragment_4
KPLECWQWQLNPDKCSYTGLYPRSWTEYDLSEYGIKLTCRQVSPVIPHEYKDSCLPCAVF
VWSVDNICSEERTVSITFSFKNGTGTAKQDAEGNASSFKVSNENITSVCISQAIDNMICT
YNLSTRNNVKNIQVSTCDKFDPCGNGGKFWQELYENGCFTQRIDDGVFKGKDVGVAICAQ
SKIAPNNSCDFEFALSWDMPSIHFMGK
>Locus_7677_Transcript_1/2_Confidence_0.667_Length_942|.|sp|Q7KT91|
C3390_DROME Non-lysosomal glucosylceramidase OS=Drosophila
melanogaster GN=CG33090 PE=1 SV=1|e=4.27044E-86|from=5|to=934|
frame=2|Fragment_5
NLYKRYYTKYFEGK
>Locus_7677_Transcript_1/2_Confidence_0.667_Length_942|.|sp|Q7KT91|
C3390_DROME Non-lysosomal glucosylceramidase OS=Drosophila
melanogaster GN=CG33090 PE=1 SV=1|e=4.27044E-86|from=5|to=934|
frame=2|Fragment_6
NAGILIGEHALTNYKMWE
>Locus_4025_Transcript_2/7_Confidence_0.312_Length_368|UNIGENE
REPRESENTATIVE TRANSCRIPT|sp|Q7KVA1|XYLT_DROME Xylosyltransferase
oxt OS=Drosophila melanogaster GN=oxt PE=2 SV=1|e=8.38441E-43|
from=1|to=363|frame=1|Fragment_1

NMKCPGDPKEICGGFFKMNIYETGISKFSPQTAVTLTTNDTEPKVRIVYLLTLNGRAVRQ
VYRLIKALFHVDHYFYIHVDSRQDYMFRQLLQLENNFPNIRLSRNRFSTIWGGASLLQML
L
>Locus_167_Transcript_1/1_Confidence_1.000_Length_302|.|sp|Q7KVA1|
XYLT_DROME Xylosyltransferase oxt OS=Drosophila melanogaster GN=oxt
PE=2 SV=1|e=1.47479E-39|from=3|to=302|frame=-1|Fragment_1
LTANKNKNFVKSHGREVQRFIQKQGLDKTFIECDTHMWRTGDRTLPWGIQIDGGSDWITL
TREFVKYIT
>Locus_167_Transcript_1/1_Confidence_1.000_Length_302|.|sp|Q7KVA1|
XYLT_DROME Xylosyltransferase oxt OS=Drosophila melanogaster GN=oxt
PE=2 SV=1|e=1.47479E-39|from=3|to=302|frame=-1|Fragment_2
QEQDALVKGLMTVFRHTLLPAESFFHTVLRN
>Locus_26648_Transcript_1/1_Confidence_1.000_Length_302|.|sp|Q7KVA1|
XYLT_DROME Xylosyltransferase oxt OS=Drosophila melanogaster GN=oxt
PE=2 SV=1|e=1.47479E-39|from=1|to=300|frame=1|Fragment_1
LTANKNKNFVKSHGREVQRFIQKQGLDKTFIECDTHMWRTGDRTLPWGIQIDGGSDWITL
TRDFVKYIT
>Locus_26648_Transcript_1/1_Confidence_1.000_Length_302|.|sp|Q7KVA1|
XYLT_DROME Xylosyltransferase oxt OS=Drosophila melanogaster GN=oxt
PE=2 SV=1|e=1.47479E-39|from=1|to=300|frame=1|Fragment_2
QEQDALVKGLMTVFRHTLLPAESFFHTVLRN
>Locus_8527_Transcript_1/1_Confidence_1.000_Length_299|.|sp|Q7KVA1|
XYLT_DROME Xylosyltransferase oxt OS=Drosophila melanogaster GN=oxt
PE=2 SV=1|e=1.58034E-41|from=1|to=297|frame=1|Fragment_1
FPNIRLSRNRFSTIWGGASLLQMLLASMNELLKSDWKWDFIINLSESDFPIKSNERLVQF
LTANKNKNFVKSHGREVQRFIQKQGLDKTFIECDTHMWR
>Locus_18073_Transcript_1/1_Confidence_1.000_Length_299|.|sp|Q7KVA1|
XYLT_DROME Xylosyltransferase oxt OS=Drosophila melanogaster GN=oxt
PE=2 SV=1|e=1.58034E-41|from=3|to=299|frame=-1|Fragment_1
FPNIRLSRNRFSTIWGGASLLQMLLASMNELLKSDWKWDFIINLSESDFPIKSNERLVQF
LTANKNKNFVKSHGREVQRFIQKQGLDKTFIECDTHMWR
>Locus_4025_Transcript_1/7_Confidence_0.188_Length_245|.|sp|Q7KVA1|
XYLT_DROME Xylosyltransferase oxt OS=Drosophila melanogaster GN=oxt
PE=2 SV=1|e=4.53162E-28|from=7|to=240|frame=1|Fragment_1
KVRIVYLLTLNGRAVRQVYRLIKALFHVDHYFYIHVDSRQDYMFRQLLQLENNFPNIRLS
RNRFSTIWGGASLLQMLL
>Locus_23234_Transcript_1/1_Confidence_1.000_Length_527|UNIGENE
REPRESENTATIVE TRANSCRIPT|sp|Q7KVW5|KCNN_DROME Small conductance
calcium-activated potassium channel protein OS=Drosophila
melanogaster GN=SK PE=2 SV=2|e=2.26533E-13|from=4|to=471|frame=1|
Fragment_1
PPVLLTAA
>Locus_23234_Transcript_1/1_Confidence_1.000_Length_527|UNIGENE
REPRESENTATIVE TRANSCRIPT|sp|Q7KVW5|KCNN_DROME Small conductance
calcium-activated potassium channel protein OS=Drosophila
melanogaster GN=SK PE=2 SV=2|e=2.26533E-13|from=4|to=471|frame=1|
Fragment_2
GGRIVRQSSQPE
>Locus_23234_Transcript_1/1_Confidence_1.000_Length_527|UNIGENE
REPRESENTATIVE TRANSCRIPT|sp|Q7KVW5|KCNN_DROME Small conductance
calcium-activated potassium channel protein OS=Drosophila
melanogaster GN=SK PE=2 SV=2|e=2.26533E-13|from=4|to=471|frame=1|
Fragment_3
PSVSLRQLRDP

>Locus_23234_Transcript_1/1_Confidence_1.000_Length_527|UNIGENE REPRESENTATIVE TRANSCRIPT|sp|Q7KVW5|KCNN_DROME Small conductance calcium-activated potassium channel protein OS=Drosophila melanogaster GN=SK PE=2 SV=2|e=2.26533E-13|from=4|to=471|frame=1|Fragment_4
SLRINGAMRQFKQLRKPVSTLSIPGAMRASGCLRDGSGQ
>Locus_23234_Transcript_1/1_Confidence_1.000_Length_527|UNIGENE REPRESENTATIVE TRANSCRIPT|sp|Q7KVW5|KCNN_DROME Small conductance calcium-activated potassium channel protein OS=Drosophila melanogaster GN=SK PE=2 SV=2|e=2.26533E-13|from=4|to=471|frame=1|Fragment_5
DEAGVALVGVHSEYPR
>Locus_23234_Transcript_1/1_Confidence_1.000_Length_527|UNIGENE REPRESENTATIVE TRANSCRIPT|sp|Q7KVW5|KCNN_DROME Small conductance calcium-activated potassium channel protein OS=Drosophila melanogaster GN=SK PE=2 SV=2|e=2.26533E-13|from=4|to=471|frame=1|Fragment_6
FMEER
>Locus_4771_Transcript_2/3_Confidence_0.667_Length_952|UNIGENE REPRESENTATIVE TRANSCRIPT|sp|Q8IQC1|TVP23_DROME Uncharacterized Golgi apparatus membrane protein-like protein CG5021 OS=Drosophila melanogaster GN=CG5021 PE=2 SV=2|e=1.40421E-76|from=115|to=765|frame=-2|Fragment_1
IGSTMQKEVPLLDDDTIPFGEEDEVN
>Locus_4771_Transcript_2/3_Confidence_0.667_Length_952|UNIGENE REPRESENTATIVE TRANSCRIPT|sp|Q8IQC1|TVP23_DROME Uncharacterized Golgi apparatus membrane protein-like protein CG5021 OS=Drosophila melanogaster GN=CG5021 PE=2 SV=2|e=1.40421E-76|from=115|to=765|frame=-2|Fragment_2
ARNSGKLTHPYVTFFHLFFRCSSIVVYMLCGWFSDSFITSFVCVVLLLSADFWTVKNITG
RLLVGLRWWNYVDDEGVSHWVYEARKG
>Locus_4771_Transcript_2/3_Confidence_0.667_Length_952|UNIGENE REPRESENTATIVE TRANSCRIPT|sp|Q8IQC1|TVP23_DROME Uncharacterized Golgi apparatus membrane protein-like protein CG5021 OS=Drosophila melanogaster GN=CG5021 PE=2 SV=2|e=1.40421E-76|from=115|to=765|frame=-2|Fragment_3
MIQGRVNQRESKIFWLALVLCPVVWAVFFIIALFGFKFKW
>Locus_4771_Transcript_2/3_Confidence_0.667_Length_952|UNIGENE REPRESENTATIVE TRANSCRIPT|sp|Q8IQC1|TVP23_DROME Uncharacterized Golgi apparatus membrane protein-like protein CG5021 OS=Drosophila melanogaster GN=CG5021 PE=2 SV=2|e=1.40421E-76|from=115|to=765|frame=-2|Fragment_4
LHGYIKCNFGASKDLGSVTTEFFKAKLLQNAVSFMNKPA
>Locus_4771_Transcript_2/3_Confidence_0.667_Length_952|UNIGENE REPRESENTATIVE TRANSCRIPT|sp|Q8IQC1|TVP23_DROME Uncharacterized Golgi apparatus membrane protein-like protein CG5021 OS=Drosophila melanogaster GN=CG5021 PE=2 SV=2|e=1.40421E-76|from=115|to=765|frame=-2|Fragment_5
QPSTAQPTGIV
>Locus_4771_Transcript_1/3_Confidence_0.500_Length_940|.|sp|Q8IQC1|TVP23_DROME Uncharacterized Golgi apparatus membrane protein-like protein CG5021 OS=Drosophila melanogaster GN=CG5021 PE=2 SV=2|e=6.8337E-76|from=115|to=753|frame=-2|Fragment_1
IGSTMQKEVPLLDDDTIPFGEEDEVN

>Locus_4771_Transcript_1/3_Confidence_0.500_Length_940|.|sp|Q8IQC1|
TVP23_DROME Uncharacterized Golgi apparatus membrane protein-like
protein CG5021 OS=Drosophila melanogaster GN=CG5021 PE=2 SV=2|
e=6.8337E-76|from=115|to=753|frame=-2|Fragment_2
ARNSGKLTHPYVTFFHLFFRCSSIVVYMLCGWFSDSFITSFVCVVLLLSADFWTVKNITG
RLLVGLRWWNYVDDEGVSHWVYEAR
>Locus_4771_Transcript_1/3_Confidence_0.500_Length_940|.|sp|Q8IQC1|
TVP23_DROME Uncharacterized Golgi apparatus membrane protein-like
protein CG5021 OS=Drosophila melanogaster GN=CG5021 PE=2 SV=2|
e=6.8337E-76|from=115|to=753|frame=-2|Fragment_3
KGRVNQRESKIFWLALVLCPVVWAVFFIIALFGFKFKW
>Locus_4771_Transcript_1/3_Confidence_0.500_Length_940|.|sp|Q8IQC1|
TVP23_DROME Uncharacterized Golgi apparatus membrane protein-like
protein CG5021 OS=Drosophila melanogaster GN=CG5021 PE=2 SV=2|
e=6.8337E-76|from=115|to=753|frame=-2|Fragment_4
LHGYIKCNFGASKDLGSVTTEFFKAKLLQNAVSFMNKPA
>Locus_4771_Transcript_1/3_Confidence_0.500_Length_940|.|sp|Q8IQC1|
TVP23_DROME Uncharacterized Golgi apparatus membrane protein-like
protein CG5021 OS=Drosophila melanogaster GN=CG5021 PE=2 SV=2|
e=6.8337E-76|from=115|to=753|frame=-2|Fragment_5
QPSTAQPTGIV
>Locus_4771_Transcript_3/3_Confidence_0.667_Length_940|.|sp|Q8IQC1|
TVP23_DROME Uncharacterized Golgi apparatus membrane protein-like
protein CG5021 OS=Drosophila melanogaster GN=CG5021 PE=2 SV=2|
e=6.8337E-76|from=115|to=753|frame=-2|Fragment_1
IGSTMQKEVPLLDDDTIPFGEEDEVN
>Locus_4771_Transcript_3/3_Confidence_0.667_Length_940|.|sp|Q8IQC1|
TVP23_DROME Uncharacterized Golgi apparatus membrane protein-like
protein CG5021 OS=Drosophila melanogaster GN=CG5021 PE=2 SV=2|
e=6.8337E-76|from=115|to=753|frame=-2|Fragment_2
ARNSGKLTHPYVTFFHLFFRCSSIVVYMLCGWFSDSFITSFVCVVLLLSADFWTVKNITG
RLLVGLRWWNYVDDEGVSHWVYEAR
>Locus_4771_Transcript_3/3_Confidence_0.667_Length_940|.|sp|Q8IQC1|
TVP23_DROME Uncharacterized Golgi apparatus membrane protein-like
protein CG5021 OS=Drosophila melanogaster GN=CG5021 PE=2 SV=2|
e=6.8337E-76|from=115|to=753|frame=-2|Fragment_3
KGRVNQRESKIFWLALVLCPVVWAVFFIIALFGFKFKW
>Locus_4771_Transcript_3/3_Confidence_0.667_Length_940|.|sp|Q8IQC1|
TVP23_DROME Uncharacterized Golgi apparatus membrane protein-like
protein CG5021 OS=Drosophila melanogaster GN=CG5021 PE=2 SV=2|
e=6.8337E-76|from=115|to=753|frame=-2|Fragment_4
LHGYIKCNFGASKDLGSVTTEFFKAKLLQNAVSFMNKPA
>Locus_4771_Transcript_3/3_Confidence_0.667_Length_940|.|sp|Q8IQC1|
TVP23_DROME Uncharacterized Golgi apparatus membrane protein-like
protein CG5021 OS=Drosophila melanogaster GN=CG5021 PE=2 SV=2|
e=6.8337E-76|from=115|to=753|frame=-2|Fragment_5
QPSTAQPTGIV
>Locus_30807_Transcript_1/1_Confidence_1.000_Length_246|UNIGENE
REPRESENTATIVE TRANSCRIPT|sp|Q8IS44|DRD2L_DROME Dopamine D2-like
receptor OS=Drosophila melanogaster GN=D2R PE=2 SV=1|e=7.00752E-21|
from=3|to=245|frame=-2|Fragment_1
YNYWALILMVFPFLTLFGNVLVIMAVARERTLQTVTNYFIVS
>Locus_30807_Transcript_1/1_Confidence_1.000_Length_246|UNIGENE
REPRESENTATIVE TRANSCRIPT|sp|Q8IS44|DRD2L_DROME Dopamine D2-like

receptor OS=Drosophila melanogaster GN=D2R PE=2 SV=1|e=7.00752E-21|
from=3|to=245|frame=-2|Fragment_2
FNGTWGLPPFVCDFYIAMD
>Locus_7398_Transcript_1/10_Confidence_0.097_Length_1265|UNIGENE
REPRESENTATIVE TRANSCRIPT|sp|Q8MRC9|GALT9_DROME Putative polypeptide
N-acetylgalactosaminyltransferase 9 OS=Drosophila melanogaster
GN=pgant9 PE=2 SV=2|e=2.80626E-65|from=816|to=1265|frame=-1|
Fragment_1
CKPFKWYLDTVFPEMFIPGEAIANGEIRNLGYGGKTCLDSPARRVDMHKAVGLYPCHRQG
GNQYWMLSKSGEIRRDDACLDYAGQDVILYQCHGSKGNQMWTYNDETNQIKHGSSEKCLA
ISETKQKLLMEDCDNAAARQKWTLENYDPS
>Locus_13990_Transcript_1/1_Confidence_1.000_Length_414|UNIGENE
REPRESENTATIVE TRANSCRIPT|sp|Q8MSU3|FRRS1_DROME Putative ferric-
chelate reductase 1 homolog OS=Drosophila melanogaster GN=CG8399
PE=2 SV=1|e=2.24073E-23|from=2|to=412|frame=2|Fragment_1
DDTYKLIDCATP
>Locus_13990_Transcript_1/1_Confidence_1.000_Length_414|UNIGENE
REPRESENTATIVE TRANSCRIPT|sp|Q8MSU3|FRRS1_DROME Putative ferric-
chelate reductase 1 homolog OS=Drosophila melanogaster GN=CG8399
PE=2 SV=1|e=2.24073E-23|from=2|to=412|frame=2|Fragment_2
GSTATHTSTVKKQDLQLEWTAPDNFLGRVIFRATVAQEYAQFWVGLESEVVDVVPRGQGG
>Locus_13990_Transcript_1/1_Confidence_1.000_Length_414|UNIGENE
REPRESENTATIVE TRANSCRIPT|sp|Q8MSU3|FRRS1_DROME Putative ferric-
chelate reductase 1 homolog OS=Drosophila melanogaster GN=CG8399
PE=2 SV=1|e=2.24073E-23|from=2|to=412|frame=2|Fragment_3
QHGGI
>Locus_13990_Transcript_1/1_Confidence_1.000_Length_414|UNIGENE
REPRESENTATIVE TRANSCRIPT|sp|Q8MSU3|FRRS1_DROME Putative ferric-
chelate reductase 1 homolog OS=Drosophila melanogaster GN=CG8399
PE=2 SV=1|e=2.24073E-23|from=2|to=412|frame=2|Fragment_4
ESSSALKNDPFYNGCGSQKTCFGQPEGCVTTKDCKLVMACTVR
>Locus_25853_Transcript_1/1_Confidence_1.000_Length_318|.|sp|Q8MSU3|
FRRS1_DROME Putative ferric-chelate reductase 1 homolog
OS=Drosophila melanogaster GN=CG8399 PE=2 SV=1|e=9.16432E-18|
from=56|to=316|frame=2|Fragment_1
LPQGAPESVCDSMMPFH
>Locus_25853_Transcript_1/1_Confidence_1.000_Length_318|.|sp|Q8MSU3|
FRRS1_DROME Putative ferric-chelate reductase 1 homolog
OS=Drosophila melanogaster GN=CG8399 PE=2 SV=1|e=9.16432E-18|
from=56|to=316|frame=2|Fragment_2
GGGISAQNSQAPFQIFLDSTRTENGQPLRLVL
>Locus_25853_Transcript_1/1_Confidence_1.000_Length_318|.|sp|Q8MSU3|
FRRS1_DROME Putative ferric-chelate reductase 1 homolog
OS=Drosophila melanogaster GN=CG8399 PE=2 SV=1|e=9.16432E-18|
from=56|to=316|frame=2|Fragment_3
GSSQGVPFKGFMIHARRVMPPYDVVGTFTPAGDDTYKL
>Locus_24214_Transcript_1/1_Confidence_1.000_Length_267|.|sp|Q8MSU3|
FRRS1_DROME Putative ferric-chelate reductase 1 homolog
OS=Drosophila melanogaster GN=CG8399 PE=2 SV=1|e=1.53396E-12|
from=57|to=266|frame=-2|Fragment_1
AGDDTYKLIDCATP
>Locus_24214_Transcript_1/1_Confidence_1.000_Length_267|.|sp|Q8MSU3|
FRRS1_DROME Putative ferric-chelate reductase 1 homolog
OS=Drosophila melanogaster GN=CG8399 PE=2 SV=1|e=1.53396E-12|

from=57|to=266|frame=-2|Fragment_2
GSTATHTSTVKKQDLQLEWTAPDNFLGRVIFRATVAQEYAQFWVGLESEVVDVVPR
>Locus_5466_Transcript_1/2_Confidence_0.750_Length_1600|UNIGENE REPRESENTATIVE TRANSCRIPT|sp|Q8MZR5|HYEP2_CTEFE Juvenile hormone epoxide hydrolase 2 OS=Ctenocephalides felis GN=EH2 PE=2 SV=1|e=0|from=107|to=1501|frame=2|Fragment_1
MSNCCRILWIAIVIGLGVLYYEITKEFPKPNIPLDTWWGTGKSQKIDTSMRPFKIAINDE
VLNTLKVKLSDVSFTPPLEGIDFQYGFNTNTLKKLVDFWRTQYNWREREALLNKYPHFKT
NIQGLDIHYVHIKPQVSKNIHVLPMIMVHGWPGSFVEFYKIIPMLTTPRTDYNFVFELIL
PSIPGYGFSQAAAKPGLGATQIAVIMHNLMDRIGFKKYYVQGGDWGSRIVSAMSTLFPEN
VLGHHSNLCFLNTLSSNIKSFVGSLFPEWFAGKQNVHKIYPLSEHFFTLLEESGYFHIQA
TKPDTVGVALRDSPAGLAAYILEKFSTGTNKAWRSAKDGNLQSKFTFTELLDNVMIYYVT
GSITTSMRIYAESYSWDHLSLNMDRVPTIVPTACAKFPHEIAYKTDFQLAEKYKTLLQST
IMPRGGHF
>Locus_5466_Transcript_1/2_Confidence_0.750_Length_1600|UNIGENE REPRESENTATIVE TRANSCRIPT|sp|Q8MZR5|HYEP2_CTEFE Juvenile hormone epoxide hydrolase 2 OS=Ctenocephalides felis GN=EH2 PE=2 SV=1|e=0|from=107|to=1501|frame=2|Fragment_2
DIFSAVKKFIDHHSKKDSKNQENRDL
>Locus_5466_Transcript_2/2_Confidence_0.750_Length_1600|.|sp|Q8MZR5|HYEP2_CTEFE Juvenile hormone epoxide hydrolase 2 OS=Ctenocephalides felis GN=EH2 PE=2 SV=1|e=0|from=107|to=1501|frame=2|Fragment_1
MSNCCRILWIAIVIGLGVLYYEITKEFPKPNIPLDTWWGTGKSQKIDTSMRPFKIAINDE
VLNTLKVKLSDVSFTPPLEGIDFQYGFNTNTLKKLIDFWRTQYNWREREALLNKYPHFKT
NIQGLDIHYVHIKPQVSKNIHVLPMIMVHGWPGSFVEFYKIIPMLTTPRTDYNFVFELIL
PSIPGYGFSQAAAKPGLGATQIAVIMHNLMDRIGFKKYYVQGGDWGSRIVSAMSTLFPEN
VLGHHSNLCFLNTLSSNIKSFVGSLFPEWFAGKQNVHKIYPLSEHFFTLLEESGYFHIQA
TKPDTVGVALRDSPAGLAAYILEKFSTGTNKAWRSAKDGNLQSKFTFTELLDNVMIYYVT
GSITTSMRIYAESYSWDHLSLNMDRVPTIVPTACAKFPHEIAYKTDFQLAEKYKTLLQST
IMPRGGHF
>Locus_5466_Transcript_2/2_Confidence_0.750_Length_1600|.|sp|Q8MZR5|HYEP2_CTEFE Juvenile hormone epoxide hydrolase 2 OS=Ctenocephalides felis GN=EH2 PE=2 SV=1|e=0|from=107|to=1501|frame=2|Fragment_2
DIFSAVKKFIDHHSKKDSKNQENRDL
>Locus_8898_Transcript_1/1_Confidence_1.000_Length_1648|UNIGENE REPRESENTATIVE TRANSCRIPT|sp|Q8MZR6|HYEP1_CTEFE Juvenile hormone epoxide hydrolase 1 OS=Ctenocephalides felis GN=EH1 PE=1 SV=3|e=0|from=112|to=1506|frame=-2|Fragment_1
MGKCCRMLIFAAIAGIAVLYYQITKELPKPNIPLDTWWGPGKPQNVDISIRPFKININNK
VIENLKLKLNDVQYTLPLEGINFEYGFNTDSLKKIVDFWRTQYNWREREALLNKYPHFKT
NIQGLDIHYVHIKPQVSKNIEVLPLVMIHGWPGSFVEFYKIIPMLTTPRAGYNFVFELIL
PSIPGYGFSQAAAKPGLGSTQVAVIMRNLMERIGFKKYYVQGGDWGSMIISAMSTLFPEN
VLGQHSNMCFVNTPSSNIKAIIGSFFPESFAGTGNAHKMYPMSEHFFTLLEEMGYLHLQA
TKPDTVGVALRDSPAGLAAYILEKFSTWTNRSWRSVKDGNLLLKYNIPELLDNVMIYYVT
DSITTSMRLYAESFTKAHLALNLDRVRNHVPAACAKFPNELAYVTDCQLAEKYKTLLQSN
DMPSGGHFAAFEEPGLLAEDIFTAVKKFKEFYSKKAEESQKKADL
>Locus_2081_Transcript_1/1_Confidence_1.000_Length_709|UNIGENE REPRESENTATIVE TRANSCRIPT|sp|Q8SWV6|FICD_DROME Adenosine monophosphate-protein transferase FICD homolog OS=Drosophila melanogaster GN=CG9523 PE=1 SV=1|e=1.23443E-38|from=315|to=707|frame=3|Fragment_1
EANKVSEIEALSSLKAAMDLKVQGRLDRALKLFEHAAALAPKHPDVLTKYGEFLEETKKD
IVKADQLYFQALTYCPTHTDAVSNRQRTSTIVANIDNTRLESIDRKRDALAAIPESNGSL
RRAKKEAYFSY

>Locus_28693_Transcript_1/1_Confidence_1.000_Length_379|UNIGENE
REPRESENTATIVE TRANSCRIPT|sp|Q94887|NRX4_DROME Neurexin-4
OS=Drosophila melanogaster GN=Nrx-IV PE=1 SV=2|e=2.48117E-55|from=2|
to=379|frame=-1|Fragment_1
GRYTLGPLLCEGDDLFNNVVTIRIKDAALNIPTFDMGHSGDIYFEFKTTIENAVLLHSTG
PTDNIKLSIIGGTKLQFQYQAGSGPLGVSVETAYHLNDDNWHSVSVERNRKEARIVVDGA
QKAEVR
>Locus_28338_Transcript_1/1_Confidence_1.000_Length_245|.|sp|Q94887|
NRX4_DROME Neurexin-4 OS=Drosophila melanogaster GN=Nrx-IV PE=1
SV=2|e=8.52647E-35|from=1|to=243|frame=1|Fragment_1
AYHLNDDNWHSVSVERNRKEARIVVDGAQKAEVREPPGPVRALHLTSDLTIGASVDYRDG
FVGCIRALLLNGELVDLKGYA
>Locus_8009_Transcript_1/1_Confidence_1.000_Length_1781|UNIGENE
REPRESENTATIVE TRANSCRIPT|sp|Q95T64|PGPLA_DROME Peptidoglycan-
recognition protein LA OS=Drosophila melanogaster GN=PGRP-LA PE=1
SV=2|e=3.93151E-34|from=1128|to=1778|frame=-1|Fragment_1
LLVCALLAFGLYFIVDVKKREVPYYDDIISDGRGSNAT
>Locus_8009_Transcript_1/1_Confidence_1.000_Length_1781|UNIGENE
REPRESENTATIVE TRANSCRIPT|sp|Q95T64|PGPLA_DROME Peptidoglycan-
recognition protein LA OS=Drosophila melanogaster GN=PGRP-LA PE=1
SV=2|e=3.93151E-34|from=1128|to=1778|frame=-1|Fragment_2
LGGGHQLIYRDTWGGVEPR
>Locus_8009_Transcript_1/1_Confidence_1.000_Length_1781|UNIGENE
REPRESENTATIVE TRANSCRIPT|sp|Q95T64|PGPLA_DROME Peptidoglycan-
recognition protein LA OS=Drosophila melanogaster GN=PGRP-LA PE=1
SV=2|e=3.93151E-34|from=1128|to=1778|frame=-1|Fragment_3
ATRRLYHPTPYVVLTHVGGLSRRCEDLSTCSLEMRNMQADHYIRFGSPDIGQNFMVGGDG
NVYVGRGWD
>Locus_8009_Transcript_1/1_Confidence_1.000_Length_1781|UNIGENE
REPRESENTATIVE TRANSCRIPT|sp|Q95T64|PGPLA_DROME Peptidoglycan-
recognition protein LA OS=Drosophila melanogaster GN=PGRP-LA PE=1
SV=2|e=3.93151E-34|from=1128|to=1778|frame=-1|Fragment_4
SGDYSKIHATPRQLDATQRLLALGVSMGKLSEDYKLIGHNQSTFTLSPGPNLFSEISKWP
HWYNCSYKMETTCGAEI
>Locus_13006_Transcript_1/1_Confidence_1.000_Length_349|UNIGENE
REPRESENTATIVE TRANSCRIPT|sp|Q95TU8|UNC5_DROME Netrin receptor unc-5
OS=Drosophila melanogaster GN=unc-5 PE=2 SV=1|e=7.79991E-17|from=48|
to=347|frame=-3|Fragment_1
GRRISSQVMLTVYVDGGWSAWSAWTPCDCPL
>Locus_13006_Transcript_1/1_Confidence_1.000_Length_349|UNIGENE
REPRESENTATIVE TRANSCRIPT|sp|Q95TU8|UNC5_DROME Netrin receptor unc-5
OS=Drosophila melanogaster GN=unc-5 PE=2 SV=1|e=7.79991E-17|from=48|
to=347|frame=-3|Fragment_2
GHERITSSARSSSQQGRKRIRYCNNPTPVNDGRKCPGKNVQRTSDCAQCP
>Locus_27691_Transcript_1/1_Confidence_1.000_Length_284|UNIGENE
REPRESENTATIVE TRANSCRIPT|sp|Q967D7|TUTL_DROME Protein turtle
OS=Drosophila melanogaster GN=tutl PE=2 SV=2|e=1.36073E-32|from=2|
to=265|frame=-2|Fragment_1
PNEKIFQL
>Locus_27691_Transcript_1/1_Confidence_1.000_Length_284|UNIGENE
REPRESENTATIVE TRANSCRIPT|sp|Q967D7|TUTL_DROME Protein turtle
OS=Drosophila melanogaster GN=tutl PE=2 SV=2|e=1.36073E-32|from=2|
to=265|frame=-2|Fragment_2
PRNLTVTEISNGFLITWQVPLERAHLIQFYTIKYRTDAQWKTLNKGQIRPEETSYLVKNL

VGGRTYYFR
>Locus_8363_Transcript_4/5_Confidence_0.667_Length_1008|UNIGENE REPRESENTATIVE TRANSCRIPT|sp|Q9GQQ0|SPIN_DROME Protein spinster OS=Drosophila melanogaster GN=spin PE=1 SV=1|e=7.06886E-74|from=1|to=627|frame=1|Fragment_1
LFYFAIPVGSGLGYIAGSAMAHIGGSWHWALRVTPALGLVAVILIKFIQDPVRGHSEQSM
DLEPTSYTDDLKSLLKNRSFMLSTAGFTCVAFVTGALAWWGPKFIHSGMILQPGNRDVTI
NDVSFKFGIVAMLAGVTGVPLGSLIASRLRPLYPSCDPLICATGMLVAAPLLYFAAITPG
YNTNLCFALMFFGQVSMNMNWPLVADMLL
>Locus_8363_Transcript_4/5_Confidence_0.667_Length_1008|.|sp|Q9GQQ0|SPIN_DROME Protein spinster OS=Drosophila melanogaster GN=spin PE=1 SV=1|e=1.05893E-45|from=629|to=1003|frame=2|Fragment_1
VAFVFGLLTMISGLVGVPLGSYLSQSLKKTYPRADPLLCAAGLLISTPLLAAGMLVVQLN
STVAFILMFFGELALNLNWAIVADILLYVVIPTRRSTAEAFQILISHTFGDAGSPYLIGV
ISEAL
>Locus_8363_Transcript_1/5_Confidence_0.500_Length_800|.|sp|Q9GQQ0|SPIN_DROME Protein spinster OS=Drosophila melanogaster GN=spin PE=1 SV=1|e=6.10372E-80|from=1|to=666|frame=1|Fragment_1
LFYFAIPVGSGLGYIAGSAMAHIGGSWHWALRVTPALGLVAVILIKFIQDPVRGHSEQSM
DLEPTSYTDDLKSLLKNRSFMLSTAGFTCVAFVTGALAWWGPKFIHSGMILQPGNRDVTI
NEVAFVFGLLTMISGLVGVPLGSYLSQSLKKTYPRADPLLCAAGLLISTPLLAAGMLVVQ
LNSTVAFILMFFGELALNLNWAIVADILL
>Locus_8363_Transcript_1/5_Confidence_0.500_Length_800|.|sp|Q9GQQ0|SPIN_DROME Protein spinster OS=Drosophila melanogaster GN=spin PE=1 SV=1|e=6.10372E-80|from=1|to=666|frame=1|Fragment_2
VRMPFMNNMANDF
>Locus_8363_Transcript_5/5_Confidence_0.583_Length_746|.|sp|Q9GQQ0|SPIN_DROME Protein spinster OS=Drosophila melanogaster GN=spin PE=1 SV=1|e=2.3579E-99|from=1|to=741|frame=1|Fragment_1
LFYFAIPVGSGLGYIAGSAMAHIGGSWHWALRVTPALGLVAVILIKFIQDPVRGHSEQSM
DLEPTSYTDDLKSLLKNRSFMLSTAGFTCVAFVTGALAWWGPKFIHSGMILQPGNRDVTI
NEVAFVFGLLTMISGLVGVPLGSYLSQSLKKTYPRADPLLCAAGLLISTPLLAAGMLVVQ
LNSTVAFILMFFGELALNLNWAIVADILLYVVIPTRRSTAEAFQILISHTFGDAGSPYLI
GVISEAL
>Locus_8363_Transcript_2/5_Confidence_0.500_Length_746|.|sp|Q9GQQ0|SPIN_DROME Protein spinster OS=Drosophila melanogaster GN=spin PE=1 SV=1|e=1.13342E-93|from=1|to=741|frame=1|Fragment_1
LFYFAIPVGSGLGYIAGSAMAHIGGSWHWALRVTPALGLVAVILIKFIQDPVRGHSEQSM
DLEPTSYTDDLKSLLKNRSFMLSTAGFTCVAFVTGALAWWGPKFIHSGMILQPGNRDVTI
NDVSFKFGIVAMLAGVTGVPLGSLIASRLRPLYPSCDPLICATGMLVAAPLLYFAAITPG
YNTNLCFALMFFGQVSMNMNWPLVADMLLYVVIPTRRSTAEAFQILISHTFGDAGSPYLI
GVISEAL
>Locus_8363_Transcript_3/5_Confidence_0.500_Length_719|.|sp|Q9GQQ0|SPIN_DROME Protein spinster OS=Drosophila melanogaster GN=spin PE=1 SV=1|e=8.12042E-94|from=1|to=714|frame=1|Fragment_1
SGLGYIAGSAMAHIGGSWHWALRVTPALGLVAVILIKFIQDPVRGHSEQSMDLEPTSYTD
DLKSLLKNRSFMLSTAGFTCVAFVTGALAWWGPKFIHSGMILQPGNRDVTINEVAFVFGL
LTMISGLVGVPLGSYLSQSLKKTYPRADPLLCAAGLLISTPLLAAGMLVVQLNSTVAFIL
MFFGELALNLNWAIVADILLYVVIPTRRSTAEAFQILISHTFGDAGSPYLIGVISEAL
>Locus_128_Transcript_5/10_Confidence_0.148_Length_1034|UNIGENE REPRESENTATIVE TRANSCRIPT|sp|Q9V3L1|NDST_DROME Bifunctional heparan sulfate N-deacetylase/N-sulfotransferase OS=Drosophila melanogaster GN=sfl PE=1 SV=1|e=4.74554E-73|from=3|to=719|frame=-1|Fragment_1
FTMASDSSKFAGYAQWRSEESRVLLNGNVSGHRNSS

>Locus_128_Transcript_5/10_Confidence_0.148_Length_1034|UNIGENE
REPRESENTATIVE TRANSCRIPT|sp|Q9V3L1|NDST_DROME Bifunctional heparan
sulfate N-deacetylase/N-sulfotransferase OS=Drosophila melanogaster
GN=sfl PE=1 SV=1|e=4.74554E-73|from=3|to=719|frame=-1|Fragment_2
LCRQVN
>Locus_128_Transcript_5/10_Confidence_0.148_Length_1034|UNIGENE
REPRESENTATIVE TRANSCRIPT|sp|Q9V3L1|NDST_DROME Bifunctional heparan
sulfate N-deacetylase/N-sulfotransferase OS=Drosophila melanogaster
GN=sfl PE=1 SV=1|e=4.74554E-73|from=3|to=719|frame=-1|Fragment_3
LMSAITILCYAHYVMNSPFTSLIHRDTVPTPLVQCRLGLA
>Locus_128_Transcript_5/10_Confidence_0.148_Length_1034|UNIGENE
REPRESENTATIVE TRANSCRIPT|sp|Q9V3L1|NDST_DROME Bifunctional heparan
sulfate N-deacetylase/N-sulfotransferase OS=Drosophila melanogaster
GN=sfl PE=1 SV=1|e=4.74554E-73|from=3|to=719|frame=-1|Fragment_4
KNDYQQSDHRSEARLRIDPKVLVFVETTYTQLGRDIAELLVHTRIKYKLEVAGKSLPVLT
NLDKGRYGVIVFENIEKYFMMDKWNRELLDKYCREYSVGIIGFMKPSEETLVGAQLQGFP
LFIHTNLRLQDASLNPKSPILRMVRSGE
>Locus_128_Transcript_1/10_Confidence_0.148_Length_953|.|sp|Q9V3L1|
NDST_DROME Bifunctional heparan sulfate N-deacetylase/N-
sulfotransferase OS=Drosophila melanogaster GN=sfl PE=1 SV=1|
e=9.76967E-62|from=3|to=638|frame=-1|Fragment_1
FTMASDSSKFAGYAQWRSEESRVLLNGNVSGHRNSS
>Locus_128_Transcript_1/10_Confidence_0.148_Length_953|.|sp|Q9V3L1|
NDST_DROME Bifunctional heparan sulfate N-deacetylase/N-
sulfotransferase OS=Drosophila melanogaster GN=sfl PE=1 SV=1|
e=9.76967E-62|from=3|to=638|frame=-1|Fragment_2
LCRQVN
>Locus_128_Transcript_1/10_Confidence_0.148_Length_953|.|sp|Q9V3L1|
NDST_DROME Bifunctional heparan sulfate N-deacetylase/N-
sulfotransferase OS=Drosophila melanogaster GN=sfl PE=1 SV=1|
e=9.76967E-62|from=3|to=638|frame=-1|Fragment_3
LMSAITILCYAHYVMNSPFTSLIHRDTVPTPLVQCRLGLA
>Locus_128_Transcript_1/10_Confidence_0.148_Length_953|.|sp|Q9V3L1|
NDST_DROME Bifunctional heparan sulfate N-deacetylase/N-
sulfotransferase OS=Drosophila melanogaster GN=sfl PE=1 SV=1|
e=9.76967E-62|from=3|to=638|frame=-1|Fragment_4
KNDYQQSDHRSEARLRIDPKVLVFVETTYTQLGRDIAELLVHTRIKYKLEVAGKSLPVLT
NLDKGRYGVIVFENIEKYFMMDKWNRELLDKYCREYSVGIIGFMKPSEETLVGAQLQGFP
L
>Locus_28925_Transcript_1/1_Confidence_1.000_Length_275|UNIGENE
REPRESENTATIVE TRANSCRIPT|sp|Q9V3X5|TMTC2_DROME Transmembrane and
TPR repeat-containing protein CG4341 OS=Drosophila melanogaster
GN=CG4341 PE=2 SV=1|e=2.5009E-23|from=9|to=275|frame=-1|Fragment_1
GYHLVNVFLHVLSTFLVVRLTRTFLTNTFGVFVTGALFAVHPVHVEAVAGIVGRADLLAC
CMQLGCFVLYEAHIRWRKIDDLRHWMALG
>Locus_1484_Transcript_1/2_Confidence_0.800_Length_1879|UNIGENE
REPRESENTATIVE TRANSCRIPT|sp|Q9V5E1|PAL1_DROME Peptidyl-alpha-
hydroxyglycine alpha-amidating lyase 1 OS=Drosophila melanogaster
GN=Pal1 PE=1 SV=1|e=2.42497E-90|from=397|to=1413|frame=1|Fragment_1
PVQQLVDVENTLSSLNFSPTYVPSWPKISDSLGQVSAVSLDSFGNVVLFHRGSRKWDAES
FNKDEIFLQQNLGPIPENTILVLNSTTGDVSYEWGSHMFYMPHGLTVDYFNNIWITDVAL
HQVMKFSFNASTSTKPSLTLGKKFLPGNSLDRFCKPTSVAVMRSGDFFVADGYCNSRIMK
FSMNGEYLTSWGKSTRNN
>Locus_1484_Transcript_1/2_Confidence_0.800_Length_1879|UNIGENE

REPRESENTATIVE TRANSCRIPT|sp|Q9V5E1|PAL1_DROME Peptidyl-alpha-
hydroxyglycine alpha-amidating lyase 1 OS=Drosophila melanogaster
GN=Pal1 PE=1 SV=1|e=2.42497E-90|from=397|to=1413|frame=1|Fragment_2
DRKPPPYNFRIPHALALAEDKDMLCVADRENGRVQCFNAINGSYIEKFQHPIIGQRIFSV
AYSSVNGGQLYVVNGPN
>Locus_1484_Transcript_1/2_Confidence_0.800_Length_1879|UNIGENE
REPRESENTATIVE TRANSCRIPT|sp|Q9V5E1|PAL1_DROME Peptidyl-alpha-
hydroxyglycine alpha-amidating lyase 1 OS=Drosophila melanogaster
GN=Pal1 PE=1 SV=1|e=2.42497E-90|from=397|to=1413|frame=1|Fragment_3
WMPYYNSVRGFVINMTSGKIVSQFRAGSKDFQNPHDIAVSSDGKQAYVVEIDPYVIHKFE
DNTL
>Locus_1484_Transcript_2/2_Confidence_0.800_Length_1879|.|sp|Q9V5E1|
PAL1_DROME Peptidyl-alpha-hydroxyglycine alpha-amidating lyase 1
OS=Drosophila melanogaster GN=Pal1 PE=1 SV=1|e=2.42497E-90|from=397|
to=1413|frame=1|Fragment_1
PVQQLVDVENTLSSLNFSPTYVPSWPKISDSLGQVSAVSLDSFGNVVLFHRGSRKWDAES
FNKDEIFLQQNLGPIPENTILVLNSTTGDVSYEWGSHMFYMPHGLTVDYFNNIWITDVAL
HQVMKFSFNASTSTKPSLTLGKKFLPGNSLDRFCKPTSVAVMRSGDFFVADGYCNSRIMK
FSMNGEYLTSWGKSTRNN
>Locus_1484_Transcript_2/2_Confidence_0.800_Length_1879|.|sp|Q9V5E1|
PAL1_DROME Peptidyl-alpha-hydroxyglycine alpha-amidating lyase 1
OS=Drosophila melanogaster GN=Pal1 PE=1 SV=1|e=2.42497E-90|from=397|
to=1413|frame=1|Fragment_2
DRKPPPYNFRIPHALALAEDKDMLCVADRENGRVQCFNAINGSYIEKFQHPIIGQRIFSV
AYSSVNGGQLYVVNGPN
>Locus_1484_Transcript_2/2_Confidence_0.800_Length_1879|.|sp|Q9V5E1|
PAL1_DROME Peptidyl-alpha-hydroxyglycine alpha-amidating lyase 1
OS=Drosophila melanogaster GN=Pal1 PE=1 SV=1|e=2.42497E-90|from=397|
to=1413|frame=1|Fragment_3
WMPYYNSVRGFVINMTSGKIVSQFRAGSKDFQNPHDIAVSSDGKQAYVVEIDPYVIHKFE
DNTL
>Locus_6536_Transcript_1/1_Confidence_1.000_Length_684|UNIGENE
REPRESENTATIVE TRANSCRIPT|sp|Q9V5N8|STAN_DROME Protocadherin-like
wing polarity protein stan OS=Drosophila melanogaster GN=stan PE=1
SV=4|e=4.46772E-22|from=186|to=488|frame=3|Fragment_1
YLPNITE
>Locus_6536_Transcript_1/1_Confidence_1.000_Length_684|UNIGENE
REPRESENTATIVE TRANSCRIPT|sp|Q9V5N8|STAN_DROME Protocadherin-like
wing polarity protein stan OS=Drosophila melanogaster GN=stan PE=1
SV=4|e=4.46772E-22|from=186|to=488|frame=3|Fragment_2
AQNPPDLHVMNSPQLFPSVQSNPQYMKPIYAPKWSSKLPDSYL
>Locus_6536_Transcript_1/1_Confidence_1.000_Length_684|UNIGENE
REPRESENTATIVE TRANSCRIPT|sp|Q9V5N8|STAN_DROME Protocadherin-like
wing polarity protein stan OS=Drosophila melanogaster GN=stan PE=1
SV=4|e=4.46772E-22|from=186|to=488|frame=3|Fragment_3
PYNKGRWSQDTGSDNEIQPNLVQSTLKNSVTQSSMPLPNPDLTLDTNHVRK
>Locus_26433_Transcript_1/1_Confidence_1.000_Length_271|.|sp|Q9V5N8|
STAN_DROME Protocadherin-like wing polarity protein stan
OS=Drosophila melanogaster GN=stan PE=1 SV=4|e=4.14612E-26|from=6|
to=254|frame=-3|Fragment_1
IQKRVEDAKRWVSETYASFAIPTTDKPWQSICLRKSQFINSLASFMPKSVEQYCKVTFID
LNDDRFAVETQNFHLVARDDVCI
>Locus_20402_Transcript_1/1_Confidence_1.000_Length_262|.|sp|Q9V5N8|
STAN_DROME Protocadherin-like wing polarity protein stan

OS=Drosophila melanogaster GN=stan PE=1 SV=4|e=1.34839E-16|from=63|
to=254|frame=3|Fragment_1
NKLYGADVLISEGILQYLIAYELSQTGLNLSHSQDKDYIQNIVSVASNILDVRYEKEWNQ
IAKL
>Locus_6927_Transcript_1/1_Confidence_1.000_Length_676|UNIGENE
REPRESENTATIVE TRANSCRIPT|sp|Q9V668|S61G1_DROME Protein transport
protein Sec61 gamma-1 subunit OS=Drosophila melanogaster GN=SEC61G1
PE=3 SV=1|e=2.31513E-15|from=264|to=437|frame=3|Fragment_1
MDHVTKFIEPGRQFAKDSIRLVKRCTKPDRKEFQKIAIATA
>Locus_6927_Transcript_1/1_Confidence_1.000_Length_676|UNIGENE
REPRESENTATIVE TRANSCRIPT|sp|Q9V668|S61G1_DROME Protein transport
protein Sec61 gamma-1 subunit OS=Drosophila melanogaster GN=SEC61G1
PE=3 SV=1|e=2.31513E-15|from=264|to=437|frame=3|Fragment_2
VKLIH
>Locus_3215_Transcript_1/4_Confidence_0.700_Length_2763|UNIGENE
REPRESENTATIVE TRANSCRIPT|sp|Q9V730|EXT1_DROME Exostosin-1
OS=Drosophila melanogaster GN=ttv PE=1 SV=1|e=0|from=217|to=2418|
frame=-1|Fragment_1
MRAKKRYLLLIVSCAFLAYCYFGGYRLK
>Locus_3215_Transcript_1/4_Confidence_0.700_Length_2763|UNIGENE
REPRESENTATIVE TRANSCRIPT|sp|Q9V730|EXT1_DROME Exostosin-1
OS=Drosophila melanogaster GN=ttv PE=1 SV=1|e=0|from=217|to=2418|
frame=-1|Fragment_2
SISKTLPYETLPSFVSV
>Locus_3215_Transcript_1/4_Confidence_0.700_Length_2763|UNIGENE
REPRESENTATIVE TRANSCRIPT|sp|Q9V730|EXT1_DROME Exostosin-1
OS=Drosophila melanogaster GN=ttv PE=1 SV=1|e=0|from=217|to=2418|
frame=-1|Fragment_3
S
>Locus_3215_Transcript_1/4_Confidence_0.700_Length_2763|UNIGENE
REPRESENTATIVE TRANSCRIPT|sp|Q9V730|EXT1_DROME Exostosin-1
OS=Drosophila melanogaster GN=ttv PE=1 SV=1|e=0|from=217|to=2418|
frame=-1|Fragment_4
PQAGSRPCRMETCFDFSRCRGEFRVYVYPQQDINE
>Locus_3215_Transcript_1/4_Confidence_0.700_Length_2763|UNIGENE
REPRESENTATIVE TRANSCRIPT|sp|Q9V730|EXT1_DROME Exostosin-1
OS=Drosophila melanogaster GN=ttv PE=1 SV=1|e=0|from=217|to=2418|
frame=-1|Fragment_5
ASTPISSSYMKVLSVIRESRYYTPDPSRACLFILALDTLDRDKLSSDFVRGMPGRLARLP
YWNNGRNHLIFNLYSGSWPDYAERDLGFDPGYALLAQASMSVAHFRHGFDVSLPLFHRGH
PERGGAPGNAQSNNFPANKKYLLAFKGKRYVHGIGSETRNSLWHLHNENDMVLVTTCRHG
KSWKDLKDERCDEDNAEYDRYDYDVLLANSTFCLVPRGRRLGSYRFLEALQAGCVPVLLS
NAWALPFQQVIDWSQAVLWADERLLLQVPDLVRSVSADRVLALRQQTQVLWERYFSSIEK
IVFTTLEIIRERLPGEPVRSGLVWNTNPGALLTLTRFSDSWARYPFYLDALGASPSAGFT
AVIYCQLGAVTGGAPASVLQRLIRNVARSKSVQRIIIVWANEKSPPARNRWPPTPTSVPL
NILQAPVTLIY
>Locus_3215_Transcript_1/4_Confidence_0.700_Length_2763|UNIGENE
REPRESENTATIVE TRANSCRIPT|sp|Q9V730|EXT1_DROME Exostosin-1
OS=Drosophila melanogaster GN=ttv PE=1 SV=1|e=0|from=217|to=2418|
frame=-1|Fragment_6
SR
>Locus_3215_Transcript_1/4_Confidence_0.700_Length_2763|UNIGENE
REPRESENTATIVE TRANSCRIPT|sp|Q9V730|EXT1_DROME Exostosin-1
OS=Drosophila melanogaster GN=ttv PE=1 SV=1|e=0|from=217|to=2418|

frame=-1|Fragment_7
RFLPHPLITTDAILSLDEDALLTTDEVDFAFSVWRNFPDRIVGYPARAHYWDDAKSTWGY
TSKWTNEYSIVLTGAAFYHRYYNELYTRWLSPLLHKTVEQSDNCEDILMNFLVAHVTRRP
PIKVTQRKQYKEAPAGGARSPWNDPDHFIQRQSCLNTFAAVFGYMPLLRSSLRLDPVLFK
DPVSNLRKKYRQIELVGS
>Locus_3215_Transcript_2/4_Confidence_0.700_Length_2763|.|sp|Q9V730|
EXT1_DROME Exostosin-1 OS=Drosophila melanogaster GN=ttv PE=1 SV=1|
e=0|from=217|to=2418|frame=-1|Fragment_1
MRAKKRYLLLIVSCAFLAYCYFGGYRLK
>Locus_3215_Transcript_2/4_Confidence_0.700_Length_2763|.|sp|Q9V730|
EXT1_DROME Exostosin-1 OS=Drosophila melanogaster GN=ttv PE=1 SV=1|
e=0|from=217|to=2418|frame=-1|Fragment_2
SISKTLPYETLPSFVSV
>Locus_3215_Transcript_2/4_Confidence_0.700_Length_2763|.|sp|Q9V730|
EXT1_DROME Exostosin-1 OS=Drosophila melanogaster GN=ttv PE=1 SV=1|
e=0|from=217|to=2418|frame=-1|Fragment_3
S
>Locus_3215_Transcript_2/4_Confidence_0.700_Length_2763|.|sp|Q9V730|
EXT1_DROME Exostosin-1 OS=Drosophila melanogaster GN=ttv PE=1 SV=1|
e=0|from=217|to=2418|frame=-1|Fragment_4
PQAGSRPCRMETCFDFSRCRGEFRVYVYPQQDINE
>Locus_3215_Transcript_2/4_Confidence_0.700_Length_2763|.|sp|Q9V730|
EXT1_DROME Exostosin-1 OS=Drosophila melanogaster GN=ttv PE=1 SV=1|
e=0|from=217|to=2418|frame=-1|Fragment_5
ASTPISSSYMKVLSVIRESRYYTPDPSRACLFILALDTLDRDKLSSDFVRGMPGRLARLP
YWNNGRNHLIFNLYSGSWPDYAERDLGFDPGYALLAQASMSVAHFRHGFDVSLPLFHRGH
PERGGAPGNAQSNNFPANKKYLLAFKGKRYVHGIGSETRNSLWHLHNENDMVLVTTCRHG
KSWKDLKDERCDEDNAEYDRYDYDVLLANSTFCLVPRGRRLGSYRFLEALQAGCVPVLLS
NAWALPFQQVIDWSQAVLWADERLLLQVPDLVRSVSADRVLALRQQTQVLWERYFSSIEK
IVFTTLEIIRERLPGEPVRSGLVWNTNPGALLTLTRFSDSWARYPFYLDALGASPSAGFT
AVIYCQLGAVTGGAPASVLQRLIRNVARSKSVQRIIIVWANEKSPPARNRWPPTPTSVPL
NILQAPVTLIY
>Locus_3215_Transcript_2/4_Confidence_0.700_Length_2763|.|sp|Q9V730|
EXT1_DROME Exostosin-1 OS=Drosophila melanogaster GN=ttv PE=1 SV=1|
e=0|from=217|to=2418|frame=-1|Fragment_6
SR
>Locus_3215_Transcript_2/4_Confidence_0.700_Length_2763|.|sp|Q9V730|
EXT1_DROME Exostosin-1 OS=Drosophila melanogaster GN=ttv PE=1 SV=1|
e=0|from=217|to=2418|frame=-1|Fragment_7
RFLPHPLITTDAILSLDEDALLTTDEVDFAFSVWRNFPDRIVGYPARAHYWDDAKSTWGY
TSKWTNEYSIVLTGAAFYHRYYNELYTRWLSPLLHKTVEQSDNCEDILMNFLVAHVTRRP
PIKVTQRKQYKEAPAGGARSPWNDPDHFIQRQSCLNTFAAVFGYMPLLRSSLRLDPVLFK
DPVSNLRKKYRQIELVGS
>Locus_3215_Transcript_3/4_Confidence_0.700_Length_2763|.|sp|Q9V730|
EXT1_DROME Exostosin-1 OS=Drosophila melanogaster GN=ttv PE=1 SV=1|
e=0|from=217|to=2418|frame=-1|Fragment_1
MRAKKRYLLLIVSCAFLAYCYFGGYRLK
>Locus_3215_Transcript_3/4_Confidence_0.700_Length_2763|.|sp|Q9V730|
EXT1_DROME Exostosin-1 OS=Drosophila melanogaster GN=ttv PE=1 SV=1|
e=0|from=217|to=2418|frame=-1|Fragment_2
SISKTLPYETLPSFVSV
>Locus_3215_Transcript_3/4_Confidence_0.700_Length_2763|.|sp|Q9V730|
EXT1_DROME Exostosin-1 OS=Drosophila melanogaster GN=ttv PE=1 SV=1|
e=0|from=217|to=2418|frame=-1|Fragment_3

S
>Locus_3215_Transcript_3/4_Confidence_0.700_Length_2763|.|sp|Q9V730|
EXT1_DROME Exostosin-1 OS=Drosophila melanogaster GN=ttv PE=1 SV=1|
e=0|from=217|to=2418|frame=-1|Fragment_4
PQAGSRPCRMETCFDFSRCRGEFRVYVYPQQDINE
>Locus_3215_Transcript_3/4_Confidence_0.700_Length_2763|.|sp|Q9V730|
EXT1_DROME Exostosin-1 OS=Drosophila melanogaster GN=ttv PE=1 SV=1|
e=0|from=217|to=2418|frame=-1|Fragment_5
ASTPISSSYMKVLSVIRESRYYTPDPSRACLFILALDTLDRDKLSSDFVRGMPGRLARLP
YWNNGRNHLIFNLYSGSWPDYAERDLGFDPGYALLAQASMSVAHFRHGFDVSLPLFHRGH
PERGGAPGNAQSNNFPANKKYLLAFKGKRYVHGIGSETRNSLWHLHNENDMVLVTTCRHG
KSWKDLKDERCDEDNAEYDRYDYDVLLANSTFCLVPRGRRLGSYRFLEALQAGCVPVLLS
NAWALPFQQVIDWSQAVLWADERLLLQVPDLVRSVSADRVLALRQQTQVLWERYFSSIEK
IVFTTLEIIRERLPGEPVRSGLVWNTNPGALLTLTRFSDSWARYPFYLDALGASPSAGFT
AVIYCQLGAVTGGAPASVLQRLIRNVARSKSVQRIIIVWANEKSPPARNRWPPTPTSVPL
NILQAPVTLIY
>Locus_3215_Transcript_3/4_Confidence_0.700_Length_2763|.|sp|Q9V730|
EXT1_DROME Exostosin-1 OS=Drosophila melanogaster GN=ttv PE=1 SV=1|
e=0|from=217|to=2418|frame=-1|Fragment_6
SR
>Locus_3215_Transcript_3/4_Confidence_0.700_Length_2763|.|sp|Q9V730|
EXT1_DROME Exostosin-1 OS=Drosophila melanogaster GN=ttv PE=1 SV=1|
e=0|from=217|to=2418|frame=-1|Fragment_7
RFLPHPLITTDAILSLDEDALLTTDEVDFAFSVWRNFPDRIVGYPARAHYWDDAKSTWGY
TSKWTNEYSIVLTGAAFYHRYYNELYTRWLSPLLHKTVEQSDNCEDILMNFLVAHVTRRP
PIKVTQRKQYKEAPAGGARSPWNDPDHFIQRQSCLNTFAAVFGYMPLLRSSLRLDPVLFK
DPVSNLRKKYRQIELVGS
>Locus_3215_Transcript_4/4_Confidence_0.700_Length_2763|.|sp|Q9V730|
EXT1_DROME Exostosin-1 OS=Drosophila melanogaster GN=ttv PE=1 SV=1|
e=0|from=217|to=2418|frame=-1|Fragment_1
MRAKKRYLLLIVSCAFLAYCYFGGYRLK
>Locus_3215_Transcript_4/4_Confidence_0.700_Length_2763|.|sp|Q9V730|
EXT1_DROME Exostosin-1 OS=Drosophila melanogaster GN=ttv PE=1 SV=1|
e=0|from=217|to=2418|frame=-1|Fragment_2
SISKTLPYETLPSFVSV
>Locus_3215_Transcript_4/4_Confidence_0.700_Length_2763|.|sp|Q9V730|
EXT1_DROME Exostosin-1 OS=Drosophila melanogaster GN=ttv PE=1 SV=1|
e=0|from=217|to=2418|frame=-1|Fragment_3
S
>Locus_3215_Transcript_4/4_Confidence_0.700_Length_2763|.|sp|Q9V730|
EXT1_DROME Exostosin-1 OS=Drosophila melanogaster GN=ttv PE=1 SV=1|
e=0|from=217|to=2418|frame=-1|Fragment_4
PQAGSRPCRMETCFDFSRCRGEFRVYVYPQQDINE
>Locus_3215_Transcript_4/4_Confidence_0.700_Length_2763|.|sp|Q9V730|
EXT1_DROME Exostosin-1 OS=Drosophila melanogaster GN=ttv PE=1 SV=1|
e=0|from=217|to=2418|frame=-1|Fragment_5
ASTPISSSYMKVLSVIRESRYYTPDPSRACLFILALDTLDRDKLSSDFVRGMPGRLARLP
YWNNGRNHLIFNLYSGSWPDYAERDLGFDPGYALLAQASMSVAHFRHGFDVSLPLFHRGH
PERGGAPGNAQSNNFPANKKYLLAFKGKRYVHGIGSETRNSLWHLHNENDMVLVTTCRHG
KSWKDLKDERCDEDNAEYDRYDYDVLLANSTFCLVPRGRRLGSYRFLEALQAGCVPVLLS
NAWALPFQQVIDWSQAVLWADERLLLQVPDLVRSVSADRVLALRQQTQVLWERYFSSIEK
IVFTTLEIIRERLPGEPVRSGLVWNTNPGALLTLTRFSDSWARYPFYLDALGASPSAGFT
AVIYCQLGAVTGGAPASVLQRLIRNVARSKSVQRIIIVWANEKSPPARNRWPPTPTSVPL
NILQAPVTLIY

>Locus_3215_Transcript_4/4_Confidence_0.700_Length_2763|.|sp|Q9V730|
EXT1_DROME Exostosin-1 OS=Drosophila melanogaster GN=ttv PE=1 SV=1|
e=0|from=217|to=2418|frame=-1|Fragment_6
SR
>Locus_3215_Transcript_4/4_Confidence_0.700_Length_2763|.|sp|Q9V730|
EXT1_DROME Exostosin-1 OS=Drosophila melanogaster GN=ttv PE=1 SV=1|
e=0|from=217|to=2418|frame=-1|Fragment_7
RFLPHPLITTDAILSLDEDALLTTDEVDFAFSVWRNFPDRIVGYPARAHYWDDAKSTWGY
TSKWTNEYSIVLTGAAFYHRYYNELYTRWLSPLLHKTVEQSDNCEDILMNFLVAHVTRRP
PIKVTQRKQYKEAPAGGARSPWNDPDHFIQRQSCLNTFAAVFGYMPLLRSSLRLDPVLFK
DPVSNLRKKYRQIELVGS
>Locus_1200_Transcript_3/8_Confidence_0.556_Length_1274|UNIGENE
REPRESENTATIVE TRANSCRIPT|sp|Q9V7S5|PICO_DROME Putative inorganic
phosphate cotransporter OS=Drosophila melanogaster GN=Picot PE=1
SV=1|e=3.263E-154|from=229|to=1272|frame=1|Fragment_1
RPEGWWGTRHFVTFMLFLGMANAYVMRTNMSVAIVAMVNHTAVGVGH
>Locus_1200_Transcript_3/8_Confidence_0.556_Length_1274|UNIGENE
REPRESENTATIVE TRANSCRIPT|sp|Q9V7S5|PICO_DROME Putative inorganic
phosphate cotransporter OS=Drosophila melanogaster GN=Picot PE=1
SV=1|e=3.263E-154|from=229|to=1272|frame=1|Fragment_2
EIVDDECPDTDYGNHTSTVQDGEFIWSTSLQSQILSSFFWGYVITQIPFGILSKRYGAKW
FLGVGMMINSLFGLLVPVAAREGGSGWLIAVRFIQGLGEGPIVPCTHAMLAKWIPPNERS
RMGAAVYAGAQFGTVVSMPLSGLLSEYGFDGGWPSIFYVFGLVGAIWSAAFLLWVHEDPE
VHPTIDQREKKYIVSSLWGTAGTSTPPIPFKKILLSLPFWAIMFAHMGQNYGYETLMTEL
PTYMKQVLRFSIKDNGILSALPYLAMWLFSMFISVIADWLLTSERLTHTSLRKIINGIGQ
F
>Locus_1200_Transcript_4/8_Confidence_0.500_Length_1079|.|sp|Q9V7S5|
PICO_DROME Putative inorganic phosphate cotransporter OS=Drosophila
melanogaster GN=Picot PE=1 SV=1|e=2.0042E-154|from=31|to=1077|
frame=1|Fragment_1
KKPEGWWGTRHFVTFMLFLGMANAYVMRTNMSVAIVAMVNHTAVGVGH
>Locus_1200_Transcript_4/8_Confidence_0.500_Length_1079|.|sp|Q9V7S5|
PICO_DROME Putative inorganic phosphate cotransporter OS=Drosophila
melanogaster GN=Picot PE=1 SV=1|e=2.0042E-154|from=31|to=1077|
frame=1|Fragment_2
EIVDDECPDTDYGNHTSTVQDGEFIWSTSLQSQILSSFFWGYVITQIPFGILSKRYGAKW
FLGVGMMINSLFGLLVPVAAREGGSGWLIAVRFIQGLGEGPIVPCTHAMLAKWIPPNERS
RMGAAVYAGAQFGTVVSMPLSGLLSEYGFDGGWPSIFYVFGLVGAIWSAAFLLWVHEDPE
VHPTIDQREKKYIVSSLWGTAGTSTPPIPFKKILLSLPFWAIMFAHMGQNYGYETLMTEL
PTYMKQVLRFSIKDNGILSALPYLAMWLFSMFISVIADWLLTSERLTHTSLRKIINGIGQ
F
>Locus_1200_Transcript_5/8_Confidence_0.556_Length_1079|.|sp|Q9V7S5|
PICO_DROME Putative inorganic phosphate cotransporter OS=Drosophila
melanogaster GN=Picot PE=1 SV=1|e=2.0042E-154|from=31|to=1077|
frame=1|Fragment_1
KKPEGWWGTRHFVTFMLFLGMANAYVMRTNMSVAIVAMVNHTAVGVGH
>Locus_1200_Transcript_5/8_Confidence_0.556_Length_1079|.|sp|Q9V7S5|
PICO_DROME Putative inorganic phosphate cotransporter OS=Drosophila
melanogaster GN=Picot PE=1 SV=1|e=2.0042E-154|from=31|to=1077|
frame=1|Fragment_2
EIVDDECPDTDYGNHTSTVQDGEFIWSTSLQSQILSSFFWGYVITQIPFGILSKRYGAKW
FLGVGMMINSLFGLLVPVAAREGGSGWLIAVRFIQGLGEGPIVPCTHAMLAKWIPPNERS
RMGAAVYAGAQFGTVVSMPLSGLLSEYGFDGGWPSIFYVFGLVGAIWSAAFLLWVHEDPE
VHPTIDQREKKYIVSSLWGTAGTSTPPIPFKKILLSLPFWAIMFAHMGQNYGYETLMTEL

PTYMKQVLRFSIKDNGILSALPYLAMWLFSMFISVIADWLLTSERLTHTSLRKIINGIGQ
F
>Locus_1200_Transcript_6/8_Confidence_0.611_Length_1079|.|sp|Q9V7S5|
PICO_DROME Putative inorganic phosphate cotransporter OS=Drosophila
melanogaster GN=Picot PE=1 SV=1|e=2.0042E-154|from=31|to=1077|
frame=1|Fragment_1
KKPEGWWGTRHFVTFMLFLGMANAYVMRTNMSVAIVAMVNHTAVGVGH
>Locus_1200_Transcript_6/8_Confidence_0.611_Length_1079|.|sp|Q9V7S5|
PICO_DROME Putative inorganic phosphate cotransporter OS=Drosophila
melanogaster GN=Picot PE=1 SV=1|e=2.0042E-154|from=31|to=1077|
frame=1|Fragment_2
EIVDDECPDTDYGNHTSTVQDGEFIWSTSLQSQILSSFFWGYVITQIPFGILSKRYGAKW
FLGVGMMINSLFGLLVPVAAREGGSGWLIAVRFIQGLGEGPIVPCTHAMLAKWIPPNERS
RMGAAVYAGAQFGTVVSMPLSGLLSEYGFDGGWPSIFYVFGLVGAIWSAAFLLWVHEDPE
VHPTIDQREKKYIVSSLWGTAGTSTPPIPFKKILLSLPFWAIMFAHMGQNYGYETLMTEL
PTYMKQVLRFSIKDNGILSALPYLAMWLFSMFISVIADWLLTSERLTHTSLRKIINGIGQ
F
>Locus_1200_Transcript_8/8_Confidence_0.556_Length_1079|.|sp|Q9V7S5|
PICO_DROME Putative inorganic phosphate cotransporter OS=Drosophila
melanogaster GN=Picot PE=1 SV=1|e=2.0042E-154|from=31|to=1077|
frame=1|Fragment_1
KKPEGWWGTRHFVTFMLFLGMANAYVMRTNMSVAIVAMVNHTAVGVGH
>Locus_1200_Transcript_8/8_Confidence_0.556_Length_1079|.|sp|Q9V7S5|
PICO_DROME Putative inorganic phosphate cotransporter OS=Drosophila
melanogaster GN=Picot PE=1 SV=1|e=2.0042E-154|from=31|to=1077|
frame=1|Fragment_2
EIVDDECPDTDYGNHTSTVQDGEFIWSTSLQSQILSSFFWGYVITQIPFGILSKRYGAKW
FLGVGMMINSLFGLLVPVAAREGGSGWLIAVRFIQGLGEGPIVPCTHAMLAKWIPPNERS
RMGAAVYAGAQFGTVVSMPLSGLLSEYGFDGGWPSIFYVFGLVGAIWSAAFLLWVHEDPE
VHPTIDQREKKYIVSSLWGTAGTSTPPIPFKKILLSLPFWAIMFAHMGQNYGYETLMTEL
PTYMKQVLRFSIKDNGILSALPYLAMWLFSMFISVIADWLLTSERLTHTSLRKIINGIGQ
F
>Locus_1200_Transcript_2/8_Confidence_0.500_Length_1079|.|sp|Q9V7S5|
PICO_DROME Putative inorganic phosphate cotransporter OS=Drosophila
melanogaster GN=Picot PE=1 SV=1|e=1.2991E-153|from=49|to=1077|
frame=1|Fragment_1
WGTRHFVTFMLFLGMANAYVMRTNMSVAIVAMVNHTAVGVGH
>Locus_1200_Transcript_2/8_Confidence_0.500_Length_1079|.|sp|Q9V7S5|
PICO_DROME Putative inorganic phosphate cotransporter OS=Drosophila
melanogaster GN=Picot PE=1 SV=1|e=1.2991E-153|from=49|to=1077|
frame=1|Fragment_2
EIVDDECPDTDYGNHTSTVQDGEFIWSTSLQSQILSSFFWGYVITQIPFGILSKRYGAKW
FLGVGMMINSLFGLLVPVAAREGGSGWLIAVRFIQGLGEGPIVPCTHAMLAKWIPPNERS
RMGAAVYAGAQFGTVVSMPLSGLLSEYGFDGGWPSIFYVFGLVGAIWSAAFLLWVHEDPE
VHPTIDQREKKYIVSSLWGTAGTSTPPIPFKKILLSLPFWAIMFAHMGQNYGYETLMTEL
PTYMKQVLRFSIKDNGILSALPYLAMWLFSMFISVIADWLLTSERLTHTSLRKIINGIGQ
F
>Locus_1200_Transcript_1/8_Confidence_0.167_Length_272|.|sp|Q9V7S5|
PICO_DROME Putative inorganic phosphate cotransporter OS=Drosophila
melanogaster GN=Picot PE=1 SV=1|e=2.33668E-37|from=4|to=270|frame=1|
Fragment_1
ILLSLPFWAIMFAHMGQNYGYETLMTELPTYMKQVLRFSIKDNGILSALPYLAMWLFSMF
ISVIADWLLTSERLTHTSLRKIINGIGQF
>Locus_1695_Transcript_1/7_Confidence_0.667_Length_2073|UNIGENE

REPRESENTATIVE TRANSCRIPT|sp|Q9V7Y2|SGPL_DROME Sphingosine-1-phosphate lyase OS=Drosophila melanogaster GN=Sply PE=2 SV=1|e=0|from=172|to=1755|frame=1|Fragment_1
INARLASKEPWQIIAITATSVISFLWLHDLLTADEGIKTRLLKSLFKWAKKIPYVKKRID
DELNKISAQFDKDIQKRMQDVPYITTLPEQGLSDEEIVKMAKSYLNLGTYKWKEGRVSGA
VYYYDPELIELNTKIYGMTSYTNPLHPDVFPGVSKMEAEVVRITAKLFHGGPNACGTMTT
GGTESIIMACKAYRDYARE
>Locus_1695_Transcript_1/7_Confidence_0.667_Length_2073|UNIGENE REPRESENTATIVE TRANSCRIPT|sp|Q9V7Y2|SGPL_DROME Sphingosine-1-phosphate lyase OS=Drosophila melanogaster GN=Sply PE=2 SV=1|e=0|from=172|to=1755|frame=1|Fragment_2
RGITKPNIVMPVTAHSGFDKAAQYLGLGVRTVRVDPETTLVDLKAMKRAINGNTCMLVAS
APNFPYGTMDDVQGVAELGLKYNIPVHVDACLGGFVIAFMRKAGFELPPFDFEVEGVTSI
SADTHKYGFAPKGSSVILYSDKKYRDHQFVVTTDWPGGVYGSPTVNGSRTGGIIAACWST
LLRFGETGYIDATKKIIDTTKYVEKEIRKIDEVFIFGKPATTVVALGSKVFDIYHLADKL
GGMGWSLNVLQFPSGIHICITHVHTQNGVADQFLDDMKSCIAEIMKNPSAPVEGKMAIYG
MAQSVPDRSTVTAFTKCYINSMYYTPADK
>Locus_1695_Transcript_2/7_Confidence_0.611_Length_2073|.|sp|Q9V7Y2|SGPL_DROME Sphingosine-1-phosphate lyase OS=Drosophila melanogaster GN=Sply PE=2 SV=1|e=0|from=172|to=1755|frame=1|Fragment_1
INARLASKEPWQIIAITATSVISFLWLHDLLTADEGIKTRLLKSLFKWAKKIPYVKKRID
DELNKISAQFDKDIQKRMQDVPYITTLPEQGLSDEEIVKMAKSYLNLGTYKWKEGRVSGA
VYYYDPELIELNTKIYGMTSYTNPLHPDVFPGVSKMEAEVVRITAKLFHGGPNACGTMTT
GGTESIIMACKAYRDYARE
>Locus_1695_Transcript_2/7_Confidence_0.611_Length_2073|.|sp|Q9V7Y2|SGPL_DROME Sphingosine-1-phosphate lyase OS=Drosophila melanogaster GN=Sply PE=2 SV=1|e=0|from=172|to=1755|frame=1|Fragment_2
RGITKPNIVMPVTAHSGFDKAAQYLGLGVRTVRVDPETTLVDLKAMKRAINGNTCMLVAS
APNFPYGTMDDVQGVAKLGLKYNIPVHVDACLGGFVIAFMRKAGFELPPFDFEVEGVTSI
SADTHKYGFAPKGSSVILYSDKKYRDHQFVVTTDWPGGVYGSPTVNGSRTGGIIAACWST
LLRFGETGYIDATKKIIDTTKYVEKEIRKIDEVFIFGKPATTVVALGSKVFDIYHLADKL
GGMGWSLNVLQFPSGIHICITHVHTQNGVADQFLDDMKSCIAEIMKNPSAPVEGKMAIYG
MAQSVPDRSTVTAFTKCYINSMYYTPADK
>Locus_1695_Transcript_3/7_Confidence_0.667_Length_2073|.|sp|Q9V7Y2|SGPL_DROME Sphingosine-1-phosphate lyase OS=Drosophila melanogaster GN=Sply PE=2 SV=1|e=0|from=172|to=1755|frame=1|Fragment_1
INARLASKEPWQIIAITATSVISFLWLHDLLTADEGIKTRLLKSLFKWAKKIPYVKKRID
DELNKISAQFDKDIQKRMQDVPYITTLPEQGLSDEEIVKMAKSYLNLGTYKWKEGRVSGA
VYYYDPELIELNTKIYGMTSYTNPLHPDVFPGVSKMEAEVVRITAKLFHGGPNACGTMTT
GGTESIIMACKAYRDYARE
>Locus_1695_Transcript_3/7_Confidence_0.667_Length_2073|.|sp|Q9V7Y2|SGPL_DROME Sphingosine-1-phosphate lyase OS=Drosophila melanogaster GN=Sply PE=2 SV=1|e=0|from=172|to=1755|frame=1|Fragment_2
RGITKPNIVMPVTAHSGFDKAAQYLGLGVRTVRVDPETTLVDLKAMKRAINGNTCMLVAS
APNFPYGTMDDVQGVAELGLKYNIPVHVDACLGGFVIAFMRKAGFELPPFDFEVEGVTSI
SADTHKYGFAPKGSSVILYSDKKYRDHQFVVTTDWPGGVYGSPTVNGSRTGGIIAACWST
LLRFGETGYIDATKKIIDTTKYVEKEIRKIDEVFIFGKPATTVVALGSKVFDIYHLADKL
GGMGWSLNVLQFPSGIHICITHVHTQNGVADQFLDDMKSCIAEIMKNPSAPVEGKMAIYG
MAQSVPDRSTVTAFTKCYINSMYYTPADK
>Locus_1695_Transcript_5/7_Confidence_0.611_Length_2073|.|sp|Q9V7Y2|SGPL_DROME Sphingosine-1-phosphate lyase OS=Drosophila melanogaster GN=Sply PE=2 SV=1|e=0|from=172|to=1755|frame=1|Fragment_1
INARLASKEPWQIIAITATSVISFLWLHDLLTADEGIKTRLLKSLFKWAKKIPYVKKRID
DELNKISAQFDKDIQKRMQDVPYITTLPEQGLSDEEIVKMAKSYLNLGTYKWKEGRVSGA

VYYYDPELIELNTKIYGMTSYTNPLHPDVFPGVSKMEAEVVRITAKLFHGGPNACGTMTT
GGTESIIMACKAYRDYARE
>Locus_1695_Transcript_5/7_Confidence_0.611_Length_2073|.|sp|Q9V7Y2|
SGPL_DROME Sphingosine-1-phosphate lyase OS=Drosophila melanogaster
GN=Sply PE=2 SV=1|e=0|from=172|to=1755|frame=1|Fragment_2
RGITKPNIVMPVTAHSGFDKAAQYLGLGVRTVRVDPETTLVDLKAMKRAINGNTCMLVAS
APNFPYGTMDDVQGVAELGLKYNIPVHVDACLGGFVIAFMRKAGFELPPFDFEVEGVTSI
SADTHKYGFAPKGSSVILYSDKKYRDHQFVVTTDWPGGVYGSPTVNGSRTGGIIAACWST
LLRFGETGYIDATKKIIDTTKYVEKEIRKIDEVFIFGKPATTVVALGSKVFDIYHLADKL
GGMGWSLNVLQFPSGIHICITHVHTQNGVADQFLDDMKSCIAEIMKNPSAPVEGKMAIYG
MAQSVPDRSTVTAFTKCYINSMYYTPADK
>Locus_1695_Transcript_6/7_Confidence_0.556_Length_2073|.|sp|Q9V7Y2|
SGPL_DROME Sphingosine-1-phosphate lyase OS=Drosophila melanogaster
GN=Sply PE=2 SV=1|e=0|from=172|to=1755|frame=1|Fragment_1
INARLASKEPWQIIAITATSVISFLWLHDLLTADEGIKTRLLKSLFKWAKKIPYVKKCIN
DELNKISAQFDKDIQKRMQDVPYITTLPEQGLSDEEIVKMAKSYLNLGTYKWKEGRVSGA
VYYYDPELIELNTKIYGMTSYTNPLHPDVFPGVSKMEAEVVRITAKLFHGGPNACGTMTT
GGTESIIMACKAYRDYARE
>Locus_1695_Transcript_6/7_Confidence_0.556_Length_2073|.|sp|Q9V7Y2|
SGPL_DROME Sphingosine-1-phosphate lyase OS=Drosophila melanogaster
GN=Sply PE=2 SV=1|e=0|from=172|to=1755|frame=1|Fragment_2
RGITKPNIVMPVTAHSGFDKAAQYLGLGVRTVRVDPETTLVDLKAMKRAINGNTCMLVAS
APNFPYGTMDDVQGVAELGLKYNIPVHVDACLGGFVIAFMRKAGFELPPFDFEVEGVTSI
SADTHKYGFAPKGSSVILYSDKKYRDHQFVVTTDWPGGVYGSPTVNGSRTGGIIAACWST
LLRFGETGYIDATKKIIDTTKYVEKEIRKIDEVFIFGKPATTVVALGSKVFDIYHLADKL
GGMGWSLNVLQFPSGIHICITHVHTQNGVADQFLDDMKSCIAEIMKNPSAPVEGKMAIYG
MAQSVPDRSTVTAFTKCYINSMYYTPADK
>Locus_1695_Transcript_7/7_Confidence_0.667_Length_2073|.|sp|Q9V7Y2|
SGPL_DROME Sphingosine-1-phosphate lyase OS=Drosophila melanogaster
GN=Sply PE=2 SV=1|e=0|from=172|to=1755|frame=1|Fragment_1
INARLASKEPWQIIAITATSVISFLWLHDLLTADEGIKTRLLKSLFKWAKKIPYVKKRID
DELNKISAQFDKDIQKRMQDVPYITTLPEQGLSDEEIVKMAKSYLNLGTYKWKEGRVSGA
VYYYDPELIELNTKIYGMTSYTNPLHPDVFPGVSKMEAEVVRITAKLFHGGPNACGTMTT
GGTESIIMACKAYRDYARE
>Locus_1695_Transcript_7/7_Confidence_0.667_Length_2073|.|sp|Q9V7Y2|
SGPL_DROME Sphingosine-1-phosphate lyase OS=Drosophila melanogaster
GN=Sply PE=2 SV=1|e=0|from=172|to=1755|frame=1|Fragment_2
RGITKPNIVMPVTAHSGFDKAAQYLGLGVRTVRVDPETTLVDLKAMKRAINGNTCMLVAS
APNFPYGTMDDVQGVAELGLKYNIPVHVDACLGGFVIAFMRKAGFELPPFDFEVEGVTSI
SADTHKYGFAPKGSSVILYSDKKYRDHQFVVTTDWPGGVYGSPTVNGSRTGGIIAACWST
LLRFGETGYIDATKKIIDTTKYVEKEIRKIDEVFIFGKPATTVVALGSKVFDIYHLADKL
GGMGWSLNVLQFPSGIHICITHVHTQNGVADQFLDDMKSCIAEIMKNPSAPVEGKMAIYG
MAQSVPDRSTVTAFTKCYINSMYYTPADK
>Locus_1695_Transcript_4/7_Confidence_0.667_Length_1894|.|sp|Q9V7Y2|
SGPL_DROME Sphingosine-1-phosphate lyase OS=Drosophila melanogaster
GN=Sply PE=2 SV=1|e=0|from=172|to=1755|frame=1|Fragment_1
INARLASKEPWQIIAITATSVISFLWLHDLLTADEGIKTRLLKSLFKWAKKIPYVKKRID
DELNKISAQFDKDIQKRMQDVPYITTLPEQGLSDEEIVKMAKSYLNLGTYKWKEGRVSGA
VYYYDPELIELNTKIYGMTSYTNPLHPDVFPGVSKMEAEVVRITAKLFHGGPNACGTMTT
GGTESIIMACKAYRDYARE
>Locus_1695_Transcript_4/7_Confidence_0.667_Length_1894|.|sp|Q9V7Y2|
SGPL_DROME Sphingosine-1-phosphate lyase OS=Drosophila melanogaster
GN=Sply PE=2 SV=1|e=0|from=172|to=1755|frame=1|Fragment_2
RGITKPNIVMPVTAHSGFDKAAQYLGLGVRTVRVDPETTLVDLKAMKRAINGNTCMLVAS

APNFPYGTMDDVQGVAELGLKYNIPVHVDACLGGFVIAFMRKAGFELPPFDFEVEGVTSI
SADTHKYGFAPKGSSVILYSDKKYRDHQFVVTTDWPGGVYGSPTVNGSRTGGIIAACWST
LLRFGETGYIDATKKIIDTTKYVEKEIRKIDEVFIFGKPATTVVALGSKVFDIYHLADKL
GGMGWSLNVLQFPSGIHICITHVHTQNGVADQFLDDMKSCIAEIMKNPSAPVEGKMAIYG
MAQSVPDRSTVTAFTKCYINSMYYTPADK
>Locus_4149_Transcript_2/4_Confidence_0.700_Length_2043|UNIGENE
REPRESENTATIVE TRANSCRIPT|sp|Q9VAF0|Y816_DROME Uncharacterized
protein CG7816 OS=Drosophila melanogaster GN=CG7816 PE=2 SV=1|
e=5.38886E-83|from=290|to=1429|frame=-3|Fragment_1
NSSIWESDSAFRFIYTDLVMEYIPEYFKDWQYHPWIF
>Locus_4149_Transcript_2/4_Confidence_0.700_Length_2043|UNIGENE
REPRESENTATIVE TRANSCRIPT|sp|Q9VAF0|Y816_DROME Uncharacterized
protein CG7816 OS=Drosophila melanogaster GN=CG7816 PE=2 SV=1|
e=5.38886E-83|from=290|to=1429|frame=-3|Fragment_2
L
>Locus_4149_Transcript_2/4_Confidence_0.700_Length_2043|UNIGENE
REPRESENTATIVE TRANSCRIPT|sp|Q9VAF0|Y816_DROME Uncharacterized
protein CG7816 OS=Drosophila melanogaster GN=CG7816 PE=2 SV=1|
e=5.38886E-83|from=290|to=1429|frame=-3|Fragment_3
K
>Locus_4149_Transcript_2/4_Confidence_0.700_Length_2043|UNIGENE
REPRESENTATIVE TRANSCRIPT|sp|Q9VAF0|Y816_DROME Uncharacterized
protein CG7816 OS=Drosophila melanogaster GN=CG7816 PE=2 SV=1|
e=5.38886E-83|from=290|to=1429|frame=-3|Fragment_4
LLRVLLSFAVGGLLGDVFLHLLPEAWENYSNKGERLVVMYEFVDSGGGSPSMTCGCWVLA
GLLLFTMVEKLFAGYMEDDDVSEEKTERKRKKMSKINCLIKNNNVLRARNENGRFSTSKC
SGGQNGKMCDIEVINGAIKNGCCMVEELEKEAVSNKS
>Locus_4149_Transcript_2/4_Confidence_0.700_Length_2043|UNIGENE
REPRESENTATIVE TRANSCRIPT|sp|Q9VAF0|Y816_DROME Uncharacterized
protein CG7816 OS=Drosophila melanogaster GN=CG7816 PE=2 SV=1|
e=5.38886E-83|from=290|to=1429|frame=-3|Fragment_5
KHVSGYLNLMANSIDNFTHGLAVGGSFIVSFKLGILTTFAILVHEIPHEVGDFAILLRSG
FSRWDAAR
>Locus_4149_Transcript_2/4_Confidence_0.700_Length_2043|UNIGENE
REPRESENTATIVE TRANSCRIPT|sp|Q9VAF0|Y816_DROME Uncharacterized
protein CG7816 OS=Drosophila melanogaster GN=CG7816 PE=2 SV=1|
e=5.38886E-83|from=290|to=1429|frame=-3|Fragment_6
GSRATSAMEARTCWIMPFTAGGFLHISLVTVLPDLLQERDPKESIKQFGALIGGIVLMAS
LTTLLE
>Locus_4149_Transcript_4/4_Confidence_0.600_Length_1962|.|sp|Q9VAF0|
Y816_DROME Uncharacterized protein CG7816 OS=Drosophila melanogaster
GN=CG7816 PE=2 SV=1|e=5.12996E-83|from=290|to=1429|frame=-3|
Fragment_1
NSSIWESDSAFRFIYTDLVMEYIPEYFKDWQYHPWIF
>Locus_4149_Transcript_4/4_Confidence_0.600_Length_1962|.|sp|Q9VAF0|
Y816_DROME Uncharacterized protein CG7816 OS=Drosophila melanogaster
GN=CG7816 PE=2 SV=1|e=5.12996E-83|from=290|to=1429|frame=-3|
Fragment_2
L
>Locus_4149_Transcript_4/4_Confidence_0.600_Length_1962|.|sp|Q9VAF0|
Y816_DROME Uncharacterized protein CG7816 OS=Drosophila melanogaster
GN=CG7816 PE=2 SV=1|e=5.12996E-83|from=290|to=1429|frame=-3|
Fragment_3
K

>Locus_4149_Transcript_4/4_Confidence_0.600_Length_1962|.|sp|Q9VAF0|
Y816_DROME Uncharacterized protein CG7816 OS=Drosophila melanogaster
GN=CG7816 PE=2 SV=1|e=5.12996E-83|from=290|to=1429|frame=-3|
Fragment_4
LLRVLLSFAVGGLLGDVFLHLLPEAWENYSNKGERLVVMYEFVDSGGGSPSMTCGCWVLA
GLLLFTMVEKLFAGYMEDDDVSEEKTERKRKKMSKINCLIKNNNVLRARNENGRFSTSKC
SGGQNGKMCDIEVINGAIKNGCCMVEELEKEAVSNKS
>Locus_4149_Transcript_4/4_Confidence_0.600_Length_1962|.|sp|Q9VAF0|
Y816_DROME Uncharacterized protein CG7816 OS=Drosophila melanogaster
GN=CG7816 PE=2 SV=1|e=5.12996E-83|from=290|to=1429|frame=-3|
Fragment_5
KHVSGYLNLMANSIDNFTHGLAVGGSFIVSFKLGILTTFAILVHEIPHEVGDFAILLRSG
FSRWDAAR
>Locus_4149_Transcript_4/4_Confidence_0.600_Length_1962|.|sp|Q9VAF0|
Y816_DROME Uncharacterized protein CG7816 OS=Drosophila melanogaster
GN=CG7816 PE=2 SV=1|e=5.12996E-83|from=290|to=1429|frame=-3|
Fragment_6
GSRATSAMEARTCWIMPFTAGGFLHISLVTVLPDLLQERDPKESIKQFGALIGGIVLMAS
LTTLLE
>Locus_4149_Transcript_3/4_Confidence_0.600_Length_1932|.|sp|Q9VAF0|
Y816_DROME Uncharacterized protein CG7816 OS=Drosophila melanogaster
GN=CG7816 PE=2 SV=1|e=1.57328E-84|from=290|to=1399|frame=-3|
Fragment_1
NSSIWESDSAFRFIYTDLVMEYIPEYFKDWQYHPWIF
>Locus_4149_Transcript_3/4_Confidence_0.600_Length_1932|.|sp|Q9VAF0|
Y816_DROME Uncharacterized protein CG7816 OS=Drosophila melanogaster
GN=CG7816 PE=2 SV=1|e=1.57328E-84|from=290|to=1399|frame=-3|
Fragment_2
L
>Locus_4149_Transcript_3/4_Confidence_0.600_Length_1932|.|sp|Q9VAF0|
Y816_DROME Uncharacterized protein CG7816 OS=Drosophila melanogaster
GN=CG7816 PE=2 SV=1|e=1.57328E-84|from=290|to=1399|frame=-3|
Fragment_3
K
>Locus_4149_Transcript_3/4_Confidence_0.600_Length_1932|.|sp|Q9VAF0|
Y816_DROME Uncharacterized protein CG7816 OS=Drosophila melanogaster
GN=CG7816 PE=2 SV=1|e=1.57328E-84|from=290|to=1399|frame=-3|
Fragment_4
LLRVLLSFAVGGLLGDVFLHLLPEAWENYSNKGDSGGGSPSMTCGCWVLAGLLLFTMVEK
LFAGYMEDDDVSEEKTERKRKKMSKINCLIKNNNVLRARNENGRFSTSKCSGGQNGKMCD
IEVINGAIKNGCCMVEELEKEAVSNKS
>Locus_4149_Transcript_3/4_Confidence_0.600_Length_1932|.|sp|Q9VAF0|
Y816_DROME Uncharacterized protein CG7816 OS=Drosophila melanogaster
GN=CG7816 PE=2 SV=1|e=1.57328E-84|from=290|to=1399|frame=-3|
Fragment_5
KHVSGYLNLMANSIDNFTHGLAVGGSFIVSFKLGILTTFAILVHEIPHEVGDFAILLRSG
FSRWDAAR
>Locus_4149_Transcript_3/4_Confidence_0.600_Length_1932|.|sp|Q9VAF0|
Y816_DROME Uncharacterized protein CG7816 OS=Drosophila melanogaster
GN=CG7816 PE=2 SV=1|e=1.57328E-84|from=290|to=1399|frame=-3|
Fragment_6
GSRATSAMEARTCWIMPFTAGGFLHISLVTVLPDLLQERDPKESIKQFGALIGGIVLMAS
LTTLLE
>Locus_4149_Transcript_1/4_Confidence_0.600_Length_1883|.|sp|Q9VAF0|

Y816_DROME Uncharacterized protein CG7816 OS=Drosophila melanogaster
GN=CG7816 PE=2 SV=1|e=4.90712E-83|from=290|to=1429|frame=-2|
Fragment_1
NSSIWESDSAFRFIYTDLVMEYIPEYFKDWQYHPWIF
>Locus_4149_Transcript_1/4_Confidence_0.600_Length_1883|.|sp|Q9VAF0|
Y816_DROME Uncharacterized protein CG7816 OS=Drosophila melanogaster
GN=CG7816 PE=2 SV=1|e=4.90712E-83|from=290|to=1429|frame=-2|
Fragment_2
L
>Locus_4149_Transcript_1/4_Confidence_0.600_Length_1883|.|sp|Q9VAF0|
Y816_DROME Uncharacterized protein CG7816 OS=Drosophila melanogaster
GN=CG7816 PE=2 SV=1|e=4.90712E-83|from=290|to=1429|frame=-2|
Fragment_3
K
>Locus_4149_Transcript_1/4_Confidence_0.600_Length_1883|.|sp|Q9VAF0|
Y816_DROME Uncharacterized protein CG7816 OS=Drosophila melanogaster
GN=CG7816 PE=2 SV=1|e=4.90712E-83|from=290|to=1429|frame=-2|
Fragment_4
LLRVLLSFAVGGLLGDVFLHLLPEAWENYSNKGERLVVMYEFVDSGGGSPSMTCGCWVLA
GLLLFTMVEKLFAGYMEDDDVSEEKTERKRKKMSKINCLIKNNNVLRARNENGRFSTSKC
SGGQNGKMCDIEVINGAIKNGCCMVEELEKEAVSNKS
>Locus_4149_Transcript_1/4_Confidence_0.600_Length_1883|.|sp|Q9VAF0|
Y816_DROME Uncharacterized protein CG7816 OS=Drosophila melanogaster
GN=CG7816 PE=2 SV=1|e=4.90712E-83|from=290|to=1429|frame=-2|
Fragment_5
KHVSGYLNLMANSIDNFTHGLAVGGSFIVSFKLGILTTFAILVHEIPHEVGDFAILLRSG
FSRWDAAR
>Locus_4149_Transcript_1/4_Confidence_0.600_Length_1883|.|sp|Q9VAF0|
Y816_DROME Uncharacterized protein CG7816 OS=Drosophila melanogaster
GN=CG7816 PE=2 SV=1|e=4.90712E-83|from=290|to=1429|frame=-2|
Fragment_6
GSRATSAMEARTCWIMPFTAGGFLHISLVTVLPDLLQERDPKESIKQFGALIGGIVLMAS
LTTLLE
>Locus_21525_Transcript_1/1_Confidence_1.000_Length_388|UNIGENE
REPRESENTATIVE TRANSCRIPT|sp|Q9VAP3|CTLH2_DROME CTL-like protein 2
OS=Drosophila melanogaster GN=CG11880 PE=3 SV=1|e=4.52959E-20|
from=4|to=189|frame=1|Fragment_1
PVILVVIGTFFITCVFFGVYDMAVDTLLLCFLEDSERNDGSPERPYYMSKELMKILGRRN
KV
>Locus_22736_Transcript_1/1_Confidence_1.000_Length_662|UNIGENE
REPRESENTATIVE TRANSCRIPT|sp|Q9VB11|UNC80_DROME Protein unc-80
homolog OS=Drosophila melanogaster GN=CG18437 PE=3 SV=4|
e=2.69553E-61|from=2|to=523|frame=2|Fragment_1
GRG
>Locus_22736_Transcript_1/1_Confidence_1.000_Length_662|UNIGENE
REPRESENTATIVE TRANSCRIPT|sp|Q9VB11|UNC80_DROME Protein unc-80
homolog OS=Drosophila melanogaster GN=CG18437 PE=3 SV=4|
e=2.69553E-61|from=2|to=523|frame=2|Fragment_2
DVAELHQNDISFLVTVVLNAMSPP
>Locus_22736_Transcript_1/1_Confidence_1.000_Length_662|UNIGENE
REPRESENTATIVE TRANSCRIPT|sp|Q9VB11|UNC80_DROME Protein unc-80
homolog OS=Drosophila melanogaster GN=CG18437 PE=3 SV=4|
e=2.69553E-61|from=2|to=523|frame=2|Fragment_3
SLTFTGSRETKRPARVRSTLYQAAFLALRVLCVCFEQRLSRDWSRIARVMRDLGRRNEAA

PELWSFLEFVVTHRTPLYIVLQPFLLHKISQPPIGDHERHMQFLIRERMRGTPPSASPRS
RG
>Locus_1706_Transcript_5/6_Confidence_0.583_Length_2041|UNIGENE
REPRESENTATIVE TRANSCRIPT|sp|Q9VC27|NICA_DROME Nicastrin
OS=Drosophila melanogaster GN=nct PE=1 SV=3|e=4.9653E-129|from=6|
to=1931|frame=-3|Fragment_1
INNIYVFIILVISFIYFCHGERTRDKMYVPITGASCIRRLNATHQTGCTSARQGSVGALH
AIHSESDFEFVLNSGTSPPYAVLMPPIFFNDHNMMQLKESA
>Locus_1706_Transcript_5/6_Confidence_0.583_Length_2041|UNIGENE
REPRESENTATIVE TRANSCRIPT|sp|Q9VC27|NICA_DROME Nicastrin
OS=Drosophila melanogaster GN=nct PE=1 SV=3|e=4.9653E-129|from=6|
to=1931|frame=-3|Fragment_2
RVTAVIVTNNTESLNAFSTESKCPNQFIGKHD
>Locus_1706_Transcript_5/6_Confidence_0.583_Length_2041|UNIGENE
REPRESENTATIVE TRANSCRIPT|sp|Q9VC27|NICA_DROME Nicastrin
OS=Drosophila melanogaster GN=nct PE=1 SV=3|e=4.9653E-129|from=6|
to=1931|frame=-3|Fragment_3
EYCDASDPLKSWNQYGSNLMHQDFPFPILFISDEEQINLLYKCFEKFNNFDIEKQFQRSL
CSVELSSFMAAAANSKVCHRRSELSKNLSPVKFCDPLGDKNIFATLFPRPYV
>Locus_1706_Transcript_5/6_Confidence_0.583_Length_2041|UNIGENE
REPRESENTATIVE TRANSCRIPT|sp|Q9VC27|NICA_DROME Nicastrin
OS=Drosophila melanogaster GN=nct PE=1 SV=3|e=4.9653E-129|from=6|
to=1931|frame=-3|Fragment_4
NTVFEYKTELNGMKHYTDDNSVVDKFTEKKIQKSLTKTEEREKLILLMARIDTTSMFDGI
APGAMDSLLSFVTLLSTANMLNYLLVRNNIKKSYD
>Locus_1706_Transcript_5/6_Confidence_0.583_Length_2041|UNIGENE
REPRESENTATIVE TRANSCRIPT|sp|Q9VC27|NICA_DROME Nicastrin
OS=Drosophila melanogaster GN=nct PE=1 SV=3|e=4.9653E-129|from=6|
to=1931|frame=-3|Fragment_5
KNIFYLLLNGESYDYIGSQRLLYDIEKGSFPPKQDN
>Locus_1706_Transcript_5/6_Confidence_0.583_Length_2041|UNIGENE
REPRESENTATIVE TRANSCRIPT|sp|Q9VC27|NICA_DROME Nicastrin
OS=Drosophila melanogaster GN=nct PE=1 SV=3|e=4.9653E-129|from=6|
to=1931|frame=-3|Fragment_6
IVFEDFDMIIEIGSLGGKPNYLSSHYAQNNPKIDNFLQNMKTYSEKNKFG
>Locus_1706_Transcript_5/6_Confidence_0.583_Length_2041|UNIGENE
REPRESENTATIVE TRANSCRIPT|sp|Q9VC27|NICA_DROME Nicastrin
OS=Drosophila melanogaster GN=nct PE=1 SV=3|e=4.9653E-129|from=6|
to=1931|frame=-3|Fragment_7
ITFHSSQRLPPSSIRTFYSNDNIKAPAMVLTDFDDKFVNPYYHSVYDSASNIKYQYINAS
EAADDLSKLMNLSNFGDFNPDSVQILVRNISTTLGMALYQEITNMSLTGELGTNPYLIDE
LLHCYLESADCIAFRAVSPPNKNNVSPTPPYRYVGVAGYINDATIWTARVLAFLTGSSSN
RTEED
>Locus_1706_Transcript_5/6_Confidence_0.583_Length_2041|UNIGENE
REPRESENTATIVE TRANSCRIPT|sp|Q9VC27|NICA_DROME Nicastrin
OS=Drosophila melanogaster GN=nct PE=1 SV=3|e=4.9653E-129|from=6|
to=1931|frame=-3|Fragment_8
CDFLPYSWVAGFNGLG
>Locus_1706_Transcript_1/6_Confidence_0.583_Length_2033|.|sp|Q9VC27|
NICA_DROME Nicastrin OS=Drosophila melanogaster GN=nct PE=1 SV=3|
e=6.4617E-129|from=1|to=1923|frame=-3|Fragment_1
INNIYVFIILVISFIYFCHGERTRDKMYVPITGASCIRRLNATHQTGCTSARQGSVGALH
AIHSESDFEFVLNSGTSPPYAVLMPPIFFNDHNMMQLKESA
>Locus_1706_Transcript_1/6_Confidence_0.583_Length_2033|.|sp|Q9VC27|

NICA_DROME Nicastrin OS=Drosophila melanogaster GN=nct PE=1 SV=3|
e=6.4617E-129|from=1|to=1923|frame=-3|Fragment_2
RVTAVIVTNNTESLNAFSTESKCPNQFIGKHD
>Locus_1706_Transcript_1/6_Confidence_0.583_Length_2033|.|sp|Q9VC27|
NICA_DROME Nicastrin OS=Drosophila melanogaster GN=nct PE=1 SV=3|
e=6.4617E-129|from=1|to=1923|frame=-3|Fragment_3
EYCDASDPLKSWNQYGSNLMHQDFPFPILFISDEEQINLLYKCFEKFNNFDIEKQFQRSL
CSVELSSFMAAAANSKVCHRRSELSKNLSPVKFCDPLGDKNIFATLFPRPYV
>Locus_1706_Transcript_1/6_Confidence_0.583_Length_2033|.|sp|Q9VC27|
NICA_DROME Nicastrin OS=Drosophila melanogaster GN=nct PE=1 SV=3|
e=6.4617E-129|from=1|to=1923|frame=-3|Fragment_4
NTVFEYKTELNGMKHYTDDNSVVDKFTEKKIQKSLTKTEEREKLILLMARIDTTSMFDGI
APGAMDSLLSFVTLLSTANMLNYLLVRNNIKKSYD
>Locus_1706_Transcript_1/6_Confidence_0.583_Length_2033|.|sp|Q9VC27|
NICA_DROME Nicastrin OS=Drosophila melanogaster GN=nct PE=1 SV=3|
e=6.4617E-129|from=1|to=1923|frame=-3|Fragment_5
KNIFYLLLNGESYDYIGSQRLLYDIEKGSFPPKQDN
>Locus_1706_Transcript_1/6_Confidence_0.583_Length_2033|.|sp|Q9VC27|
NICA_DROME Nicastrin OS=Drosophila melanogaster GN=nct PE=1 SV=3|
e=6.4617E-129|from=1|to=1923|frame=-3|Fragment_6
IVFEDFDMIIEIGSLGGKPNYLSSHYAQNNPKIDNFLQNMKTYSEKNKFG
>Locus_1706_Transcript_1/6_Confidence_0.583_Length_2033|.|sp|Q9VC27|
NICA_DROME Nicastrin OS=Drosophila melanogaster GN=nct PE=1 SV=3|
e=6.4617E-129|from=1|to=1923|frame=-3|Fragment_7
ITFHSSQRLPPSSIRTFYSNDNIKAPAMVLTDFDDKFVNPYYHSVYDSASNIKYQYINAS
EAADDLSKLMNLNNFVDFNPDSVQILVRNISTTLGMALYQEITNMSLTGELGTNPYLIDE
LLHCYLESADCIAFRAVSPPNKNNVSPTPPYRYVGVAGYINDATIWTARVLAFLTGSSSN
RTEED
>Locus_1706_Transcript_1/6_Confidence_0.583_Length_2033|.|sp|Q9VC27|
NICA_DROME Nicastrin OS=Drosophila melanogaster GN=nct PE=1 SV=3|
e=6.4617E-129|from=1|to=1923|frame=-3|Fragment_8
CDFLPYSWVAGFNGL
>Locus_1706_Transcript_6/6_Confidence_0.583_Length_2033|.|sp|Q9VC27|
NICA_DROME Nicastrin OS=Drosophila melanogaster GN=nct PE=1 SV=3|
e=2.4555E-128|from=1|to=1923|frame=-3|Fragment_1
INNIYVFIILVISFIYFCHGERTRDKMYVPITGASCIRRLNATHQTGCTSARQGSVGALH
AIHSESDFEFVLNSGTSPPYAVLMPPIFFNDHNMMQLKESA
>Locus_1706_Transcript_6/6_Confidence_0.583_Length_2033|.|sp|Q9VC27|
NICA_DROME Nicastrin OS=Drosophila melanogaster GN=nct PE=1 SV=3|
e=2.4555E-128|from=1|to=1923|frame=-3|Fragment_2
RVTAVIVTNNTESLNAFSTESKCPNQFIGKHD
>Locus_1706_Transcript_6/6_Confidence_0.583_Length_2033|.|sp|Q9VC27|
NICA_DROME Nicastrin OS=Drosophila melanogaster GN=nct PE=1 SV=3|
e=2.4555E-128|from=1|to=1923|frame=-3|Fragment_3
EYCDASDPLKSWNQYGSNLMHQDFPFPILFISDEEQINLLYKCFEKFNNFDIEKQFQRSL
CSVELSSFMAAAANSKVCHRRSELSKNLSPVKFCDPLGDKNIFATLFPRPYV
>Locus_1706_Transcript_6/6_Confidence_0.583_Length_2033|.|sp|Q9VC27|
NICA_DROME Nicastrin OS=Drosophila melanogaster GN=nct PE=1 SV=3|
e=2.4555E-128|from=1|to=1923|frame=-3|Fragment_4
NTVFEYKTELNGMKHYTDDNSVVDKFTEKKIQKSLTKTEEREKLILLMARIDTTSMFDGI
APGAMDSLLSFVTLLSTANMLNYLLVRNNIKKSYD
>Locus_1706_Transcript_6/6_Confidence_0.583_Length_2033|.|sp|Q9VC27|
NICA_DROME Nicastrin OS=Drosophila melanogaster GN=nct PE=1 SV=3|
e=2.4555E-128|from=1|to=1923|frame=-3|Fragment_5

KNIFYLLLNGESYDYIGSQRLLYDIEKGSFPPKQDN
>Locus_1706_Transcript_6/6_Confidence_0.583_Length_2033|.|sp|Q9VC27|NICA_DROME Nicastrin OS=Drosophila melanogaster GN=nct PE=1 SV=3|e=2.4555E-128|from=1|to=1923|frame=-3|Fragment_6
IVFEDFDMIIEIGSLGGKPNYLSSHYAQNNPKIDNFLQNMKTYSEKNKFG
>Locus_1706_Transcript_6/6_Confidence_0.583_Length_2033|.|sp|Q9VC27|NICA_DROME Nicastrin OS=Drosophila melanogaster GN=nct PE=1 SV=3|e=2.4555E-128|from=1|to=1923|frame=-3|Fragment_7
ITFHSSQRLPPSSIRTFYSNDNIKAPAMVLTDFDDKFVNPYYHSVYDSASNIKYQYINAS
EAADDLSKLMNLSNFGDFNPDSVQILVRNISTTLGMALYQEITNMSLTGELGTNPYLIDE
LLHCYLESADCIAFRAVSPPNKNNVSPTPPYRYVGVAGYINDATIWTARVLAFLTGSSSN
RTEED
>Locus_1706_Transcript_6/6_Confidence_0.583_Length_2033|.|sp|Q9VC27|NICA_DROME Nicastrin OS=Drosophila melanogaster GN=nct PE=1 SV=3|e=2.4555E-128|from=1|to=1923|frame=-3|Fragment_8
CDFLPYSWVAGFNGL
>Locus_1706_Transcript_4/6_Confidence_0.583_Length_1794|.|sp|Q9VC27|NICA_DROME Nicastrin OS=Drosophila melanogaster GN=nct PE=1 SV=3|e=7.0742E-116|from=1|to=1794|frame=-1|Fragment_1
HQTGCTSARQGSVGALHAIHSESDFEFVLNSGTSPPYAVLMPPIFFNDHNMMQLKESA
>Locus_1706_Transcript_4/6_Confidence_0.583_Length_1794|.|sp|Q9VC27|NICA_DROME Nicastrin OS=Drosophila melanogaster GN=nct PE=1 SV=3|e=7.0742E-116|from=1|to=1794|frame=-1|Fragment_2
RVTAVIVTNNTESLNAFSTESKCPNQFIGKHD
>Locus_1706_Transcript_4/6_Confidence_0.583_Length_1794|.|sp|Q9VC27|NICA_DROME Nicastrin OS=Drosophila melanogaster GN=nct PE=1 SV=3|e=7.0742E-116|from=1|to=1794|frame=-1|Fragment_3
EYCDASDPLKSWNQYGSNLMHQDFPFPILFISDEEQINLLYKCFEKFNNFDIEKQFQRSL
CSVELSSFMAAAANSKVCHRRSELSKNLSPVKFCDPLGDKNIFATLFPRPYV
>Locus_1706_Transcript_4/6_Confidence_0.583_Length_1794|.|sp|Q9VC27|NICA_DROME Nicastrin OS=Drosophila melanogaster GN=nct PE=1 SV=3|e=7.0742E-116|from=1|to=1794|frame=-1|Fragment_4
NTVFEYKTELNGMKHYTDDNSVVDKFTEKKIQKSLTKTEEREKLILLMARIDTTSMFDGI
APGAMDSLLSFVTLLSTANMLNYLLVRNNIKKSYD
>Locus_1706_Transcript_4/6_Confidence_0.583_Length_1794|.|sp|Q9VC27|NICA_DROME Nicastrin OS=Drosophila melanogaster GN=nct PE=1 SV=3|e=7.0742E-116|from=1|to=1794|frame=-1|Fragment_5
KNIFYLLLNGESYDYIGSQRLLYDIEKGSFPPKQDN
>Locus_1706_Transcript_4/6_Confidence_0.583_Length_1794|.|sp|Q9VC27|NICA_DROME Nicastrin OS=Drosophila melanogaster GN=nct PE=1 SV=3|e=7.0742E-116|from=1|to=1794|frame=-1|Fragment_6
IVFEDFDMIIEIGSLGGKPNYLSSHYAQNNPKIDNFLQNMKTYSEKNKFGITFH
>Locus_1706_Transcript_4/6_Confidence_0.583_Length_1794|.|sp|Q9VC27|NICA_DROME Nicastrin OS=Drosophila melanogaster GN=nct PE=1 SV=3|e=7.0742E-116|from=1|to=1794|frame=-1|Fragment_7
SSQRLPPSSIRTFYSNDNIKAPAMVLTDFDDKFVNPYYHSVYDSASNIKYQYINASEAAD
DLSKLMNLSNFGDFNPDSVQILVRNISTTLGMALYQEITNMSLTGELGTNPYLIDELLHC
YLESADCIAFRAVSPPNKNNVSPTPPYRYVGVAGYINDATIWTARVLAFLTGSSSNRTEE
D
>Locus_1706_Transcript_4/6_Confidence_0.583_Length_1794|.|sp|Q9VC27|NICA_DROME Nicastrin OS=Drosophila melanogaster GN=nct PE=1 SV=3|e=7.0742E-116|from=1|to=1794|frame=-1|Fragment_8
CDFLPYSWVAGFNGL
>Locus_1706_Transcript_2/6_Confidence_0.500_Length_1353|.|sp|Q9VC27|

NICA_DROME Nicastrin OS=Drosophila melanogaster GN=nct PE=1 SV=3|
e=2.20251E-71|from=1|to=1353|frame=-1|Fragment_1
RSLCSVELSSFMAAAANSEVCHRRSELSKNLSPVKFCDPLGDKNIFATLFPRPYVNASDN
NIDDSLNASNNTVFEYKTELNGMKHYTDDNSVVDKFTEKKIQKSLTKTEEREKLILLMAR
IDTTSMFDGIAPGAMDSLLSFVTLLSTANMLNYLLVRNNIKKSYD
>Locus_1706_Transcript_2/6_Confidence_0.500_Length_1353|.|sp|Q9VC27|
NICA_DROME Nicastrin OS=Drosophila melanogaster GN=nct PE=1 SV=3|
e=2.20251E-71|from=1|to=1353|frame=-1|Fragment_2
KNIFYLLLNGESYDYIGSQRLLYDIEKGSFPPKQDN
>Locus_1706_Transcript_2/6_Confidence_0.500_Length_1353|.|sp|Q9VC27|
NICA_DROME Nicastrin OS=Drosophila melanogaster GN=nct PE=1 SV=3|
e=2.20251E-71|from=1|to=1353|frame=-1|Fragment_3
IVFEDFDMIIEIGSLGGKPNYLSSHYAQNNPKIDNFLQNMKTYSEKNKFGITFH
>Locus_1706_Transcript_2/6_Confidence_0.500_Length_1353|.|sp|Q9VC27|
NICA_DROME Nicastrin OS=Drosophila melanogaster GN=nct PE=1 SV=3|
e=2.20251E-71|from=1|to=1353|frame=-1|Fragment_4
SSQRLPPSSIRTFYSNDNIKAPAMVLTDFDDKFVNPYYHSVYDSASNIKYQYINASEAAD
DLSKLMNLSNFGDFNPDSVQILVRNISTTLGMALYQEITNMSLTGELGTNPYLIDELLHC
YLESADCIAFRAVSPPNKNNVSPTPPYRYVGVAGYINDATIWTARVLAFLTGSSSNRTEE
D
>Locus_1706_Transcript_2/6_Confidence_0.500_Length_1353|.|sp|Q9VC27|
NICA_DROME Nicastrin OS=Drosophila melanogaster GN=nct PE=1 SV=3|
e=2.20251E-71|from=1|to=1353|frame=-1|Fragment_5
CDFLPYSWVAGFNGL
>Locus_1706_Transcript_3/6_Confidence_0.417_Length_873|.|sp|Q9VC27|
NICA_DROME Nicastrin OS=Drosophila melanogaster GN=nct PE=1 SV=3|
e=4.58821E-47|from=1|to=855|frame=-1|Fragment_1
NIFYLLLNGESYDYIGSQRLLYDIEKGSFPPKQEN
>Locus_1706_Transcript_3/6_Confidence_0.417_Length_873|.|sp|Q9VC27|
NICA_DROME Nicastrin OS=Drosophila melanogaster GN=nct PE=1 SV=3|
e=4.58821E-47|from=1|to=855|frame=-1|Fragment_2
IVFEDFDMIIEIGSLGGKPNFLSSHYAQNNPKIDNFLQNMKTYSEKNKFGITFH
>Locus_1706_Transcript_3/6_Confidence_0.417_Length_873|.|sp|Q9VC27|
NICA_DROME Nicastrin OS=Drosophila melanogaster GN=nct PE=1 SV=3|
e=4.58821E-47|from=1|to=855|frame=-1|Fragment_3
SSQRLPPSSIRTFYSNDNIKAPAMVLTDFDDKFVNPYYHSVYDSASNIKYQYINASEAAD
DLSKLMNLSNFGDFNPDSVQILVRNISTTLGMALYQEITNMSLTGELGTNPYLIDELLHC
YLESADCIAFRAVSPPNKNNVSPTPPYRYVGVAGYINDATIWTARVLAFLTGSSSNRTEE
D
>Locus_1706_Transcript_3/6_Confidence_0.417_Length_873|.|sp|Q9VC27|
NICA_DROME Nicastrin OS=Drosophila melanogaster GN=nct PE=1 SV=3|
e=4.58821E-47|from=1|to=855|frame=-1|Fragment_4
CDFLPYSWVAGFNGL

>Locus_5652_Transcript_1/9_Confidence_0.481_Length_2048|UNIGENE
REPRESENTATIVE TRANSCRIPT|sp|Q9VC57|ATLAS_DROME Atlastin
OS=Drosophila melanogaster GN=atl PE=1 SV=1|e=0|from=123|to=1721|
frame=-1|Fragment_1
GHPVQIVRTLSDHTFELDEAALSEILNRPELKDRAAVVVSVAGAFRKGKSFLLDFFLRYM
NSKYVNRESSGDWLGAEDAPLSGFSWRGGSERDTTGILMWSEPFLHESANGQKLAVLLLD
TQGTFDSESTVKDCATVFALSTMLSSVQIYNLTQNIQEDDLQHLHLFTEYGRLALADSGQ
KPFQRLQFLVRDWSFPYEEQYGAVGGQNLLQRRMKISERQHPELQSLRKHIASCFTEVAC
FLMPHPGLTVATNPNFDGRLTDITPEFKDSLKVFIPMLLAPENLIAKRINGVPVKCRDLV
QYFKAYMSIYKGNELPEPKSMLVATAEANNLSAVAEAKDMYTQLMEDICGGSKPYLSTAH

LESEHLRIRDKAVGTFIQKRKMGGSAFSENYRIKLEADLDEAFEQFKGHNDSKNIFKAAR
TPAVFFALLILMYVLSGVFGLVGLYAISNICNFAMGVALLTLVLWAYIRYSGELTDIGVT
IDEIANMIWINAMKPIYESCMEKSIQHV
>Locus_5652_Transcript_1/9_Confidence_0.481_Length_2048|UNIGENE
REPRESENTATIVE TRANSCRIPT|sp|Q9VC57|ATLAS_DROME Atlastin
OS=Drosophila melanogaster GN=atl PE=1 SV=1|e=0|from=123|to=1721|
frame=-1|Fragment_2
TNQAFTAGATTALNSTH
>Locus_5652_Transcript_1/9_Confidence_0.481_Length_2048|UNIGENE
REPRESENTATIVE TRANSCRIPT|sp|Q9VC57|ATLAS_DROME Atlastin
OS=Drosophila melanogaster GN=atl PE=1 SV=1|e=0|from=123|to=1721|
frame=-1|Fragment_3
ANGKVKQS
>Locus_5652_Transcript_2/9_Confidence_0.556_Length_2048|.|sp|Q9VC57|
ATLAS_DROME Atlastin OS=Drosophila melanogaster GN=atl PE=1 SV=1|
e=0|from=123|to=1721|frame=-1|Fragment_1
GHPVQIVRTLSDHTFELDEAALSEILNRPELKDRAAVVVSVAGAFRKGKSFLLDFFLRYM
NSKYVNRETAGDWLGAEDAPLSGFSWRGGSERDTTGILMWSEPFLHESANGQKLAVLLLD
TQGTFDSESTVKDCATVFALSTMLSSVQIYNLTQNIQEDDLQHLHLFTEYGRLALADSGQ
KPFQRLQFLVRDWSFPYEEQYGAVGGQNLLQRRMKISERQHPELQSLRKHIASCFTEVAC
FLMPHPGLTVATNPNFDGRLTDITPEFKDSLKVFIPMLLAPENLIAKRINGVPVKCRDLV
QYFKAYMSIYKGNELPEPKSMLVATAEANNLSAVAEAKDMYTQLMEDICGGSKPYLSTAH
LESEHLRIRDKAVGTFIQKRKMGGSAFSENYRIKLEADLDEAFEQFKGHNDSKNIFKAAR
TPAVFFALLILMYVLSGVFGLVGLYAISNICNFAMGVALLTLVLWAYIRYSGELTDIGVT
IDEIANMIWINAMKPIYESCMEKSIQHV
>Locus_5652_Transcript_2/9_Confidence_0.556_Length_2048|.|sp|Q9VC57|
ATLAS_DROME Atlastin OS=Drosophila melanogaster GN=atl PE=1 SV=1|
e=0|from=123|to=1721|frame=-1|Fragment_2
TNQAFTAGATTALNSTH
>Locus_5652_Transcript_2/9_Confidence_0.556_Length_2048|.|sp|Q9VC57|
ATLAS_DROME Atlastin OS=Drosophila melanogaster GN=atl PE=1 SV=1|
e=0|from=123|to=1721|frame=-1|Fragment_3
ANGKVKQS
>Locus_5652_Transcript_3/9_Confidence_0.593_Length_2048|.|sp|Q9VC57|
ATLAS_DROME Atlastin OS=Drosophila melanogaster GN=atl PE=1 SV=1|
e=0|from=123|to=1721|frame=-1|Fragment_1
GHPVQIVRTLSDHTFELDEAALSEILNRPELKDRAAVVVSVAGAFRKGKSFLLDFFLRYM
NSKYVNRETAGDWLGAEDAPLSGFSWRGGSERDTTGILMWSEPFLHESANGQKLAVLLLD
TQGTFDSESTVKDCATVFALSTMLSSVQIYNLTQNIQEDDLQHLHLFTEYGRLALADSGQ
KPFQRLQFLVRDWSFPYEEQYGAVGGQNLLQRRMKISERQHPELQSLRKHIASCFTEVAC
FLMPHPGLTVATNPNFDGRLTDITPEFKDSLKVFIPMLLAPENLIAKRINGVPVKCRDLV
QYFKAYMSIYKGNELPEPKSMLVATAEANNLSAVAEAKDMYTQLMEDICGGSKPYLSTAH
LESEHLRIRDKAVGTFIQKRKMGGSAFSENYRIKLEADLDEAFEQFKGHNDSKNIFKAAR
TPAVFFALLILMYVLSGVFGLVGLYAISNICNFAMGVALLTLVLWAYIRYSGELTDIGVT
IDEIANMIWINAMKPIYESCMEKSIQHV
>Locus_5652_Transcript_3/9_Confidence_0.593_Length_2048|.|sp|Q9VC57|
ATLAS_DROME Atlastin OS=Drosophila melanogaster GN=atl PE=1 SV=1|
e=0|from=123|to=1721|frame=-1|Fragment_2
TNQAFTAGATTALNSTH
>Locus_5652_Transcript_3/9_Confidence_0.593_Length_2048|.|sp|Q9VC57|
ATLAS_DROME Atlastin OS=Drosophila melanogaster GN=atl PE=1 SV=1|
e=0|from=123|to=1721|frame=-1|Fragment_3
ANGKVKQS
>Locus_5652_Transcript_5/9_Confidence_0.593_Length_2048|.|sp|Q9VC57|

ATLAS_DROME Atlastin OS=Drosophila melanogaster GN=atl PE=1 SV=1|
e=0|from=123|to=1721|frame=-1|Fragment_1
GHPVQIVRTLSDHTFELDEAALSEILNRPELKDRAAVVVSVAGAFRKGKSFLLDFFLRYM
NSKYVNRESSGDWLGAEDAPLSGFSWRGGSERDTTGILMWSEPFLHESANGQKLAVLLLD
TQGTFDSESTVKDCATVFALSTMLSSVQIYNLTQNIQEDDLQHLHLFTEYGRLALADSGQ
KPFQRLQFLVRDWSFPYEEQYGAVGGQNLLQRRMKISERQHPELQSLRKHIASCFTEVAC
FLMPHPGLTVATNPNFDGRLTDITPEFKDSLKVFIPMLLAPENLIAKRINGVPVKCRDLV
QYFKAYMSIYKGNELPEPKSMLVATAEANNLSAVAEAKDMYTQLMEDICGGSKPYLSTAH
LESEHLRIRDKAVGTFIQKRKMGGSAFSENYRIKLEADLDEAFEQFKGHNDSKNIFKAAR
TPAVFFALLILMYVLSGVFGLVGLYAISNICNFAMGVALLTLVLWAYIRYSGELTDIGVT
IDEIANMIWINAMKPIYESCMEKSIQHV
>Locus_5652_Transcript_5/9_Confidence_0.593_Length_2048|.|sp|Q9VC57|
ATLAS_DROME Atlastin OS=Drosophila melanogaster GN=atl PE=1 SV=1|
e=0|from=123|to=1721|frame=-1|Fragment_2
TNQAFTAGATTALNSTH
>Locus_5652_Transcript_5/9_Confidence_0.593_Length_2048|.|sp|Q9VC57|
ATLAS_DROME Atlastin OS=Drosophila melanogaster GN=atl PE=1 SV=1|
e=0|from=123|to=1721|frame=-1|Fragment_3
ANGKVKQS
>Locus_5652_Transcript_7/9_Confidence_0.556_Length_2048|.|sp|Q9VC57|
ATLAS_DROME Atlastin OS=Drosophila melanogaster GN=atl PE=1 SV=1|
e=0|from=123|to=1721|frame=-1|Fragment_1
GHPVQIVRTLSDHTFELDEAALSEILNRPELKDRAAVVVSVAGAFRKGKSFLLDFFLRYM
NSKYVNRESSGDWLGAEDAPLSGFSWRGGSERDTTGILMWSEPFLHESANGQKLAVLLLD
TQGTFDSESTVKDCATVFALSTMLSSVQIYNLTQNIQEDDLQHLHLFTEYGRLALADSGQ
KPFQRLQFLVRDWSFPYEEQYGAVGGQNLLQRRMKISERQHPELQSLRKHIASCFTEVAC
FLMPHPGLTVATNPNFDGRLTDITPEFKDSLKVFIPMLLAPENLIAKRINGVPVKCRDLV
QYFKAYMSIYKGNELPEPKSMLVATAEANNLSAVAEAKDMYTQLMEDICGGSKPYLSTAH
LESEHLRIRDKAVGTFIQKRKMGGSAFSENYRIKLEADLDEAFEQFKGHNDSKNIFKAAR
TPAVFFALLILMYVLSGVFGLVGLYAISNICNFAMGVALLTLVLWAYIRYSGELTDIGVT
IDEIANMIWINAMKPIYESCMEKSIQHV
>Locus_5652_Transcript_7/9_Confidence_0.556_Length_2048|.|sp|Q9VC57|
ATLAS_DROME Atlastin OS=Drosophila melanogaster GN=atl PE=1 SV=1|
e=0|from=123|to=1721|frame=-1|Fragment_2
TNQAFTAGATTALNSTH
>Locus_5652_Transcript_7/9_Confidence_0.556_Length_2048|.|sp|Q9VC57|
ATLAS_DROME Atlastin OS=Drosophila melanogaster GN=atl PE=1 SV=1|
e=0|from=123|to=1721|frame=-1|Fragment_3
ANGKVKQS
>Locus_5652_Transcript_8/9_Confidence_0.556_Length_2048|.|sp|Q9VC57|
ATLAS_DROME Atlastin OS=Drosophila melanogaster GN=atl PE=1 SV=1|
e=0|from=123|to=1721|frame=-1|Fragment_1
GHPVQIVRTLSDHTFELDEAALSEILNRPELKDRAAVVVSVAGAFRKGKSFLLDFFLRYM
NSKYVNRESSGDWLGAEDAPLSGFSWRGGSERDTTGILMWSEPFLHESANGQKLAVLLLD
TQGTFDSESTVKDCATVFALSTMLSSVQIYNLTQNIQEDDLQHLHLFTEYGRLALADSGQ
KPFQRLQFLVRDWSFPYEEQYGAVGGQNLLQRRMKISERQHPELQSLRKHIASCFTEVAC
FLMPHPGLTVATNPNFDGRLTDITPEFKDSLKVFIPMLLAPENLIAKRINGVPVKCRDLV
QYFKAYMSIYKGNELPEPKSMLVATAEANNLSAVAEAKDMYTQLMEDICGGSKPYLSTAH
LESEHLRIRDKAVGTFIQKRKMGGSAFSENYRIKLEADLDEAFEQFKGHNDSKNIFKAAR
TPAVFFALLILMYVLSGVFGLVGLYAISNICNFAMGVALLTLVLWAYIRYSGELTDIGVT
IDEIANMIWINAMKPIYESCMEKSIQHV
>Locus_5652_Transcript_8/9_Confidence_0.556_Length_2048|.|sp|Q9VC57|
ATLAS_DROME Atlastin OS=Drosophila melanogaster GN=atl PE=1 SV=1|
e=0|from=123|to=1721|frame=-1|Fragment_2

TNQAFTAGATTALNSTH
>Locus_5652_Transcript_8/9_Confidence_0.556_Length_2048|.|sp|Q9VC57|
ATLAS_DROME Atlastin OS=Drosophila melanogaster GN=atl PE=1 SV=1|
e=0|from=123|to=1721|frame=-1|Fragment_3
ANGKVKQS
>Locus_5652_Transcript_9/9_Confidence_0.556_Length_2048|.|sp|Q9VC57|
ATLAS_DROME Atlastin OS=Drosophila melanogaster GN=atl PE=1 SV=1|
e=0|from=123|to=1721|frame=-1|Fragment_1
GHPVQIVRTLSDHTFELDEAALSEILNRPELKDRAAVVVSVAGAFRKGKSFLLDFFLRYM
NSKYVNRESSGDWLGAEDAPLSGFSWRGGSERDTTGILMWSEPFLHESANGQKLAVLLLD
TQGTFDSESTVKDCATVFALSTMLSSVQIYNLTQNIQEDDLQHLHLFTEYGRLALADSGQ
KPFQRLQFLVRDWSFPYEEQYGAVGGQNLLQRRMKISERQHPELQSLRKHIASCFTEVAC
FLMPHPGLTVATNPNFDGRLTDITPEFKDSLKVFIPMLLAPENLIAKRINGVPVKCRDLV
QYFKAYMSIYKGNELPEPKSMLVATAEANNLSAVAEAKDMYTQLMEDICGGSKPYLSTAH
LESEHLRIRDKAVGTFIQKRKMGGSAFSENYRIKLEADLDEAFEQFKGHNDSKNIFKAAR
TPAVFFALLILMYVLSGVFGLVGLYAISNICNFAMGVALLTLVLWAYIRYSGELTDIGVT
IDEIANMIWINAMKPIYESCMEKSIQHV
>Locus_5652_Transcript_9/9_Confidence_0.556_Length_2048|.|sp|Q9VC57|
ATLAS_DROME Atlastin OS=Drosophila melanogaster GN=atl PE=1 SV=1|
e=0|from=123|to=1721|frame=-1|Fragment_2
TNQAFTAGATTALNSTH
>Locus_5652_Transcript_9/9_Confidence_0.556_Length_2048|.|sp|Q9VC57|
ATLAS_DROME Atlastin OS=Drosophila melanogaster GN=atl PE=1 SV=1|
e=0|from=123|to=1721|frame=-1|Fragment_3
ANGKVKQS
>Locus_5652_Transcript_4/9_Confidence_0.519_Length_1850|.|sp|Q9VC57|
ATLAS_DROME Atlastin OS=Drosophila melanogaster GN=atl PE=1 SV=1|
e=0|from=3|to=1523|frame=-1|Fragment_1
GHPVQIVRTLSDHTFELDEAALSEILNRPELKDRAAVVVSVAGAFRKGKSFLLDFFLRYM
NSKYVNRESSGDWLGAEDAPLSGFSWRGGSERDTTGILMWSEPFLHESANGQKLAVLLLD
TQGTFDSESTVKDCATVFALSTMLSSVQIYNLTQNIQEDDLQHLHLFTEYGRLALADSGQ
KPFQRLQFLVRDWSFPYEEQYGAVGGQNLLQRRMKISERQHPELQSLRKHIASCFTEVAC
FLMPHPGLTVATNPNFDGRLTDITPEFKDSLKVFIPMLLAPENLIAKRINGVPVKCRDLV
QYFKAYMSIYKGNELPEPKSMLVATAEANNLSAVAEAKDMYTQLMEDICGGSKPYLSTAH
LESEHLRIRDKAVGTFIQKRKMGGSAFSENYRIKLEADLDEAFEQFKGHNDSKNIFKAAR
TPAVFFALLILMYVLSGVFGLVGLYAISNICNFAMGVALLTLVLWAYIRYSGELTDIGVT
IDEIANMIWINAMKPVYESCMEKSIQH
>Locus_5652_Transcript_6/9_Confidence_0.556_Length_1850|.|sp|Q9VC57|
ATLAS_DROME Atlastin OS=Drosophila melanogaster GN=atl PE=1 SV=1|
e=0|from=3|to=1523|frame=-1|Fragment_1
GHPVQIVRTLSDHTFELDEAALSEILNRPELKDRAAVVVSVAGAFRKGKSFLLDFFLRYM
NSKYVNRESSGDWLGAEDAPLSGFSWRGGSERDTTGILMWSEPFLHESANGQKLAVLLLD
TQGTFDSESTVKDCATVFALSTMLSSVQIYNLTQNIQEDDLQHLHLFTEYGRLALADSGQ
KPFQRLQFLVRDWSFPYEEQYGAVGGQNLLQRRMKISERQHPELQSLRKHIASCFTEVAC
FLMPHPGLTVATNPNFDGRLTDITPEFKDSLKVFIPMLLAPENLIAKRINGVPVKCRDLV
QYFKAYMSIYKGNELPEPKSMLVATAEANNLSAVAEAKDMYTQLMEDICGGSKPYLSTAH
LESEHLRIRDKAVGTFIQKRKMGGSAFSENYRIKLEADLDEAFEQFKGHNDSKNIFKAAR
TPAVFFALLILMYVLSGVFGLVGLYAISNICNFAMGVALLTLVLWAYIRYSGELTDIGVT
IDEIANMIWINAMKPVYESCMEKSIQH
>Locus_2946_Transcript_5/6_Confidence_0.308_Length_890|UNIGENE
REPRESENTATIVE TRANSCRIPT|sp|Q9VDD7|S35B1_DROME Solute carrier
family 35 member B1 homolog OS=Drosophila melanogaster GN=CG5802
PE=1 SV=1|e=7.2237E-72|from=1|to=846|frame=1|Fragment_1
ETFKFALTLVFIQCVVNYIFAKFILFVTPRDEDRTPDSLYAGASLTYLGAMVTSTMALQW

VSYPTQVIGKAAKPIPVMLLGVLIGR
>Locus_2946_Transcript_5/6_Confidence_0.308_Length_890|UNIGENE REPRESENTATIVE TRANSCRIPT|sp|Q9VDD7|S35B1_DROME Solute carrier family 35 member B1 homolog OS=Drosophila melanogaster GN=CG5802 PE=1 SV=1|e=7.2237E-72|from=1|to=846|frame=1|Fragment_2
CVVLIVAGIALFMYKDNESSNSSSTNKFGIGEILLLFSLLMDGILGALQDLIRDYKPPNV
LKTSTEEHTPPSGLNMMLAINGWSANYLALILLFISEGKDFIRFSVCYPYILQQLAILAV
TGAVGQLFIYIIISKFGPLSCSIVTTTRKFFTVLFSVIFFANPINTRQWVGAAVVFTGLF
LDIIYGK
>Locus_2946_Transcript_3/6_Confidence_0.385_Length_838|.|sp|Q9VDD7|S35B1_DROME Solute carrier family 35 member B1 homolog OS=Drosophila melanogaster GN=CG5802 PE=1 SV=1|e=1.31772E-64|from=258|to=827|frame=3|Fragment_1
ENSKFLFYAAGIFVCYFYFGILQEKITRGKYGDEVNEKGEKGEKFKCALALVFVQCVVNY
VFAKGILYFKPQDEDRTPKVYYASSALTYLLAMVSSNMALQWVPYPTQVVGKAAKPIPVM
LLGVLIGHKSYPMFRYFCVLLIVIGVVLFMYKDNKSSVNVDSSFGIGELLLVMSLSMDGL
TGAIQDRMRA
>Locus_2946_Transcript_1/6_Confidence_0.308_Length_838|.|sp|Q9VDD7|S35B1_DROME Solute carrier family 35 member B1 homolog OS=Drosophila melanogaster GN=CG5802 PE=1 SV=1|e=1.843E-58|from=258|to=827|frame=3|Fragment_1
ENSKFLFYAAGIFVCYFYFGILQEKITRGKYGDEVNEKGEKGEKFKCALALVFVQCVVNY
VFAKGILYFKPQDEDRTPKVYYASSALTYLLAMVSSNMALQWVPYPTQVVGKAAKPIPVM
LLGVLIGHKSYPMFRY
>Locus_2946_Transcript_1/6_Confidence_0.308_Length_838|.|sp|Q9VDD7|S35B1_DROME Solute carrier family 35 member B1 homolog OS=Drosophila melanogaster GN=CG5802 PE=1 SV=1|e=1.843E-58|from=258|to=827|frame=3|Fragment_2
FMYKDNKSSVNVDSSFGIGELLLVMSLSMDGLTGAIQDRMRA
>Locus_14123_Transcript_1/1_Confidence_1.000_Length_663|.|sp|Q9VDD7|S35B1_DROME Solute carrier family 35 member B1 homolog OS=Drosophila melanogaster GN=CG5802 PE=1 SV=1|e=6.08631E-45|from=16|to=483|frame=1|Fragment_1
IGELLLVMSLSMDGLTGAIQDRMRAHKPSTSTAQSHKPPSAEKMMVATNGWSATYLSVAL
LLSGEGREFLSFAVRYPYIGTQLAALAITGALGQLFIFMMVSKFGPLACSVVTTTRKFFT
VLFSVIFLANPLIARQWFGAVLVFTGLFLDMIYGKK
>Locus_7682_Transcript_1/1_Confidence_1.000_Length_429|.|sp|Q9VDD7|S35B1_DROME Solute carrier family 35 member B1 homolog OS=Drosophila melanogaster GN=CG5802 PE=1 SV=1|e=3.40001E-36|from=4|to=414|frame=-1|Fragment_1
IGELLLVMSLSMDGLTGAIQDRMRAHKPSSSTAQFHKPPSAEKMMVATNGWSATYISVAL
LLSGEGRDFLSFAVRYPYIGTQLAALAVTGALGQLFIFMMVSKFGPLACSVVTTTRKFFT
VLFSVIFLANPLIARQW
>Locus_2768_Transcript_4/7_Confidence_0.320_Length_1304|UNIGENE REPRESENTATIVE TRANSCRIPT|sp|Q9VE50|GOSR1_DROME Golgi SNAP receptor complex member 1 OS=Drosophila melanogaster GN=Gos28 PE=2 SV=1|e=9.24175E-11|from=2|to=178|frame=2|Fragment_1
LRKQARHLENHVDLKLVAFSKMGISSPGCLSS
>Locus_2768_Transcript_4/7_Confidence_0.320_Length_1304|UNIGENE REPRESENTATIVE TRANSCRIPT|sp|Q9VE50|GOSR1_DROME Golgi SNAP receptor complex member 1 OS=Drosophila melanogaster GN=Gos28 PE=2 SV=1|e=9.24175E-11|from=2|to=178|frame=2|Fragment_2
GGSDKSPLLVEHICDNMAMEIDGMLDK
>Locus_1401_Transcript_1/1_Confidence_1.000_Length_879|.|sp|Q9VE50|

GOSR1_DROME Golgi SNAP receptor complex member 1 OS=Drosophila
melanogaster GN=Gos28 PE=2 SV=1|e=1.86874E-56|from=336|to=878|
frame=-2|Fragment_1
GEHICDNMAMEIDGMLEKLSTLNEKMSELPASGAAMLHTIQRHREILQGYKQEFRKIQSN
HNS
>Locus_1401_Transcript_1/1_Confidence_1.000_Length_879|.|sp|Q9VE50|
GOSR1_DROME Golgi SNAP receptor complex member 1 OS=Drosophila
melanogaster GN=Gos28 PE=2 SV=1|e=1.86874E-56|from=336|to=878|
frame=-2|Fragment_2
RDNDSSMDKMNSVNAGLNRRDLFLKESSHINSSHNLVNDQISIAVETRDNLLSQRQTFKR
MKTRFNDINNKFPVINNLMQRIRIRKRSDSLILGFVIAVCTILLFLYAFH
>Locus_19876_Transcript_1/1_Confidence_1.000_Length_871|UNIGENE
REPRESENTATIVE TRANSCRIPT|sp|Q9VEG9|NRM_DROME Nurim homolog
OS=Drosophila melanogaster GN=nrm PE=1 SV=1|e=3.99726E-43|from=13|
to=723|frame=1|Fragment_1
AALLSFLCSFYSAVCLLLFL
>Locus_19876_Transcript_1/1_Confidence_1.000_Length_871|UNIGENE
REPRESENTATIVE TRANSCRIPT|sp|Q9VEG9|NRM_DROME Nurim homolog
OS=Drosophila melanogaster GN=nrm PE=1 SV=1|e=3.99726E-43|from=13|
to=723|frame=1|Fragment_2
TAHLEH
>Locus_19876_Transcript_1/1_Confidence_1.000_Length_871|UNIGENE
REPRESENTATIVE TRANSCRIPT|sp|Q9VEG9|NRM_DROME Nurim homolog
OS=Drosophila melanogaster GN=nrm PE=1 SV=1|e=3.99726E-43|from=13|
to=723|frame=1|Fragment_3
DLLEAVYS
>Locus_19876_Transcript_1/1_Confidence_1.000_Length_871|UNIGENE
REPRESENTATIVE TRANSCRIPT|sp|Q9VEG9|NRM_DROME Nurim homolog
OS=Drosophila melanogaster GN=nrm PE=1 SV=1|e=3.99726E-43|from=13|
to=723|frame=1|Fragment_4
LIKDYALLALFIIQHSCMASSTYKKFIERINLGVIERSIYNFATSLCILLLINQWKYVNT
YKLWDLPTESYPIINSGIFIFHLISWSVIYGGSLLMDTPEIFGIKQVIYDVKGLSCPIAY
KSRELRRLYQHMRHPSFIALFVIFWCTPVMSLDRLLLASMWSLYMLLGWRTDSFDVDYQK
DQLDRKRHEL
>Locus_2718_Transcript_3/6_Confidence_0.667_Length_3396|UNIGENE
REPRESENTATIVE TRANSCRIPT|sp|Q9VEI3|S35B2_DROME Adenosine 3'-phospho
5'-phosphosulfate transporter 1 OS=Drosophila melanogaster GN=sll
PE=1 SV=1|e=1.6013E-138|from=666|to=2009|frame=-2|Fragment_1
IPEIIICLLIAGCVGTVFLFSQIIHNAVG
>Locus_2718_Transcript_3/6_Confidence_0.667_Length_3396|UNIGENE
REPRESENTATIVE TRANSCRIPT|sp|Q9VEI3|S35B2_DROME Adenosine 3'-phospho
5'-phosphosulfate transporter 1 OS=Drosophila melanogaster GN=sll
PE=1 SV=1|e=1.6013E-138|from=666|to=2009|frame=-2|Fragment_2
SSEISLSQIINNQPKEYSWLLRLGLNCIGYVSIFGPLYIINKYVHKTNYLDRAGSGYVSC
LTRMCLSEQEKLG
>Locus_2718_Transcript_3/6_Confidence_0.667_Length_3396|UNIGENE
REPRESENTATIVE TRANSCRIPT|sp|Q9VEI3|S35B2_DROME Adenosine 3'-phospho
5'-phosphosulfate transporter 1 OS=Drosophila melanogaster GN=sll
PE=1 SV=1|e=1.6013E-138|from=666|to=2009|frame=-2|Fragment_3
DTQSQTLSK
>Locus_2718_Transcript_3/6_Confidence_0.667_Length_3396|UNIGENE
REPRESENTATIVE TRANSCRIPT|sp|Q9VEI3|S35B2_DROME Adenosine 3'-phospho
5'-phosphosulfate transporter 1 OS=Drosophila melanogaster GN=sll
PE=1 SV=1|e=1.6013E-138|from=666|to=2009|frame=-2|Fragment_4

ADLNTKKSFLEECSLLTVCLTGLMGSYLTWGLYQEEIMTHEYINSEGKKTTFNDSQFLVF
INRSLSFILALIYISIRKQP
>Locus_2718_Transcript_3/6_Confidence_0.667_Length_3396|UNIGENE
REPRESENTATIVE TRANSCRIPT|sp|Q9VEI3|S35B2_DROME Adenosine 3'-phospho
5'-phosphosulfate transporter 1 OS=Drosophila melanogaster GN=sll
PE=1 SV=1|e=1.6013E-138|from=666|to=2009|frame=-2|Fragment_5
RHRAPLYKYSFCSFSNIMSAWSQYEALKYVSFPTQVLAKSCKILPVMAMGKIISRVKYEN
YEYITAALICIGMVFFLFGSTSDHKGNSATTMSGVILLSLYMLFDSFTSNWQGALFKKYS
MSSIQMMCGVNLFSALFTLISLLQQGGFMESIMFMTQHQKFIGDCILLSISSASGQLFVY
FTIATFGPVVFTIIMTLRQAIAILLSCIVYGHSITALGIFGVLLVFCAIFLRVYCNQRLK
SINQKRLNLENKPKVVI
>Locus_2718_Transcript_4/6_Confidence_0.667_Length_3396|.|sp|Q9VEI3|
S35B2_DROME Adenosine 3'-phospho 5'-phosphosulfate transporter 1
OS=Drosophila melanogaster GN=sll PE=1 SV=1|e=1.6013E-138|from=666|
to=2009|frame=-2|Fragment_1
IPEIIICLLIAGCVGTVFLFSQIIHNAVG
>Locus_2718_Transcript_4/6_Confidence_0.667_Length_3396|.|sp|Q9VEI3|
S35B2_DROME Adenosine 3'-phospho 5'-phosphosulfate transporter 1
OS=Drosophila melanogaster GN=sll PE=1 SV=1|e=1.6013E-138|from=666|
to=2009|frame=-2|Fragment_2
SSEISLSQIINNQPKEYSWLLRLGLNCIGYVSIFGPLYIINKYVHKTNYLDRAGSGYVSC
LTRMCLSEQEKLG
>Locus_2718_Transcript_4/6_Confidence_0.667_Length_3396|.|sp|Q9VEI3|
S35B2_DROME Adenosine 3'-phospho 5'-phosphosulfate transporter 1
OS=Drosophila melanogaster GN=sll PE=1 SV=1|e=1.6013E-138|from=666|
to=2009|frame=-2|Fragment_3
DTQSQTLSK
>Locus_2718_Transcript_4/6_Confidence_0.667_Length_3396|.|sp|Q9VEI3|
S35B2_DROME Adenosine 3'-phospho 5'-phosphosulfate transporter 1
OS=Drosophila melanogaster GN=sll PE=1 SV=1|e=1.6013E-138|from=666|
to=2009|frame=-2|Fragment_4
ADLNTKKSFLEECSLLTVCLTGLMGSYLTWGLYQEEIMTHEYINSEGKKTTFNDSQFLVF
INRSLSFILALIYISIRKQP
>Locus_2718_Transcript_4/6_Confidence_0.667_Length_3396|.|sp|Q9VEI3|
S35B2_DROME Adenosine 3'-phospho 5'-phosphosulfate transporter 1
OS=Drosophila melanogaster GN=sll PE=1 SV=1|e=1.6013E-138|from=666|
to=2009|frame=-2|Fragment_5
RHRAPLYKYSFCSFSNIMSAWSQYEALKYVSFPTQVLAKSCKILPVMAMGKIISRVKYEN
YEYITAALICIGMVFFLFGSTSDHKGNSATTMSGVILLSLYMLFDSFTSNWQGALFKKYS
MSSIQMMCGVNLFSALFTLISLLQQGGFMESIMFMTQHQKFIGDCILLSISSASGQLFVY
FTIATFGPVVFTIIMTLRQAIAILLSCIVYGHSITALGIFGVLLVFCAIFLRVYCNQRLK
SINQKRLNLENKPKVVI
>Locus_2718_Transcript_6/6_Confidence_0.667_Length_3396|.|sp|Q9VEI3|
S35B2_DROME Adenosine 3'-phospho 5'-phosphosulfate transporter 1
OS=Drosophila melanogaster GN=sll PE=1 SV=1|e=1.6013E-138|from=666|
to=2009|frame=-2|Fragment_1
IPEIIICLLIAGCVGTVFLFSQIIHNAVG
>Locus_2718_Transcript_6/6_Confidence_0.667_Length_3396|.|sp|Q9VEI3|
S35B2_DROME Adenosine 3'-phospho 5'-phosphosulfate transporter 1
OS=Drosophila melanogaster GN=sll PE=1 SV=1|e=1.6013E-138|from=666|
to=2009|frame=-2|Fragment_2
SSEISLSQIINNQPKEYSWLLRLGLNCIGYVSIFGPLYIINKYVHKTNYLDRAGSGYVSC
LTRMCLSEQEKLG
>Locus_2718_Transcript_6/6_Confidence_0.667_Length_3396|.|sp|Q9VEI3|

S35B2_DROME Adenosine 3'-phospho 5'-phosphosulfate transporter 1 OS=Drosophila melanogaster GN=sll PE=1 SV=1|e=1.6013E-138|from=666|to=2009|frame=-2|Fragment_3
DTQSQTLSK
>Locus_2718_Transcript_6/6_Confidence_0.667_Length_3396|.|sp|Q9VEI3|S35B2_DROME Adenosine 3'-phospho 5'-phosphosulfate transporter 1 OS=Drosophila melanogaster GN=sll PE=1 SV=1|e=1.6013E-138|from=666|to=2009|frame=-2|Fragment_4
ADLNTKKSFLEECSLLTVCLTGLMGSYLTWGLYQEEIMTHEYINSEGKKTTFNDSQFLVF
INRSLSFILALIYISIRKQP
>Locus_2718_Transcript_6/6_Confidence_0.667_Length_3396|.|sp|Q9VEI3|S35B2_DROME Adenosine 3'-phospho 5'-phosphosulfate transporter 1 OS=Drosophila melanogaster GN=sll PE=1 SV=1|e=1.6013E-138|from=666|to=2009|frame=-2|Fragment_5
RHRAPLYKYSFCSFSNIMSAWSQYEALKYVSFPTQVLAKSCKILPVMAMGKIISRVKYEN
YEYITAALICIGMVFFLFGSTSDHKGNSATTMSGVILLSLYMLFDSFTSNWQGALFKKYS
MSSIQMMCGVNLFSALFTLISLLQQGGFMESIMFMTQHQKFIGDCILLSISSASGQLFVY
FTIATFGPVVFTIIMTLRQAIAILLSCIVYGHSITALGIFGVLLVFCAIFLRVYCNQRLK
SINQKRLNLENKPKVVI
>Locus_2718_Transcript_2/6_Confidence_0.667_Length_3288|.|sp|Q9VEI3|S35B2_DROME Adenosine 3'-phospho 5'-phosphosulfate transporter 1 OS=Drosophila melanogaster GN=sll PE=1 SV=1|e=1.5442E-138|from=558|to=1901|frame=-2|Fragment_1
IPEIIICLLIAGCVGTVFLFSQIIHNAVG
>Locus_2718_Transcript_2/6_Confidence_0.667_Length_3288|.|sp|Q9VEI3|S35B2_DROME Adenosine 3'-phospho 5'-phosphosulfate transporter 1 OS=Drosophila melanogaster GN=sll PE=1 SV=1|e=1.5442E-138|from=558|to=1901|frame=-2|Fragment_2
SSEISLSQIINNQPKEYSWLLRLGLNCIGYVSIFGPLYIINKYVHKTNYLDRAGSGYVSC
LTRMCLSEQEKLG
>Locus_2718_Transcript_2/6_Confidence_0.667_Length_3288|.|sp|Q9VEI3|S35B2_DROME Adenosine 3'-phospho 5'-phosphosulfate transporter 1 OS=Drosophila melanogaster GN=sll PE=1 SV=1|e=1.5442E-138|from=558|to=1901|frame=-2|Fragment_3
DTQSQTLSK
>Locus_2718_Transcript_2/6_Confidence_0.667_Length_3288|.|sp|Q9VEI3|S35B2_DROME Adenosine 3'-phospho 5'-phosphosulfate transporter 1 OS=Drosophila melanogaster GN=sll PE=1 SV=1|e=1.5442E-138|from=558|to=1901|frame=-2|Fragment_4
ADLNTKKSFLEECSLLTVCLTGLMGSYLTWGLYQEEIMTHEYINSEGKKTTFNDSQFLVF
INRSLSFILALIYISIRKQP
>Locus_2718_Transcript_2/6_Confidence_0.667_Length_3288|.|sp|Q9VEI3|S35B2_DROME Adenosine 3'-phospho 5'-phosphosulfate transporter 1 OS=Drosophila melanogaster GN=sll PE=1 SV=1|e=1.5442E-138|from=558|to=1901|frame=-2|Fragment_5
RHRAPLYKYSFCSFSNIMSAWSQYEALKYVSFPTQVLAKSCKILPVMAMGKIISRVKYEN
YEYITAALICIGMVFFLFGSTSDHKGNSATTMSGVILLSLYMLFDSFTSNWQGALFKKYS
MSSIQMMCGVNLFSALFTLISLLQQGGFMESIMFMTQHQKFIGDCILLSISSASGQLFVY
FTIATFGPVVFTIIMTLRQAIAILLSCIVYGHSITALGIFGVLLVFCAIFLRVYCNQRLK
SINQKRLNLENKPKVVI
>Locus_2718_Transcript_1/6_Confidence_0.667_Length_3253|.|sp|Q9VEI3|S35B2_DROME Adenosine 3'-phospho 5'-phosphosulfate transporter 1 OS=Drosophila melanogaster GN=sll PE=1 SV=1|e=1.5251E-138|from=666|to=2009|frame=-3|Fragment_1

IPEIIICLLIAGCVGTVFLFSQIIHNAVG
>Locus_2718_Transcript_1/6_Confidence_0.667_Length_3253|.|sp|Q9VEI3|S35B2_DROME Adenosine 3'-phospho 5'-phosphosulfate transporter 1 OS=Drosophila melanogaster GN=sll PE=1 SV=1|e=1.5251E-138|from=666|to=2009|frame=-3|Fragment_2
SSEISLSQIINNQPKEYSWLLRLGLNCIGYVSIFGPLYIINKYVHKTNYLDRAGSGYVSC
LTRMCLSEQEKLG
>Locus_2718_Transcript_1/6_Confidence_0.667_Length_3253|.|sp|Q9VEI3|S35B2_DROME Adenosine 3'-phospho 5'-phosphosulfate transporter 1 OS=Drosophila melanogaster GN=sll PE=1 SV=1|e=1.5251E-138|from=666|to=2009|frame=-3|Fragment_3
DTQSQTLSK
>Locus_2718_Transcript_1/6_Confidence_0.667_Length_3253|.|sp|Q9VEI3|S35B2_DROME Adenosine 3'-phospho 5'-phosphosulfate transporter 1 OS=Drosophila melanogaster GN=sll PE=1 SV=1|e=1.5251E-138|from=666|to=2009|frame=-3|Fragment_4
ADLNTKKSFLEECSLLTVCLTGLMGSYLTWGLYQEEIMTHEYINSEGKKTTFNDSQFLVF
INRSLSFILALIYISIRKQP
>Locus_2718_Transcript_1/6_Confidence_0.667_Length_3253|.|sp|Q9VEI3|S35B2_DROME Adenosine 3'-phospho 5'-phosphosulfate transporter 1 OS=Drosophila melanogaster GN=sll PE=1 SV=1|e=1.5251E-138|from=666|to=2009|frame=-3|Fragment_5
RHRAPLYKYSFCSFSNIMSAWSQYEALKYVSFPTQVLAKSCKILPVMAMGKIISRVKYEN
YEYITAALICIGMVFFLFGSTSDHKGNSATTMSGVILLSLYMLFDSFTSNWQGALFKKYS
MSSIQMMCGVNLFSALFTLISLLQQGGFMESIMFMTQHQKFIGDCILLSISSASGQLFVY
FTIATFGPVVFTIIMTLRQAIAILLSCIVYGHSITALGIFGVLLVFCAIFLRVYCNQRLK
SINQKRLNLENKPKVVI
>Locus_14167_Transcript_1/3_Confidence_0.600_Length_1039|.|sp|Q9VEI3|S35B2_DROME Adenosine 3'-phospho 5'-phosphosulfate transporter 1 OS=Drosophila melanogaster GN=sll PE=1 SV=1|e=6.1936E-105|from=146|to=1039|frame=-1|Fragment_1
LTWGLYQEEIMTHEYINNKGNKTTFNDSQFLVFINRSLSFILALIYITITKQP
>Locus_14167_Transcript_1/3_Confidence_0.600_Length_1039|.|sp|Q9VEI3|S35B2_DROME Adenosine 3'-phospho 5'-phosphosulfate transporter 1 OS=Drosophila melanogaster GN=sll PE=1 SV=1|e=6.1936E-105|from=146|to=1039|frame=-1|Fragment_2
RHRAPLYKYSFCSFSNIMSAWSQYEALKYVSFPTQVLAKSCKILPVMAMGKIISRNKYEN
YEYITAALICIGMVLFLFGSTSDHTDTPATTMSGVIILSLYMLFDSFTSNWQGALFKRYS
MTSIQMMCGVN
>Locus_14167_Transcript_1/3_Confidence_0.600_Length_1039|.|sp|Q9VEI3|S35B2_DROME Adenosine 3'-phospho 5'-phosphosulfate transporter 1 OS=Drosophila melanogaster GN=sll PE=1 SV=1|e=6.1936E-105|from=146|to=1039|frame=-1|Fragment_3
QGGFMESMIFMTQHQKFIGDCILLSISSASGQLFVYFTIATFGPVVFTIIMTMRQAVAII
LSCIVYGHNITAIGIFGVLLVFSAILLRVYCNQRLKSINQK
>Locus_19552_Transcript_1/1_Confidence_1.000_Length_474|UNIGENE REPRESENTATIVE TRANSCRIPT|sp|Q9VGG8|MTH5_DROME Probable G-protein coupled receptor Mth-like 5 OS=Drosophila melanogaster GN=mthl5 PE=2 SV=2|e=1.29188E-56|from=3|to=473|frame=-2|Fragment_1
INPALHILCIAILLTVAVIYFVLPTLRDLVGNIITTMSMCLIITQAADLVRIYTEFSSHV
SFMVADSVLYVSLQATFFWLSGLGYYIWSTFRSRNVFLRVTDGRKYCWYSGYVWSCTTIM
AALALFAHYTLDNVKTNNAVKDTNQPTVGWLGIAVFF
>Locus_20137_Transcript_1/1_Confidence_1.000_Length_425|.|sp|Q9VGG8|MTH5_DROME Probable G-protein coupled receptor Mth-like 5

OS=Drosophila melanogaster GN=mthl5 PE=2 SV=2|e=5.5555E-20|from=1|
to=216|frame=1|Fragment_1
WMSMLLSWLNFPLLFYTHVIINALHAPAILYVCVLSQKHVTFLLRKSCCYGQEAVPQNEW
GDEMTHMNGFDY
>Locus_10361_Transcript_1/1_Confidence_1.000_Length_410|.|sp|Q9VGG8|
MTH5_DROME Probable G-protein coupled receptor Mth-like 5
OS=Drosophila melanogaster GN=mthl5 PE=2 SV=2|e=4.2481E-27|from=4|
to=378|frame=1|Fragment_1
NYVRVNKCCEINELKVEDRCTHVNETSAELWQPIFTGLNGEQKVQVKFKFVTGIPQCGST
EQRHIYHYAASSDKLVLLSNGNLRH
>Locus_10361_Transcript_1/1_Confidence_1.000_Length_410|.|sp|Q9VGG8|
MTH5_DROME Probable G-protein coupled receptor Mth-like 5
OS=Drosophila melanogaster GN=mthl5 PE=2 SV=2|e=4.2481E-27|from=4|
to=378|frame=1|Fragment_2
GSSEPTYYDYIPGLYCMDKIISN
>Locus_10556_Transcript_2/6_Confidence_0.133_Length_494|UNIGENE
REPRESENTATIVE TRANSCRIPT|sp|Q9VGH7|CLCN2_DROME Chloride channel
protein 2 OS=Drosophila melanogaster GN=ClC-a PE=2 SV=3|
e=1.43809E-32|from=29|to=382|frame=2|Fragment_1
HVDPAPFQLVERTSLLKVHSLFSMVGVNHAYVTTIGKLIGVVALKELRKAIEDANSGNLP
SSQAPSVPAKSTDCEALKSADTPLLVNSHSQDHHNQNTVTSMDSALSSCENCSDIEMD
>Locus_19114_Transcript_1/1_Confidence_1.000_Length_1347|UNIGENE
REPRESENTATIVE TRANSCRIPT|sp|Q9VGW1|CAD86_DROME Cadherin-86C
OS=Drosophila melanogaster GN=Cad86C PE=2 SV=3|e=6.0527E-122|from=2|
to=1201|frame=2|Fragment_1
YVIRLDENSPQGTTLFF
>Locus_19114_Transcript_1/1_Confidence_1.000_Length_1347|UNIGENE
REPRESENTATIVE TRANSCRIPT|sp|Q9VGW1|CAD86_DROME Cadherin-86C
OS=Drosophila melanogaster GN=Cad86C PE=2 SV=3|e=6.0527E-122|from=2|
to=1201|frame=2|Fragment_2
KGVFSLQLIGNNGTFEISPSVGDRDVTFLIRVRDPAMLDYEKMPVLSFQIVAQELGPTTN
LSAIANVTIFLNDVNDNPPVFLQPEYRADLAENATAGTRVIQVQAKDEDTGPGGRLRYTR
ILGYRNTSLKLDSETGWITVATNNHGFDSEEMPEYSFLVEARDADGEGNAATTSLVLSIT
DVNDNAPHFERDVYEFMLSSGLNNFTVPAFLHATDADATSPNNQVRYEIVHGNYDEKFRL
DEITGELTVN
>Locus_19114_Transcript_1/1_Confidence_1.000_Length_1347|UNIGENE
REPRESENTATIVE TRANSCRIPT|sp|Q9VGW1|CAD86_DROME Cadherin-86C
OS=Drosophila melanogaster GN=Cad86C PE=2 SV=3|e=6.0527E-122|from=2|
to=1201|frame=2|Fragment_3
NIKTSRDTSNKQGNGLKPE
>Locus_19114_Transcript_1/1_Confidence_1.000_Length_1347|UNIGENE
REPRESENTATIVE TRANSCRIPT|sp|Q9VGW1|CAD86_DROME Cadherin-86C
OS=Drosophila melanogaster GN=Cad86C PE=2 SV=3|e=6.0527E-122|from=2|
to=1201|frame=2|Fragment_4
VYAMTVRAYDLGVPHMAATTSVRVYPPESRARSLMFVVPGSKPDLKATADTIRALTGAAV
TIEDVRPHDTD
>Locus_19114_Transcript_1/1_Confidence_1.000_Length_1347|UNIGENE
REPRESENTATIVE TRANSCRIPT|sp|Q9VGW1|CAD86_DROME Cadherin-86C
OS=Drosophila melanogaster GN=Cad86C PE=2 SV=3|e=6.0527E-122|from=2|
to=1201|frame=2|Fragment_5
PDKSIVLASLHYTSNSVVDVDQIQRRLS
>Locus_30916_Transcript_1/1_Confidence_1.000_Length_419|.|sp|Q9VGW1|
CAD86_DROME Cadherin-86C OS=Drosophila melanogaster GN=Cad86C PE=2
SV=3|e=6.79928E-55|from=3|to=419|frame=-1|Fragment_1

IMVPEDAKRGTELEFIIARKNPLSQKPVYLELWGSPLFAIRQRLTTPDTTEGVIFLLGPL
DFETQSMHHLTILANDAFPNPRYDSRNIFELRVAVAVQDVQDTPPVFSSAPPVTSLAPTL
LPGDVVARVKAEDGDRGKP
>Locus_30094_Transcript_1/1_Confidence_1.000_Length_261|.|sp|Q9VGW1|
CAD86_DROME Cadherin-86C OS=Drosophila melanogaster GN=Cad86C PE=2
SV=3|e=6.03862E-25|from=2|to=259|frame=2|Fragment_1
DYPLHFELLEGASSSAFSVDSLPCTRYNSVCQANVVLRRRLERGRYYDFRVRVADTAGDS
TTLDCAVSATNGTTPIQDTFPKAPGL
>Locus_12723_Transcript_1/1_Confidence_1.000_Length_1179|UNIGENE
REPRESENTATIVE TRANSCRIPT|sp|Q9VHN5|UNC50_DROME Protein unc-50
homolog OS=Drosophila melanogaster GN=CG9773 PE=1 SV=1|
e=2.22109E-93|from=203|to=1009|frame=2|Fragment_1
LLKFKIMKQPRSPSASLFS
>Locus_12723_Transcript_1/1_Confidence_1.000_Length_1179|UNIGENE
REPRESENTATIVE TRANSCRIPT|sp|Q9VHN5|UNC50_DROME Protein unc-50
homolog OS=Drosophila melanogaster GN=CG9773 PE=1 SV=1|
e=2.22109E-93|from=203|to=1009|frame=2|Fragment_2
YSRSTSPLPAPINHRHDCMSAFSKIHRYVRRLFKFQQMDFEFAVWQMVYLFIAPQKVYRN
FNYRKQTKSQFARDDPAFLVLFTGMLCVTSLGFGWVFNLQFSQTLYFLLYVIFVDCIFMG
VLVATMFWFVTNRYLRINP
>Locus_12723_Transcript_1/1_Confidence_1.000_Length_1179|UNIGENE
REPRESENTATIVE TRANSCRIPT|sp|Q9VHN5|UNC50_DROME Protein unc-50
homolog OS=Drosophila melanogaster GN=CG9773 PE=1 SV=1|
e=2.22109E-93|from=203|to=1009|frame=2|Fragment_3
NADVEWGYAFDVHLNAFVPPLIILHFLQLFFYYGVISHDLFISRLIGNSFWMMAVSYYIY
ITFLGYSCIPE
>Locus_12723_Transcript_1/1_Confidence_1.000_Length_1179|UNIGENE
REPRESENTATIVE TRANSCRIPT|sp|Q9VHN5|UNC50_DROME Protein unc-50
homolog OS=Drosophila melanogaster GN=CG9773 PE=1 SV=1|
e=2.22109E-93|from=203|to=1009|frame=2|Fragment_4
FVVYLISLAVGWNVSETLINFYLGRV
>Locus_21424_Transcript_1/1_Confidence_1.000_Length_743|UNIGENE
REPRESENTATIVE TRANSCRIPT|sp|Q9VL32|SUR_DROME ATP-binding cassette
sub-family C member Sur OS=Drosophila melanogaster GN=Sur PE=1 SV=4|
e=9.74999E-13|from=12|to=680|frame=-1|Fragment_1
SEKPNGHEDIRSKRHTI
>Locus_21424_Transcript_1/1_Confidence_1.000_Length_743|UNIGENE
REPRESENTATIVE TRANSCRIPT|sp|Q9VL32|SUR_DROME ATP-binding cassette
sub-family C member Sur OS=Drosophila melanogaster GN=Sur PE=1 SV=4|
e=9.74999E-13|from=12|to=680|frame=-1|Fragment_2
DSPHASNILRQILSKTQNVNKTLSDNKSGEQRNIFRRLA
>Locus_21424_Transcript_1/1_Confidence_1.000_Length_743|UNIGENE
REPRESENTATIVE TRANSCRIPT|sp|Q9VL32|SUR_DROME ATP-binding cassette
sub-family C member Sur OS=Drosophila melanogaster GN=Sur PE=1 SV=4|
e=9.74999E-13|from=12|to=680|frame=-1|Fragment_3
DRLSFRRSNRSKEINRHKIISRLMSDASDITVDLNDLASCETHQISSLSEYNYNQRTAEN
QERIYGTIPLNIYLYYIKCCGIKICTLYIFLAFLWQGLRLFADMSLGNWSR
>Locus_21424_Transcript_1/1_Confidence_1.000_Length_743|UNIGENE
REPRESENTATIVE TRANSCRIPT|sp|Q9VL32|SUR_DROME ATP-binding cassette
sub-family C member Sur OS=Drosophila melanogaster GN=Sur PE=1 SV=4|
e=9.74999E-13|from=12|to=680|frame=-1|Fragment_4
ENMESYEGF
>Locus_21424_Transcript_1/1_Confidence_1.000_Length_743|UNIGENE
REPRESENTATIVE TRANSCRIPT|sp|Q9VL32|SUR_DROME ATP-binding cassette

sub-family C member Sur OS=Drosophila melanogaster GN=Sur PE=1 SV=4|
e=9.74999E-13|from=12|to=680|frame=-1|Fragment_5
DQSHEKAFSLLSTYCYQSAICALVSLLCAFSGQWAGLVARSNIHEYL
>Locus_26063_Transcript_1/1_Confidence_1.000_Length_355|.|sp|Q9VL32|
SUR_DROME ATP-binding cassette sub-family C member Sur OS=Drosophila
melanogaster GN=Sur PE=1 SV=4|e=6.32552E-22|from=2|to=355|frame=2|
Fragment_1
LHVAYLEIPKGKLTLIVGNTGSGKTSFMSAILSEMFLIQGSMKWASDTRIAYCAQKPFLM
NASIQENITMFGQHLCRDKNCKRYKKVLKLCALDKELTEIQQSNLQTIGENGNTLSGG
>Locus_15616_Transcript_3/3_Confidence_0.667_Length_785|UNIGENE
REPRESENTATIVE TRANSCRIPT|sp|Q9VM08|GR28B_DROME Putative gustatory
receptor 28b OS=Drosophila melanogaster GN=Gr28b PE=1 SV=2|
e=1.0971E-17|from=27|to=755|frame=3|Fragment_1
IFTTQIIVRLYG
>Locus_15616_Transcript_3/3_Confidence_0.667_Length_785|UNIGENE
REPRESENTATIVE TRANSCRIPT|sp|Q9VM08|GR28B_DROME Putative gustatory
receptor 28b OS=Drosophila melanogaster GN=Gr28b PE=1 SV=2|
e=1.0971E-17|from=27|to=755|frame=3|Fragment_2
IYPRASLWFSFVFPNIVSTMIILQFSFYVYILTLRMGLIKTLINNLEIISTSAWLDGDEK
THNMKQKKY
>Locus_15616_Transcript_3/3_Confidence_0.667_Length_785|UNIGENE
REPRESENTATIVE TRANSCRIPT|sp|Q9VM08|GR28B_DROME Putative gustatory
receptor 28b OS=Drosophila melanogaster GN=Gr28b PE=1 SV=2|
e=1.0971E-17|from=27|to=755|frame=3|Fragment_3
ATIIDVIGKLHEKLCEIVDHINAYFNNQMLVLCLDAFVMIVFFAYYSLISIMDYEADLAK
Y
>Locus_15616_Transcript_3/3_Confidence_0.667_Length_785|UNIGENE
REPRESENTATIVE TRANSCRIPT|sp|Q9VM08|GR28B_DROME Putative gustatory
receptor 28b OS=Drosophila melanogaster GN=Gr28b PE=1 SV=2|
e=1.0971E-17|from=27|to=755|frame=3|Fragment_4
IGVMAFFLCPFIANICQMIYLVVFCAQFYNEITKCYKKLLVLINESEKRCTKLRLNQLSI
QIIIRSPKFSAGGVFILKPTLLRSMVASSSIYLIILFQFSN
>Locus_15616_Transcript_2/3_Confidence_0.667_Length_785|.|sp|Q9VM08|
GR28B_DROME Putative gustatory receptor 28b OS=Drosophila
melanogaster GN=Gr28b PE=1 SV=2|e=3.19208E-17|from=27|to=755|
frame=3|Fragment_1
IFTTQIIVRLYG
>Locus_15616_Transcript_2/3_Confidence_0.667_Length_785|.|sp|Q9VM08|
GR28B_DROME Putative gustatory receptor 28b OS=Drosophila
melanogaster GN=Gr28b PE=1 SV=2|e=3.19208E-17|from=27|to=755|
frame=3|Fragment_2
IYPRASLWFSFVFPNIVSTMIILQFSFYVYILTLRMGLIKTLINNLEIISTSAWLDGDEK
R
>Locus_15616_Transcript_2/3_Confidence_0.667_Length_785|.|sp|Q9VM08|
GR28B_DROME Putative gustatory receptor 28b OS=Drosophila
melanogaster GN=Gr28b PE=1 SV=2|e=3.19208E-17|from=27|to=755|
frame=3|Fragment_3
HTMKQKKYATIIDVIGKLHEKLCEIVDHINAYFNNQMLVLCLDAFVMIVFFAYYSLISIM
DYEADLAKY
>Locus_15616_Transcript_2/3_Confidence_0.667_Length_785|.|sp|Q9VM08|
GR28B_DROME Putative gustatory receptor 28b OS=Drosophila
melanogaster GN=Gr28b PE=1 SV=2|e=3.19208E-17|from=27|to=755|
frame=3|Fragment_4
IGVMAFFLCPFIANICQMIYLVVFCAQFYNEITKCYKKLLVLINESEKRCTKLRLNQLSI

QIIIRSPKFSAGGVFILKPTLLRSMVASSSIYLIILFQFSN
>Locus_15616_Transcript_1/3_Confidence_0.667_Length_708|.|sp|Q9VM08|GR28B_DROME Putative gustatory receptor 28b OS=Drosophila melanogaster GN=Gr28b PE=1 SV=2|e=6.38409E-11|from=27|to=695|frame=3|Fragment_1
IFTTQIIVRLYG
>Locus_15616_Transcript_1/3_Confidence_0.667_Length_708|.|sp|Q9VM08|GR28B_DROME Putative gustatory receptor 28b OS=Drosophila melanogaster GN=Gr28b PE=1 SV=2|e=6.38409E-11|from=27|to=695|frame=3|Fragment_2
IYPRASLWFSFVFPNIVSTMIILQFSFYVYILTLRMGLIKTLINNLEIISTSAWLDGDEK
THNMKQKKY
>Locus_15616_Transcript_1/3_Confidence_0.667_Length_708|.|sp|Q9VM08|GR28B_DROME Putative gustatory receptor 28b OS=Drosophila melanogaster GN=Gr28b PE=1 SV=2|e=6.38409E-11|from=27|to=695|frame=3|Fragment_3
ATIIDVIGKLHEKLCEIVDHINAYFNNQMLVLCLDAFVMIVFFAYYSLISIMDYEADLAK
Y
>Locus_15616_Transcript_1/3_Confidence_0.667_Length_708|.|sp|Q9VM08|GR28B_DROME Putative gustatory receptor 28b OS=Drosophila melanogaster GN=Gr28b PE=1 SV=2|e=6.38409E-11|from=27|to=695|frame=3|Fragment_4
IGVMTFFLCPFIANICQMICLVVFCAQFYNEITNCHKKLLVLINESEKRCTK
>Locus_15616_Transcript_1/3_Confidence_0.667_Length_708|.|sp|Q9VM08|GR28B_DROME Putative gustatory receptor 28b OS=Drosophila melanogaster GN=Gr28b PE=1 SV=2|e=6.38409E-11|from=27|to=695|frame=3|Fragment_5
PKFSAGGVFILKPTL
TRANSCRIPT|sp|Q9VMA7|TGO1_DROME Transport and Golgi organization protein 1 OS=Drosophila melanogaster GN=Tango1 PE=1 SV=2|e=9.84324E-66|from=16|to=1332|frame=1|Fragment_1
QDGSLLRKVNTLEQALMVSSKECEILREKLDSTQTKLTSIKEGAFGSNEEVISLQEEKKK
LEATILDK
>Locus_2188_Transcript_3/6_Confidence_0.529_Length_1584|UNIGENE REPRESENTATIVE TRANSCRIPT|sp|Q9VMA7|TGO1_DROME Transport and Golgi organization protein 1 OS=Drosophila melanogaster GN=Tango1 PE=1 SV=2|e=9.84324E-66|from=16|to=1332|frame=1|Fragment_2
ERQLMDLQKEFEAATETGLELNKMLQDMMMTAQKGDTIAESMEHAQLQLNEQKETINSMS
AALATRNKENNDLQSKLNEYEKQIETAQKLLNQEKSS
>Locus_2188_Transcript_3/6_Confidence_0.529_Length_1584|UNIGENE REPRESENTATIVE TRANSCRIPT|sp|Q9VMA7|TGO1_DROME Transport and Golgi organization protein 1 OS=Drosophila melanogaster GN=Tango1 PE=1 SV=2|e=9.84324E-66|from=16|to=1332|frame=1|Fragment_3
N
>Locus_2188_Transcript_3/6_Confidence_0.529_Length_1584|UNIGENE REPRESENTATIVE TRANSCRIPT|sp|Q9VMA7|TGO1_DROME Transport and Golgi organization protein 1 OS=Drosophila melanogaster GN=Tango1 PE=1 SV=2|e=9.84324E-66|from=16|to=1332|frame=1|Fragment_4
KIREENAYEIQQVRSVLTET
>Locus_2188_Transcript_3/6_Confidence_0.529_Length_1584|UNIGENE REPRESENTATIVE TRANSCRIPT|sp|Q9VMA7|TGO1_DROME Transport and Golgi organization protein 1 OS=Drosophila melanogaster GN=Tango1 PE=1 SV=2|e=9.84324E-66|from=16|to=1332|frame=1|Fragment_5
QRDLYTKTNQVQLLEDCIKKLKSGSQLDVTELADIAGLRSDLQRTTTELQILKEHDAQES

LLKKKLEENVKTLMN
>Locus_2188_Transcript_3/6_Confidence_0.529_Length_1584|UNIGENE REPRESENTATIVE TRANSCRIPT|sp|Q9VMA7|TGO1_DROME Transport and Golgi organization protein 1 OS=Drosophila melanogaster GN=Tango1 PE=1 SV=2|e=9.84324E-66|from=16|to=1332|frame=1|Fragment_6
QTRLEVLSGYFKEKETQLQKELGIQEALWQQKQGEATSTVERLHSMQDEIQSLKSQNQSL
HSEIEQQAAAHRSQVTALESRIHEAWLNSRQTQRKLEESRQEASSLRRKLTSLVE
>Locus_2188_Transcript_3/6_Confidence_0.529_Length_1584|UNIGENE REPRESENTATIVE TRANSCRIPT|sp|Q9VMA7|TGO1_DROME Transport and Golgi organization protein 1 OS=Drosophila melanogaster GN=Tango1 PE=1 SV=2|e=9.84324E-66|from=16|to=1332|frame=1|Fragment_7
QENMQKRGSSPLEVQL
>Locus_2188_Transcript_3/6_Confidence_0.529_Length_1584|UNIGENE REPRESENTATIVE TRANSCRIPT|sp|Q9VMA7|TGO1_DROME Transport and Golgi organization protein 1 OS=Drosophila melanogaster GN=Tango1 PE=1 SV=2|e=9.84324E-66|from=16|to=1332|frame=1|Fragment_8
SAQSPI
>Locus_2188_Transcript_1/6_Confidence_0.588_Length_1584|.|sp|Q9VMA7|TGO1_DROME Transport and Golgi organization protein 1 OS=Drosophila melanogaster GN=Tango1 PE=1 SV=2|e=1.28557E-65|from=16|to=1332|frame=1|Fragment_1
QDGSLLRKVNTLEQALMVSSKECEILREKLDSTQTKLTSIKEGAFGSNEEVISLQEEKKK
LEATILDK
>Locus_2188_Transcript_1/6_Confidence_0.588_Length_1584|.|sp|Q9VMA7|TGO1_DROME Transport and Golgi organization protein 1 OS=Drosophila melanogaster GN=Tango1 PE=1 SV=2|e=1.28557E-65|from=16|to=1332|frame=1|Fragment_2
ERQLMDLQKEFEAATETGLELNKMLQDMMMTAQKGDTIAESMEHAQLQLNEQKETINSMS
AALATRNKENNDLQSKLNEYEKQIETVQKLLNQEKSS
>Locus_2188_Transcript_1/6_Confidence_0.588_Length_1584|.|sp|Q9VMA7|TGO1_DROME Transport and Golgi organization protein 1 OS=Drosophila melanogaster GN=Tango1 PE=1 SV=2|e=1.28557E-65|from=16|to=1332|frame=1|Fragment_3
N
>Locus_2188_Transcript_1/6_Confidence_0.588_Length_1584|.|sp|Q9VMA7|TGO1_DROME Transport and Golgi organization protein 1 OS=Drosophila melanogaster GN=Tango1 PE=1 SV=2|e=1.28557E-65|from=16|to=1332|frame=1|Fragment_4
KIREENAYEIQQVRSVLTET
>Locus_2188_Transcript_1/6_Confidence_0.588_Length_1584|.|sp|Q9VMA7|TGO1_DROME Transport and Golgi organization protein 1 OS=Drosophila melanogaster GN=Tango1 PE=1 SV=2|e=1.28557E-65|from=16|to=1332|frame=1|Fragment_5
QRDLYTKTNQVQLLEDCIKKLKSGSQLDVTELADIAGLRSDLQRTTTELQILKEHDAQES
LLKKKLEENVKTLMN
>Locus_2188_Transcript_1/6_Confidence_0.588_Length_1584|.|sp|Q9VMA7|TGO1_DROME Transport and Golgi organization protein 1 OS=Drosophila melanogaster GN=Tango1 PE=1 SV=2|e=1.28557E-65|from=16|to=1332|frame=1|Fragment_6
QTRLEVLSGYFKEKETQLQKELGIQEALWQQKQGEATSTVERLHSMQDEIQSLKSQNQSL
HSEIEQQAAAHRSQVTALESRIHEAWLNSRQTQRKLEESRQEASSLRRKLTSLVE
>Locus_2188_Transcript_1/6_Confidence_0.588_Length_1584|.|sp|Q9VMA7|TGO1_DROME Transport and Golgi organization protein 1 OS=Drosophila melanogaster GN=Tango1 PE=1 SV=2|e=1.28557E-65|from=16|to=1332|

frame=1|Fragment_7
QENMQKRGSSPLEVQL
>Locus_2188_Transcript_1/6_Confidence_0.588_Length_1584|.|sp|Q9VMA7|
TGO1_DROME Transport and Golgi organization protein 1 OS=Drosophila
melanogaster GN=Tango1 PE=1 SV=2|e=1.28557E-65|from=16|to=1332|
frame=1|Fragment_8
SAQSPI
>Locus_2188_Transcript_2/6_Confidence_0.647_Length_1584|.|sp|Q9VMA7|
TGO1_DROME Transport and Golgi organization protein 1 OS=Drosophila
melanogaster GN=Tango1 PE=1 SV=2|e=1.28557E-65|from=16|to=1332|
frame=1|Fragment_1
QDGSLLRKVNTLEQALMVSSKECEILREKLDSTQTKLTSIKEGAFGSNEEVISLQEEKKK
LEATILDK
>Locus_2188_Transcript_2/6_Confidence_0.647_Length_1584|.|sp|Q9VMA7|
TGO1_DROME Transport and Golgi organization protein 1 OS=Drosophila
melanogaster GN=Tango1 PE=1 SV=2|e=1.28557E-65|from=16|to=1332|
frame=1|Fragment_2
ERQLMDLQKEFEAATETGLELNKMLQDMMMTAQKGDTIAESMEHAQLQLNEQKETINSMS
AALATRNKENNDLQSKLNEYEKQIETVQKLLNQEKSS
>Locus_2188_Transcript_2/6_Confidence_0.647_Length_1584|.|sp|Q9VMA7|
TGO1_DROME Transport and Golgi organization protein 1 OS=Drosophila
melanogaster GN=Tango1 PE=1 SV=2|e=1.28557E-65|from=16|to=1332|
frame=1|Fragment_3
N
>Locus_2188_Transcript_2/6_Confidence_0.647_Length_1584|.|sp|Q9VMA7|
TGO1_DROME Transport and Golgi organization protein 1 OS=Drosophila
melanogaster GN=Tango1 PE=1 SV=2|e=1.28557E-65|from=16|to=1332|
frame=1|Fragment_4
KIREENAYEIQQVRSVLTET
>Locus_2188_Transcript_2/6_Confidence_0.647_Length_1584|.|sp|Q9VMA7|
TGO1_DROME Transport and Golgi organization protein 1 OS=Drosophila
melanogaster GN=Tango1 PE=1 SV=2|e=1.28557E-65|from=16|to=1332|
frame=1|Fragment_5
QRDLYTKTNQVQLLEDCIKKLKSGSQLDVTELADIAGLRSDLQRTTTELQILKEHDAQES
LLKKKLEENVKTLMN
>Locus_2188_Transcript_2/6_Confidence_0.647_Length_1584|.|sp|Q9VMA7|
TGO1_DROME Transport and Golgi organization protein 1 OS=Drosophila
melanogaster GN=Tango1 PE=1 SV=2|e=1.28557E-65|from=16|to=1332|
frame=1|Fragment_6
QTRLEVLSGYFKEKETQLQKELGIQEALWQQKQGEATSTVERLHSMQDEIQSLKSQNQSL
HSEIEQQAAAHRSQVTALESRIHEAWLNSRQTQRKLEESRQEASSLRRKLTSLVE
>Locus_2188_Transcript_2/6_Confidence_0.647_Length_1584|.|sp|Q9VMA7|
TGO1_DROME Transport and Golgi organization protein 1 OS=Drosophila
melanogaster GN=Tango1 PE=1 SV=2|e=1.28557E-65|from=16|to=1332|
frame=1|Fragment_7
QENMQKRGSSPLEVQL
>Locus_2188_Transcript_2/6_Confidence_0.647_Length_1584|.|sp|Q9VMA7|
TGO1_DROME Transport and Golgi organization protein 1 OS=Drosophila
melanogaster GN=Tango1 PE=1 SV=2|e=1.28557E-65|from=16|to=1332|
frame=1|Fragment_8
SAQSPI
>Locus_2188_Transcript_4/6_Confidence_0.706_Length_1584|.|sp|Q9VMA7|
TGO1_DROME Transport and Golgi organization protein 1 OS=Drosophila
melanogaster GN=Tango1 PE=1 SV=2|e=1.28557E-65|from=16|to=1332|

frame=1|Fragment_1
QDGSLLRKVNTLEQALMVSSKECEILREKLDSTQTKLTSIKEGAFGSNEEVISLQEEKKK
LEATILDK
>Locus_2188_Transcript_4/6_Confidence_0.706_Length_1584|.|sp|Q9VMA7|
TGO1_DROME Transport and Golgi organization protein 1 OS=Drosophila
melanogaster GN=Tango1 PE=1 SV=2|e=1.28557E-65|from=16|to=1332|
frame=1|Fragment_2
ERQLMDLQKEFEAATETGLELNKMLQDMMMTAQKGDTIAESMEHAQLQLNEQKETINSMS
AALATRNKENNDLQSKLNEYEKQIETVQKLLNQEKSS
>Locus_2188_Transcript_4/6_Confidence_0.706_Length_1584|.|sp|Q9VMA7|
TGO1_DROME Transport and Golgi organization protein 1 OS=Drosophila
melanogaster GN=Tango1 PE=1 SV=2|e=1.28557E-65|from=16|to=1332|
frame=1|Fragment_3
N
>Locus_2188_Transcript_4/6_Confidence_0.706_Length_1584|.|sp|Q9VMA7|
TGO1_DROME Transport and Golgi organization protein 1 OS=Drosophila
melanogaster GN=Tango1 PE=1 SV=2|e=1.28557E-65|from=16|to=1332|
frame=1|Fragment_4
KIREENAYEIQQVRSVLTET
>Locus_2188_Transcript_4/6_Confidence_0.706_Length_1584|.|sp|Q9VMA7|
TGO1_DROME Transport and Golgi organization protein 1 OS=Drosophila
melanogaster GN=Tango1 PE=1 SV=2|e=1.28557E-65|from=16|to=1332|
frame=1|Fragment_5
QRDLYTKTNQVQLLEDCIKKLKSGSQLDVTELADIAGLRSDLQRTTTELQILKEHDAQES
LLKKKLEENVKTLMN
>Locus_2188_Transcript_4/6_Confidence_0.706_Length_1584|.|sp|Q9VMA7|
TGO1_DROME Transport and Golgi organization protein 1 OS=Drosophila
melanogaster GN=Tango1 PE=1 SV=2|e=1.28557E-65|from=16|to=1332|
frame=1|Fragment_6
QTRLEVLSGYFKEKETQLQKELGIQEALWQQKQGEATSTVERLHSMQDEIQSLKSQNQSL
HSEIEQQAAAHRSQVTALESRIHEAWLNSRQTQRKLEESRQEASSLRRKLTSLVE
>Locus_2188_Transcript_4/6_Confidence_0.706_Length_1584|.|sp|Q9VMA7|
TGO1_DROME Transport and Golgi organization protein 1 OS=Drosophila
melanogaster GN=Tango1 PE=1 SV=2|e=1.28557E-65|from=16|to=1332|
frame=1|Fragment_7
QENMQKRGSSPLEVQL
>Locus_2188_Transcript_4/6_Confidence_0.706_Length_1584|.|sp|Q9VMA7|
TGO1_DROME Transport and Golgi organization protein 1 OS=Drosophila
melanogaster GN=Tango1 PE=1 SV=2|e=1.28557E-65|from=16|to=1332|
frame=1|Fragment_8
SAQSPI
>Locus_2188_Transcript_5/6_Confidence_0.647_Length_1584|.|sp|Q9VMA7|
TGO1_DROME Transport and Golgi organization protein 1 OS=Drosophila
melanogaster GN=Tango1 PE=1 SV=2|e=1.28557E-65|from=16|to=1332|
frame=1|Fragment_1
QDGSLLRKVNTLEQALMVSSKECEILREKLDSTQTKLTSIKEGAFGSNEEVISLQEEKKK
LEATILDK
>Locus_2188_Transcript_5/6_Confidence_0.647_Length_1584|.|sp|Q9VMA7|
TGO1_DROME Transport and Golgi organization protein 1 OS=Drosophila
melanogaster GN=Tango1 PE=1 SV=2|e=1.28557E-65|from=16|to=1332|
frame=1|Fragment_2
ERQLMDLQKEFEAATETGLELNKMLQDMMMTAQKGDTIAESMEHAQLQLNEQKETINSMS
AALATRNKENNDLQSKLNEYEKQIETVQKLLNQEKSS
>Locus_2188_Transcript_5/6_Confidence_0.647_Length_1584|.|sp|Q9VMA7|

TGO1_DROME Transport and Golgi organization protein 1 OS=Drosophila
melanogaster GN=Tango1 PE=1 SV=2|e=1.28557E-65|from=16|to=1332|
frame=1|Fragment_3
N
>Locus_2188_Transcript_5/6_Confidence_0.647_Length_1584|.|sp|Q9VMA7|
TGO1_DROME Transport and Golgi organization protein 1 OS=Drosophila
melanogaster GN=Tango1 PE=1 SV=2|e=1.28557E-65|from=16|to=1332|
frame=1|Fragment_4
KIREENAYEIQQVRSVLTET
>Locus_2188_Transcript_5/6_Confidence_0.647_Length_1584|.|sp|Q9VMA7|
TGO1_DROME Transport and Golgi organization protein 1 OS=Drosophila
melanogaster GN=Tango1 PE=1 SV=2|e=1.28557E-65|from=16|to=1332|
frame=1|Fragment_5
QRDLYTKTNQVQLLEDCIKKLKSGSQLDVTELADIAGLRSDLQRTTTELQILKEHDAQES
LLKKKLEENVKTLMN
>Locus_2188_Transcript_5/6_Confidence_0.647_Length_1584|.|sp|Q9VMA7|
TGO1_DROME Transport and Golgi organization protein 1 OS=Drosophila
melanogaster GN=Tango1 PE=1 SV=2|e=1.28557E-65|from=16|to=1332|
frame=1|Fragment_6
QTRLEVLSGYFKEKETQLQKELGIQEALWQQKQGEATSTVERLHSMQDEIQSLKSQNQSL
HSEIEQQAAAHRSQVTALESRIHEAWLNSRQTQRKLEESRQEASSLRRKLTSLVE
>Locus_2188_Transcript_5/6_Confidence_0.647_Length_1584|.|sp|Q9VMA7|
TGO1_DROME Transport and Golgi organization protein 1 OS=Drosophila
melanogaster GN=Tango1 PE=1 SV=2|e=1.28557E-65|from=16|to=1332|
frame=1|Fragment_7
QENMQKRGSSPLEVQL
>Locus_2188_Transcript_5/6_Confidence_0.647_Length_1584|.|sp|Q9VMA7|
TGO1_DROME Transport and Golgi organization protein 1 OS=Drosophila
melanogaster GN=Tango1 PE=1 SV=2|e=1.28557E-65|from=16|to=1332|
frame=1|Fragment_8
SAQSPI
>Locus_2188_Transcript_6/6_Confidence_0.647_Length_1584|.|sp|Q9VMA7|
TGO1_DROME Transport and Golgi organization protein 1 OS=Drosophila
melanogaster GN=Tango1 PE=1 SV=2|e=1.28557E-65|from=16|to=1332|
frame=1|Fragment_1
QDGSLLRKVNTLEQALMVSSKECEILREKLDSTQTKLTSIKEGAFGSNEEVISLQEEKKK
LEATILDK
>Locus_2188_Transcript_6/6_Confidence_0.647_Length_1584|.|sp|Q9VMA7|
TGO1_DROME Transport and Golgi organization protein 1 OS=Drosophila
melanogaster GN=Tango1 PE=1 SV=2|e=1.28557E-65|from=16|to=1332|
frame=1|Fragment_2
ERQLMDLQKEFEAATETGLELNKMLQDMMMTAQKGDTIAESMEHAQLQLNEQKETINSMS
AALATRNKENNDLQSKLNEYEKQIETVQKLLNQEKSS
>Locus_2188_Transcript_6/6_Confidence_0.647_Length_1584|.|sp|Q9VMA7|
TGO1_DROME Transport and Golgi organization protein 1 OS=Drosophila
melanogaster GN=Tango1 PE=1 SV=2|e=1.28557E-65|from=16|to=1332|
frame=1|Fragment_3
N
>Locus_2188_Transcript_6/6_Confidence_0.647_Length_1584|.|sp|Q9VMA7|
TGO1_DROME Transport and Golgi organization protein 1 OS=Drosophila
melanogaster GN=Tango1 PE=1 SV=2|e=1.28557E-65|from=16|to=1332|
frame=1|Fragment_4
KIREENAYEIQQVRSVLTET
>Locus_2188_Transcript_6/6_Confidence_0.647_Length_1584|.|sp|Q9VMA7|

TGO1_DROME Transport and Golgi organization protein 1 OS=Drosophila melanogaster GN=Tango1 PE=1 SV=2|e=1.28557E-65|from=16|to=1332|frame=1|Fragment_5
QRDLYTKTNQVQLLEDCIKKLKSGSQLDVTELADIAGLRSDLQRTTTELQILKEHDAQES
LLKKKLEENVKTLMN
>Locus_2188_Transcript_6/6_Confidence_0.647_Length_1584|.|sp|Q9VMA7|TGO1_DROME Transport and Golgi organization protein 1 OS=Drosophila melanogaster GN=Tango1 PE=1 SV=2|e=1.28557E-65|from=16|to=1332|frame=1|Fragment_6
QTRLEVLSGYFKEKETQLQKELGIQEALWQQKQGEATSTVERLHSMQDEIQSLKSQNQSL
HSEIEQQAAAHRSQVTALESRIHEAWLNSRQTQRKLEESRQEASSLRRKLTSLVE
>Locus_2188_Transcript_6/6_Confidence_0.647_Length_1584|.|sp|Q9VMA7|TGO1_DROME Transport and Golgi organization protein 1 OS=Drosophila melanogaster GN=Tango1 PE=1 SV=2|e=1.28557E-65|from=16|to=1332|frame=1|Fragment_7
QENMQKRGSSPLEVQL
>Locus_2188_Transcript_6/6_Confidence_0.647_Length_1584|.|sp|Q9VMA7|TGO1_DROME Transport and Golgi organization protein 1 OS=Drosophila melanogaster GN=Tango1 PE=1 SV=2|e=1.28557E-65|from=16|to=1332|frame=1|Fragment_8
SAQSPI
>Locus_7633_Transcript_1/1_Confidence_1.000_Length_383|UNIGENE REPRESENTATIVE TRANSCRIPT|sp|Q9VMW8|MPU1_DROME Mannose-P-dolichol utilization defect 1 protein homolog OS=Drosophila melanogaster GN=CG3792 PE=1 SV=2|e=1.16228E-12|from=3|to=200|frame=-1|Fragment_1
CYHNYFEDFNFLDVPCFKAT
>Locus_7633_Transcript_1/1_Confidence_1.000_Length_383|UNIGENE REPRESENTATIVE TRANSCRIPT|sp|Q9VMW8|MPU1_DROME Mannose-P-dolichol utilization defect 1 protein homolog OS=Drosophila melanogaster GN=CG3792 PE=1 SV=2|e=1.16228E-12|from=3|to=200|frame=-1|Fragment_2
LVKVPQILKISSNKSGQGINILSVLLDLFVIT
>Locus_13968_Transcript_1/1_Confidence_1.000_Length_383|.|sp|Q9VMW8|MPU1_DROME Mannose-P-dolichol utilization defect 1 protein homolog OS=Drosophila melanogaster GN=CG3792 PE=1 SV=2|e=1.16228E-12|from=3|to=200|frame=-1|Fragment_1
CYHNYFEDFNFLDVPCFKAT
>Locus_13968_Transcript_1/1_Confidence_1.000_Length_383|.|sp|Q9VMW8|MPU1_DROME Mannose-P-dolichol utilization defect 1 protein homolog OS=Drosophila melanogaster GN=CG3792 PE=1 SV=2|e=1.16228E-12|from=3|to=200|frame=-1|Fragment_2
LVKVPQILKISSNKSGQGINILSVLLDLFVIT
>Locus_1489_Transcript_1/3_Confidence_0.714_Length_2216|UNIGENE REPRESENTATIVE TRANSCRIPT|sp|Q9VNB5|OR83B_DROME Odorant receptor coreceptor OS=Drosophila melanogaster GN=Orco PE=1 SV=2|e=6.5818E-175|from=953|to=2215|frame=-2|Fragment_1
QYGSLFANLATHTGDVAELTANTITVLFFLHPITKNIYFAVNSASFYRTWGIWNQSNSHP
LFAESNARYHSIALAKMRRLLVAVVITSLLSVAAWTTLTFFGDSTRTVMDMETNETMTEP
VPRLMLRAWYPWPSMSGPMYMISFGLQLYYLTFSLLQSNLLDVLFCSWLIFACEQLQHLK
GIMRPLMELSSVLDTYRPDSAALFRTTSANSQTELIPNDDNDV
>Locus_1489_Transcript_1/3_Confidence_0.714_Length_2216|UNIGENE REPRESENTATIVE TRANSCRIPT|sp|Q9VNB5|OR83B_DROME Odorant receptor coreceptor OS=Drosophila melanogaster GN=Orco PE=1 SV=2|e=6.5818E-175|from=953|to=2215|frame=-2|Fragment_2
KDIDAAAIYNSKADWGAHYRGPSVLQNFNQ

>Locus_1489_Transcript_1/3_Confidence_0.714_Length_2216|UNIGENE REPRESENTATIVE TRANSCRIPT|sp|Q9VNB5|OR83B_DROME Odorant receptor coreceptor OS=Drosophila melanogaster GN=Orco PE=1 SV=2|e=6.5818E-175|from=953|to=2215|frame=-2|Fragment_3
GAN
>Locus_1489_Transcript_1/3_Confidence_0.714_Length_2216|UNIGENE REPRESENTATIVE TRANSCRIPT|sp|Q9VNB5|OR83B_DROME Odorant receptor coreceptor OS=Drosophila melanogaster GN=Orco PE=1 SV=2|e=6.5818E-175|from=953|to=2215|frame=-2|Fragment_4
NPNGLTKKQEMMVRSAIKYWVERHKHVVRLVAGIGDTYGGALLLHMLTSTITLTLLAYQA
TKIDRLNVFAFGVIGYLVYSLAQVFLFCVFGNRLIEESSSVMEAAYSCHWYDGSEEAKTF
VQIVCQQCQKAMTISGAKFFTVSLDLFASVLGAVVTYFMVLVQLK
>Locus_1489_Transcript_2/3_Confidence_0.714_Length_2216|.|sp|Q9VNB5|OR83B_DROME Odorant receptor coreceptor OS=Drosophila melanogaster GN=Orco PE=1 SV=2|e=6.5818E-175|from=953|to=2215|frame=-2|Fragment_1
QYGSLFANLATHTGDVAELTANTITVLFFLHPITKNIYFAVNSASFYRTWGIWNQSNSHP
LFAESNARYHSIALAKMRRLLVAVVITSLLSVAAWTTLTFFGDSTRTVMDMETNETMTEP
VPRLMLRAWYPWPSMSGPMYMISFGLQLYYLTFSLLQSNLLDVLFCSWLIFACEQLQHLK
GIMRPLMELSSVLDTYRPDSAALFRTTSANSQTELIPNDDNDV
>Locus_1489_Transcript_2/3_Confidence_0.714_Length_2216|.|sp|Q9VNB5|OR83B_DROME Odorant receptor coreceptor OS=Drosophila melanogaster GN=Orco PE=1 SV=2|e=6.5818E-175|from=953|to=2215|frame=-2|Fragment_2
KDIDAAAIYNSKADWGAHYRGPSVLQNFNQ
>Locus_1489_Transcript_2/3_Confidence_0.714_Length_2216|.|sp|Q9VNB5|OR83B_DROME Odorant receptor coreceptor OS=Drosophila melanogaster GN=Orco PE=1 SV=2|e=6.5818E-175|from=953|to=2215|frame=-2|Fragment_3
GAN
>Locus_1489_Transcript_2/3_Confidence_0.714_Length_2216|.|sp|Q9VNB5|OR83B_DROME Odorant receptor coreceptor OS=Drosophila melanogaster GN=Orco PE=1 SV=2|e=6.5818E-175|from=953|to=2215|frame=-2|Fragment_4
NPNGLTKKQEMMVRSAIKYWVERHKHVVRLVAGIGDTYGGALLLHMLTSTITLTLLAYQA
TKIDRLNVFAFGVIGYLVYSLAQVFLFCVFGNRLIEESSSVMEAAYSCHWYDGSEEAKTF
VQIVCQQCQKAMTISGAKFFTVSLDLFASVLGAVVTYFMVLVQLK
>Locus_1489_Transcript_3/3_Confidence_0.714_Length_2216|.|sp|Q9VNB5|OR83B_DROME Odorant receptor coreceptor OS=Drosophila melanogaster GN=Orco PE=1 SV=2|e=6.5818E-175|from=953|to=2215|frame=-2|Fragment_1
QYGSLFANLATHTGDVAELTANTITVLFFLHPITKNIYFAVNSASFYRTWGIWNQSNSHP
LFAESNARYHSIALAKMRRLLVAVVITSLLSVAAWTTLTFFGDSTRTVMDMETNETMTEP
VPRLMLRAWYPWPSMSGPMYMISFGLQLYYLTFSLLQSNLLDVLFCSWLIFACEQLQHLK
GIMRPLMELSSVLDTYRPDSAALFRTTSANSQTELIPNDDNDV
>Locus_1489_Transcript_3/3_Confidence_0.714_Length_2216|.|sp|Q9VNB5|OR83B_DROME Odorant receptor coreceptor OS=Drosophila melanogaster GN=Orco PE=1 SV=2|e=6.5818E-175|from=953|to=2215|frame=-2|Fragment_2
KDIDAAAIYNSKADWGAHYRGPSVLQNFNQ
>Locus_1489_Transcript_3/3_Confidence_0.714_Length_2216|.|sp|Q9VNB5|OR83B_DROME Odorant receptor coreceptor OS=Drosophila melanogaster GN=Orco PE=1 SV=2|e=6.5818E-175|from=953|to=2215|frame=-2|Fragment_3
GAN
>Locus_1489_Transcript_3/3_Confidence_0.714_Length_2216|.|sp|Q9VNB5|OR83B_DROME Odorant receptor coreceptor OS=Drosophila melanogaster GN=Orco PE=1 SV=2|e=6.5818E-175|from=953|to=2215|frame=-2|Fragment_4
NPNGLTKKQEMMVRSAIKYWVERHKHVVRLVAGIGDTYGGALLLHMLTSTITLTLLAYQA
TKIDRLNVFAFGVIGYLVYSLAQVFLFCVFGNRLIEESSSVMEAAYSCHWYDGSEEAKTF
VQIVCQQCQKAMTISGAKFFTVSLDLFASVLGAVVTYFMVLVQLK

>Locus_6731_Transcript_3/3_Confidence_0.667_Length_2064|UNIGENE REPRESENTATIVE TRANSCRIPT|sp|Q9VPF8|TM104_DROME Transmembrane protein 104 homolog OS=Drosophila melanogaster GN=CG5262 PE=2 SV=2|e=1.155E-149|from=442|to=1860|frame=-1|Fragment_1
YSSWMGLVYVFNLIVGTGALTLPAVFARAGWGLGLAAIIVLAFISFVTVTFVIETMSCAN
ALMHWKRLGLLKREKCITD
>Locus_6731_Transcript_3/3_Confidence_0.667_Length_2064|UNIGENE REPRESENTATIVE TRANSCRIPT|sp|Q9VPF8|TM104_DROME Transmembrane protein 104 homolog OS=Drosophila melanogaster GN=CG5262 PE=2 SV=2|e=1.155E-149|from=442|to=1860|frame=-1|Fragment_2
EDCADEIS
>Locus_6731_Transcript_3/3_Confidence_0.667_Length_2064|UNIGENE REPRESENTATIVE TRANSCRIPT|sp|Q9VPF8|TM104_DROME Transmembrane protein 104 homolog OS=Drosophila melanogaster GN=CG5262 PE=2 SV=2|e=1.155E-149|from=442|to=1860|frame=-1|Fragment_3
EDSFEIDNLERHPL
>Locus_6731_Transcript_3/3_Confidence_0.667_Length_2064|UNIGENE REPRESENTATIVE TRANSCRIPT|sp|Q9VPF8|TM104_DROME Transmembrane protein 104 homolog OS=Drosophila melanogaster GN=CG5262 PE=2 SV=2|e=1.155E-149|from=442|to=1860|frame=-1|Fragment_4
MSRMPRFYEIDHQVELGEMASMFFNTAGRSVFYLCLCIYLYGDLSIYCAAVSESLVDITC
>Locus_6731_Transcript_3/3_Confidence_0.667_Length_2064|UNIGENE REPRESENTATIVE TRANSCRIPT|sp|Q9VPF8|TM104_DROME Transmembrane protein 104 homolog OS=Drosophila melanogaster GN=CG5262 PE=2 SV=2|e=1.155E-149|from=442|to=1860|frame=-1|Fragment_5
YIPANNTNNTILPCFPSEENSISRITVYNLCLSAFFCLMGPFAFFNVQKTKYLQMITSFL
RWLAFSIMIGIASWRLATQGPQSQPSVAQVSGVPGLFGACVYSFMCHHSIPSLVAPIRNK
SSLKKLLSCDYFLICVFYIVLAVTGTFAFANLEDLYTLNFLPSPD
>Locus_6731_Transcript_3/3_Confidence_0.667_Length_2064|UNIGENE REPRESENTATIVE TRANSCRIPT|sp|Q9VPF8|TM104_DROME Transmembrane protein 104 homolog OS=Drosophila melanogaster GN=CG5262 PE=2 SV=2|e=1.155E-149|from=442|to=1860|frame=-1|Fragment_6
SVMMTLAHYFLALFPVFTLSASFPIIAITLRNNLQTLFLDNSRIESYNFFVRKMCFPLLA
IVPPFLVAIGARSLSGLVGFTGSYAGTGVQYLVPIGLVYSARRQCNTIPALRHENQFKSP
FKSNLWLIFILLWSIACVVLVSVNLFA
>Locus_6731_Transcript_2/3_Confidence_0.667_Length_2064|.|sp|Q9VPF8|TM104_DROME Transmembrane protein 104 homolog OS=Drosophila melanogaster GN=CG5262 PE=2 SV=2|e=2.573E-149|from=442|to=1860|frame=-1|Fragment_1
YSSWMGLVYVFNLIVGTGALTLPAVFSRAGWGLGLAAIIVLAFISFVTVTFVIETMSCAN
ALMHWKRLGLLKREKCITD
>Locus_6731_Transcript_2/3_Confidence_0.667_Length_2064|.|sp|Q9VPF8|TM104_DROME Transmembrane protein 104 homolog OS=Drosophila melanogaster GN=CG5262 PE=2 SV=2|e=2.573E-149|from=442|to=1860|frame=-1|Fragment_2
EDCADEIS
>Locus_6731_Transcript_2/3_Confidence_0.667_Length_2064|.|sp|Q9VPF8|TM104_DROME Transmembrane protein 104 homolog OS=Drosophila melanogaster GN=CG5262 PE=2 SV=2|e=2.573E-149|from=442|to=1860|frame=-1|Fragment_3
EDSFEIDNLERHPL
>Locus_6731_Transcript_2/3_Confidence_0.667_Length_2064|.|sp|Q9VPF8|TM104_DROME Transmembrane protein 104 homolog OS=Drosophila melanogaster GN=CG5262 PE=2 SV=2|e=2.573E-149|from=442|to=1860|

frame=-1|Fragment_4
MSRMPRFYEIDHQVELGEMASMFFNTAGRSVFYLCLCIYLYGDLSIYCAAVSESLVDITC
>Locus_6731_Transcript_2/3_Confidence_0.667_Length_2064|.|sp|Q9VPF8|
TM104_DROME Transmembrane protein 104 homolog OS=Drosophila
melanogaster GN=CG5262 PE=2 SV=2|e=2.573E-149|from=442|to=1860|
frame=-1|Fragment_5
YIPANNTNNTILPCFPSEENSISRITVYNLCLSAFFCLMGPFAFFNVQKTKYLQMITSFL
RWLAFSIMIGIASWRLATQGPQSQPSVAQVSGVPGLFGACVYSFMCHHSIPSLVAPIRNK
SSLKKLLSCDYFLICVFYIVLAVTGTFAFANLEDLYTLNFLPSPD
>Locus_6731_Transcript_2/3_Confidence_0.667_Length_2064|.|sp|Q9VPF8|
TM104_DROME Transmembrane protein 104 homolog OS=Drosophila
melanogaster GN=CG5262 PE=2 SV=2|e=2.573E-149|from=442|to=1860|
frame=-1|Fragment_6
SVMMTLAHYFLALFPVFTLSASFPIIAITLRNNLQTLFLDNSRIESYNFFVRKMCFPLLA
IVPPFLVAIGARSLSGLVGFTGSYAGTGVQYLVPIGLVYSARRQCNTIPALRHENQFKSP
FKSNLWLIFILLWSIACVVLVSVNLFA
>Locus_6731_Transcript_1/3_Confidence_0.667_Length_1984|.|sp|Q9VPF8|
TM104_DROME Transmembrane protein 104 homolog OS=Drosophila
melanogaster GN=CG5262 PE=2 SV=2|e=1.1003E-149|from=362|to=1780|
frame=-1|Fragment_1
YSSWMGLVYVFNLIVGTGALTLPAVFARAGWGLGLAAIIVLAFISFVTVTFVIETMSCAN
ALMHWKRLGLLKREKCITD
>Locus_6731_Transcript_1/3_Confidence_0.667_Length_1984|.|sp|Q9VPF8|
TM104_DROME Transmembrane protein 104 homolog OS=Drosophila
melanogaster GN=CG5262 PE=2 SV=2|e=1.1003E-149|from=362|to=1780|
frame=-1|Fragment_2
EDCADEIS
>Locus_6731_Transcript_1/3_Confidence_0.667_Length_1984|.|sp|Q9VPF8|
TM104_DROME Transmembrane protein 104 homolog OS=Drosophila
melanogaster GN=CG5262 PE=2 SV=2|e=1.1003E-149|from=362|to=1780|
frame=-1|Fragment_3
EDSFEIDNLERHPL
>Locus_6731_Transcript_1/3_Confidence_0.667_Length_1984|.|sp|Q9VPF8|
TM104_DROME Transmembrane protein 104 homolog OS=Drosophila
melanogaster GN=CG5262 PE=2 SV=2|e=1.1003E-149|from=362|to=1780|
frame=-1|Fragment_4
MSRMPRFYEIDHQVELGEMASMFFNTAGRSVFYLCLCIYLYGDLSIYCAAVSESLVDITC
>Locus_6731_Transcript_1/3_Confidence_0.667_Length_1984|.|sp|Q9VPF8|
TM104_DROME Transmembrane protein 104 homolog OS=Drosophila
melanogaster GN=CG5262 PE=2 SV=2|e=1.1003E-149|from=362|to=1780|
frame=-1|Fragment_5
YIPANNTNNTILPCFPSEENSISRITVYNLCLSAFFCLMGPFAFFNVQKTKYLQMITSFL
RWLAFSIMIGIASWRLATQGPQSQPSVAQVSGVPGLFGACVYSFMCHHSIPSLVAPIRNK
SSLKKLLSCDYFLICVFYIVLAVTGTFAFANLEDLYTLNFLPSPD
>Locus_6731_Transcript_1/3_Confidence_0.667_Length_1984|.|sp|Q9VPF8|
TM104_DROME Transmembrane protein 104 homolog OS=Drosophila
melanogaster GN=CG5262 PE=2 SV=2|e=1.1003E-149|from=362|to=1780|
frame=-1|Fragment_6
SVMMTLAHYFLALFPVFTLSASFPIIAITLRNNLQTLFLDNSRIESYNFFVRKMCFPLLA
IVPPFLVAIGARSLSGLVGFTGSYAGTGVQYLVPIGLVYSARRQCNTIPALRHENQFKSP
FKSNLWLIFILLWSIACVVLVSVNLFA
>Locus_1834_Transcript_6/13_Confidence_0.583_Length_4374|UNIGENE
REPRESENTATIVE TRANSCRIPT|sp|Q9VQH2|DUOX_DROME Dual oxidase
OS=Drosophila melanogaster GN=Duox PE=1 SV=2|e=0|from=677|to=4372|

frame=2|Fragment_1
LFVGLLTYCSCLGS
>Locus_1834_Transcript_6/13_Confidence_0.583_Length_4374|UNIGENE REPRESENTATIVE TRANSCRIPT|sp|Q9VQH2|DUOX_DROME Dual oxidase OS=Drosophila melanogaster GN=Duox PE=1 SV=2|e=0|from=677|to=4372|frame=2|Fragment_2
EKILSNTEKQRYDGWYNNLAHPDWGSVGSHLTRKTPPAYADGVYALAGQGRPSPRRLSRL
FMKGQDGLASKMNRTALLAFFGQVVTSEIVMASESGCPIEMHRIEIDRCDEMYDPECRGD
RYIPFHRAAYDRRTGMSPNMPREQINQMTSWIDGSFIYSSSEPWLNAMRSFHNGTLLGEE
TSHGTMPIRNTMRVPLFNNPVPHVMRMLSPERLFLLGDPRSNQNPALLAFGILFFRWHNV
IAGRVQRQHPTWPDEEVFQRARRIVVASLQNIIAYEYLPAFLGEELPEYTGYRPDVHPGV
SHVFQSAAFRFGHTMIPPGMYRRDGKCNYKRTAAGHQAIRLCATWWDSNDV
>Locus_1834_Transcript_6/13_Confidence_0.583_Length_4374|UNIGENE REPRESENTATIVE TRANSCRIPT|sp|Q9VQH2|DUOX_DROME Dual oxidase OS=Drosophila melanogaster GN=Duox PE=1 SV=2|e=0|from=677|to=4372|frame=2|Fragment_3
GMASQIAEREDSLLCSDVRDKLFGPMEFTRRDLGALNIMRGRDNGLPDYNTARSYFRLPK
VNDWTEINPELFAARPELLGLLVAAYNNRLDNVDVYVGGMLESDGKPGELFTAVIREQFV
RIRDADRFWFENE
>Locus_1834_Transcript_6/13_Confidence_0.583_Length_4374|UNIGENE REPRESENTATIVE TRANSCRIPT|sp|Q9VQH2|DUOX_DROME Dual oxidase OS=Drosophila melanogaster GN=Duox PE=1 SV=2|e=0|from=677|to=4372|frame=2|Fragment_4
NGIFTKEEIEDIKKITLWDIIVNSTSIEPHEIQKDVFRFAEGDPCPQPAQLNASLLEPCK
YLRGYDYFEGSELAYIYVCVLLGFVPILCAAAGYGV
>Locus_1834_Transcript_6/13_Confidence_0.583_Length_4374|UNIGENE REPRESENTATIVE TRANSCRIPT|sp|Q9VQH2|DUOX_DROME Dual oxidase OS=Drosophila melanogaster GN=Duox PE=1 SV=2|e=0|from=677|to=4372|frame=2|Fragment_5
EALKSKAEGKGGSVDKMIVREWLHANHKRLVKVRFGPEAALHTVDRKGEKLRTVSFKNVD
SVTIEESQDNYHNKKPLLLMRIPRDHDLVLELDSLGSRRKFVSKLESFLLSHKKHLICIQ
QMRDMILAKAETRERRQKKLEHFFREAYALTFGLRP
>Locus_1834_Transcript_6/13_Confidence_0.583_Length_4374|UNIGENE REPRESENTATIVE TRANSCRIPT|sp|Q9VQH2|DUOX_DROME Dual oxidase OS=Drosophila melanogaster GN=Duox PE=1 SV=2|e=0|from=677|to=4372|frame=2|Fragment_6
VVTVMRTSLSKGEFASALGMKSDAVFVRKMFNIVDKDGDGRISFQEFLDTVVLFSRGKTE
DKLRIIFDMCDNDRNGVIDKGELSEMLRSLVEIARTTSLSDDQVTELIDGMFQDAGLEHK
DSLTYVDFKQMMKEYKGDFVAIGLDCKGAKQNFLDTSTNVARMTSFHIEPAMEKRRPYLL
RQWDCLTTLLEENRQNIFYLFIFYVITIALFVERFIHYSFMAEHTDLRHIMGVGIAITRG
SAASLSFCYSLLLLTMSRNLITKLKQFAVQQYIPLDSHVQFHKIAACTALFFSLLHTVGH
IVNFYHVSTQPVEHLKCMTREVHFTSDYKPDISFWMFQTITGVTGVMLFITMCIIFVFAH
PTIRKKAYSYFWRAHSLYVLLYALCLVHGLARLTGAPRFWLFFIGPGIIYALDKIVTLRT
KYMALDVIETELLPSDVI
>Locus_1834_Transcript_7/13_Confidence_0.583_Length_4374|.|sp|Q9VQH2|DUOX_DROME Dual oxidase OS=Drosophila melanogaster GN=Duox PE=1 SV=2|e=0|from=677|to=4372|frame=2|Fragment_1
LFVGLLTYCSCLGS
>Locus_1834_Transcript_7/13_Confidence_0.583_Length_4374|.|sp|Q9VQH2|DUOX_DROME Dual oxidase OS=Drosophila melanogaster GN=Duox PE=1 SV=2|e=0|from=677|to=4372|frame=2|Fragment_2
EKILSNTEKQRYDGWYNNLAHPDWGSVGSHLTRKTPPAYADGVYALAGQGRPSPRRLSRL
FMKGQDGLASKMNRTALLAFFGQVVTSEIVMASESGCPIEMHRIEIDRCDEMYDPECRGD
RYIPFHRAAYDRRTGMSPNMPREQINQMTSWIDGSFIYSSSEPWLNAMRSFHNGTLLGEE

TSHGTMPIRNTMRVPLFNNPVPHVMRMLSPERLFLLGDPRSNQNPALLAFGILFFRWHNV
IAGRVQRQHPTWPDEEVFQRARRIVVASLQNIIAYEYLPAFLGEELPEYTGYRPDVHPGV
SHVFQSAAFRFGHTMIPPGMYRRDGKCNYKRTAAGHQAIRLCATWWDSNDV
>Locus_1834_Transcript_7/13_Confidence_0.583_Length_4374|.|sp|
Q9VQH2|DUOX_DROME Dual oxidase OS=Drosophila melanogaster GN=Duox
PE=1 SV=2|e=0|from=677|to=4372|frame=2|Fragment_3
GMASQIAEREDSLLCSDVRDKLFGPMEFTRRDLGALNIMRGRDNGLPDYNTARSYFRLPK
VNDWTEINPELFAARPELLGLLVAAYNNRLDNVDVYVGGMLESDGKPGELFTAVIREQFV
RIRDADRFWFENE
>Locus_1834_Transcript_7/13_Confidence_0.583_Length_4374|.|sp|
Q9VQH2|DUOX_DROME Dual oxidase OS=Drosophila melanogaster GN=Duox
PE=1 SV=2|e=0|from=677|to=4372|frame=2|Fragment_4
NGIFTKEEIEDIKKITLWDIIVNSTSIEPHEIQKDVFRFAEGDPCPQPAQLNASLLEPCK
YLRGYDYFEGSELAYIYVCVLLGFVPILCAAAGYGV
>Locus_1834_Transcript_7/13_Confidence_0.583_Length_4374|.|sp|
Q9VQH2|DUOX_DROME Dual oxidase OS=Drosophila melanogaster GN=Duox
PE=1 SV=2|e=0|from=677|to=4372|frame=2|Fragment_5
EALKSKAEGKGGSVDKMIVREWLHANHKRLVKVRFGPEAALHTVDRKGEKLRTVSFKNVD
SVTIEESQDNYHNKKPLLLMRIPRDHDLVLELDSLGSRRKFVSKLESFLLSHKKHLICIQ
QMRDMILAKAETRERRQKKLEHFFREAYALTFGLRP
>Locus_1834_Transcript_7/13_Confidence_0.583_Length_4374|.|sp|
Q9VQH2|DUOX_DROME Dual oxidase OS=Drosophila melanogaster GN=Duox
PE=1 SV=2|e=0|from=677|to=4372|frame=2|Fragment_6
VVTVMRTSLSKGEFASALGMKSDAVFVRKMFNIVDKDGDGRISFQEFLDTVVLFSRGKTE
DKLRIIFDMCDNDRNGVIDKGELSEMLRSLVEIARTTSLSDDQVTELIDGMFQDAGLEHK
DSLTYVDFKQMMKEYKGDFVAIGLDCKGAKQNFLDTSTNVARMTSFHIEPAMEKRRPYLL
RQWDCLTTLLEENRQNIFYLFIFYVITIALFVERFIHYSFMAEHTDLRHIMGVGIAITRG
SAASLSFCYSLLLLTMSRNLITKLKQFAVQQYIPLDSHVQFHKIAACTALFFSLLHTVGH
IVNFYHVSTQPVEHLKCMTREVHFTSDYKPDISFWMFQTITGVTGVMLFITMCIIFVFAH
PTIRKKAYSYFWRAHSLYVLLYALCLVHGLARLTGAPRFWLFFIGPGIIYALDKIVTLRT
KYMALDVIETELLPSDVI
>Locus_1834_Transcript_8/13_Confidence_0.528_Length_4374|.|sp|
Q9VQH2|DUOX_DROME Dual oxidase OS=Drosophila melanogaster GN=Duox
PE=1 SV=2|e=0|from=677|to=4372|frame=2|Fragment_1
LFVGLLTYCSCLGS
>Locus_1834_Transcript_8/13_Confidence_0.528_Length_4374|.|sp|
Q9VQH2|DUOX_DROME Dual oxidase OS=Drosophila melanogaster GN=Duox
PE=1 SV=2|e=0|from=677|to=4372|frame=2|Fragment_2
EKILSNTEKQRYDGWYNNLAHPDWGSVGSHLTRKTPPAYADGVYALAGQGRPSPRRLSRL
FMKGQDGLASKMNRTALLAFFGQVVTSEIVMASESGCPIEMHRIEIDRCDEMYDPECRGD
RYIPFHRAAYDRRTGMSPNMPREQINQMTSWIDGSFIYSSSEPWLNAMRSFHNGTLLGEE
TSHGTMPIRNTMRVPLFNNPVPHVMRMLSPERLFLLGDPRSNQNPALLAFGILFFRWHNV
IAGRVQRQHPTWPDEEVFQRARRIVVASLQNIIAYEYLPAFLGEELPEYTGYRPDVHPGV
SHVFQSAAFRFGHTMIPPGMYRRDGKCNYKRTAAGHQAIRLCATWWDSNDV
>Locus_1834_Transcript_8/13_Confidence_0.528_Length_4374|.|sp|
Q9VQH2|DUOX_DROME Dual oxidase OS=Drosophila melanogaster GN=Duox
PE=1 SV=2|e=0|from=677|to=4372|frame=2|Fragment_3
GMASQIAEREDSLLCSDVRDKLFGPMEFTRRDLGALNIMRGRDNGLPDYNTARSYFRLPK
VNDWTEINPELFAARPELLGLLVAAYNNRLDNVDVYVGGMLESDGKPGELFTAVIREQFV
RIRDADRFWFENE
>Locus_1834_Transcript_8/13_Confidence_0.528_Length_4374|.|sp|
Q9VQH2|DUOX_DROME Dual oxidase OS=Drosophila melanogaster GN=Duox
PE=1 SV=2|e=0|from=677|to=4372|frame=2|Fragment_4
NGIFTKEEIEDIKKITLWDIIVNSTSIEPHEIQKDVFRFAEGDPCPQPAQLNASLLEPCK

YLRGYDYFEGSELAYIYVCVLLGFVPILCAAAGYGV
>Locus_1834_Transcript_8/13_Confidence_0.528_Length_4374|.|sp|
Q9VQH2|DUOX_DROME Dual oxidase OS=Drosophila melanogaster GN=Duox
PE=1 SV=2|e=0|from=677|to=4372|frame=2|Fragment_5
EALKSKAEGKGGSVDKMIVREWLHANHKRLVKVRFGPEAALHTVDRKGEKLRTVSFKNVD
SVTIEESQDNYHNKKPLLLMRIPRDHDLVLELDSLGSRRKFVSKLESFLLSHKKHLICIQ
QMRDMILAKAETRERRQKKLEHFFREAYALTFGLRP
>Locus_1834_Transcript_8/13_Confidence_0.528_Length_4374|.|sp|
Q9VQH2|DUOX_DROME Dual oxidase OS=Drosophila melanogaster GN=Duox
PE=1 SV=2|e=0|from=677|to=4372|frame=2|Fragment_6
VVTVMRTSLSKGEFASALGMKSDAVFVRKMFNIVDKDGDGRISFQEFLDTVVLFSRGKTE
DKLRIIFDMCDNDRNGVIDKGELSEMLRSLVEIARTTSLSDDQVTELIDGMFQDAGLEHK
DSLTYVDFKQMMKEYKGDFVAIGLDCKGAKQNFLDTSTNVARMTSFHIEPAMEKRRPYLL
RQWDCLTTLLEENRQNIFYLFIFYVITIALFVERFIHYSFMAEHTDLRHIMGVGIAITRG
SAASLSFCYSLLLLTMSRNLITKLKQFAVQQYIPLDSHVQFHKIAACTALFFSLLHTVGH
IVNFYHVSTQPVEHLKCMTREVHFTSDYKPDISFWMFQTITGVTGVMLFITMCIIFVFAH
PTIRKKAYSYFWRAHSLYVLLYALCLVHGLARLTGAPRFWLFFIGPGIIYALDKIVTLRT
KYMALDVIETELLPSDVI
>Locus_1834_Transcript_9/13_Confidence_0.583_Length_4374|.|sp|
Q9VQH2|DUOX_DROME Dual oxidase OS=Drosophila melanogaster GN=Duox
PE=1 SV=2|e=0|from=677|to=4372|frame=2|Fragment_1
LFVGLLTYCSCLGS
>Locus_1834_Transcript_9/13_Confidence_0.583_Length_4374|.|sp|
Q9VQH2|DUOX_DROME Dual oxidase OS=Drosophila melanogaster GN=Duox
PE=1 SV=2|e=0|from=677|to=4372|frame=2|Fragment_2
EKILSNTEKQRYDGWYNNLAHPDWGSVGSHLTRKTPPAYADGVYALAGQGRPSPRRLSRL
FMKGQDGLASKMNRTALLAFFGQVVTSEIVMASESGCPIEMHRIEIDRCDEMYDPECRGD
RYIPFHRAAYDRRTGMSPNMPREQINQMTSWIDGSFIYSSSEPWLNAMRSFHNGTLLGEE
TSHGTMPIRNTMRVPLFNNPVPHVMRMLSPERLFLLGDPRSNQNPALLAFGILFFRWHNV
IAGRVQRQHPTWPDEEVFQRARRIVVASLQNIIAYEYLPAFLGEELPEYTGYRPDVHPGV
SHVFQSAAFRFGHTMIPPGMYRRDGKCNYKRTAAGHQAIRLCATWWDSNDV
>Locus_1834_Transcript_9/13_Confidence_0.583_Length_4374|.|sp|
Q9VQH2|DUOX_DROME Dual oxidase OS=Drosophila melanogaster GN=Duox
PE=1 SV=2|e=0|from=677|to=4372|frame=2|Fragment_3
GMASQIAEREDSLLCSDVRDKLFGPMEFTRRDLGALNIMRGRDNGLPDYNTARSYFRLPK
VNDWTEINPELFAARPELLGLLVAAYNNRLDNVDVYVGGMLESDGKPGELFTAVIREQFV
RIRDADRFWFENE
>Locus_1834_Transcript_9/13_Confidence_0.583_Length_4374|.|sp|
Q9VQH2|DUOX_DROME Dual oxidase OS=Drosophila melanogaster GN=Duox
PE=1 SV=2|e=0|from=677|to=4372|frame=2|Fragment_4
NGIFTKEEIEDIKKITLWDIIVNSTSIEPHEIQKDVFRFAEGDPCPQPAQLNASLLEPCK
YLRGYDYFEGSELAYIYVCVLLGFVPILCAAAGYGV
>Locus_1834_Transcript_9/13_Confidence_0.583_Length_4374|.|sp|
Q9VQH2|DUOX_DROME Dual oxidase OS=Drosophila melanogaster GN=Duox
PE=1 SV=2|e=0|from=677|to=4372|frame=2|Fragment_5
EALKSKAEGKGGSVDKMIVREWLHANHKRLVKVRFGPEAALHTVDRKGEKLRTVSFKNVD
SVTIEESQDNYHNKKPLLLMRIPRDHDLVLELDSLGSRRKFVSKLESFLLSHKKHLICIQ
QMRDMILAKAETRERRQKKLEHFFREAYALTFGLRP
>Locus_1834_Transcript_9/13_Confidence_0.583_Length_4374|.|sp|
Q9VQH2|DUOX_DROME Dual oxidase OS=Drosophila melanogaster GN=Duox
PE=1 SV=2|e=0|from=677|to=4372|frame=2|Fragment_6
VVTVMRTSLSKGEFASALGMKSDAVFVRKMFNIVDKDGDGRISFQEFLDTVVLFSRGKTE
DKLRIIFDMCDNDRNGVIDKGELSEMLRSLVEIARTTSLSDDQVTELIDGMFQDAGLEHK
DSLTYVDFKQMMKEYKGDFVAIGLDCKGAKQNFLDTSTNVARMTSFHIEPAMEKRRPYLL

RQWDCLTTLLEENRQNIFYLFIFYVITIALFVERFIHYSFMAEHTDLRHIMGVGIAITRG
SAASLSFCYSLLLLTMSRNLITKLKQFAVQQYIPLDSHVQFHKIAACTALFFSLLHTVGH
IVNFYHVSTQPVEHLKCMTREVHFTSDYKPDISFWMFQTITGVTGVMLFITMCIIFVFAH
PTIRKKAYSYFWRAHSLYVLLYALCLVHGLARLTGAPRFWLFFIGPGIIYALDKIVTLRT
KYMALDVIETELLPSDVI
>Locus_1834_Transcript_10/13_Confidence_0.583_Length_4374|.|sp|
Q9VQH2|DUOX_DROME Dual oxidase OS=Drosophila melanogaster GN=Duox
PE=1 SV=2|e=0|from=677|to=4372|frame=2|Fragment_1
LFVGLLTYCSCLGS
>Locus_1834_Transcript_10/13_Confidence_0.583_Length_4374|.|sp|
Q9VQH2|DUOX_DROME Dual oxidase OS=Drosophila melanogaster GN=Duox
PE=1 SV=2|e=0|from=677|to=4372|frame=2|Fragment_2
EKILSNTEKQRYDGWYNNLAHPDWGSVGSHLTRKTPPAYADGVYALAGQGRPSPRRLSRL
FMKGQDGLASKMNRTALLAFFGQVVTSEIVMASESGCPIEMHRIEIDRCDEMYDPECRGD
RYIPFHRAAYDRRTGMSPNMPREQINQMTSWIDGSFIYSSSEPWLNAMRSFHNGTLLGEE
TSHGTMPIRNTMRVPLFNNPVPHVMRMLSPERLFLLGDPRSNQNPALLAFGILFFRWHNV
IAGRVQRQHPTWPDEEVFQRARRIVVASLQNIIAYEYLPAFLGEELPEYTGYRPDVHPGV
SHVFQSAAFRFGHTMIPPGMYRRDGKCNYKRTAAGHQAIRLCATWWDSNDV
>Locus_1834_Transcript_10/13_Confidence_0.583_Length_4374|.|sp|
Q9VQH2|DUOX_DROME Dual oxidase OS=Drosophila melanogaster GN=Duox
PE=1 SV=2|e=0|from=677|to=4372|frame=2|Fragment_3
GMASQIAEREDSLLCSDVRDKLFGPMEFTRRDLGALNIMRGRDNGLPDYNTARSYFRLPK
VNDWTEINPELFAARPELLGLLVAAYNNRLDNVDVYVGGMLESDGKPGELFTAVIREQFV
RIRDADRFWFENE
>Locus_1834_Transcript_10/13_Confidence_0.583_Length_4374|.|sp|
Q9VQH2|DUOX_DROME Dual oxidase OS=Drosophila melanogaster GN=Duox
PE=1 SV=2|e=0|from=677|to=4372|frame=2|Fragment_4
NGIFTKEEIEDIKKITLWDIIVNSTSIEPHEIQKDVFRFAEGDPCPQPAQLNASLLEPCK
YLRGYDYFEGSELAYIYVCVLLGFVPILCAAAGYGV
>Locus_1834_Transcript_10/13_Confidence_0.583_Length_4374|.|sp|
Q9VQH2|DUOX_DROME Dual oxidase OS=Drosophila melanogaster GN=Duox
PE=1 SV=2|e=0|from=677|to=4372|frame=2|Fragment_5
EALKSKAEGKGGSVDKMIVREWLHANHKRLVKVRFGPEAALHTVDRKGEKLRTVSFKNVD
SVTIEESQDNYHNKKPLLLMRIPRDHDLVLELDSLGSRRKFVSKLESFLLSHKKHLICIQ
QMRDMILAKAETRERRQKKLEHFFREAYALTFGLRP
>Locus_1834_Transcript_10/13_Confidence_0.583_Length_4374|.|sp|
Q9VQH2|DUOX_DROME Dual oxidase OS=Drosophila melanogaster GN=Duox
PE=1 SV=2|e=0|from=677|to=4372|frame=2|Fragment_6
VVTVMRTSLSKGEFASALGMKSDAVFVRKMFNIVDKDGDGRISFQEFLDTVVLFSRGKTE
DKLRIIFDMCDNDRNGVIDKGELSEMLRSLVEIARTTSLSDDQVTELIDGMFQDAGLEHK
DSLTYVDFKQMMKEYKGDFVAIGLDCKGAKQNFLDTSTNVARMTSFHIEPAMEKRRPYLL
RQWDCLTTLLEENRQNIFYLFIFYVITIALFVERFIHYSFMAEHTDLRHIMGVGIAITRG
SAASLSFCYSLLLLTMSRNLITKLKQFAVQQYIPLDSHVQFHKIAACTALFFSLLHTVGH
IVNFYHVSTQPVEHLKCMTREVHFTSDYKPDISFWMFQTITGVTGVMLFITMCIIFVFAH
PTIRKKAYSYFWRAHSLYVLLYALCLVHGLARLTGAPRFWLFFIGPGIIYALDKIVTLRT
KYMALDVIETELLPSDVI
>Locus_1834_Transcript_11/13_Confidence_0.556_Length_4374|.|sp|
Q9VQH2|DUOX_DROME Dual oxidase OS=Drosophila melanogaster GN=Duox
PE=1 SV=2|e=0|from=677|to=4372|frame=2|Fragment_1
LFVGLLTYCSCLGS
>Locus_1834_Transcript_11/13_Confidence_0.556_Length_4374|.|sp|
Q9VQH2|DUOX_DROME Dual oxidase OS=Drosophila melanogaster GN=Duox
PE=1 SV=2|e=0|from=677|to=4372|frame=2|Fragment_2
EKILSNTEKQRYDGWYNNLAHPDWGSVGSHLTRKTPPAYADGVYALAGQGRPSPRRLSRL

FMKGQDGLASKMNRTALLAFFGQVVTSEIVMASESGCPIEMHRIEIDRCDEMYDPECRGD
RYIPFHRAAYDRRTGMSPNMPREQINQMTSWIDGSFIYSSSEPWLNAMRSFHNGTLLGEE
TSHGTMPIRNTMRVPLFNNPVPHVMRMLSPERLFLLGDPRSNQNPALLAFGILFFRWHNV
IAGRVQRQHPTWPDEEVFQRARRIVVASLQNIIAYEYLPAFLGEELPEYTGYRPDVHPGV
SHVFQSAAFRFGHTMIPPGMYRRDGKCNYKRTAAGHQAIRLCATWWDSNDV
>Locus_1834_Transcript_11/13_Confidence_0.556_Length_4374|.|sp|
Q9VQH2|DUOX_DROME Dual oxidase OS=Drosophila melanogaster GN=Duox
PE=1 SV=2|e=0|from=677|to=4372|frame=2|Fragment_3
GMASQIAEREDSLLCSDVRDKLFGPMEFTRRDLGALNIMRGRDNGLPDYNTARSYFRLPK
VNDWTEINPELFAARPELLGLLVAAYNNRLDNVDVYVGGMLESDGKPGELFTAVIREQFV
RIRDADRFWFENE
>Locus_1834_Transcript_11/13_Confidence_0.556_Length_4374|.|sp|
Q9VQH2|DUOX_DROME Dual oxidase OS=Drosophila melanogaster GN=Duox
PE=1 SV=2|e=0|from=677|to=4372|frame=2|Fragment_4
NGIFTKEEIEDIKKITLWDIIVNSTSIEPHEIQKDVFRFAEGDPCPQPAQLNASLLEPCK
YLRGYDYFEGSELAYIYVCVLLGFVPILCAAAGYGV
>Locus_1834_Transcript_11/13_Confidence_0.556_Length_4374|.|sp|
Q9VQH2|DUOX_DROME Dual oxidase OS=Drosophila melanogaster GN=Duox
PE=1 SV=2|e=0|from=677|to=4372|frame=2|Fragment_5
EALKSKAEGKGGSVDKMIVREWLHANHKRLVKVRFGPEAALHTVDRKGEKLRTVSFKNVD
SVTIEESQDNYHNKKPLLLMRIPRDHDLVLELDSLGSRRKFVSKLESFLLSHKKHLICIQ
QMRDMILAKAETRERRQKKLEHFFREAYALTFGLRP
>Locus_1834_Transcript_11/13_Confidence_0.556_Length_4374|.|sp|
Q9VQH2|DUOX_DROME Dual oxidase OS=Drosophila melanogaster GN=Duox
PE=1 SV=2|e=0|from=677|to=4372|frame=2|Fragment_6
VVTVMRTSLSKGEFASALGMKSDAVFVRKMFNIVDKDGDGRISFQEFLDTVVLFSRGKTE
DKLRIIFDMCDNDRNGVIDKGELSEMLRSLVEIARTTSLSDDQVTELIDGMFQDAGLEHK
DSLTYVDFKQMMKEYKGDFVAIGLDCKGAKQNFLDTSTNVARMTSFHIEPAMEKRRPYLL
RQWDCLTTLLEENRQNIFYLFIFYVITIALFVERFIHYSFMAEHTDLRHIMGVGIAITRG
SAASLSFCYSLLLLTMSRNLITKLKQFAVQQYIPLDSHVQFHKIAACTALFFSLLHTVGH
IVNFYHVSTQPVEHLKCMTREVHFTSDYKPDISFWMFQTITGVTGVMLFITMCIIFVFAH
PTIRKKAYSYFWRAHSLYVLLYALCLVHGLARLTGAPRFWLFFIGPGIIYALDKIVTLRT
KYMALDVIETELLPSDVI
>Locus_1834_Transcript_13/13_Confidence_0.583_Length_4374|.|sp|
Q9VQH2|DUOX_DROME Dual oxidase OS=Drosophila melanogaster GN=Duox
PE=1 SV=2|e=0|from=677|to=4372|frame=2|Fragment_1
LFVGLLTYCSCLGS
>Locus_1834_Transcript_13/13_Confidence_0.583_Length_4374|.|sp|
Q9VQH2|DUOX_DROME Dual oxidase OS=Drosophila melanogaster GN=Duox
PE=1 SV=2|e=0|from=677|to=4372|frame=2|Fragment_2
EKILSNTEKQRYDGWYNNLAHPDWGSVGSHLTRKTPPAYADGVYALAGQGRPSPRRLSRL
FMKGQDGLASKMNRTALLAFFGQVVTSEIVMASESGCPIEMHRIEIDRCDEMYDPECRGD
RYIPFHRAAYDRRTGMSPNMPREQINQMTSWIDGSFIYSSSEPWLNAMRSFHNGTLLGEE
TSHGTMPIRNTMRVPLFNNPVPHVMRMLSPERLFLLGDPRSNQNPALLAFGILFFRWHNV
IAGRVQRQHPTWPDEEVFQRARRIVVASLQNIIAYEYLPAFLGEELPEYTGYRPDVHPGV
SHVFQSAAFRFGHTMIPPGMYRRDGKCNYKRTAAGHQAIRLCATWWDSNDV
>Locus_1834_Transcript_13/13_Confidence_0.583_Length_4374|.|sp|
Q9VQH2|DUOX_DROME Dual oxidase OS=Drosophila melanogaster GN=Duox
PE=1 SV=2|e=0|from=677|to=4372|frame=2|Fragment_3
GMASQIAEREDSLLCSDVRDKLFGPMEFTRRDLGALNIMRGRDNGLPDYNTARSYFRLPK
VNDWTEINPELFAARPELLGLLVAAYNNRLDNVDVYVGGMLESDGKPGELFTAVIREQFV
RIRDADRFWFENE
>Locus_1834_Transcript_13/13_Confidence_0.583_Length_4374|.|sp|
Q9VQH2|DUOX_DROME Dual oxidase OS=Drosophila melanogaster GN=Duox

PE=1 SV=2|e=0|from=677|to=4372|frame=2|Fragment_4
NGIFTKEEIEDIKKITLWDIIVNSTSIEPHEIQKDVFRFAEGDPCPQPAQLNASLLEPCK
YLRGYDYFEGSELAYIYVCVLLGFVPILCAAAGYGV
>Locus_1834_Transcript_13/13_Confidence_0.583_Length_4374|.|sp|
Q9VQH2|DUOX_DROME Dual oxidase OS=Drosophila melanogaster GN=Duox
PE=1 SV=2|e=0|from=677|to=4372|frame=2|Fragment_5
EALKSKAEGKGGSVDKMIVREWLHANHKRLVKVRFGPEAALHTVDRKGEKLRTVSFKNVD
SVTIEESQDNYHNKKPLLLMRIPRDHDLVLELDSLGSRRKFVSKLESFLLSHKKHLICIQ
QMRDMILAKAETRERRQKKLEHFFREAYALTFGLRP
>Locus_1834_Transcript_13/13_Confidence_0.583_Length_4374|.|sp|
Q9VQH2|DUOX_DROME Dual oxidase OS=Drosophila melanogaster GN=Duox
PE=1 SV=2|e=0|from=677|to=4372|frame=2|Fragment_6
VVTVMRTSLSKGEFASALGMKSDAVFVRKMFNIVDKDGDGRISFQEFLDTVVLFSRGKTE
DKLRIIFDMCDNDRNGVIDKGELSEMLRSLVEIARTTSLSDDQVTELIDGMFQDAGLEHK
DSLTYVDFKQMMKEYKGDFVAIGLDCKGAKQNFLDTSTNVARMTSFHIEPAMEKRRPYLL
RQWDCLTTLLEENRQNIFYLFIFYVITIALFVERFIHYSFMAEHTDLRHIMGVGIAITRG
SAASLSFCYSLLLLTMSRNLITKLKQFAVQQYIPLDSHVQFHKIAACTALFFSLLHTVGH
IVNFYHVSTQPVEHLKCMTREVHFTSDYKPDISFWMFQTITGVTGVMLFITMCIIFVFAH
PTIRKKAYSYFWRAHSLYVLLYALCLVHGLARLTGAPRFWLFFIGPGIIYALDKIVTLRT
KYMALDVIETELLPSDVI
>Locus_1834_Transcript_12/13_Confidence_0.278_Length_1991|.|sp|
Q9VQH2|DUOX_DROME Dual oxidase OS=Drosophila melanogaster GN=Duox
PE=1 SV=2|e=0|from=1|to=1989|frame=1|Fragment_1
YLRGYDYFEGSELAYIYVCVLLGFVPILCAAAGYGV
>Locus_1834_Transcript_12/13_Confidence_0.278_Length_1991|.|sp|
Q9VQH2|DUOX_DROME Dual oxidase OS=Drosophila melanogaster GN=Duox
PE=1 SV=2|e=0|from=1|to=1989|frame=1|Fragment_2
EALKSKAEGKGGSVDKMIVREWLHANHKRLVKVRFGPEAALHTVDRKGEKLRTVSFKNVD
SVTIEESQDNYHNKKPLLLMRIPRDHDLVLELDSLGSRRKFVSKLESFLLSHKKHLICIQ
QMRDMILAKAETRERRQKKLEHFFREAYALTFGLRP
>Locus_1834_Transcript_12/13_Confidence_0.278_Length_1991|.|sp|
Q9VQH2|DUOX_DROME Dual oxidase OS=Drosophila melanogaster GN=Duox
PE=1 SV=2|e=0|from=1|to=1989|frame=1|Fragment_3
VVTVMRTSLSKGEFASALGMKSDAVFVRKMFNIVDKDGDGRISFQEFLDTVVLFSRGKTE
DKLRIIFDMCDNDRNGVIDKGELSEMLRSLVEIARTTSLSDDQVTELIDGMFQDAGLEHK
DSLTYVDFKQMMKEYKGDFVAIGLDCKGAKQNFLDTSTNVARMTSFHIEPAMEKRRPYLL
RQWDCLTTLLEENRQNIFYLFIFYVITIALFVERFIHYSFMAEHTDLRHIMGVGIAITRG
SAASLSFCYSLLLLTMSRNLITKLKQFAVQQYIPLDSHVQFHKIAACTALFFSLLHTVGH
IVNFYHVSTQPVEHLKCMTREVHFTSDYKPDISFWMFQTITGVTGVMLFITMCIIFVFAH
PTIRKKAYSYFWRAHSLYVLLYALCLVHGLARLTGAPRFWLFFIGPGIIYALDKIVTLRT
KYMALDVIETELLPSDVI
>Locus_1834_Transcript_5/13_Confidence_0.139_Length_1409|.|sp|
Q9VQH2|DUOX_DROME Dual oxidase OS=Drosophila melanogaster GN=Duox
PE=1 SV=2|e=1.0231E-111|from=677|to=1408|frame=2|Fragment_1
LFVGLLTYCSCLGS
>Locus_1834_Transcript_5/13_Confidence_0.139_Length_1409|.|sp|
Q9VQH2|DUOX_DROME Dual oxidase OS=Drosophila melanogaster GN=Duox
PE=1 SV=2|e=1.0231E-111|from=677|to=1408|frame=2|Fragment_2
EKILSNTEKQRYDGWYNNLAHPDWGSVGSHLTRKTPPAYADGVYALAGQGRPSPRRLSRL
FMKGQDGLASKMNRTALLAFFGQVVTSEIVMASESGCPIEMHRIEIDRCDEMYDPECRGD
RYIPFHRAAYDRRTGMSPNMPREQINQMTSWIDGSFIYSSSEPWLNAMRSFHNGTLLGEE
TSHGTMPIRNTMRVPLFNNPVPHVMRMLSPERLFLLGDPRSNQNPALLAF
>Locus_14687_Transcript_1/2_Confidence_0.625_Length_578|.|sp|Q9VQH2|
DUOX_DROME Dual oxidase OS=Drosophila melanogaster GN=Duox PE=1

SV=2|e=1.5775E-109|from=1|to=576|frame=1|Fragment_1
GIIYALDKIVTLRTKYMALDVIETELLPSDVIRIKFYRPPNLKYLSGQWVRVACTAFRSN
EFHSFTLTSAPHENFLSCHIKAQGPWTWKLRNYFDPCNFNPEDQPKLRIEGPFGGGNQDW
YKFEVAVMVGGGIGVTPYASILNDLVFGTSTNRYSGVACKKVYFLWICPSHKHFEWFIDV
LRDVEKKDVTNV
>Locus_14687_Transcript_2/2_Confidence_0.875_Length_578|.|sp|Q9VQH2|
DUOX_DROME Dual oxidase OS=Drosophila melanogaster GN=Duox PE=1
SV=2|e=1.5775E-109|from=1|to=576|frame=1|Fragment_1
GIIYALDKIVTLRTKYMALDVIETELLPSDVIRIKFYRPPNLKYLSGQWVRVACTAFRSN
EFHSFTLTSAPHENFLSCHIKAQGPWTWKLRNYFDPCNFNPEDQPKLRIEGPFGGGNQDW
YKFEVAVMVGGGIGVTPYASILNDLVFGTSTNRYSGVACKKVYFLWICPSHKHFEWFIDV
LRDVEKKDVTNV
>Locus_1834_Transcript_4/13_Confidence_0.056_Length_511|.|sp|Q9VQH2|
DUOX_DROME Dual oxidase OS=Drosophila melanogaster GN=Duox PE=1
SV=2|e=1.55772E-80|from=1|to=510|frame=1|Fragment_1
FMKGQDGLASKMNRTALLAFFGQVVTSEIVMASESGCPIEMHRIEIDRCDEMYDPECRGD
RYIPFHRAAYDRRTGMSPNMPREQINQMTSWIDGSFIYSSSEPWLNAMRSFHNGTLLGEE
TSHGTMPIRNTMRVPLFNNPVPHVMRMLSPERLFLLGDPRSNQNPALLAF
>Locus_21100_Transcript_1/1_Confidence_1.000_Length_383|.|sp|Q9VQH2|
DUOX_DROME Dual oxidase OS=Drosophila melanogaster GN=Duox PE=1
SV=2|e=3.107E-74|from=3|to=380|frame=-1|Fragment_1
NEFHSFTLTSAPHENFLSCHIKAQGPWTWKLRNYFDPCNFNPEDQPKLRIEGPFGGGNQD
WYKFEVAVMVGGGIGVTPYASILNDLVFGTSTNRYSGVACKKVYFLWICPSHKHFEWFID
VLRDVE
>Locus_11826_Transcript_1/1_Confidence_1.000_Length_368|.|sp|Q9VQH2|
DUOX_DROME Dual oxidase OS=Drosophila melanogaster GN=Duox PE=1
SV=2|e=1.32623E-72|from=3|to=368|frame=-1|Fragment_1
SFTLTSAPHENFLSCHIKAQGPWTWKLRNYFDPCNFNPEDQPKLRIEGPFGGGNQDWYKF
EVAVMVGGGIGVTPYASILNDLVFGTSTNRYSGVACKKVYFLWICPSHKHFEWFIDVLRD
VE
>Locus_27287_Transcript_1/1_Confidence_1.000_Length_368|.|sp|Q9VQH2|
DUOX_DROME Dual oxidase OS=Drosophila melanogaster GN=Duox PE=1
SV=2|e=2.68469E-57|from=1|to=366|frame=1|Fragment_1
CMTREVHFTSDYKPDISFWMFQTITGVTGVMLFITMCIIFVFAHPTIRKKAYSYFWRAHS
LYVLLYALCLIHGLARLTGAPRFWLFFIGPGIIYALDKIVTLRTKYMALDVIETELLPSD
VI
>Locus_21056_Transcript_1/1_Confidence_1.000_Length_344|.|sp|Q9VQH2|
DUOX_DROME Dual oxidase OS=Drosophila melanogaster GN=Duox PE=1
SV=2|e=4.39151E-52|from=3|to=341|frame=-1|Fragment_1
SDYKPDISFWMFQTITGVTGVMLFITMCIIFVFAHPTIRKKAYSYFWRAHSLYVLLYALC
LVHGLARLTGAPRFWLFFIGPGIIYALDKIVTLRTKYMALDVIETELLPSDVI
>Locus_17924_Transcript_1/1_Confidence_1.000_Length_248|.|sp|Q9VQH2|
DUOX_DROME Dual oxidase OS=Drosophila melanogaster GN=Duox PE=1
SV=2|e=4.49798E-44|from=1|to=246|frame=1|Fragment_1
KKVYFLWICPSHKHFEWFIDVLRDVEKKDVTNVLEIHIFITQFFHKFDLRTTMLYICENH
FQRLSKMSMFTGLKAVNHFGRP
>Locus_20864_Transcript_1/1_Confidence_1.000_Length_248|.|sp|Q9VQH2|
DUOX_DROME Dual oxidase OS=Drosophila melanogaster GN=Duox PE=1
SV=2|e=4.49798E-44|from=1|to=246|frame=1|Fragment_1
KKVYFLWICPSHKHFEWFIDVLRDVEKKDVTNVLEIHIFITQFFHKFDLRTTMLYICENH
FQRLSKMSMFTGLKAVNHFGRP
>Locus_1834_Transcript_3/13_Confidence_1.000_Length_233|.|sp|Q9VQH2|
DUOX_DROME Dual oxidase OS=Drosophila melanogaster GN=Duox PE=1
SV=2|e=1.05263E-21|from=31|to=231|frame=1|Fragment_1

LFVGLLTYCSCLGS
>Locus_1834_Transcript_3/13_Confidence_1.000_Length_233|.|sp|Q9VQH2|DUOX_DROME Dual oxidase OS=Drosophila melanogaster GN=Duox PE=1 SV=2|e=1.05263E-21|from=31|to=231|frame=1|Fragment_2
EKILSNTEKQRYDGWYNNLAHPDWGSVGSHLTRKTPPAYADGVYALAGQGRPS
>Locus_873_Transcript_1/2_Confidence_0.667_Length_1168|UNIGENE REPRESENTATIVE TRANSCRIPT|sp|Q9VRG7|CNEP1_DROME CTD nuclear envelope phosphatase 1 homolog OS=Drosophila melanogaster GN=l(1)G0269 PE=2 SV=1|e=9.7646E-107|from=152|to=796|frame=2|Fragment_1
KNILKWVQHQPVKYQLYPLSPISRHRLSIVKRKTLVLDLDETLIHSHHDAMQRSSVKPGT
PPDFILKVTIDKHPVRFFVHKRPHVDFFLDIVSQWYDLVVFTASMEIYGAAVADKLDNGR
GILRRRYYRQHCTPDLGSYTKDLSAICADLNRVFILDNSPGAYRGYPDNAIPIKSWFSDP
MDTA
>Locus_873_Transcript_1/2_Confidence_0.667_Length_1168|UNIGENE REPRESENTATIVE TRANSCRIPT|sp|Q9VRG7|CNEP1_DROME CTD nuclear envelope phosphatase 1 homolog OS=Drosophila melanogaster GN=l(1)G0269 PE=2 SV=1|e=9.7646E-107|from=152|to=796|frame=2|Fragment_2
RFTQDVRSVLSRNLHLHRLW
>Locus_873_Transcript_1/2_Confidence_0.667_Length_1168|.|sp|Q9VRG7|CNEP1_DROME CTD nuclear envelope phosphatase 1 homolog OS=Drosophila melanogaster GN=l(1)G0269 PE=2 SV=1|e=9.7646E-107|from=12|to=92|frame=3|Fragment_1
AFYVVASKVWTCLCFMFKKQVRAGLTY
>Locus_873_Transcript_2/2_Confidence_0.667_Length_1132|.|sp|Q9VRG7|CNEP1_DROME CTD nuclear envelope phosphatase 1 homolog OS=Drosophila melanogaster GN=l(1)G0269 PE=2 SV=1|e=6.98018E-97|from=2|to=760|frame=2|Fragment_1
QVRAWVQHQPVKYQLYPLSPISRHRLNFLPRKTLVLDLDETLVHAYVVKDHSTKSKSKIL
RKKSPGIVKRKTLVLDLDETLIHSHHDAMQRSSVKPGTPPDFILKVTIDKHPVRFFVHKR
PHVDFFLDIVSQWYDLVVFTASMEIYGAAVADKLDNGRGILRRRYYRQHCTPDLGSYTKD
LSAICADLNRVFILDNSPGAYRGYPDNAIPIKSWFSDPMDTA
>Locus_873_Transcript_2/2_Confidence_0.667_Length_1132|.|sp|Q9VRG7|CNEP1_DROME CTD nuclear envelope phosphatase 1 homolog OS=Drosophila melanogaster GN=l(1)G0269 PE=2 SV=1|e=6.98018E-97|from=2|to=760|frame=2|Fragment_2
RFTQDVRSVLSRNLHLHRLW
>Locus_4883_Transcript_4/5_Confidence_0.692_Length_1710|UNIGENE REPRESENTATIVE TRANSCRIPT|sp|Q9VVT2|INDY1_DROME Protein I'm not dead yet OS=Drosophila melanogaster GN=Indy PE=1 SV=2|e=3.4176E-128|from=377|to=1705|frame=2|Fragment_1
RPQHRGCCSKMLYFFKVYWRSFVIVLAPILLTPVFLVNNEPKFRCMYVVMLMSAYWVTEV
LPLPVTAMLPMVLFPTMGILGTDRTCMMYMRETNVMFIGGLIIALAVEHCNLHKRLALKV
ISIIGCSHRRLNFGLVVVTMFVSMWISNTAAVAMMCPIMQAVLEELETQGLCEMYIKKKS
TPESEERMLQSNEAT
>Locus_4883_Transcript_4/5_Confidence_0.692_Length_1710|UNIGENE REPRESENTATIVE TRANSCRIPT|sp|Q9VVT2|INDY1_DROME Protein I'm not dead yet OS=Drosophila melanogaster GN=Indy PE=1 SV=2|e=3.4176E-128|from=377|to=1705|frame=2|Fragment_2
DEPKRPSKTTLCYFMSAAYASTLGGVGSIVGSGTNLTFKGIYESRFPDA
>Locus_4883_Transcript_4/5_Confidence_0.692_Length_1710|UNIGENE REPRESENTATIVE TRANSCRIPT|sp|Q9VVT2|INDY1_DROME Protein I'm not dead yet OS=Drosophila melanogaster GN=Indy PE=1 SV=2|e=3.4176E-128|from=377|to=1705|frame=2|Fragment_3
PGIDFPKWMFYNVPGMLIFTFLTWVYLQWLYMGMFRPNSVEAKQATLSKEGERVARSVIE

TKYKELGPMSVHEKQVG
>Locus_4883_Transcript_4/5_Confidence_0.692_Length_1710|UNIGENE REPRESENTATIVE TRANSCRIPT|sp|Q9VVT2|INDY1_DROME Protein I'm not dead yet OS=Drosophila melanogaster GN=Indy PE=1 SV=2|e=3.4176E-128|from=377|to=1705|frame=2|Fragment_4
RAPGFITGWAEAITDTKVKDATPAIFVVIILFMLPQNLDFLKFCRSKKGEKGEIEPLPTK
PAGALITWKYVLAKIPW
>Locus_4883_Transcript_4/5_Confidence_0.692_Length_1710|UNIGENE REPRESENTATIVE TRANSCRIPT|sp|Q9VVT2|INDY1_DROME Protein I'm not dead yet OS=Drosophila melanogaster GN=Indy PE=1 SV=2|e=3.4176E-128|from=377|to=1705|frame=2|Fragment_5
EGSKESGMSEYLGQCLIGLK
>Locus_5414_Transcript_1/3_Confidence_0.571_Length_658|.|sp|Q9VVT2|INDY1_DROME Protein I'm not dead yet OS=Drosophila melanogaster GN=Indy PE=1 SV=2|e=7.56413E-40|from=37|to=459|frame=1|Fragment_1
EGSKESGMSEYLGQ
>Locus_5414_Transcript_1/3_Confidence_0.571_Length_658|.|sp|Q9VVT2|INDY1_DROME Protein I'm not dead yet OS=Drosophila melanogaster GN=Indy PE=1 SV=2|e=7.56413E-40|from=37|to=459|frame=1|Fragment_2
TAEFLTEFSSNVAIANIMLPVLAEMSVAIEMHPLYLMLPAALCCSMAFHMPVGTPPNAIA
AGMCNISIGDMAKAGIGPSIVTLLVIWASFPTWGSVVYPELVTFPTWA
>Locus_5414_Transcript_2/3_Confidence_0.571_Length_658|.|sp|Q9VVT2|INDY1_DROME Protein I'm not dead yet OS=Drosophila melanogaster GN=Indy PE=1 SV=2|e=7.56413E-40|from=37|to=459|frame=1|Fragment_1
EGSKESGMSEYLGQ
>Locus_5414_Transcript_2/3_Confidence_0.571_Length_658|.|sp|Q9VVT2|INDY1_DROME Protein I'm not dead yet OS=Drosophila melanogaster GN=Indy PE=1 SV=2|e=7.56413E-40|from=37|to=459|frame=1|Fragment_2
TAEFLTEFSSNVAIANIMLPVLAEMSVAIEMHPLYLMLPAALCCSMAFHMPVGTPPNAIA
AGMCNISIGDMAKAGIGPSIVTLLVIWASFPTWGSVVYPELVTFPTWA
>Locus_5414_Transcript_3/3_Confidence_0.714_Length_658|.|sp|Q9VVT2|INDY1_DROME Protein I'm not dead yet OS=Drosophila melanogaster GN=Indy PE=1 SV=2|e=7.56413E-40|from=37|to=459|frame=1|Fragment_1
EGSKESGMSEYLGQ
>Locus_5414_Transcript_3/3_Confidence_0.714_Length_658|.|sp|Q9VVT2|INDY1_DROME Protein I'm not dead yet OS=Drosophila melanogaster GN=Indy PE=1 SV=2|e=7.56413E-40|from=37|to=459|frame=1|Fragment_2
TAEFLTEFSSNVAIANIMLPVLAEMSVAIEMHPLYLMLPAALCCSMAFHMPVGTPPNAIA
AGMCNISIGDMAKAGIGPSIVTLLVIWASFPTWGSVVYPELVTFPTWA
>Locus_7301_Transcript_1/2_Confidence_0.800_Length_374|.|sp|Q9VVT2|INDY1_DROME Protein I'm not dead yet OS=Drosophila melanogaster GN=Indy PE=1 SV=2|e=2.35889E-21|from=6|to=335|frame=-1|Fragment_1
RAPGFITGWAEAITDTKVKDATPAIFVVIILFMLPQNLDFLKFCRSKKGEKGEIEPLPTK
PAGALITWKYVLAKIPW
>Locus_7301_Transcript_1/2_Confidence_0.800_Length_374|.|sp|Q9VVT2|INDY1_DROME Protein I'm not dead yet OS=Drosophila melanogaster GN=Indy PE=1 SV=2|e=2.35889E-21|from=6|to=335|frame=-1|Fragment_2
AEGSKESGMSEYLGQCLIGLK
>Locus_7301_Transcript_2/2_Confidence_0.800_Length_374|.|sp|Q9VVT2|INDY1_DROME Protein I'm not dead yet OS=Drosophila melanogaster GN=Indy PE=1 SV=2|e=2.35889E-21|from=6|to=335|frame=-1|Fragment_1
RAPGFITGWAEAITDTKVKDATPAIFVVIILFMLPQNLDFLKFCRSKKGEKGEIEPLPTK
PAGALITWKYVLAKIPW
>Locus_7301_Transcript_2/2_Confidence_0.800_Length_374|.|sp|Q9VVT2|

INDY1_DROME Protein I'm not dead yet OS=Drosophila melanogaster
GN=Indy PE=1 SV=2|e=2.35889E-21|from=6|to=335|frame=-1|Fragment_2
AEGSKESGMSEYLGQCLIGLK
>Locus_11446_Transcript_1/1_Confidence_1.000_Length_380|UNIGENE
REPRESENTATIVE TRANSCRIPT|sp|Q9VW12|U389_DROME UPF0389 protein
CG9231 OS=Drosophila melanogaster GN=CG9231 PE=1 SV=1|e=1.47157E-15|
from=25|to=279|frame=1|Fragment_1
IKGATPSNFDKKLLVYFKRYSSVEEIPKLVPHDTLHKVRGKARMYFATAFMVVTLIGCYG
MVKSGKHAAERGEFVQRQNIEWHKQ
>Locus_14791_Transcript_1/2_Confidence_0.667_Length_592|UNIGENE
REPRESENTATIVE TRANSCRIPT|sp|Q9VW60|ADCY2_DROME Adenylate cyclase
type 2 OS=Drosophila melanogaster GN=Ac76E PE=2 SV=3|e=2.30745E-26|
from=80|to=448|frame=2|Fragment_1
SPSGLAVRSRPSSKMTKYVECWGADKPFANITDSSMAKNIGLASIAMIESNLEPSRM
>Locus_14791_Transcript_1/2_Confidence_0.667_Length_592|UNIGENE
REPRESENTATIVE TRANSCRIPT|sp|Q9VW60|ADCY2_DROME Adenylate cyclase
type 2 OS=Drosophila melanogaster GN=Ac76E PE=2 SV=3|e=2.30745E-26|
from=80|to=448|frame=2|Fragment_2
APRELKPCTLRYRVDERESMYATRRDDLFRRDVACAYGLFILMAAVLLL
>Locus_14791_Transcript_2/2_Confidence_0.667_Length_574|.|sp|Q9VW60|
ADCY2_DROME Adenylate cyclase type 2 OS=Drosophila melanogaster
GN=Ac76E PE=2 SV=3|e=4.34613E-27|from=62|to=430|frame=2|Fragment_1
SYTGRLLRSRPSSKMTKYVECWGADKPFANITDSSMAKNIGLASIAMIESNLEPSRM
>Locus_14791_Transcript_2/2_Confidence_0.667_Length_574|.|sp|Q9VW60|
ADCY2_DROME Adenylate cyclase type 2 OS=Drosophila melanogaster
GN=Ac76E PE=2 SV=3|e=4.34613E-27|from=62|to=430|frame=2|Fragment_2
APRELKPCTLRYRVDERESMYATRRDDLFRRDVACAYGLFILMAAVLLL
>Locus_16734_Transcript_1/1_Confidence_1.000_Length_505|.|sp|Q9VW60|
ADCY2_DROME Adenylate cyclase type 2 OS=Drosophila melanogaster
GN=Ac76E PE=2 SV=3|e=2.62602E-16|from=79|to=504|frame=1|Fragment_1
GPADIVAASRFLRLAVFGLMTSLIACCAAIGLINFE
>Locus_16734_Transcript_1/1_Confidence_1.000_Length_505|.|sp|Q9VW60|
ADCY2_DROME Adenylate cyclase type 2 OS=Drosophila melanogaster
GN=Ac76E PE=2 SV=3|e=2.62602E-16|from=79|to=504|frame=1|Fragment_2
DNTIELVNNLNTTDIENNEWILTEMENFKNV
>Locus_16734_Transcript_1/1_Confidence_1.000_Length_505|.|sp|Q9VW60|
ADCY2_DROME Adenylate cyclase type 2 OS=Drosophila melanogaster
GN=Ac76E PE=2 SV=3|e=2.62602E-16|from=79|to=504|frame=1|Fragment_3
PTYLHACAISLTTVSVFLRSGFLLKLCVMIIIGLCQLAVIATSEFAQNYDGFYDTR
>Locus_16734_Transcript_1/1_Confidence_1.000_Length_505|.|sp|Q9VW60|
ADCY2_DROME Adenylate cyclase type 2 OS=Drosophila melanogaster
GN=Ac76E PE=2 SV=3|e=2.62602E-16|from=79|to=504|frame=1|Fragment_4
VWLWALVMFIVLISILHTL
>Locus_2421_Transcript_1/1_Confidence_1.000_Length_2356|UNIGENE
REPRESENTATIVE TRANSCRIPT|sp|Q9VXT2|DJC25_DROME DnaJ homolog
subfamily C member 25 homolog OS=Drosophila melanogaster GN=CG7872
PE=2 SV=1|e=2.33342E-85|from=1521|to=2354|frame=-3|Fragment_1
QLARKFHPDLQKGEDKKAEATEKFKAIATAYEILKDEEARSDYDYMLDNPSE
>Locus_2421_Transcript_1/1_Confidence_1.000_Length_2356|UNIGENE
REPRESENTATIVE TRANSCRIPT|sp|Q9VXT2|DJC25_DROME DnaJ homolog
subfamily C member 25 homolog OS=Drosophila melanogaster GN=CG7872
PE=2 SV=1|e=2.33342E-85|from=1521|to=2354|frame=-3|Fragment_2
RVAPKVDIRIVLAVTITIISVIQYYGAWQRYETAISYFMTVPKYRHKALELAK
>Locus_2421_Transcript_1/1_Confidence_1.000_Length_2356|UNIGENE

REPRESENTATIVE TRANSCRIPT|sp|Q9VXT2|DJC25_DROME DnaJ homolog subfamily C member 25 homolog OS=Drosophila melanogaster GN=CG7872 PE=2 SV=1|e=2.33342E-85|from=1521|to=2354|frame=-3|Fragment_3
VNNDAAG
>Locus_2421_Transcript_1/1_Confidence_1.000_Length_2356|UNIGENE REPRESENTATIVE TRANSCRIPT|sp|Q9VXT2|DJC25_DROME DnaJ homolog subfamily C member 25 homolog OS=Drosophila melanogaster GN=CG7872 PE=2 SV=1|e=2.33342E-85|from=1521|to=2354|frame=-3|Fragment_4
LIRMVIEDNMDIKGAYAKPDLWNILWVQLIISPYTITKYIIWYTSWIWKFDILKRPYGTE
EKLYLIRKNLKLGEHQFNAI
>Locus_2421_Transcript_1/1_Confidence_1.000_Length_2356|UNIGENE REPRESENTATIVE TRANSCRIPT|sp|Q9VXT2|DJC25_DROME DnaJ homolog subfamily C member 25 homolog OS=Drosophila melanogaster GN=CG7872 PE=2 SV=1|e=2.33342E-85|from=1521|to=2354|frame=-3|Fragment_5
ELWIKDKFIAWKKIQEEEMKKNLAENARAKAYRRYMKNHGVSRMTFDD
>Locus_9997_Transcript_1/1_Confidence_1.000_Length_1151|UNIGENE REPRESENTATIVE TRANSCRIPT|sp|Q9VYB7|TPST_DROME Protein-tyrosine sulfotransferase OS=Drosophila melanogaster GN=Tango13 PE=2 SV=2|e=9.8237E-147|from=69|to=989|frame=3|Fragment_1
REMPLIFIGGVPRSGTTLMRAMLDAHPDVRCGQETRVIPRILQMRSHWLKSAKESVRLEE
AGITKEVMDAAIAAFCLEVIVKHGEPAKRLCNKDPLTLKMGTYVLDLFPNAKFLFMVRDG
RATVHSIISRKVTITGFDLSSYRQCLQKWNKAIETMHGQCREAGKDRCMMVPYEQLVLHP
KLWMEKILQFLDVPWSDSVLHHEEFINKPNGVSLSKVERSSDQVIKPVNLEALTKWVGNI
PQDVVRDMADVAPMLSVLGYDPYANPPNYGQPDSSVKYNTYRIQKEESKWNERAMEVL
>Locus_9997_Transcript_1/1_Confidence_1.000_Length_1151|UNIGENE REPRESENTATIVE TRANSCRIPT|sp|Q9VYB7|TPST_DROME Protein-tyrosine sulfotransferase OS=Drosophila melanogaster GN=Tango13 PE=2 SV=2|e=9.8237E-147|from=69|to=989|frame=3|Fragment_2
DNKQDDFPN
>Locus_8222_Transcript_1/1_Confidence_1.000_Length_1565|UNIGENE REPRESENTATIVE TRANSCRIPT|sp|Q9VYF2|IMPA3_DROME Putative inositol monophosphatase 3 OS=Drosophila melanogaster GN=CG15743 PE=2 SV=1|e=7.39149E-74|from=4|to=840|frame=1|Fragment_1
GGKEVVLISETKNLDLKSKGKTREGANDPVTNADLRSHCVMYYGLNKMFPNIKIISEEKV
SEAECPVKKPLELDSTLLHSDVHKLLDENVSTEDITVWIDPLDATQEFTESLYHYVTTMV
CVAVRGKPIVGIIHFPFTQKTYWAWIGNTASDSLLEIKNKPVTEKTENAAPTFIVSRSHA
GGVSNITKTAFGAQTKVIPAGGAGYKAIQVAQGNATVYIHTTVIKKWDICAGNAILNYLD
GDMTTLVGDKIDYSSTSNPKNEGGLLAALKDHKMYVEQL
>Locus_22975_Transcript_1/2_Confidence_0.750_Length_1185|UNIGENE REPRESENTATIVE TRANSCRIPT|sp|Q9VYN8|TENA_DROME Teneurin-a OS=Drosophila melanogaster GN=Ten-a PE=1 SV=2|e=9.9257E-134|from=20|to=1183|frame=2|Fragment_1
LRTMVLMDGSESSEATLHSNNSISLRTGRGLALDSSAWLRHPLLVRDVPILGEMMPLWSQ
QRAS
>Locus_22975_Transcript_1/2_Confidence_0.750_Length_1185|UNIGENE REPRESENTATIVE TRANSCRIPT|sp|Q9VYN8|TENA_DROME Teneurin-a OS=Drosophila melanogaster GN=Ten-a PE=1 SV=2|e=9.9257E-134|from=20|to=1183|frame=2|Fragment_2
LGSLTNFMSQRYMLSGDERNPHRTLSREIWVNNSRVFGVEFDQFTSKETFYDRDKKTLLT
TSFDPAGLPKFYQPSDGRAPLNITYDRFNRIEGWHWGPAEEHYTYDRRGFLAEVTSPQDG
TKSYTYNDWNLVSEITLASQRRFLFSYDGDGGLRHVTLPSGTKHSFSLQPSIGFLRFTYT
PPGASRCYTRHYSHSGALLQTVFPGDGARVLYRYHASGKLAELLHGDGRSEFSYSPVSGA
PSSVLHRDTELEYRWDYRYAAGLLVEERLDFSAKTGLSNAKFTYEYDSNFRVSSIQGRIG
GQTLPAHGLAWD

```
>Locus_22975_Transcript_2/2_Confidence_0.500_Length_1143|.|sp|
Q9VYN8|TENA_DROME Teneurin-a OS=Drosophila melanogaster GN=Ten-a
PE=1 SV=2|e=1.5582E-128|from=20|to=1141|frame=2|Fragment_1
LRTMVLMDGSESSEATLHSNNSISLRTGRGLALDSSAWLRHPLLVRDVPILGEMMPLWSQ
QRAS
>Locus_22975_Transcript_2/2_Confidence_0.500_Length_1143|.|sp|
Q9VYN8|TENA_DROME Teneurin-a OS=Drosophila melanogaster GN=Ten-a
PE=1 SV=2|e=1.5582E-128|from=20|to=1141|frame=2|Fragment_2
LGSLTNFMSQRYMLSGDERNPHRTLSREIWVNNSRVFGVEFDQFTSKETFYDRDKKTLLT
TSFDPAGLPKFYQPSDGRAPLNITYDRFNRIEGWHWGPAEEHYTYDRRGFLAEVTSPQDG
TKSYTYNDWNLVSEITLASQRRFLFSYDGDGGLRHVTLPSGTKHSFSLQPSIGFLRFTYT
PPGASRCYTRHYSHSGALLQTVFPGDGARVLYRYHASGKLAELLHGDGRSEFSYSPVSGA
PSSVLHRDTELEYRWDYRYAAGLLVEERLDFSAKTGLSNAKFTYEYDSNFRVSSIQGR
>Locus_17231_Transcript_1/1_Confidence_1.000_Length_545|.|sp|Q9VYN8|
TENA_DROME Teneurin-a OS=Drosophila melanogaster GN=Ten-a PE=1 SV=2|
e=3.37294E-71|from=2|to=544|frame=2|Fragment_1
ENSVQSYAHMDEYSESRVSVMRGQVVTPQGLGIVGIRVSVDRDSRFGFTLTRQGGWFDVL
VNGGGAVTLQFQRSPFRPLTRTVFVPWNQIVALPTVHMQL
>Locus_17231_Transcript_1/1_Confidence_1.000_Length_545|.|sp|Q9VYN8|
TENA_DROME Teneurin-a OS=Drosophila melanogaster GN=Ten-a PE=1 SV=2|
e=3.37294E-71|from=2|to=544|frame=2|Fragment_2
NEETPNGIPPTVPSPAFSFLSLPHY
>Locus_17231_Transcript_1/1_Confidence_1.000_Length_545|.|sp|Q9VYN8|
TENA_DROME Teneurin-a OS=Drosophila melanogaster GN=Ten-a PE=1 SV=2|
e=3.37294E-71|from=2|to=544|frame=2|Fragment_3
LPGQGQGAS
>Locus_17231_Transcript_1/1_Confidence_1.000_Length_545|.|sp|Q9VYN8|
TENA_DROME Teneurin-a OS=Drosophila melanogaster GN=Ten-a PE=1 SV=2|
e=3.37294E-71|from=2|to=544|frame=2|Fragment_4
CAAHDHELLRPRIAATWMPHAVGGKPGKSLVFAETQIVQESIPIPGS
>Locus_22876_Transcript_1/1_Confidence_1.000_Length_317|.|sp|Q9VYN8|
TENA_DROME Teneurin-a OS=Drosophila melanogaster GN=Ten-a PE=1 SV=2|
e=6.93597E-58|from=2|to=316|frame=2|Fragment_1
CVCSQGWNGRHCTLPGCENACSRHGQCTLNNGEYSCMCIEGWAGKDCSIALEMDCNDNID
NDHDGMTDCSDSECCSHPACAEHIMCLASNDPVDVLLRKQPPSVT
>Locus_29787_Transcript_1/1_Confidence_1.000_Length_274|.|sp|Q9VYN8|
TENA_DROME Teneurin-a OS=Drosophila melanogaster GN=Ten-a PE=1 SV=2|
e=1.15968E-36|from=3|to=272|frame=3|Fragment_1
TGQQRALVCNGRCDGAAIDAQLLTPVALTSGPDGSLYVGDFNLVRRITPDGKVHTVLQLS
ATQVAYQYYLVVSPADGHLYISDPERQQVL
>Locus_18041_Transcript_1/1_Confidence_1.000_Length_1778|UNIGENE
REPRESENTATIVE TRANSCRIPT|sp|Q9VZM5|PIGB_DROME GPI
mannosyltransferase 3 OS=Drosophila melanogaster GN=CG12006 PE=2
SV=2|e=2.1034E-112|from=44|to=1498|frame=-2|Fragment_1
LLILGVRVLSVFAVRTWYVPDEFWQSLEVAHYLTFGYGYLTWEWHERIRNFLYPLFISVL
YKCLDILHLDSVYMLTTVPRIVQAIFTGLAEYKLYRWSGHSKWTLFMCLSSWFVFYIGSR
TLLNTVEYGIACIAITQFPWRNCRSGDQCRFLYTIGLGTAMRPTFIIPWIPLCIYHLYNP
KLIFLKEIIVRYVVITLIILFASVGIDSLVTQRFSFTSWNFLKINIVQGVANFYGSHSFH
WYLLIGLPTILSISIIPFALAAYSMMKNKFSDHISSLMLISITWNLVIYSMITHKEFRFL
SGSLPMILVVTSKYLSFVCSLRAVPKLVLYFIVCAIMLFNLLPMYYFSWIHQSGTTLLMP
ALLKTVENYSRSNAVPPNLLFLMPCHSTPYYSHIHQNLSMRFLTCEPDI
>Locus_18041_Transcript_1/1_Confidence_1.000_Length_1778|UNIGENE
REPRESENTATIVE TRANSCRIPT|sp|Q9VZM5|PIGB_DROME GPI
mannosyltransferase 3 OS=Drosophila melanogaster GN=CG12006 PE=2
```

SV=2|e=2.1034E-112|from=44|to=1498|frame=-2|Fragment_2
KYNGIEDEADVFYQNPQKWLDINYFNVMDDECKSSSCIGRQNIKDKLPTHLIMFEELQNR
IHKSLKHYKEVLKLFH
>Locus_5299_Transcript_2/4_Confidence_0.667_Length_2193|UNIGENE
REPRESENTATIVE TRANSCRIPT|"sp|Q9W3V8|ALG8_DROME Probable dolichyl
pyrophosphate Glc1Man9GlcNAc2 alpha-1,3-glucosyltransferase
OS=Drosophila melanogaster GN=CG4542 PE=2 SV=1"|e=1.3305E-119|
from=861|to=2123|frame=3|Fragment_1
TCVKFLFIPAYRSTDFEVHRNWLAITHSKPIYLWYFENTSEWTLDYPPFFAWFEFMLSHI
AKYFDPNMLNVKNLNYASDMTVLFQRLSVMVTDLLLLFGTRECSNSLGKSNSQPKTFLA
>Locus_5299_Transcript_2/4_Confidence_0.667_Length_2193|UNIGENE
REPRESENTATIVE TRANSCRIPT|"sp|Q9W3V8|ALG8_DROME Probable dolichyl
pyrophosphate Glc1Man9GlcNAc2 alpha-1,3-glucosyltransferase
OS=Drosophila melanogaster GN=CG4542 PE=2 SV=1"|e=1.3305E-119|
from=861|to=2123|frame=3|Fragment_2
VLILCNAGLFMVDHIHFQYNGMLYGILLLSISCIMKEQYLMSALWFSILLNLKHIFAYVA
PAYVVYLLGSYCLQGNFDLSKTAKRISKLVLIGFITCTVSFGPFIRQISQVMARLFPFKR
GLCHAYWAPNFWAIYNVADKAATITAKKLGYSVSNSTATMTGGLVEDVVHTTLPTITPSI
TFYLTFITIVPALIKLWRIIRFNKTPIVISPLQFIRCLVICGACSFLFGWHVHEKAILML
IIPMSISAVLEKEDAKIFLMLSTIGHYS
>Locus_5299_Transcript_2/4_Confidence_0.667_Length_2193|UNIGENE
REPRESENTATIVE TRANSCRIPT|"sp|Q9W3V8|ALG8_DROME Probable dolichyl
pyrophosphate Glc1Man9GlcNAc2 alpha-1,3-glucosyltransferase
OS=Drosophila melanogaster GN=CG4542 PE=2 SV=1"|e=1.3305E-119|
from=861|to=2123|frame=3|Fragment_3
KALIFISYSGIAFKFLSQLFP
>Locus_5299_Transcript_4/4_Confidence_0.667_Length_2193|.|"sp|
Q9W3V8|ALG8_DROME Probable dolichyl pyrophosphate Glc1Man9GlcNAc2
alpha-1,3-glucosyltransferase OS=Drosophila melanogaster GN=CG4542
PE=2 SV=1"|e=2.964E-119|from=861|to=2123|frame=3|Fragment_1
TCVKFLFIPAYRSTDFEVHRNWLAITHSKPIYLWYFENTSEWTLDYPPFFAWFEFMLSHI
AKYFDPNMLNVKNLNYASDMTVLFQRLSVMVTDLLLLFGTRECSNSLGKSNSQPKTFLA
>Locus_5299_Transcript_4/4_Confidence_0.667_Length_2193|.|"sp|
Q9W3V8|ALG8_DROME Probable dolichyl pyrophosphate Glc1Man9GlcNAc2
alpha-1,3-glucosyltransferase OS=Drosophila melanogaster GN=CG4542
PE=2 SV=1"|e=2.964E-119|from=861|to=2123|frame=3|Fragment_2
VLILCNAGLFMVDHIHFQYNGMLYGILLLSISCIMKEQYLMSALWFSILLNLKHIFAYVA
PAYVVYLLGSYCLQGNFDLSKTAKRISKLVLIGFITCTVSFGPFIRQISQVMARLFPFKR
GLCHAYWAPNFWAIYNVADKASTITAKKLGYSVSNSTATMTGGLVEDVVHTTLPTITPSI
TFYLTFITIVPALIKLWRIIRFNKTPIVISPLQFIRCLVICGACSFLFGWHVHEKAILML
IIPMSISAVLEKEDAKIFLMLSTIGHYS
>Locus_5299_Transcript_4/4_Confidence_0.667_Length_2193|.|"sp|
Q9W3V8|ALG8_DROME Probable dolichyl pyrophosphate Glc1Man9GlcNAc2
alpha-1,3-glucosyltransferase OS=Drosophila melanogaster GN=CG4542
PE=2 SV=1"|e=2.964E-119|from=861|to=2123|frame=3|Fragment_3
KALIFISYSGIAFKFLSQLFP
>Locus_5299_Transcript_3/4_Confidence_0.222_Length_1259|.|"sp|
Q9W3V8|ALG8_DROME Probable dolichyl pyrophosphate Glc1Man9GlcNAc2
alpha-1,3-glucosyltransferase OS=Drosophila melanogaster GN=CG4542
PE=2 SV=1"|e=4.33127E-42|from=861|to=1259|frame=3|Fragment_1
TCVKFLFIPAYRSTDFEVHRNWLAITHSKPIYLWYFENTSEWTLDYPPFFAWFEFMLSHI
AKYFDPNMLNVKNLNYASDMTVLFQRLSVMVTDLLLLFGTRECCNSLGKSNSQPKTFLA
>Locus_5299_Transcript_3/4_Confidence_0.222_Length_1259|.|"sp|
Q9W3V8|ALG8_DROME Probable dolichyl pyrophosphate Glc1Man9GlcNAc2

alpha-1,3-glucosyltransferase OS=Drosophila melanogaster GN=CG4542
PE=2 SV=1"|e=4.33127E-42|from=861|to=1259|frame=3|Fragment_2
FLILCNAGLFMVDH
>Locus_5299_Transcript_1/4_Confidence_0.444_Length_1056|.|"sp|
Q9W3V8|ALG8_DROME Probable dolichyl pyrophosphate Glc1Man9GlcNAc2
alpha-1,3-glucosyltransferase OS=Drosophila melanogaster GN=CG4542
PE=2 SV=1"|e=8.60943E-78|from=3|to=986|frame=3|Fragment_1
LLLLFGTRECSNSLGKSNSQPKTFLA
>Locus_5299_Transcript_1/4_Confidence_0.444_Length_1056|.|"sp|
Q9W3V8|ALG8_DROME Probable dolichyl pyrophosphate Glc1Man9GlcNAc2
alpha-1,3-glucosyltransferase OS=Drosophila melanogaster GN=CG4542
PE=2 SV=1"|e=8.60943E-78|from=3|to=986|frame=3|Fragment_2
FLILCNAGLFMVDHIHFQYNGMLYGILLLSISCIMKEQYLMSALWFSILLNLKHIFAYVA
PAYVVYLLGSYCLQGNFDLSKTAKRISKLVLIGFITCTVSFGPFIRQISQVMARLFPFKR
GLCHAYWAPNFWAIYNVADKASTITAKKLGYSVSNSTATMTGGLVEDVVHTTLPTITPSI
TFYLTFITIVPALIKLWRIIRFNKTPIVISPLQFIRCLVICGACSFLFGWHVHEKAILML
IIPMSISAVLEKEDAKIFLMLSTIGHYS
>Locus_5299_Transcript_1/4_Confidence_0.444_Length_1056|.|"sp|
Q9W3V8|ALG8_DROME Probable dolichyl pyrophosphate Glc1Man9GlcNAc2
alpha-1,3-glucosyltransferase OS=Drosophila melanogaster GN=CG4542
PE=2 SV=1"|e=8.60943E-78|from=3|to=986|frame=3|Fragment_3
KALIFISYSGIAFKFLSQLFP
>Locus_10077_Transcript_1/1_Confidence_1.000_Length_1120|UNIGENE
REPRESENTATIVE TRANSCRIPT|sp|Q9W4C5|NAAT1_DROME Sodium-dependent
nutrient amino acid transporter 1 OS=Drosophila melanogaster
GN=NAAT1 PE=1 SV=2|e=2.1927E-111|from=3|to=1118|frame=-3|Fragment_1
FVIGKPVYYLEMTLGQFTSKSSVKIWALCPILKGIGIGQIISTCCVVSYYCCLMALTLFY
LYSSFASDLPWSYCKPEWEETINKICVDSAPSNSTTSTLQG
>Locus_10077_Transcript_1/1_Confidence_1.000_Length_1120|UNIGENE
REPRESENTATIVE TRANSCRIPT|sp|Q9W4C5|NAAT1_DROME Sodium-dependent
nutrient amino acid transporter 1 OS=Drosophila melanogaster
GN=NAAT1 PE=1 SV=2|e=2.1927E-111|from=3|to=1118|frame=-3|Fragment_2
NLTRGI
>Locus_10077_Transcript_1/1_Confidence_1.000_Length_1120|UNIGENE
REPRESENTATIVE TRANSCRIPT|sp|Q9W4C5|NAAT1_DROME Sodium-dependent
nutrient amino acid transporter 1 OS=Drosophila melanogaster
GN=NAAT1 PE=1 SV=2|e=2.1927E-111|from=3|to=1118|frame=-3|Fragment_3
SSSELFFVKEVLKENKLISSSLGLPDVNLTIC
>Locus_10077_Transcript_1/1_Confidence_1.000_Length_1120|UNIGENE
REPRESENTATIVE TRANSCRIPT|sp|Q9W4C5|NAAT1_DROME Sodium-dependent
nutrient amino acid transporter 1 OS=Drosophila melanogaster
GN=NAAT1 PE=1 SV=2|e=2.1927E-111|from=3|to=1118|frame=-3|Fragment_4
SYFLAIFPYVVMIALLIRAATLEGAGKGILFFIEPQWGELLNPKVWKEAVNQCFFSLAIS
FGPILIFSSYNNFDRNIHRDVLIVTTLDTFTSLLAGTTIFGILGNLAHNLNIDDIGQVIQ
SGTGLAFISYPDAISKFDMVPQ
>Locus_10077_Transcript_1/1_Confidence_1.000_Length_1120|UNIGENE
REPRESENTATIVE TRANSCRIPT|sp|Q9W4C5|NAAT1_DROME Sodium-dependent
nutrient amino acid transporter 1 OS=Drosophila melanogaster
GN=NAAT1 PE=1 SV=2|e=2.1927E-111|from=3|to=1118|frame=-3|Fragment_5
SAVALHGAIITVISDQF
>Locus_10077_Transcript_1/1_Confidence_1.000_Length_1120|UNIGENE
REPRESENTATIVE TRANSCRIPT|sp|Q9W4C5|NAAT1_DROME Sodium-dependent
nutrient amino acid transporter 1 OS=Drosophila melanogaster
GN=NAAT1 PE=1 SV=2|e=2.1927E-111|from=3|to=1118|frame=-3|Fragment_6

GIRYWKVALAASTVGFLIGLVYVTPEGQWLLNLVDY
>Locus_13104_Transcript_1/2_Confidence_0.750_Length_1001|.|sp|
Q9W4C5|NAAT1_DROME Sodium-dependent nutrient amino acid transporter
1 OS=Drosophila melanogaster GN=NAAT1 PE=1 SV=2|e=2.96707E-56|
from=1|to=600|frame=1|Fragment_1
YWKVALAASTVGFLIGLVYVTPEGQWLLNLVDYFGATFLIFALAIIQNIGVFWIYGLENY
CLDVEFMVGVRVGPYWRICWGFVTPVLMTIIFIYSLAVMKPLKYSELFYPDSYYAAGWTM
LAVGILQVPLWAIWIYLKNRKHSINDTLKNVFTPNEKWGPKSNKIRKEWIKFKTSAKERR
>Locus_13104_Transcript_1/2_Confidence_0.750_Length_1001|.|sp|
Q9W4C5|NAAT1_DROME Sodium-dependent nutrient amino acid transporter
1 OS=Drosophila melanogaster GN=NAAT1 PE=1 SV=2|e=2.96707E-56|
from=1|to=600|frame=1|Fragment_2
EAMNLNLFQHVMYSFLGKYK
>Locus_13104_Transcript_2/2_Confidence_0.750_Length_1001|.|sp|
Q9W4C5|NAAT1_DROME Sodium-dependent nutrient amino acid transporter
1 OS=Drosophila melanogaster GN=NAAT1 PE=1 SV=2|e=2.96707E-56|
from=1|to=600|frame=1|Fragment_1
YWKVALAASTVGFLIGLVYVTPEGQWLLNLVDYFGATFLIFALAIIQNIGVFWIYGLENY
CLDVEFMVGVRVGPYWRICWGFVTPVLMTIIFIYSLAVMKPLKYSELFYPDSYYAAGWTM
LAVGILQVPLWAIWIYLKNRKHSINDTLKNVFTPNEKWGPKSNKIRKEWIKFKTSAKERR
>Locus_13104_Transcript_2/2_Confidence_0.750_Length_1001|.|sp|
Q9W4C5|NAAT1_DROME Sodium-dependent nutrient amino acid transporter
1 OS=Drosophila melanogaster GN=NAAT1 PE=1 SV=2|e=2.96707E-56|
from=1|to=600|frame=1|Fragment_2
EAMNLNLFQHVMYSFLGKYK
>Locus_29275_Transcript_1/1_Confidence_1.000_Length_209|.|sp|Q9W4C5|
NAAT1_DROME Sodium-dependent nutrient amino acid transporter 1
OS=Drosophila melanogaster GN=NAAT1 PE=1 SV=2|e=4.09864E-13|from=3|
to=155|frame=-1|Fragment_1
VALHGAIITVISDQF
>Locus_29275_Transcript_1/1_Confidence_1.000_Length_209|.|sp|Q9W4C5|
NAAT1_DROME Sodium-dependent nutrient amino acid transporter 1
OS=Drosophila melanogaster GN=NAAT1 PE=1 SV=2|e=4.09864E-13|from=3|
to=155|frame=-1|Fragment_2
GIRYWKVALAASTVGFLIGLVYVTPEGQWLLNLVDY
>Locus_3907_Transcript_7/7_Confidence_0.438_Length_2688|UNIGENE
REPRESENTATIVE TRANSCRIPT|sp|Q9W534|MOODY_DROME G-protein coupled
receptor moody OS=Drosophila melanogaster GN=moody PE=2 SV=2|
e=1.6289E-114|from=1040|to=2167|frame=-3|Fragment_1
FSRPLLTF
>Locus_3907_Transcript_7/7_Confidence_0.438_Length_2688|UNIGENE
REPRESENTATIVE TRANSCRIPT|sp|Q9W534|MOODY_DROME G-protein coupled
receptor moody OS=Drosophila melanogaster GN=moody PE=2 SV=2|
e=1.6289E-114|from=1040|to=2167|frame=-3|Fragment_2
IVGILGNSLTVVALLRCPKVRNVAAAFIISLCFADLVFCSIVLPFSAARFVQ
>Locus_3907_Transcript_7/7_Confidence_0.438_Length_2688|UNIGENE
REPRESENTATIVE TRANSCRIPT|sp|Q9W534|MOODY_DROME G-protein coupled
receptor moody OS=Drosophila melanogaster GN=moody PE=2 SV=2|
e=1.6289E-114|from=1040|to=2167|frame=-3|Fragment_3
SLISSENEGVMGNVTLCKLVPFMRYGSVGVSLLSIAMITVNRYIMITHSGIYARVYRKRY
IAAMLIGAWIFSYGFQVPTLLGVWGAFAYDPRLGTCSVTKDSRGASSKTALFVVGFVTPC
IVIIACYARIFWVVHSSEVRMRQHAAKANNKPQN
>Locus_3907_Transcript_7/7_Confidence_0.438_Length_2688|UNIGENE
REPRESENTATIVE TRANSCRIPT|sp|Q9W534|MOODY_DROME G-protein coupled

receptor moody OS=Drosophila melanogaster GN=moody PE=2 SV=2|
e=1.6289E-114|from=1040|to=2167|frame=-3|Fragment_4
NGNSATPA
>Locus_3907_Transcript_7/7_Confidence_0.438_Length_2688|UNIGENE
REPRESENTATIVE TRANSCRIPT|sp|Q9W534|MOODY_DROME G-protein coupled
receptor moody OS=Drosophila melanogaster GN=moody PE=2 SV=2|
e=1.6289E-114|from=1040|to=2167|frame=-3|Fragment_5
KNGPQIARTPESSGARMKRNEWRITKMVLAIFLSFVVCYLPITIAKVADPEVKYPGLHIM
GYILLYLSACINPIIYVIMNKQYRQAYKTVLLCERPRIL
>Locus_3907_Transcript_7/7_Confidence_0.438_Length_2688|UNIGENE
REPRESENTATIVE TRANSCRIPT|sp|Q9W534|MOODY_DROME G-protein coupled
receptor moody OS=Drosophila melanogaster GN=moody PE=2 SV=2|
e=1.6289E-114|from=1040|to=2167|frame=-3|Fragment_6
NFTAGGNSSAQEKSKERLACSYNHSRTFLSIVS
>Locus_3907_Transcript_6/7_Confidence_0.438_Length_2123|.|sp|Q9W534|
MOODY_DROME G-protein coupled receptor moody OS=Drosophila
melanogaster GN=moody PE=2 SV=2|e=1.2466E-114|from=475|to=1602|
frame=-3|Fragment_1
FSRPLLTF
>Locus_3907_Transcript_6/7_Confidence_0.438_Length_2123|.|sp|Q9W534|
MOODY_DROME G-protein coupled receptor moody OS=Drosophila
melanogaster GN=moody PE=2 SV=2|e=1.2466E-114|from=475|to=1602|
frame=-3|Fragment_2
IVGILGNSLTVVALLRCPKVRNVAAAFIISLCFADLVFCSIVLPFSAARFVQ
>Locus_3907_Transcript_6/7_Confidence_0.438_Length_2123|.|sp|Q9W534|
MOODY_DROME G-protein coupled receptor moody OS=Drosophila
melanogaster GN=moody PE=2 SV=2|e=1.2466E-114|from=475|to=1602|
frame=-3|Fragment_3
SLISSENEGVMGNVTLCKLVPFMRYGSVGVSLLSIAMITVNRYIMITHSGIYARVYRKRY
IAAMLIGAWIFSYGFQVPTLLGVWGAFAYDPRLGTCSVTKDSRGASSKTALFVVGFVTPC
IVIIACYARIFWVVHSSEVRMRQHAAKANNKPQN
>Locus_3907_Transcript_6/7_Confidence_0.438_Length_2123|.|sp|Q9W534|
MOODY_DROME G-protein coupled receptor moody OS=Drosophila
melanogaster GN=moody PE=2 SV=2|e=1.2466E-114|from=475|to=1602|
frame=-3|Fragment_4
NGNSATPA
>Locus_3907_Transcript_6/7_Confidence_0.438_Length_2123|.|sp|Q9W534|
MOODY_DROME G-protein coupled receptor moody OS=Drosophila
melanogaster GN=moody PE=2 SV=2|e=1.2466E-114|from=475|to=1602|
frame=-3|Fragment_5
KNGPQIARTPESSGARMKRNEWRITKMVLAIFLSFVVCYLPITIAKVADPEVKYPGLHIM
GYILLYLSACINPIIYVIMNKQYRQAYKTVLLCERPRIL
>Locus_3907_Transcript_6/7_Confidence_0.438_Length_2123|.|sp|Q9W534|
MOODY_DROME G-protein coupled receptor moody OS=Drosophila
melanogaster GN=moody PE=2 SV=2|e=1.2466E-114|from=475|to=1602|
frame=-3|Fragment_6
NFTAGGNSSAQEKSKERLACSYNHSRTFLSIVS
>Locus_3907_Transcript_3/7_Confidence_0.375_Length_2017|.|sp|Q9W534|
MOODY_DROME G-protein coupled receptor moody OS=Drosophila
melanogaster GN=moody PE=2 SV=2|e=1.43994E-96|from=1040|to=2017|
frame=-1|Fragment_1
ADLVFCSIVLPFSAARFVQ
>Locus_3907_Transcript_3/7_Confidence_0.375_Length_2017|.|sp|Q9W534|
MOODY_DROME G-protein coupled receptor moody OS=Drosophila

melanogaster GN=moody PE=2 SV=2|e=1.43994E-96|from=1040|to=2017|
frame=-1|Fragment_2
SLISSDNEGVMGNVTLCKLVPFMRYGSVGVSLLSIAMITVNRYIMITHSGIYARVYRKRY
IAAMLIGAWIFSYGFQVPTLLGVWGAFAYDPRLGTCSVTKDSRGASSKTALFVVGFVTPC
IVIIACYARIFWVVHSSEVRMRQHAAKANNKPQN
>Locus_3907_Transcript_3/7_Confidence_0.375_Length_2017|.|sp|Q9W534|
MOODY_DROME G-protein coupled receptor moody OS=Drosophila
melanogaster GN=moody PE=2 SV=2|e=1.43994E-96|from=1040|to=2017|
frame=-1|Fragment_3
NGNSATPA
>Locus_3907_Transcript_3/7_Confidence_0.375_Length_2017|.|sp|Q9W534|
MOODY_DROME G-protein coupled receptor moody OS=Drosophila
melanogaster GN=moody PE=2 SV=2|e=1.43994E-96|from=1040|to=2017|
frame=-1|Fragment_4
KNGPQIARTPESSGARMKRNEWRITKMVLAIFLSFVVCYLPITIAKVADPEVKYPGLHIM
GYILLYLSACINPIIYVIMNKQYRQAYKTVLLCERPRIL
>Locus_3907_Transcript_3/7_Confidence_0.375_Length_2017|.|sp|Q9W534|
MOODY_DROME G-protein coupled receptor moody OS=Drosophila
melanogaster GN=moody PE=2 SV=2|e=1.43994E-96|from=1040|to=2017|
frame=-1|Fragment_5
NFTAGGNSSAQEKSKERLACSYNHSRTFLSIVS
>Locus_3907_Transcript_2/7_Confidence_0.438_Length_1327|.|sp|Q9W534|
MOODY_DROME G-protein coupled receptor moody OS=Drosophila
melanogaster GN=moody PE=2 SV=2|e=2.43956E-75|from=3|to=806|
frame=-3|Fragment_1
FSRPLLTF
>Locus_3907_Transcript_2/7_Confidence_0.438_Length_1327|.|sp|Q9W534|
MOODY_DROME G-protein coupled receptor moody OS=Drosophila
melanogaster GN=moody PE=2 SV=2|e=2.43956E-75|from=3|to=806|
frame=-3|Fragment_2
IVGILGNSLTVVALLRCPKVRNVAAAFIISLCFADLVFCSIVLPFSAARFVQ
>Locus_3907_Transcript_2/7_Confidence_0.438_Length_1327|.|sp|Q9W534|
MOODY_DROME G-protein coupled receptor moody OS=Drosophila
melanogaster GN=moody PE=2 SV=2|e=2.43956E-75|from=3|to=806|
frame=-3|Fragment_3
SLISSENEGVMGNVTLCKLVPFMRYGSVGVSLLSIAMITVNRYIMITHSGIYARVYRKRY
IAAMLIGAWIFSYGFQVPTLLGVWGAFAYDPRLGTCSVTKDSRGASSKTALFVVGFVTPC
IVIIACYARIFWVVHSSEVRMRQHAAKANNKPQN
>Locus_3907_Transcript_2/7_Confidence_0.438_Length_1327|.|sp|Q9W534|
MOODY_DROME G-protein coupled receptor moody OS=Drosophila
melanogaster GN=moody PE=2 SV=2|e=2.43956E-75|from=3|to=806|
frame=-3|Fragment_4
NGNSATP
>Locus_3907_Transcript_2/7_Confidence_0.438_Length_1327|.|sp|Q9W534|
MOODY_DROME G-protein coupled receptor moody OS=Drosophila
melanogaster GN=moody PE=2 SV=2|e=2.43956E-75|from=3|to=806|
frame=-3|Fragment_5
AKSG
>Locus_3907_Transcript_2/7_Confidence_0.438_Length_1327|.|sp|Q9W534|
MOODY_DROME G-protein coupled receptor moody OS=Drosophila
melanogaster GN=moody PE=2 SV=2|e=2.43956E-75|from=3|to=806|
frame=-3|Fragment_6
PQIARTPESSGARMKRNEWRI
>Locus_3907_Transcript_4/7_Confidence_0.438_Length_1327|.|sp|Q9W534|

MOODY_DROME G-protein coupled receptor moody OS=Drosophila
melanogaster GN=moody PE=2 SV=2|e=2.43956E-75|from=3|to=806|
frame=-3|Fragment_1
FSRPLLTF
>Locus_3907_Transcript_4/7_Confidence_0.438_Length_1327|.|sp|Q9W534|
MOODY_DROME G-protein coupled receptor moody OS=Drosophila
melanogaster GN=moody PE=2 SV=2|e=2.43956E-75|from=3|to=806|
frame=-3|Fragment_2
IVGILGNSLTVVALLRCPKVRNVAAAFIISLCFADLVFCSIVLPFSAARFVQ
>Locus_3907_Transcript_4/7_Confidence_0.438_Length_1327|.|sp|Q9W534|
MOODY_DROME G-protein coupled receptor moody OS=Drosophila
melanogaster GN=moody PE=2 SV=2|e=2.43956E-75|from=3|to=806|
frame=-3|Fragment_3
SLISSENEGVMGNVTLCKLVPFMRYGSVGVSLLSIAMITVNRYIMITHSGIYARVYRKRY
IAAMLIGAWIFSYGFQVPTLLGVWGAFAYDPRLGTCSVTKDSRGASSKTALFVVGFVTPC
IVIIACYARIFWVVHSSEVRMRQHAAKANNKPQN
>Locus_3907_Transcript_4/7_Confidence_0.438_Length_1327|.|sp|Q9W534|
MOODY_DROME G-protein coupled receptor moody OS=Drosophila
melanogaster GN=moody PE=2 SV=2|e=2.43956E-75|from=3|to=806|
frame=-3|Fragment_4
NGNSATP
>Locus_3907_Transcript_4/7_Confidence_0.438_Length_1327|.|sp|Q9W534|
MOODY_DROME G-protein coupled receptor moody OS=Drosophila
melanogaster GN=moody PE=2 SV=2|e=2.43956E-75|from=3|to=806|
frame=-3|Fragment_5
AKSG
>Locus_3907_Transcript_4/7_Confidence_0.438_Length_1327|.|sp|Q9W534|
MOODY_DROME G-protein coupled receptor moody OS=Drosophila
melanogaster GN=moody PE=2 SV=2|e=2.43956E-75|from=3|to=806|
frame=-3|Fragment_6
PQIARTPESSGARMKRNEWRI
>Locus_3907_Transcript_5/7_Confidence_0.375_Length_1327|.|sp|Q9W534|
MOODY_DROME G-protein coupled receptor moody OS=Drosophila
melanogaster GN=moody PE=2 SV=2|e=2.43956E-75|from=3|to=806|
frame=-3|Fragment_1
FSRPLLTF
>Locus_3907_Transcript_5/7_Confidence_0.375_Length_1327|.|sp|Q9W534|
MOODY_DROME G-protein coupled receptor moody OS=Drosophila
melanogaster GN=moody PE=2 SV=2|e=2.43956E-75|from=3|to=806|
frame=-3|Fragment_2
IVGILGNSLTVVALLRCPKVRNVAAAFIISLCFADLVFCSIVLPFSAARFVQ
>Locus_3907_Transcript_5/7_Confidence_0.375_Length_1327|.|sp|Q9W534|
MOODY_DROME G-protein coupled receptor moody OS=Drosophila
melanogaster GN=moody PE=2 SV=2|e=2.43956E-75|from=3|to=806|
frame=-3|Fragment_3
SLISSENEGVMGNVTLCKLVPFMRYGSVGVSLLSIAMITVNRYIMITHSGIYARVYRKRY
IAAMLIGAWIFSYGFQVPTLLGVWGAFAYDPRLGTCSVTKDSRGASSKTALFVVGFVTPC
IVIIACYARIFWVVHSSEVRMRQHAAKANNKPQN
>Locus_3907_Transcript_5/7_Confidence_0.375_Length_1327|.|sp|Q9W534|
MOODY_DROME G-protein coupled receptor moody OS=Drosophila
melanogaster GN=moody PE=2 SV=2|e=2.43956E-75|from=3|to=806|
frame=-3|Fragment_4
NGNSATP
>Locus_3907_Transcript_5/7_Confidence_0.375_Length_1327|.|sp|Q9W534|

MOODY_DROME G-protein coupled receptor moody OS=Drosophila melanogaster GN=moody PE=2 SV=2|e=2.43956E-75|from=3|to=806|
frame=-3|Fragment_5
AKSG
>Locus_3907_Transcript_5/7_Confidence_0.375_Length_1327|.|sp|Q9W534|
MOODY_DROME G-protein coupled receptor moody OS=Drosophila melanogaster GN=moody PE=2 SV=2|e=2.43956E-75|from=3|to=806|
frame=-3|Fragment_6
PQIARTPESSGARMKRNEWRI
>Locus_3907_Transcript_1/7_Confidence_0.188_Length_1245|.|sp|Q9W534|
MOODY_DROME G-protein coupled receptor moody OS=Drosophila melanogaster GN=moody PE=2 SV=2|e=4.15521E-13|from=1040|to=1243|
frame=-3|Fragment_1
LYLSACINPIIYVIMNKQYRQAYKTVLLCERPRIL
>Locus_3907_Transcript_1/7_Confidence_0.188_Length_1245|.|sp|Q9W534|
MOODY_DROME G-protein coupled receptor moody OS=Drosophila melanogaster GN=moody PE=2 SV=2|e=4.15521E-13|from=1040|to=1243|
frame=-3|Fragment_2
NFTAGGNSSAQEKSKERLACSYNHSRTFLSIVS
>Locus_14715_Transcript_1/1_Confidence_1.000_Length_320|.|sp|Q9W534|
MOODY_DROME G-protein coupled receptor moody OS=Drosophila melanogaster GN=moody PE=2 SV=2|e=2.39329E-34|from=37|to=318|
frame=1|Fragment_1
QIARTPESSGARMKRNEWRITKMVLAIFLSFVVCYLPITIAKVADPEVKYPGLHIMGYIL
LYLSACINPIIYVIMNKQYRQAYKTVLLCERPRI
>Locus_23513_Transcript_1/1_Confidence_1.000_Length_320|.|sp|Q9W534|
MOODY_DROME G-protein coupled receptor moody OS=Drosophila melanogaster GN=moody PE=2 SV=2|e=2.39329E-34|from=37|to=318|
frame=1|Fragment_1
QIARTPESSGARMKRNEWRITKMVLAIFLSFVVCYLPITIAKVADPEVKYPGLHIMGYIL
LYLSACINPIIYVIMNKQYRQAYKTVLLCERPRI

>Locus_24585_Transcript_1/1_Confidence_1.000_Length_292|UNIGENE REPRESENTATIVE TRANSCRIPT|sp|Q9W5D4|POMT2_DROME Protein O-mannosyl-transferase 2 OS=Drosophila melanogaster GN=tw PE=2 SV=1|
e=2.00627E-44|from=2|to=289|frame=2|Fragment_1
VFGVITLLTFCTRFYKVTEPDHVCWDETHFGKMASWYINRTFFFDVHPPLGKMLIALSGT
LTGYDGTYPFEKPGDKYNGTRYEGMRIFCTAVGAAI
