# Supplementary material for: A reverse vaccinology approach to the identification and characterization of Ctenocephalides felis candidate protective antigens for the control of cat flea infestations
Source: Parasit Vectors. 2018 Jan 18;11:43. doi: 10.1186/s13071-018-2618-x (PMC5774092; doi:10.1186/s13071-018-2618-x)
Supplement: Supplementary file 2 — Coding sequences for selected candidate protective antigens. Dataset 3. De novo sequence assembly process for sequences F5 and F6. (PDF 148 kb) [file 13071_2018_2618_MOESM2_ESM.pdf]

Additional file 2. Dataset 2. Coding sequences for selected candidate protective antigens.

Sequence ID: F1

>gi|241896700|emb|FN421429.1| Ctenocephalides felis argk1 gene for arginine kinase 1
ATGGTTGACGCAGCCGTCTTGGACAAATTGGAATCTGGCTACGCCAAATTGGCAGCCTCAGATTCCAAAT
CCCTTTTGAAGAAACATTTAACAAAGGAAATCTTTGACAACCTCAAAACCAAGAAGACTTCCTTTGGCTC
TACCTTGCTTGACTGCATCCAGTCTGGTAAGATCTAGGAAACTAACAATAATTTTCATTGATATTATGAT
ATTATAATAAGCTCATGTCTCATTTTGTATTATTTTAGGTTTGGAAAACCATGATTCAGGTGTAGGTATC
TATGCCCCAGATGCTGAAGCTTACTCCGTTTTTGCTGATCTGTTCGACCCGATCATTGAAGAATATCACT
TCGGTTTCAAGAAAACTGACAAGCACCCCCCCAAGAACTGGGGTGATGTAGATACTTTGGGCAACTTGGA
CCCCACTGGTGAATTTGTTGTATCCACCCGTGTCCGTTGTGGACGTTCCATGGAAGGCTATCCATTCAAC
CCTTGCTTGACTGAAGCCCAATACAAGGAAATGGAAGAAAAAGTATCCAGCACACTTTCTGGATTGGAAG
GAGAATTGAAAGGAAAATTCCACCCATTGACTGGTATGCCAAAAGATGTTCAGCAACAGCTCATTGATGA
TCACTTCTTATTCAAGGAGGGCGATCGTTTCTTGCAATCTGCTAATGCTTGCCGTTTCTGGCCAACTGGA
CGTGGTATCTATCACAATGATAACAAAACTTTCCTCGTCTGGTGCAATGAGGAAGATCACTTGAGAATCA
TCTCCATGCAAATGGGAGGAGATTTAGGCTGTGTGTACCGTCGTCTTGTAAATGCTGTGAATGATATTGA
GAAGCGTATTCCTTTCTCTCACCACGATAGATTAGGTTTCCTCACTTTCTGTCCCACTAACTTGGGAACT
ACTGTCCGTGCCTCTGTGCACATCAAGGTCCCTAAGTTGGCTGCTAACCGCGCCAAGTTGGAAGAAGTTG
CTGGACGTTACAATCTCCAAGTTCGTGGAACTCGTGGTGAACACACTGAAGCTGAAGGTGGTGTCTACGA
TATCTCCAACAAGAGGCGCATGGGCCTGACTGAATACCAAGCTGTCAAGGAGATGCACGATGGCATTGCT
GAACTCATTAAGATGGAGAAAGAAATGTAA

Sequence of the primers used for cloning coding regions by RT-PCR:
Forward: 5′-CACCATGGTTGACGCAGCCGTCTT-3′
Reverse: 5′-TTACATTTCTTTCTCCATCTTA-3′

Sequence ID: F2

>gb|S66043.1|:122-3235 sodium pump alpha subunit [Ctenocephalides felis=cat fleas, mRNA, 3369 nt]
ATGGATGATAAGCATGGGCGTTCCGATTCGTATCGCGTGGCTACAGTACCTACCATAGATGACAATTTGA
CAGCAGACGGTCAATACAAGTCGCGACGTAAAACGCCAACGAAAAAGCAAAGGAAGGAAGGAGAGCTTGA
TGACTTGAAACAAGAATTAGATATCGATTTTCACAAAGTATCACCCGAAGAATTATATCAACGATTTAAT
ACTCACCCCGAAAATGGTCTTAGTCACGCCAAGGCGAAAGAAAACTTAGAAAGAGATGGACCGAATGCTT
TGACCCCGCCGAAACAAACACCAGAATGGGTCAAATTTTGCAAGAACTTGTTTGGAGGATTCGCCTTGTT
GTTGTGGATCGGTGCCATTTTATGTTTTGTCGCATACTCCATCCAAGCTAGTACTGTGGAAGAACCAGCA
GATGATAATTTGTATCTTGGTATTGTATTGGCGGCTGTAGTTATAGTTACTGGTATATTTTCGTATTACC
AAGAATCCAAGAGTTCCAAAATTATGGAAAGTTTCAAAAACATGGTTCCACAGTTTGCAACAGTATTACG
TGAAGGTGAAAAATTAACCTTACGAGCGGAAGATTTAGTACTCGGAGATGTCGTAGAAGTGAAATTCGGC
AGCAGGATACCTGCAGATATCCGTATTATTGAAAGCCGAGGATTCAAGGTAGACAAGTCTTCCTTGACTG
GTGAATCAGAACCTCAATCTCGAGGTCCCGAGTTTACAAATGAAAAGCCTTTAGAAACGAAGAACTTGGC
GTTCTTCTCTATCAACGCCGTCGAAGGTACTGCCAAAGGTGTCGTTATCAGCTGTGGAGACAACACTGTC
ATGGGTCGTATTGCCGGCTTGGCTTCAGGCTTGGACACTGGGGAGACTCCAATCGCTAAAGAAATTCATC
ACTTCATTCATCTCATCACTGGAGTCGCTGTATTTTTAGGTGTAACATTCTTTGTTATTGCAATTATTTT
GAACTACCATTGGTTAGACGCTGTCATCTTCTTGATTGGTATCATCGTCGCTAATGTCCCTGAAGGTTTA
TTGGCTACTGTAACCGTATGTCTAACCCTTACTGCTAAGCGTATGGCATCCAAGAATTGTCTTGTCAAGA
ATCTTGAAGCTGTAGAAACTCTTGGTTCTACCTCAACTATCTGCTCAGACAAAACTGGCACACTGACCCA
GAACAGAATGACTGTAGCCCACATGTGGTTTGACAACCAGATTATTGAAGCCGACACCACTGAAGATCAA
TCTGGAGTCGTATATGACAGAACCAGCCCTGGTTTCAAAGCTTTGGCGCGCATTGCAACTTTGTGCAACA
GAGCAGAATTCAAGGGAGGTCAGGAAGGTGTACCCATCTTGAAAAAAGAAGTCAGTGGTGATGCATCTGA
AGCTGCTCTTCTCAAATGTATGGAACTGGCTTTAGGAGATGTTATGTCTATTCGAAAACGAAATAAGAAA
GTCTGTGAAATTCCATTTAACTCCACAAACAAATACCAGGTTTCCATTCACGAAACTGAAGATGCGTCAG
ACCCCCGTCATGTAATGGTTATGAAAGGAGCTCCTGAAAGAATCTTAGAAAAATGTTCCACCATCTTCAT

TGGAGGAAAGGAAAAACTACTGGACGAAGAGATGAAAGAAGCTTTCAATAATGCATATCTGGAATTGGGC
GGTCTTGGAGAGCGTGTATTGGGCTTTTGTGATCTCATGTTGCCTACAGACAAATTCCCCTTAGGTTTCA
AATTCGACAGCGATGATCCCAACTTCCCAATTGAAAACCTTAGATTTGTTGGACTCATGTCTATGATTGA
TCCTCCTAGAGCTGCCGTACCTGACGCTGTTGCCAAGTGCCGATCTGCTGGTATCAAGGTTATCATGGTT
ACAGGAGATCATCCAATCACTGCAAAAGCCATTGCTAAATCAGTGGGTATCATCTCAGAGGGTAATGAAA
CTGTAGAAGATATCGCGCAAAGATTGAATATTCCTGTATCAGAGGTAAATCCACGAGAAGCCAAGGCAGC
TGTTGTACATGGAACTGAGCTTAGGGAACTCAACTCTGATCAGCTCGATGAAATTCTTAGGTATCACACT
GAAATTGTATTTGCTCGGACATCTCCTCAACAAAAGCTGATTATTGTTGAAGGATGCCAACGTATGGGTG
CTATTGTCGCCGTAACTGGTGATGGTGTGAATGACTCACCTGCTTTGAAAAAGGCTGATATTGGTGTTGC
CATGGGTATTGCCGGATCTGATGTATCAAAACAGGCTGCTGACATGATTTTATTAGATGACAACTTTGCA
TCTATTGTCACTGGTGTGGAAGAGGGTCGTTTGATATTCGACAATCTGAAGAAATCTATTGCTTACACAT
TGACTTCAAATATCCCAGAAATTTCACCATTCTTGGCATTCATCTTATGTGATATCCCGCTACCTTTGGG
AACTGTAACAATCTTGTGCATTGACTTGGGAACTGACATGGTGCCTGCCATCTCATTGGCCTACGAACAT
GCTGAAGCTGATATCATGAAGAGGCCGCCTAGAGATCCAGTCAATGACAAACTTGTAAATTCCAGACTTA
TCTCTATGGCTTATGGGCAAATCGGAATGATTCAAGCAGCTGCTGGATTCTTTGTATACTTTGTAATCAT
GGCTGAAAATGGATTCTTACCCATGAAATTGTTTGGAATTAGAAAACAATGGGACTCGAAAGCTGTCAAT
GATCTAACAGATTCTTATGGACAAGAATGGACATACAGAGATCGCAAGACTCTTGAATATACCTGCCACA
CTGCCTTCTTCGTCTCTATTGTCGTTGTACAATGGGCTGATTTGATTGTCTGCAAAACGCGCCGTAATTC
CTTGTTACACCAGGGAATGAGAAATTGGGCTCTCAACTTTGGTCTAGTTTTTGAAACTGCCTTAGCAGCA
TTCCTGTCATACACACCAGGAATGGACAAGGGACTGAGGATGTTCCCACTGAAGTTTGTTTGGTGGCTGC
CTGCTCTGCCATTCATGATTTCCATCTTCATCTATGATGAGACTAGAAGATTTTACCTACGTCGCAACCC
TGGTGGTTGGTTAGAACAAGAAACATATTATTAA

Sequence of the primers used for cloning coding regions by RT-PCR:
Forward: 5’-CACCATGGATGATAAGCATGGGCGTT-3’
Reverse: 5’-TTAATAATATGTTTCTTGTTCTAACC-3’

Sequence ID: F3

>gi|25527510|gb|AY150533.1| Ctenocephalides felis serpin 4 mRNA, partial cds
ATGGCGGATCCCCAGGAATTGTCTACAAGTATTAACCAGTTTGCTGGAAGCCTGTACAATACGGTTGCTTC
TGGCAACAAAGACAATCTCATCATGTCCCCATTGTCTGTACAAACTGTTCTATCCCTGGTGTCAATGGGAG
CTGGTGGTAATACTGCCACACAAATAGCTGCTGGTTTACGTCAGCCTCAATCAAAAGAAAAAATTCAAGAT
GACTACCATGCATTGATGAACACTCTTAATACACAAAAAGGTGTAACTCTGGAAATTGCCAACAAAGTTTA
CGTTATGGAAGGCTATACATTGAAACCCACCTTCAAAGAAGTTGCCACCAACAAATTCTTAGCTGGAGCAG
AAAACTTGAACTTTGCCCAAAATGCTGAAAGCGCTAAAGTTATCAACACTTGGGTTGAAGAAAAAACTCAT
GACAAAATTCATGATTTGATCAAAGCCGGTGATCTAGACCAGGATTCAAGAATGGTTCTTGTCAATGCATT
GTACTTCAAGGGTCTTTGGGAGAAACAATTCAAGAAGGAAAACACTCAAGACAAACCTTTCTATGTTACTG
AAACAGAGACAAAGAATGTACGAATGATGCACATTAAGGATAAATTCCGTTATGGAGAATTTGAAGAATTA
GATGCCAAGGCTGTAGAATTGCCCTACAGGAACTCAGATTTGGCCATGTTAATCATTTTGCCAAACAGCAA
AACTGGTCTCCCCGCTCTTGAAGAAAAATTACAAAATGTTGACTTGCAAAACTTGACTCAACGCATGTACT
CTGTTGAAGTTATTTTGGATCTGCCTAAATTCAAGATTGAATCTGAAATTAATTTGAATGATCCTCTGAAA
AAGTTGGGTATGTCTGATATGTTTGTTCCTGGAAAAGCTGATTTCAAAGGATTGCTTGAAGGATCTGATGA
GATGTTATATATTTCTAAAGTAATTCAAAAAGCTTTCATTGAAGTAAATGAAGAAGGTGCTGAAGCTGCAG
CTGCCACAGGCGTGATGTTAATGATGCGTTGTATGCCAATGATGCCAATGGCCTTCAATGCTGAGCATCCA
TTCCTGTACTTCTTACACAGCAAAAATTCTGTTCTATTCAATGGTCGTCTTGTTAAACCAACAACTGAATA
A

Sequence of the primers used for cloning coding regions by RT-PCR:
Forward: 5’-CACCATGGCGGATCCCCAGGAATTGT-3’
Reverse: 5’-TTATTCAGTTGTTGGTTTAACT-3’

Sequence ID: F4

>gi|20502971|gb|AF503908.1| Ctenocephalides felis juvenile hormone epoxide hydrolase mRNA, complete cds

ATGGGTAAATGTTGTCGTATGCTCATTTTTGCTGCAATAGCTGGTATAGCAGTGTTGTATTACCAAATTAC
GAAAGAACTTCCAAAACCAAATATACCTCTCGACACTTGGTGGGGTCCAGGAAAGCCACAAAATGTTGACA
TATCAATAAGACCGTTTAAAATTAATATTAATAACAAAGTGATTGAAAATCTGAAACTAAAACTCAATGAT
GTTCAATATACTTTACCTTTAGAGGGCATCAATTTTGAATATGGTTTCAATACAGATTCCCTGAAAAAGAT
TGTAGATTTTTGGCGAACTCAATATAATTGGCGTGAACGTGAAGCATTATTAAATAAATATCCACACTTCA
AAACAAATATTCAAGGCCTGGATATTCATTATGTCCACATAAAACCACAGGTCTCTAAAAATATTGAAGTT
TTGCCTTTGGTAATGATCCATGGTTGGCCAGGATCTTTTGTGGAATTCTACAAGATCATACCTATGTTGAC
AACTCCAAGAGCAGGTTACAATTTCGTATTCGAATTGATATTGCCTAGTATTCCTGGATACGGCTTTTCAC
AGGCTGCAGCTAAACCTGGTCTTGGATCGACTCAGGTCGCCGTTATAATGCGCAATTTGATGGAGCGCATT
GGATTCAAAAAATATTATGTACAAGGAGGCGACTGGGGTTCTATGATCATAAGCGCGATGTCAACATTATT
TCCAGAAAATGTCCTGGGACAGCACTCAAACATGTGTTTTGTCAATACTCCATCATCAAATATCAAGGCTA
TAATTGGAAGCTTTTTCCCGGAATCGTTTGCTGGCACGGGAAATGCGCATAAAATGTATCCCATGAGTGAA
CACTTTTTCACACTTTTGGAAGAAATGGGTTATTTGCATCTACAAGCTACCAAACCAGATACAGTGGGCGT
TGCTTTAAGAGATTCACCAGCTGGTTTAGCAGCTTATATTTTGGAGAAATTTTCAACATGGACTAACAGAT
CTTGGAGGTCAGTTAAAGATGGAAACTTGCTGTTAAAATACAATATTCCTGAACTTTTAGACAATGTCATG
ATATACTACGTTACTGATTCCATTACTACTTCAATGAGATTATATGCAGAATCATTCACAAAAGCACACCT
TGCTTTGAACTTAGATAGGGTGCGCAATCATGTCCCAGCAGCCTGCGCAAAATTTCCAAACGAGTTGGCTT
ATGTGACCGATTGCCAACTTGCTGAGAAATATAAAACTTTATTGCAGTCCAATGACATGCCAAGTGGTGGC
CATTTTGCAGCATTTGAGGAACCTGGTCTTTTAGCAGAAGACATTTTCACTGCGGTGAAAAAGTTTAAAGA
ATTTTATTCCAAAAAAGCTGAAAGCCAAAAGAAAGCTGATTTGTGA

Sequence of the primers used for cloning coding regions by RT-PCR:
Forward: 5’-CACCATGGGTAAATGTTGTCGTATGC-3’
Reverse: 5’-TCACAAATCAGCTTTCTTTTGG-3’

Sequence ID: F5

>De_novo_sequence_assembly_of_Locus_4025_Transcript_2/7|Locus_18073_Tra
nscript_1/1|Locus_26648_Trancript_1/1|Q7KVA1|Xylosyltransferase_oxt
ATGAAATGTCCTGGAGATCCCAAAGAAATTTGTGGTGGCTTTTTTAAAATGAACATATATGAAACAGGAAT
TTCAAAATTCTCACCTCAAACTGCAGTAACTTTAACAACAAATGATACAGAACCTAAAGTACGCATTGTAT
ATTTGCTGACTTTAAATGGACGAGCAGTACGTCAAGTTTACAGATTAATTAAAGCTCTATTTCATGTAGAT
CATTATTTTTATATTCACGTAGACTCTCGCCAAGATTACATGTTTCGTCAACTTTTGCAATTAGAAAATAA
CTTTCCAAATATACGTCTTTCTCGCAATCGTTTTTCTACTATTTGGGGAGGTGCATCTTTACTCCAAATGT
TGCTAGCTTCTATGAATGAACTGTTAAAATCTGATTGGAAGTGGGATTTTATTATAAATTTAAGTGAAAGT
GATTTTCCAATTAAATCAAATGAAAGACTTGTGCAATTCTTAACAGCAAATAAAAATAAAAACTTTGTGAA
ATCACATGGGCGAGAAGTACAAAGATTTATTCAGAAGCAAGGCTTAGACAAAACTTTCATTGAATGTGATA
CGCATATGTGGAGAACTGGTGATAGAACCTTGCCATGGGGTATTCAAATTGATGGCGGTAGTGATTGGATA
ACTCTGACTAGAGACTTTGTGAAATATATCACACAAGAACAAGATGCCCTTGTCAAGGGCCTAATGACTGT
TTTTAGGCATACTTTATTACCTGCTGAATCATTTTTCCATACAGTTTTACGCAACTC

Sequence ID: F6

>De_novo_sequence_assembly_of_Locus_4149_Transcript_2/4_Confidence_0.70
0_Length_2043|Q9VAF0|
ATGAATTCCTCAATTTGGGAGTCAGACTCAGCCTTCAGATTTATTTACACTGATTTAGTTATGGAATACAT
ACCAGAATATTTCAAAGACTGGCAATATCATCCATGGATTTTTTCGGTCTTAGGTTCTATTTTAATTGGAT
TAAGTGGTGTGTTGCCTTTACTTATAATTCCATTGGAAGGAGGAGAACATTTTAAAAGTGGAGGAGGGGGC
AAACTTTTGCGCGTATTGCTCAGTTTTGCAGTAGGAGGTCTGCTCGGAGATGTGTTTTTGCATTTGTTGCC
AGAAGCATGGGAGAACTACAGTAACAAAGGAGAGCGACTCGTAGTCATGTACGAATTCGTAGATTCAGGAG
GAGGTAGTCCATCAATGACATGCGGGTGCTGGGTATTAGCTGGCCTCTTGTTATTCACGATGGTCGAGAAG
TTATTTGCTGGTTACATGGAAGATGATGACGTTTCCGAGGAAAAGACCGAACGAAAGCGCAAGAAAATGTC
TAAAATTAATTGTTTGATAAAGAACAACAATGTCTTGAGGGCTAGAAACGAAAATGGACGATTCTCGACCA
GCAAATGCTCAGGCGGACAAAATGGAAAAATGTGTGACATTGAAGTTATAAATGGAGCTATCAAAAACGGC
TGTTGTATGGTCGAGGAGCTAGAGAAAGAGGCTGTGAGCAACAAGTCTAAGCATGTTTCTGGATACTTAAA
TCTCATGGCAAACTCAATTGATAATTTCACACACGGTCTTGCTGTTGGTGGTTCTTTCATTGTTTCATTCA
AACTAGGAATTTTAACAACATTCGCAATTCTAGTGCATGAGATTCCACACGAAGTGGGCGATTTTGCAATT

TTGTTGCGGTCAGGTTTCTCGCGGTGGGACGCGGCTCGAGCGCAACTCATGACCGCTGGCGCCGGCCTGCT
TGGAGCTATGACCGCAATCGGCGGAAGTCGGGCTACGAGTGCAATGGAAGCTAGAACATGCTGGATAATGC
CCTTCACAGCCGGCGGATTTCTCCACATAAGTTTAGTTACAGTGCTACCTGACTTGCTGCAAGAAAGGGAT
CCAAAAGAGTCGATTAAACAATTTGGAGCATTGATTGGTGGTATAGTTCTAATGGCATCATTGACCACATT
ATTGGAGTGA

Additional file 2. Dataset 3. De novo sequence assembly process for sequences F5 and F6.

De novo sequence assembly for sequence F5: *Drosophila melanogaster* Xylosyltransferase (oxt) Q7KVA1.

1. Protein oriented transcripts for Q7KVA1.

```
>Locus_167_Transcript_1/1_Confidence_1.000_Length_302|Q7KVA1|Xylosyltra
nsferase_oxt
TTAACAGCAAATAAAAATAAAAACTTTGTGAAATCACATGGGCGAGAAGTACAAAGATTT
ATTCAGAAGCAAGGCTTAGACAAAACTTTCATTGAATGTGATACGCATATGTGGAGAACA
GGTGATAGAACATTGCCATGGGGTATTCAAATTGATGGCGGTAGTGATTGGATAACTCTG
ACTAGAGAGTTTGTGAAATATATCACACAAGAACAAGATGCCCTTGTCAAGGGCCTAATG
ACTGTTTTTAGGCATACTTTATTACCTGCTGAATCATTTTTCCATACAGTTTTACGCAAC
TC
>Locus_4025_Transcript_1/7_Confidence_0.188_Length_245|Q7KVA1|Xylosyltr
ansferase_oxt
GAACCTAAAGTGCGCATTGTATATTTGCTAACTTTAAATGGGCGAGCAGTACGTCAAGTT
TACAGATTAATTAAAGCTCTATTTCATGTAGATCATTATTTTTATATTCACGTAGACTCT
CGCCAAGATTACATGTTTCGTCAACTTTTGCAATTAGAAAATAACTTTCCAAATATACGT
CTTTCTCGCAATCGTTTTTCTACTATTTGGGGAGGTGCATCTTTACTCCAAATGTTGCTA
GCTTC
>Locus_4025_Transcript_2/7_Confidence_0.312_Length_368|Q7KVA1|Xylosyltr
ansferase_oxt
AATATGAAATGTCCTGGAGATCCCAAAGAAATTTGTGGTGGCTTTTTTAAAATGAACATA
TATGAAACAGGAATTTCAAAATTCTCACCTCAAACTGCAGTAACTTTAACAACAAATGAT
ACAGAACCTAAAGTACGCATTGTATATTTGCTGACTTTAAATGGACGAGCAGTACGTCAA
GTTTACAGATTAATTAAAGCTCTATTTCATGTAGATCATTATTTTTATATTCACGTAGAC
TCTCGCCAAGATTACATGTTTCGTCAACTTTTGCAATTAGAAAATAACTTTCCAAATATA
CGTCTTTCTCGCAATCGTTTTTCTACTATTTGGGGAGGTGCATCTTTACTCCAAATGTTG
CTAGCTTC
>Locus_8527_Transcript_1/1_Confidence_1.000_Length_299|Q7KVA1|Xylosyltr
ansferase_oxt
TTTCCAAATATACGTCTTTCTCGCAATCGTTTTTCTACTATTTGGGGAGGTGCATCTTTA
CTCCAAATGTTGCTAGCTTCTATGAATGAACTGTTAAAATCTGATTGGAAATGGGATTTT
ATTATAAATTTAAGTGAAAGTGATTTTCCAATTAAATCAAATGAAAGACTTGTGCAATTT
TTAACAGCAAATAAAAATAAAAACTTTGTGAAATCACATGGGCGAGAAGTACAAAGATTT
ATTCAGAAGCAAGGCTTAGACAAAACTTTCATTGAATGTGATACGCATATGTGGAGAAC
>Locus_18073_Transcript_1/1_Confidence_1.000_Length_299|Q7KVA1|Xylosylt
ransferase_oxt
TTTCCAAATATACGTCTTTCTCGCAATCGTTTTTCTACTATTTGGGGAGGTGCATCTTTA
CTCCAAATGTTGCTAGCTTCTATGAATGAACTGTTAAAATCTGATTGGAAGTGGGATTTT
ATTATAAATTTAAGTGAAAGTGATTTTCCAATTAAATCAAATGAAAGACTTGTGCAATTC
TTAACAGCAAATAAAAATAAAAACTTTGTGAAATCACATGGGCGAGAAGTACAAAGATTT
ATTCAGAAGCAAGGCTTAGACAAAACTTTCATTGAATGTGATACGCATATGTGGAGAAC
>Locus_26648_Transcript_1/1_Confidence_1.000_Length_302|Q7KVA1|Xylosylt
ransferase_oxt
TTAACAGCAAATAAAAATAAAAACTTTGTGAAATCACATGGGCGAGAAGTACAAAGATTT
ATTCAGAAGCAAGGCTTAGACAAAACTTTCATTGAATGTGATACGCATATGTGGAGAACT
GGTGATAGAACCTTGCCATGGGGTATTCAAATTGATGGCGGTAGTGATTGGATAACTCTG
ACTAGAGACTTTGTGAAATATATCACACAAGAACAAGATGCCCTTGTCAAGGGCCTAATG
```

ACTGTTTTTAGGCATACTTTATTACCTGCTGAATCATTTTTCCATACAGTTTTACGCAAC
TC

2. Assembly of the transcripts clustered by Q7KVA1.

- Reference protein Q7KVA1:

>sp|Q7KVA1|XYLT_DROME Xylosyltransferase oxt OS=Drosophila melanogaster GN=oxt PE=2 SV=1
MEQSVSARWLKRYRAFFLILLLIVAIQLFLAYKSLDIVGGGSGSGFDAAEAPASPPPPHA
QARVQPPARTKLTAQQLGFQPECDILAREAISALQRAKTKDCREHIAQIACAIQAGRFYA
PQLRSSCPAGNHTANVSLGCFKDEKDRRLLAGYYSSSKTSNSPAKCVELCLQSGYPYAGV
QYGRECFCGYDPPPKASKLPDSSCNTKCLGNAKEICGGFYAMNIYETGIAKFTAQLAATT
PSEETKRVRIAFLLTLNGRALRQVHRLLKALYAPEHVYYIHVDERQDYLYRKLLELESKF
PNIRLARKRFSTIWGGASLLTMLLQCMEDLLQSNWHWDFVINLSESDFPVKTLDKLVDFL
SANPGRNFVKGHGRETQKFIQKQGLDKTFVECDTHMWRIGDRKLPAGIQVDGGSDWVALS
RPFVGYVTHPREDDELLQALLKLFRHTLLPAESFFHTVLRNTKHCTSYVDNNLHVTNWKR
KQGCKCQYKHVVDWCGCSPNDFKPEDWPRLQATEQKSLFFARKFEPVINQAVLLQLEEWL
YGPYTSEYANLHGYWQSLYHHEDVHGSGDDLARSIGDSVMRLSARQAKLYPLELIELTHY
LHRDQYKGFLVRYRARGSTGKPLHLETRVRPTQQGKLARNARFSKRLRNFEVSTDFDQKE
QIARNFGKLLGPQSDLLLSYTLQANADSGAASHSYNLTLLWIDPLGRLQDFNELHVEDST
SDVINHSKTLLKHPITPGIWTAKLIGRNSIYAQLKFLIAPLAYYKGYPLAKSSDAEALNA
GLTVALPEDFEMPVEWQQHLQTDDEQFTMREESLAKGKMLGQELHSWIDGLVGQFFQLRE
SCVVEADSEVSLPLCSDAPWSSLAPDPKSDVDALLK

- Transcripts to be assembled (protein Q7KVA1):

>Locus_4025_Transcript_2/7_Confidence_0.312_Length_368|Q7KVA1|Xylosyltransferase_oxt
AATATGAAATGTCCTGGAGATCCCAAAGAAATTTGTGGTGGCTTTTTTAAAATGAACATA
TATGAAACAGGAATTTCAAAATTCTCACCTCAAACTGCAGTAACTTTAACAACAAATGAT
ACAGAACCTAAAGTACGCATTGTATATTTGCTGACTTTAAATGGACGAGCAGTACGTCAA
GTTTACAGATTAATTAAAGCTCTATTTCATGTAGATCATTATTTTTATATTCACGTAGAC
TCTCGCCAAGATTACATGTTTCGTCAACTTTTGCAATTAGAAAATAACTTTCCAAATATA
CGTCTTTCTCGCAATCGTTTTTCTACTATTTGGGGAGGTGCATCTTTACTCCAAATGTTG
CTAGCTTC
>Locus_18073_Transcript_1/1_Confidence_1.000_Length_299|Q7KVA1|Xylosyltransferase_oxt
TTTCCAAATATACGTCTTTCTCGCAATCGTTTTTCTACTATTTGGGGAGGTGCATCTTTA
CTCCAAATGTTGCTAGCTTCTATGAATGAACTGTTAAAATCTGATTGGAAGTGGGATTTT
ATTATAAATTTAAGTGAAAGTGATTTTCCAATTAAATCAAATGAAAGACTTGTGCAATTC
TTAACAGCAAATAAAAATAAAAACTTTGTGAAATCACATGGGCGAGAAGTACAAAGATTT
ATTCAGAAGCAAGGCTTAGACAAAACTTTCATTGAATGTGATACGCATATGTGGAGAAC
>Locus_26648_Transcript_1/1_Confidence_1.000_Length_302|Q7KVA1|Xylosyltransferase_oxt
TTAACAGCAAATAAAAATAAAAACTTTGTGAAATCACATGGGCGAGAAGTACAAAGATTT
ATTCAGAAGCAAGGCTTAGACAAAACTTTCATTGAATGTGATACGCATATGTGGAGAACT
GGTGATAGAACCTTGCCATGGGGTATTCAAATTGATGGCGGTAGTGATTGGATAACTCTG
ACTAGAGACTTTGTGAAATATATCACACAAGAACAAGATGCCCTTGTCAAGGGCCTAATG
ACTGTTTTTAGGCATACTTTATTACCTGCTGAATCATTTTTCCATACAGTTTTACGCAAC
TC

- Assembly of the three transcripts:

```
>Locus_4025_Transcript_2/7|Locus_18073_Transcript_1/1|Locus_26648_Tranc
ript_1/1|Q7KVA1|Xylosyltransferase_oxt
AATATGAAATGTCCTGGAGATCCCAAAGAAATTTGTGGTGGCTTTTTTAAAATGAACATA
TATGAAACAGGAATTTCAAAATTCTCACCTCAAACTGCAGTAACTTTAACAACAAATGAT
ACAGAACCTAAAGTACGCATTGTATATTTGCTGACTTTAAATGGACGAGCAGTACGTCAA
GTTTACAGATTAATTAAAGCTCTATTTCATGTAGATCATTATTTTTATATTCACGTAGAC
TCTCGCCAAGATTACATGTTTCGTCAACTTTTGCAATTAGAAAATAACTTTCCAAATATA
CGTCTTTCTCGCAATCGTTTTTCTACTATTTGGGGAGGTGCATCTTTACTCCAAATGTTG
CTAGCTTCTATGAATGAACTGTTAAAATCTGATTGGAAGTGGGATTTTATTATAAATTTA
AGTGAAAGTGATTTTCCAATTAAATCAAATGAAAGACTTGTGCAATTCTTAACAGCAAAT
AAAAATAAAAACTTTGTGAAATCACATGGGCGAGAAGTACAAAGATTTATTCAGAAGCAA
GGCTTAGACAAAACTTTCATTGAATGTGATACGCATATGTGGAGAACTGGTGATAGAACC
TTGCCATGGGGTATTCAAATTGATGGCGGTAGTGATTGGATAACTCTGACTAGAGACTTT
GTGAAATATATCACACAAGAACAAGATGCCCTTGTCAAGGGCCTAATGACTGTTTTTAGG
CATACTTTATTACCTGCTGAATCATTTTTCCATACAGTTTTACGCAACTC
```

- Alignment of the protein encoded by assembled transcript to the reference protein Q7KVA1:

```
Query  205   NTKCLGNAKEICGGFYAMNIYETGIAKFTAQLAATTPSEETK-RVRIAFLLTLNGRALRQ  263
             N KC G+ KEICGGF+ MNIYETGI+KF+ Q A T   + +T+ +VRI +LLTLNGRA+RQ
Sbjct  1     NMKCPGDPKEICGGFFKMNIYETGISKFSPQTAVTLTTNDTEPKVRIVYLLTLNGRAVRQ  180

Query  264   VHRLLKALYAPEHVYYIHVDERQDYLYRKLLELESKFPNIRLARKRFSTIWGGASLLTML  323
             V+RL+KAL+   +H +YIHVD RQDY++R+LL+LE+ FPNIRL+R RFSTIWGGASLL ML
Sbjct  181   VYRLIKALFHVDHYFYIHVDSRQDYMFRQLLQLENNFPNIRLSRNRFSTIWGGASLLQML  360

Query  324   LQCMEDLLQSNWHWDFVINLSESDFPVKTLDKLVDFLSANPGRNFVKGHGRETQKFIQKQ  383
             L  M +LL+S+W WDF+INLSESDFP+K+ ++LV FL+AN  +NFVK HGRE Q+FIQKQ
Sbjct  361   LASMNELLKSDWKWDFIINLSESDFPIKSNERLVQFLTANKNKNFVKSHGREVQRFIQKQ  540

Query  384   GLDKTFVECDTHMWRIGDRKLPAGIQVDGGSDWVALSRPFVGYVTHPREDDELLQALLKL  443
             GLDKTF+ECDTHMWR GDR LP GIQ+DGGSDW+ L+R FV Y+T  +E D L++ L+ +
Sbjct  541   GLDKTFIECDTHMWRTGDRTLPWGIQIDGGSDWITLTRDFVKYIT--QEQDALVKGLMTV  714

Query  444   FRHTLLPAESFFHTVLRN  461
             FRHTLLPAESFFHTVLRN
Sbjct  715   FRHTLLPAESFFHTVLRN  768
```

- Translated assembled transcript for protein Q7KVA1:

```
NMKCPGDPKEICGGFFKMNIYETGISKFSPQTAVTLTTNDTEPKVRIVYLLTLNGRAVRQ
VYRLIKALFHVDHYFYIHVDSRQDYMFRQLLQLENNFPNIRLSRNRFSTIWGGASLLQML
```

LASMNELLKSDWKWDFIINLSESDFPIKSNERLVQFLTANKNKNFVKSHGREVQRFIQKQ
GLDKTFIECDTHMWRTGDRTLPWGIQIDGGSDWITLTRDFVKYITQEQDALVKGLMTVFR
HTLLPAESFFHTVLRN

3. Sequence used for synthesis and expression.

ATGAAATGTCCTGGAGATCCCAAAGAAATTTGTGGTGGCTTTTTTAAAATGAACATATATGAAACAGG
AATTTCAAAATTCTCACCTCAAACTGCAGTAACTTTAACAACAAATGATACAGAACCTAAAGTACGCA
TTGTATATTTGCTGACTTTAAATGGACGAGCAGTACGTCAAGTTTACAGATTAATTAAAGCTCTATTT
CATGTAGATCATTATTTTTATATTCACGTAGACTCTCGCCAAGATTACATGTTTCGTCAACTTTTGCA
ATTAGAAAATAACTTTCCAAATATACGTCTTTCTCGCAATCGTTTTTCTACTATTTGGGGAGGTGCAT
CTTTACTCCAAATGTTGCTAGCTTCTATGAATGAACTGTTAAAATCTGATTGGAAGTGGGATTTTATT
ATAAATTTAAGTGAAAGTGATTTTCCAATTAAATCAAATGAAAGACTTGTGCAATTCTTAACAGCAAA
TAAAAATAAAAACTTTGTGAAATCACATGGGCGAGAAGTACAAAGATTTATTCAGAAGCAAGGCTTAG
ACAAAACTTTCATTGAATGTGATACGCATATGTGGAGAACTGGTGATAGAACCTTGCCATGGGGTATT
CAAATTGATGGCGGTAGTGATTGGATAACTCTGACTAGAGACTTTGTGAAATATATCACACAAGAACA
AGATGCCCTTGTCAAGGGCCTAATGACTGTTTTTAGGCATACTTTATTACCTGCTGAATCATTTTTCC
ATACAGTTTTACGCAACTC

De novo sequence assembly for sequence F5: *Drosophila melanogaster* Metal ion transporter Q9VAF0.

1. Protein oriented transcripts for Q9VAF0.

>Locus_4149_Transcript_1/4_Confidence_0.600_Length_1883|Q9VAF0|
CTTTCCTTCCCGTGTCTATGATAGACTAACTCCGACTTCGTATGCGCAGGCTCGACACGA
CGGACGTATTCGCATTCGCACGATCTACGAACCAACTGACATAGTAGTACAGCGGGTGAC
AGTCGTCGTAAAACGTAGAATAATGCGTTACAATTTCGATCTGAAGTGGACAATGACTTC
AATAATTTATAACATCAAAGCTTCTGCACGACCATAAATTGATTTTAATCTTTTAAATGA
TTTCGTTAACGCTAGCTCAATCTCAGTGCAGATTAGCTAGTCGAACGTCGAATCTTCCCA
AGAAAAACGGCTTGTGTTTCTATTGGATTCATTTGTTTTATGGATTTTGATGCAACATAA
ACGGTGTATTGTAAAAACCTGATGATAAAACAAGTATTTTTAAATAATAATTACGTTACG
TTATTTGTGATAGAGAGTGAAAGTAGTGGCAATGAATTCCTCAATTTGGGAGTCAGACTC
AGCCTTCAGATTTATTTACACTGATTTAGTTATGGAATACATACCAGAATATTTCAAAGA
CTGGCAATATCATCCATGGATTTTTTCGGTCTTAGGTTCTATTTTAATTGGATTAAGTGG
TGTGTTGCCTTTACTTATAATTCCATTGGAAGGAGGAGAACATTTTAAAAGTGGAGGAGG
GGGCAAACTTTTGCGCGTATTGCTCAGTTTTGCAGTAGGAGGTCTGCTCGGAGATGTGTT
TTTGCATTTGTTGCCAGAAGCATGGGAGAACTACAGTAACAAAGGAGAGCGACTCGTAGT
CATGTACGAATTCGTAGATTCAGGAGGAGGTAGTCCATCAATGACATGCGGGTGCTGGGT
ATTAGCTGGCCTCTTGTTATTCACGATGGTCGAGAAGTTATTTGCTGGTTACATGGAAGA
TGATGACGTTTCCGAGGAAAAGACCGAACGAAAGCGCAAGAAAATGTCTAAAATTAATTG
TTTGATAAAGAACAACAATGTCTTGAGGGCTAGAAACGAAAATGGACGATTCTCGACCAG
CAAATGCTCAGGCGGACAAAATGGAAAAATGTGTGACATTGAAGTTATAAATGGAGCTAT
CAAAAACGGCTGTTGTATGGTCGAGGAGCTAGAGAAAGAGGCTGTGAGCAACAAGTCTAA
GCATGTTTCTGGATACTTAAATCTCATGGCAAACTCAATTGATAATTTCACACACGGTCT
TGCTGTTGGTGGTTCTTTCATTGTTTCATTCAAACTAGGAATTTTAACAACATTCGCAAT
TCTAGTGCATGAGATTCCACACGAAGTGGGCGATTTTGCAATTTTGTTGCGGTCAGGTTT
CTCGCGGTGGGACGCGGCTCGAGCGCAACTCATGACCGCTGGCGCCGGCCTGCTTGGAGC
TATGACCGCAATCGGCGGAAGTCGGGCTACGAGTGCAATGGAAGCTAGAACATGCTGGAT
AATGCCCTTCACAGCCGGCGGATTTCTCCACATAAGTTTAGTTACAGTGCTACCTGACTT
GCTGCAAGAAAGGGATCCAAAAGAGTCGATTAAACAATTTGGAGCATTGATTGGTGGTAT
AGTTCTAATGGCATCATTGACCACATTATTGGAGTGAGGCATATATTTTTCTATTTCTAA

TTAGATTTCACATATCGGTGCCATCATCACATTAAGTCACTGTGATTTGACTAATATGAT
ACAGACTTGATGAAATAAAGTAAAAATAGGGAACTATTTTACAAAGAAAACTTATTGACA
TAATTAGATTAATCTATTTGATCTGTCCGGGAGAATTACTTACTTACTTACATATTATGG
TTTTGAAAAGTAATTCTCAAATAATGATATTTGTGCATGTAAATAGTTATATTTTTAATA
CTTGTTATATATATATATATATA
>Locus_4149_Transcript_2/4_Confidence_0.700_Length_2043|Q9VAF0|
CTTTCCTTCCCGTGTCTATGATAGACTAACTCCGACTTCGTATGCGCAGGCTCGACACGA
CGGACGTATTCGCATTCGCACGATCTACGAACCAACTGACATAGTAGTACAGCGGGTGAC
AGTCGTCGTAAAACGTAGAATAATGCGTTACAATTTCGATCTGAAGTGGACAATGACTTC
AATAATTTATAACATCAAAGCTTCTGCACGACCATAAATTGATTTTAATCTTTTAAATGA
TTTCGTTAACGCTAGCTCAATCTCAGTGCAGATTAGCTAGTCGAACGTCGAATCTTCCCA
AGAAAAACGGTGCGACTATACGACGTTTGATTTTTTGTTTACTGCGCGTTCTATTGGTCC
TTAAGTGACATTCTCAGATGTTTTTAATGTGATTTTCTTGTGATATAAATGCGTGCTCAT
ATCATTTGTGTTAATTTTGAAATTAACGATTTTTTATTTTCCGTTCCAGGCTTGTGTTTC
TATTGGATTCATTTGTTTTATGGATTTTGATGCAACATAAACGGTGTATTGTAAAAACCT
GATGATAAAACAAGTATTTTTAAATAATAATTACGTTACGTTATTTGTGATAGAGAGTGA
AAGTAGTGGCAATGAATTCCTCAATTTGGGAGTCAGACTCAGCCTTCAGATTTATTTACA
CTGATTTAGTTATGGAATACATACCAGAATATTTCAAAGACTGGCAATATCATCCATGGA
TTTTTTCGGTCTTAGGTTCTATTTTAATTGGATTAAGTGGTGTGTTGCCTTTACTTATAA
TTCCATTGGAAGGAGGAGAACATTTTAAAAGTGGAGGAGGGGGCAAACTTTTGCGCGTAT
TGCTCAGTTTTGCAGTAGGAGGTCTGCTCGGAGATGTGTTTTTGCATTTGTTGCCAGAAG
CATGGGAGAACTACAGTAACAAAGGAGAGCGACTCGTAGTCATGTACGAATTCGTAGATT
CAGGAGGAGGTAGTCCATCAATGACATGCGGGTGCTGGGTATTAGCTGGCCTCTTGTTAT
TCACGATGGTCGAGAAGTTATTTGCTGGTTACATGGAAGATGATGACGTTTCCGAGGAAA
AGACCGAACGAAAGCGCAAGAAAATGTCTAAAATTAATTGTTTGATAAAGAACAACAATG
TCTTGAGGGCTAGAAACGAAAATGGACGATTCTCGACCAGCAAATGCTCAGGCGGACAAA
ATGGAAAAATGTGTGACATTGAAGTTATAAATGGAGCTATCAAAAACGGCTGTTGTATGG
TCGAGGAGCTAGAGAAAGAGGCTGTGAGCAACAAGTCTAAGCATGTTTCTGGATACTTAA
ATCTCATGGCAAACTCAATTGATAATTTCACACACGGTCTTGCTGTTGGTGGTTCTTTCA
TTGTTTCATTCAAACTAGGAATTTTAACAACATTCGCAATTCTAGTGCATGAGATTCCAC
ACGAAGTGGGCGATTTTGCAATTTTGTTGCGGTCAGGTTTCTCGCGGTGGGACGCGGCTC
GAGCGCAACTCATGACCGCTGGCGCCGGCCTGCTTGGAGCTATGACCGCAATCGGCGGAA
GTCGGGCTACGAGTGCAATGGAAGCTAGAACATGCTGGATAATGCCCTTCACAGCCGGCG
GATTTCTCCACATAAGTTTAGTTACAGTGCTACCTGACTTGCTGCAAGAAAGGGATCCAA
AAGAGTCGATTAAACAATTTGGAGCATTGATTGGTGGTATAGTTCTAATGGCATCATTGA
CCACATTATTGGAGTGAGGCATATATTTTTCTATTTCTAATTAGATTTCACATATCGGTG
CCATCATCACATTAAGTCACTGTGATTTGACTAATATGATACAGACTTGATGAAATAAAG
TAAAAATAGGGAACTATTTTACAAAGAAAACTTATTGACATAATTAGATTAATCTATTTG
ATCTGTCCGGGAGAATTACTTACTTACTTACATATTATGGTTTTGAAAAGTAATTCTCAA
ATAATGATATTTGTGCATGTAAATAGTTATATTTTTAATACTTGTTATATATATATATAT
ATA
>Locus_4149_Transcript_3/4_Confidence_0.600_Length_1932|Q9VAF0|
CTTTCCTTCCCGTGTCTATGATAGACTAACTCCGACTTCGTATGCGCAGGCTCGACACGA
CGGACGTATTCGCATTCGCACGATCTACGAACCAACTGACATAGTAGTACAGCGGGTGAC
AGTCGTCGTAAAACGTAGAATAATGCGTTACAATTTCGATCTGAAGTGGACAATGACTTC
AATAATTTATAACATCAAAGCTTCTGCACGACCATAAATTGATTTTAATCTTTTAAATGA
TTTCGTTAACGCTAGCTCAATCTCAGTGCAGATTAGCTAGTCGAACGTCGAATCTTCCCA
AGAAAAACGGTGCGACTATACGACGTTTGATTTTTTGTTTACTGCGCGTTCTATTGGTCC
TTAAGTGACATTCTCAGATGTTTTTAATGCTTGTGTTTCTATTGGATTCATTTGTTTTAT
GGATTTTGATGCAACATAAACGGTGTATTGTAAAAACCTGATGATAAAACAAGTATTTTT
AAATAATAATTACGTTACGTTATTTGTGATAGAGAGTGAAAGTAGTGGCAATGAATTCCT
CAATTTGGGAGTCAGACTCAGCCTTCAGATTTATTTACACTGATTTAGTTATGGAATACA
TACCAGAATATTTCAAAGACTGGCAATATCATCCATGGATTTTTTCGGTCTTAGGTTCTA
TTTTAATTGGATTAAGTGGTGTGTTGCCTTTACTTATAATTCCATTGGAAGGAGGAGAAC
ATTTTAAAAGTGGAGGAGGGGGCAAACTTTTGCGCGTATTGCTCAGTTTTGCAGTAGGAG
GTCTGCTCGGAGATGTGTTTTTGCATTTGTTGCCAGAAGCATGGGAGAACTACAGTAACA
AAGGAGATTCAGGAGGAGGTAGTCCATCAATGACATGCGGGTGCTGGGTATTAGCTGGCC

TCTTGTTATTCACGATGGTCGAGAAGTTATTTGCTGGTTACATGGAAGATGATGACGTTT
CCGAGGAAAAGACCGAACGAAAGCGCAAGAAAATGTCTAAAATTAATTGTTTGATAAAGA
ACAACAATGTCTTGAGGGCTAGAAACGAAAATGGACGATTCTCGACCAGCAAATGCTCAG
GCGGACAAAATGGAAAAATGTGTGACATTGAAGTTATAAATGGAGCTATCAAAAACGGCT
GTTGTATGGTCGAGGAGCTAGAGAAAGAGGCTGTGAGCAACAAGTCTAAGCATGTTTCTG
GATACTTAAATCTCATGGCAAACTCAATTGATAATTTCACACACGGTCTTGCTGTTGGTG
GTTCTTTCATTGTTTCATTCAAACTAGGAATTTTAACAACATTCGCAATTCTAGTGCATG
AGATTCCACACGAAGTGGGCGATTTTGCAATTTTGTTGCGGTCAGGTTTCTCGCGGTGGG
ACGCGGCTCGAGCGCAACTCATGACCGCTGGCGCCGGCCTGCTTGGAGCTATGACCGCAA
TCGGCGGAAGTCGGGCTACGAGTGCAATGGAAGCTAGAACATGCTGGATAATGCCCTTCA
CAGCCGGCGGATTTCTCCACATAAGTTTAGTTACAGTGCTACCTGACTTGCTGCAAGAAA
GGGATCCAAAAGAGTCGATTAAACAATTTGGAGCATTGATTGGTGGTATAGTTCTAATGG
CATCATTGACCACATTATTGGAGTGAGGCATATATTTTTCTATTTCTAATTAGATTTCAC
ATATCGGTGCCATCATCACATTAAGTCACTGTGATTTGACTAATATGATACAGACTTGAT
GAAATAAAGTAAAAATAGGGAACTATTTTACAAAGAAAACTTATTGACATAATTAGATTA
ATCTATTTGATCTGTCCGGGAGAATTACTTACTTACTTACATATTATGGTTTTGAAAAGT
AATTCTCAAATAATGATATTTGTGCATGTAAATAGTTATATTTTTAATACTTGTTATATA
TATATATATATA
>Locus_4149_Transcript_4/4_Confidence_0.600_Length_1962|Q9VAF0|
CTTTCCTTCCCGTGTCTATGATAGACTAACTCCGACTTCGTATGCGCAGGCTCGACACGA
CGGACGTATTCGCATTCGCACGATCTACGAACCAACTGACATAGTAGTACAGCGGGTGAC
AGTCGTCGTAAAACGTAGAATAATGCGTTACAATTTCGATCTGAAGTGGACAATGACTTC
AATAATTTATAACATCAAAGCTTCTGCACGACCATAAATTGATTTTAATCTTTTAAATGA
TTTCGTTAACGCTAGCTCAATCTCAGTGCAGATTAGCTAGTCGAACGTCGAATCTTCCCA
AGAAAAACGGTGCGACTATACGACGTTTGATTTTTTGTTTACTGCGCGTTCTATTGGTCC
TTAAGTGACATTCTCAGATGTTTTTAATGCTTGTGTTTCTATTGGATTCATTTGTTTTAT
GGATTTTGATGCAACATAAACGGTGTATTGTAAAAACCTGATGATAAAACAAGTATTTTT
AAATAATAATTACGTTACGTTATTTGTGATAGAGAGTGAAAGTAGTGGCAATGAATTCCT
CAATTTGGGAGTCAGACTCAGCCTTCAGATTTATTTACACTGATTTAGTTATGGAATACA
TACCAGAATATTTCAAAGACTGGCAATATCATCCATGGATTTTTTCGGTCTTAGGTTCTA
TTTTAATTGGATTAAGTGGTGTGTTGCCTTTACTTATAATTCCATTGGAAGGAGGAGAAC
ATTTTAAAAGTGGAGGAGGGGGCAAACTTTTGCGCGTATTGCTCAGTTTTGCAGTAGGAG
GTCTGCTCGGAGATGTGTTTTTGCATTTGTTGCCAGAAGCATGGGAGAACTACAGTAACA
AAGGAGAGCGACTCGTAGTCATGTACGAATTCGTAGATTCAGGAGGAGGTAGTCCATCAA
TGACATGCGGGTGCTGGGTATTAGCTGGCCTCTTGTTATTCACGATGGTCGAGAAGTTAT
TTGCTGGTTACATGGAAGATGATGACGTTTCCGAGGAAAAGACCGAACGAAAGCGCAAGA
AAATGTCTAAAATTAATTGTTTGATAAAGAACAACAATGTCTTGAGGGCTAGAAACGAAA
ATGGACGATTCTCGACCAGCAAATGCTCAGGCGGACAAAATGGAAAAATGTGTGACATTG
AAGTTATAAATGGAGCTATCAAAAACGGCTGTTGTATGGTCGAGGAGCTAGAGAAAGAGG
CTGTGAGCAACAAGTCTAAGCATGTTTCTGGATACTTAAATCTCATGGCAAACTCAATTG
ATAATTTCACACACGGTCTTGCTGTTGGTGGTTCTTTCATTGTTTCATTCAAACTAGGAA
TTTTAACAACATTCGCAATTCTAGTGCATGAGATTCCACACGAAGTGGGCGATTTTGCAA
TTTTGTTGCGGTCAGGTTTCTCGCGGTGGGACGCGGCTCGAGCGCAACTCATGACCGCTG
GCGCCGGCCTGCTTGGAGCTATGACCGCAATCGGCGGAAGTCGGGCTACGAGTGCAATGG
AAGCTAGAACATGCTGGATAATGCCCTTCACAGCCGGCGGATTTCTCCACATAAGTTTAG
TTACAGTGCTACCTGACTTGCTGCAAGAAAGGGATCCAAAAGAGTCGATTAAACAATTTG
GAGCATTGATTGGTGGTATAGTTCTAATGGCATCATTGACCACATTATTGGAGTGAGGCA
TATATTTTTCTATTTCTAATTAGATTTCACATATCGGTGCCATCATCACATTAAGTCACT
GTGATTTGACTAATATGATACAGACTTGATGAAATAAAGTAAAAATAGGGAACTATTTTA
CAAAGAAAACTTATTGACATAATTAGATTAATCTATTTGATCTGTCCGGGAGAATTACTT
ACTTACTTACATATTATGGTTTTGAAAAGTAATTCTCAAATAATGATATTTGTGCATGTA
AATAGTTATATTTTTAATACTTGTTATATATATATATATATA

2. Assembly of the transcripts clustered by Q9VAF0.

-   Reference protein Q9VAF0:

```
>sp|Q9VAF0|Y816_DROME Uncharacterized protein CG7816 OS=Drosophila
melanogaster GN=CG7816 PE=2 SV=1
MTTNSSFFDEHIAMIYSNLMDQYMPEYFKSFEYTPWVFSLLGSVVIGLSGIFPLIIIPTE
EKMAKEGYKDPADSKLLRVLLSFAVGGLLGDVFLHLLPEAWEGDNQDPSSHPSLRSGLWV
LSGILIFTIVEKIFSGYASADEENPQPKCVEIANCLLRRHGGQLPEGETSESCGGACDIE
DVGKVCFLREQEQKSKERKEQPKKVAGYLNLLANSIDNFTHGLAVAGSFLVSFRHGILAT
FAILLHEIPHEVGDFAILLRSGFSRWDAARAQLLTAGAGLLGALVAIGGSGVTSAMEART
SWIMPFTAGGFLHIALVTVLPDLLKEEERKESIKQLLALVFGIALMAVMTMLFEH
```

-   Transcripts to be assembled (protein Q9VAF0):

The four transcripts are very similar with some indels that could correspond to different isoforms. This is the alignment of all the transcripts clustered by the protein Q9VAF0:

```
CLUSTAL 2.1 multiple sequence alignment


Locus_4149_Transcript_3/4_Conf      CTTTCCTTCCCGTGTCTATGATAGACTAACTCCGACTTCGTATGCGCAGG
Locus_4149_Transcript_4/4_Conf      CTTTCCTTCCCGTGTCTATGATAGACTAACTCCGACTTCGTATGCGCAGG
Locus_4149_Transcript_1/4_Conf      CTTTCCTTCCCGTGTCTATGATAGACTAACTCCGACTTCGTATGCGCAGG
Locus_4149_Transcript_2/4_Conf      CTTTCCTTCCCGTGTCTATGATAGACTAACTCCGACTTCGTATGCGCAGG
                                    **************************************************

Locus_4149_Transcript_3/4_Conf      CTCGACACGACGGACGTATTCGCATTCGCACGATCTACGAACCAACTGAC
Locus_4149_Transcript_4/4_Conf      CTCGACACGACGGACGTATTCGCATTCGCACGATCTACGAACCAACTGAC
Locus_4149_Transcript_1/4_Conf      CTCGACACGACGGACGTATTCGCATTCGCACGATCTACGAACCAACTGAC
Locus_4149_Transcript_2/4_Conf      CTCGACACGACGGACGTATTCGCATTCGCACGATCTACGAACCAACTGAC
                                    **************************************************

Locus_4149_Transcript_3/4_Conf      ATAGTAGTACAGCGGGTGACAGTCGTCGTAAAACGTAGAATAATGCGTTA
Locus_4149_Transcript_4/4_Conf      ATAGTAGTACAGCGGGTGACAGTCGTCGTAAAACGTAGAATAATGCGTTA
Locus_4149_Transcript_1/4_Conf      ATAGTAGTACAGCGGGTGACAGTCGTCGTAAAACGTAGAATAATGCGTTA
Locus_4149_Transcript_2/4_Conf      ATAGTAGTACAGCGGGTGACAGTCGTCGTAAAACGTAGAATAATGCGTTA
                                    **************************************************

Locus_4149_Transcript_3/4_Conf      CAATTTCGATCTGAAGTGGACAATGACTTCAATAATTTATAACATCAAAG
Locus_4149_Transcript_4/4_Conf      CAATTTCGATCTGAAGTGGACAATGACTTCAATAATTTATAACATCAAAG
Locus_4149_Transcript_1/4_Conf      CAATTTCGATCTGAAGTGGACAATGACTTCAATAATTTATAACATCAAAG
Locus_4149_Transcript_2/4_Conf      CAATTTCGATCTGAAGTGGACAATGACTTCAATAATTTATAACATCAAAG
                                    **************************************************

Locus_4149_Transcript_3/4_Conf      CTTCTGCACGACCATAAATTGATTTTAATCTTTTAAATGATTTCGTTAAC
Locus_4149_Transcript_4/4_Conf      CTTCTGCACGACCATAAATTGATTTTAATCTTTTAAATGATTTCGTTAAC
Locus_4149_Transcript_1/4_Conf      CTTCTGCACGACCATAAATTGATTTTAATCTTTTAAATGATTTCGTTAAC
Locus_4149_Transcript_2/4_Conf      CTTCTGCACGACCATAAATTGATTTTAATCTTTTAAATGATTTCGTTAAC
                                    **************************************************

Locus_4149_Transcript_3/4_Conf      GCTAGCTCAATCTCAGTGCAGATTAGCTAGTCGAACGTCGAATCTTCCCA
Locus_4149_Transcript_4/4_Conf      GCTAGCTCAATCTCAGTGCAGATTAGCTAGTCGAACGTCGAATCTTCCCA
Locus_4149_Transcript_1/4_Conf      GCTAGCTCAATCTCAGTGCAGATTAGCTAGTCGAACGTCGAATCTTCCCA
Locus_4149_Transcript_2/4_Conf      GCTAGCTCAATCTCAGTGCAGATTAGCTAGTCGAACGTCGAATCTTCCCA
                                    **************************************************

Locus_4149_Transcript_3/4_Conf      AGAAAAACGGTGCGACTATACGACGTTTGATTTTTTGTTTACTGCGCGTT
Locus_4149_Transcript_4/4_Conf      AGAAAAACGGTGCGACTATACGACGTTTGATTTTTTGTTTACTGCGCGTT
Locus_4149_Transcript_1/4_Conf      AGAAAAACG-----------------------------------------
Locus_4149_Transcript_2/4_Conf      AGAAAAACGGTGCGACTATACGACGTTTGATTTTTTGTTTACTGCGCGTT
                                    *********

Locus_4149_Transcript_3/4_Conf      CTATTGGTCCTTAAGTGACATTCTCAGATGTTTTTAATG-----------
Locus_4149_Transcript_4/4_Conf      CTATTGGTCCTTAAGTGACATTCTCAGATGTTTTTAATG-----------
```

```
Locus_4149_Transcript_1/4_Conf      --------------------------------------------------
Locus_4149_Transcript_2/4_Conf      CTATTGGTCCTTAAGTGACATTCTCAGATGTTTTTAATGTGATTTTCTTG

Locus_4149_Transcript_3/4_Conf      --------------------------------------------------
Locus_4149_Transcript_4/4_Conf      --------------------------------------------------
Locus_4149_Transcript_1/4_Conf      --------------------------------------------------
Locus_4149_Transcript_2/4_Conf      TGATATAAATGCGTGCTCATATCATTTGTGTTAATTTTGAAATTAACGAT

Locus_4149_Transcript_3/4_Conf      --------------------CTTGTGTTTCTATTGGATTCATTTGTTTTA
Locus_4149_Transcript_4/4_Conf      --------------------CTTGTGTTTCTATTGGATTCATTTGTTTTA
Locus_4149_Transcript_1/4_Conf      -------------------GCTTGTGTTTCTATTGGATTCATTTGTTTTA
Locus_4149_Transcript_2/4_Conf      TTTTTATTTTCCGTTCCAGGCTTGTGTTTCTATTGGATTCATTTGTTTTA
                                                        ******************************

Locus_4149_Transcript_3/4_Conf      TGGATTTTGATGCAACATAAACGGTGTATTGTAAAAACCTGATGATAAAA
Locus_4149_Transcript_4/4_Conf      TGGATTTTGATGCAACATAAACGGTGTATTGTAAAAACCTGATGATAAAA
Locus_4149_Transcript_1/4_Conf      TGGATTTTGATGCAACATAAACGGTGTATTGTAAAAACCTGATGATAAAA
Locus_4149_Transcript_2/4_Conf      TGGATTTTGATGCAACATAAACGGTGTATTGTAAAAACCTGATGATAAAA
                                    **************************************************

Locus_4149_Transcript_3/4_Conf      CAAGTATTTTTAAATAATAATTACGTTACGTTATTTGTGATAGAGAGTGA
Locus_4149_Transcript_4/4_Conf      CAAGTATTTTTAAATAATAATTACGTTACGTTATTTGTGATAGAGAGTGA
Locus_4149_Transcript_1/4_Conf      CAAGTATTTTTAAATAATAATTACGTTACGTTATTTGTGATAGAGAGTGA
Locus_4149_Transcript_2/4_Conf      CAAGTATTTTTAAATAATAATTACGTTACGTTATTTGTGATAGAGAGTGA
                                    **************************************************

Locus_4149_Transcript_3/4_Conf      AAGTAGTGGCAATGAATTCCTCAATTTGGGAGTCAGACTCAGCCTTCAGA
Locus_4149_Transcript_4/4_Conf      AAGTAGTGGCAATGAATTCCTCAATTTGGGAGTCAGACTCAGCCTTCAGA
Locus_4149_Transcript_1/4_Conf      AAGTAGTGGCAATGAATTCCTCAATTTGGGAGTCAGACTCAGCCTTCAGA
Locus_4149_Transcript_2/4_Conf      AAGTAGTGGCAATGAATTCCTCAATTTGGGAGTCAGACTCAGCCTTCAGA
                                    **************************************************

Locus_4149_Transcript_3/4_Conf      TTTATTTACACTGATTTAGTTATGGAATACATACCAGAATATTTCAAAGA
Locus_4149_Transcript_4/4_Conf      TTTATTTACACTGATTTAGTTATGGAATACATACCAGAATATTTCAAAGA
Locus_4149_Transcript_1/4_Conf      TTTATTTACACTGATTTAGTTATGGAATACATACCAGAATATTTCAAAGA
Locus_4149_Transcript_2/4_Conf      TTTATTTACACTGATTTAGTTATGGAATACATACCAGAATATTTCAAAGA
                                    **************************************************

Locus_4149_Transcript_3/4_Conf      CTGGCAATATCATCCATGGATTTTTTCGGTCTTAGGTTCTATTTTAATTG
Locus_4149_Transcript_4/4_Conf      CTGGCAATATCATCCATGGATTTTTTCGGTCTTAGGTTCTATTTTAATTG
Locus_4149_Transcript_1/4_Conf      CTGGCAATATCATCCATGGATTTTTTCGGTCTTAGGTTCTATTTTAATTG
Locus_4149_Transcript_2/4_Conf      CTGGCAATATCATCCATGGATTTTTTCGGTCTTAGGTTCTATTTTAATTG
                                    **************************************************

Locus_4149_Transcript_3/4_Conf      GATTAAGTGGTGTGTTGCCTTTACTTATAATTCCATTGGAAGGAGGAGAA
Locus_4149_Transcript_4/4_Conf      GATTAAGTGGTGTGTTGCCTTTACTTATAATTCCATTGGAAGGAGGAGAA
Locus_4149_Transcript_1/4_Conf      GATTAAGTGGTGTGTTGCCTTTACTTATAATTCCATTGGAAGGAGGAGAA
Locus_4149_Transcript_2/4_Conf      GATTAAGTGGTGTGTTGCCTTTACTTATAATTCCATTGGAAGGAGGAGAA
                                    **************************************************

Locus_4149_Transcript_3/4_Conf      CATTTTAAAAGTGGAGGAGGGGGCAAACTTTTGCGCGTATTGCTCAGTTT
Locus_4149_Transcript_4/4_Conf      CATTTTAAAAGTGGAGGAGGGGGCAAACTTTTGCGCGTATTGCTCAGTTT
Locus_4149_Transcript_1/4_Conf      CATTTTAAAAGTGGAGGAGGGGGCAAACTTTTGCGCGTATTGCTCAGTTT
Locus_4149_Transcript_2/4_Conf      CATTTTAAAAGTGGAGGAGGGGGCAAACTTTTGCGCGTATTGCTCAGTTT
                                    **************************************************

Locus_4149_Transcript_3/4_Conf      TGCAGTAGGAGGTCTGCTCGGAGATGTGTTTTTGCATTTGTTGCCAGAAG
Locus_4149_Transcript_4/4_Conf      TGCAGTAGGAGGTCTGCTCGGAGATGTGTTTTTGCATTTGTTGCCAGAAG
Locus_4149_Transcript_1/4_Conf      TGCAGTAGGAGGTCTGCTCGGAGATGTGTTTTTGCATTTGTTGCCAGAAG
Locus_4149_Transcript_2/4_Conf      TGCAGTAGGAGGTCTGCTCGGAGATGTGTTTTTGCATTTGTTGCCAGAAG
                                    **************************************************

Locus_4149_Transcript_3/4_Conf      CATGGGAGAACTACAGTAACAAAG--------------------------
Locus_4149_Transcript_4/4_Conf      CATGGGAGAACTACAGTAACAAAGGAGAGCGACTCGTAGTCATGTACGAA
Locus_4149_Transcript_1/4_Conf      CATGGGAGAACTACAGTAACAAAGGAGAGCGACTCGTAGTCATGTACGAA
Locus_4149_Transcript_2/4_Conf      CATGGGAGAACTACAGTAACAAAGGAGAGCGACTCGTAGTCATGTACGAA
                                    ************************

Locus_4149_Transcript_3/4_Conf      ----GAGATTCAGGAGGAGGTAGTCCATCAATGACATGCGGGTGCTGGGT
```

```
Locus_4149_Transcript_4/4_Conf      TTCGTAGATTCAGGAGGAGGTAGTCCATCAATGACATGCGGGTGCTGGGT
Locus_4149_Transcript_1/4_Conf      TTCGTAGATTCAGGAGGAGGTAGTCCATCAATGACATGCGGGTGCTGGGT
Locus_4149_Transcript_2/4_Conf      TTCGTAGATTCAGGAGGAGGTAGTCCATCAATGACATGCGGGTGCTGGGT
                                         *********************************************

Locus_4149_Transcript_3/4_Conf      ATTAGCTGGCCTCTTGTTATTCACGATGGTCGAGAAGTTATTTGCTGGTT
Locus_4149_Transcript_4/4_Conf      ATTAGCTGGCCTCTTGTTATTCACGATGGTCGAGAAGTTATTTGCTGGTT
Locus_4149_Transcript_1/4_Conf      ATTAGCTGGCCTCTTGTTATTCACGATGGTCGAGAAGTTATTTGCTGGTT
Locus_4149_Transcript_2/4_Conf      ATTAGCTGGCCTCTTGTTATTCACGATGGTCGAGAAGTTATTTGCTGGTT
                                    **************************************************

Locus_4149_Transcript_3/4_Conf      ACATGGAAGATGATGACGTTTCCGAGGAAAAGACCGAACGAAAGCGCAAG
Locus_4149_Transcript_4/4_Conf      ACATGGAAGATGATGACGTTTCCGAGGAAAAGACCGAACGAAAGCGCAAG
Locus_4149_Transcript_1/4_Conf      ACATGGAAGATGATGACGTTTCCGAGGAAAAGACCGAACGAAAGCGCAAG
Locus_4149_Transcript_2/4_Conf      ACATGGAAGATGATGACGTTTCCGAGGAAAAGACCGAACGAAAGCGCAAG
                                    **************************************************

Locus_4149_Transcript_3/4_Conf      AAAATGTCTAAAATTAATTGTTTGATAAAGAACAACAATGTCTTGAGGGC
Locus_4149_Transcript_4/4_Conf      AAAATGTCTAAAATTAATTGTTTGATAAAGAACAACAATGTCTTGAGGGC
Locus_4149_Transcript_1/4_Conf      AAAATGTCTAAAATTAATTGTTTGATAAAGAACAACAATGTCTTGAGGGC
Locus_4149_Transcript_2/4_Conf      AAAATGTCTAAAATTAATTGTTTGATAAAGAACAACAATGTCTTGAGGGC
                                    **************************************************

Locus_4149_Transcript_3/4_Conf      TAGAAACGAAAATGGACGATTCTCGACCAGCAAATGCTCAGGCGGACAAA
Locus_4149_Transcript_4/4_Conf      TAGAAACGAAAATGGACGATTCTCGACCAGCAAATGCTCAGGCGGACAAA
Locus_4149_Transcript_1/4_Conf      TAGAAACGAAAATGGACGATTCTCGACCAGCAAATGCTCAGGCGGACAAA
Locus_4149_Transcript_2/4_Conf      TAGAAACGAAAATGGACGATTCTCGACCAGCAAATGCTCAGGCGGACAAA
                                    **************************************************

Locus_4149_Transcript_3/4_Conf      ATGGAAAAATGTGTGACATTGAAGTTATAAATGGAGCTATCAAAAACGGC
Locus_4149_Transcript_4/4_Conf      ATGGAAAAATGTGTGACATTGAAGTTATAAATGGAGCTATCAAAAACGGC
Locus_4149_Transcript_1/4_Conf      ATGGAAAAATGTGTGACATTGAAGTTATAAATGGAGCTATCAAAAACGGC
Locus_4149_Transcript_2/4_Conf      ATGGAAAAATGTGTGACATTGAAGTTATAAATGGAGCTATCAAAAACGGC
                                    **************************************************

Locus_4149_Transcript_3/4_Conf      TGTTGTATGGTCGAGGAGCTAGAGAAAGAGGCTGTGAGCAACAAGTCTAA
Locus_4149_Transcript_4/4_Conf      TGTTGTATGGTCGAGGAGCTAGAGAAAGAGGCTGTGAGCAACAAGTCTAA
Locus_4149_Transcript_1/4_Conf      TGTTGTATGGTCGAGGAGCTAGAGAAAGAGGCTGTGAGCAACAAGTCTAA
Locus_4149_Transcript_2/4_Conf      TGTTGTATGGTCGAGGAGCTAGAGAAAGAGGCTGTGAGCAACAAGTCTAA
                                    **************************************************

Locus_4149_Transcript_3/4_Conf      GCATGTTTCTGGATACTTAAATCTCATGGCAAACTCAATTGATAATTTCA
Locus_4149_Transcript_4/4_Conf      GCATGTTTCTGGATACTTAAATCTCATGGCAAACTCAATTGATAATTTCA
Locus_4149_Transcript_1/4_Conf      GCATGTTTCTGGATACTTAAATCTCATGGCAAACTCAATTGATAATTTCA
Locus_4149_Transcript_2/4_Conf      GCATGTTTCTGGATACTTAAATCTCATGGCAAACTCAATTGATAATTTCA
                                    **************************************************

Locus_4149_Transcript_3/4_Conf      CACACGGTCTTGCTGTTGGTGGTTCTTTCATTGTTTCATTCAAACTAGGA
Locus_4149_Transcript_4/4_Conf      CACACGGTCTTGCTGTTGGTGGTTCTTTCATTGTTTCATTCAAACTAGGA
Locus_4149_Transcript_1/4_Conf      CACACGGTCTTGCTGTTGGTGGTTCTTTCATTGTTTCATTCAAACTAGGA
Locus_4149_Transcript_2/4_Conf      CACACGGTCTTGCTGTTGGTGGTTCTTTCATTGTTTCATTCAAACTAGGA
                                    **************************************************

Locus_4149_Transcript_3/4_Conf      ATTTTAACAACATTCGCAATTCTAGTGCATGAGATTCCACACGAAGTGGG
Locus_4149_Transcript_4/4_Conf      ATTTTAACAACATTCGCAATTCTAGTGCATGAGATTCCACACGAAGTGGG
Locus_4149_Transcript_1/4_Conf      ATTTTAACAACATTCGCAATTCTAGTGCATGAGATTCCACACGAAGTGGG
Locus_4149_Transcript_2/4_Conf      ATTTTAACAACATTCGCAATTCTAGTGCATGAGATTCCACACGAAGTGGG
                                    **************************************************

Locus_4149_Transcript_3/4_Conf      CGATTTTGCAATTTTGTTGCGGTCAGGTTTCTCGCGGTGGGACGCGGCTC
Locus_4149_Transcript_4/4_Conf      CGATTTTGCAATTTTGTTGCGGTCAGGTTTCTCGCGGTGGGACGCGGCTC
Locus_4149_Transcript_1/4_Conf      CGATTTTGCAATTTTGTTGCGGTCAGGTTTCTCGCGGTGGGACGCGGCTC
Locus_4149_Transcript_2/4_Conf      CGATTTTGCAATTTTGTTGCGGTCAGGTTTCTCGCGGTGGGACGCGGCTC
                                    **************************************************

Locus_4149_Transcript_3/4_Conf      GAGCGCAACTCATGACCGCTGGCGCCGGCCTGCTTGGAGCTATGACCGCA
Locus_4149_Transcript_4/4_Conf      GAGCGCAACTCATGACCGCTGGCGCCGGCCTGCTTGGAGCTATGACCGCA
Locus_4149_Transcript_1/4_Conf      GAGCGCAACTCATGACCGCTGGCGCCGGCCTGCTTGGAGCTATGACCGCA
Locus_4149_Transcript_2/4_Conf      GAGCGCAACTCATGACCGCTGGCGCCGGCCTGCTTGGAGCTATGACCGCA
                                    **************************************************
```

```
Locus_4149_Transcript_3/4_Conf      ATCGGCGGAAGTCGGGCTACGAGTGCAATGGAAGCTAGAACATGCTGGAT
Locus_4149_Transcript_4/4_Conf      ATCGGCGGAAGTCGGGCTACGAGTGCAATGGAAGCTAGAACATGCTGGAT
Locus_4149_Transcript_1/4_Conf      ATCGGCGGAAGTCGGGCTACGAGTGCAATGGAAGCTAGAACATGCTGGAT
Locus_4149_Transcript_2/4_Conf      ATCGGCGGAAGTCGGGCTACGAGTGCAATGGAAGCTAGAACATGCTGGAT
                                    **************************************************

Locus_4149_Transcript_3/4_Conf      AATGCCCTTCACAGCCGGCGGATTTCTCCACATAAGTTTAGTTACAGTGC
Locus_4149_Transcript_4/4_Conf      AATGCCCTTCACAGCCGGCGGATTTCTCCACATAAGTTTAGTTACAGTGC
Locus_4149_Transcript_1/4_Conf      AATGCCCTTCACAGCCGGCGGATTTCTCCACATAAGTTTAGTTACAGTGC
Locus_4149_Transcript_2/4_Conf      AATGCCCTTCACAGCCGGCGGATTTCTCCACATAAGTTTAGTTACAGTGC
                                    **************************************************

Locus_4149_Transcript_3/4_Conf      TACCTGACTTGCTGCAAGAAAGGGATCCAAAAGAGTCGATTAAACAATTT
Locus_4149_Transcript_4/4_Conf      TACCTGACTTGCTGCAAGAAAGGGATCCAAAAGAGTCGATTAAACAATTT
Locus_4149_Transcript_1/4_Conf      TACCTGACTTGCTGCAAGAAAGGGATCCAAAAGAGTCGATTAAACAATTT
Locus_4149_Transcript_2/4_Conf      TACCTGACTTGCTGCAAGAAAGGGATCCAAAAGAGTCGATTAAACAATTT
                                    **************************************************

Locus_4149_Transcript_3/4_Conf      GGAGCATTGATTGGTGGTATAGTTCTAATGGCATCATTGACCACATTATT
Locus_4149_Transcript_4/4_Conf      GGAGCATTGATTGGTGGTATAGTTCTAATGGCATCATTGACCACATTATT
Locus_4149_Transcript_1/4_Conf      GGAGCATTGATTGGTGGTATAGTTCTAATGGCATCATTGACCACATTATT
Locus_4149_Transcript_2/4_Conf      GGAGCATTGATTGGTGGTATAGTTCTAATGGCATCATTGACCACATTATT
                                    **************************************************

Locus_4149_Transcript_3/4_Conf      GGAGTGAGGCATATATTTTTCTATTTCTAATTAGATTTCACATATCGGTG
Locus_4149_Transcript_4/4_Conf      GGAGTGAGGCATATATTTTTCTATTTCTAATTAGATTTCACATATCGGTG
Locus_4149_Transcript_1/4_Conf      GGAGTGAGGCATATATTTTTCTATTTCTAATTAGATTTCACATATCGGTG
Locus_4149_Transcript_2/4_Conf      GGAGTGAGGCATATATTTTTCTATTTCTAATTAGATTTCACATATCGGTG
                                    **************************************************

Locus_4149_Transcript_3/4_Conf      CCATCATCACATTAAGTCACTGTGATTTGACTAATATGATACAGACTTGA
Locus_4149_Transcript_4/4_Conf      CCATCATCACATTAAGTCACTGTGATTTGACTAATATGATACAGACTTGA
Locus_4149_Transcript_1/4_Conf      CCATCATCACATTAAGTCACTGTGATTTGACTAATATGATACAGACTTGA
Locus_4149_Transcript_2/4_Conf      CCATCATCACATTAAGTCACTGTGATTTGACTAATATGATACAGACTTGA
                                    **************************************************

Locus_4149_Transcript_3/4_Conf      TGAAATAAAGTAAAAATAGGGAACTATTTTACAAAGAAAACTTATTGACA
Locus_4149_Transcript_4/4_Conf      TGAAATAAAGTAAAAATAGGGAACTATTTTACAAAGAAAACTTATTGACA
Locus_4149_Transcript_1/4_Conf      TGAAATAAAGTAAAAATAGGGAACTATTTTACAAAGAAAACTTATTGACA
Locus_4149_Transcript_2/4_Conf      TGAAATAAAGTAAAAATAGGGAACTATTTTACAAAGAAAACTTATTGACA
                                    **************************************************

Locus_4149_Transcript_3/4_Conf      TAATTAGATTAATCTATTTGATCTGTCCGGGAGAATTACTTACTTACTTA
Locus_4149_Transcript_4/4_Conf      TAATTAGATTAATCTATTTGATCTGTCCGGGAGAATTACTTACTTACTTA
Locus_4149_Transcript_1/4_Conf      TAATTAGATTAATCTATTTGATCTGTCCGGGAGAATTACTTACTTACTTA
Locus_4149_Transcript_2/4_Conf      TAATTAGATTAATCTATTTGATCTGTCCGGGAGAATTACTTACTTACTTA
                                    **************************************************

Locus_4149_Transcript_3/4_Conf      CATATTATGGTTTTGAAAAGTAATTCTCAAATAATGATATTTGTGCATGT
Locus_4149_Transcript_4/4_Conf      CATATTATGGTTTTGAAAAGTAATTCTCAAATAATGATATTTGTGCATGT
Locus_4149_Transcript_1/4_Conf      CATATTATGGTTTTGAAAAGTAATTCTCAAATAATGATATTTGTGCATGT
Locus_4149_Transcript_2/4_Conf      CATATTATGGTTTTGAAAAGTAATTCTCAAATAATGATATTTGTGCATGT
                                    **************************************************

Locus_4149_Transcript_3/4_Conf      AAATAGTTATATTTTTAATACTTGTTATATATATATATATATA
Locus_4149_Transcript_4/4_Conf      AAATAGTTATATTTTTAATACTTGTTATATATATATATATATA
Locus_4149_Transcript_1/4_Conf      AAATAGTTATATTTTTAATACTTGTTATATATATATATATATA
Locus_4149_Transcript_2/4_Conf      AAATAGTTATATTTTTAATACTTGTTATATATATATATATATA
                                    *******************************************
```

-Translation of the largest contig, Locus_4149_Transcript_2/4_Confidence_0.700_Length_2043|Q9VAF0:

```
atgaattcctcaatttgggagtcagactcagccttcagatttatttacact
 M  N  S  S  I  W  E  S  D  S  A  F  R  F  I  Y  T
gatttagttatggaatacataccagaatatttcaaagactggcaatatcatccatggat
```

```
t
 D  L  V  M  E  Y  I  P  E  Y  F  K  D  W  Q  Y  H  P  W  I
ttttcggtcttaggttctattttaattggattaagtggtgtgttgcctttacttataat
t
 F  S  V  L  G  S  I  L  I  G  L  S  G  V  L  P  L  L  I  I
ccattggaaggaggagaacattttaaaagtggaggagggggcaaacttttgcgcgtatt
g
 P  L  E  G  G  E  H  F  K  S  G  G  G  G  K  L  L  R  V  L
ctcagttttgcagtaggaggtctgctcggagatgtgtttttgcatttgttgccagaagc
a
 L  S  F  A  V  G  G  L  L  G  D  V  F  L  H  L  L  P  E  A
tgggagaactacagtaacaaaggagagcgactcgtagtcatgtacgaattcgtagattc
a
 W  E  N  Y  S  N  K  G  E  R  L  V  V  M  Y  E  F  V  D  S
ggaggaggtagtccatcaatgacatgcgggtgctgggtattagctggcctcttgttatt
c
 G  G  G  S  P  S  M  T  C  G  C  W  V  L  A  G  L  L  L  F
acgatggtcgagaagttatttgctggttacatggaagatgatgacgtttccgaggaaaa
g
 T  M  V  E  K  L  F  A  G  Y  M  E  D  D  D  V  S  E  E  K
accgaacgaaagcgcaagaaaatgtctaaaattaattgtttgataaagaacaacaatgt
c
 T  E  R  K  R  K  K  M  S  K  I  N  C  L  I  K  N  N  N  V
ttgagggctagaaacgaaaatggacgattctcgaccagcaaatgctcaggcggacaaaa
t
 L  R  A  R  N  E  N  G  R  F  S  T  S  K  C  S  G  G  Q  N
ggaaaaatgtgtgacattgaagttataaatggagctatcaaaaacggctgttgtatggt
c
 G  K  M  C  D  I  E  V  I  N  G  A  I  K  N  G  C  C  M  V
gaggagctagagaaagaggctgtgagcaacaagtctaagcatgtttctggatacttaaa
t
 E  E  L  E  K  E  A  V  S  N  K  S  K  H  V  S  G  Y  L  N
ctcatggcaaactcaattgataatttcacacacggtcttgctgttggtggttctttcat
t
 L  M  A  N  S  I  D  N  F  T  H  G  L  A  V  G  G  S  F  I
gtttcattcaaactaggaattttaacaacattcgcaattctagtgcatgagattccaca
c
 V  S  F  K  L  G  I  L  T  T  F  A  I  L  V  H  E  I  P  H
gaagtgggcgattttgcaattttgttgcggtcaggtttctcgcggtgggacgcggctcg
a
 E  V  G  D  F  A  I  L  L  R  S  G  F  S  R  W  D  A  A  R
gcgcaactcatgaccgctggcgccggcctgcttggagctatgaccgcaatcggcggaag
t
 A  Q  L  M  T  A  G  A  G  L  L  G  A  M  T  A  I  G  G  S
cgggctacgagtgcaatggaagctagaacatgctggataatgcccttcacagccggcgg
a
 R  A  T  S  A  M  E  A  R  T  C  W  I  M  P  F  T  A  G  G
```

```
tttctccacataagtttagttacagtgctacctgacttgctgcaagaaagggatccaaa
a
 F  L  H  I  S  L  V  T  V  L  P  D  L  L  Q  E  R  D  P  K
gagtcgattaaacaatttggagcattgattggtggtatagttctaatggcatcattgac
c
 E  S  I  K  Q  F  G  A  L  I  G  G  I  V  L  M  A  S  L  T
acattattggagtga
 T  L  L  E  -
```

- DNA sequence and protein for the largest open reading frame inLocus_4149_Transcript_2/4_Confidence_0.700_Length_2043|Q9VAF0:

```
ATGAATTCCTCAATTTGGGAGTCAGACTCAGCCTTCAGATTTATTTACA
CTGATTTAGTTATGGAATACATACCAGAATATTTCAAAGACTGGCAATATCATCCATGGA
TTTTTTCGGTCTTAGGTTCTATTTTAATTGGATTAAGTGGTGTGTTGCCTTTACTTATAA
TTCCATTGGAAGGAGGAGAACATTTTAAAAGTGGAGGAGGGGGCAAACTTTTGCGCGTAT
TGCTCAGTTTTGCAGTAGGAGGTCTGCTCGGAGATGTGTTTTTGCATTTGTTGCCAGAAG
CATGGGAGAACTACAGTAACAAAGGAGAGCGACTCGTAGTCATGTACGAATTCGTAGATT
CAGGAGGAGGTAGTCCATCAATGACATGCGGGTGCTGGGTATTAGCTGGCCTCTTGTTAT
TCACGATGGTCGAGAAGTTATTTGCTGGTTACATGGAAGATGATGACGTTTCCGAGGAAA
AGACCGAACGAAAGCGCAAGAAAATGTCTAAAATTAATTGTTTGATAAAGAACAACAATG
TCTTGAGGGCTAGAAACGAAAATGGACGATTCTCGACCAGCAAATGCTCAGGCGGACAAA
ATGGAAAAATGTGTGACATTGAAGTTATAAATGGAGCTATCAAAAACGGCTGTTGTATGG
TCGAGGAGCTAGAGAAAGAGGCTGTGAGCAACAAGTCTAAGCATGTTTCTGGATACTTAA
ATCTCATGGCAAACTCAATTGATAATTTCACACACGGTCTTGCTGTTGGTGGTTCTTTCA
TTGTTTCATTCAAACTAGGAATTTTAACAACATTCGCAATTCTAGTGCATGAGATTCCAC
ACGAAGTGGGCGATTTTGCAATTTTGTTGCGGTCAGGTTTCTCGCGGTGGGACGCGGCTC
GAGCGCAACTCATGACCGCTGGCGCCGGCCTGCTTGGAGCTATGACCGCAATCGGCGGAA
GTCGGGCTACGAGTGCAATGGAAGCTAGAACATGCTGGATAATGCCCTTCACAGCCGGCG
GATTTCTCCACATAAGTTTAGTTACAGTGCTACCTGACTTGCTGCAAGAAAGGGATCCAA
AAGAGTCGATTAAACAATTTGGAGCATTGATTGGTGGTATAGTTCTAATGGCATCATTGA
CCACATTATTGGAGTGA
```

```
MNSSIWESDSAFRFIYTDLVMEYIPEYFKDWQYHPWIFSVLGSILIGLSGVLPLLIIPLE
GGEHFKSGGGGKLLRVLLSFAVGGLLGDVFLHLLPEAWENYSNKGERLVVMYEFVDSGGG
SPSMTCGCWVLAGLLLFTMVEKLFAGYMEDDDVSEEKTERKRKKMSKINCLIKNNNVLRA
RNENGRFSTSKCSGGQNGKMCDIEVINGAIKNGCCMVEELEKEAVSNKSKHVSGYLNLMA
NSIDNFTHGLAVGGSFIVSFKLGILTTFAILVHEIPHEVGDFAILLRSGFSRWDAARAQL
MTAGAGLLGAMTAIGGSRATSAMEARTCWIMPFTAGGFLHISLVTVLPDLLQERDPKESI
KQFGALIGGIVLMASLTTLLE-
```

- Alignment to the reference protein Q9VAF0:

**Score**	**Expect**	**Method**	**Identities**	**Positives**	**Gaps**
402 bits(1033)	8e-143	Compositional matrix adjust.	221/385(57%)	269/385(69%)	39/385(10%)

```
Query  4    NSSFF--DEHIAMIYSNLMDQYMPEYFKSFEYTPWVFSLLGSVVIGLSGIFPLIIIPTEE  61
            NSS +  D     IY++L+ +Y+PEYFK ++Y PW+FS+LGS++IGLSG+ PL+IIP E
Sbjct  2    NSSIWESDSAFRFIYTDLVMEYIPEYFKDWQYHPWIFSVLGSILIGLSGVLPLLIIPLE-  60

Query  62   KMAKEGYKDPADSKLLRVLLSFAVGGLLGDVFLHLLPEAWEGDNQ-------------DP  108
                E +K     KLLRVLLSFAVGGLLGDVFLHLLPEAWE  +
Sbjct  61   --GGEHFKSGGGGKLLRVLLSFAVGGLLGDVFLHLLPEAWENYSNKGERLVVMYEFVDSG  118

Query  109  SSHPSLRSGLWVLSGILIFTIVEKIFSGYASAD---EENPQPKCVEIA--NCLLRRHGGQ  163
               PS+  G WVL+G+L+FT+VEK+F+GY   D   EE  + K  +++  NCL++ +
Sbjct  119  GGSPSMTCGCWVLAGLLLFTMVEKLFAGYMEDDDVSEEKTERKRKKMSKINCLIKNNNVL  178

Query  164  LPEGE-----TSESCGGA----CDIEDVGK-----VCFLREQEQKSKERKEQPKKVAGYL  209
                E     TS+  GG     CDIE +        C + E E+++   K   K V+GYL
Sbjct  179  RARNENGRFSTSKCSGGQNGKMCDIEVINGAIKNGCCMVEELEKEAVSNKS--KHVSGYL  236

Query  210  NLLANSIDNFTHGLAVAGSFLVSFRHGILATFAILLHEIPHEVGDFAILLRSGFSRWDAA  269
            NL+ANSIDNFTHGLAV GSF+VSF+ GIL TFAIL+HEIPHEVGDFAILLRSGFSRWDAA
Sbjct  237  NLMANSIDNFTHGLAVGGSFIVSFKLGILTTFAILVHEIPHEVGDFAILLRSGFSRWDAA  296
```

```
Query  270  RAQLLTAGAGLLGALVAIGGSGVTSAMEARTSWIMPFTAGGFLHIALVTVLPDLLKEEER  329
            RAQL+TAGAGLLGA+ AIGGS  TSAMEART WIMPFTAGGFLHI+LVTVLPDLL+E +
Sbjct  297  RAQLMTAGAGLLGAMTAIGGSRATSAMEARTCWIMPFTAGGFLHISLVTVLPDLLQERDP  356

Query  330  KESIKQLLALVFGIALMAVMTMLFE  354
            KESIKQ  AL+ GI LMA +T L E
Sbjct  357  KESIKQFGALIGGIVLMASLTTLLE  381
```

3. Sequence used for synthesis and expression.

```
ATGAATTCCTCAATTTGGGAGTCAGACTCAGCCTTCAGATTTATTTACACTGATTTAGTTATGGAATA
CATACCAGAATATTTCAAAGACTGGCAATATCATCCATGGATTTTTTCGGTCTTAGGTTCTATTTTAA
TTGGATTAAGTGGTGTGTTGCCTTTACTTATAATTCCATTGGAAGGAGGAGAACATTTTAAAAGTGGA
GGAGGGGGCAAACTTTTGCGCGTATTGCTCAGTTTTGCAGTAGGAGGTCTGCTCGGAGATGTGTTTTT
GCATTTGTTGCCAGAAGCATGGGAGAACTACAGTAACAAAGGAGAGCGACTCGTAGTCATGTACGAAT
TCGTAGATTCAGGAGGAGGTAGTCCATCAATGACATGCGGGTGCTGGGTATTAGCTGGCCTCTTGTTA
TTCACGATGGTCGAGAAGTTATTTGCTGGTTACATGGAAGATGATGACGTTTCCGAGGAAAAGACCGA
ACGAAAGCGCAAGAAAATGTCTAAAATTAATTGTTTGATAAAGAACAACAATGTCTTGAGGGCTAGAA
ACGAAAATGGACGATTCTCGACCAGCAAATGCTCAGGCGGACAAAATGGAAAAATGTGTGACATTGAA
GTTATAAATGGAGCTATCAAAAACGGCTGTTGTATGGTCGAGGAGCTAGAGAAAGAGGCTGTGAGCAA
CAAGTCTAAGCATGTTTCTGGATACTTAAATCTCATGGCAAACTCAATTGATAATTTCACACACGGTC
TTGCTGTTGGTGGTTCTTTCATTGTTTCATTCAAACTAGGAATTTTAACAACATTCGCAATTCTAGTG
CATGAGATTCCACACGAAGTGGGCGATTTTGCAATTTTGTTGCGGTCAGGTTTCTCGCGGTGGGACGC
GGCTCGAGCGCAACTCATGACCGCTGGCGCCGGCCTGCTTGGAGCTATGACCGCAATCGGCGGAAGTC
GGGCTACGAGTGCAATGGAAGCTAGAACATGCTGGATAATGCCCTTCACAGCCGGCGGATTTCTCCAC
ATAAGTTTAGTTACAGTGCTACCTGACTTGCTGCAAGAAAGGGATCCAAAAGAGTCGATTAAACAATT
TGGAGCATTGATTGGTGGTATAGTTCTAATGGCATCATTGACCACATTATTGGAGTGA
```
